# Supplementary figures and images for: HUWE1 controls tristetraprolin proteasomal degradation by regulating its phosphorylation (part 1 of 4)
Source: eLife. 2023 Mar 24;12:e83159. doi: 10.7554/eLife.83159 (PMC10038661; doi:10.7554/eLife.83159)

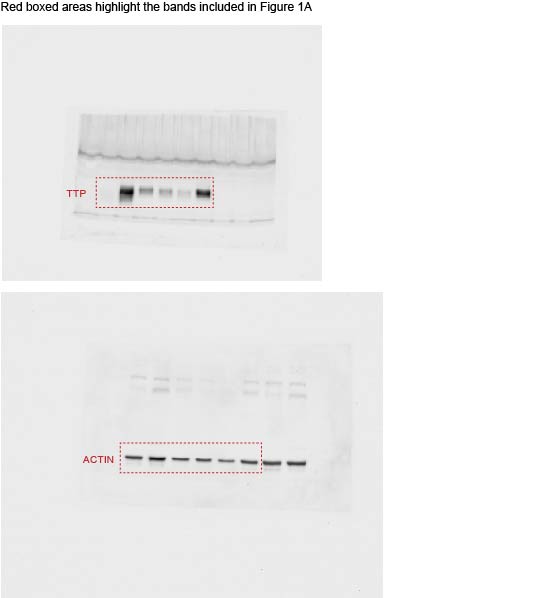

Supplement: Figure 1—source data 1. [file elife-83159-fig1-data1.zip › Figure 1-source data 1/Figure 1-source data 1.jpg]

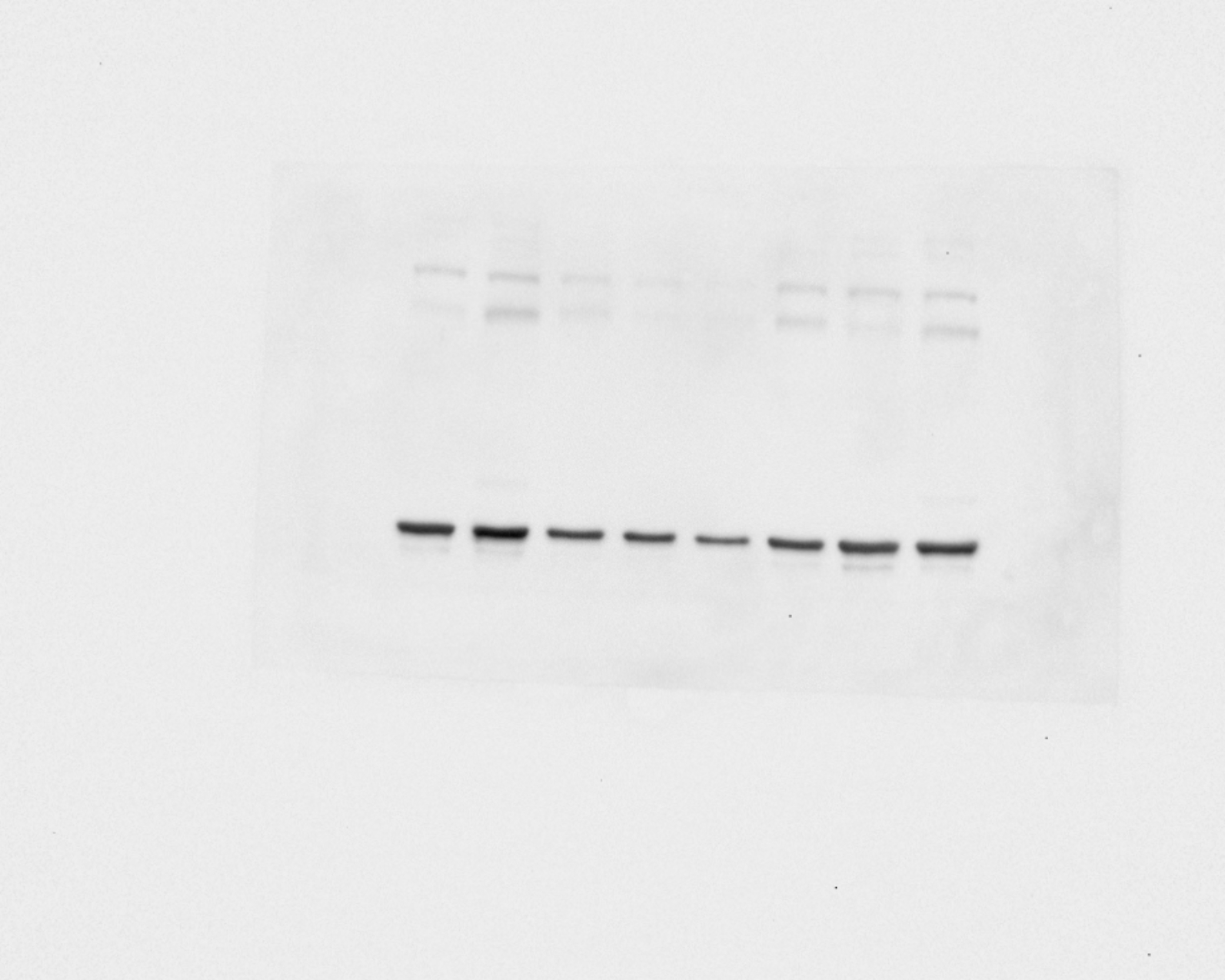

Supplement: Figure 1—source data 1. [file elife-83159-fig1-data1.zip › Figure 1-source data 1/GAPDH Figure 1-source data 1/common 2018-01-25 19h41m52s(Chemiluminescence).jpg]

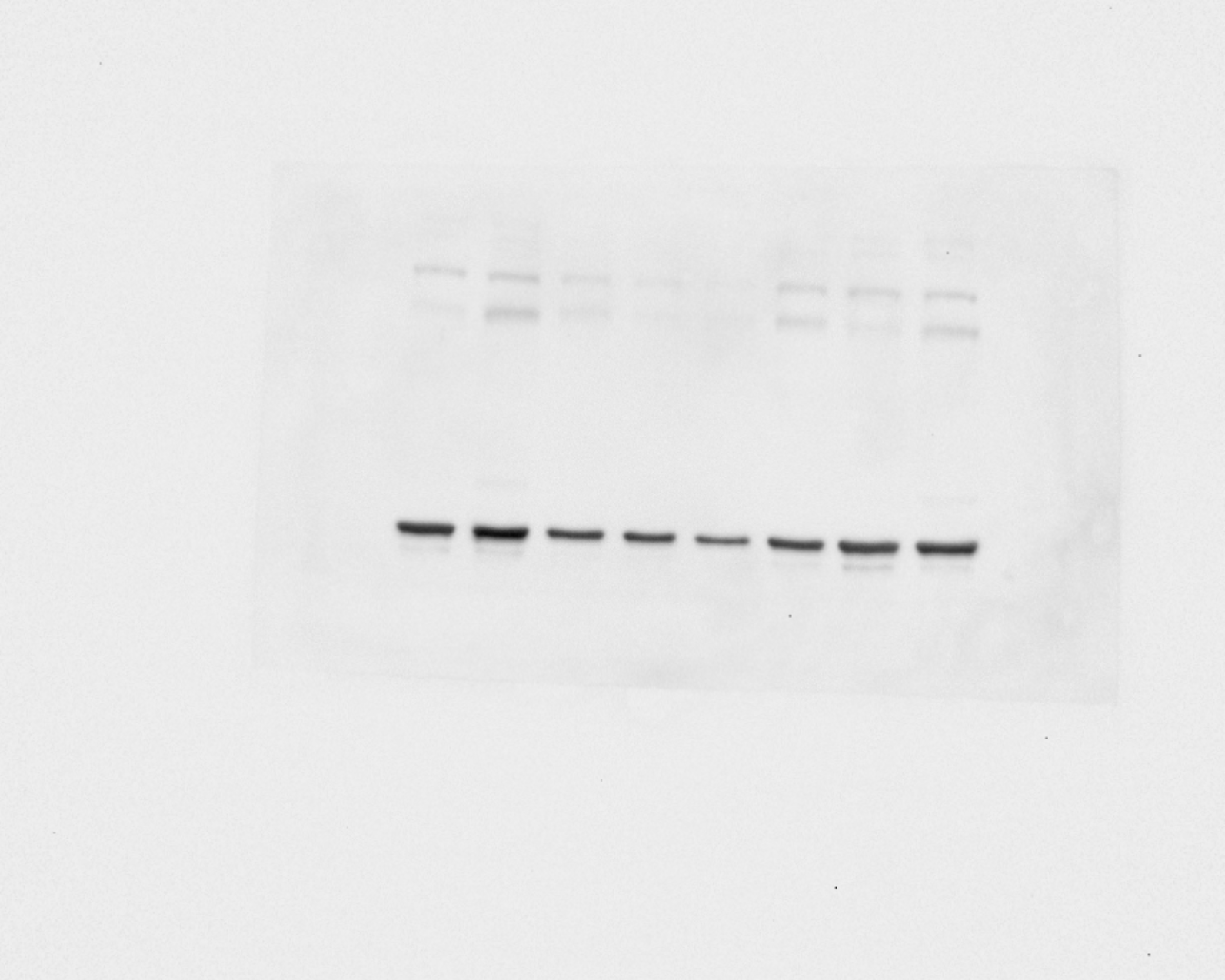

Supplement: Figure 1—source data 1. [file elife-83159-fig1-data1.zip › Figure 1-source data 1/GAPDH Figure 1-source data 1/common 2018-01-25 19h41m52s(Chemiluminescence).tif]

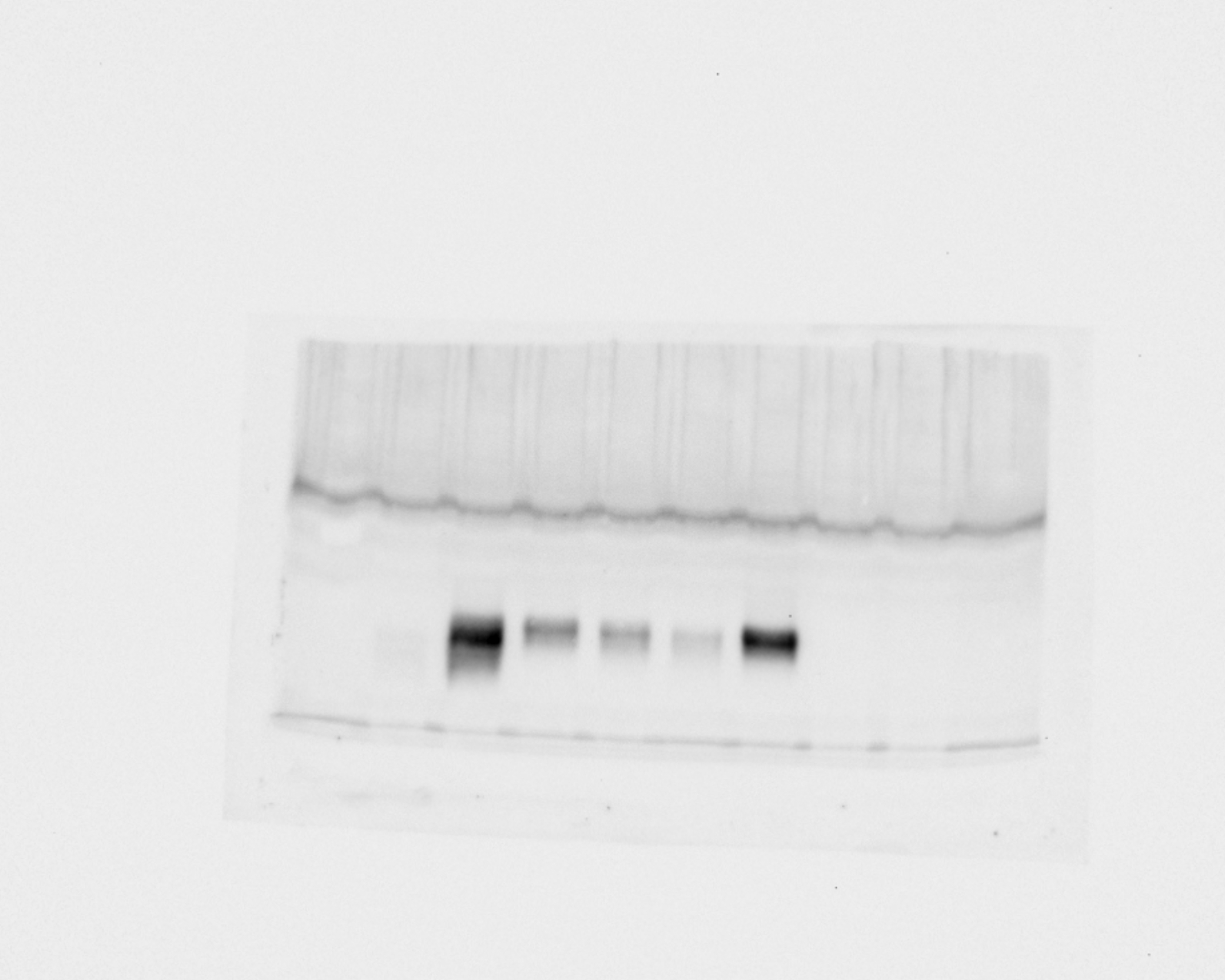

Supplement: Figure 1—source data 1. [file elife-83159-fig1-data1.zip › Figure 1-source data 1/TTP Figure 1-source data 1/common 2018-01-25 11h46m43s(Chemiluminescence).jpg]

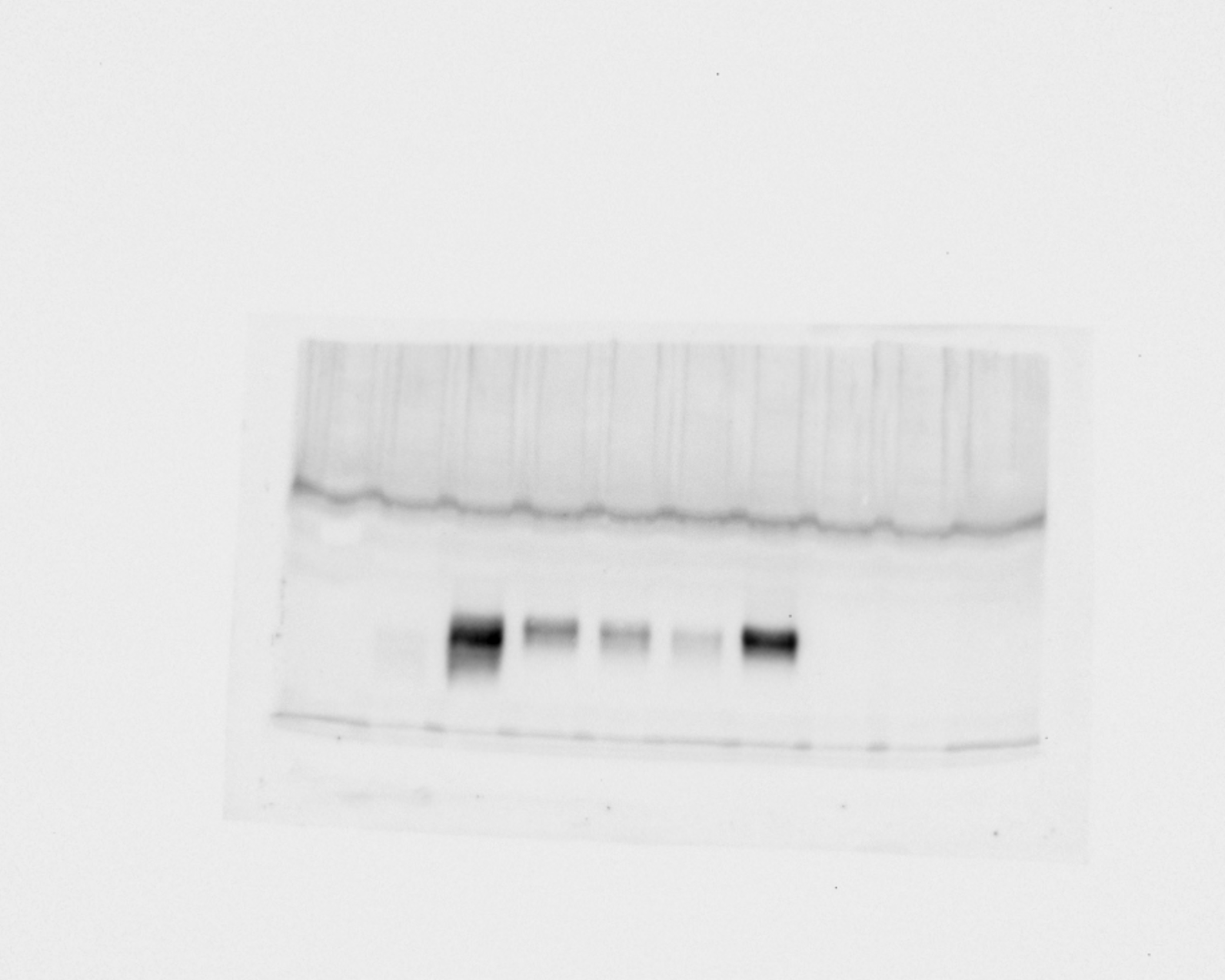

Supplement: Figure 1—source data 1. [file elife-83159-fig1-data1.zip › Figure 1-source data 1/TTP Figure 1-source data 1/common 2018-01-25 11h46m43s(Chemiluminescence).tif]

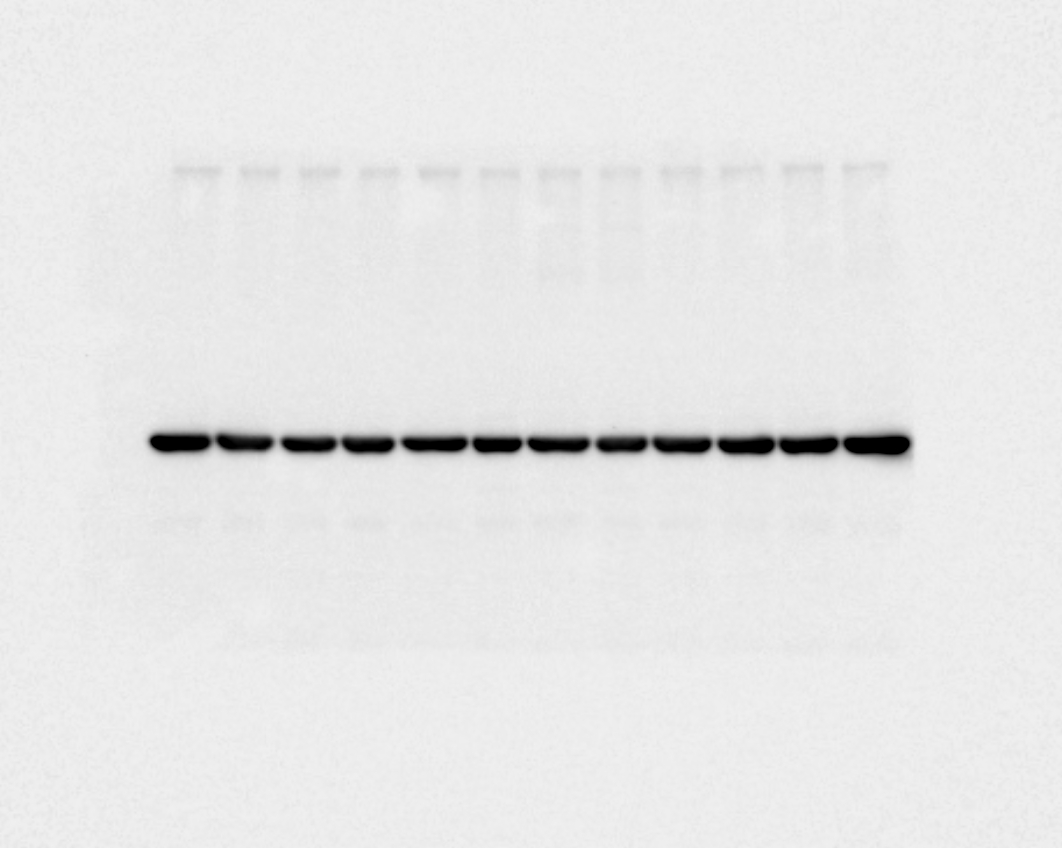

Supplement: Figure 1—source data 2. [file elife-83159-fig1-data2.zip › Figure 1-source data 2/ACTIN_1 Figure 1-source data 2/Versteeg 2021-12-07 17h49m07s 10.000s(Chemiluminescence).jpg]

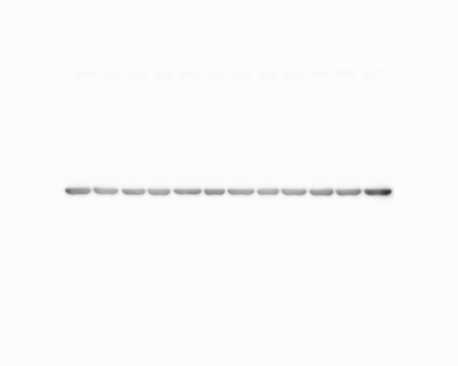

Supplement: Figure 1—source data 2. [file elife-83159-fig1-data2.zip › Figure 1-source data 2/ACTIN_1 Figure 1-source data 2/Versteeg 2021-12-07 17h49m07s 10.000s(Chemiluminescence).raw16.tif]

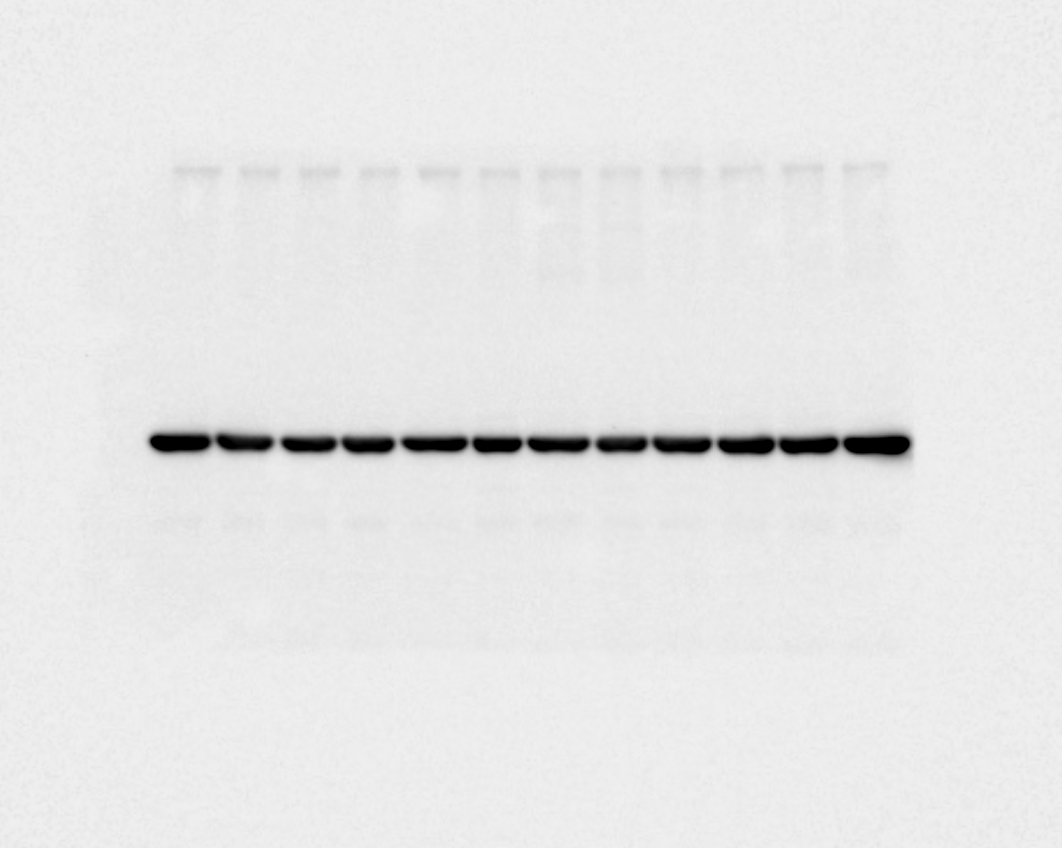

Supplement: Figure 1—source data 2. [file elife-83159-fig1-data2.zip › Figure 1-source data 2/ACTIN_1 Figure 1-source data 2/Versteeg 2021-12-07 17h49m07s 10.000s(Chemiluminescence).tif]

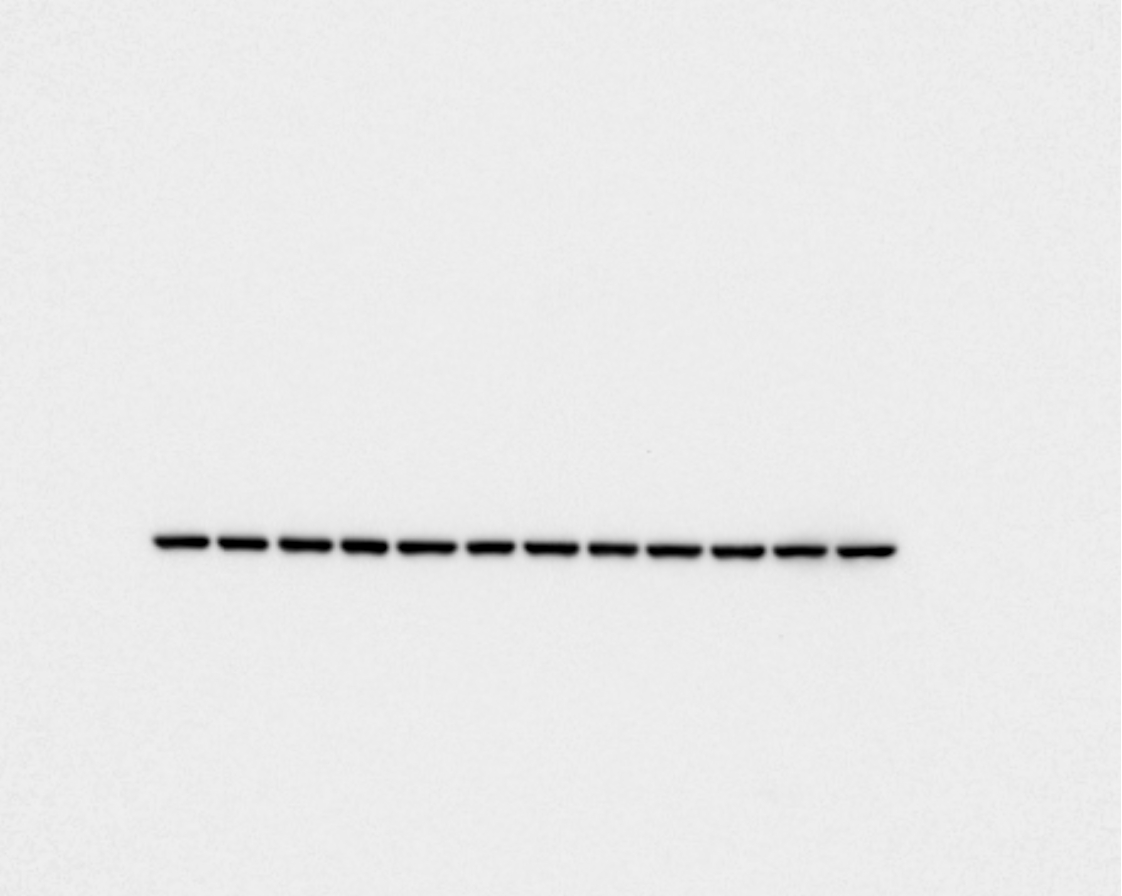

Supplement: Figure 1—source data 2. [file elife-83159-fig1-data2.zip › Figure 1-source data 2/ACTIN_2 Figure 1-source data 2/Versteeg 2021-12-03 18h37m49s 7.102s(Chemiluminescence).jpg]

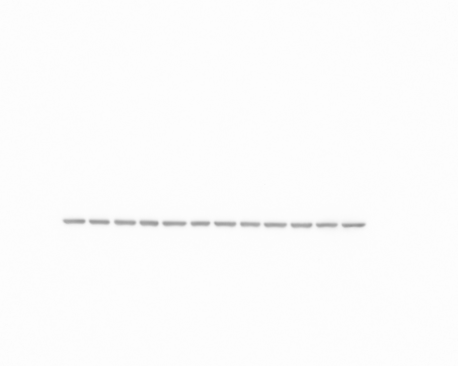

Supplement: Figure 1—source data 2. [file elife-83159-fig1-data2.zip › Figure 1-source data 2/ACTIN_2 Figure 1-source data 2/Versteeg 2021-12-03 18h37m49s 7.102s(Chemiluminescence).raw16.tif]

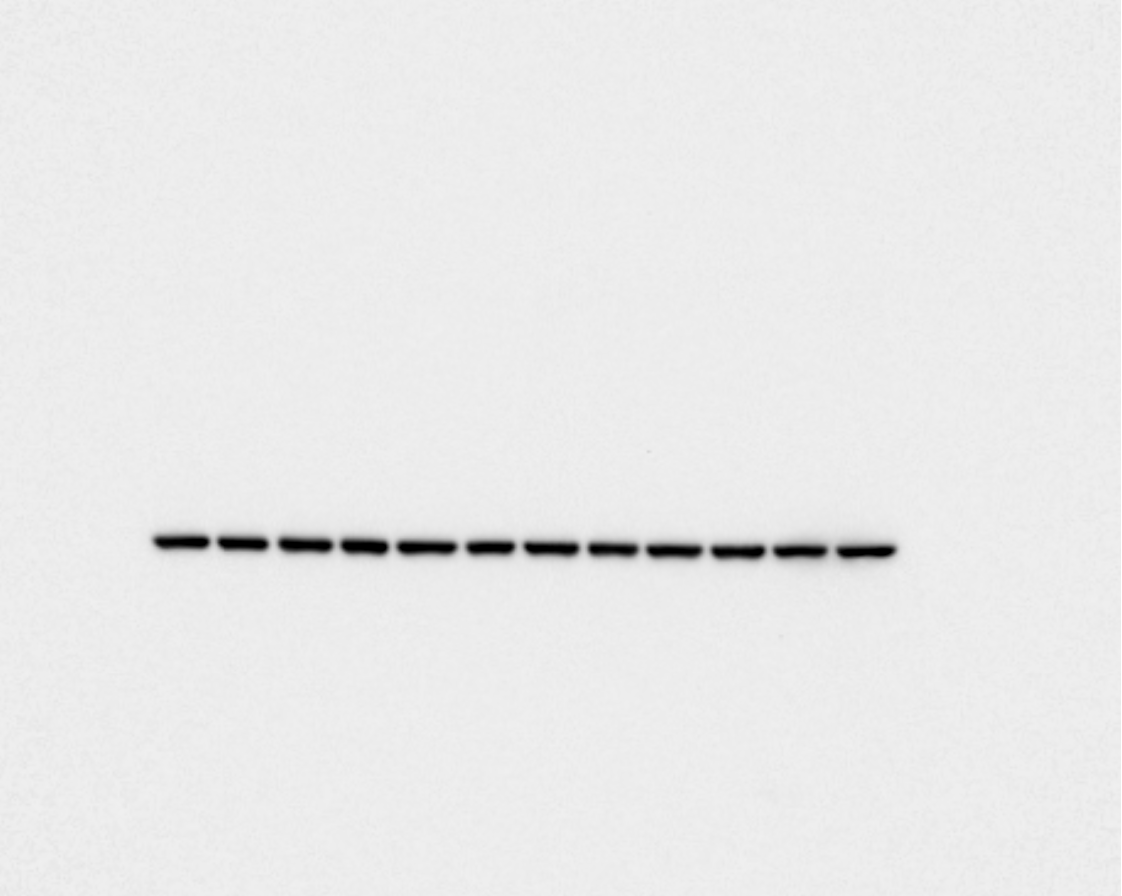

Supplement: Figure 1—source data 2. [file elife-83159-fig1-data2.zip › Figure 1-source data 2/ACTIN_2 Figure 1-source data 2/Versteeg 2021-12-03 18h37m49s 7.102s(Chemiluminescence).tif]

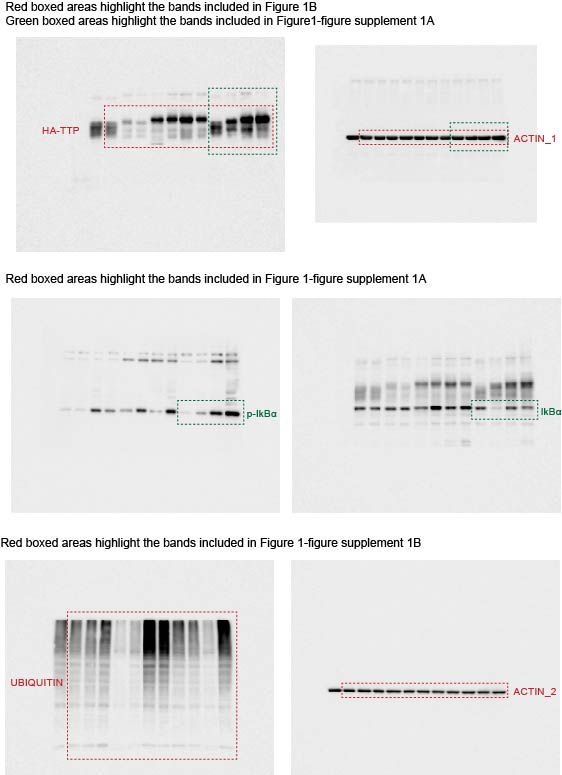

Supplement: Figure 1—source data 2. [file elife-83159-fig1-data2.zip › Figure 1-source data 2/Figure 1-source data 2.jpg]

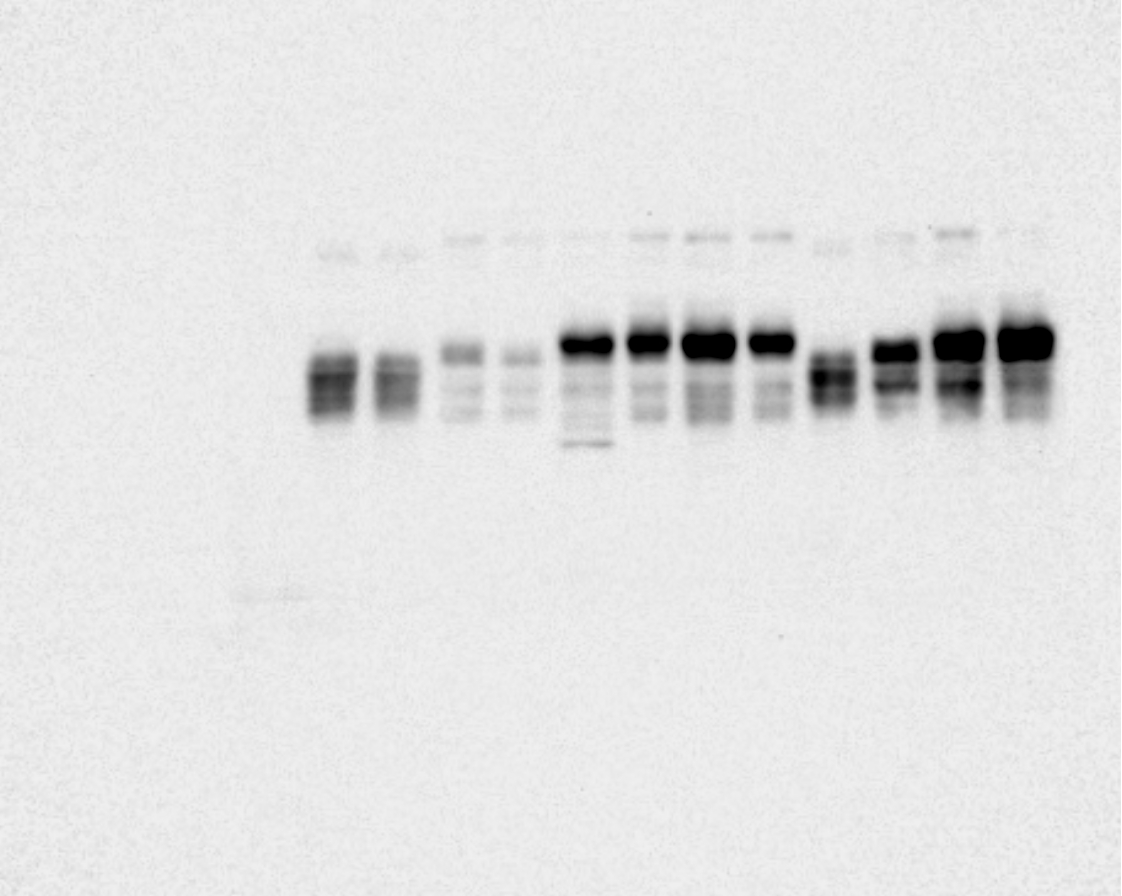

Supplement: Figure 1—source data 2. [file elife-83159-fig1-data2.zip › Figure 1-source data 2/HA-TTP Figure 1-source data 2/Versteeg 2021-12-03 13h41m09s 75.760s(Chemiluminescence).jpg]

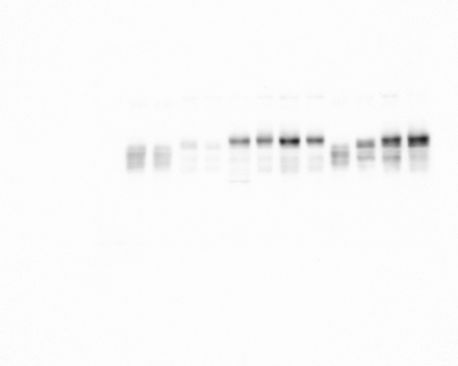

Supplement: Figure 1—source data 2. [file elife-83159-fig1-data2.zip › Figure 1-source data 2/HA-TTP Figure 1-source data 2/Versteeg 2021-12-03 13h41m09s 75.760s(Chemiluminescence).raw16.tif]

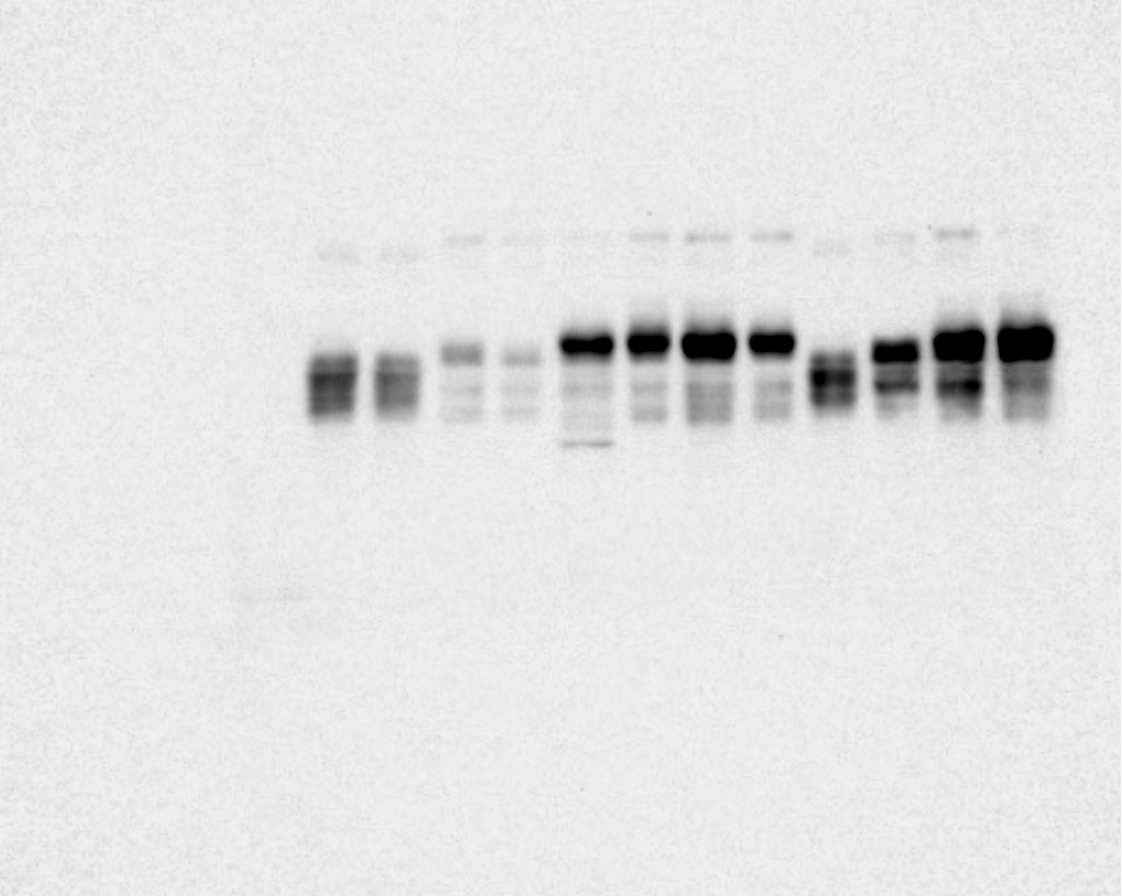

Supplement: Figure 1—source data 2. [file elife-83159-fig1-data2.zip › Figure 1-source data 2/HA-TTP Figure 1-source data 2/Versteeg 2021-12-03 13h41m09s 75.760s(Chemiluminescence).tif]

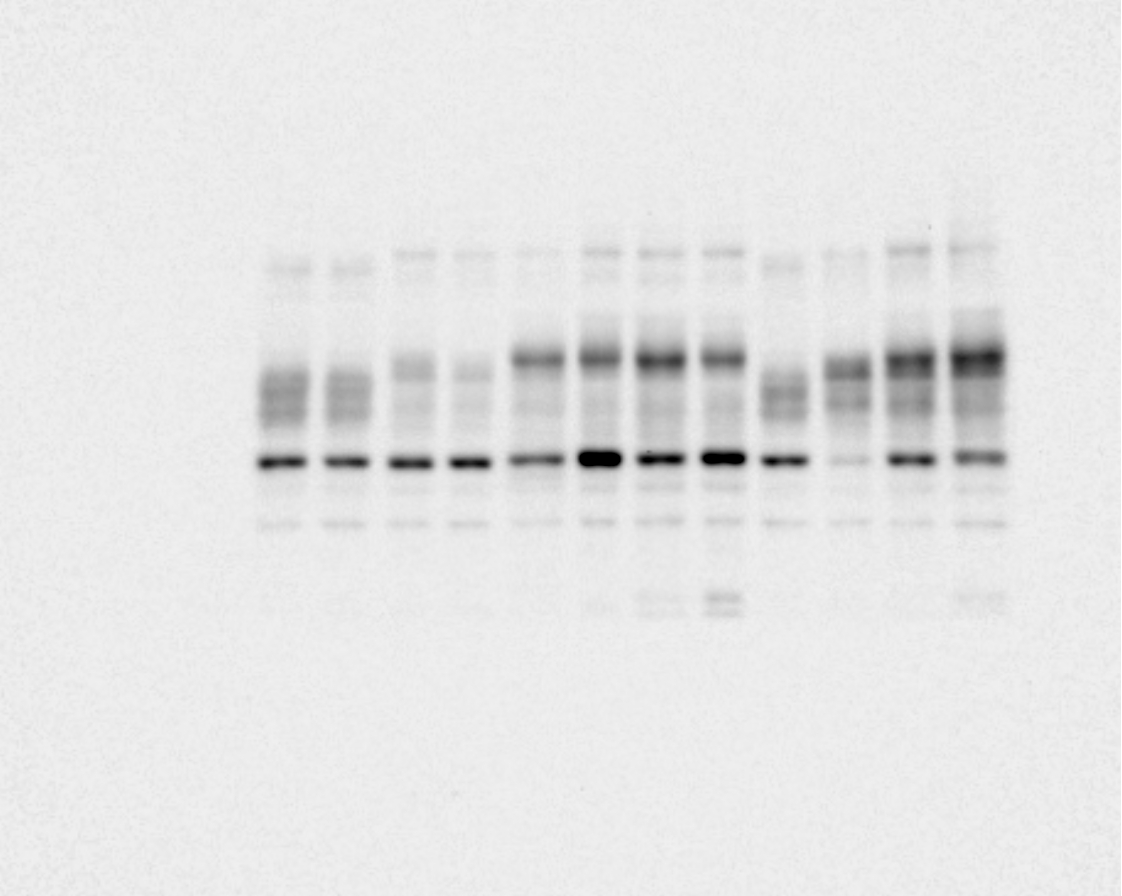

Supplement: Figure 1—source data 2. [file elife-83159-fig1-data2.zip › Figure 1-source data 2/IkBa Figure 1-source data 2/Versteeg 2021-12-07 02h48m49s 40.330s(Chemiluminescence).jpg]

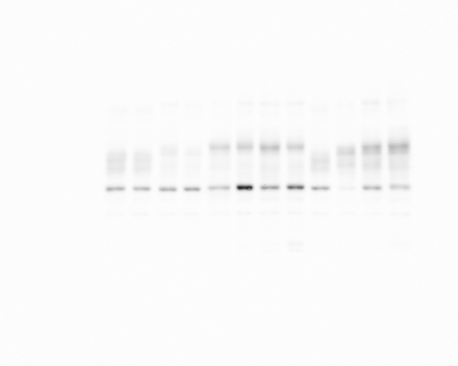

Supplement: Figure 1—source data 2. [file elife-83159-fig1-data2.zip › Figure 1-source data 2/IkBa Figure 1-source data 2/Versteeg 2021-12-07 02h48m49s 40.330s(Chemiluminescence).raw16.tif]

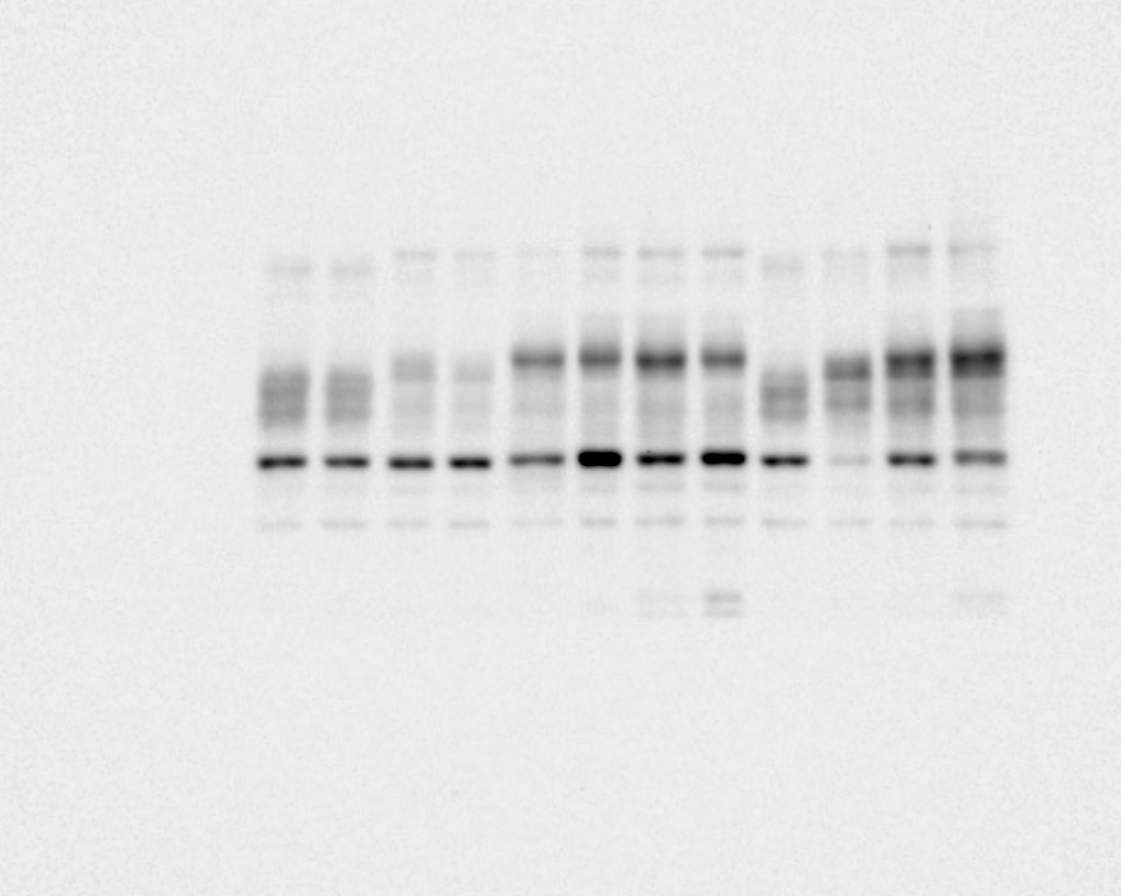

Supplement: Figure 1—source data 2. [file elife-83159-fig1-data2.zip › Figure 1-source data 2/IkBa Figure 1-source data 2/Versteeg 2021-12-07 02h48m49s 40.330s(Chemiluminescence).tif]

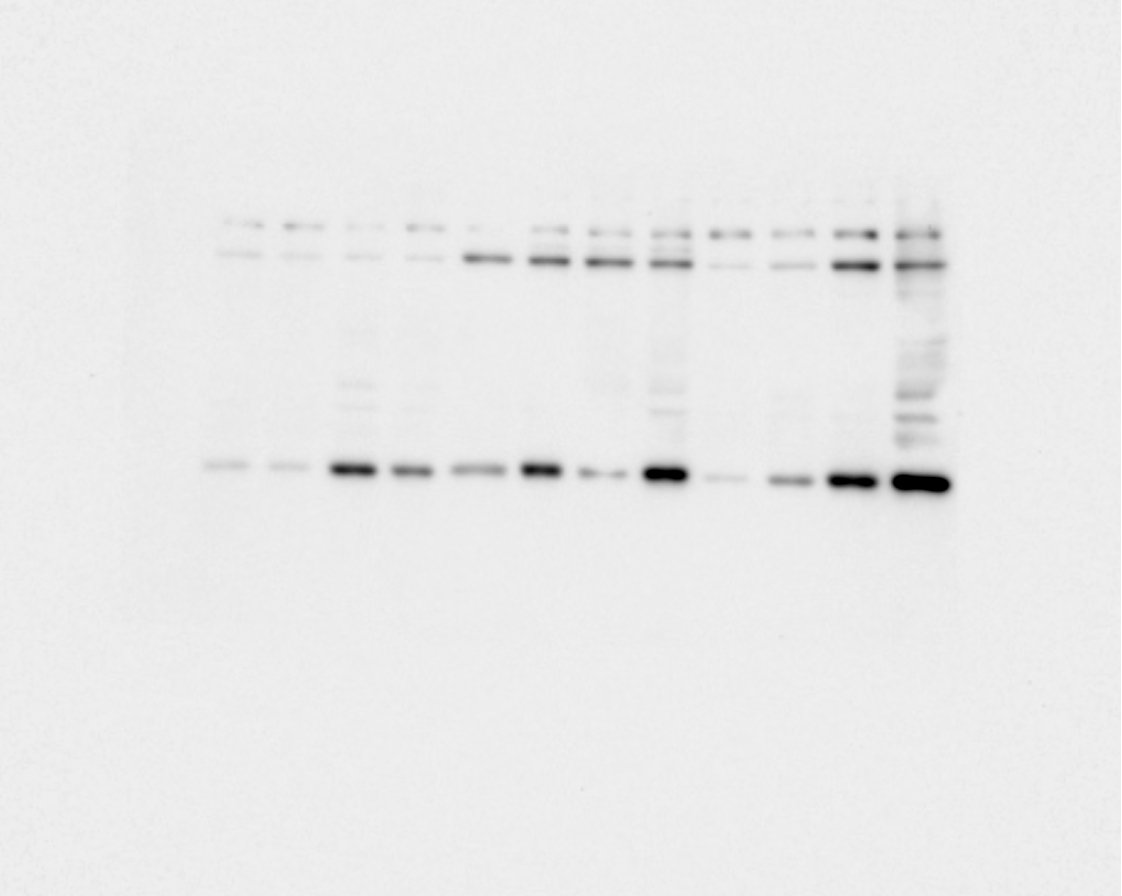

Supplement: Figure 1—source data 2. [file elife-83159-fig1-data2.zip › Figure 1-source data 2/p-IkBa Figure 1-source data 2/Versteeg 2021-12-06 14h49m54s 27.758s(Chemiluminescence).jpg]

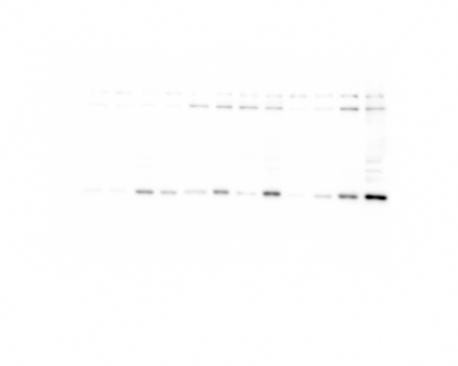

Supplement: Figure 1—source data 2. [file elife-83159-fig1-data2.zip › Figure 1-source data 2/p-IkBa Figure 1-source data 2/Versteeg 2021-12-06 14h49m54s 27.758s(Chemiluminescence).raw16.tif]

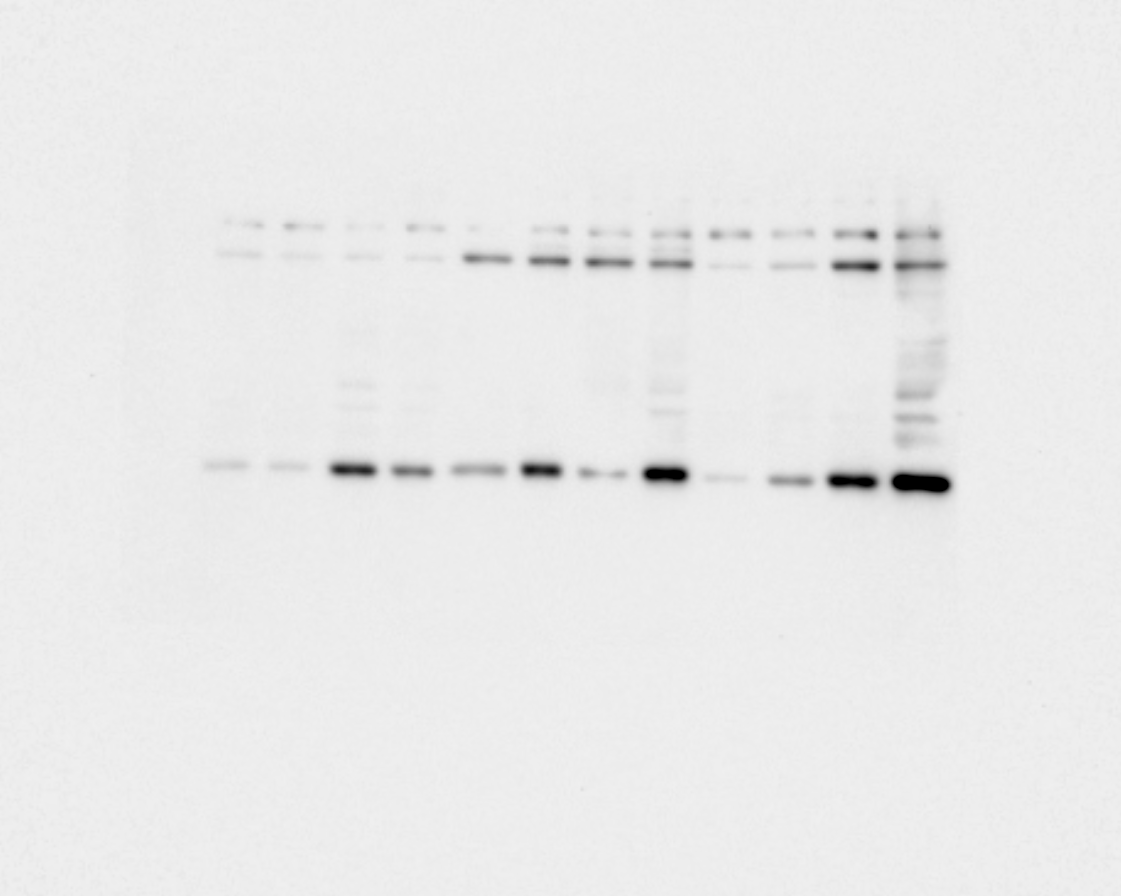

Supplement: Figure 1—source data 2. [file elife-83159-fig1-data2.zip › Figure 1-source data 2/p-IkBa Figure 1-source data 2/Versteeg 2021-12-06 14h49m54s 27.758s(Chemiluminescence).tif]

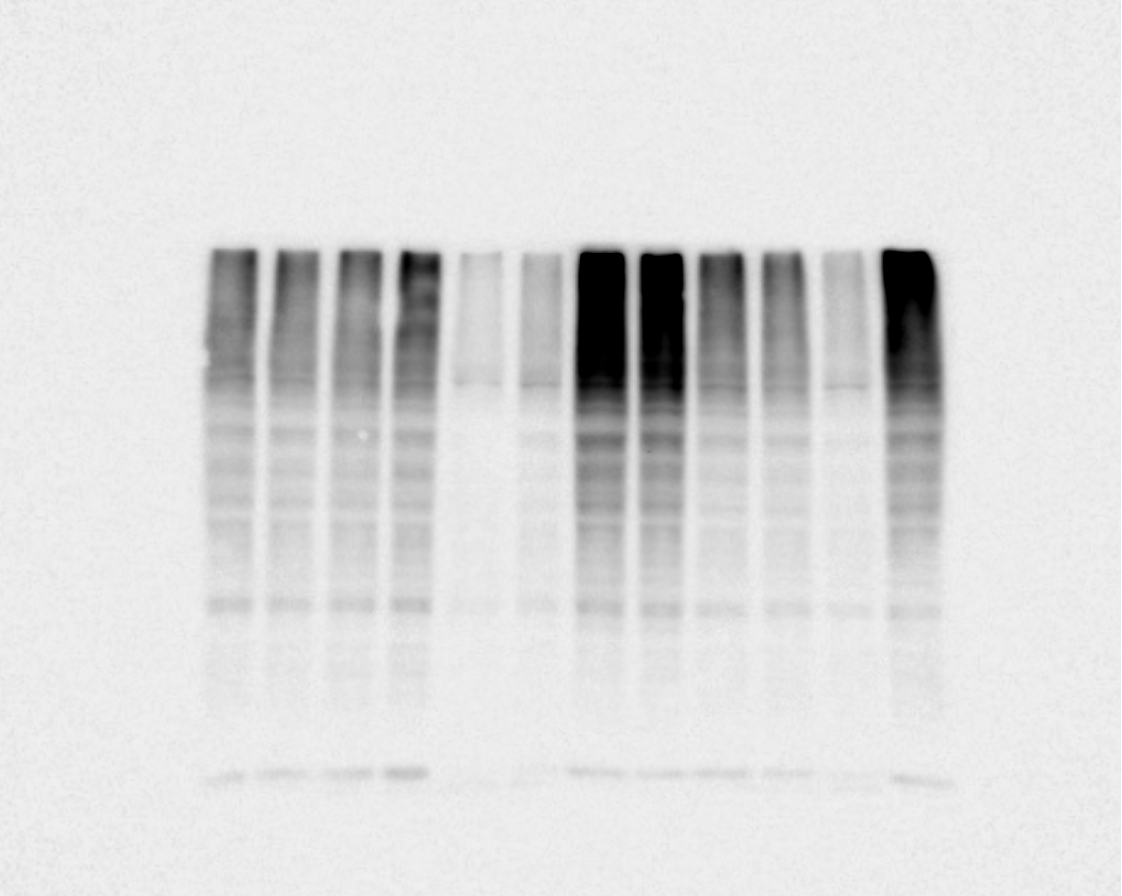

Supplement: Figure 1—source data 2. [file elife-83159-fig1-data2.zip › Figure 1-source data 2/UB Figure 1-source data 2/Versteeg 2021-12-03 13h20m30s 13.096s(Chemiluminescence).jpg]

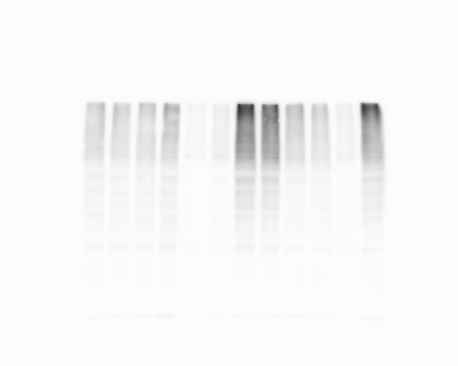

Supplement: Figure 1—source data 2. [file elife-83159-fig1-data2.zip › Figure 1-source data 2/UB Figure 1-source data 2/Versteeg 2021-12-03 13h20m30s 13.096s(Chemiluminescence).raw16.tif]

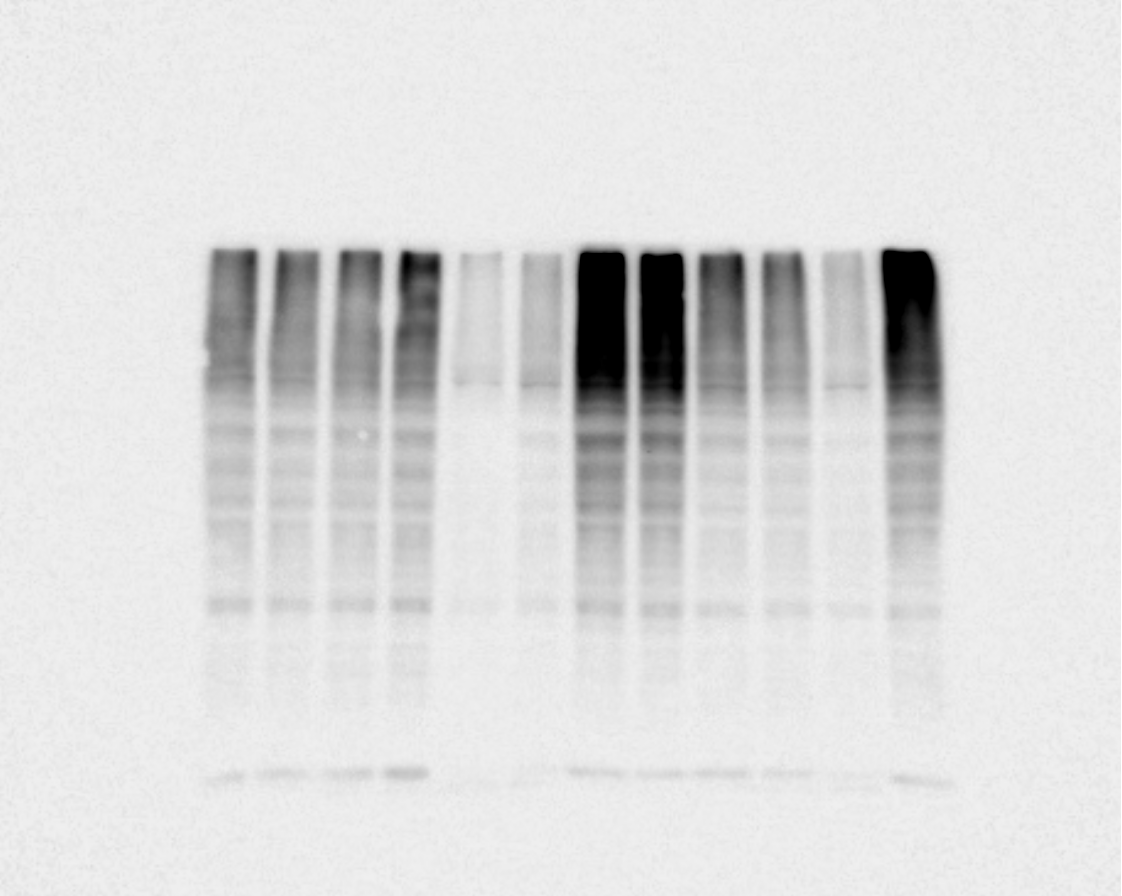

Supplement: Figure 1—source data 2. [file elife-83159-fig1-data2.zip › Figure 1-source data 2/UB Figure 1-source data 2/Versteeg 2021-12-03 13h20m30s 13.096s(Chemiluminescence).tif]

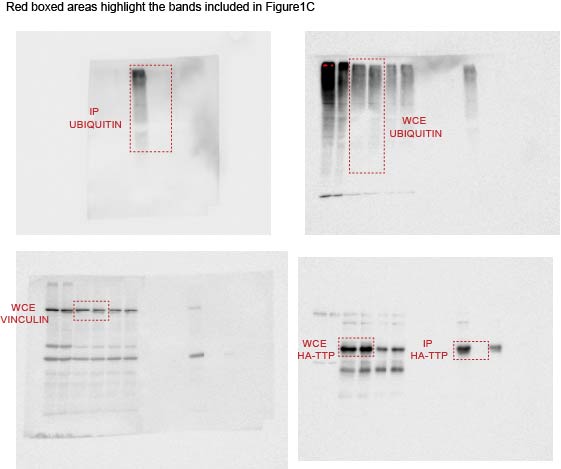

Supplement: Figure 1—source data 3. [file elife-83159-fig1-data3.zip › Figure 1-source data 3/Figure 1-source data 3.jpg]

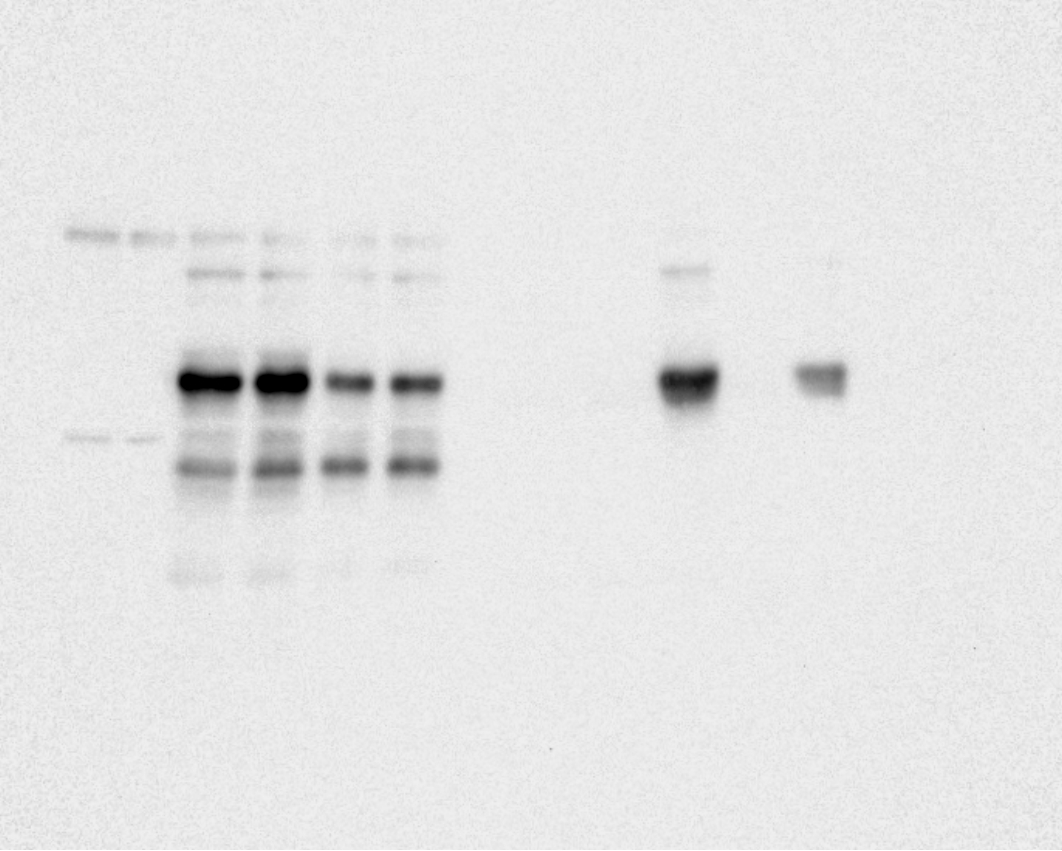

Supplement: Figure 1—source data 3. [file elife-83159-fig1-data3.zip › Figure 1-source data 3/HA-TTP Figure 1-source data 3/Versteeg 2022-01-28 10h34m51s 145.340s(Chemiluminescence).jpg]

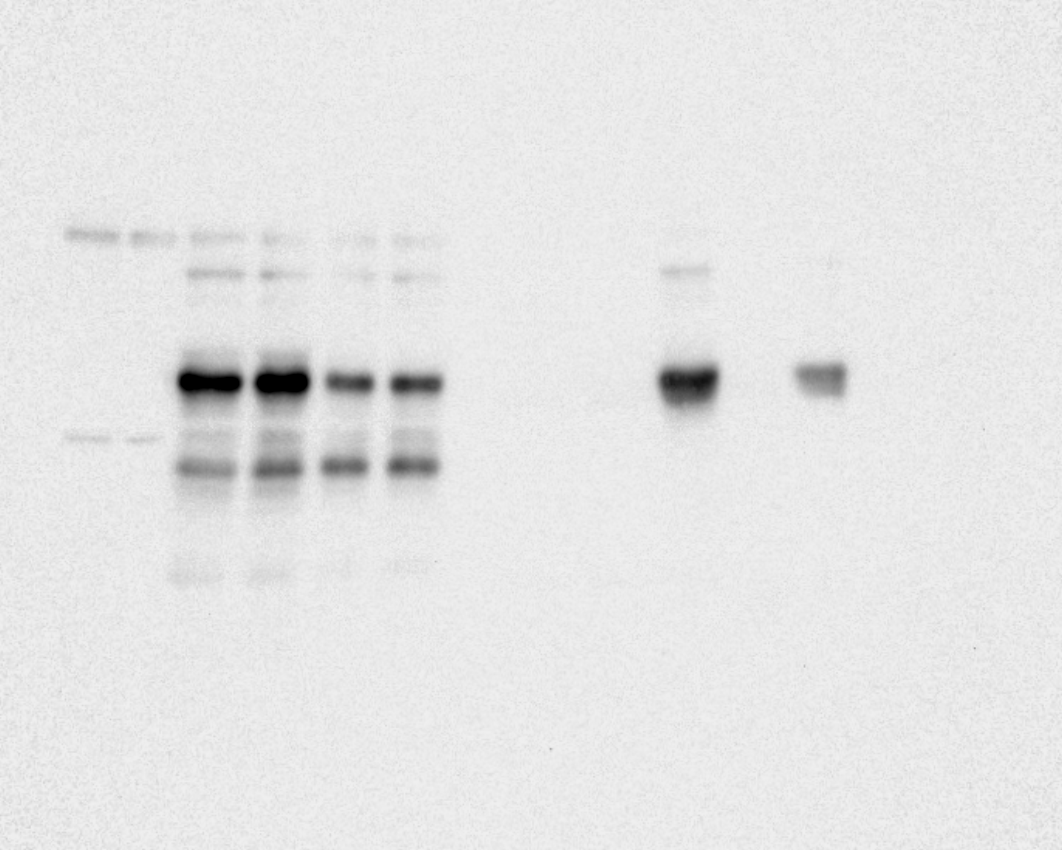

Supplement: Figure 1—source data 3. [file elife-83159-fig1-data3.zip › Figure 1-source data 3/HA-TTP Figure 1-source data 3/Versteeg 2022-01-28 10h34m51s 145.340s(Chemiluminescence).tif]

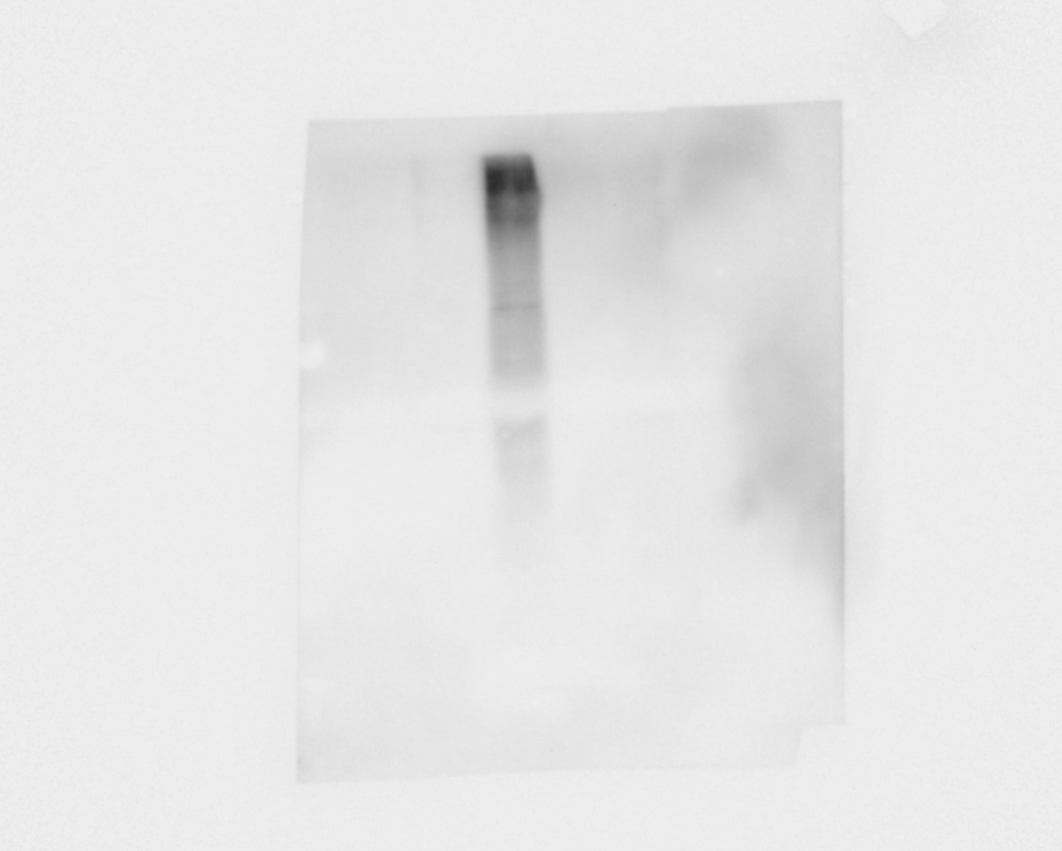

Supplement: Figure 1—source data 3. [file elife-83159-fig1-data3.zip › Figure 1-source data 3/UBIQUITIN IP Figure 1-source data 3/Versteeg 2022-01-27 12h31m18s 21.620s(Chemiluminescence).tif]

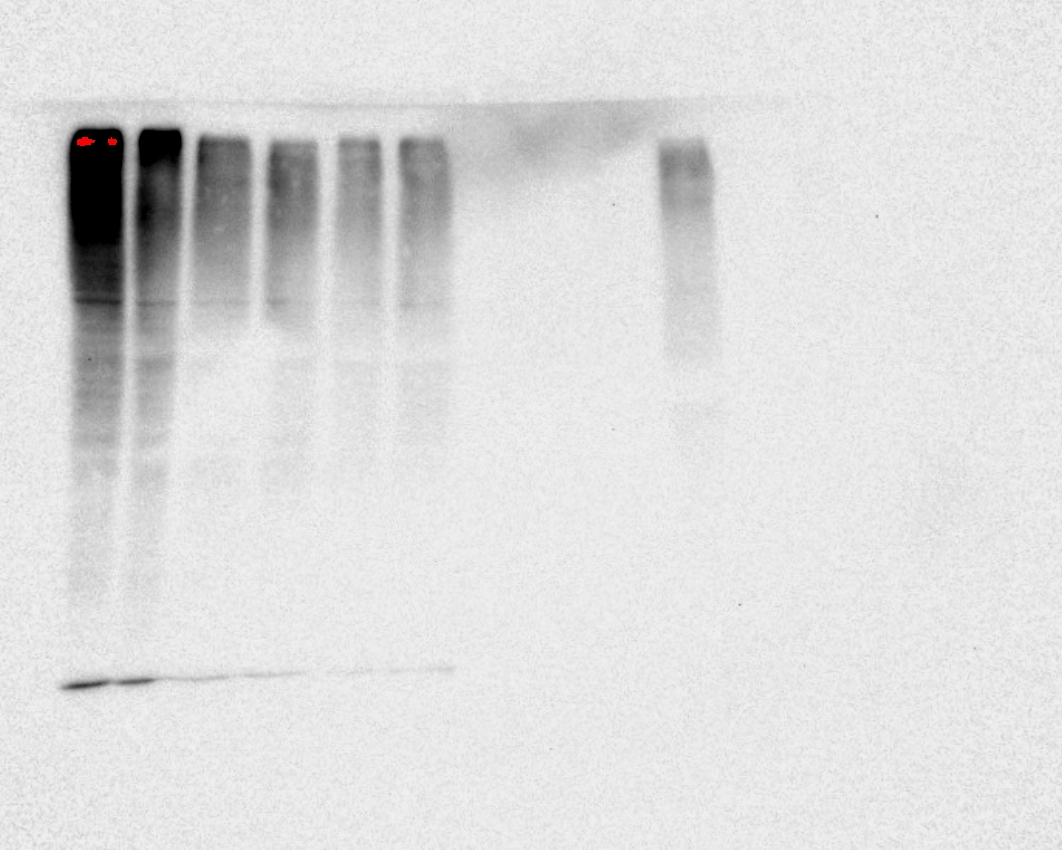

Supplement: Figure 1—source data 3. [file elife-83159-fig1-data3.zip › Figure 1-source data 3/UBIQUITIN WCE Figure 1-source data 3/Versteeg 2022-01-27 11h01m22s 99.990s(Chemiluminescence).jpg]

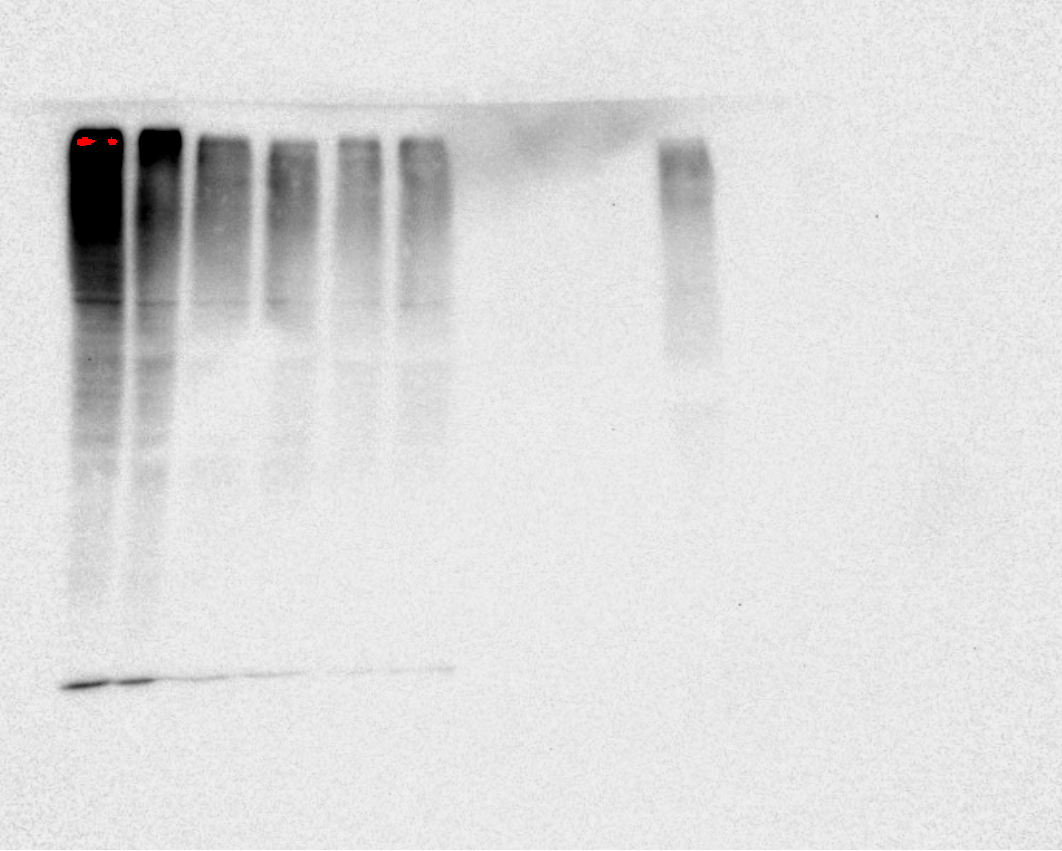

Supplement: Figure 1—source data 3. [file elife-83159-fig1-data3.zip › Figure 1-source data 3/UBIQUITIN WCE Figure 1-source data 3/Versteeg 2022-01-27 11h01m22s 99.990s(Chemiluminescence).tif]

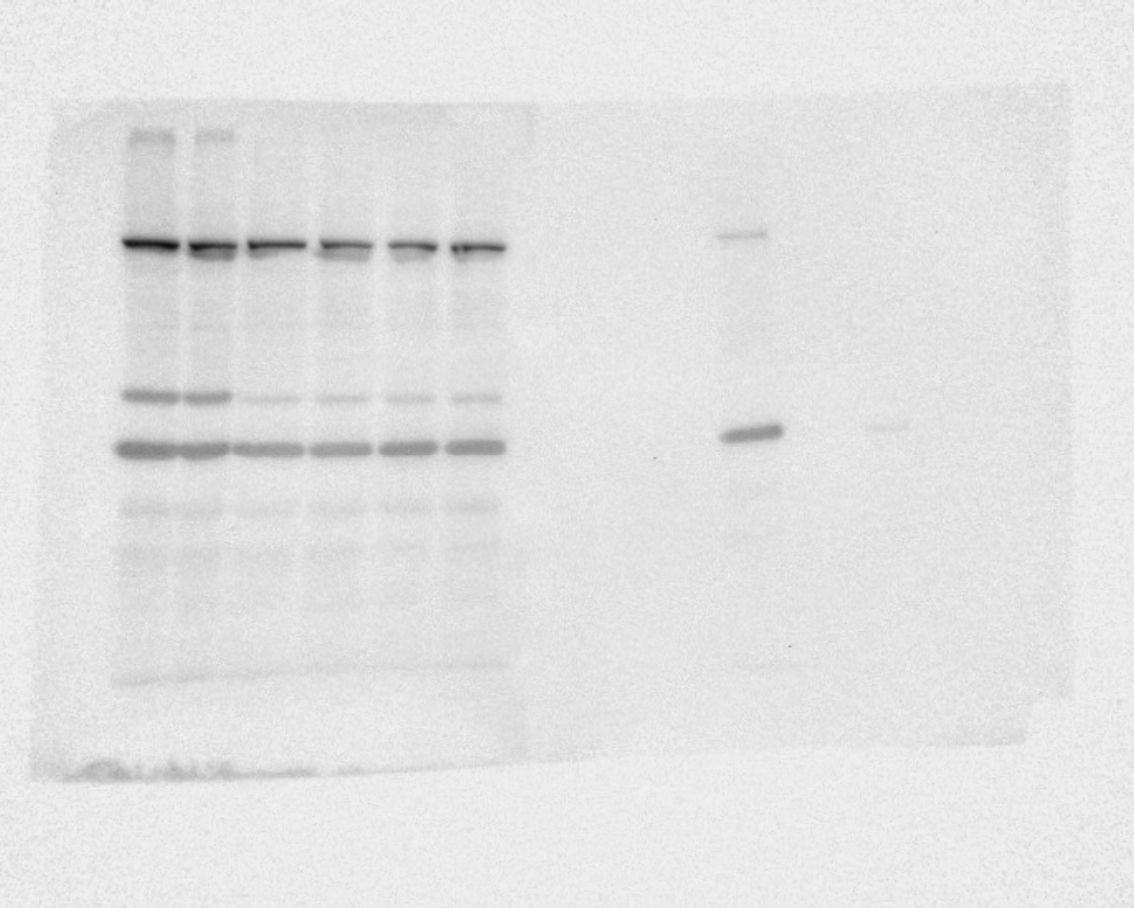

Supplement: Figure 1—source data 3. [file elife-83159-fig1-data3.zip › Figure 1-source data 3/VINCULIN WCE Figure 1-source data 3/Versteeg 2022-02-04 10h46m02s 34.514s(Chemiluminescence).jpg]

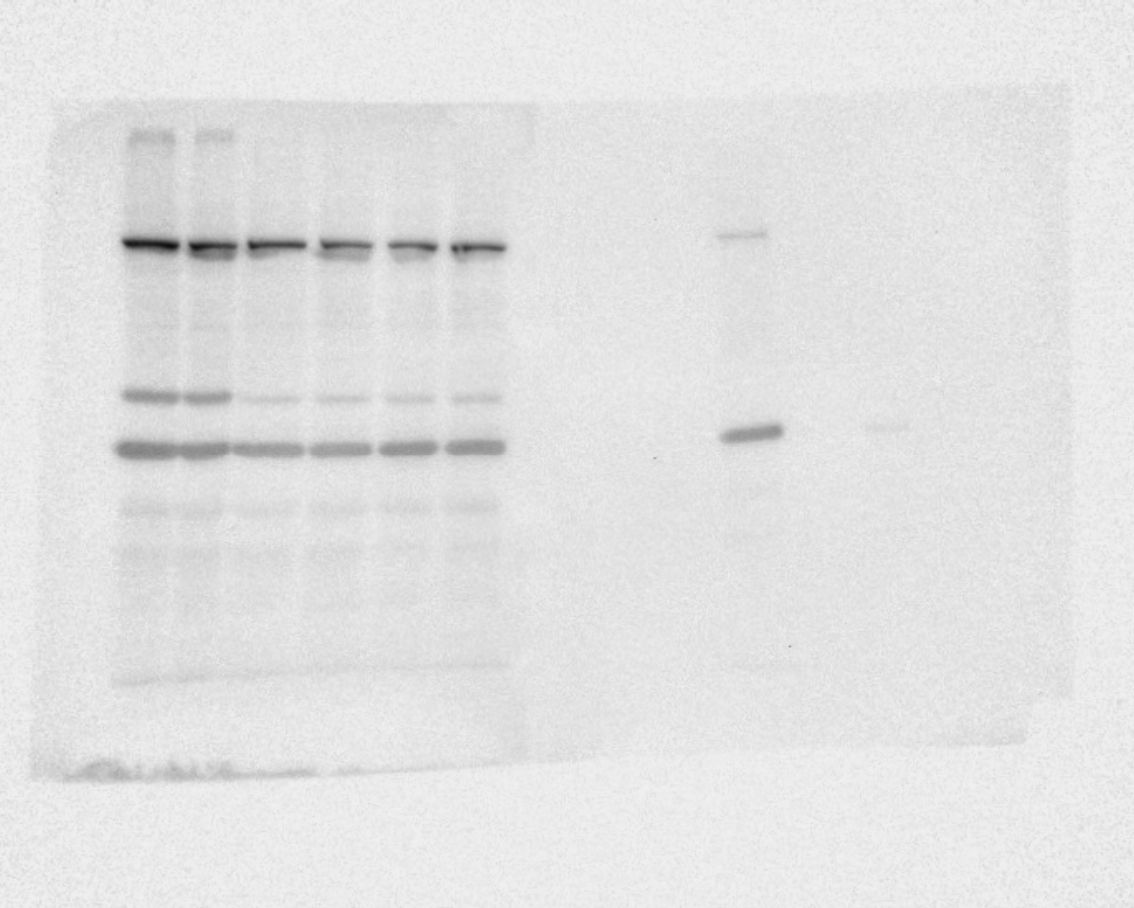

Supplement: Figure 1—source data 3. [file elife-83159-fig1-data3.zip › Figure 1-source data 3/VINCULIN WCE Figure 1-source data 3/Versteeg 2022-02-04 10h46m02s 34.514s(Chemiluminescence).tif]

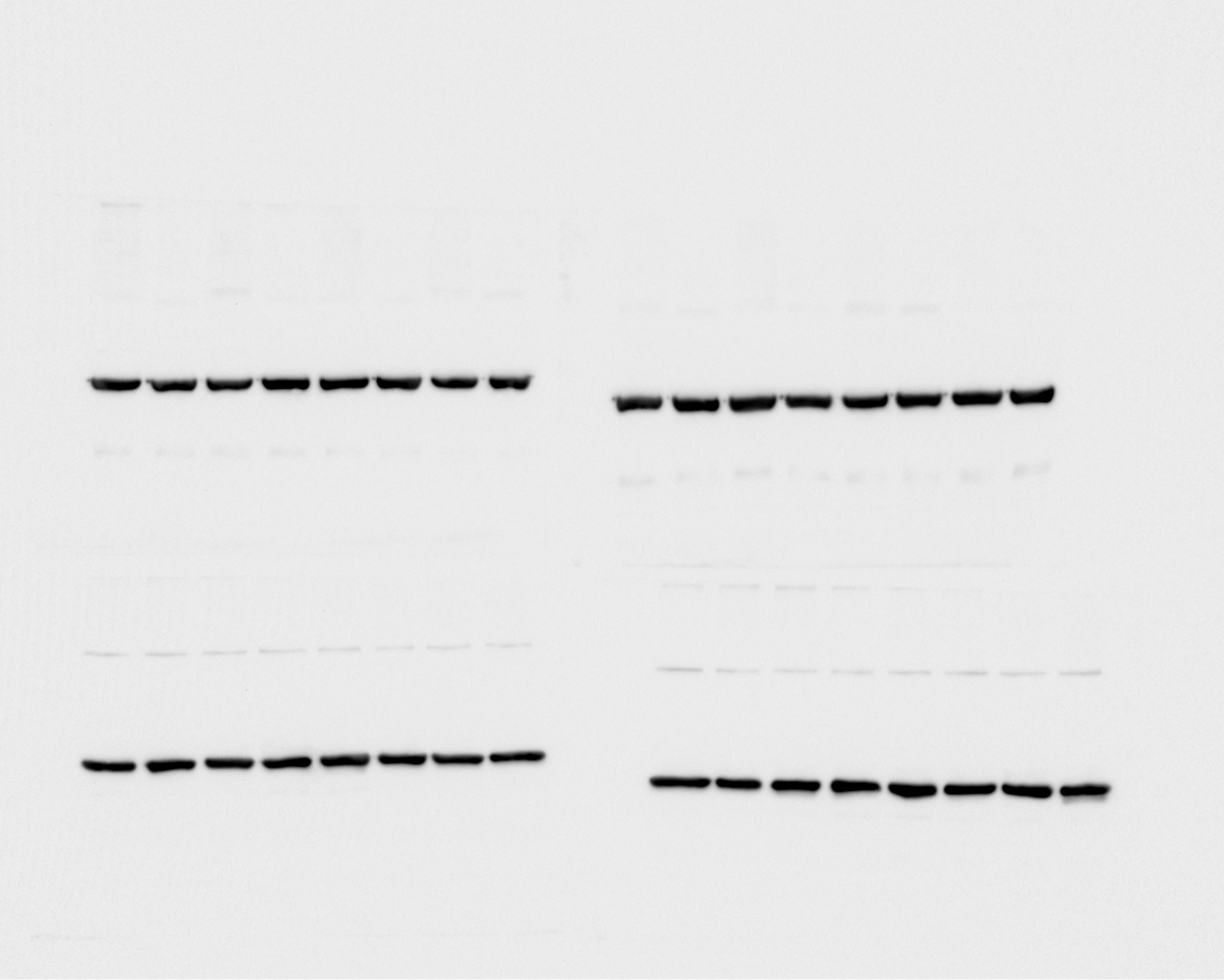

Supplement: Figure 1—source data 4. [file elife-83159-fig1-data4.zip › Figure 1-source data 4/ACTIN Figure 1-source data 4/Adriana 2019-07-03 16h28m24s 7.263s(Chemiluminescence).jpg]

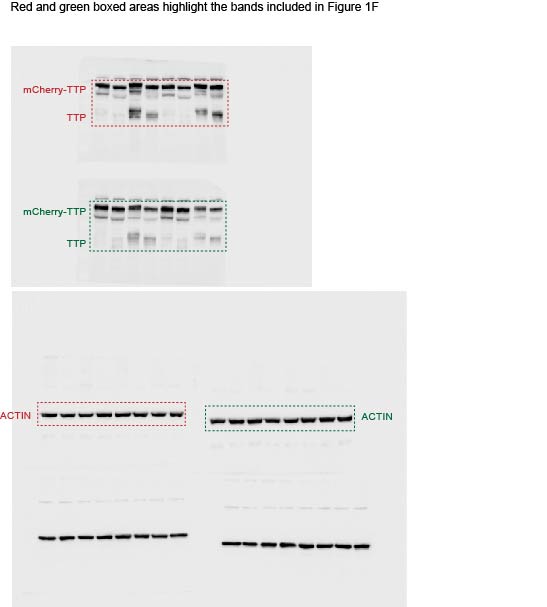

Supplement: Figure 1—source data 4. [file elife-83159-fig1-data4.zip › Figure 1-source data 4/Figure 1-source data 4.jpg]

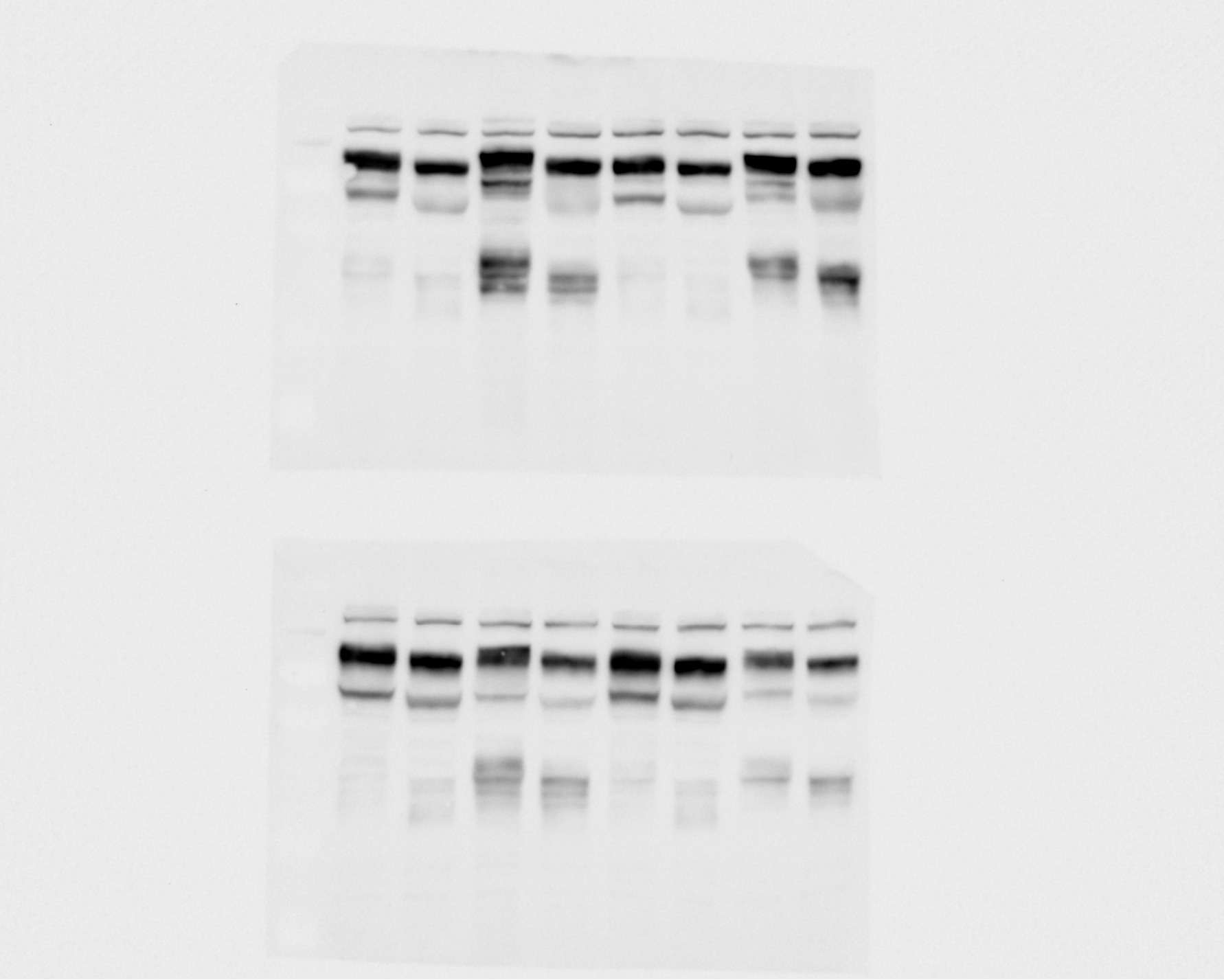

Supplement: Figure 1—source data 4. [file elife-83159-fig1-data4.zip › Figure 1-source data 4/TTP Figure 1-source data 4/Run 2019-07-03 13h04m33s 11.310s(Chemiluminescence).tif]

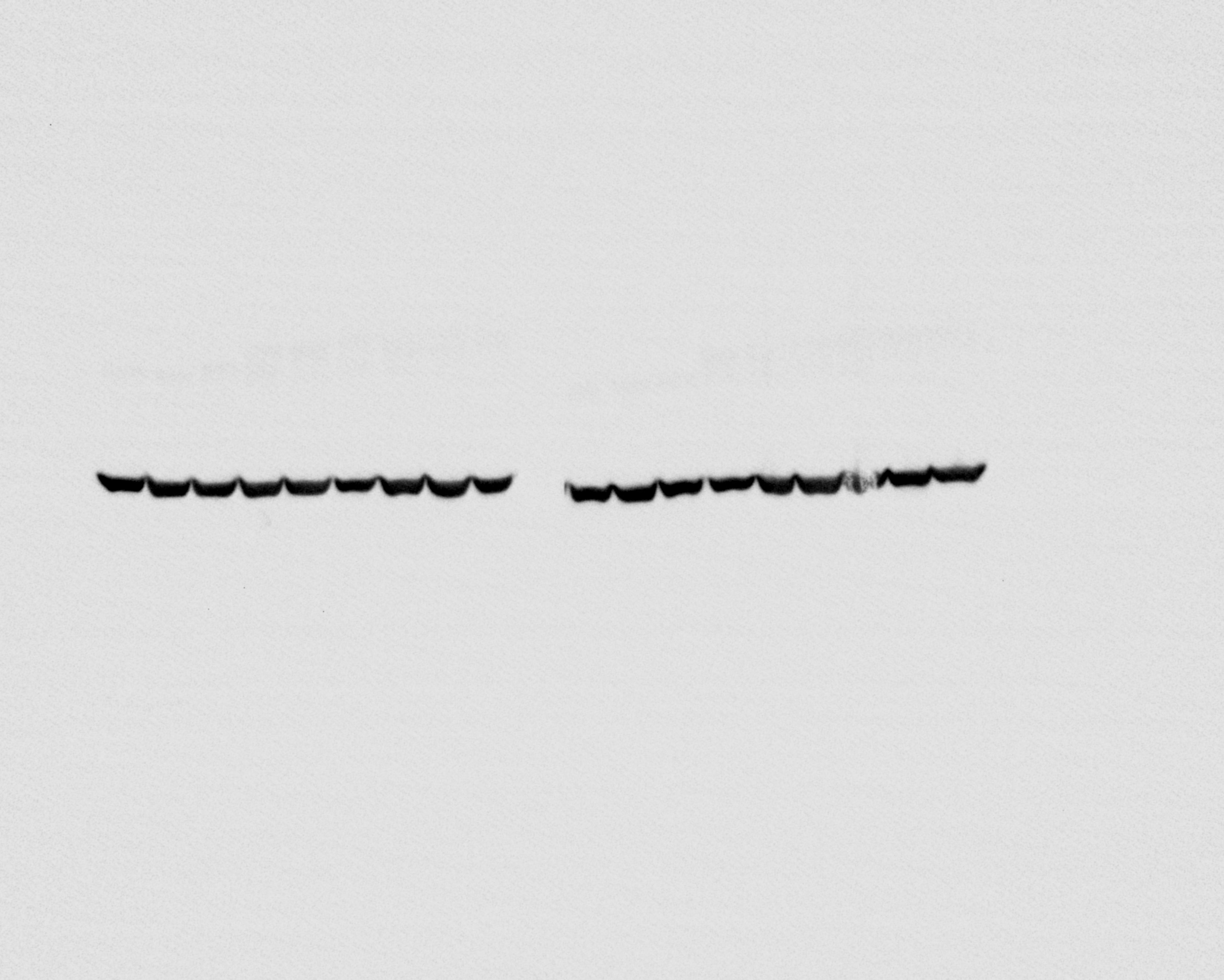

Supplement: Figure 1—source data 5. [file elife-83159-fig1-data5.zip › Figure 1-source data 5/Actin Figure 1-source data 5/Actin Run 2019-06-19 15h16m48s 33.824s.tif]

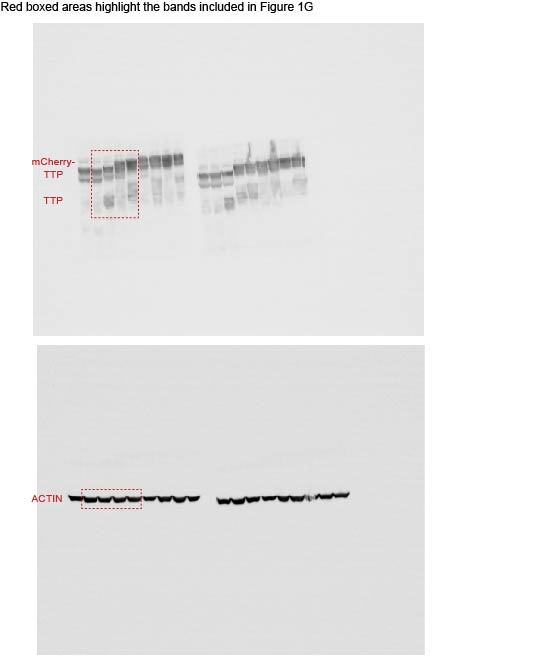

Supplement: Figure 1—source data 5. [file elife-83159-fig1-data5.zip › Figure 1-source data 5/Figure 1-source data 5.jpg]

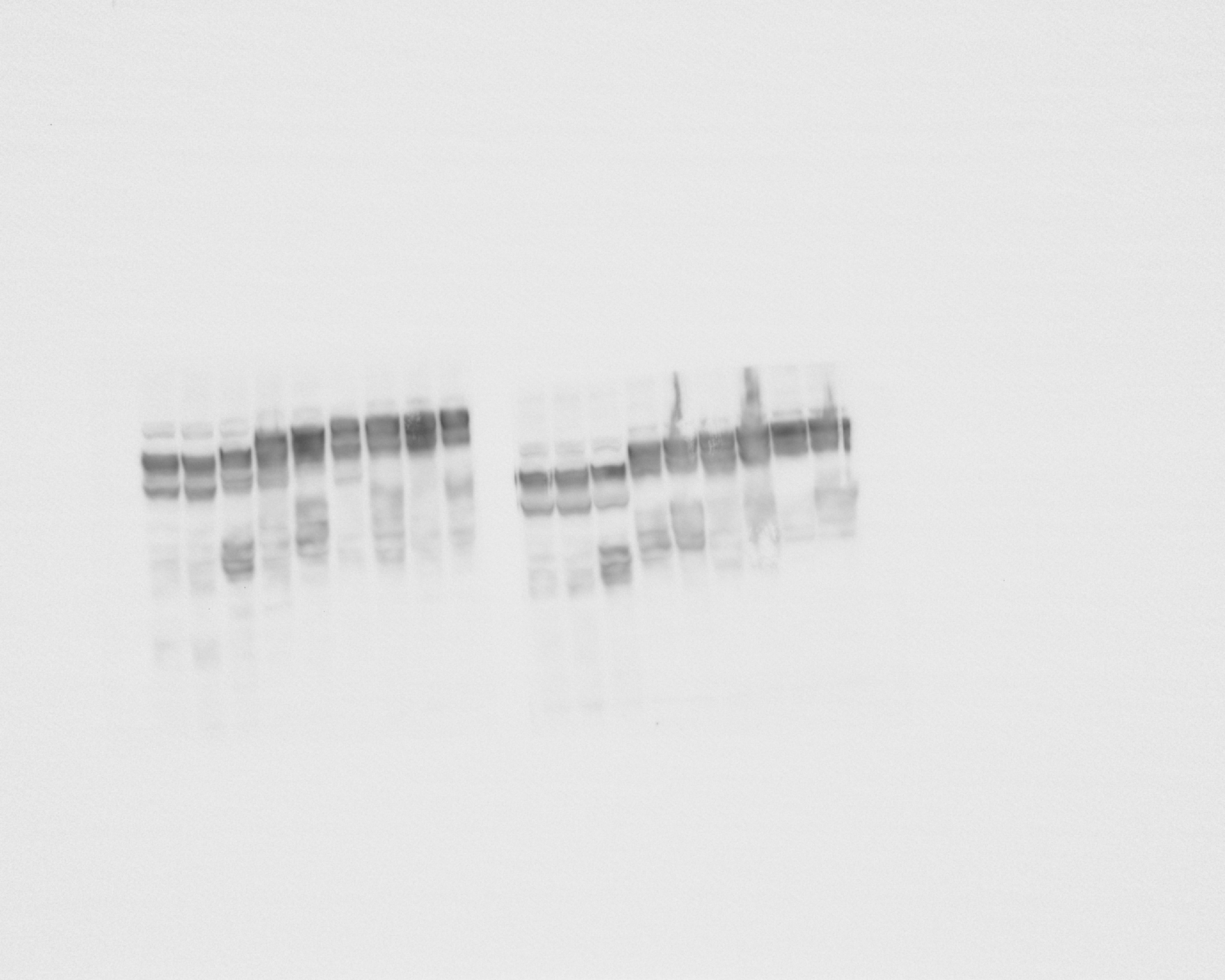

Supplement: Figure 1—source data 5. [file elife-83159-fig1-data5.zip › Figure 1-source data 5/TTP Figure 1-source data 5/Run 2019-06-19 11h12m49s 55.918s(Chemiluminescence).jpg]

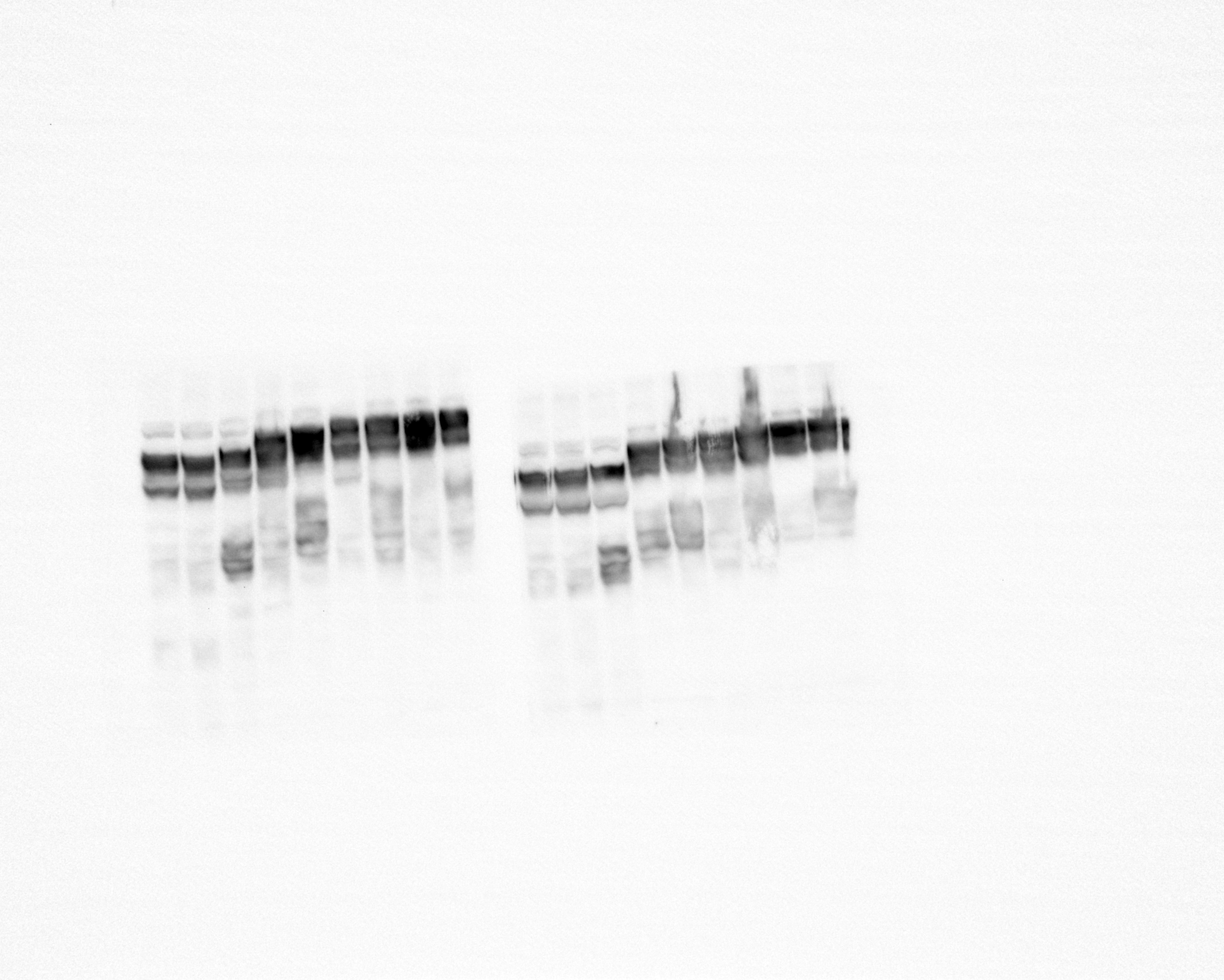

Supplement: Figure 1—source data 5. [file elife-83159-fig1-data5.zip › Figure 1-source data 5/TTP Figure 1-source data 5/Run 2019-06-19 11h12m49s 55.918s(Chemiluminescence).raw16.tif]

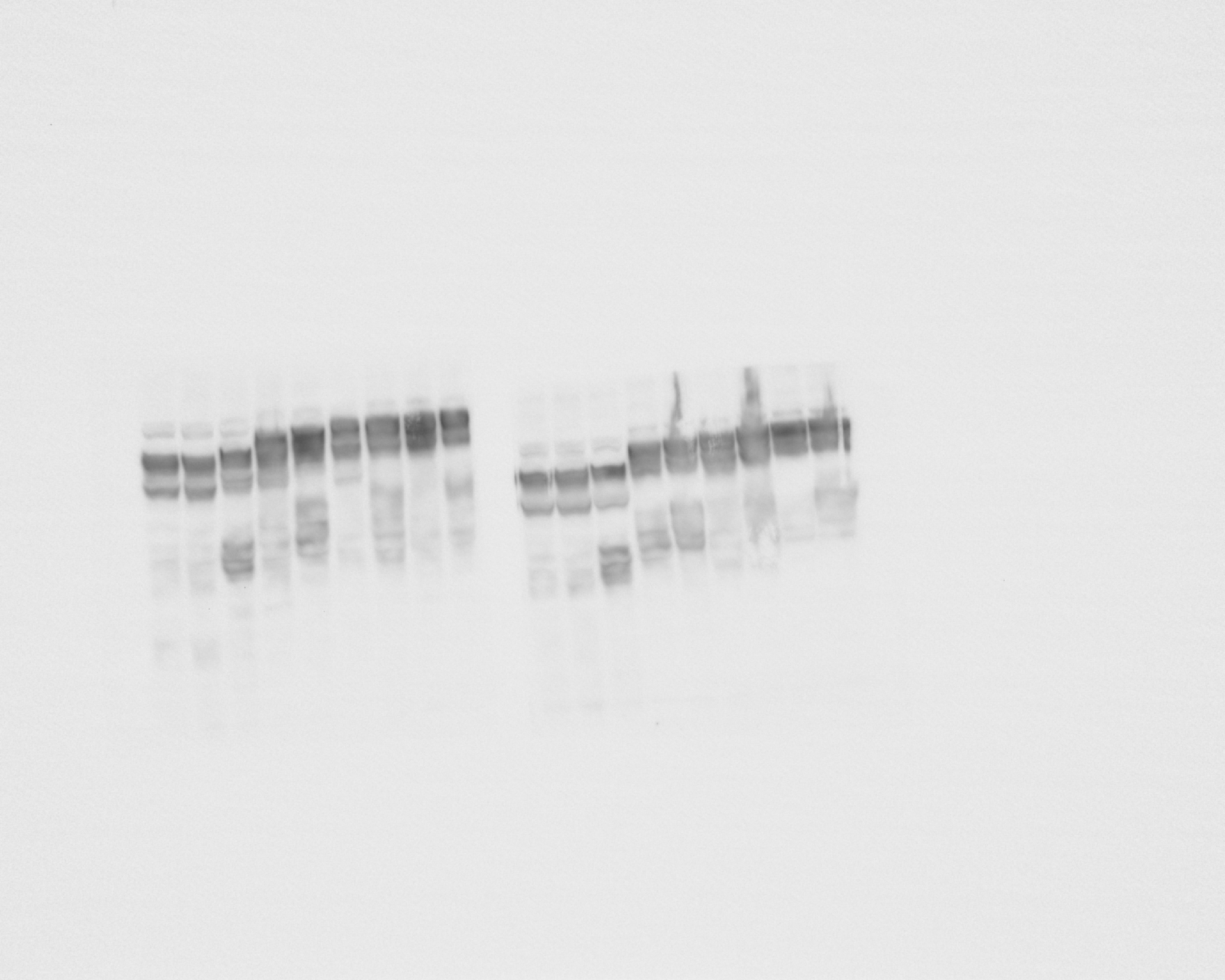

Supplement: Figure 1—source data 5. [file elife-83159-fig1-data5.zip › Figure 1-source data 5/TTP Figure 1-source data 5/Run 2019-06-19 11h12m49s 55.918s(Chemiluminescence).tif]

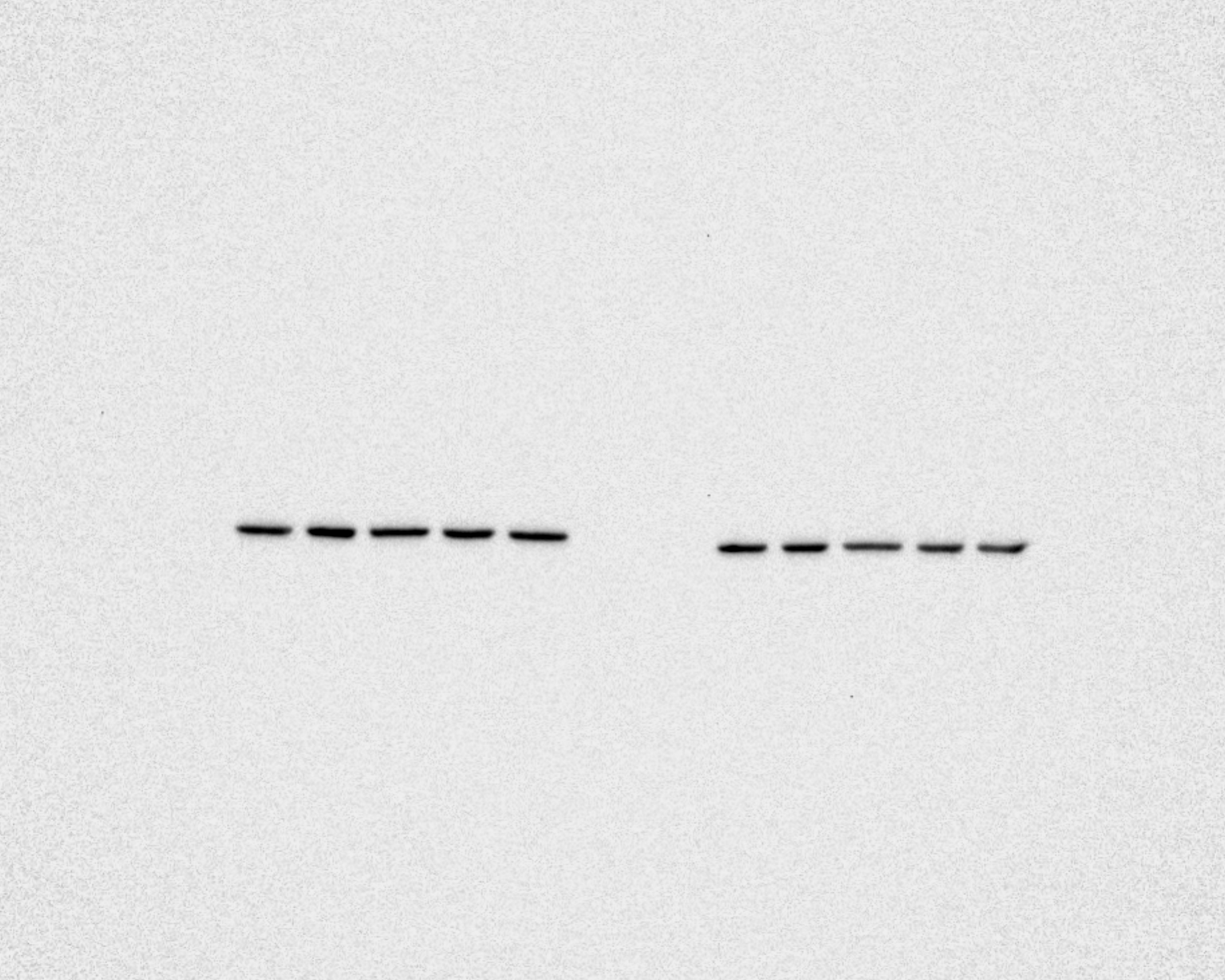

Supplement: Figure 1—figure supplement 1—source data 1. [file elife-83159-fig1-figsupp1-data1.zip › ACTIN Figure 1-figure supplement 1-source data 1/Versteeg 2022-12-02 14h09m41s 119.985s(Chemiluminescence).jpg]

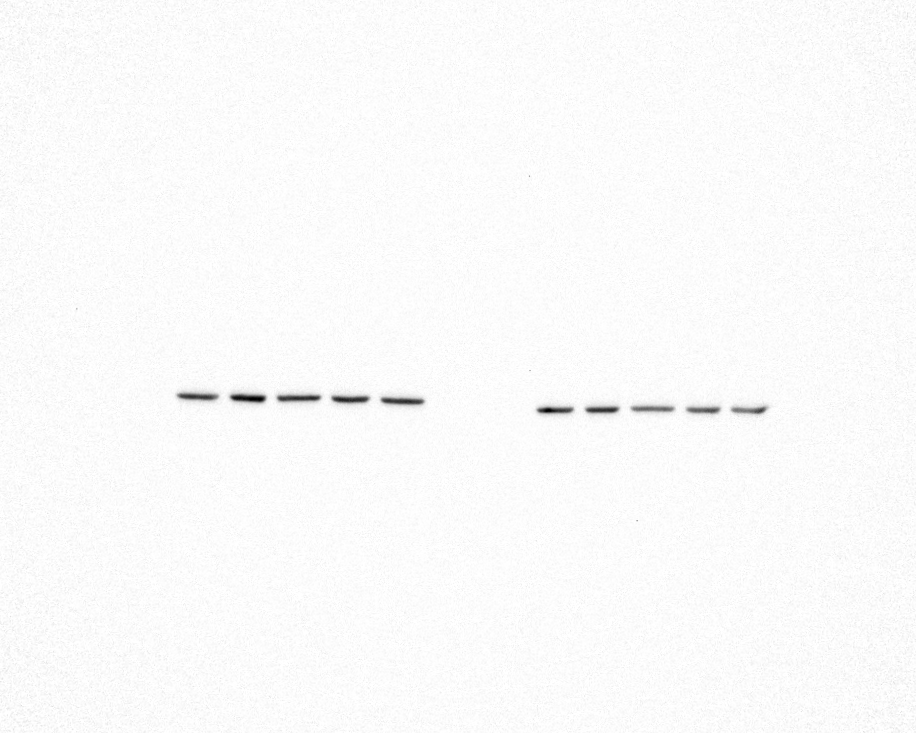

Supplement: Figure 1—figure supplement 1—source data 1. [file elife-83159-fig1-figsupp1-data1.zip › ACTIN Figure 1-figure supplement 1-source data 1/Versteeg 2022-12-02 14h09m41s 119.985s(Chemiluminescence).raw16.tif]

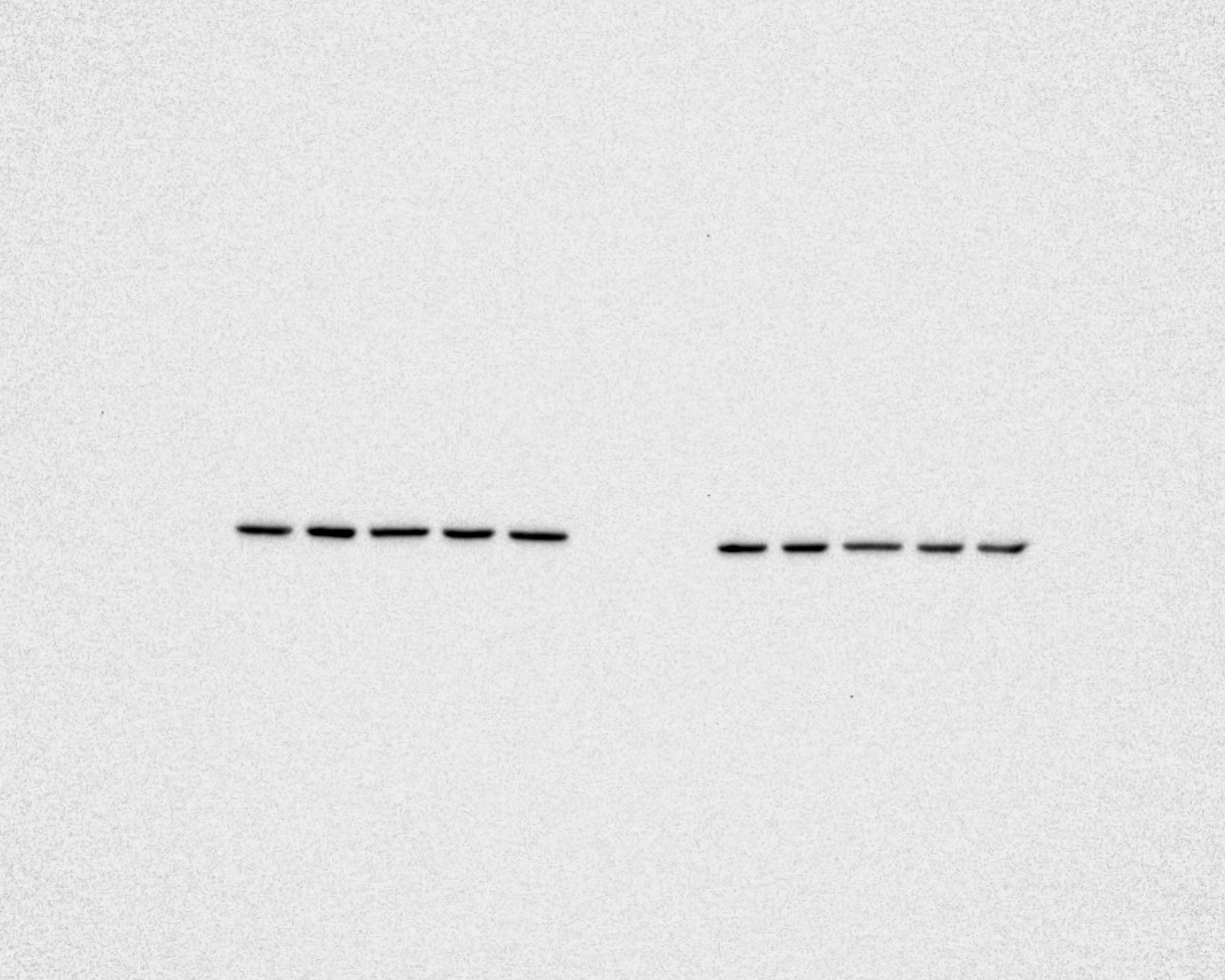

Supplement: Figure 1—figure supplement 1—source data 1. [file elife-83159-fig1-figsupp1-data1.zip › ACTIN Figure 1-figure supplement 1-source data 1/Versteeg 2022-12-02 14h09m41s 119.985s(Chemiluminescence).tif]

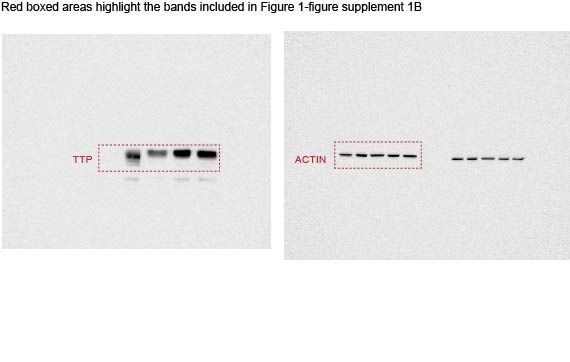

Supplement: Figure 1—figure supplement 1—source data 1. [file elife-83159-fig1-figsupp1-data1.zip › Figure 1-figure supplement 1-source data 1.jpg]

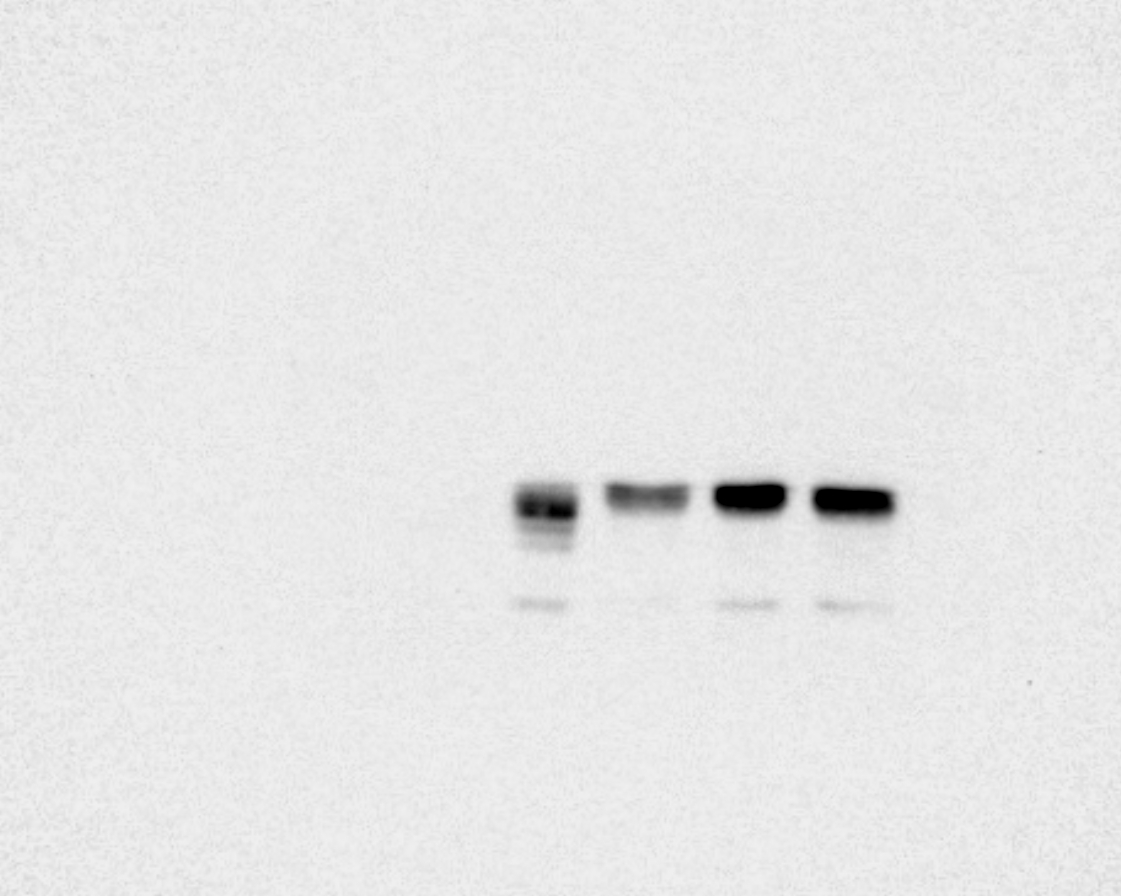

Supplement: Figure 1—figure supplement 1—source data 1. [file elife-83159-fig1-figsupp1-data1.zip › TTP Figure 1-figure supplement 1-source data 1/Versteeg 2022-12-01 13h08m02s 124.720s(Chemiluminescence).jpg]

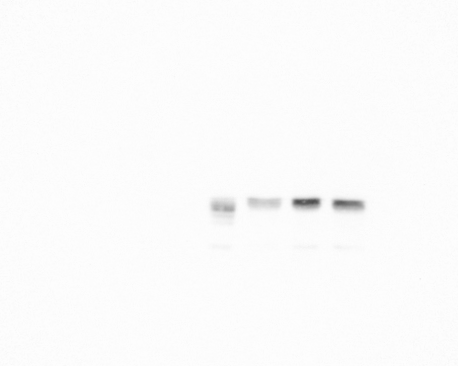

Supplement: Figure 1—figure supplement 1—source data 1. [file elife-83159-fig1-figsupp1-data1.zip › TTP Figure 1-figure supplement 1-source data 1/Versteeg 2022-12-01 13h08m02s 124.720s(Chemiluminescence).raw16.tif]

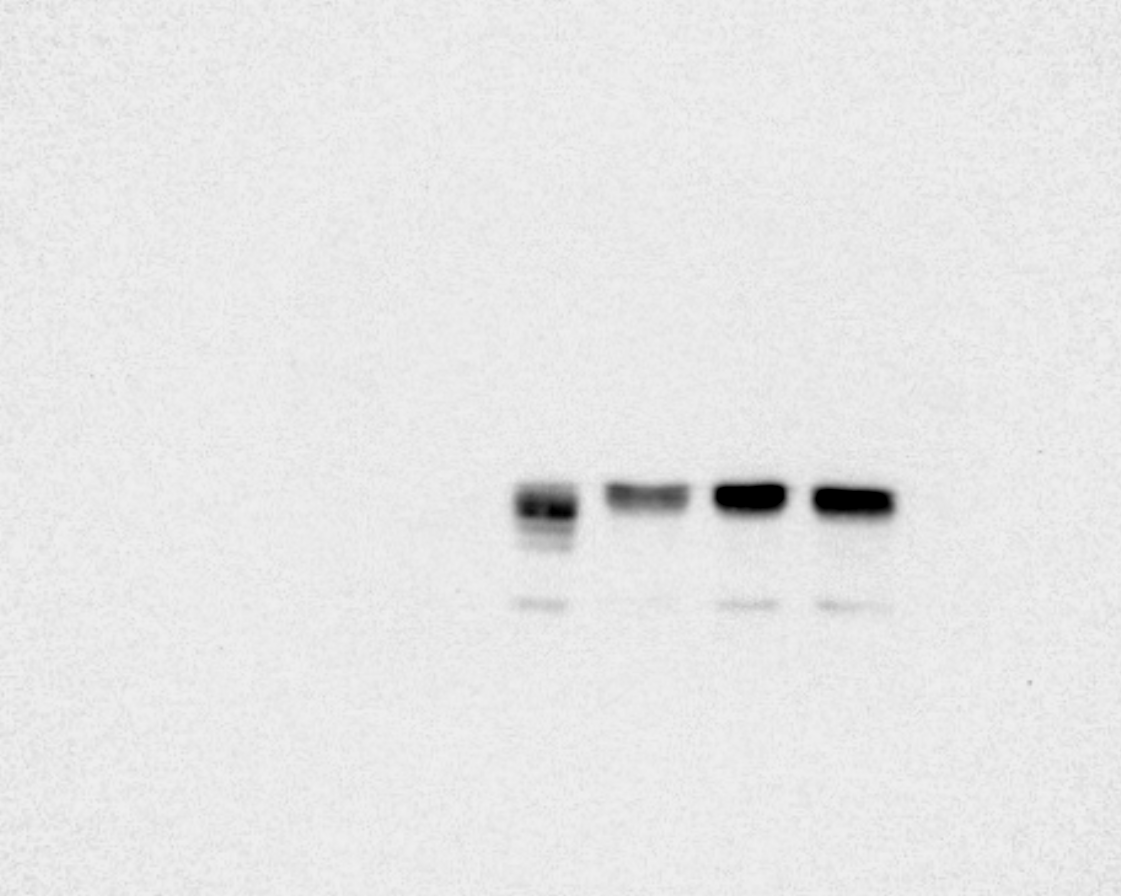

Supplement: Figure 1—figure supplement 1—source data 1. [file elife-83159-fig1-figsupp1-data1.zip › TTP Figure 1-figure supplement 1-source data 1/Versteeg 2022-12-01 13h08m02s 124.720s(Chemiluminescence).tif]

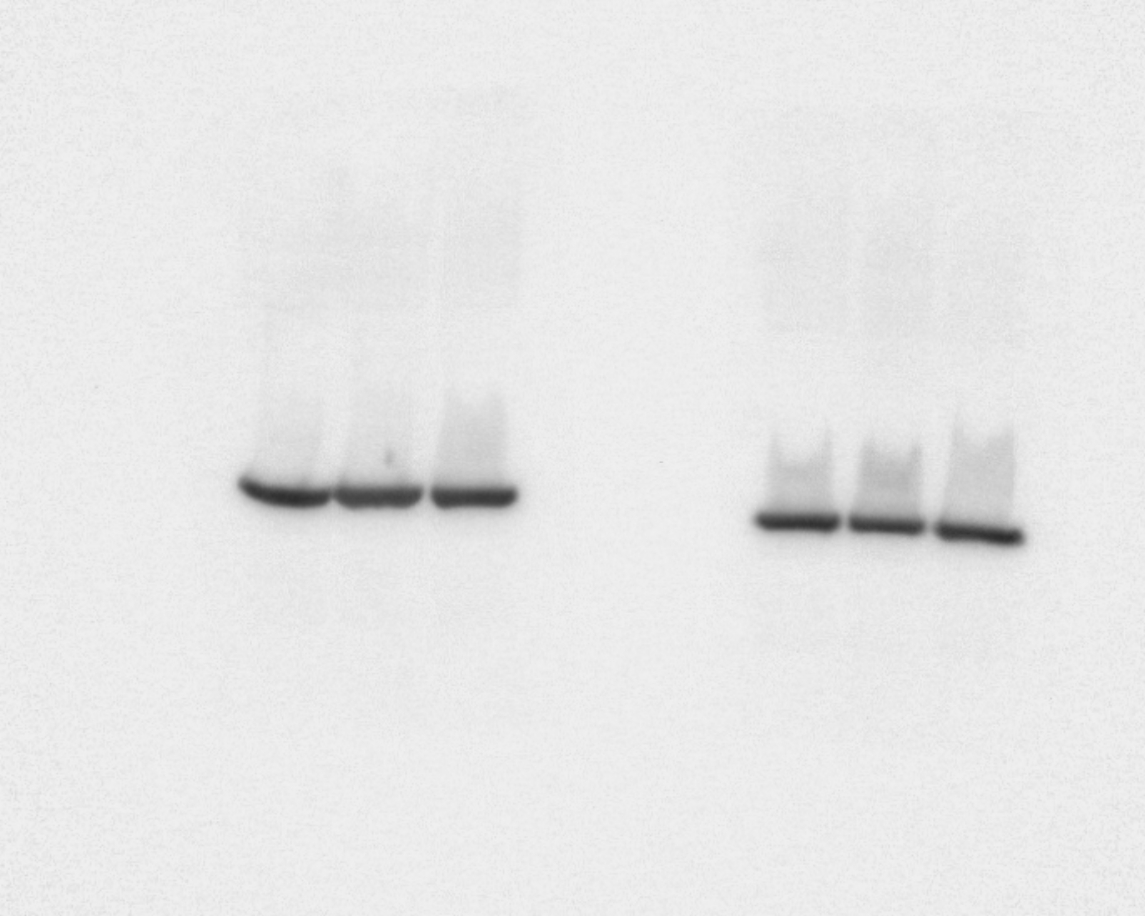

Supplement: Figure 1—figure supplement 1—source data 2. [file elife-83159-fig1-figsupp1-data2.zip › ACTIN WCE Figure 1-figure supplement 1-source data 2/Versteeg 2022-07-05 16h19m39s 19.306s(Chemiluminescence).jpg]

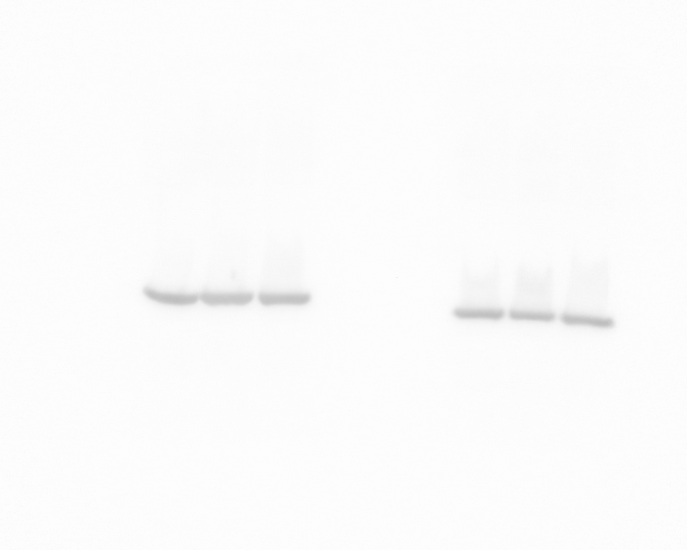

Supplement: Figure 1—figure supplement 1—source data 2. [file elife-83159-fig1-figsupp1-data2.zip › ACTIN WCE Figure 1-figure supplement 1-source data 2/Versteeg 2022-07-05 16h19m39s 19.306s(Chemiluminescence).raw16.tif]

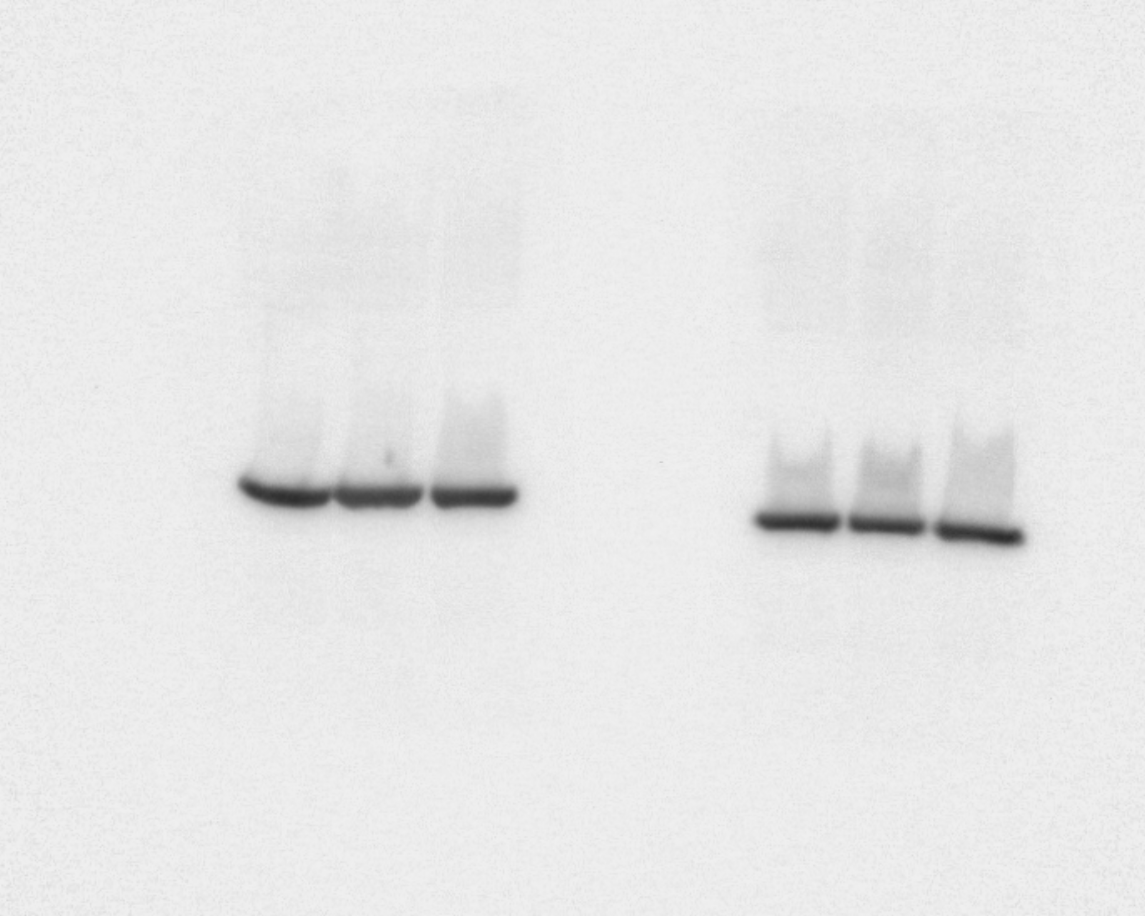

Supplement: Figure 1—figure supplement 1—source data 2. [file elife-83159-fig1-figsupp1-data2.zip › ACTIN WCE Figure 1-figure supplement 1-source data 2/Versteeg 2022-07-05 16h19m39s 19.306s(Chemiluminescence).tif]

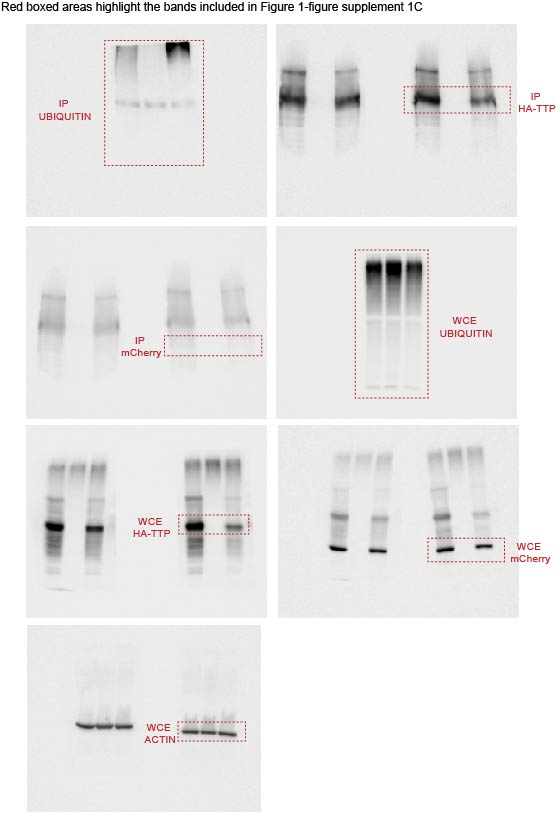

Supplement: Figure 1—figure supplement 1—source data 2. [file elife-83159-fig1-figsupp1-data2.zip › Figure 1-figure supplement 1-source data 2.jpg]

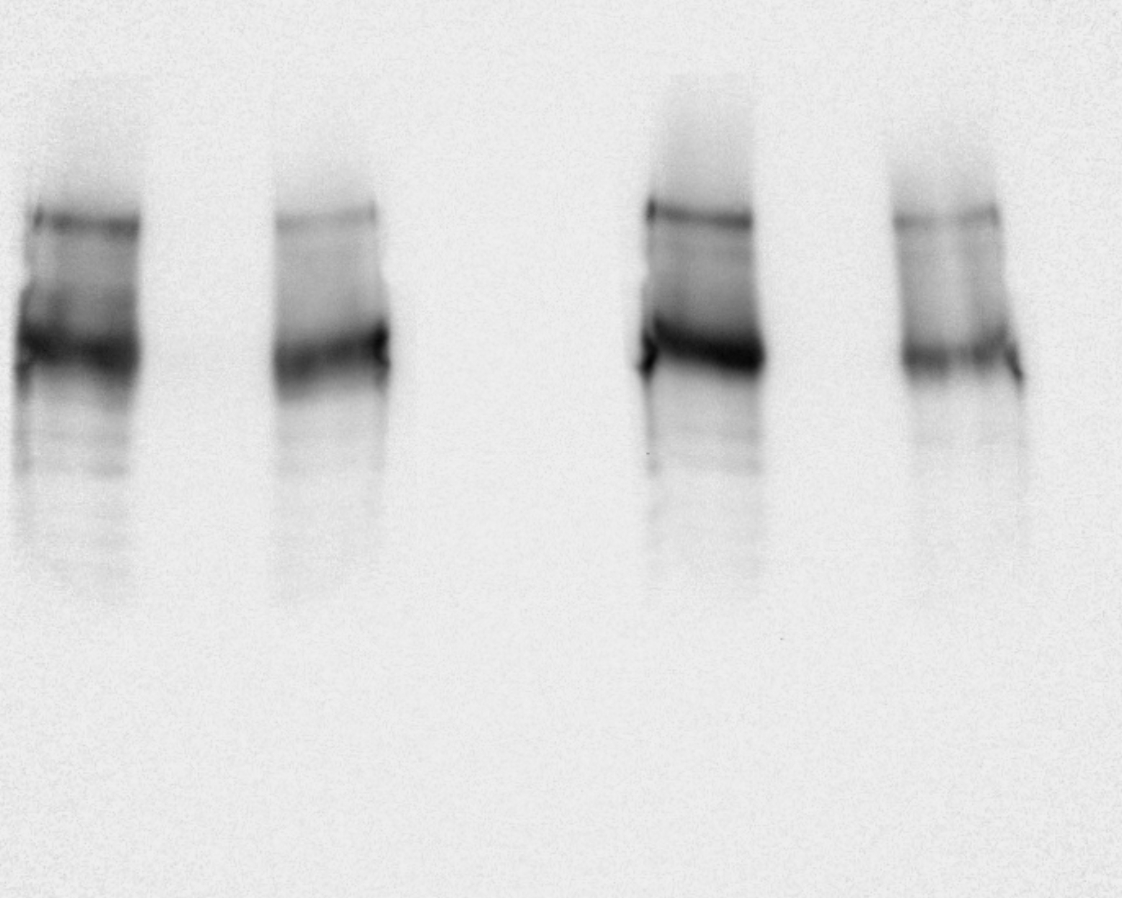

Supplement: Figure 1—figure supplement 1—source data 2. [file elife-83159-fig1-figsupp1-data2.zip › HA-TTP IP Figure 1-figure supplement 1-source data 2/Versteeg 2022-07-04 14h27m16s 23.374s(Chemiluminescence).jpg]

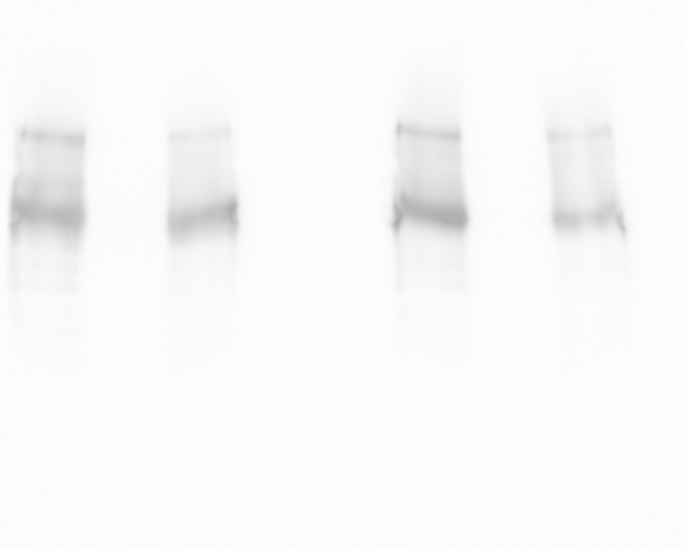

Supplement: Figure 1—figure supplement 1—source data 2. [file elife-83159-fig1-figsupp1-data2.zip › HA-TTP IP Figure 1-figure supplement 1-source data 2/Versteeg 2022-07-04 14h27m16s 23.374s(Chemiluminescence).raw16.tif]

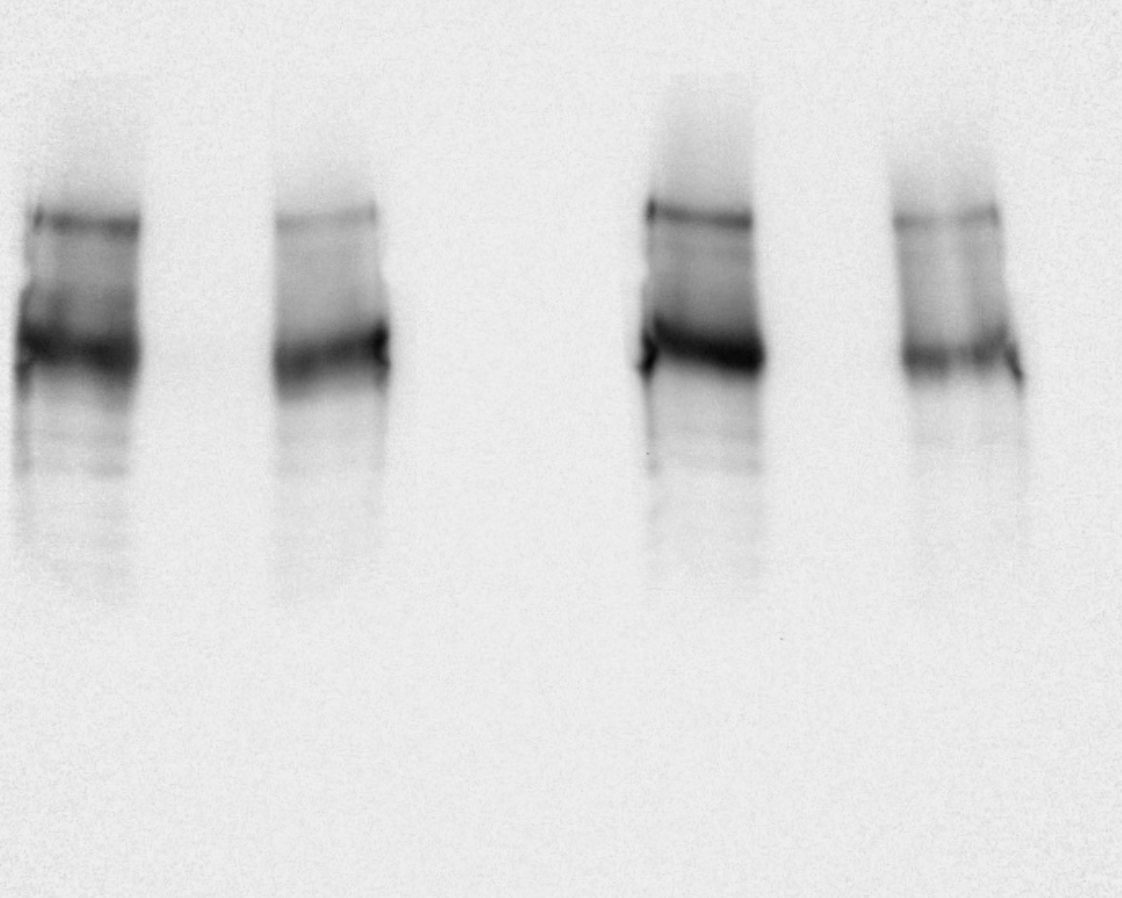

Supplement: Figure 1—figure supplement 1—source data 2. [file elife-83159-fig1-figsupp1-data2.zip › HA-TTP IP Figure 1-figure supplement 1-source data 2/Versteeg 2022-07-04 14h27m16s 23.374s(Chemiluminescence).tif]

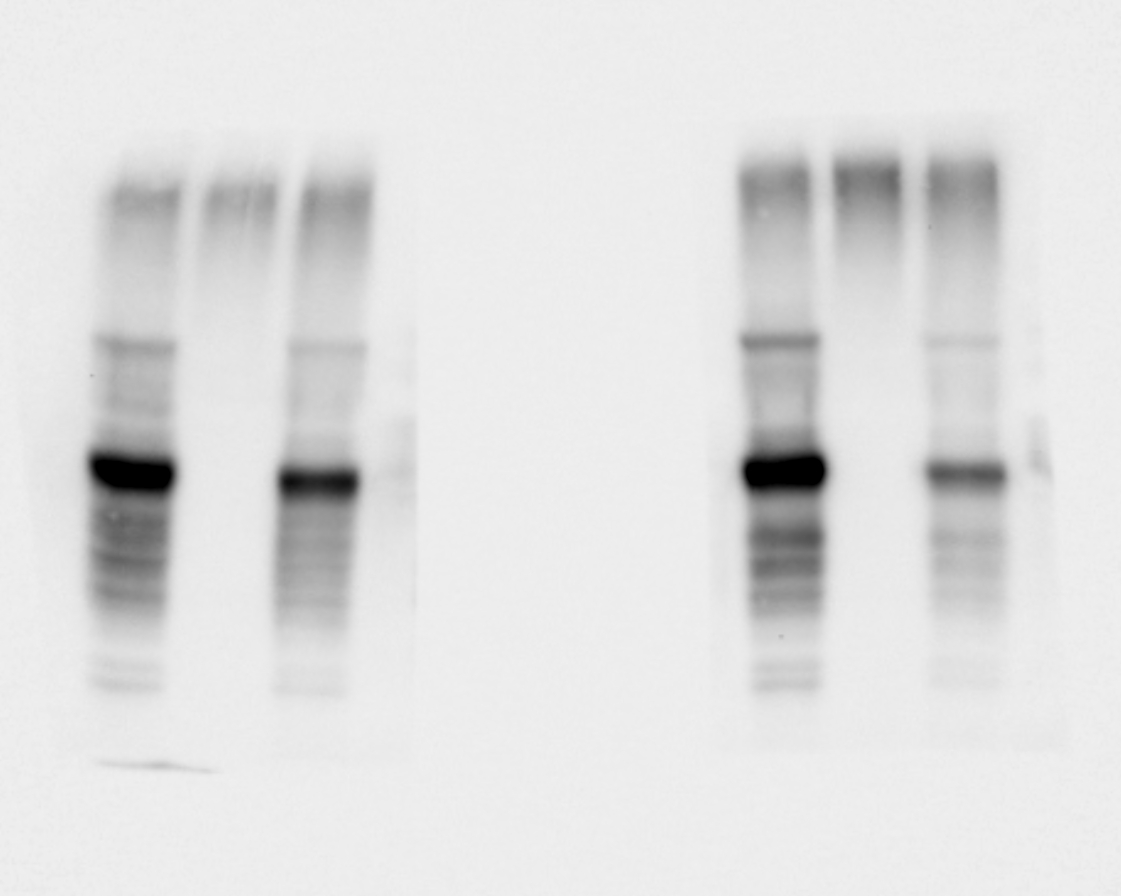

Supplement: Figure 1—figure supplement 1—source data 2. [file elife-83159-fig1-figsupp1-data2.zip › HA-TTP WCE Figure 1-figure supplement 1-source data 2/Versteeg 2022-07-04 14h11m04s 21.379s(Chemiluminescence).jpg]

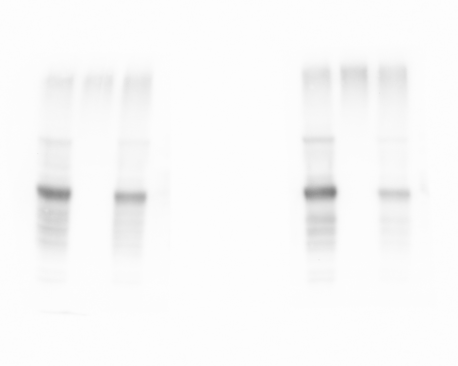

Supplement: Figure 1—figure supplement 1—source data 2. [file elife-83159-fig1-figsupp1-data2.zip › HA-TTP WCE Figure 1-figure supplement 1-source data 2/Versteeg 2022-07-04 14h11m04s 21.379s(Chemiluminescence).raw16.tif]

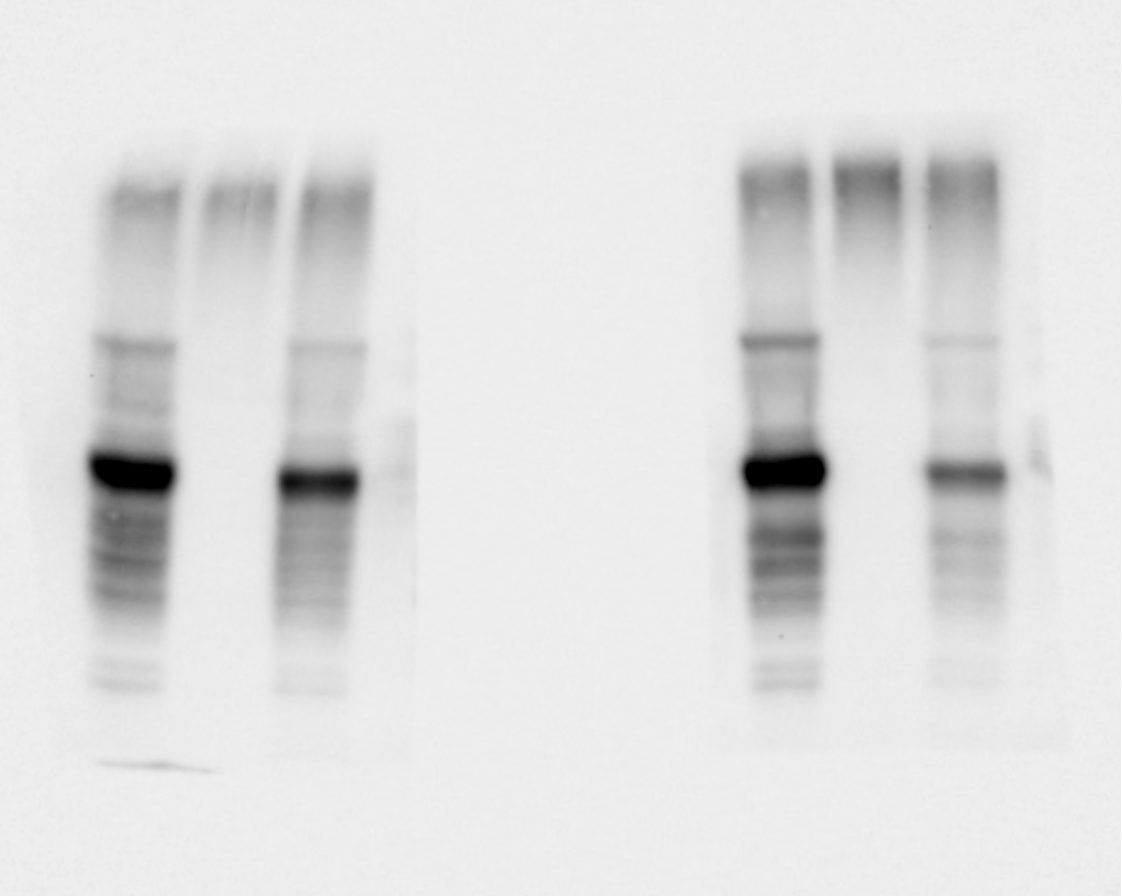

Supplement: Figure 1—figure supplement 1—source data 2. [file elife-83159-fig1-figsupp1-data2.zip › HA-TTP WCE Figure 1-figure supplement 1-source data 2/Versteeg 2022-07-04 14h11m04s 21.379s(Chemiluminescence).tif]

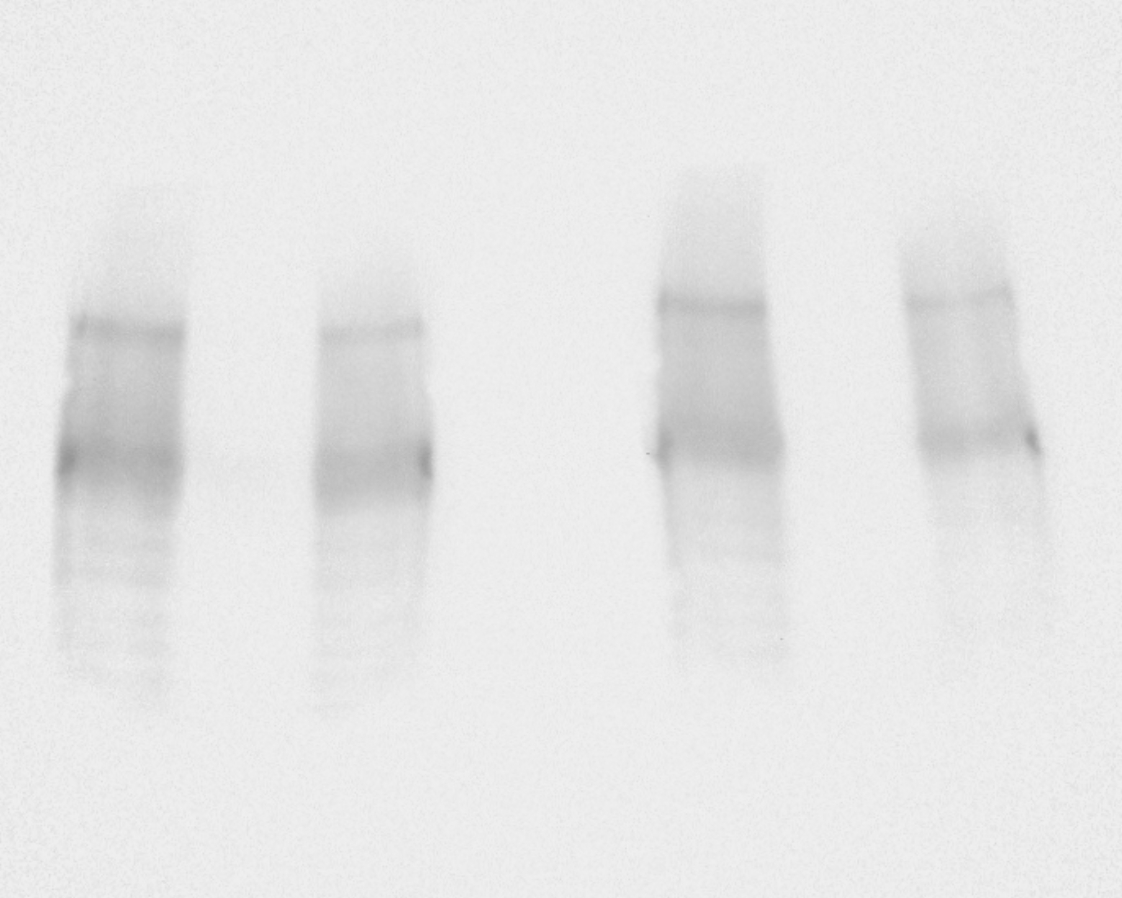

Supplement: Figure 1—figure supplement 1—source data 2. [file elife-83159-fig1-figsupp1-data2.zip › mCherry IP Figure 1-figure supplement 1-source data 2/Versteeg 2022-07-05 13h09m41s 17.272s(Chemiluminescence).jpg]

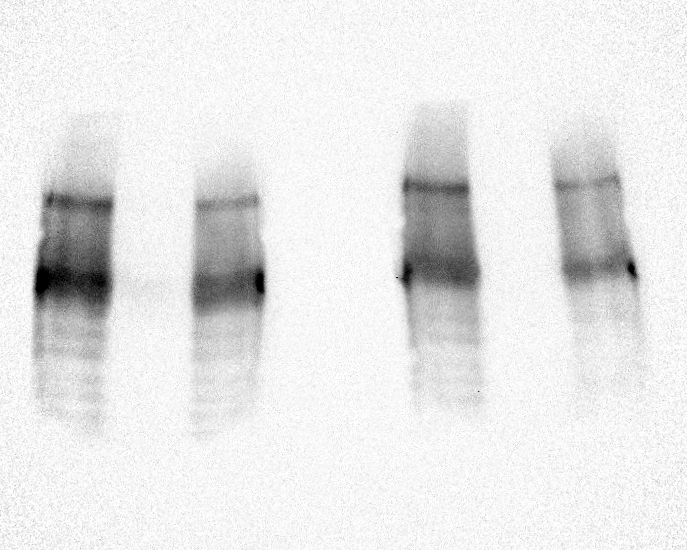

Supplement: Figure 1—figure supplement 1—source data 2. [file elife-83159-fig1-figsupp1-data2.zip › mCherry IP Figure 1-figure supplement 1-source data 2/Versteeg 2022-07-05 13h09m41s 17.272s(Chemiluminescence).raw16.tif]

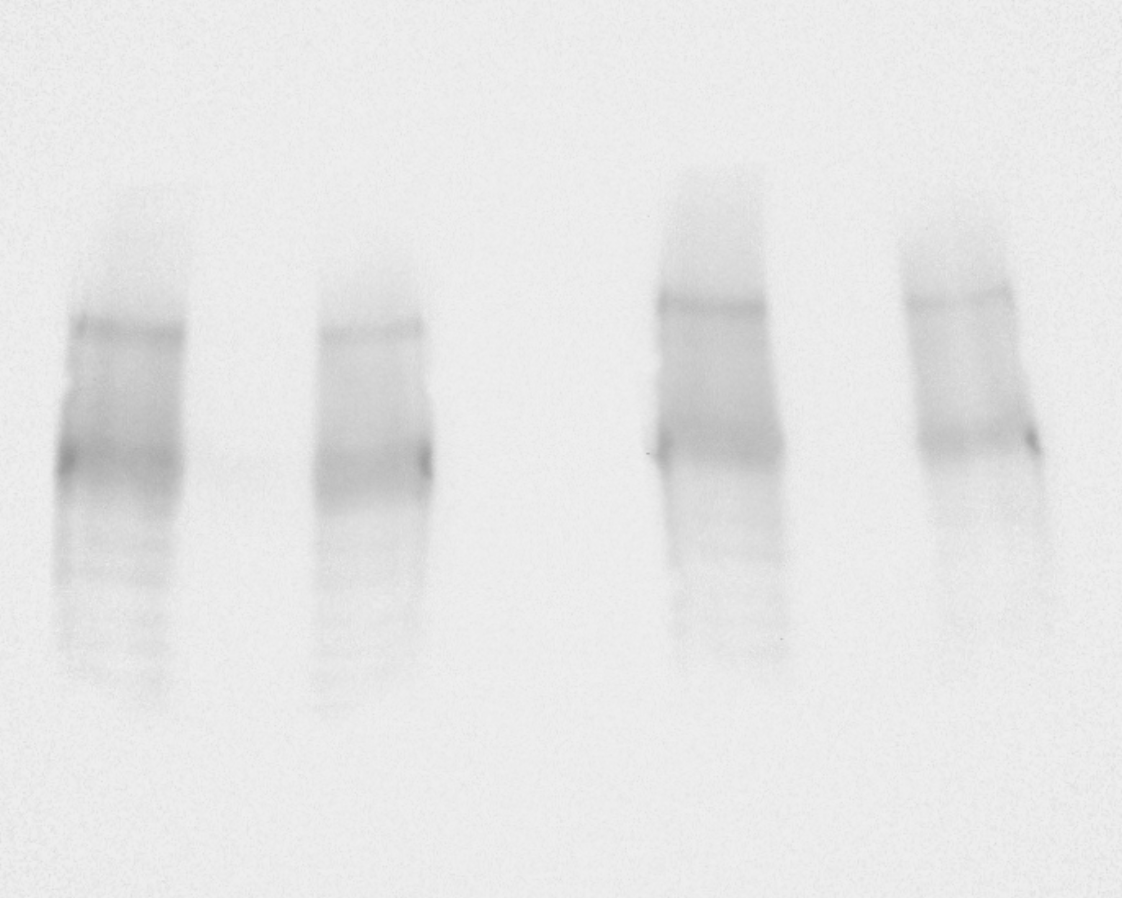

Supplement: Figure 1—figure supplement 1—source data 2. [file elife-83159-fig1-figsupp1-data2.zip › mCherry IP Figure 1-figure supplement 1-source data 2/Versteeg 2022-07-05 13h09m41s 17.272s(Chemiluminescence).tif]

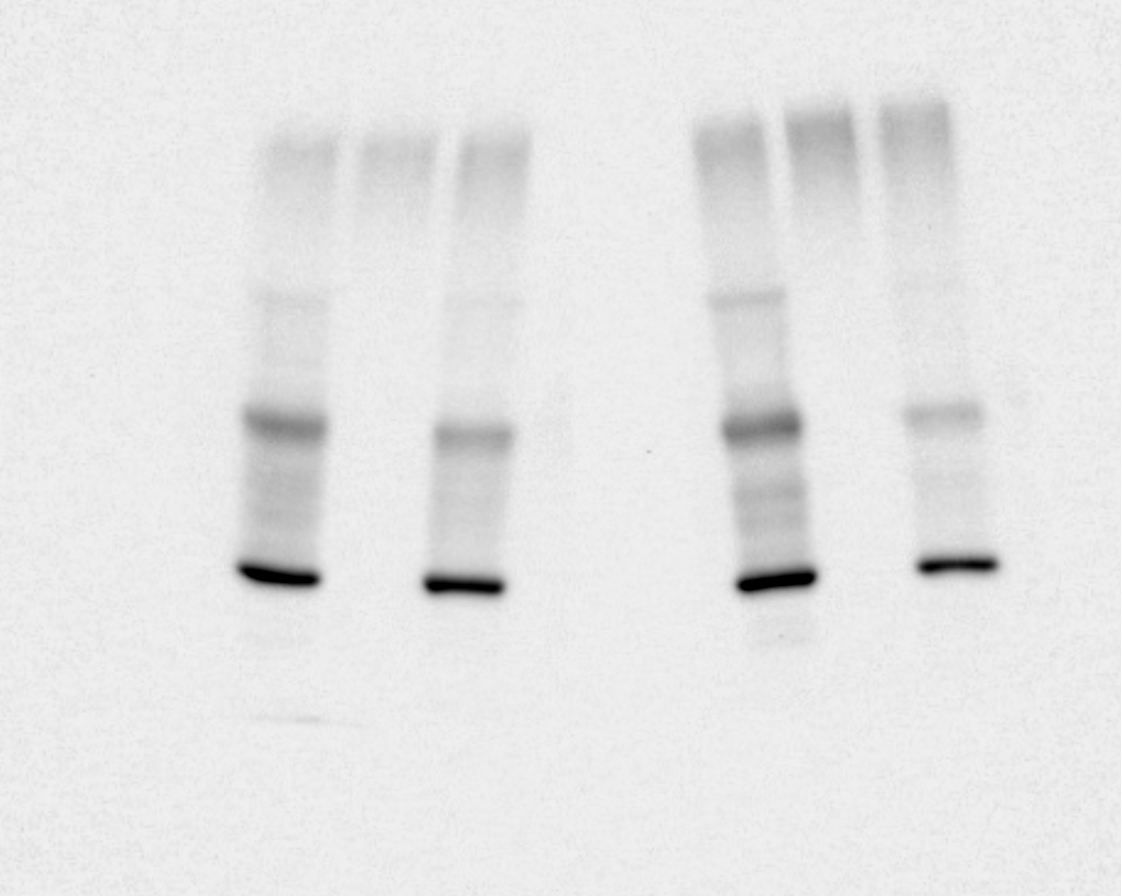

Supplement: Figure 1—figure supplement 1—source data 2. [file elife-83159-fig1-figsupp1-data2.zip › mCherry WCE Figure 1-figure supplement 1-source data 2/Versteeg 2022-07-05 13h15m14s 17.272s(Chemiluminescence).jpg]

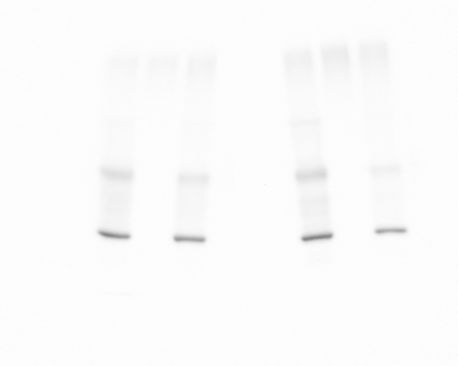

Supplement: Figure 1—figure supplement 1—source data 2. [file elife-83159-fig1-figsupp1-data2.zip › mCherry WCE Figure 1-figure supplement 1-source data 2/Versteeg 2022-07-05 13h15m14s 17.272s(Chemiluminescence).raw16.tif]

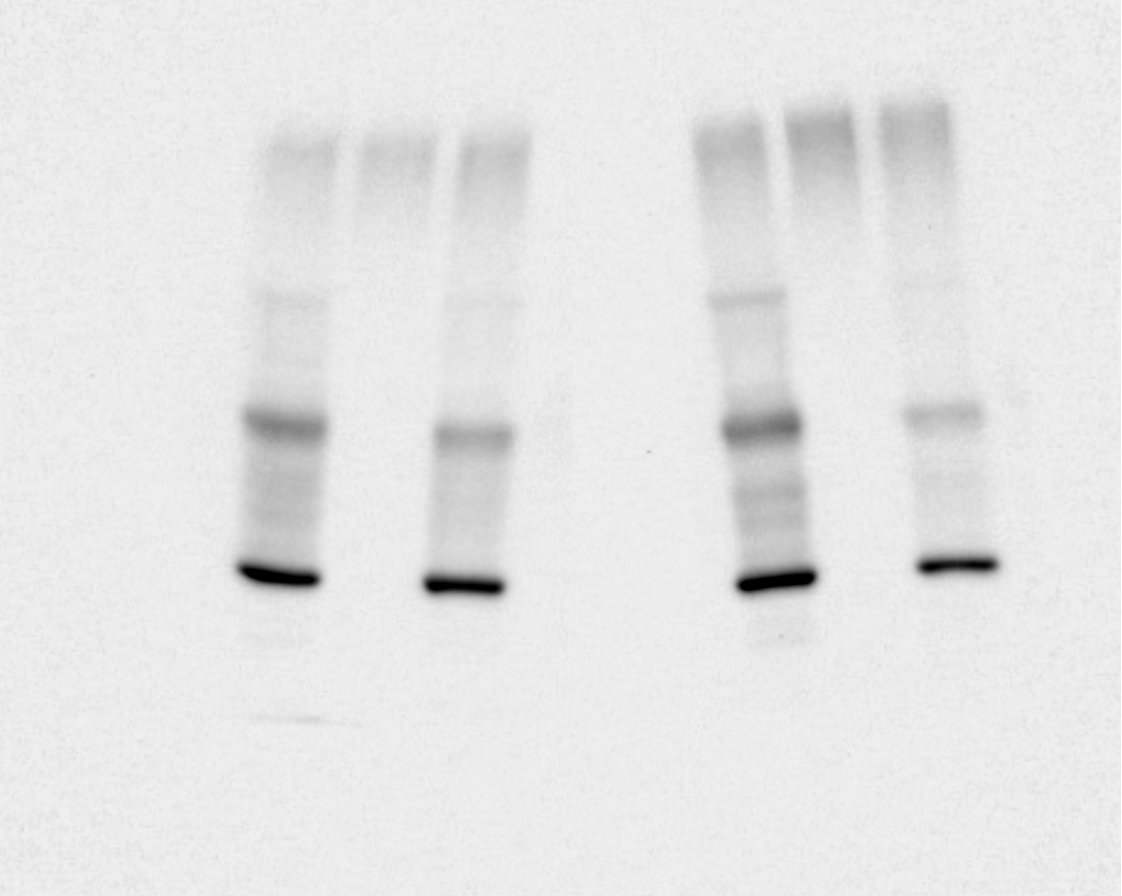

Supplement: Figure 1—figure supplement 1—source data 2. [file elife-83159-fig1-figsupp1-data2.zip › mCherry WCE Figure 1-figure supplement 1-source data 2/Versteeg 2022-07-05 13h15m14s 17.272s(Chemiluminescence).tif]

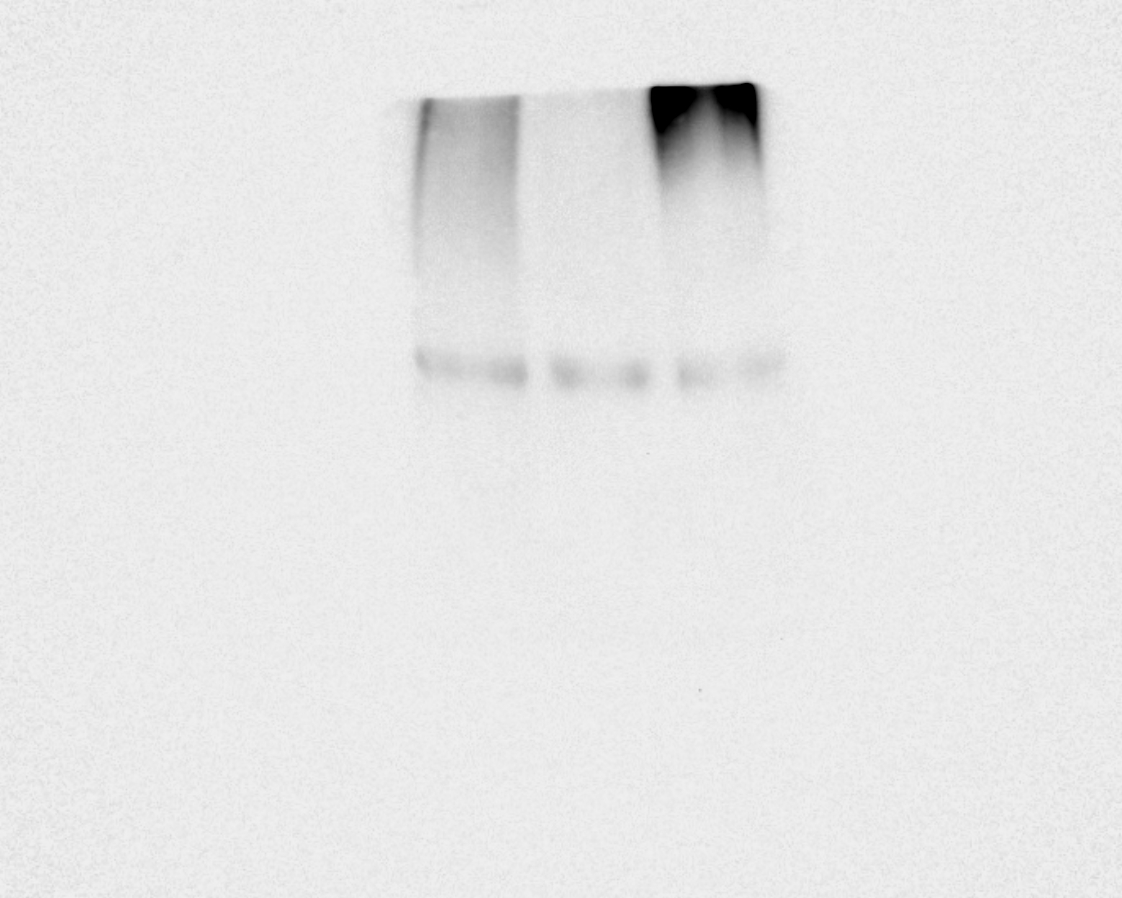

Supplement: Figure 1—figure supplement 1—source data 2. [file elife-83159-fig1-figsupp1-data2.zip › UB IP Figure 1-figure supplement 1-source data 2/Versteeg 2022-07-01 13h18m05s 12.000s(Chemiluminescence).jpg]

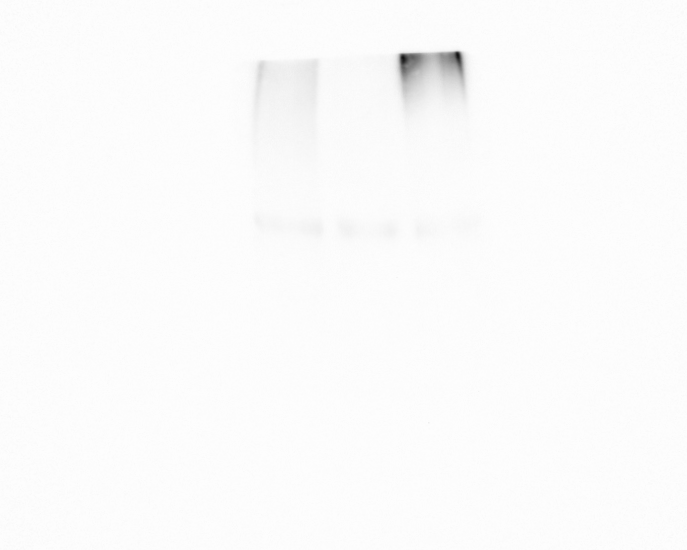

Supplement: Figure 1—figure supplement 1—source data 2. [file elife-83159-fig1-figsupp1-data2.zip › UB IP Figure 1-figure supplement 1-source data 2/Versteeg 2022-07-01 13h18m05s 12.000s(Chemiluminescence).raw16.tif]

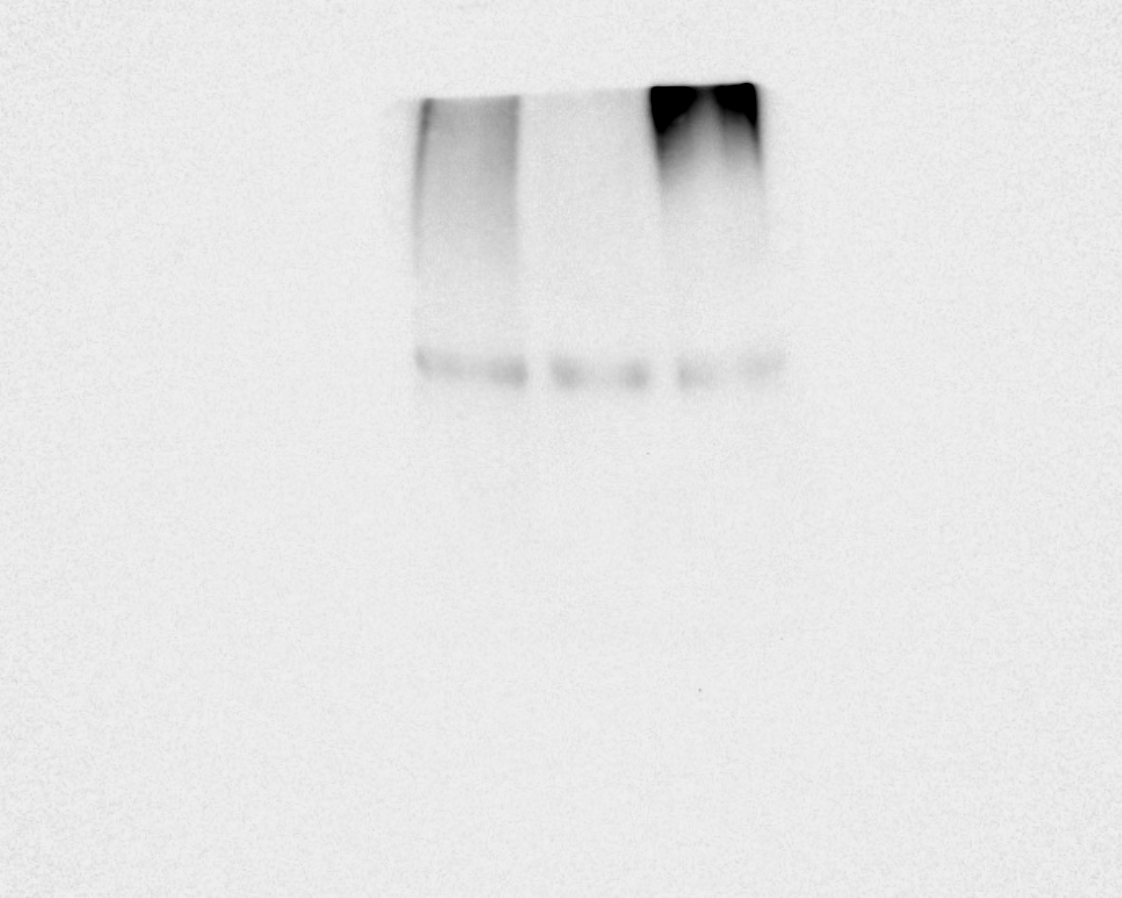

Supplement: Figure 1—figure supplement 1—source data 2. [file elife-83159-fig1-figsupp1-data2.zip › UB IP Figure 1-figure supplement 1-source data 2/Versteeg 2022-07-01 13h18m05s 12.000s(Chemiluminescence).tif]

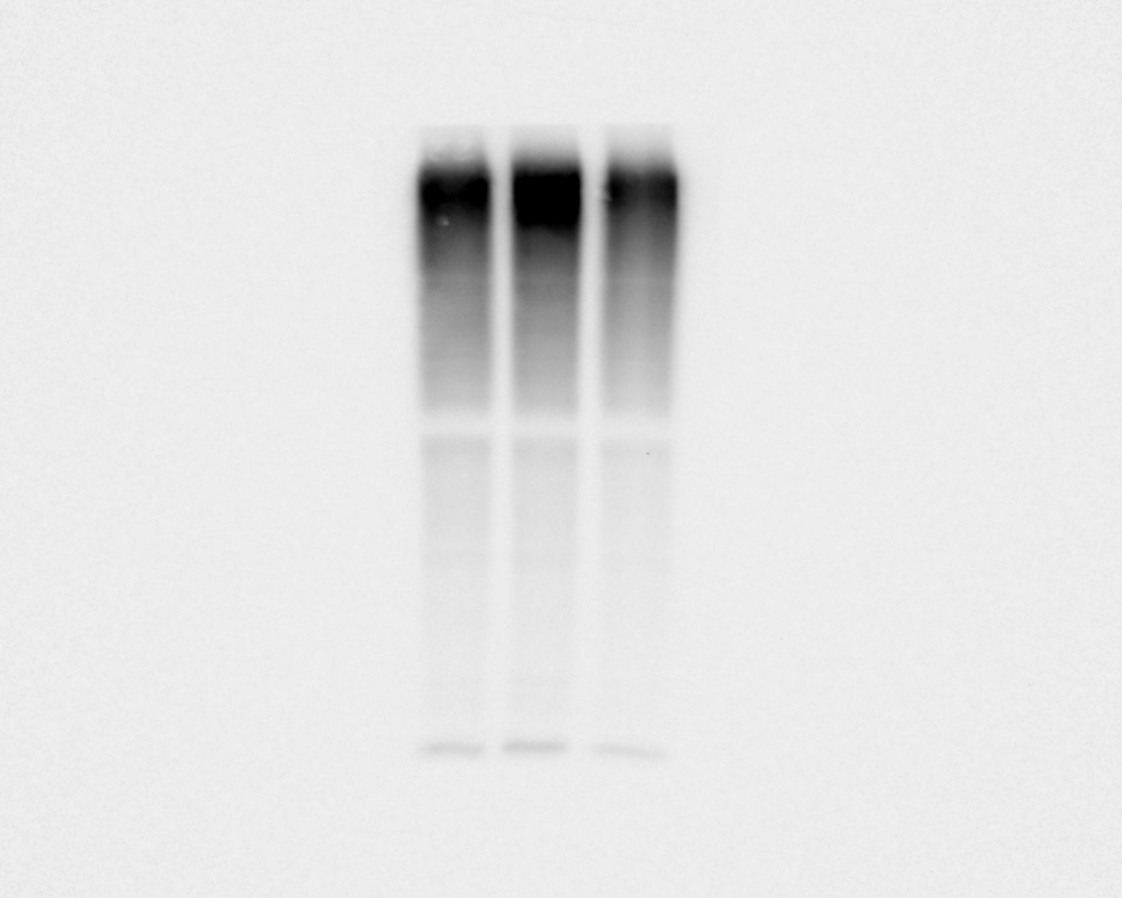

Supplement: Figure 1—figure supplement 1—source data 2. [file elife-83159-fig1-figsupp1-data2.zip › UB WCE Figure 1-figure supplement 1-source data 2/Versteeg 2022-07-01 16h05m59s 17.893s(Chemiluminescence).jpg]

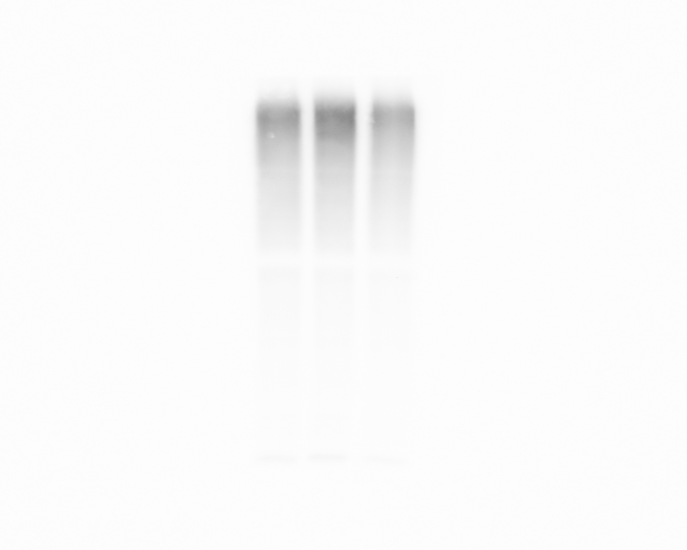

Supplement: Figure 1—figure supplement 1—source data 2. [file elife-83159-fig1-figsupp1-data2.zip › UB WCE Figure 1-figure supplement 1-source data 2/Versteeg 2022-07-01 16h05m59s 17.893s(Chemiluminescence).raw16.tif]

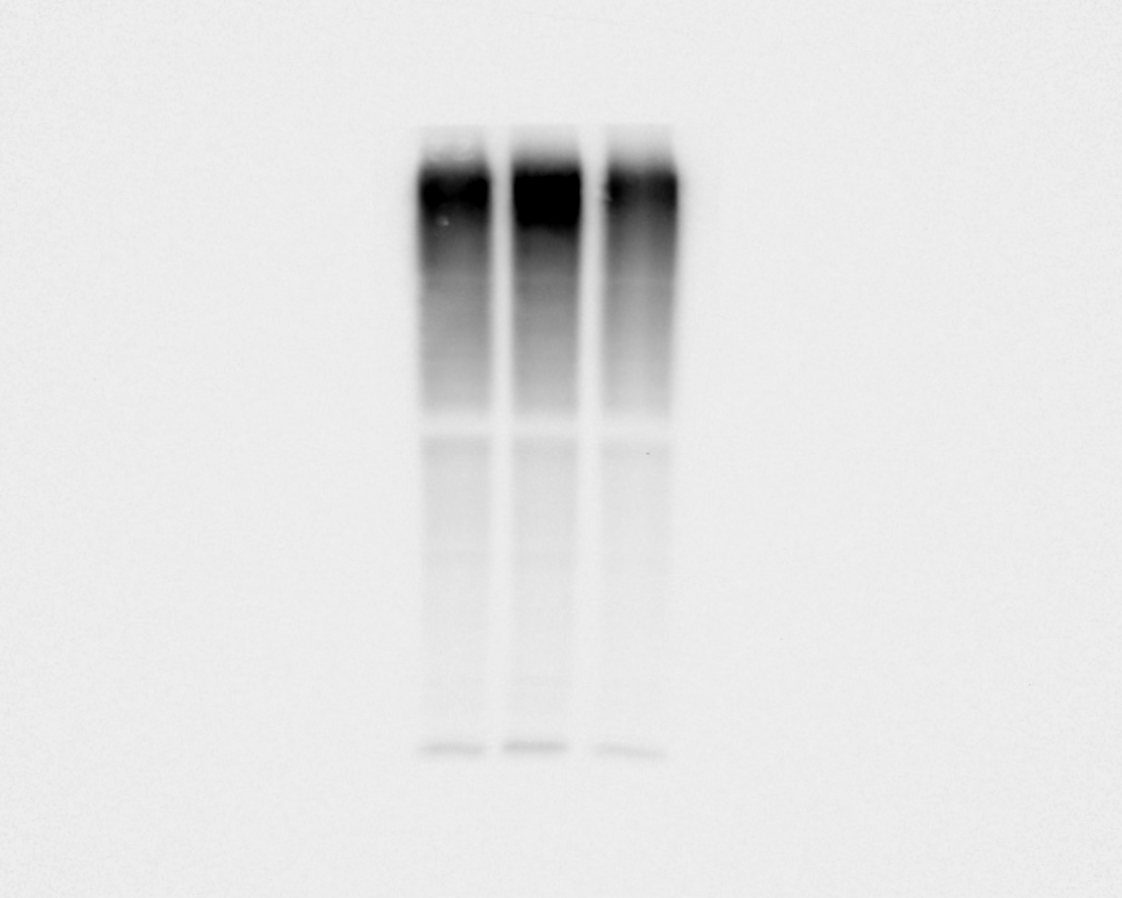

Supplement: Figure 1—figure supplement 1—source data 2. [file elife-83159-fig1-figsupp1-data2.zip › UB WCE Figure 1-figure supplement 1-source data 2/Versteeg 2022-07-01 16h05m59s 17.893s(Chemiluminescence).tif]

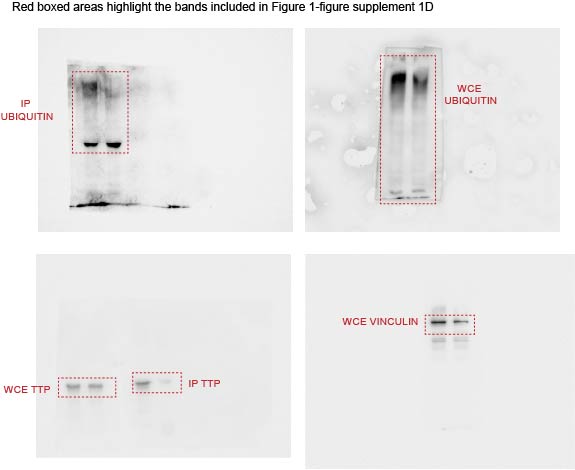

Supplement: Figure 1—figure supplement 1—source data 3. [file elife-83159-fig1-figsupp1-data3.zip › Figure 1-figure supplement 1-source data 3.jpg]

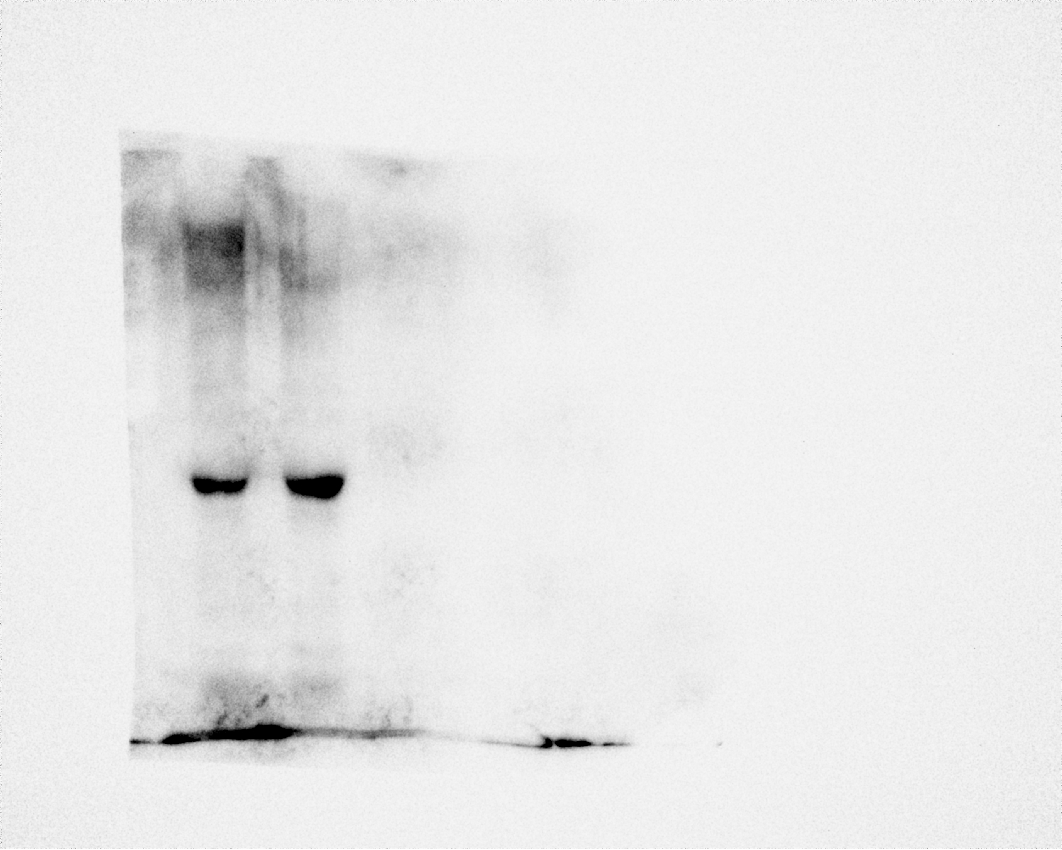

Supplement: Figure 1—figure supplement 1—source data 3. [file elife-83159-fig1-figsupp1-data3.zip › IP UBIQUITIN Figure 1-figure supplement 1- source data 3/Versteeg 2022-12-15 14h24m07s 200.000s(Chemiluminescence).jpg]

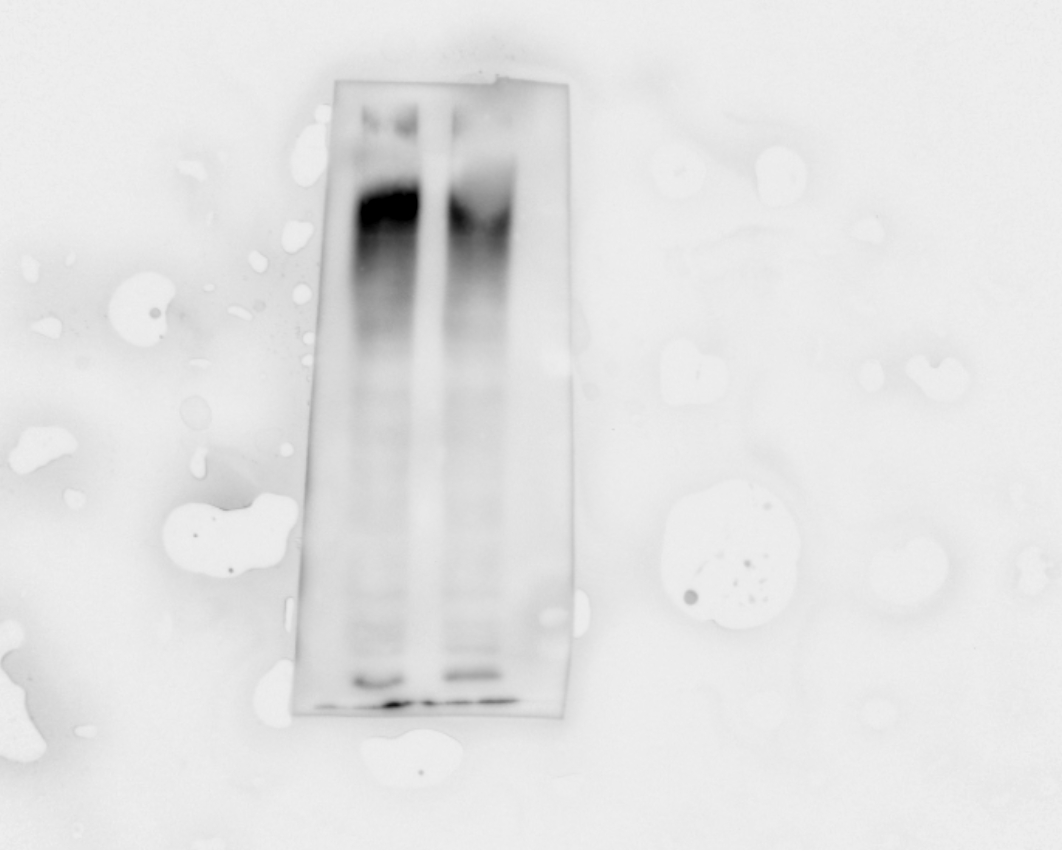

Supplement: Figure 1—figure supplement 1—source data 3. [file elife-83159-fig1-figsupp1-data3.zip › WCE UBIQUITIN Figure 1-figure supplement 1- source data 3/Versteeg 2022-12-15 10h08m45s 15.263s(Chemiluminescence).jpg]

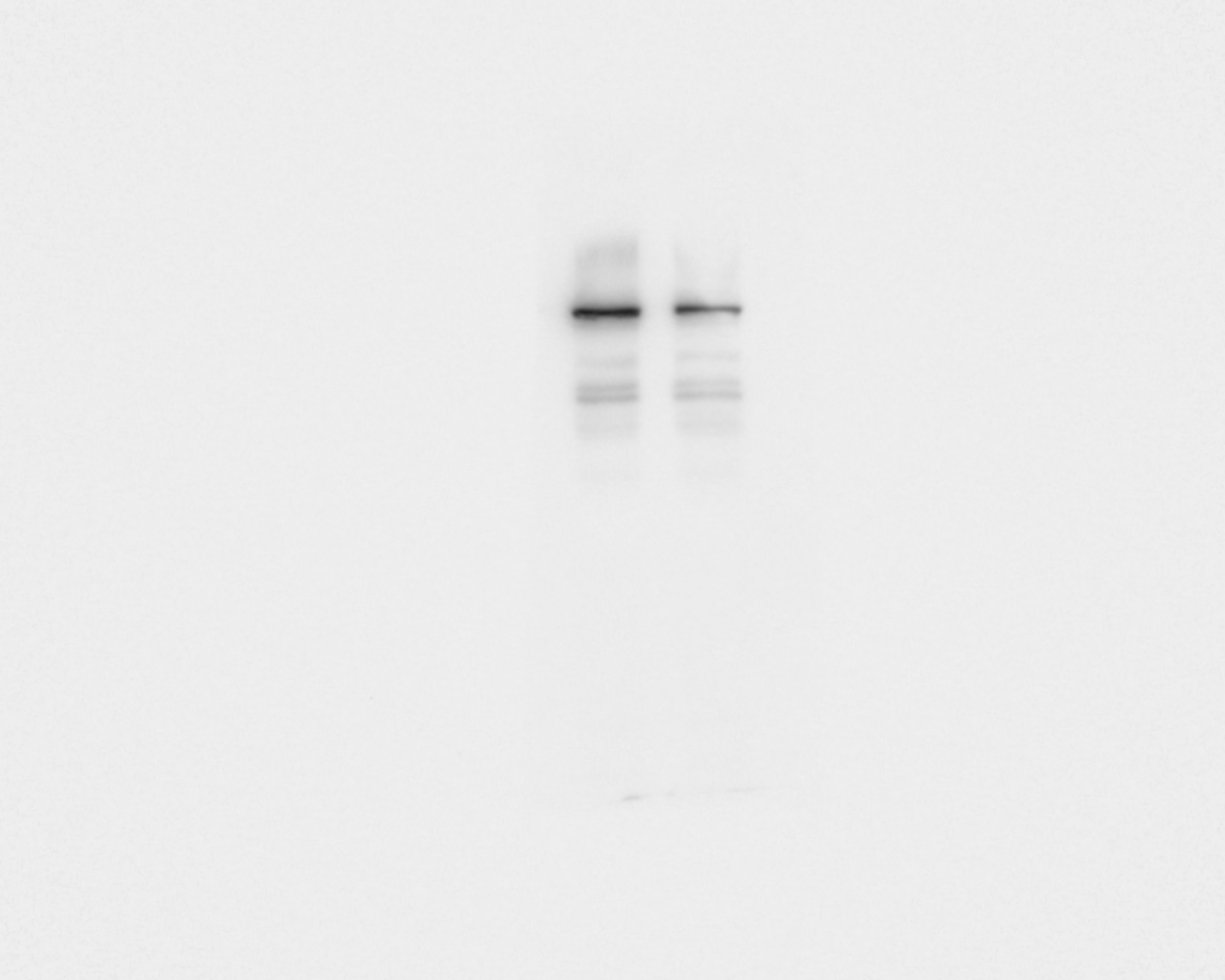

Supplement: Figure 1—figure supplement 1—source data 3. [file elife-83159-fig1-figsupp1-data3.zip › WCE VINCULIN Figure 1-figure supplement 1- source data 3/Versteeg 2022-12-21 12h06m25s 5.032s right order.tif]

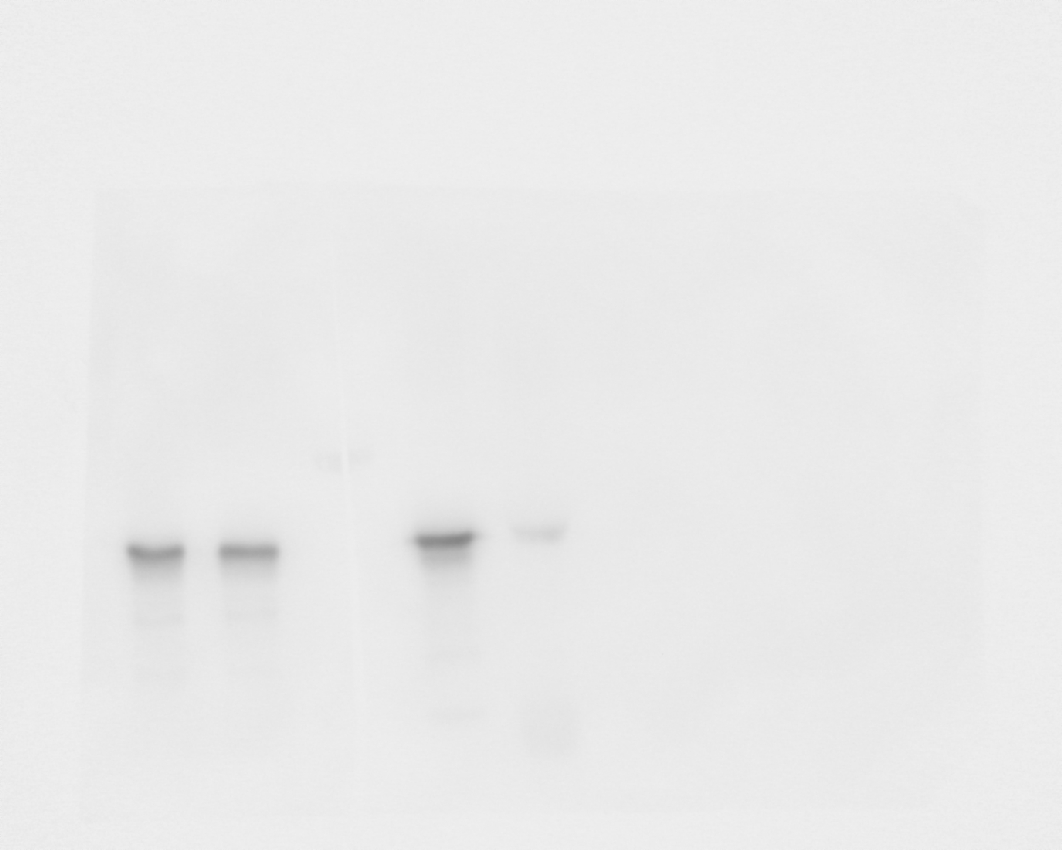

Supplement: Figure 1—figure supplement 1—source data 3. [file elife-83159-fig1-figsupp1-data3.zip › WCE+IP TTP Figure 1-figure supplement 1- source data 3/Versteeg 2022-12-16 08h48m46s 5.142s(Chemiluminescence).jpg]

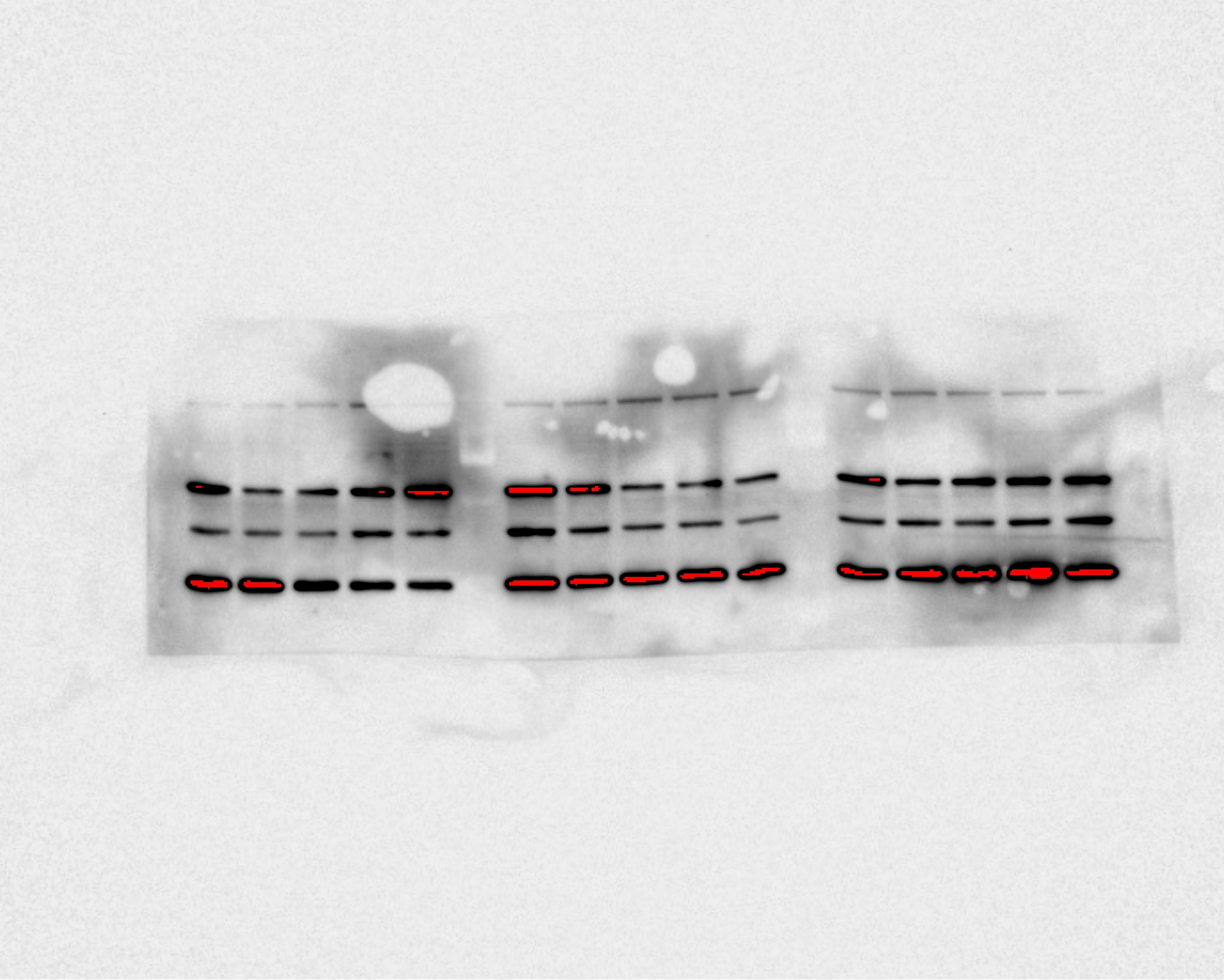

Supplement: Figure 1—figure supplement 1—source data 4. [file elife-83159-fig1-figsupp1-data4.zip › ACTIN KtoR Figure 1-figure supplement 1-source data 4/Versteeg 2022-12-22 11h17m56s 42.030s(Chemiluminescence).jpg]

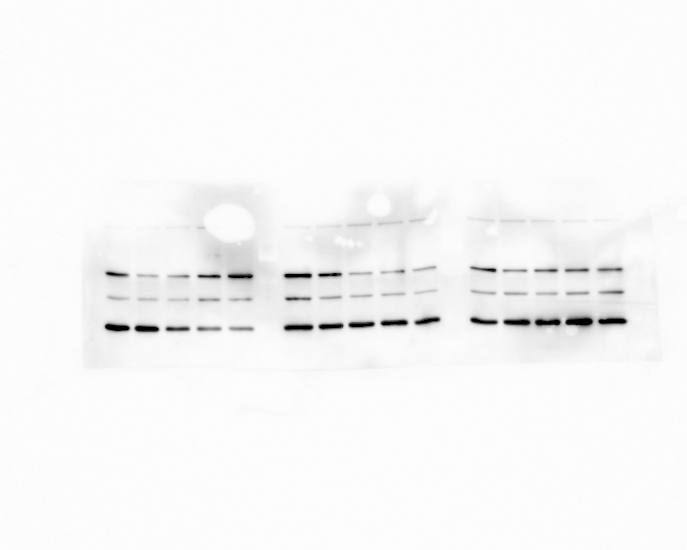

Supplement: Figure 1—figure supplement 1—source data 4. [file elife-83159-fig1-figsupp1-data4.zip › ACTIN KtoR Figure 1-figure supplement 1-source data 4/Versteeg 2022-12-22 11h17m56s 42.030s(Chemiluminescence).raw16.tif]

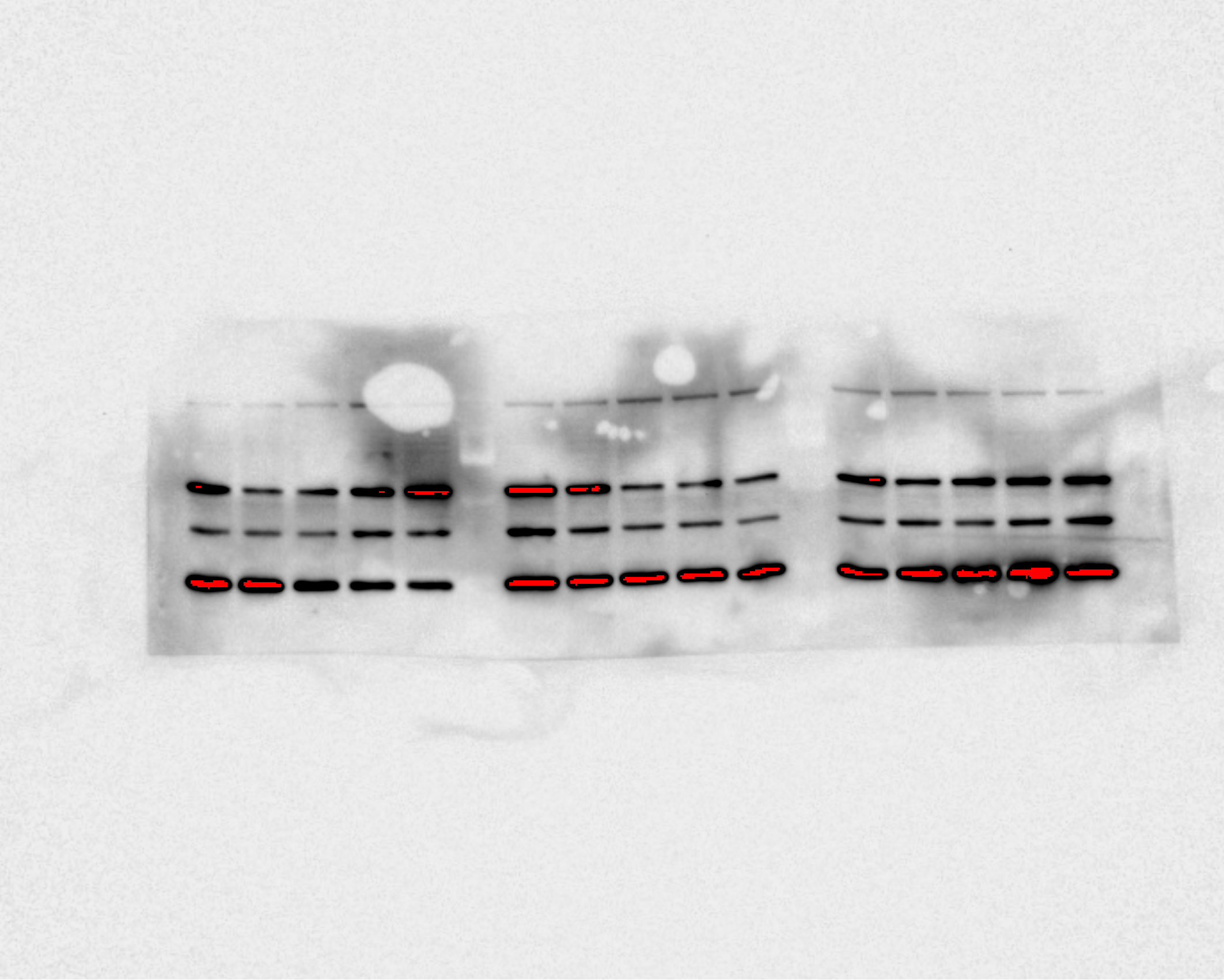

Supplement: Figure 1—figure supplement 1—source data 4. [file elife-83159-fig1-figsupp1-data4.zip › ACTIN KtoR Figure 1-figure supplement 1-source data 4/Versteeg 2022-12-22 11h17m56s 42.030s(Chemiluminescence).tif]

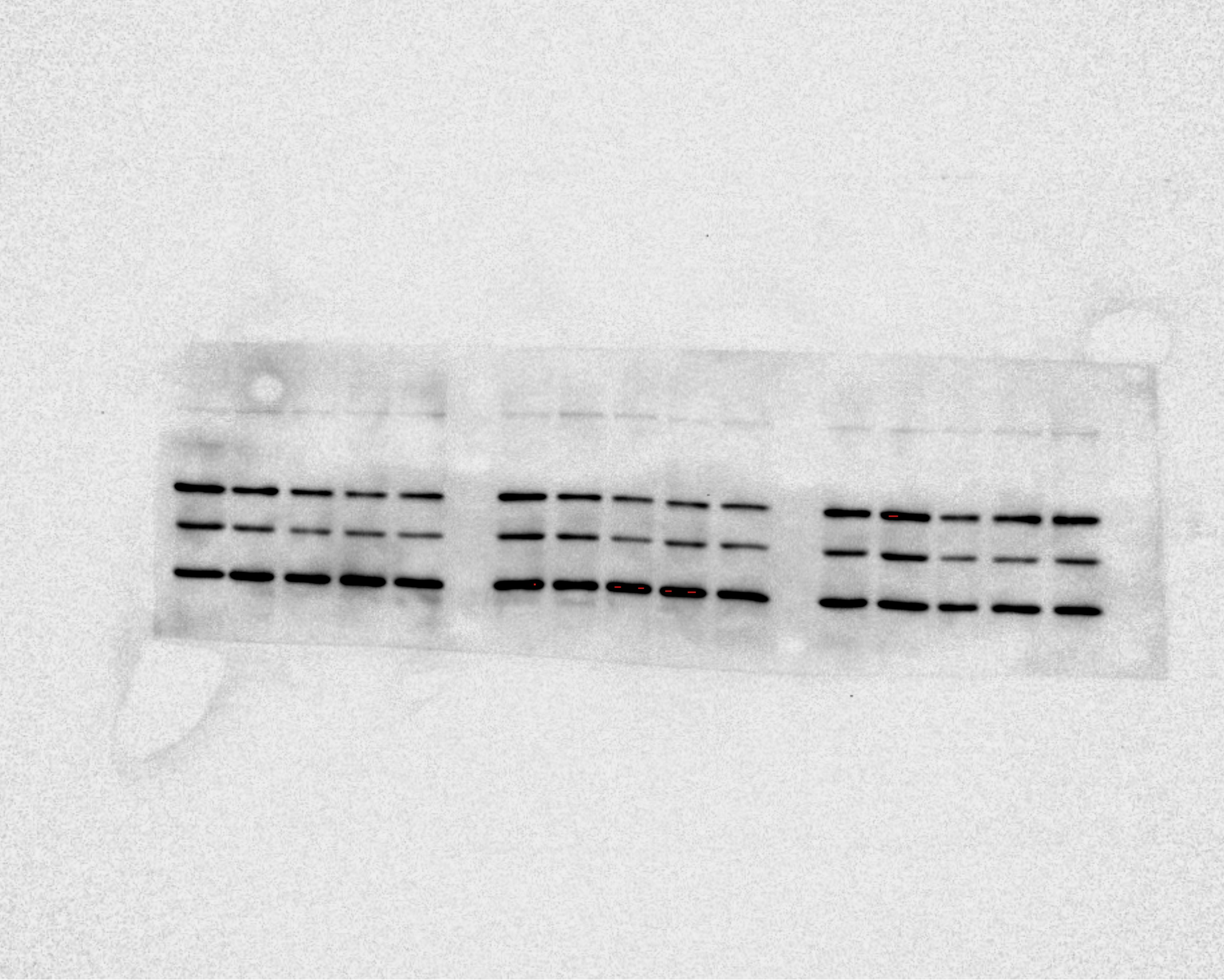

Supplement: Figure 1—figure supplement 1—source data 4. [file elife-83159-fig1-figsupp1-data4.zip › ACTIN wtTTP Figure 1-figure supplement 1-source data 4/Versteeg 2022-12-22 11h25m18s 119.987s(Chemiluminescence).jpg]

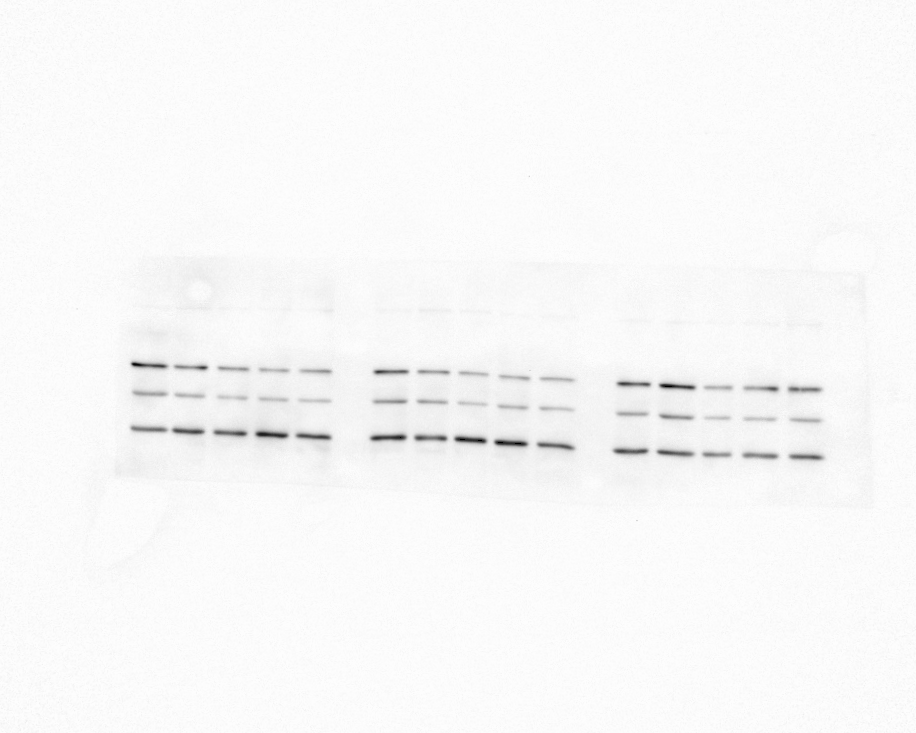

Supplement: Figure 1—figure supplement 1—source data 4. [file elife-83159-fig1-figsupp1-data4.zip › ACTIN wtTTP Figure 1-figure supplement 1-source data 4/Versteeg 2022-12-22 11h25m18s 119.987s(Chemiluminescence).raw16.tif]

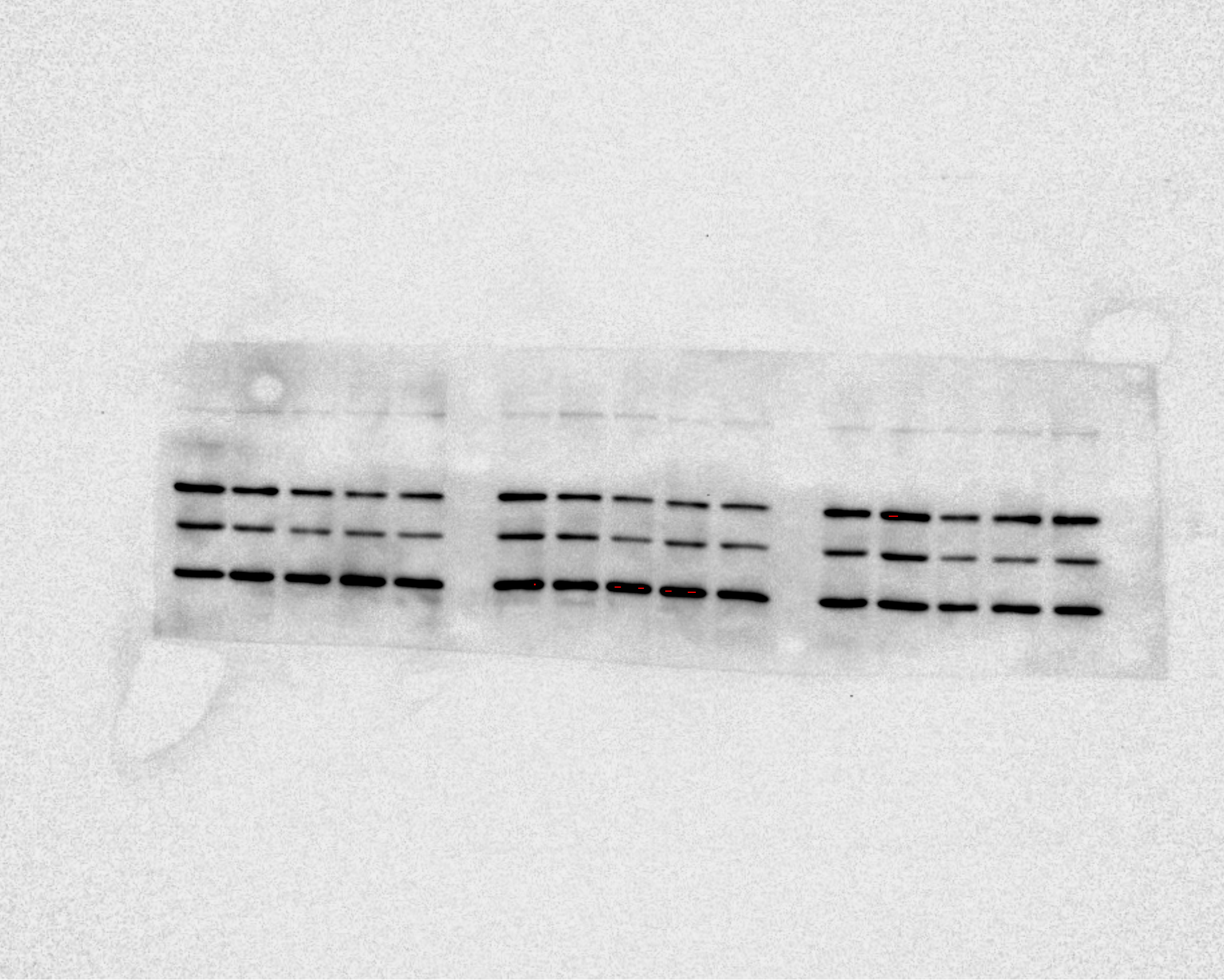

Supplement: Figure 1—figure supplement 1—source data 4. [file elife-83159-fig1-figsupp1-data4.zip › ACTIN wtTTP Figure 1-figure supplement 1-source data 4/Versteeg 2022-12-22 11h25m18s 119.987s(Chemiluminescence).tif]

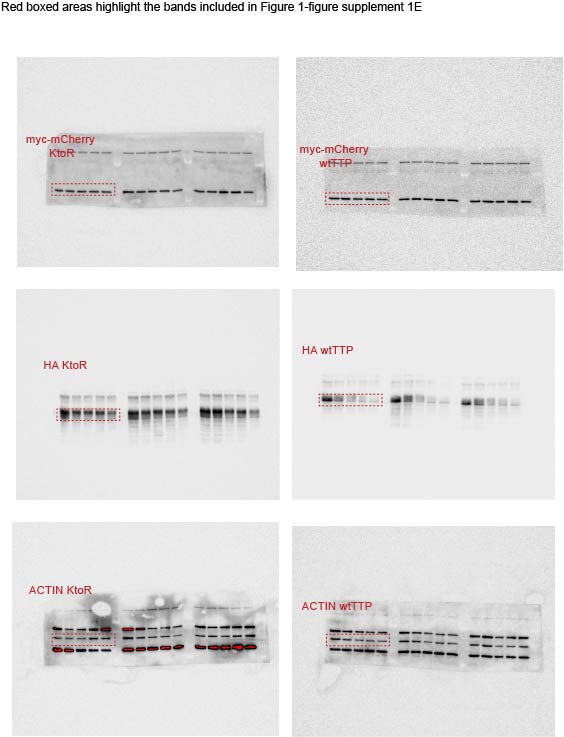

Supplement: Figure 1—figure supplement 1—source data 4. [file elife-83159-fig1-figsupp1-data4.zip › Figure 1-figure supplement 1-source data 4.jpg]

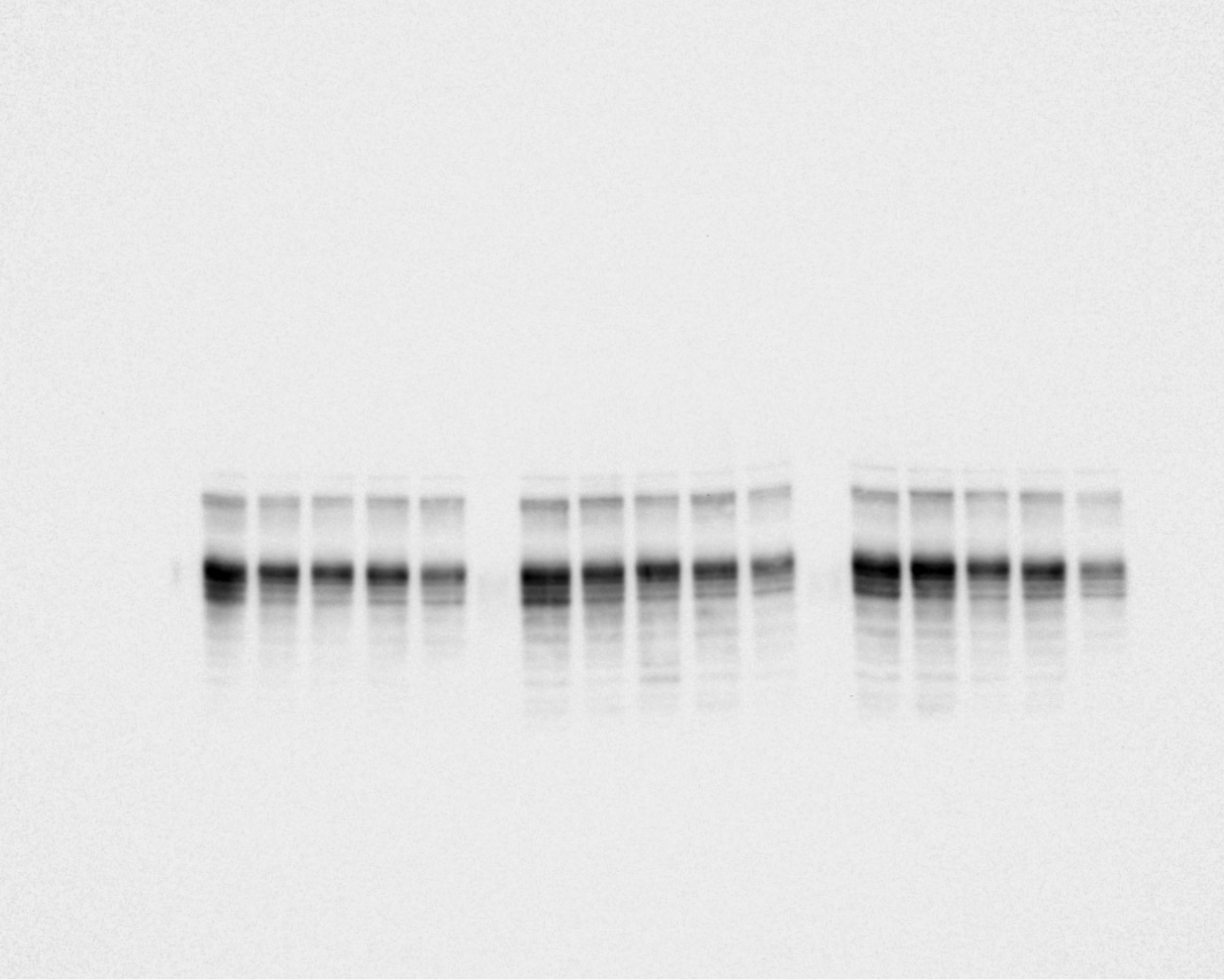

Supplement: Figure 1—figure supplement 1—source data 4. [file elife-83159-fig1-figsupp1-data4.zip › HA KtoR Figure 1-figure supplement 1-source data 4/Versteeg 2022-12-21 11h10m58s 57.927s(Chemiluminescence).jpg]

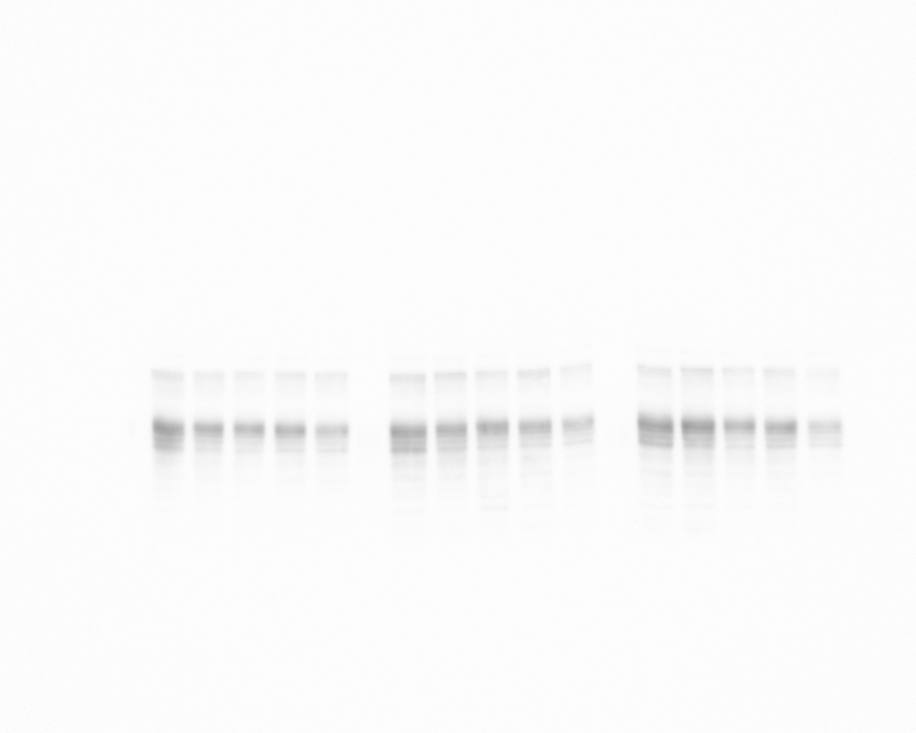

Supplement: Figure 1—figure supplement 1—source data 4. [file elife-83159-fig1-figsupp1-data4.zip › HA KtoR Figure 1-figure supplement 1-source data 4/Versteeg 2022-12-21 11h10m58s 57.927s(Chemiluminescence).raw16.tif]

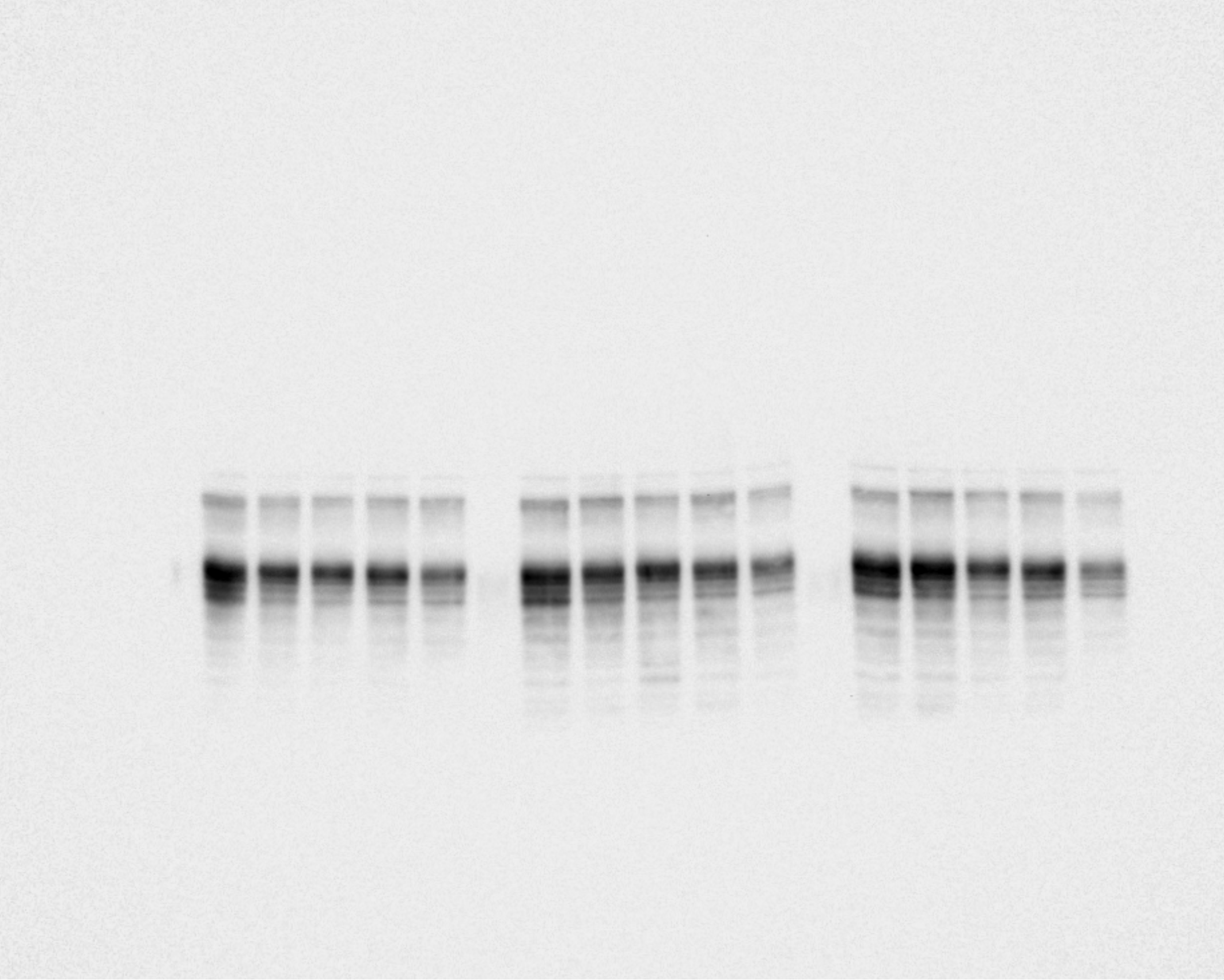

Supplement: Figure 1—figure supplement 1—source data 4. [file elife-83159-fig1-figsupp1-data4.zip › HA KtoR Figure 1-figure supplement 1-source data 4/Versteeg 2022-12-21 11h10m58s 57.927s(Chemiluminescence).tif]

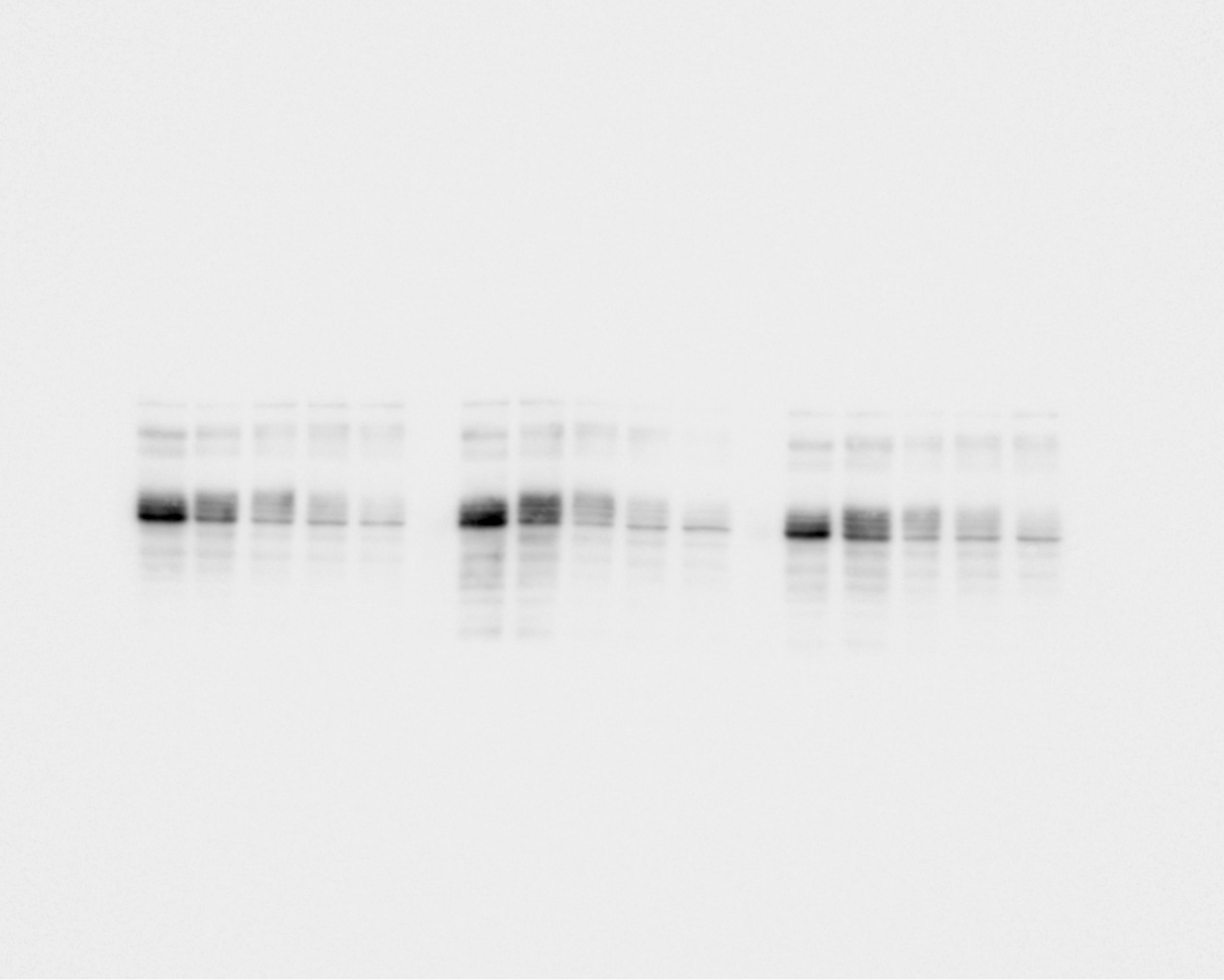

Supplement: Figure 1—figure supplement 1—source data 4. [file elife-83159-fig1-figsupp1-data4.zip › HA wtTTP Figure 1-figure supplement 1-source data 4/Versteeg 2022-12-21 11h54m08s 5.032s(Chemiluminescence).jpg]

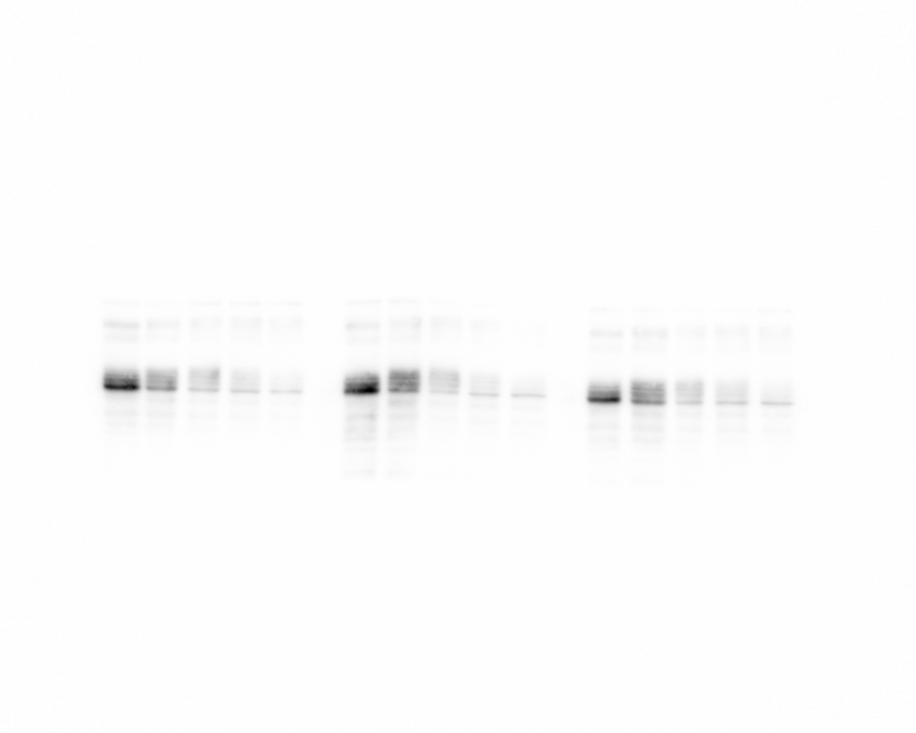

Supplement: Figure 1—figure supplement 1—source data 4. [file elife-83159-fig1-figsupp1-data4.zip › HA wtTTP Figure 1-figure supplement 1-source data 4/Versteeg 2022-12-21 11h54m08s 5.032s(Chemiluminescence).raw16.tif]

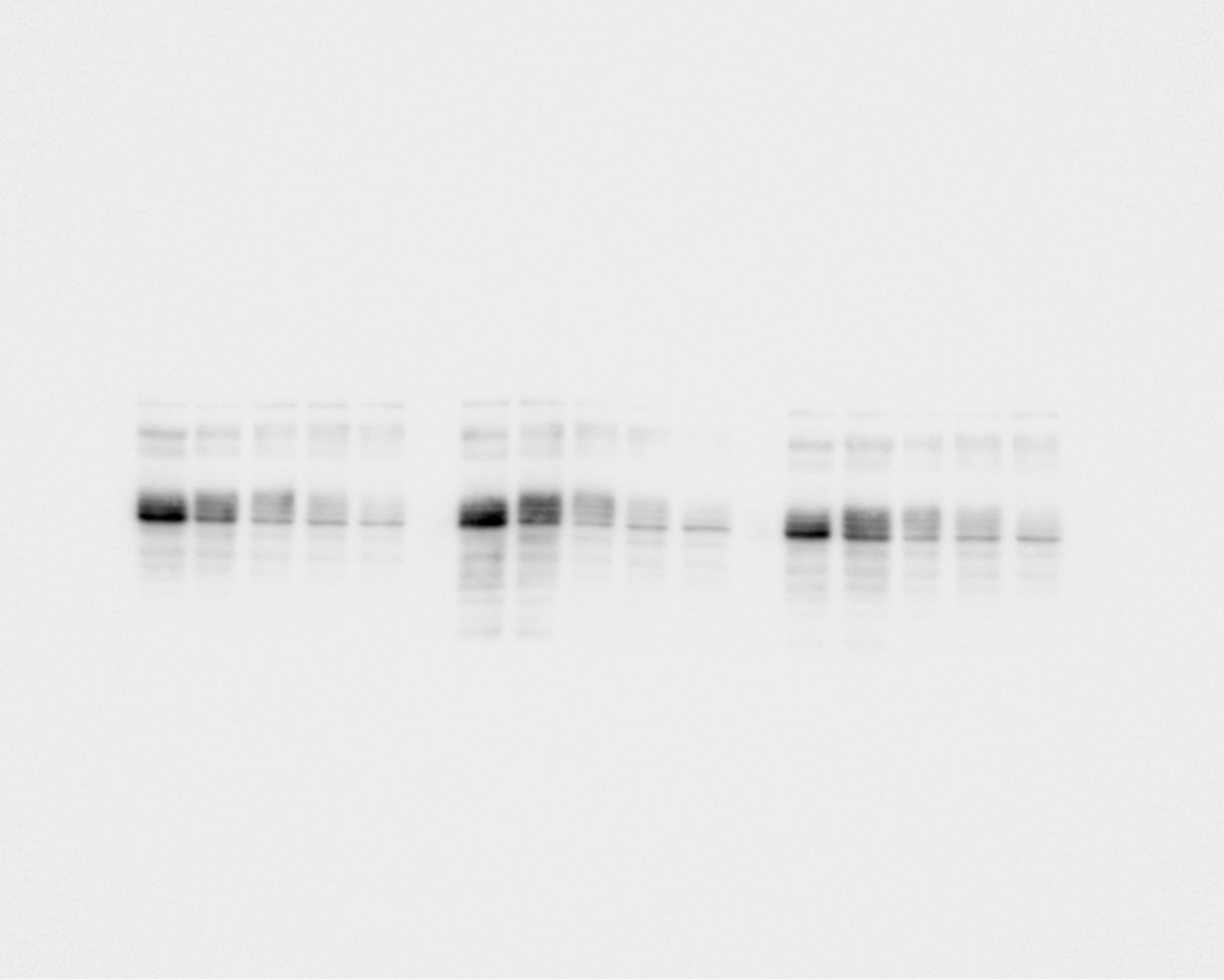

Supplement: Figure 1—figure supplement 1—source data 4. [file elife-83159-fig1-figsupp1-data4.zip › HA wtTTP Figure 1-figure supplement 1-source data 4/Versteeg 2022-12-21 11h54m08s 5.032s(Chemiluminescence).tif]

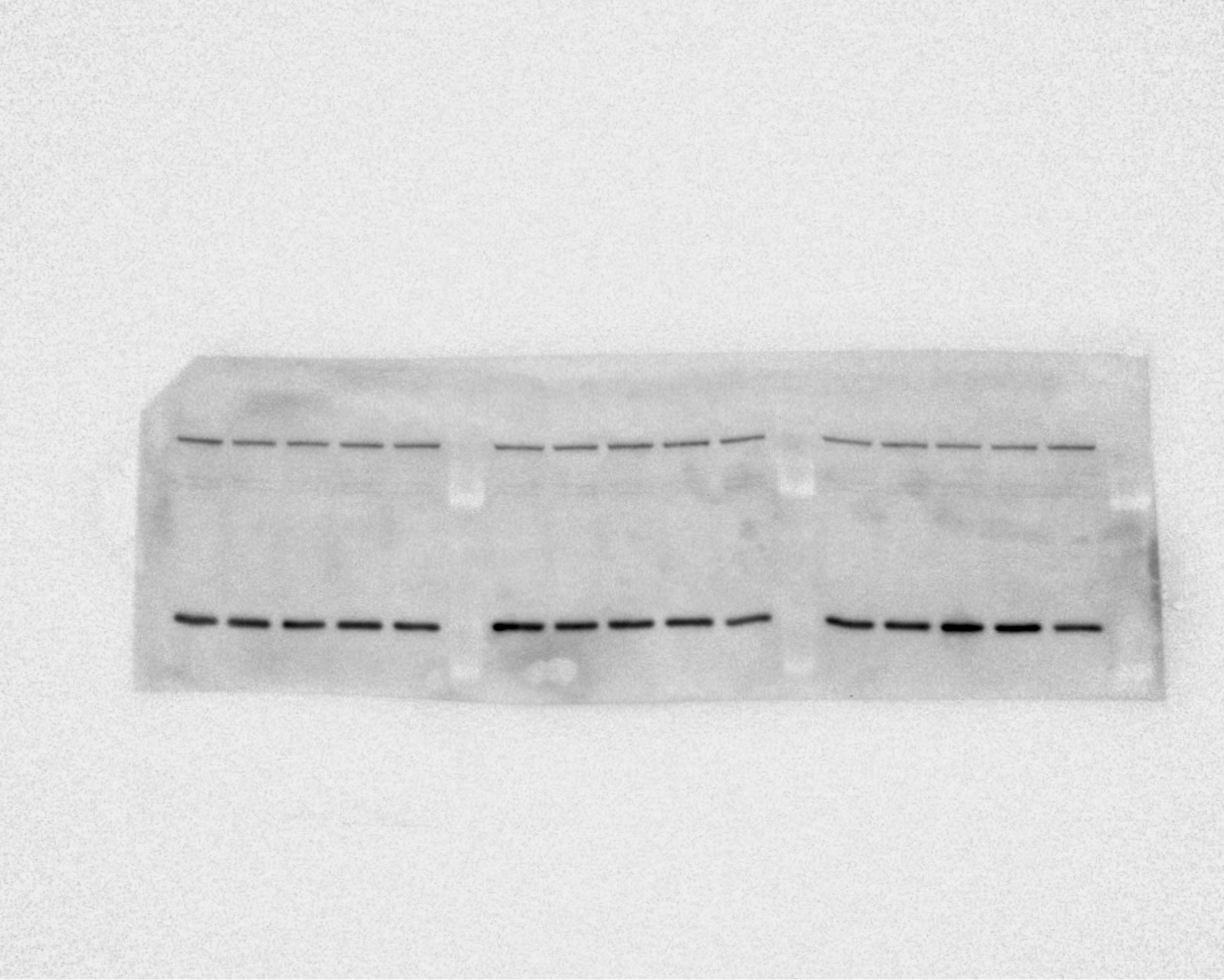

Supplement: Figure 1—figure supplement 1—source data 4. [file elife-83159-fig1-figsupp1-data4.zip › myc-mCherry KtoR Figure 1-figure supplement 1-source data 4/Versteeg 2022-12-21 14h58m03s 51.008s(Chemiluminescence).jpg]

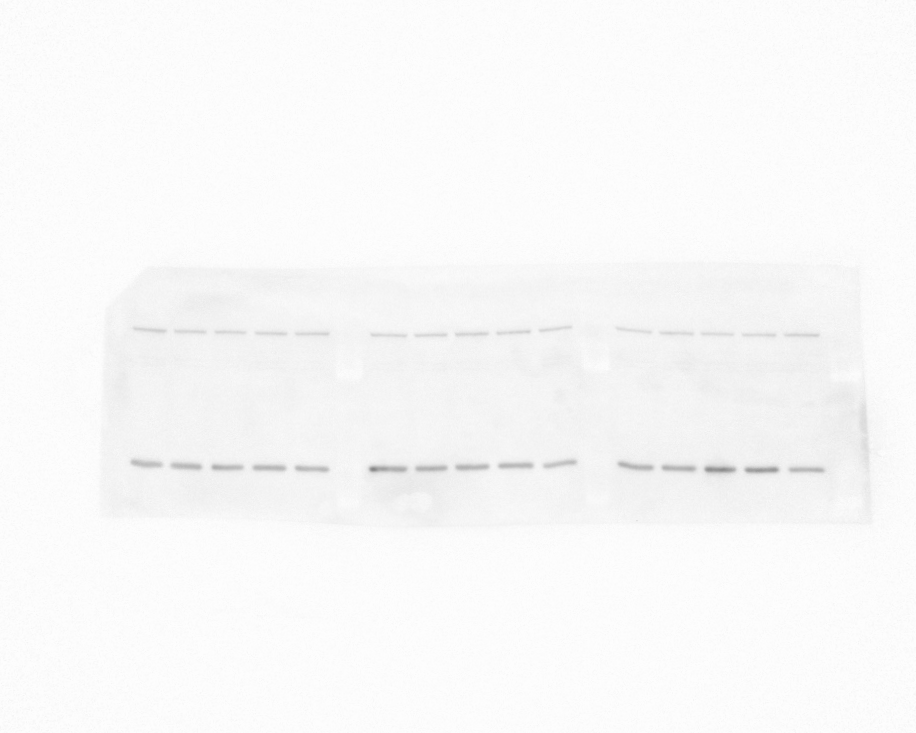

Supplement: Figure 1—figure supplement 1—source data 4. [file elife-83159-fig1-figsupp1-data4.zip › myc-mCherry KtoR Figure 1-figure supplement 1-source data 4/Versteeg 2022-12-21 14h58m03s 51.008s(Chemiluminescence).raw16.tif]

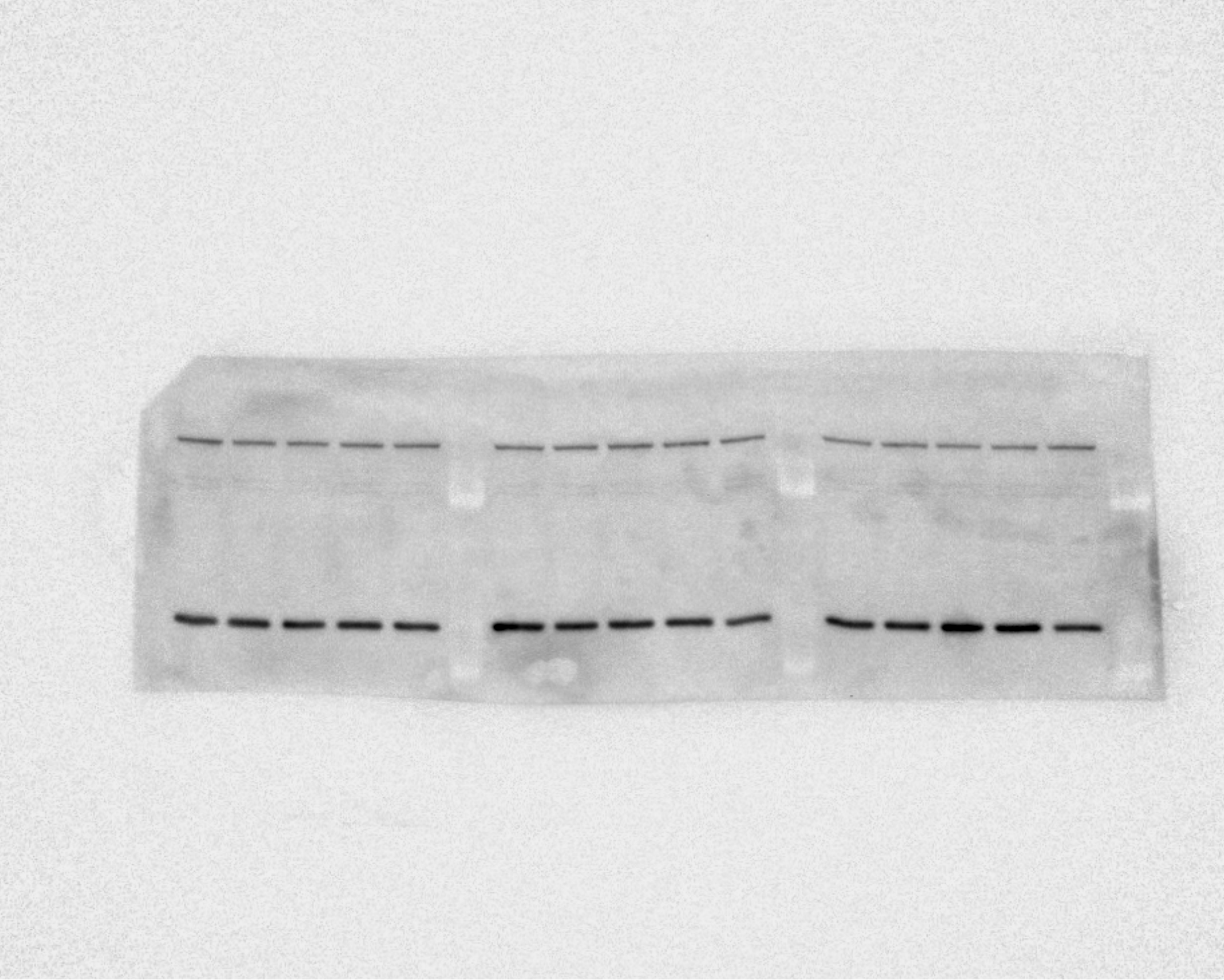

Supplement: Figure 1—figure supplement 1—source data 4. [file elife-83159-fig1-figsupp1-data4.zip › myc-mCherry KtoR Figure 1-figure supplement 1-source data 4/Versteeg 2022-12-21 14h58m03s 51.008s(Chemiluminescence).tif]

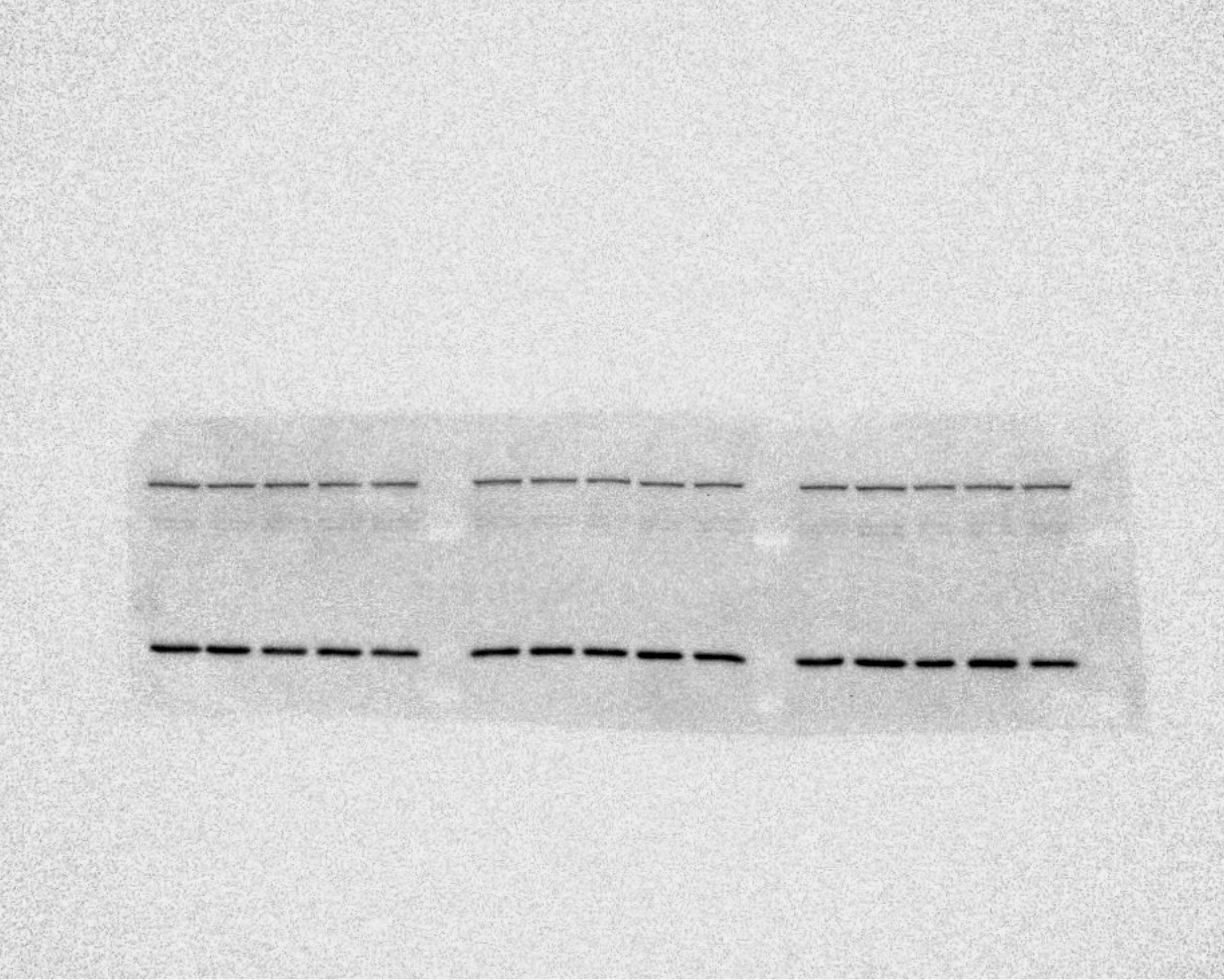

Supplement: Figure 1—figure supplement 1—source data 4. [file elife-83159-fig1-figsupp1-data4.zip › myc-mCherry wtTTP Figure 1-figure supplement 1-source data 4/Versteeg 2022-12-21 15h06m47s 106.928s(Chemiluminescence).jpg]

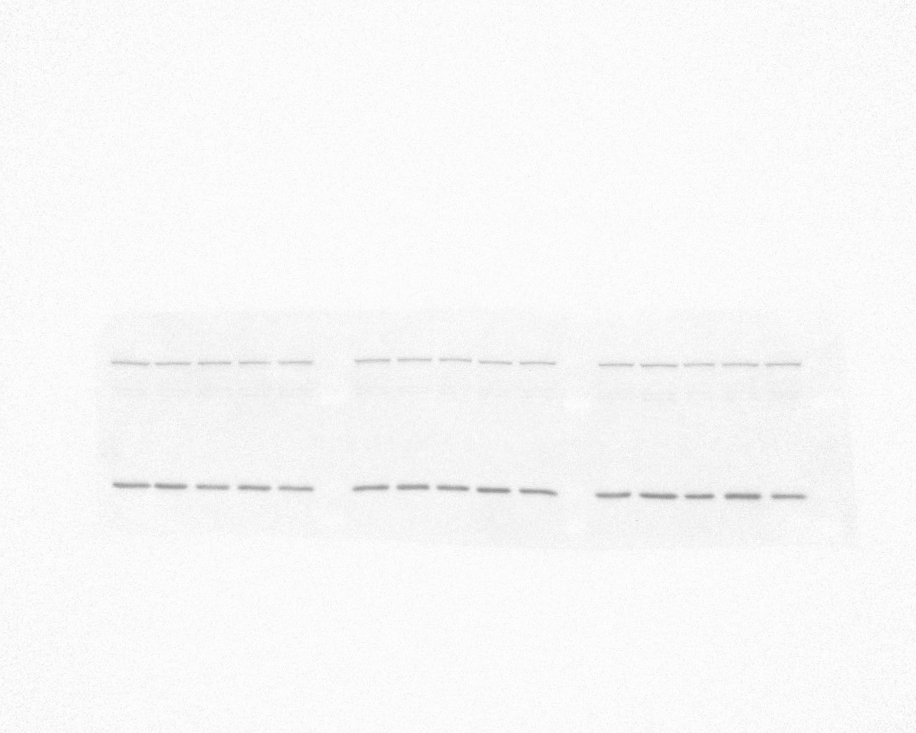

Supplement: Figure 1—figure supplement 1—source data 4. [file elife-83159-fig1-figsupp1-data4.zip › myc-mCherry wtTTP Figure 1-figure supplement 1-source data 4/Versteeg 2022-12-21 15h06m47s 106.928s(Chemiluminescence).raw16.tif]

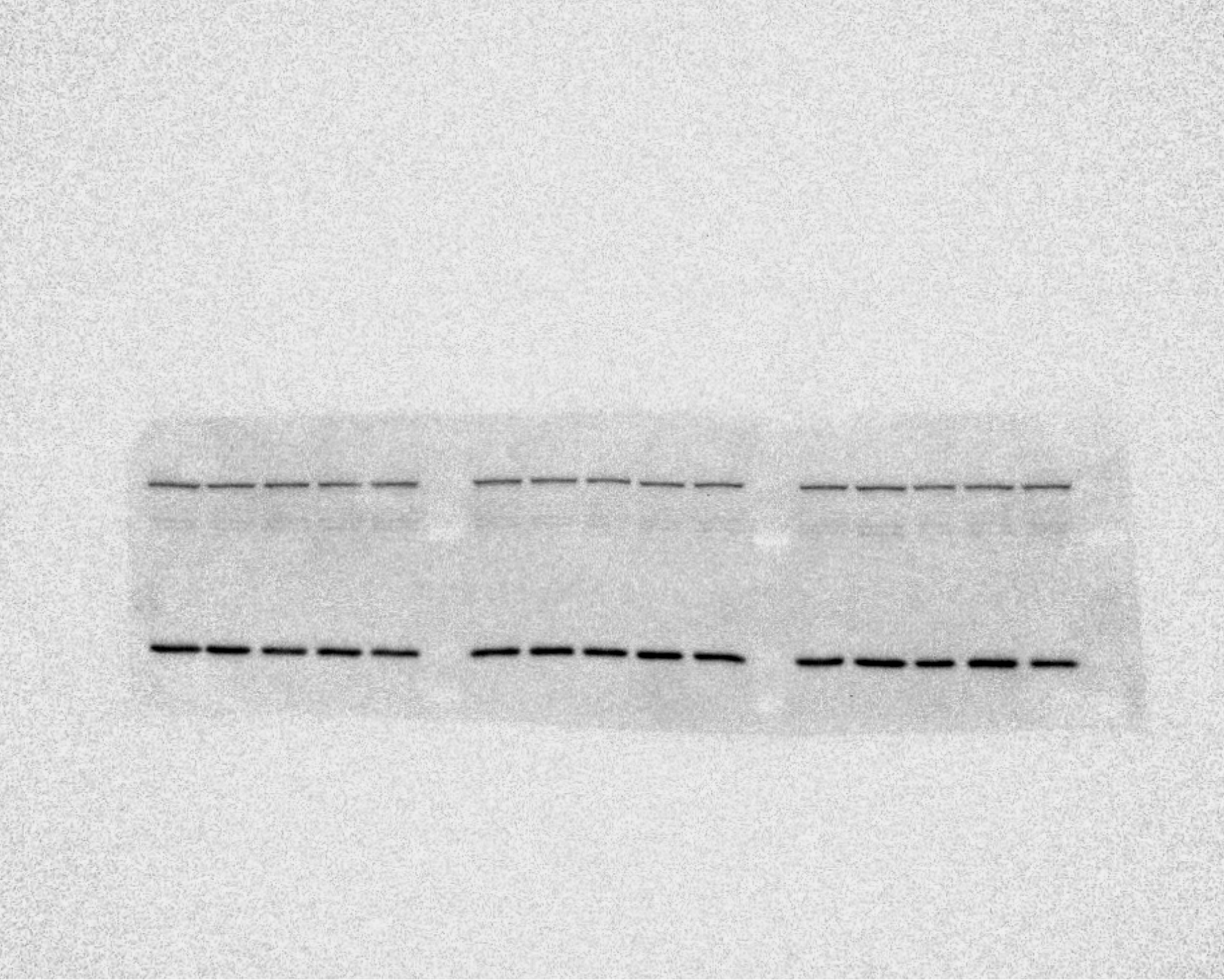

Supplement: Figure 1—figure supplement 1—source data 4. [file elife-83159-fig1-figsupp1-data4.zip › myc-mCherry wtTTP Figure 1-figure supplement 1-source data 4/Versteeg 2022-12-21 15h06m47s 106.928s(Chemiluminescence).tif]

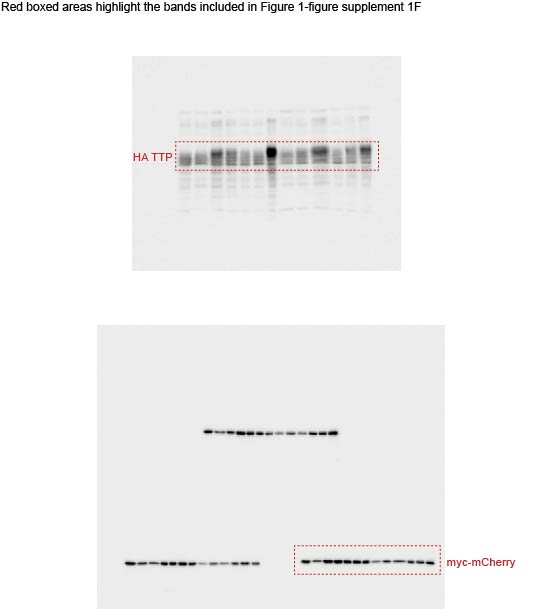

Supplement: Figure 1—figure supplement 1—source data 5. [file elife-83159-fig1-figsupp1-data5.zip › Figure 1-figure supplement 1-source data 5.jpg]

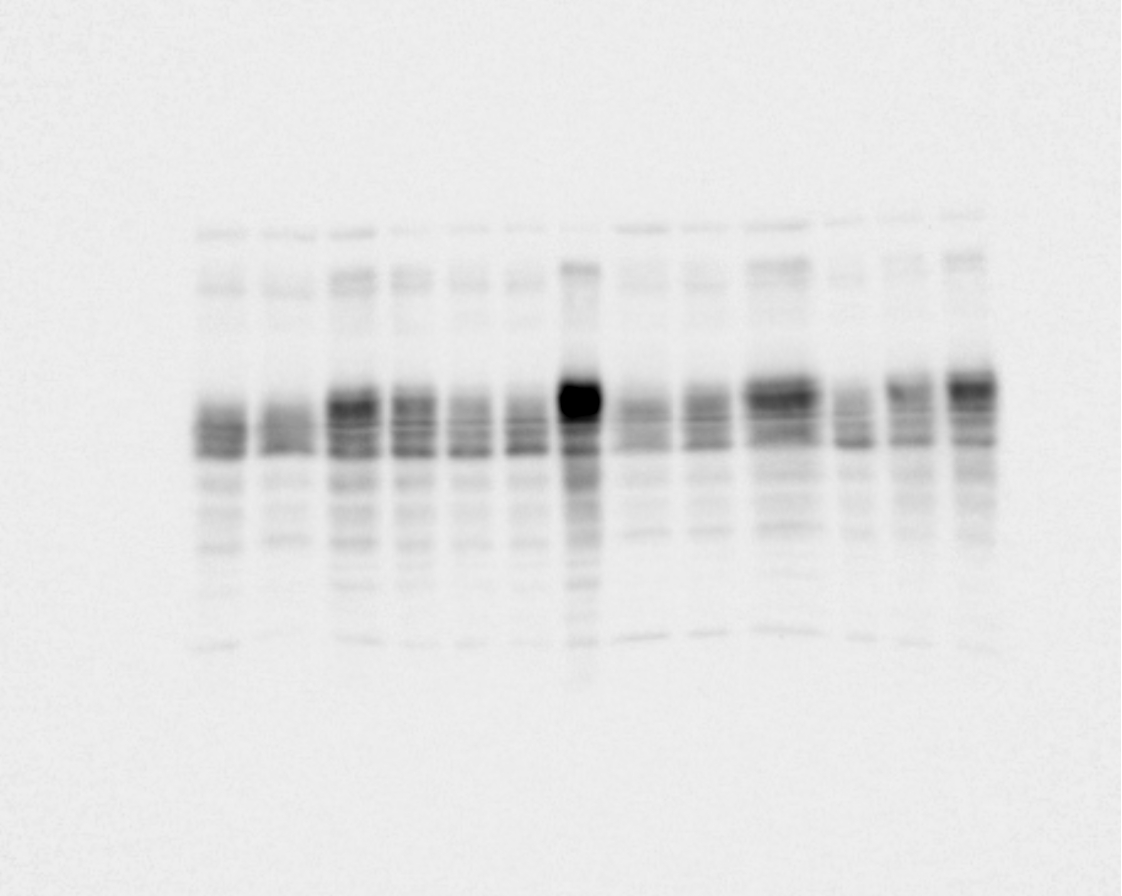

Supplement: Figure 1—figure supplement 1—source data 5. [file elife-83159-fig1-figsupp1-data5.zip › HA-TTP Figure 1-figure supplement 1-source data 5/Versteeg 2023-01-18 12h51m22s 7.102s(Chemiluminescence).jpg]

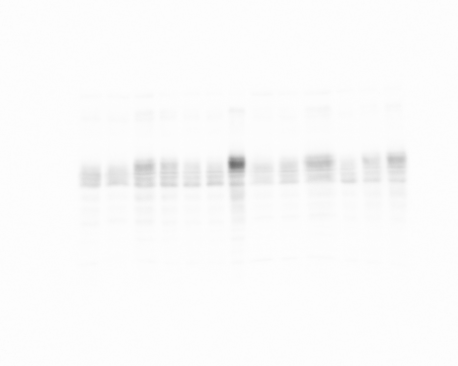

Supplement: Figure 1—figure supplement 1—source data 5. [file elife-83159-fig1-figsupp1-data5.zip › HA-TTP Figure 1-figure supplement 1-source data 5/Versteeg 2023-01-18 12h51m22s 7.102s(Chemiluminescence).raw16.tif]

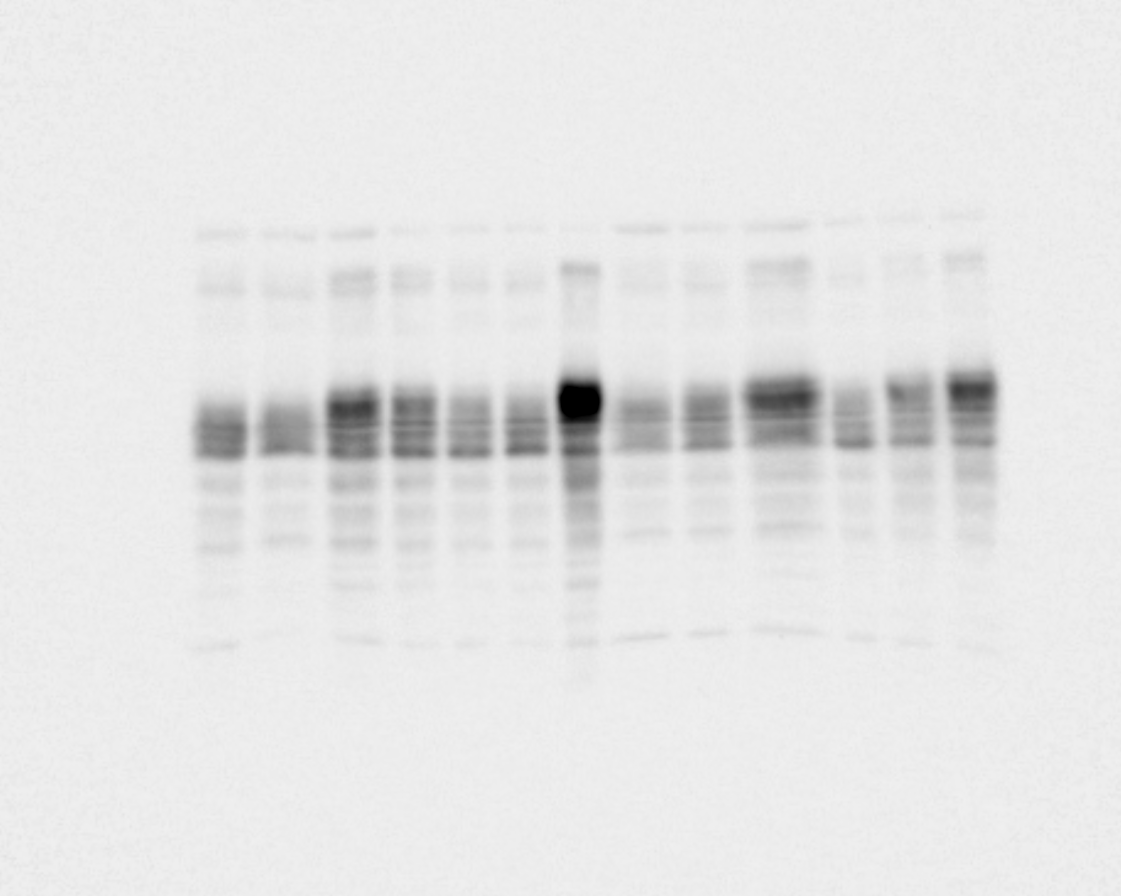

Supplement: Figure 1—figure supplement 1—source data 5. [file elife-83159-fig1-figsupp1-data5.zip › HA-TTP Figure 1-figure supplement 1-source data 5/Versteeg 2023-01-18 12h51m22s 7.102s(Chemiluminescence).tif]

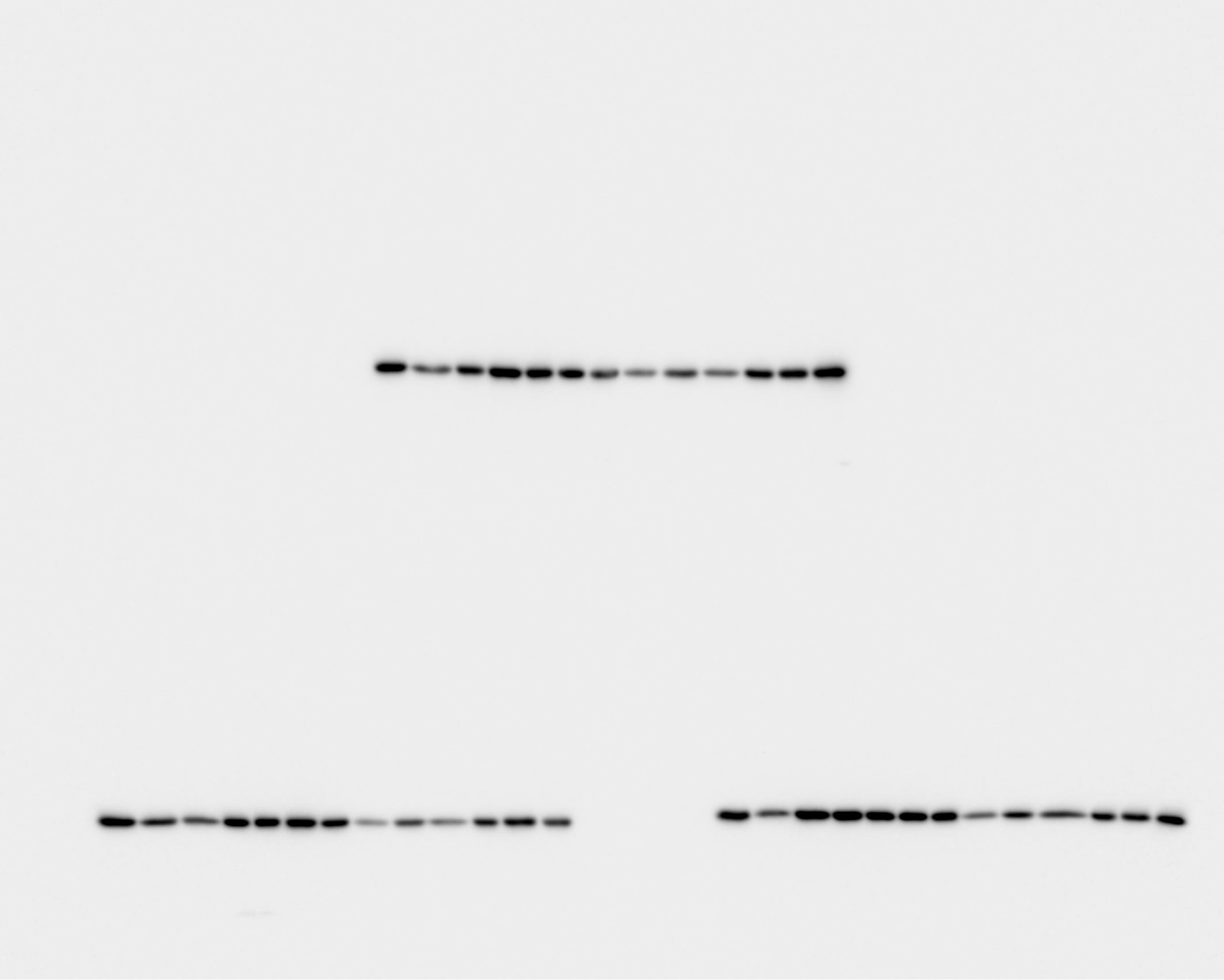

Supplement: Figure 1—figure supplement 1—source data 5. [file elife-83159-fig1-figsupp1-data5.zip › myc-mCherry Figure 1-figure supplement 1-source data 5/Versteeg 2023-01-19 13h33m25s 1.000s(Chemiluminescence).jpg]

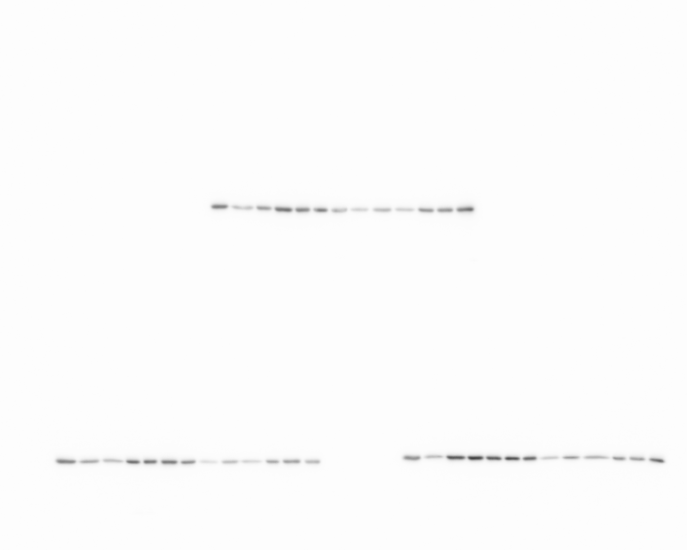

Supplement: Figure 1—figure supplement 1—source data 5. [file elife-83159-fig1-figsupp1-data5.zip › myc-mCherry Figure 1-figure supplement 1-source data 5/Versteeg 2023-01-19 13h33m25s 1.000s(Chemiluminescence).raw16.tif]

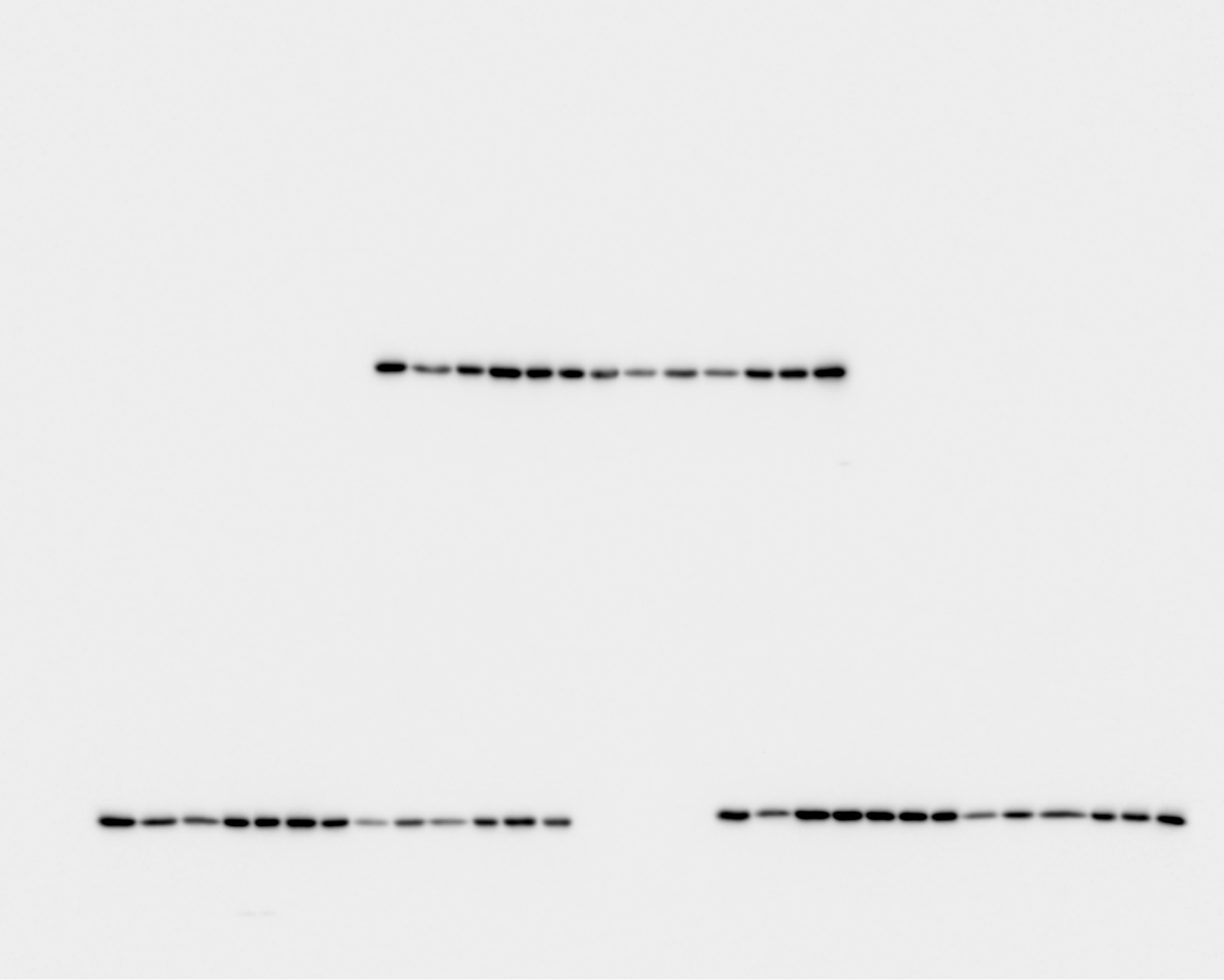

Supplement: Figure 1—figure supplement 1—source data 5. [file elife-83159-fig1-figsupp1-data5.zip › myc-mCherry Figure 1-figure supplement 1-source data 5/Versteeg 2023-01-19 13h33m25s 1.000s(Chemiluminescence).tif]
