# Supplementary figures and images for: HUWE1 controls tristetraprolin proteasomal degradation by regulating its phosphorylation (part 2 of 4)
Source: eLife. 2023 Mar 24;12:e83159. doi: 10.7554/eLife.83159 (PMC10038661; doi:10.7554/eLife.83159)

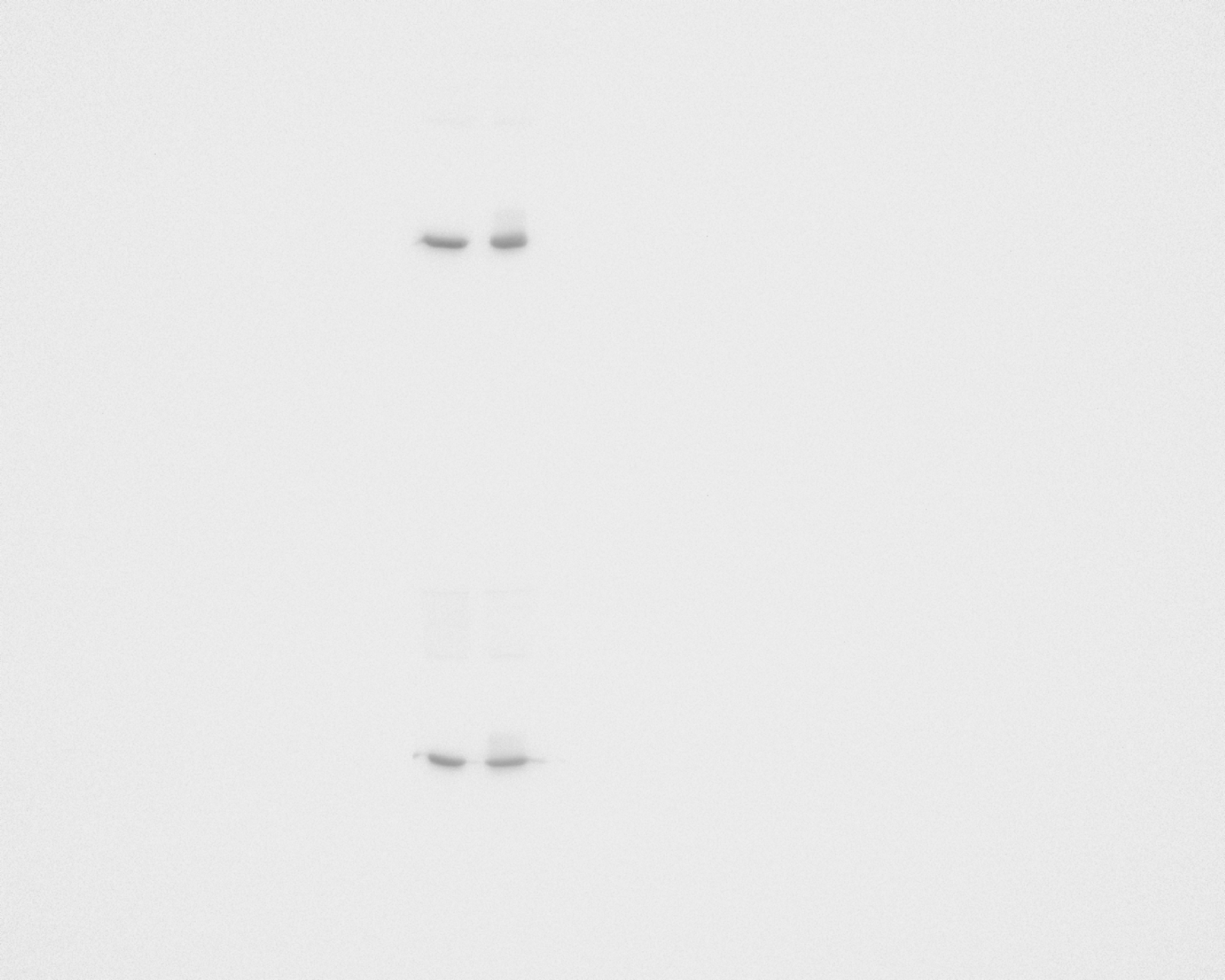

Supplement: Figure 1—figure supplement 1—source data 6. [file elife-83159-fig1-figsupp1-data6.zip › ACTIN WCE K48 and K63 Figure 1-figure supplement 1-source data 6/Versteeg 2023-02-03 10h30m08s 94.780s(Chemiluminescence).jpg]

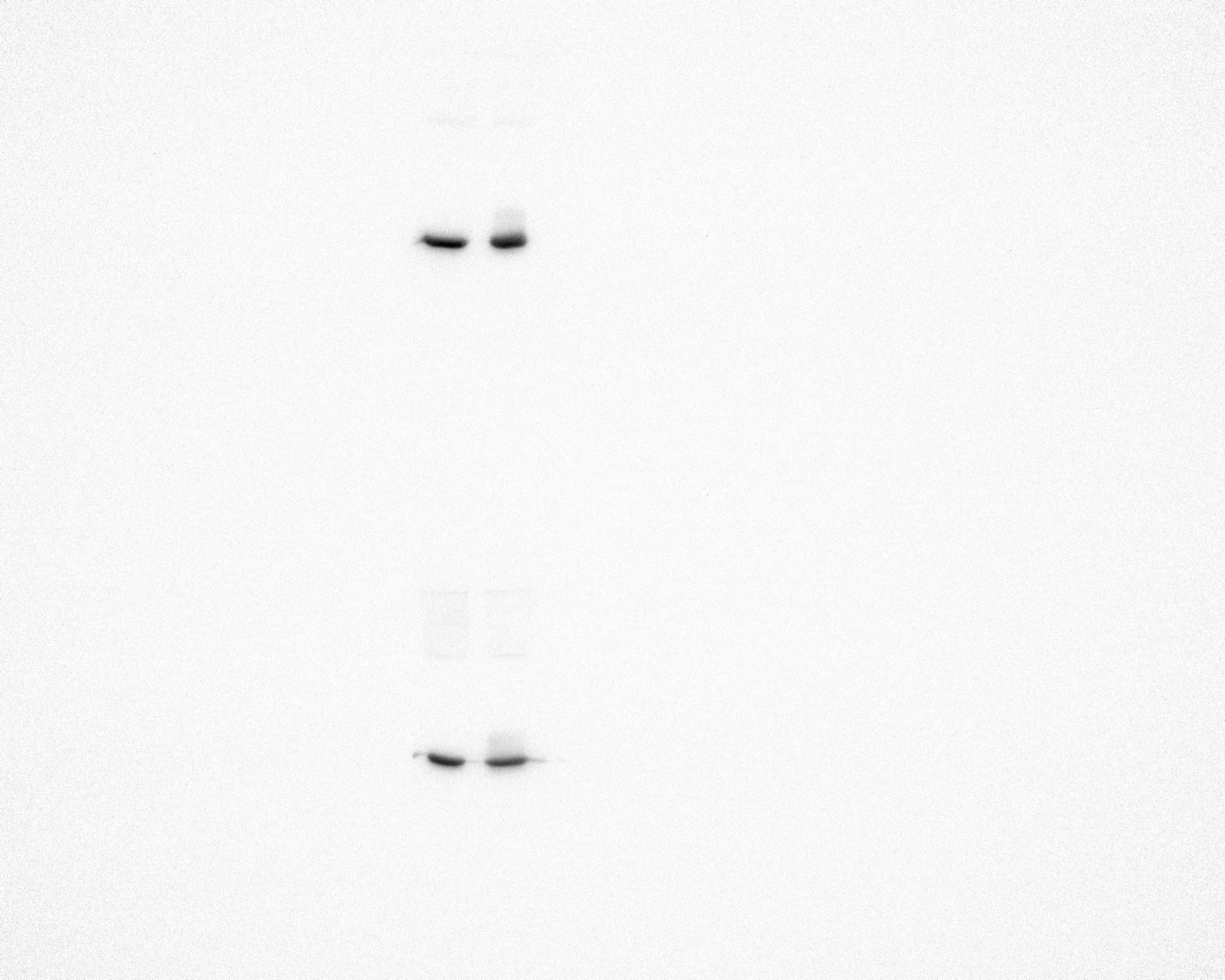

Supplement: Figure 1—figure supplement 1—source data 6. [file elife-83159-fig1-figsupp1-data6.zip › ACTIN WCE K48 and K63 Figure 1-figure supplement 1-source data 6/Versteeg 2023-02-03 10h30m08s 94.780s(Chemiluminescence).raw16.tif]

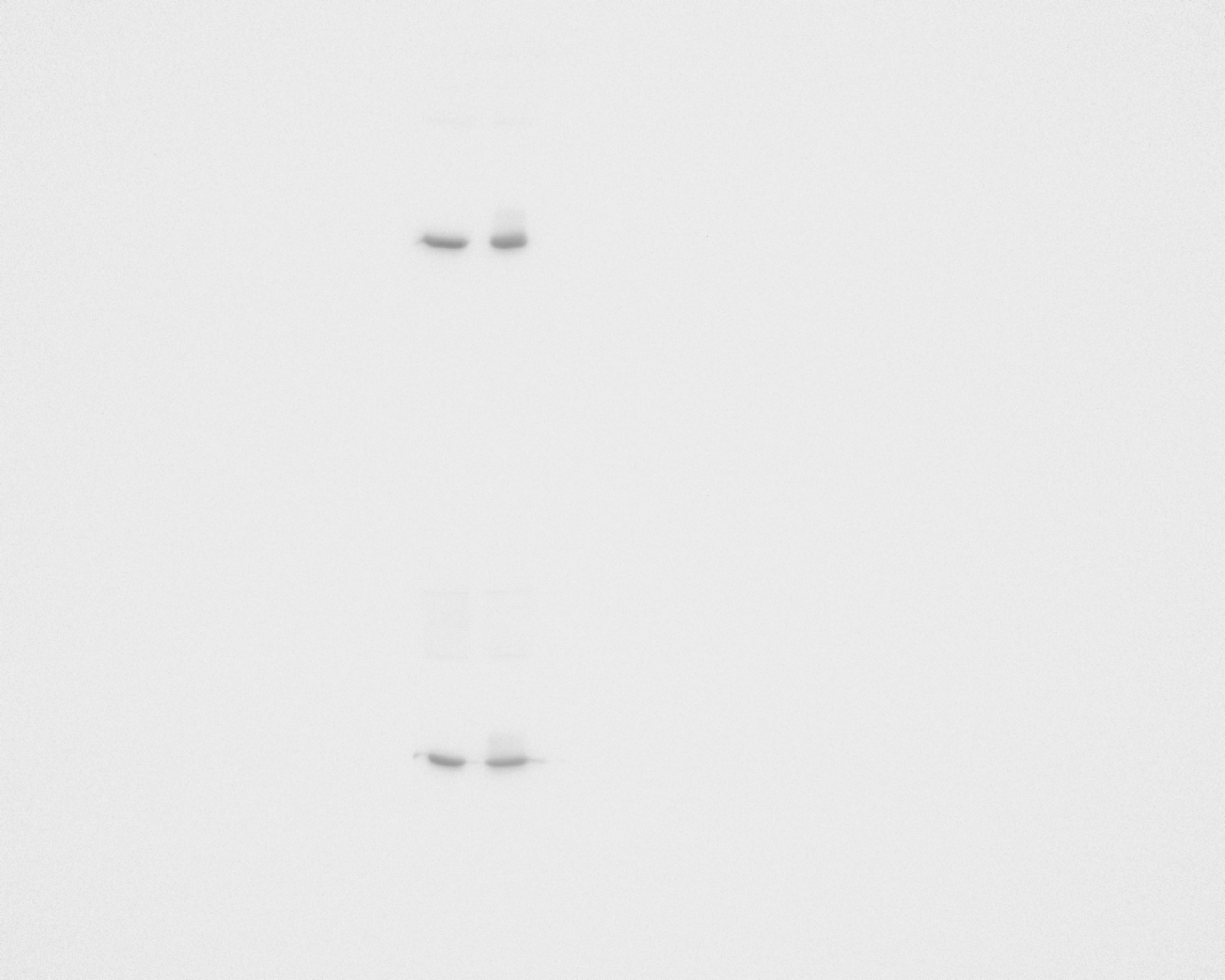

Supplement: Figure 1—figure supplement 1—source data 6. [file elife-83159-fig1-figsupp1-data6.zip › ACTIN WCE K48 and K63 Figure 1-figure supplement 1-source data 6/Versteeg 2023-02-03 10h30m08s 94.780s(Chemiluminescence).tif]

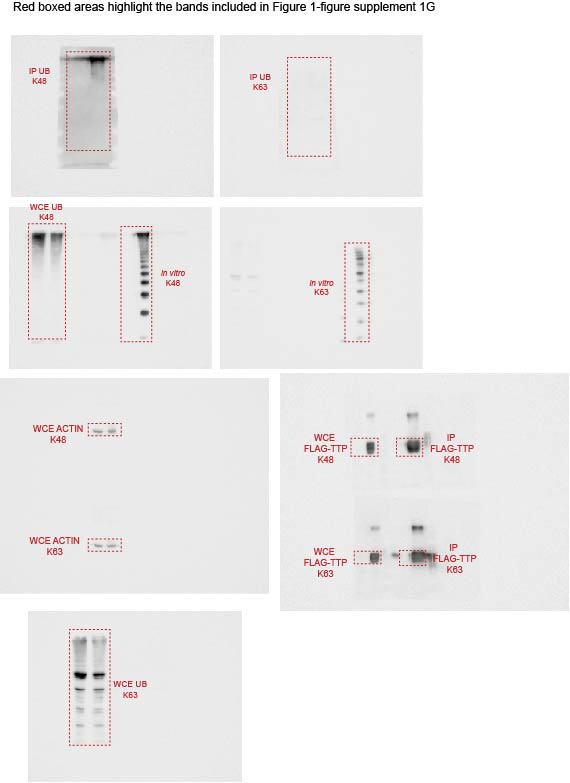

Supplement: Figure 1—figure supplement 1—source data 6. [file elife-83159-fig1-figsupp1-data6.zip › Figure 1-figure supplement 1-source data 6.jpg]

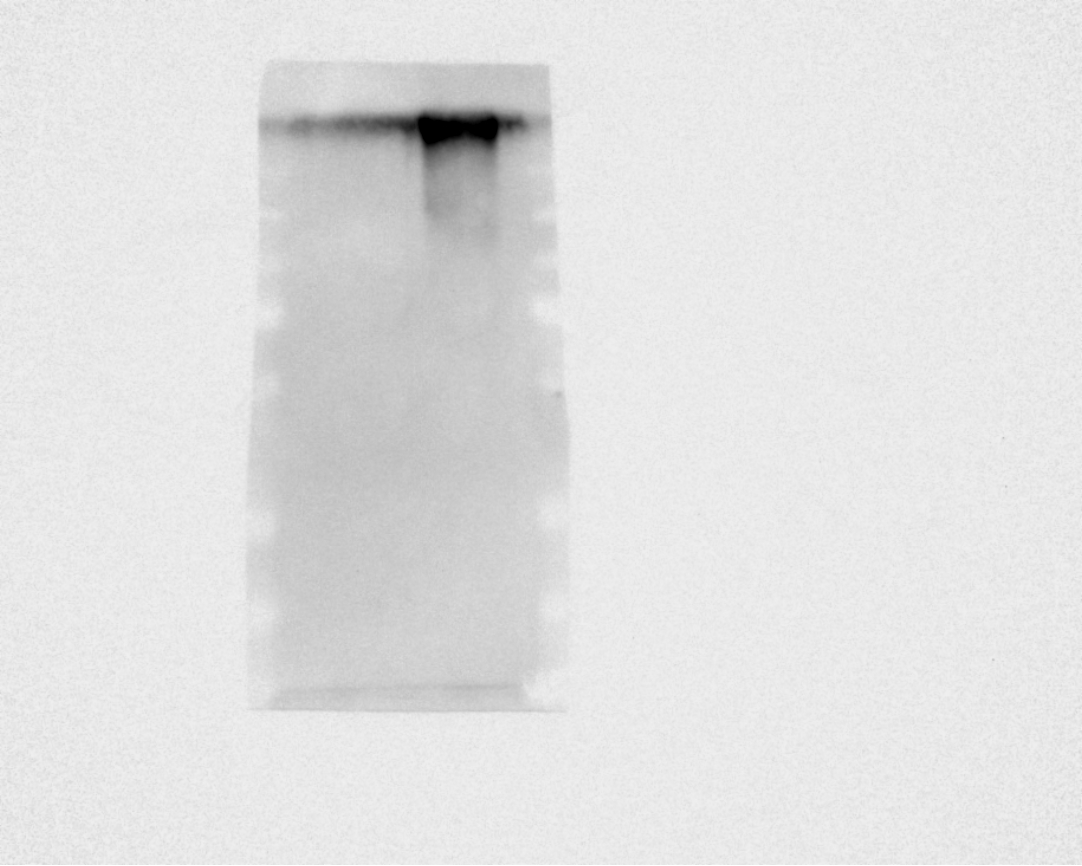

Supplement: Figure 1—figure supplement 1—source data 6. [file elife-83159-fig1-figsupp1-data6.zip › IP K48 Figure 1-figure supplement 1-source data 6/Versteeg 2023-02-01 10h18m59s 155.650s(Chemiluminescence).jpg]

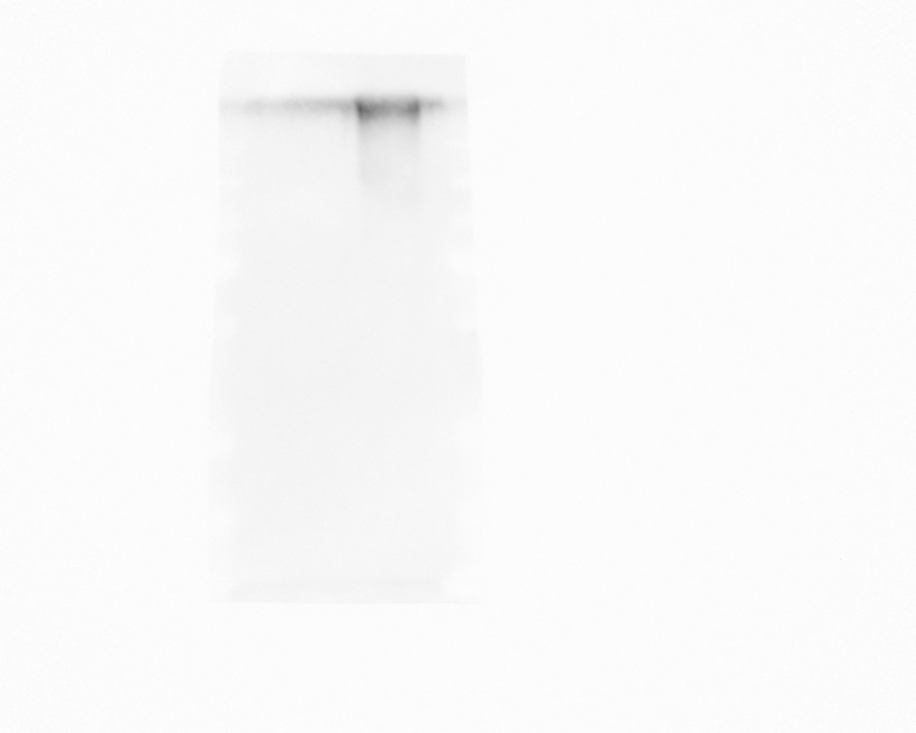

Supplement: Figure 1—figure supplement 1—source data 6. [file elife-83159-fig1-figsupp1-data6.zip › IP K48 Figure 1-figure supplement 1-source data 6/Versteeg 2023-02-01 10h18m59s 155.650s(Chemiluminescence).raw16.tif]

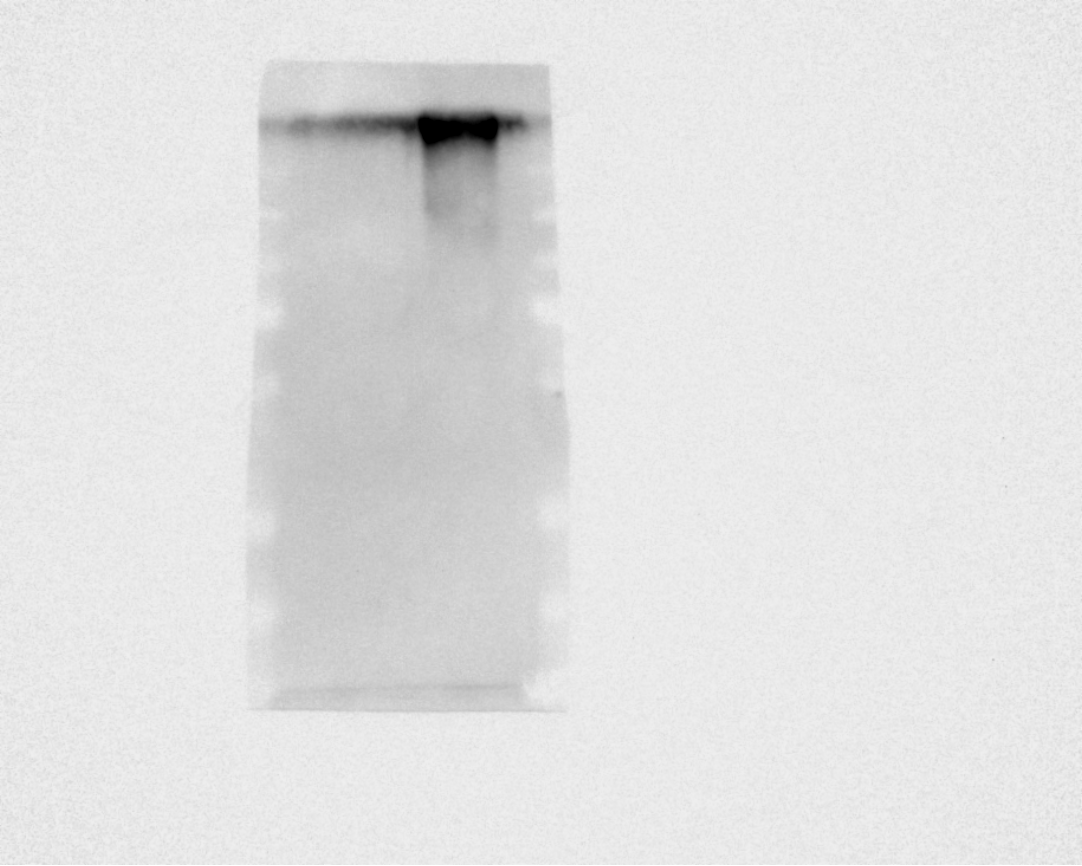

Supplement: Figure 1—figure supplement 1—source data 6. [file elife-83159-fig1-figsupp1-data6.zip › IP K48 Figure 1-figure supplement 1-source data 6/Versteeg 2023-02-01 10h18m59s 155.650s(Chemiluminescence).tif]

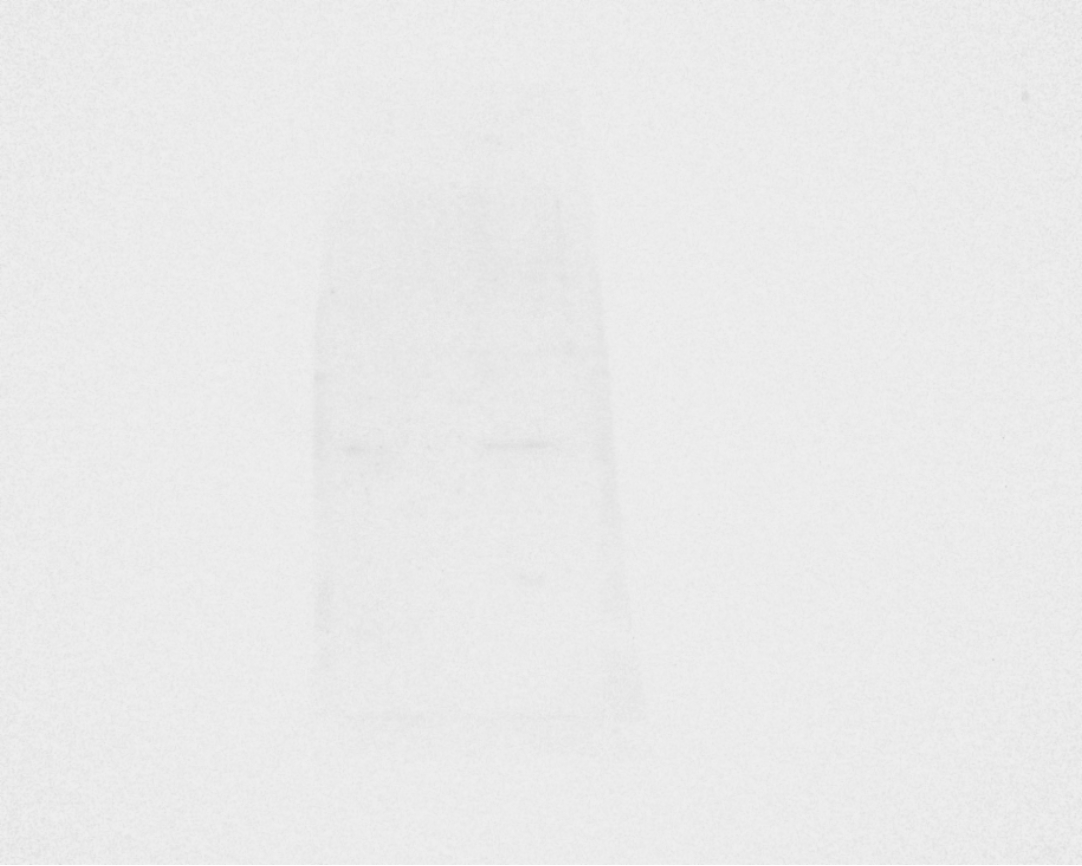

Supplement: Figure 1—figure supplement 1—source data 6. [file elife-83159-fig1-figsupp1-data6.zip › IP K63 Figure 1-figure supplement 1-source data 6/Versteeg 2023-02-01 10h45m15s 82.840s(Chemiluminescence).jpg]

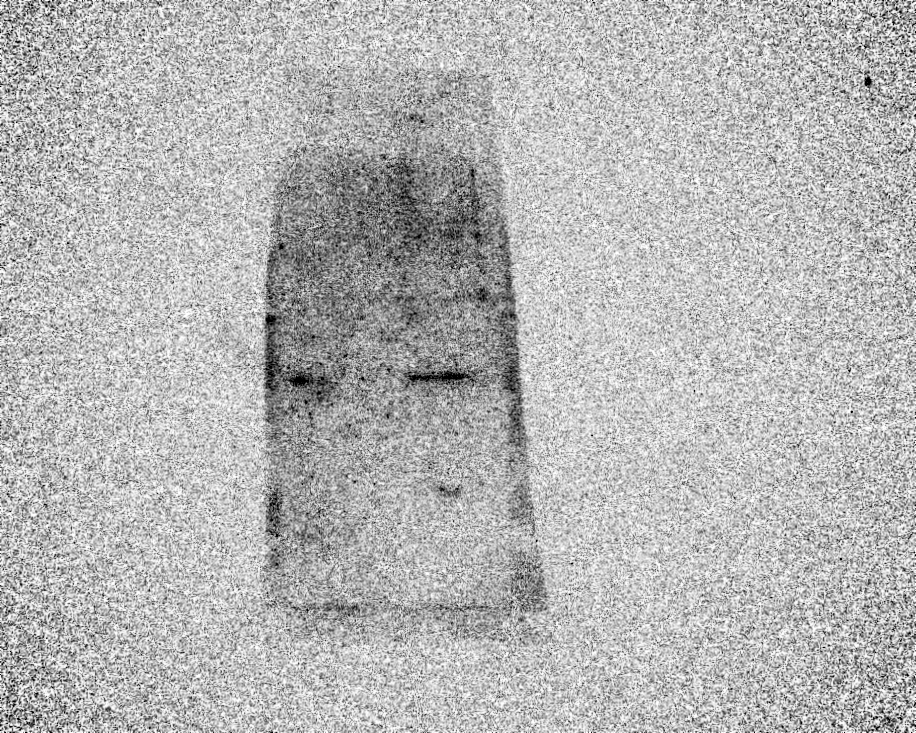

Supplement: Figure 1—figure supplement 1—source data 6. [file elife-83159-fig1-figsupp1-data6.zip › IP K63 Figure 1-figure supplement 1-source data 6/Versteeg 2023-02-01 10h45m15s 82.840s(Chemiluminescence).raw16.tif]

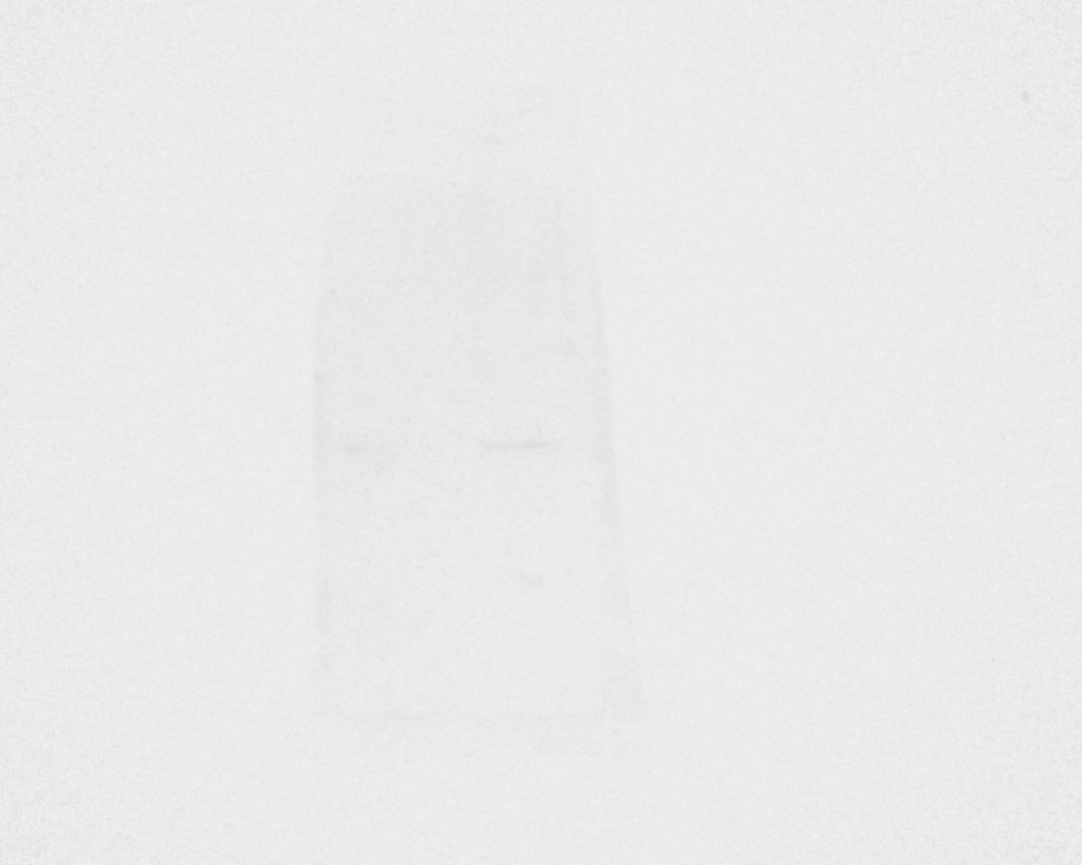

Supplement: Figure 1—figure supplement 1—source data 6. [file elife-83159-fig1-figsupp1-data6.zip › IP K63 Figure 1-figure supplement 1-source data 6/Versteeg 2023-02-01 10h45m15s 82.840s(Chemiluminescence).tif]

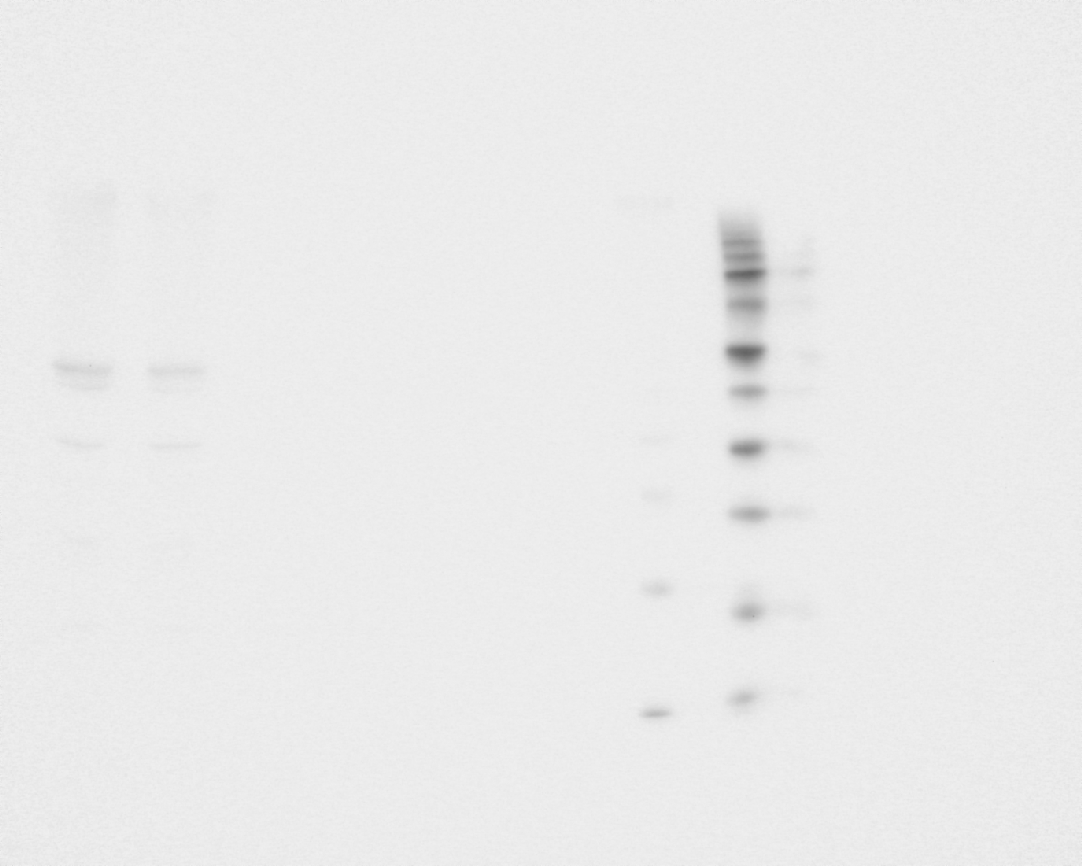

Supplement: Figure 1—figure supplement 1—source data 6. [file elife-83159-fig1-figsupp1-data6.zip › K63 in vitro Figure 1-figure supplement 1-source data 6/Versteeg 2023-02-01 10h25m52s 62.860s(Chemiluminescence).jpg]

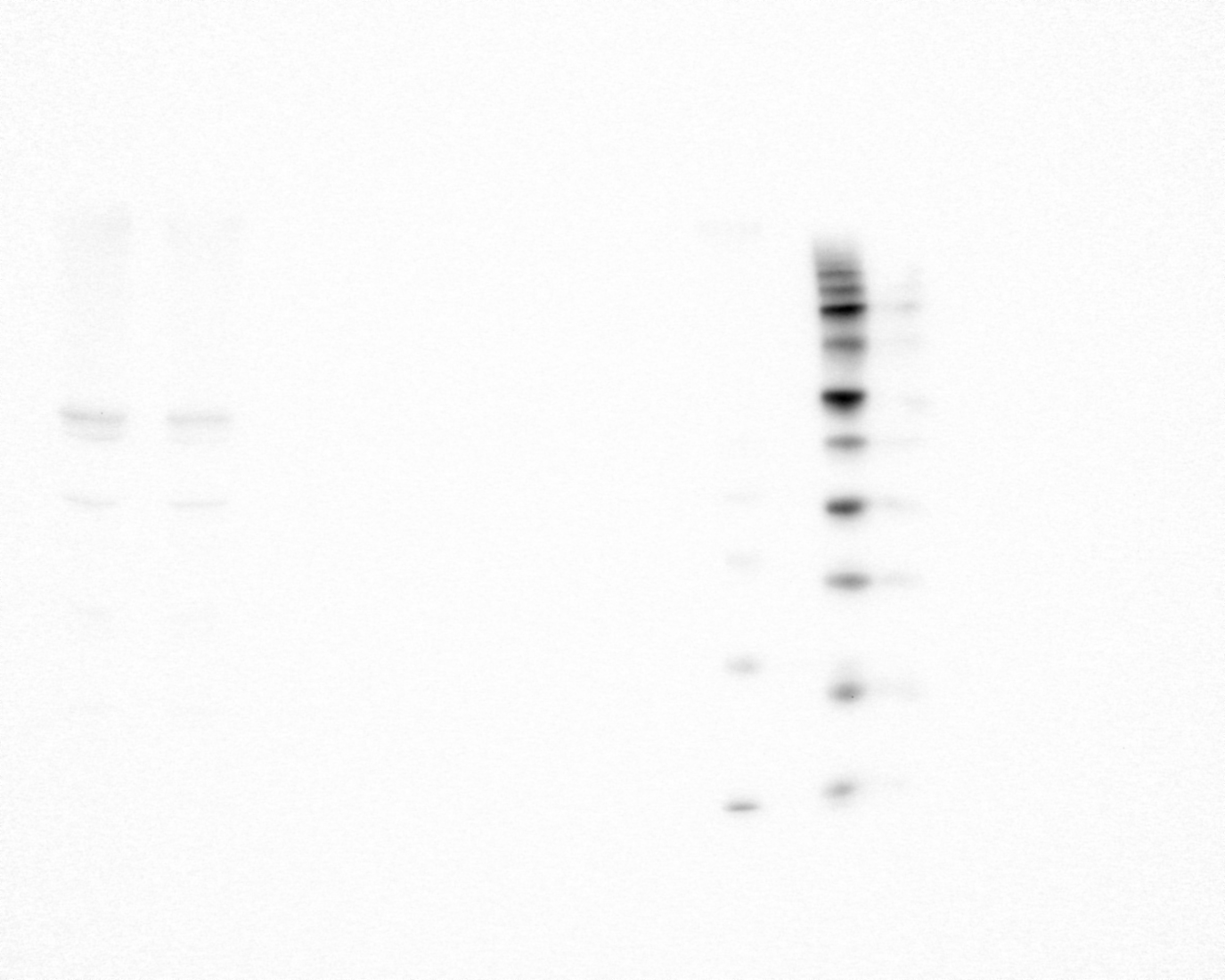

Supplement: Figure 1—figure supplement 1—source data 6. [file elife-83159-fig1-figsupp1-data6.zip › K63 in vitro Figure 1-figure supplement 1-source data 6/Versteeg 2023-02-01 10h25m52s 62.860s(Chemiluminescence).raw16.tif]

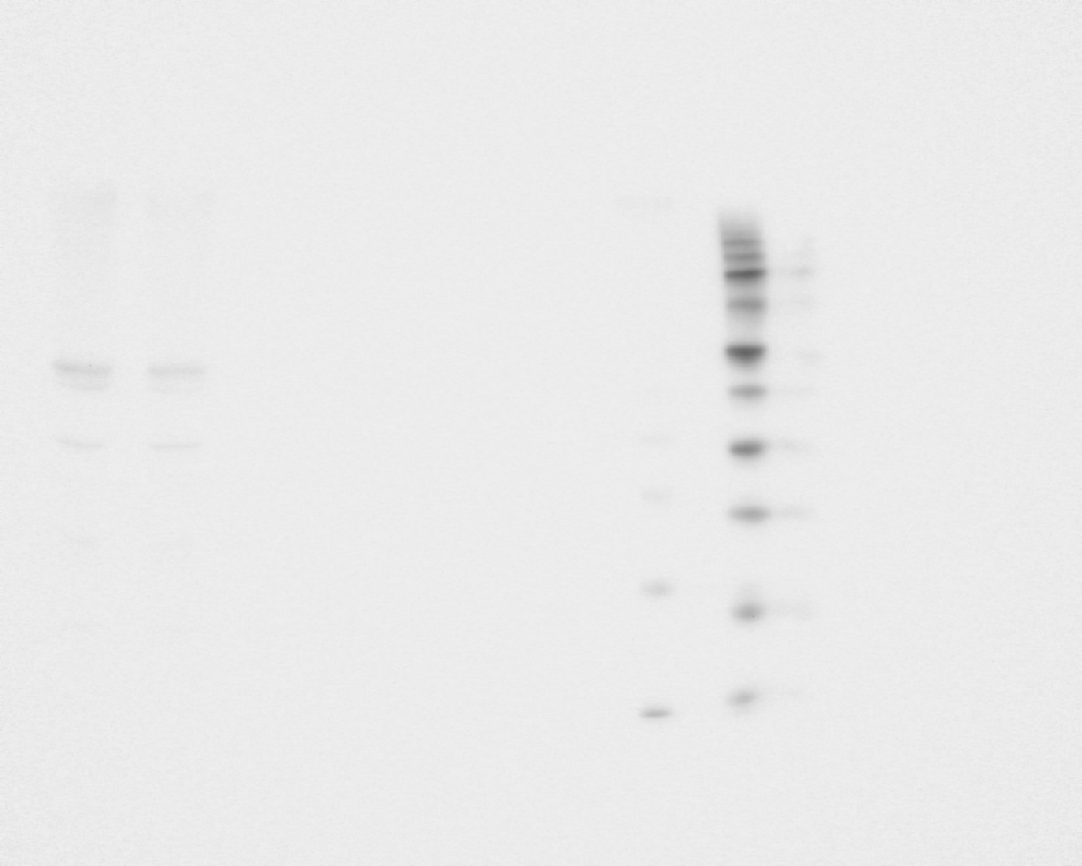

Supplement: Figure 1—figure supplement 1—source data 6. [file elife-83159-fig1-figsupp1-data6.zip › K63 in vitro Figure 1-figure supplement 1-source data 6/Versteeg 2023-02-01 10h25m52s 62.860s(Chemiluminescence).tif]

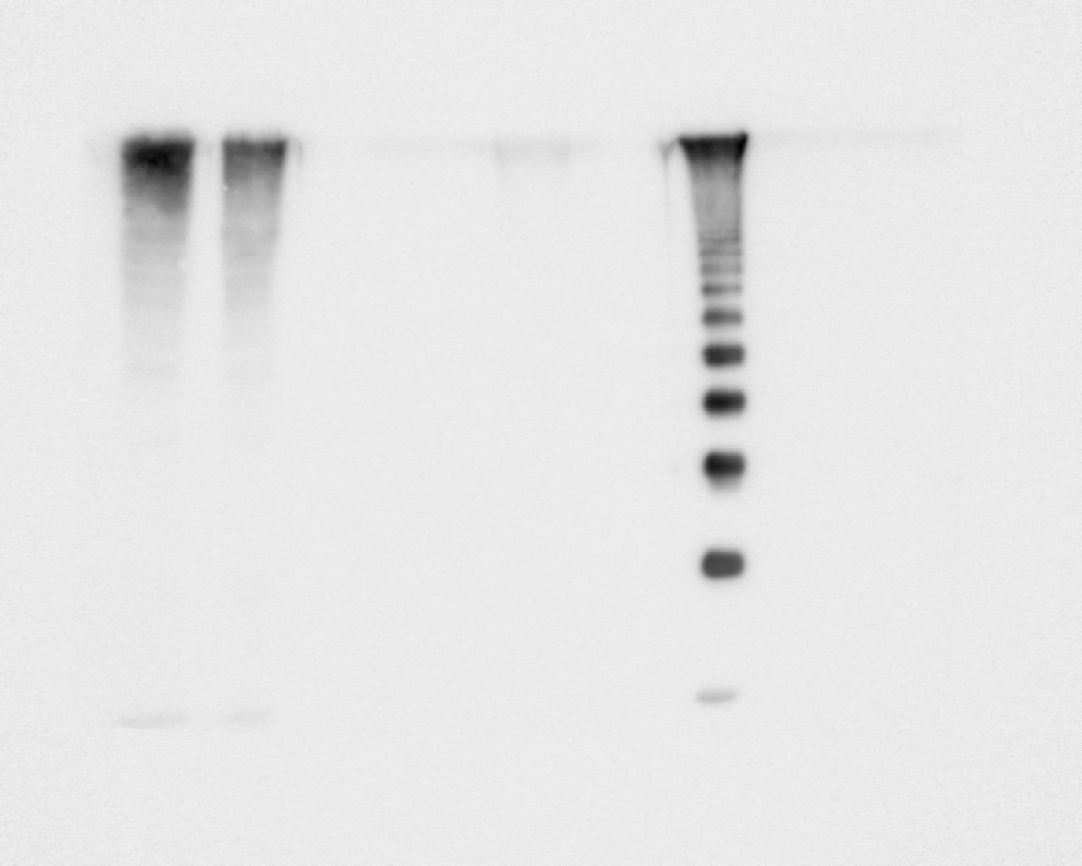

Supplement: Figure 1—figure supplement 1—source data 6. [file elife-83159-fig1-figsupp1-data6.zip › WCE K48 and K48 in vitro Figure 1-figure supplement 1-source data 6/Versteeg 2023-02-01 10h06m31s 83.480s(Chemiluminescence).jpg]

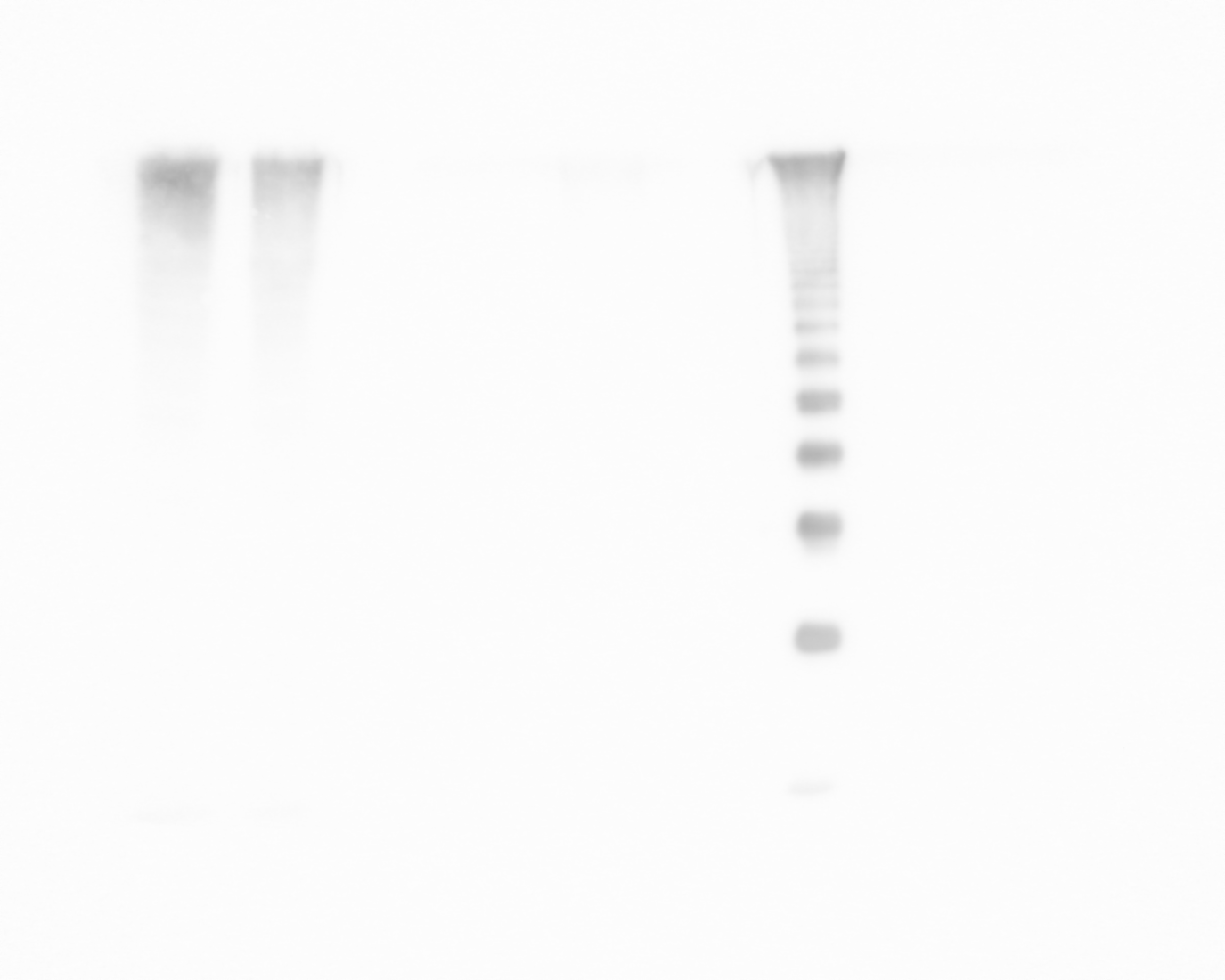

Supplement: Figure 1—figure supplement 1—source data 6. [file elife-83159-fig1-figsupp1-data6.zip › WCE K48 and K48 in vitro Figure 1-figure supplement 1-source data 6/Versteeg 2023-02-01 10h06m31s 83.480s(Chemiluminescence).raw16.tif]

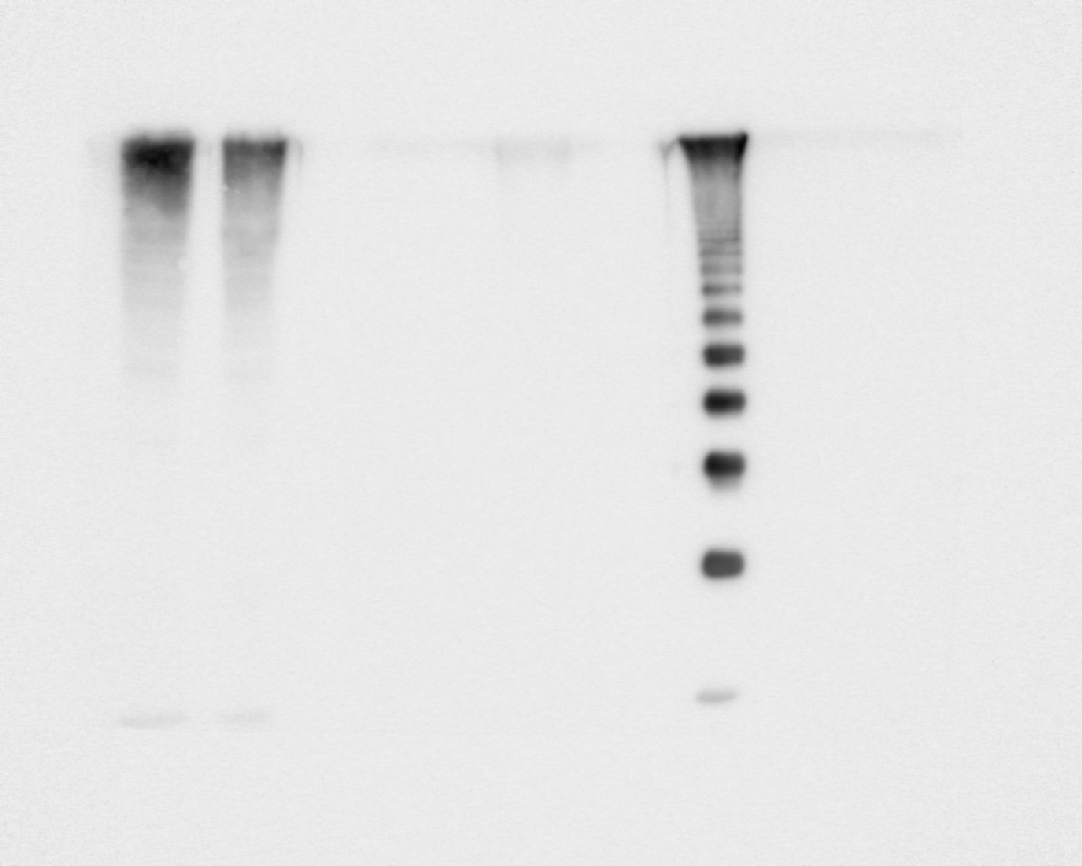

Supplement: Figure 1—figure supplement 1—source data 6. [file elife-83159-fig1-figsupp1-data6.zip › WCE K48 and K48 in vitro Figure 1-figure supplement 1-source data 6/Versteeg 2023-02-01 10h06m31s 83.480s(Chemiluminescence).tif]

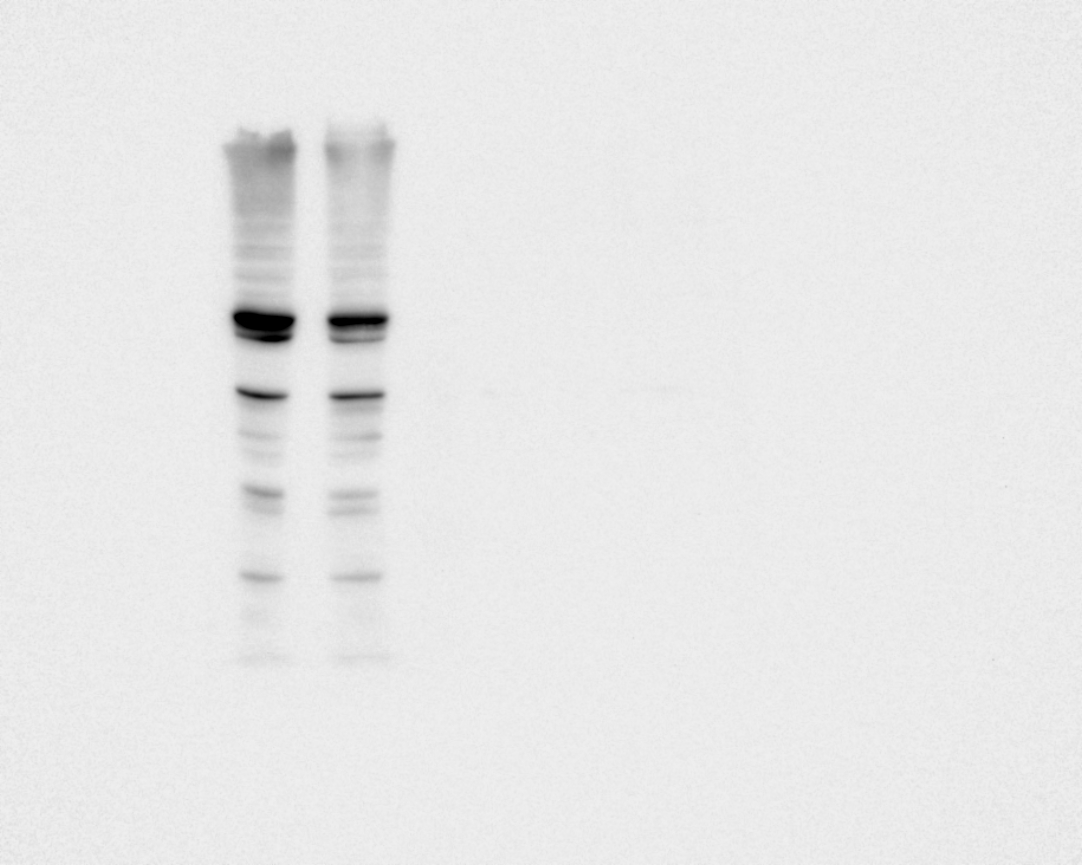

Supplement: Figure 1—figure supplement 1—source data 6. [file elife-83159-fig1-figsupp1-data6.zip › WCE K63 Figure 1-figure supplement 1-source data 6/Versteeg 2023-02-01 10h39m15s 93.790s(Chemiluminescence).jpg]

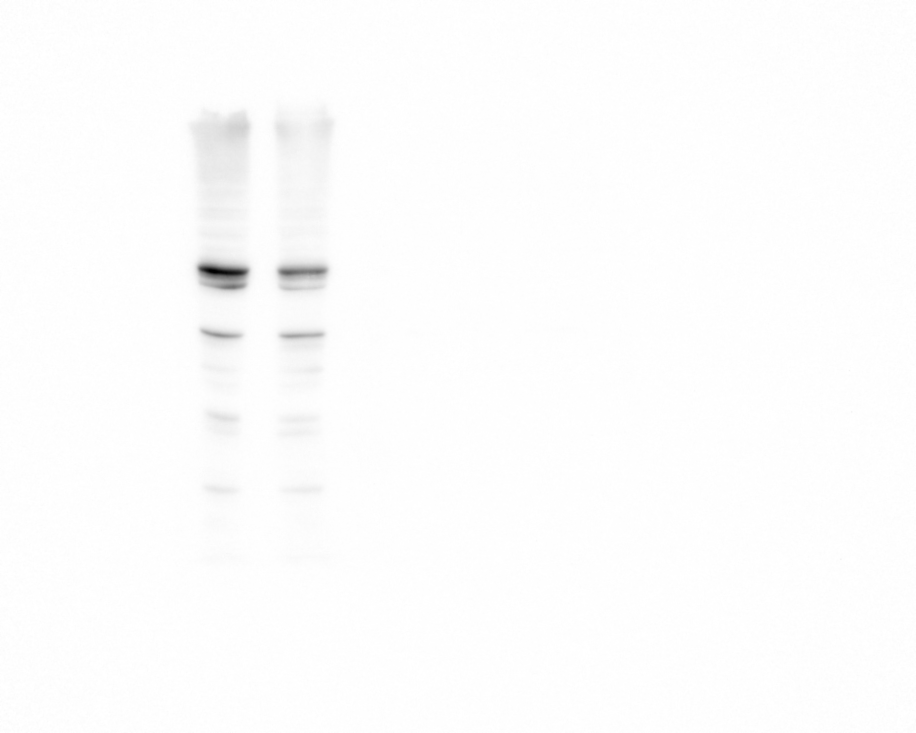

Supplement: Figure 1—figure supplement 1—source data 6. [file elife-83159-fig1-figsupp1-data6.zip › WCE K63 Figure 1-figure supplement 1-source data 6/Versteeg 2023-02-01 10h39m15s 93.790s(Chemiluminescence).raw16.tif]

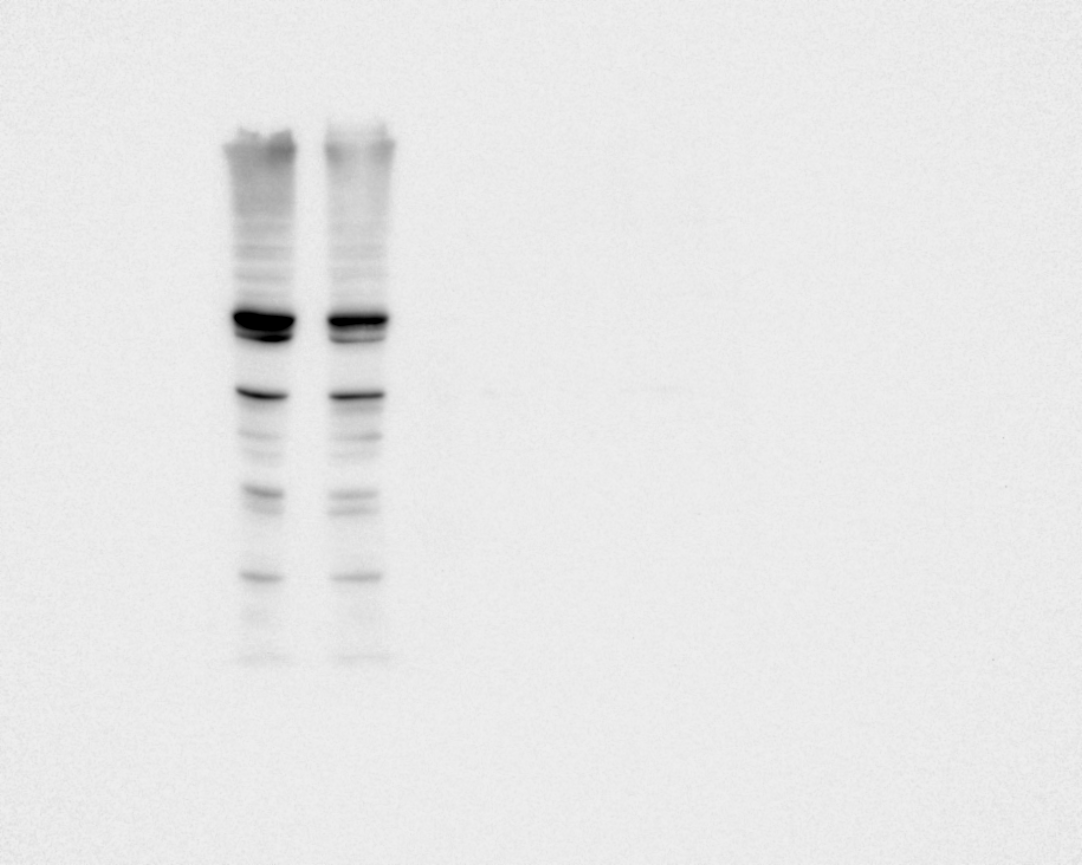

Supplement: Figure 1—figure supplement 1—source data 6. [file elife-83159-fig1-figsupp1-data6.zip › WCE K63 Figure 1-figure supplement 1-source data 6/Versteeg 2023-02-01 10h39m15s 93.790s(Chemiluminescence).tif]

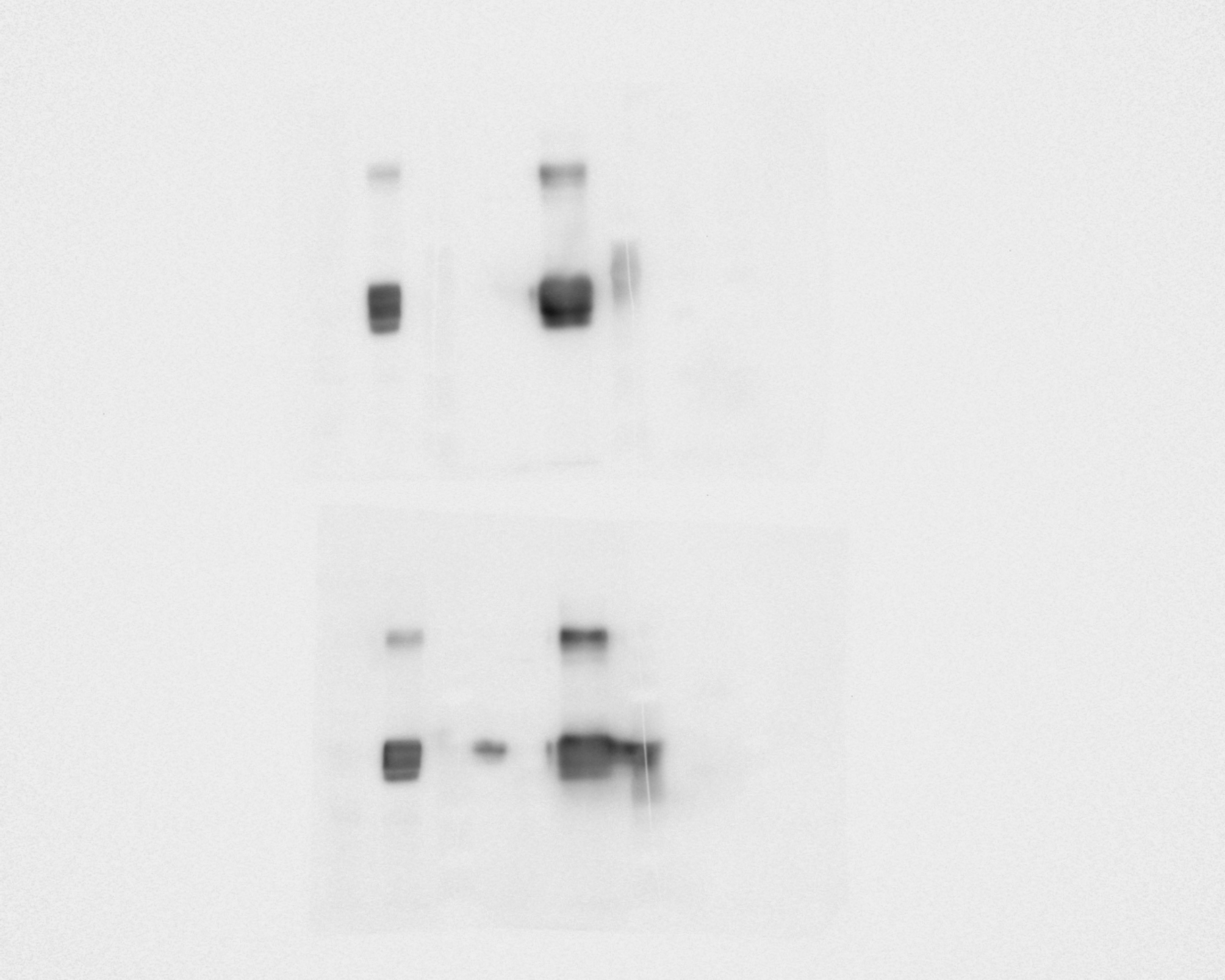

Supplement: Figure 1—figure supplement 1—source data 6. [file elife-83159-fig1-figsupp1-data6.zip › WCE+IP TTP K48 and K63 Figure 1-figure supplement 1-source data 6/Versteeg 2023-02-02 15h53m41s 37.470s(Chemiluminescence).jpg]

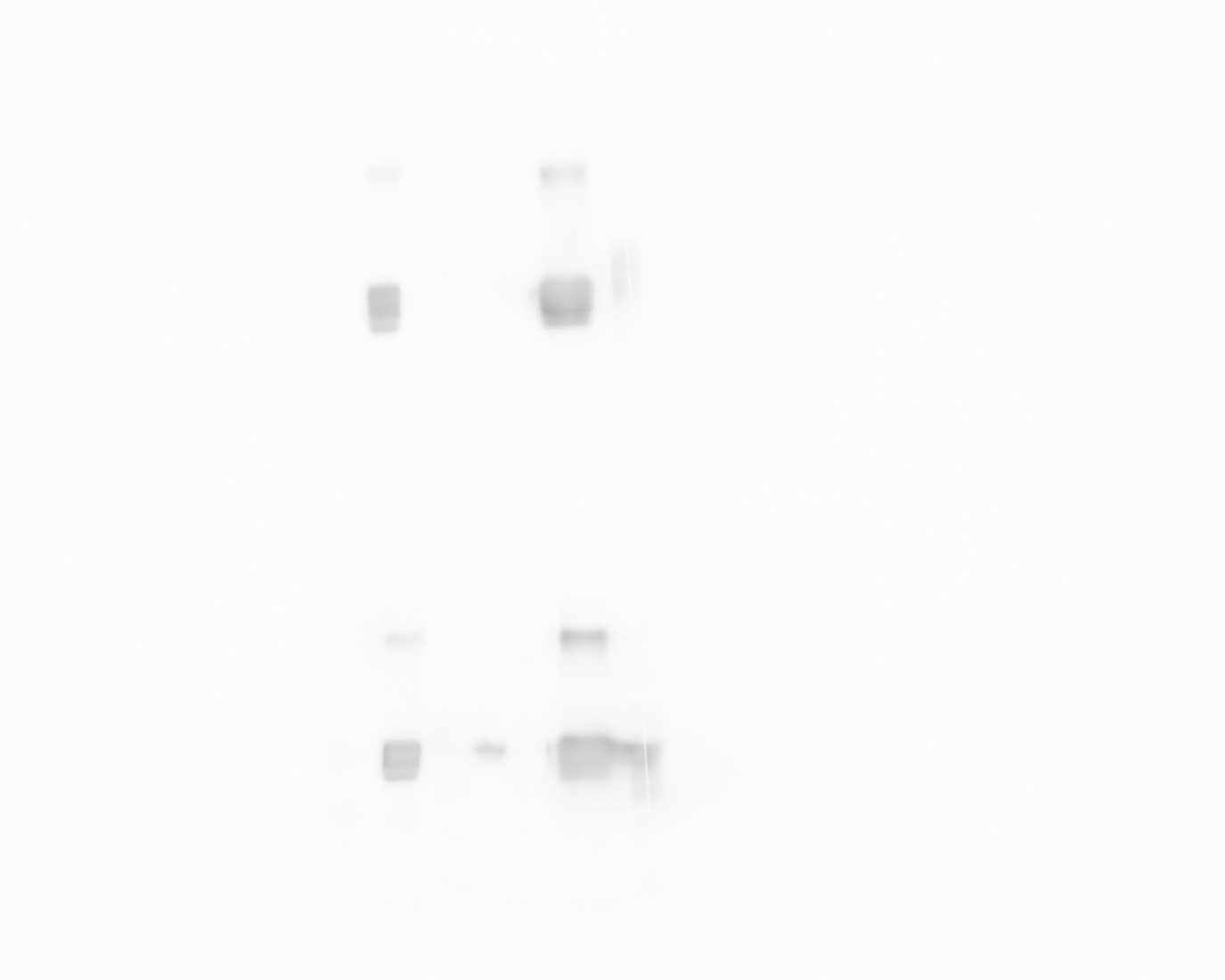

Supplement: Figure 1—figure supplement 1—source data 6. [file elife-83159-fig1-figsupp1-data6.zip › WCE+IP TTP K48 and K63 Figure 1-figure supplement 1-source data 6/Versteeg 2023-02-02 15h53m41s 37.470s(Chemiluminescence).raw16.tif]

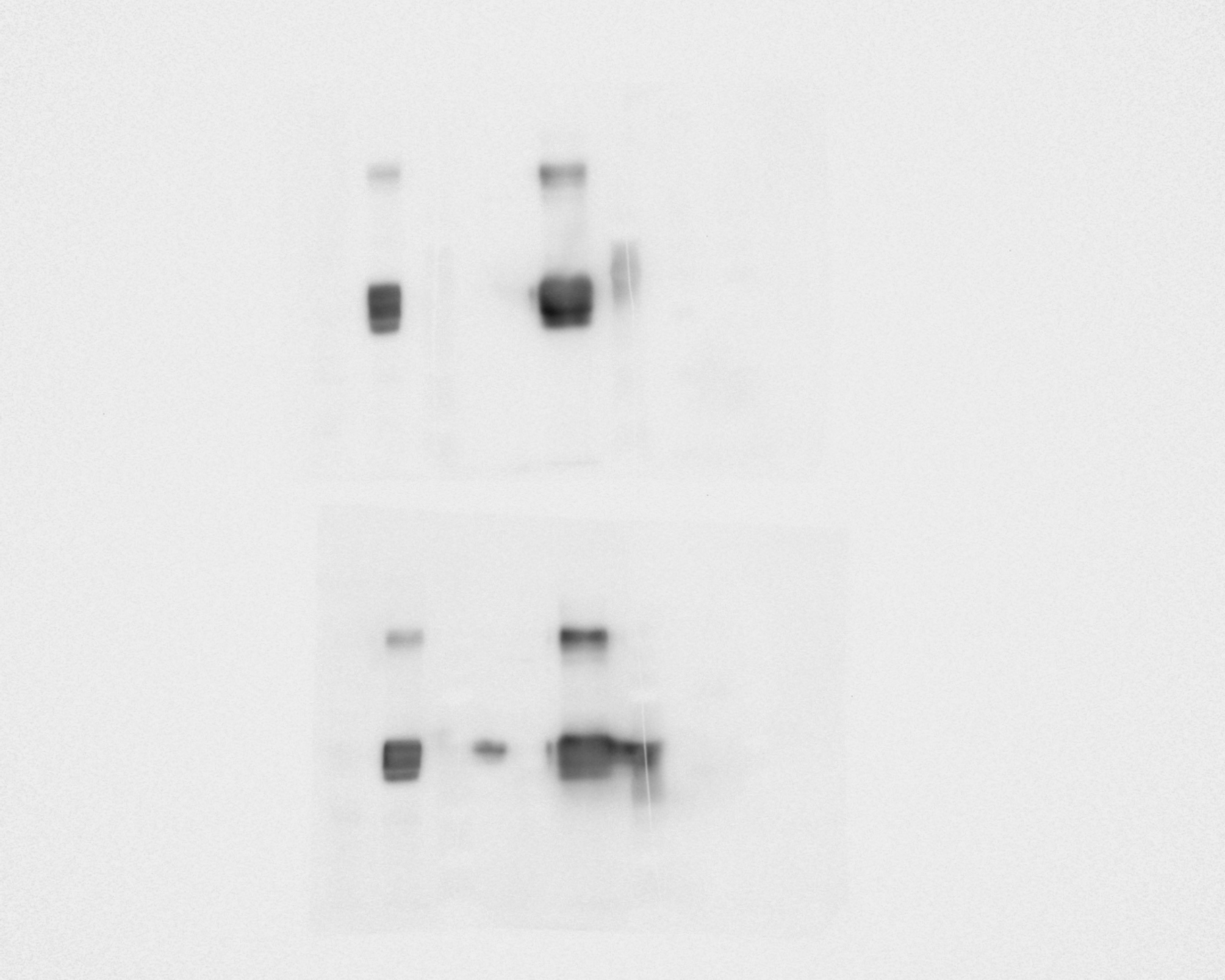

Supplement: Figure 1—figure supplement 1—source data 6. [file elife-83159-fig1-figsupp1-data6.zip › WCE+IP TTP K48 and K63 Figure 1-figure supplement 1-source data 6/Versteeg 2023-02-02 15h53m41s 37.470s(Chemiluminescence).tif]

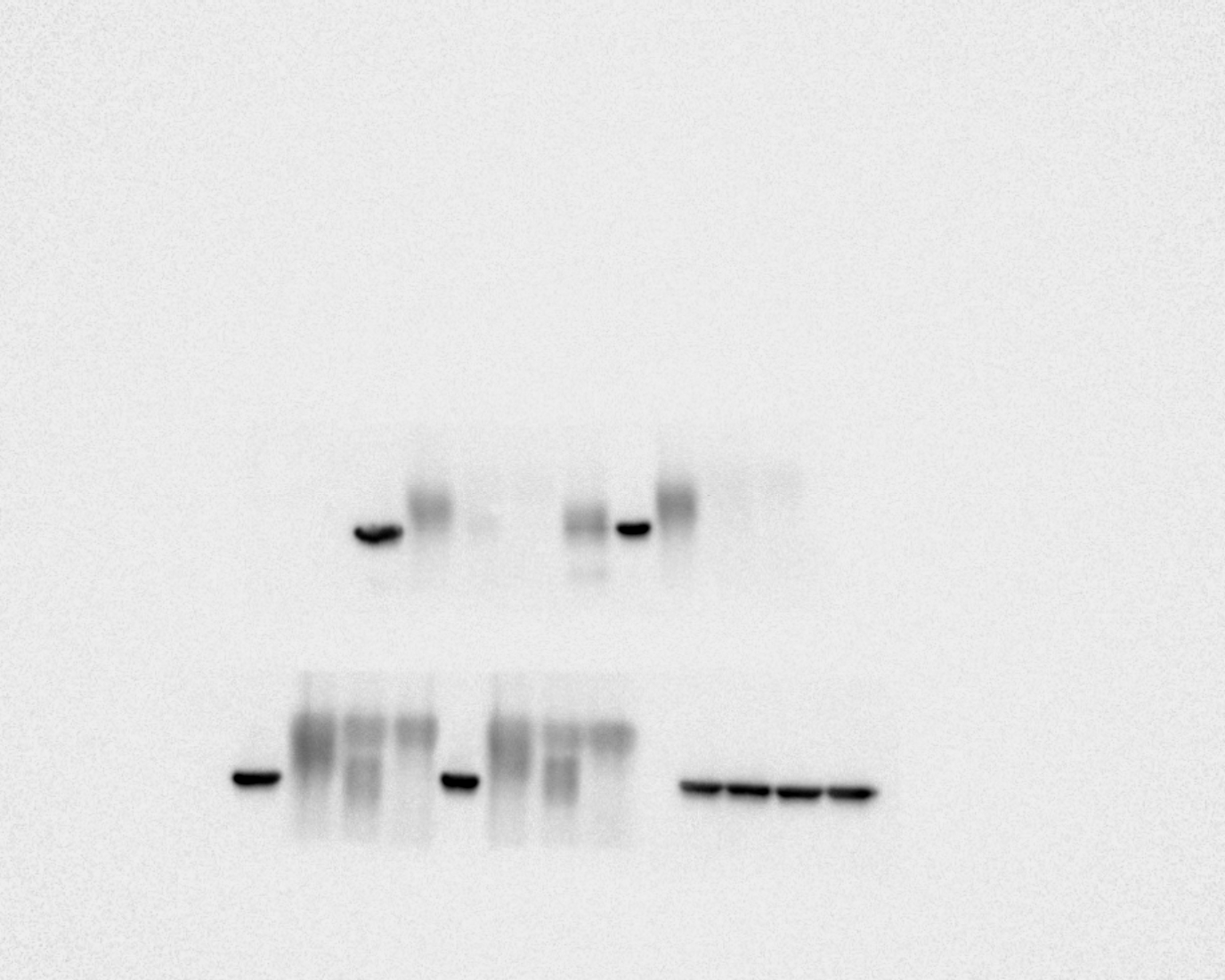

Supplement: Figure 1—figure supplement 1—source data 7. [file elife-83159-fig1-figsupp1-data7.zip › ACTIN Figure 1-figure supplement 1-source data 7/Versteeg 2021-08-02 11h16m41s 15.000s(Chemiluminescence).jpg]

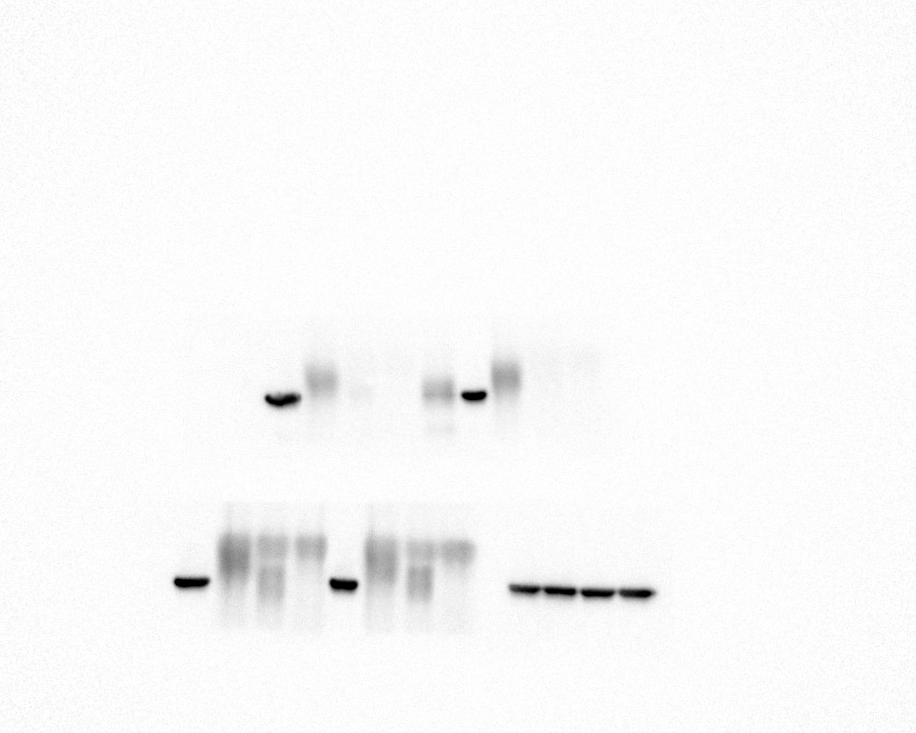

Supplement: Figure 1—figure supplement 1—source data 7. [file elife-83159-fig1-figsupp1-data7.zip › ACTIN Figure 1-figure supplement 1-source data 7/Versteeg 2021-08-02 11h16m41s 15.000s(Chemiluminescence).raw16.tif]

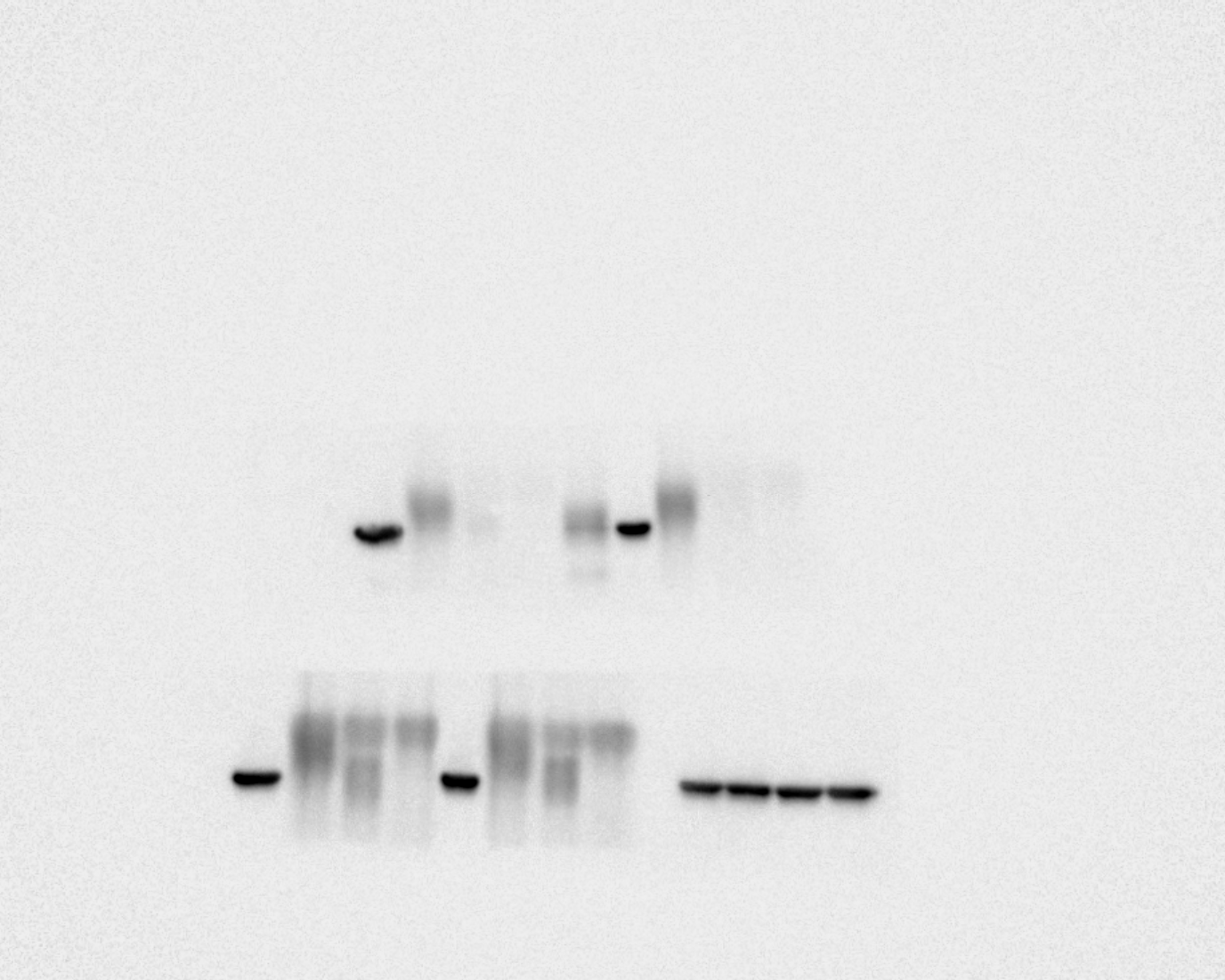

Supplement: Figure 1—figure supplement 1—source data 7. [file elife-83159-fig1-figsupp1-data7.zip › ACTIN Figure 1-figure supplement 1-source data 7/Versteeg 2021-08-02 11h16m41s 15.000s(Chemiluminescence).tif]

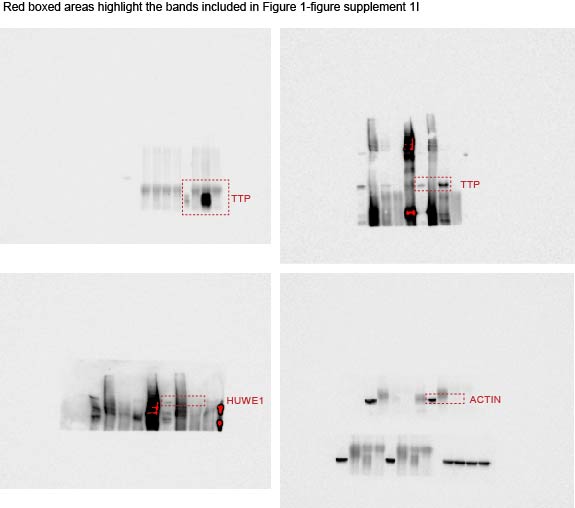

Supplement: Figure 1—figure supplement 1—source data 7. [file elife-83159-fig1-figsupp1-data7.zip › Figure 1-figure supplement 1-source data 7.jpg]

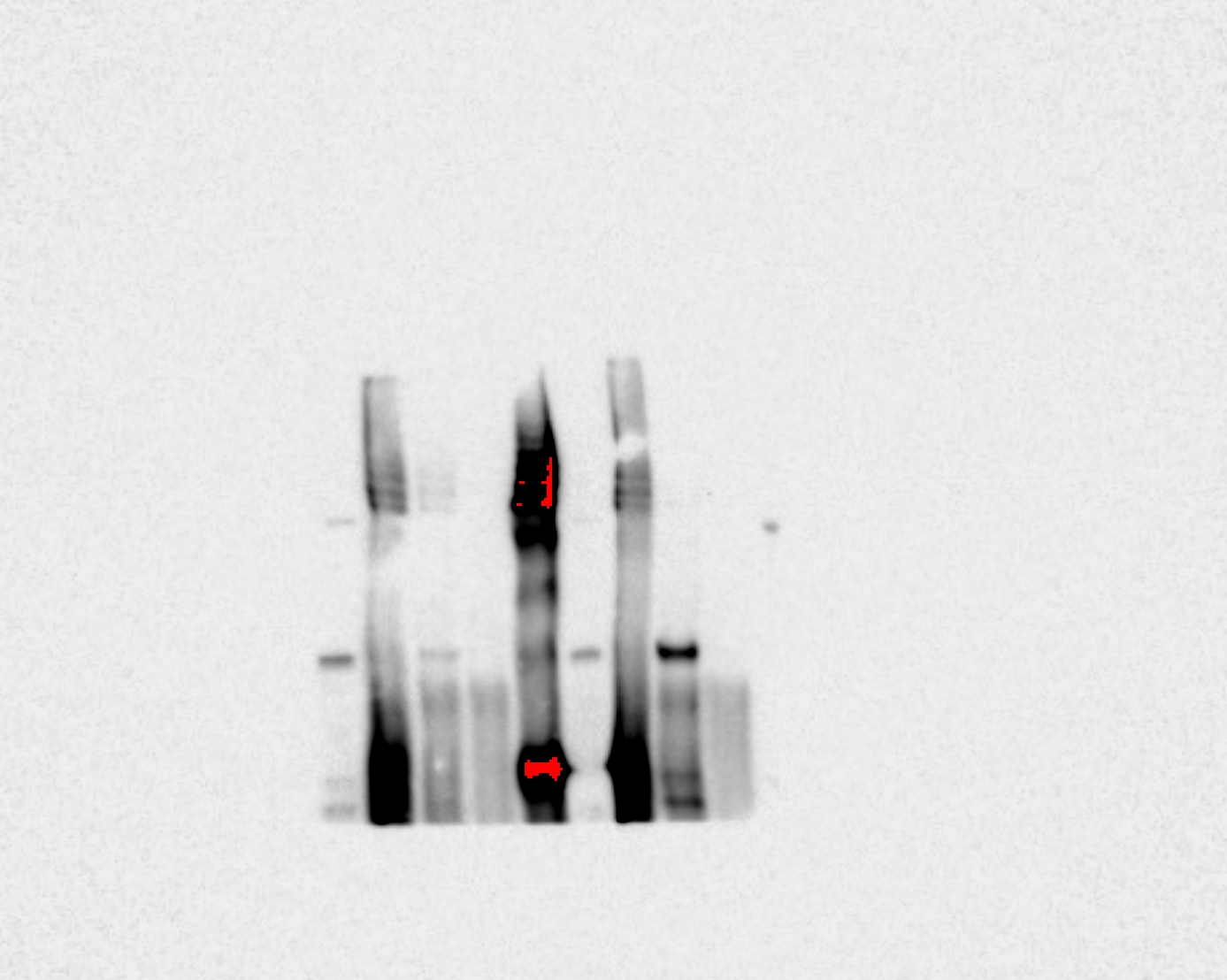

Supplement: Figure 1—figure supplement 1—source data 7. [file elife-83159-fig1-figsupp1-data7.zip › GIGYF1 Figure 1-figure supplement 1-source data 7/Versteeg 2021-07-27 14h02m14s 12.000s(Chemiluminescence).jpg]

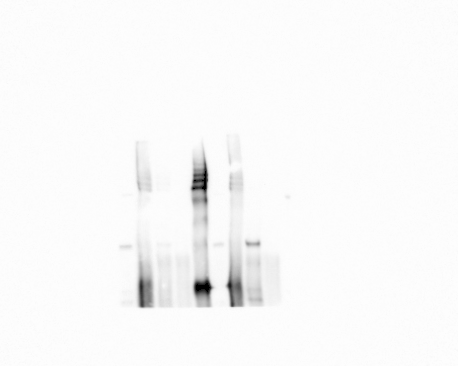

Supplement: Figure 1—figure supplement 1—source data 7. [file elife-83159-fig1-figsupp1-data7.zip › GIGYF1 Figure 1-figure supplement 1-source data 7/Versteeg 2021-07-27 14h02m14s 12.000s(Chemiluminescence).raw16.tif]

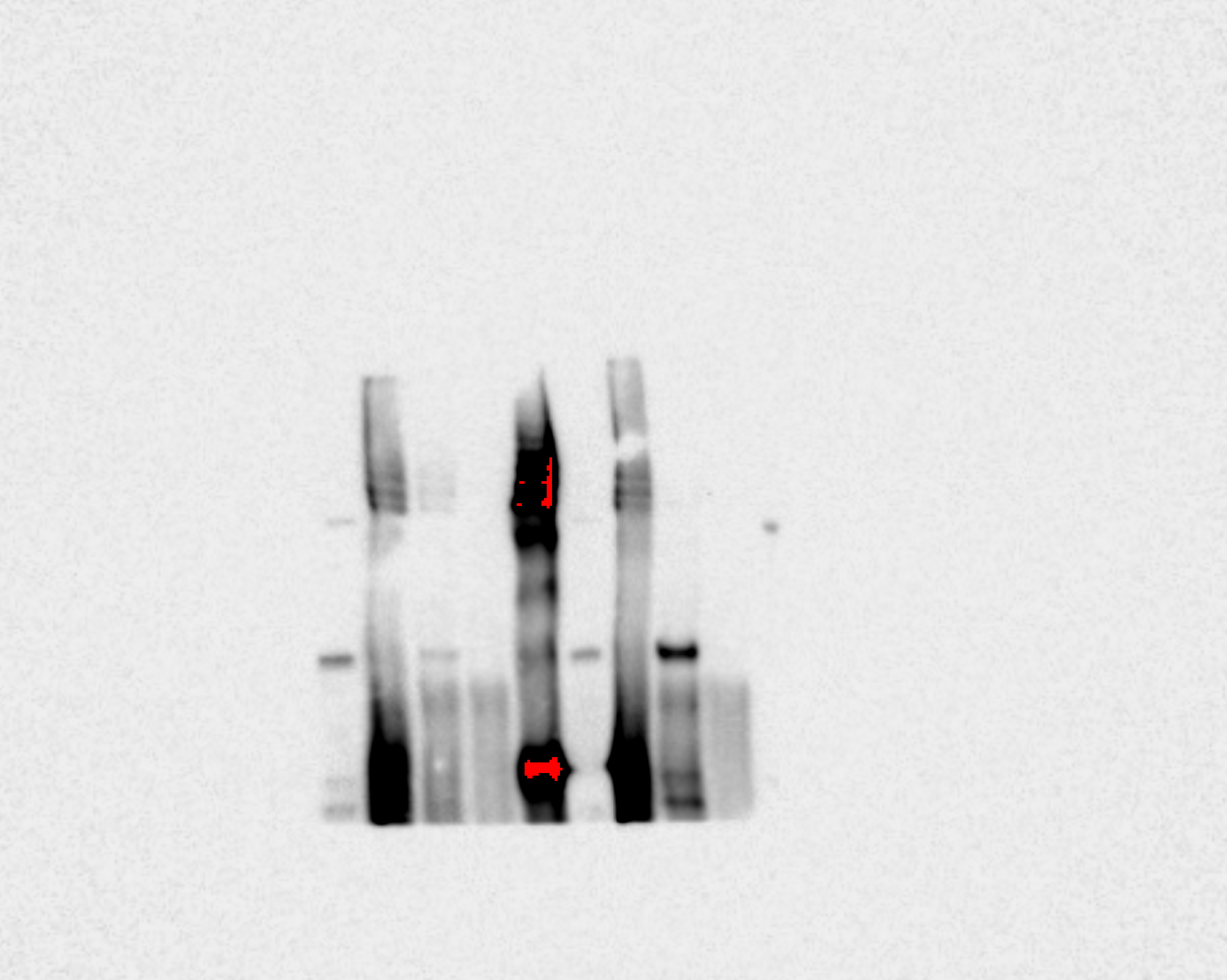

Supplement: Figure 1—figure supplement 1—source data 7. [file elife-83159-fig1-figsupp1-data7.zip › GIGYF1 Figure 1-figure supplement 1-source data 7/Versteeg 2021-07-27 14h02m14s 12.000s(Chemiluminescence).tif]

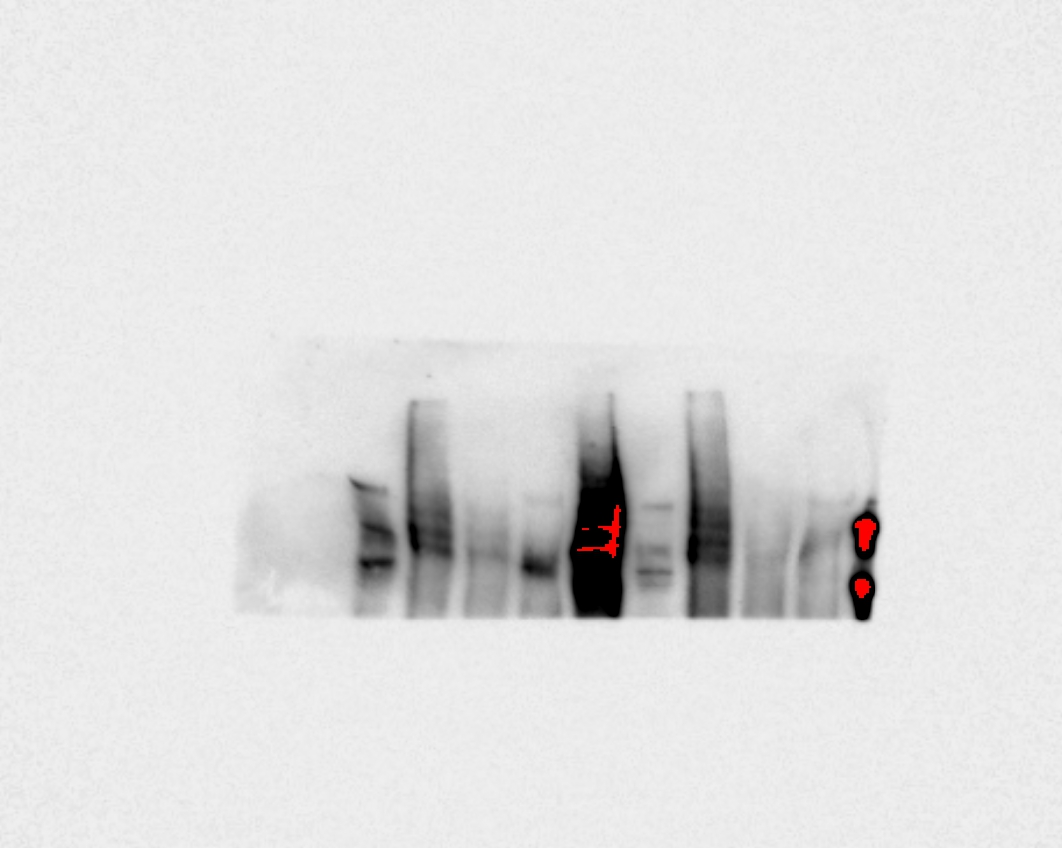

Supplement: Figure 1—figure supplement 1—source data 7. [file elife-83159-fig1-figsupp1-data7.zip › HUWE1 Figure 1-figure supplement 1-source data 7/Versteeg 2021-07-28 14h48m00s 13.204s(Chemiluminescence).jpg]

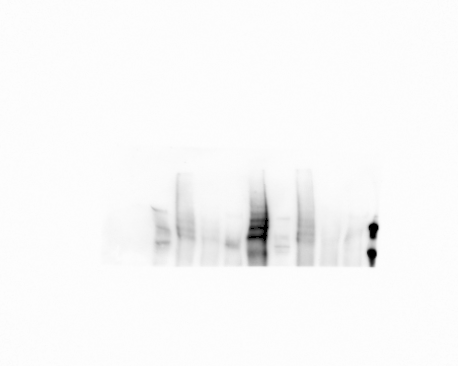

Supplement: Figure 1—figure supplement 1—source data 7. [file elife-83159-fig1-figsupp1-data7.zip › HUWE1 Figure 1-figure supplement 1-source data 7/Versteeg 2021-07-28 14h48m00s 13.204s(Chemiluminescence).raw16.tif]

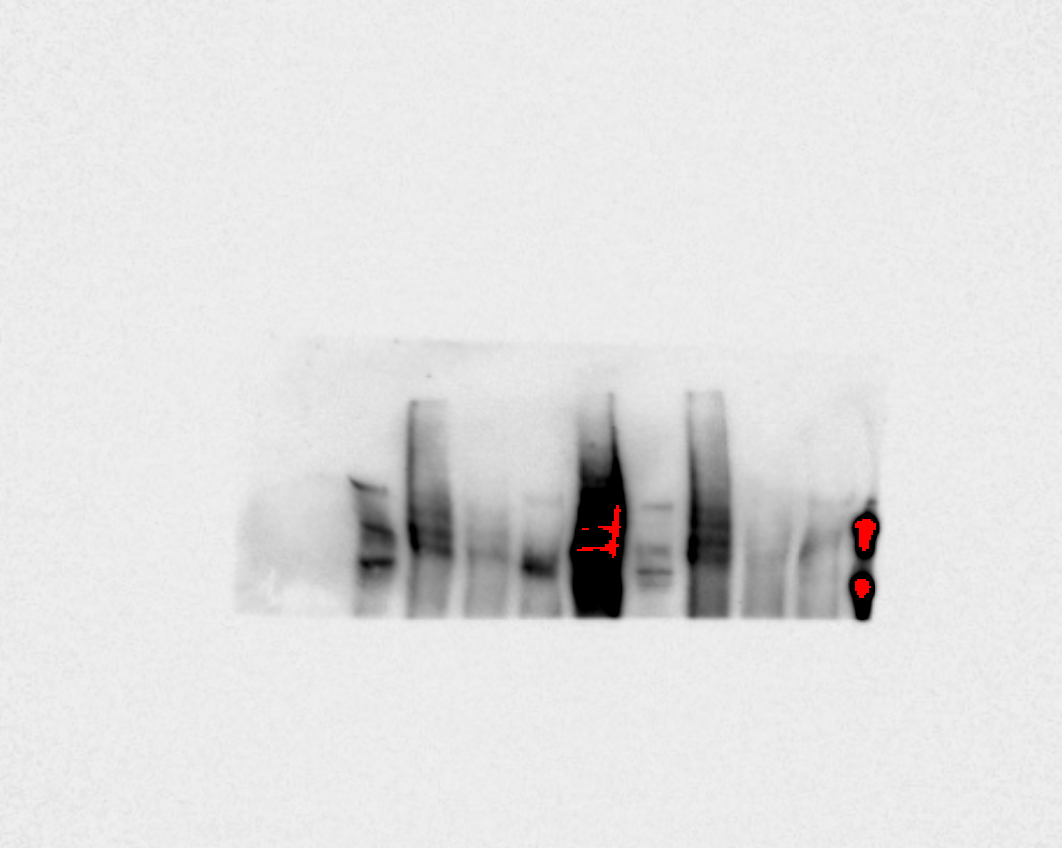

Supplement: Figure 1—figure supplement 1—source data 7. [file elife-83159-fig1-figsupp1-data7.zip › HUWE1 Figure 1-figure supplement 1-source data 7/Versteeg 2021-07-28 14h48m00s 13.204s(Chemiluminescence).tif]

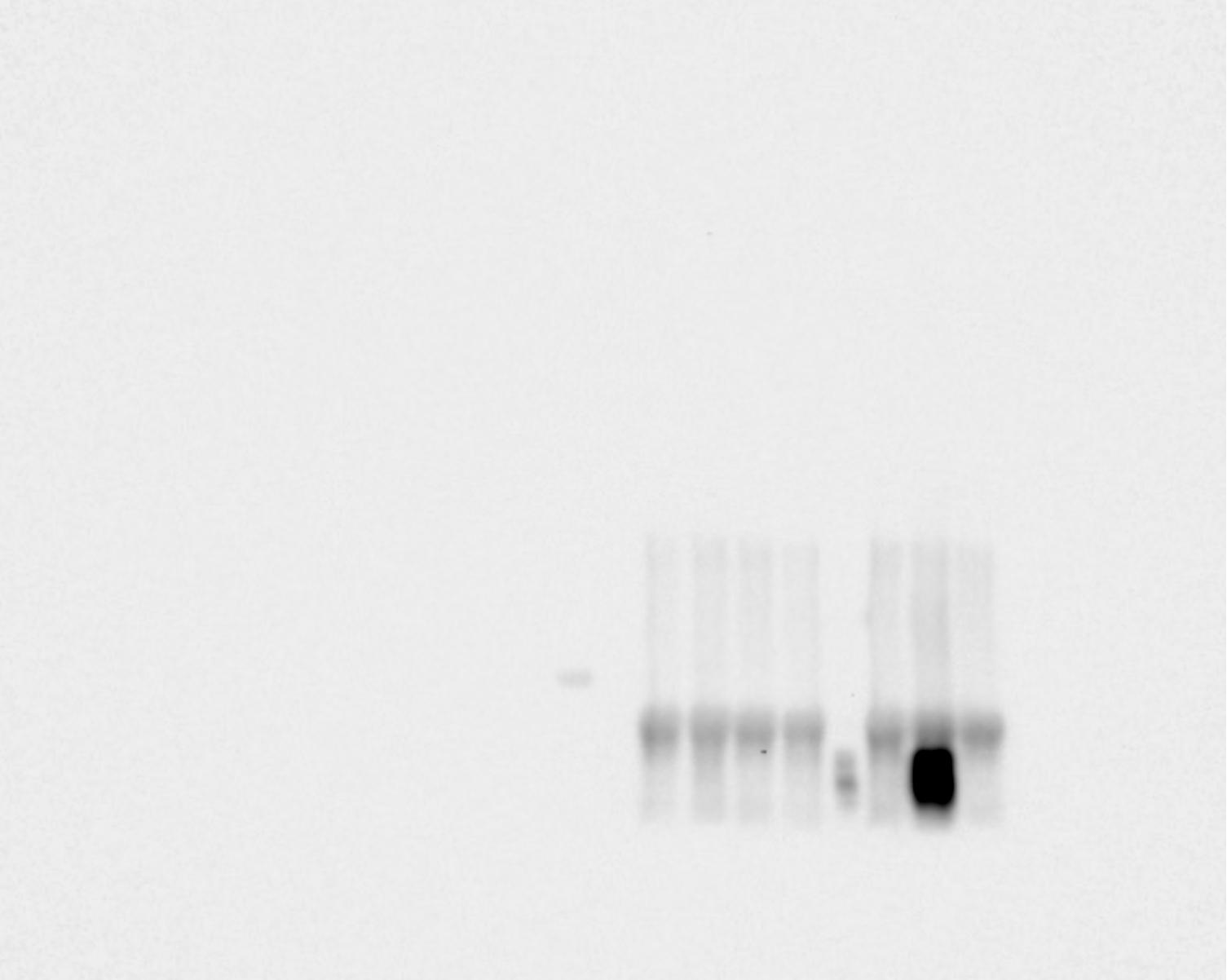

Supplement: Figure 1—figure supplement 1—source data 7. [file elife-83159-fig1-figsupp1-data7.zip › TTP Figure 1-figure supplement 1-source data 7/Versteeg 2021-07-25 17h16m25s 16.201s(Chemiluminescence).jpg]

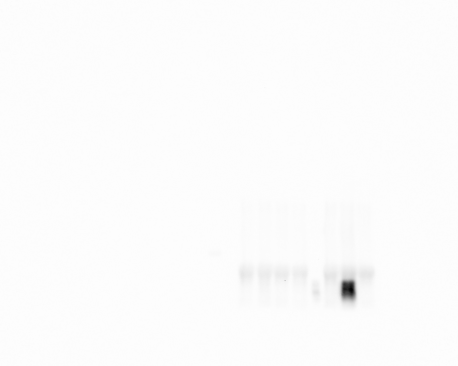

Supplement: Figure 1—figure supplement 1—source data 7. [file elife-83159-fig1-figsupp1-data7.zip › TTP Figure 1-figure supplement 1-source data 7/Versteeg 2021-07-25 17h16m25s 16.201s(Chemiluminescence).raw16.tif]

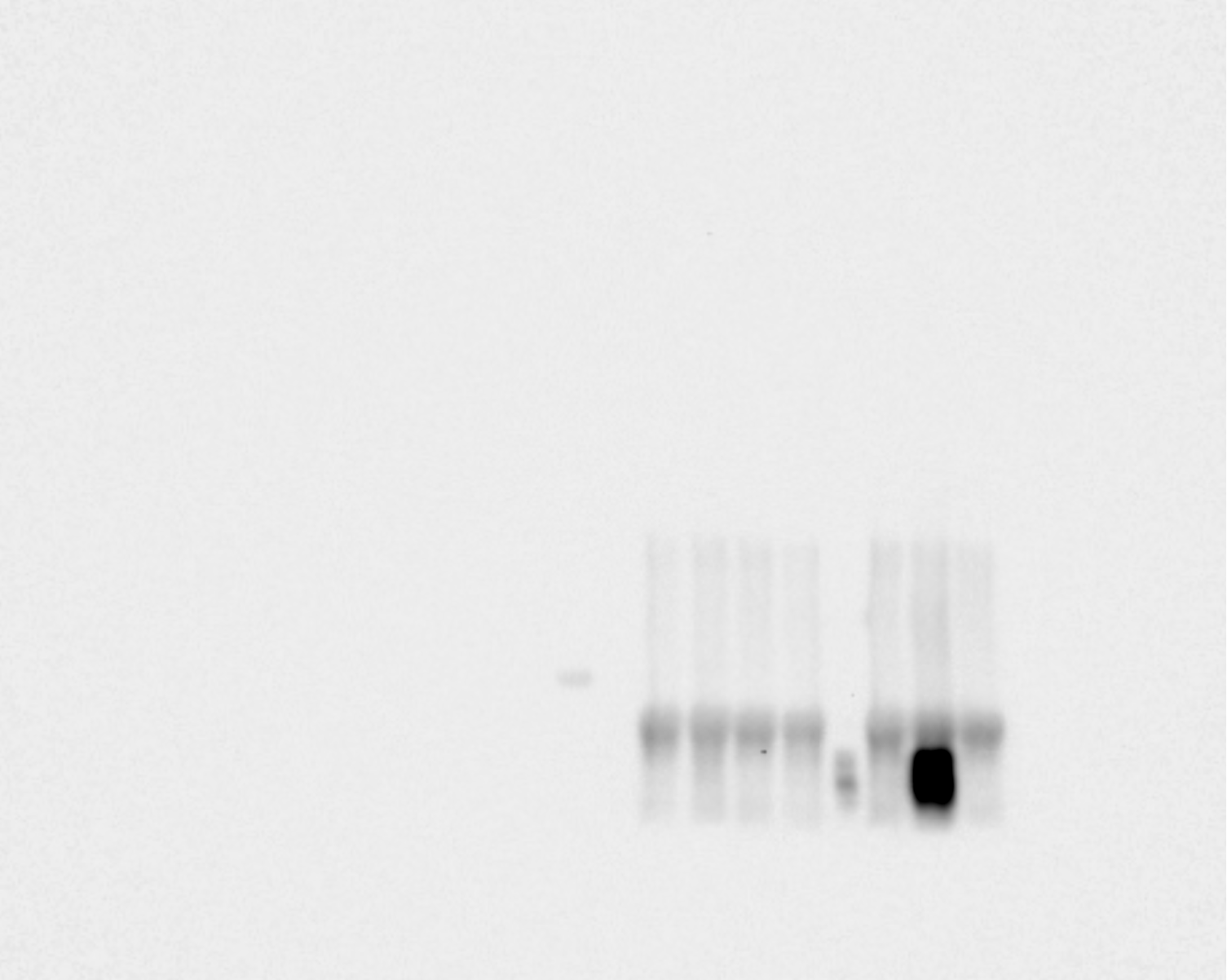

Supplement: Figure 1—figure supplement 1—source data 7. [file elife-83159-fig1-figsupp1-data7.zip › TTP Figure 1-figure supplement 1-source data 7/Versteeg 2021-07-25 17h16m25s 16.201s(Chemiluminescence).tif]

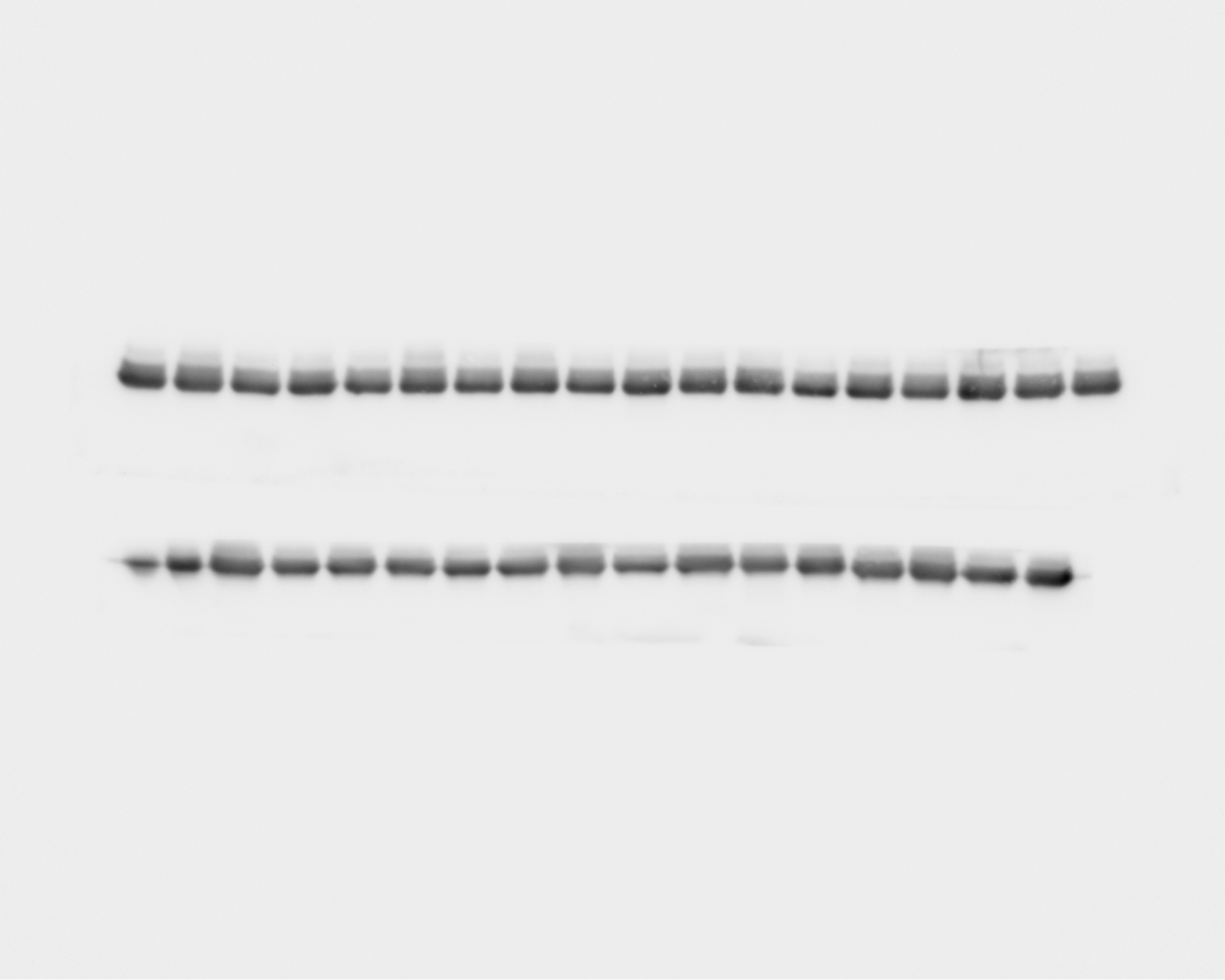

Supplement: Figure 2—source data 1. [file elife-83159-fig2-data1.zip › Figure 2-source data 1/ACTIN Figure 2-source data 1/Versteeg 2021-04-02 14h01m10s 30.000s(Chemiluminescence).jpg]

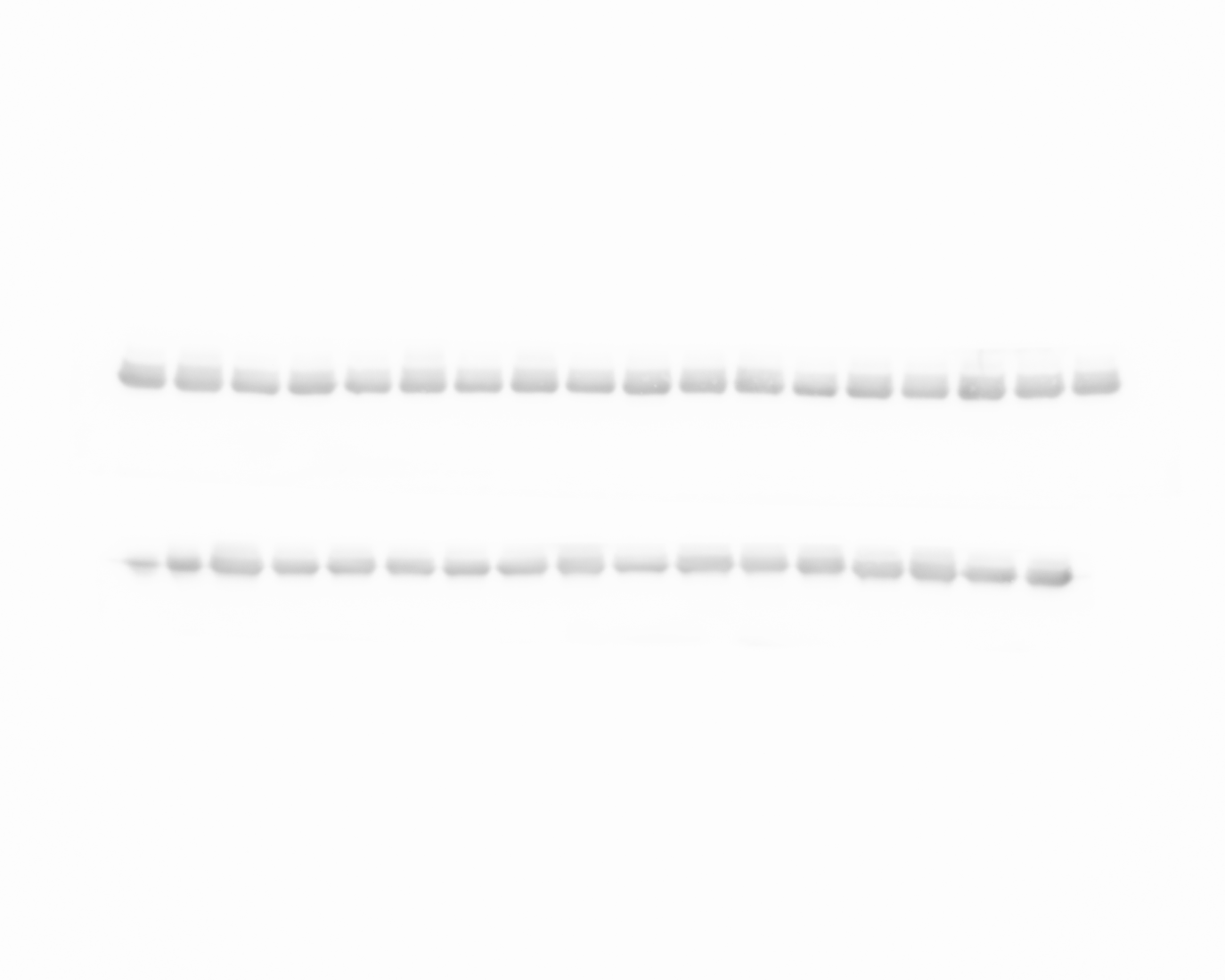

Supplement: Figure 2—source data 1. [file elife-83159-fig2-data1.zip › Figure 2-source data 1/ACTIN Figure 2-source data 1/Versteeg 2021-04-02 14h01m10s 30.000s(Chemiluminescence).raw16.tif]

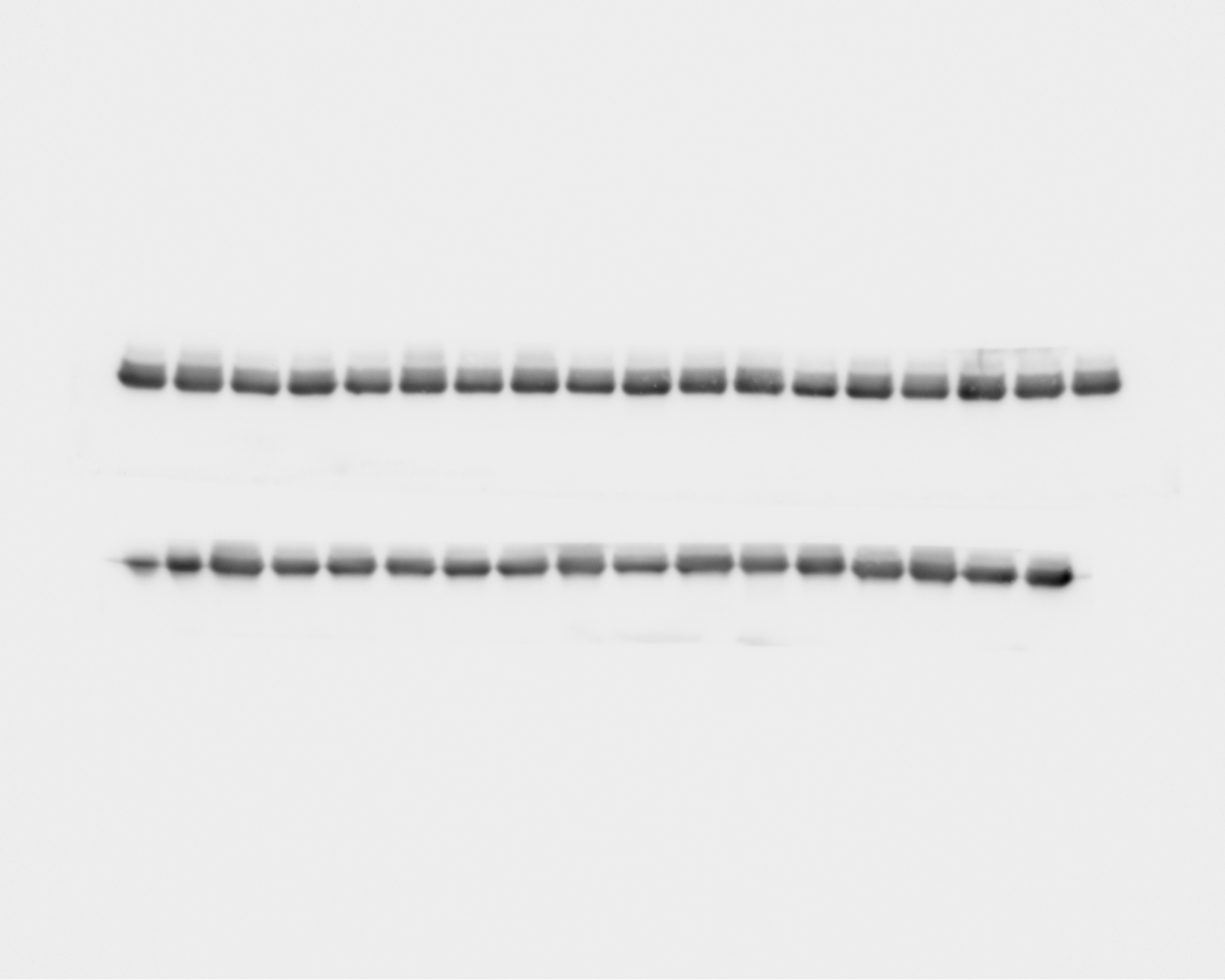

Supplement: Figure 2—source data 1. [file elife-83159-fig2-data1.zip › Figure 2-source data 1/ACTIN Figure 2-source data 1/Versteeg 2021-04-02 14h01m10s 30.000s(Chemiluminescence).tif]

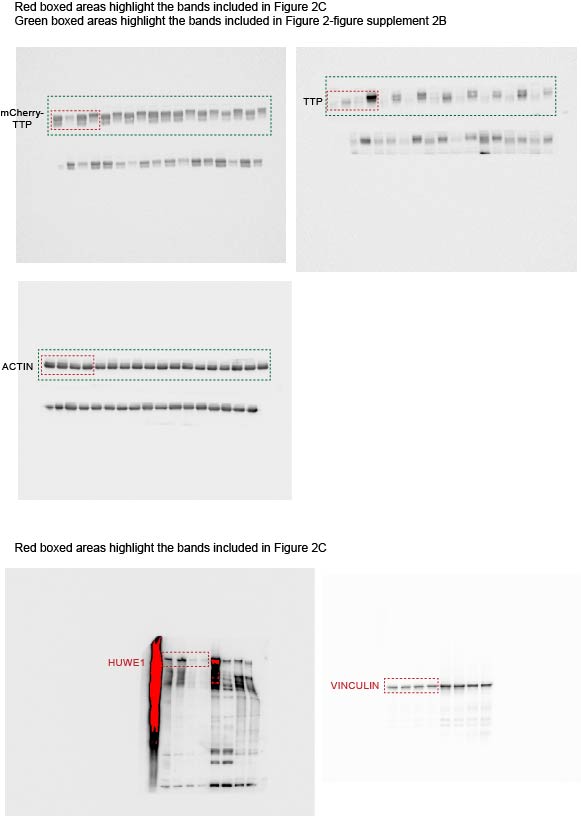

Supplement: Figure 2—source data 1. [file elife-83159-fig2-data1.zip › Figure 2-source data 1/Figure 2-source data 1.jpg]

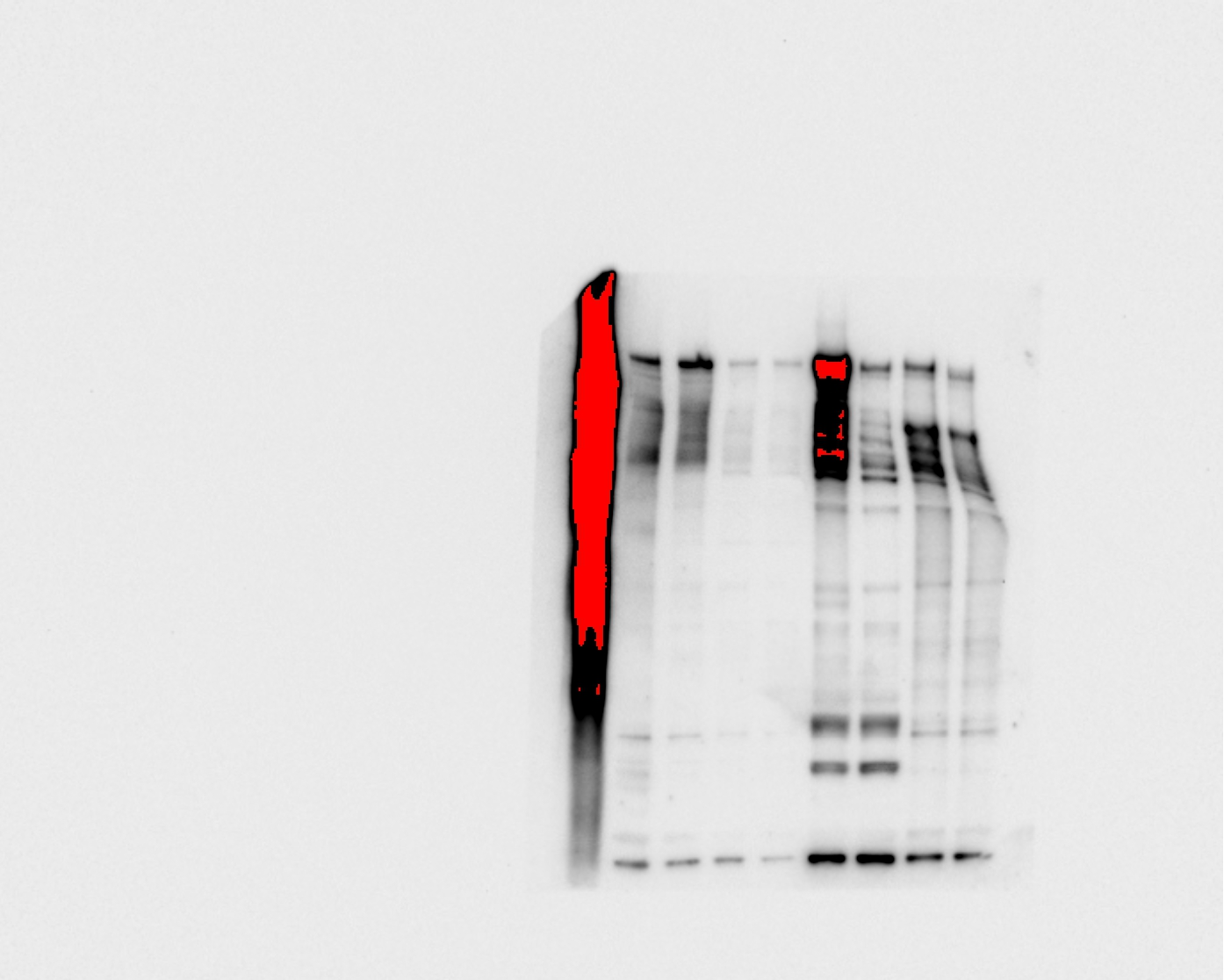

Supplement: Figure 2—source data 1. [file elife-83159-fig2-data1.zip › Figure 2-source data 1/HUWE1 Figure 2-source data 1/Run 2021-04-14 13h17m53s 60.000s(Chemiluminescence).jpg]

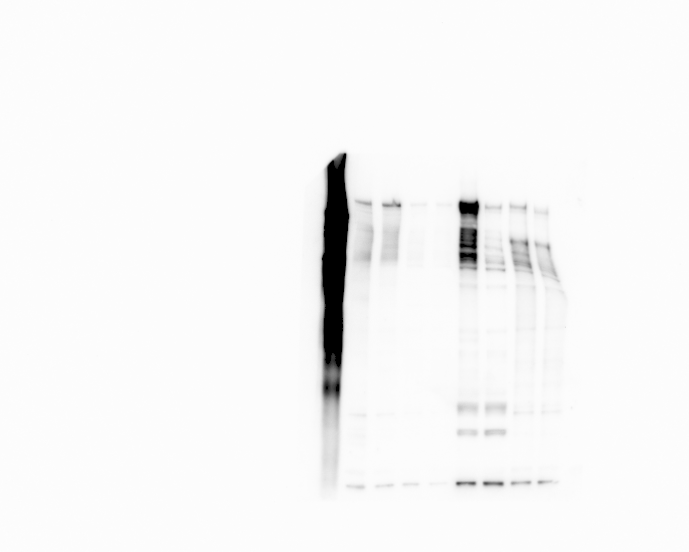

Supplement: Figure 2—source data 1. [file elife-83159-fig2-data1.zip › Figure 2-source data 1/HUWE1 Figure 2-source data 1/Run 2021-04-14 13h17m53s 60.000s(Chemiluminescence).raw16.tif]

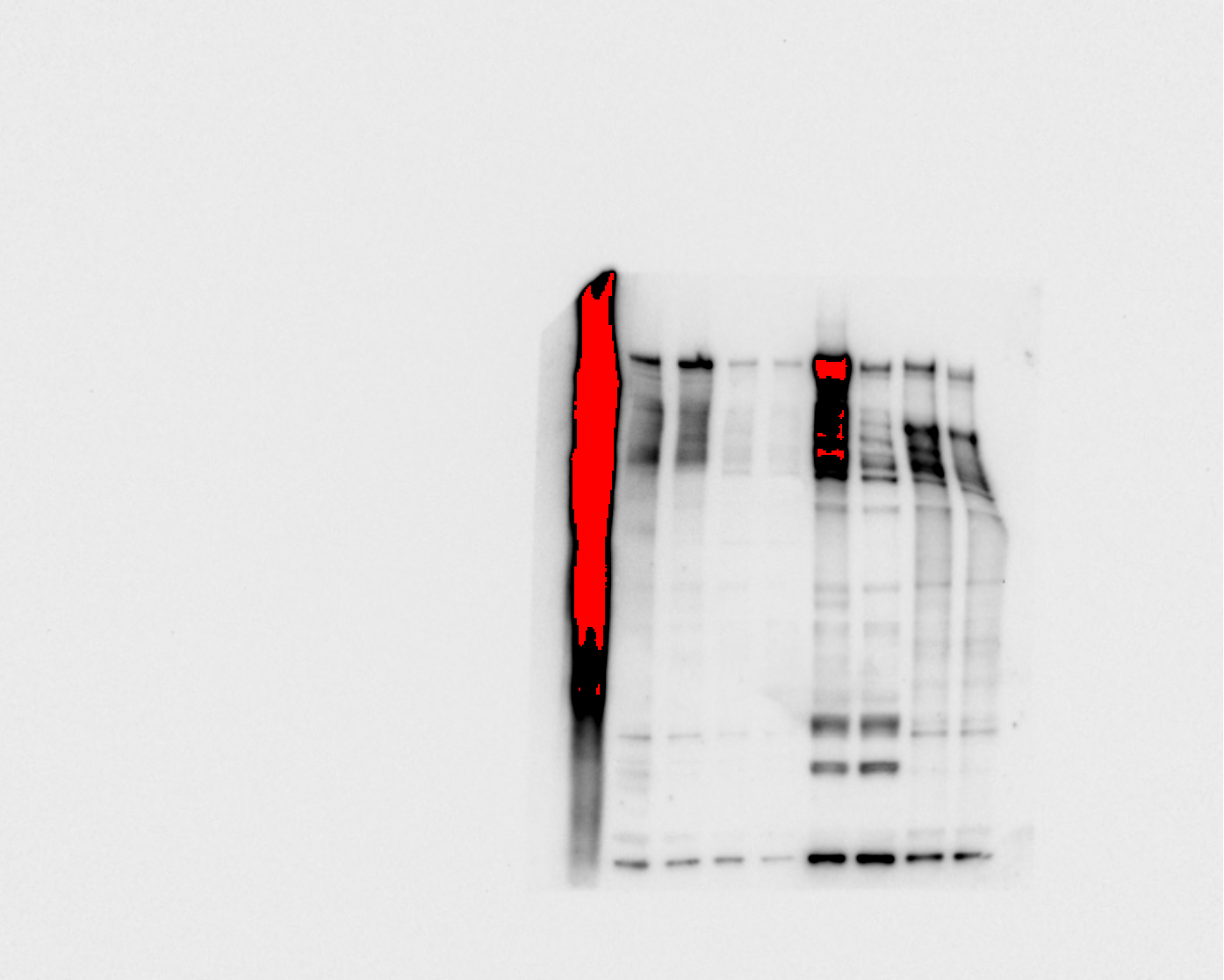

Supplement: Figure 2—source data 1. [file elife-83159-fig2-data1.zip › Figure 2-source data 1/HUWE1 Figure 2-source data 1/Run 2021-04-14 13h17m53s 60.000s(Chemiluminescence).tif]

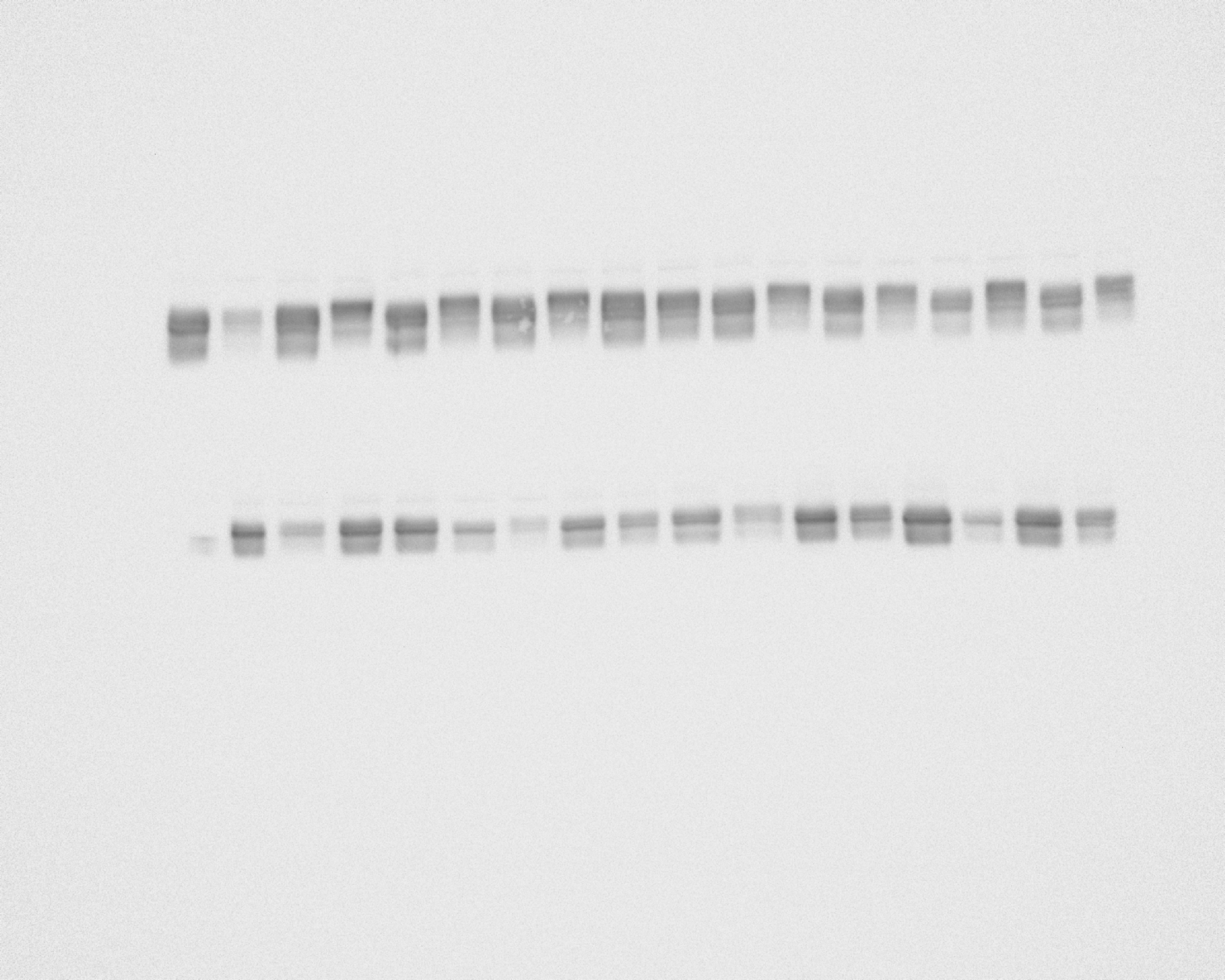

Supplement: Figure 2—source data 1. [file elife-83159-fig2-data1.zip › Figure 2-source data 1/mCherry-TTP Figure 2-source data 1/Versteeg 2021-04-02 11h49m07s 171.856s(Chemiluminescence).jpg]

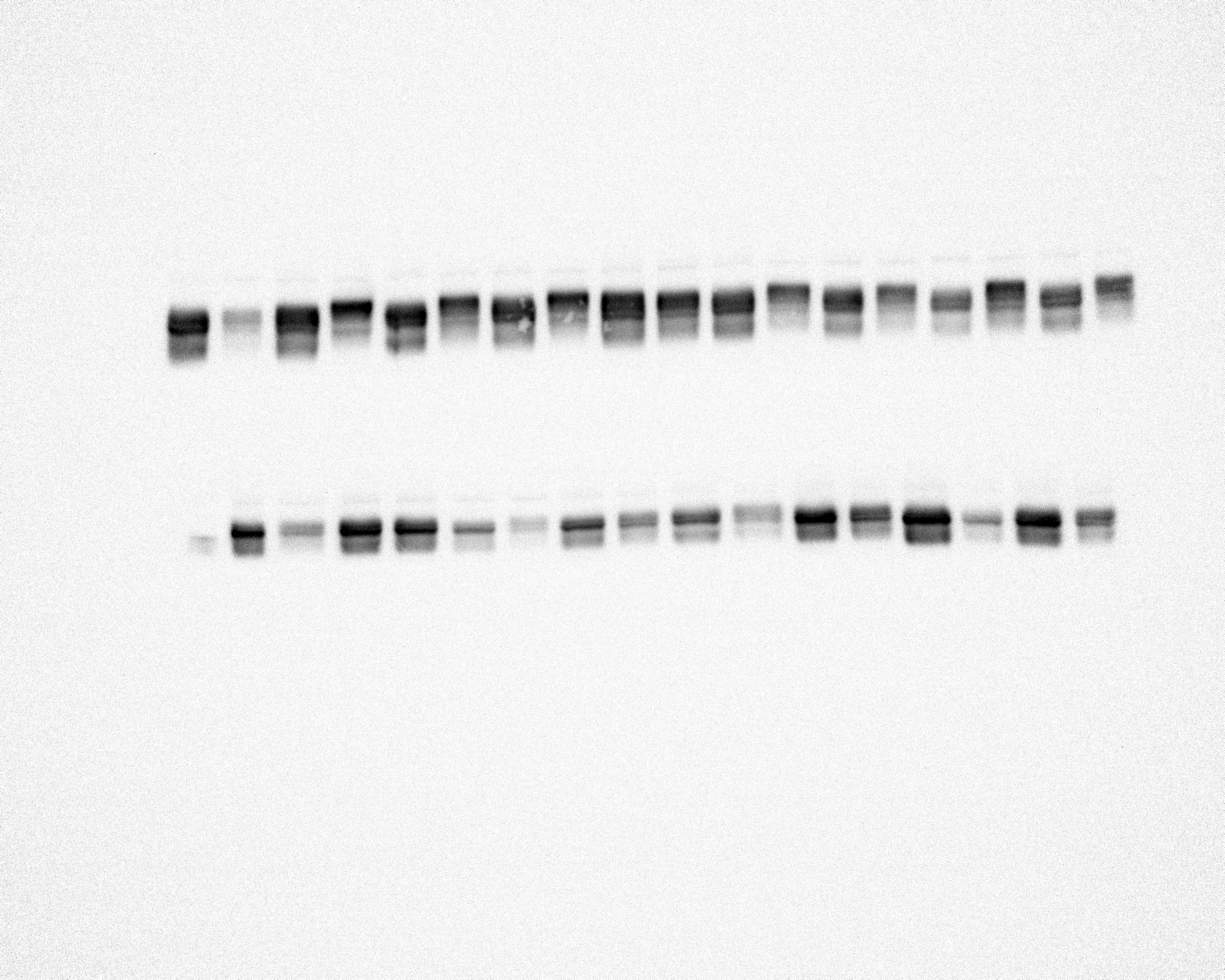

Supplement: Figure 2—source data 1. [file elife-83159-fig2-data1.zip › Figure 2-source data 1/mCherry-TTP Figure 2-source data 1/Versteeg 2021-04-02 11h49m07s 171.856s(Chemiluminescence).raw16.tif]

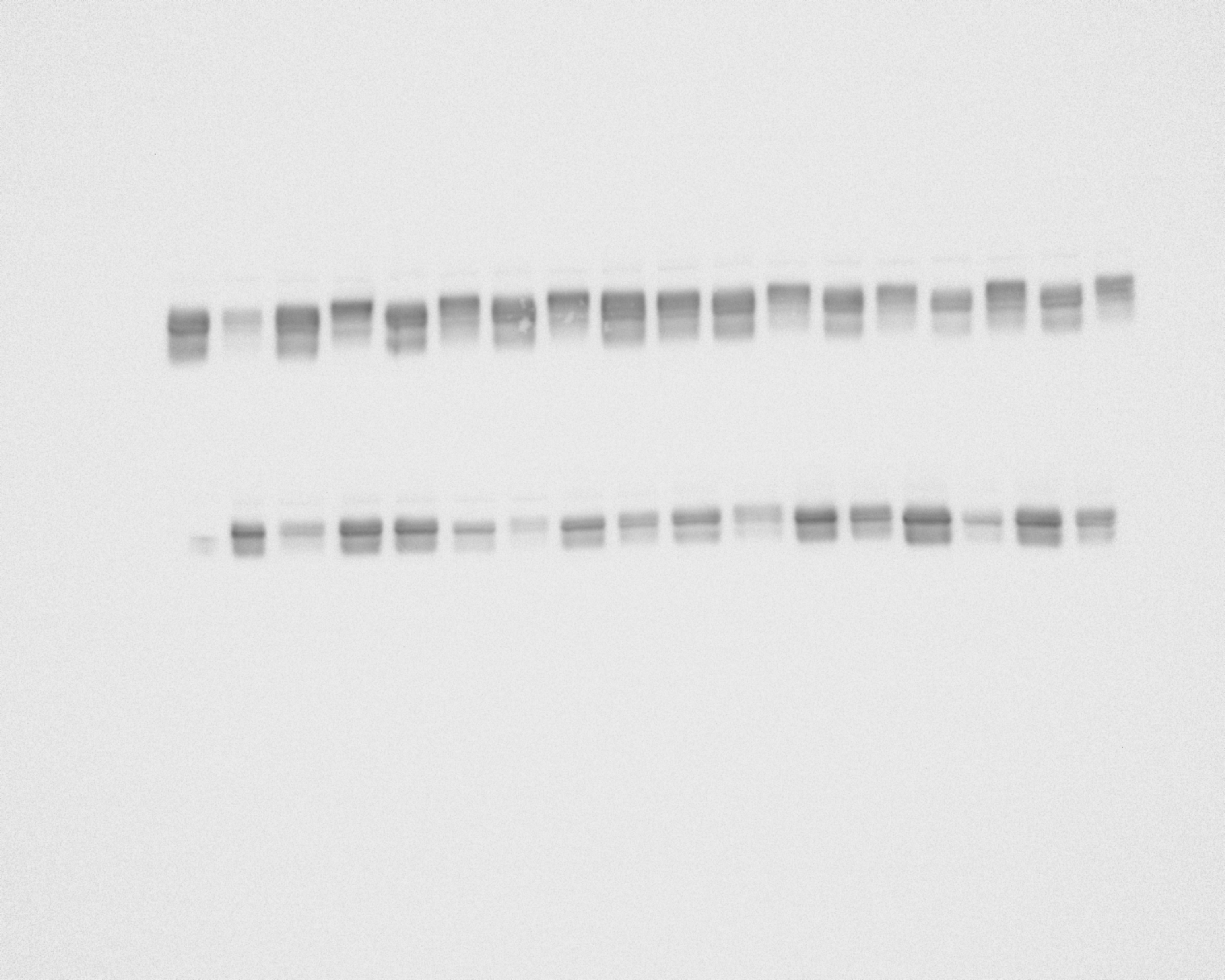

Supplement: Figure 2—source data 1. [file elife-83159-fig2-data1.zip › Figure 2-source data 1/mCherry-TTP Figure 2-source data 1/Versteeg 2021-04-02 11h49m07s 171.856s(Chemiluminescence).tif]

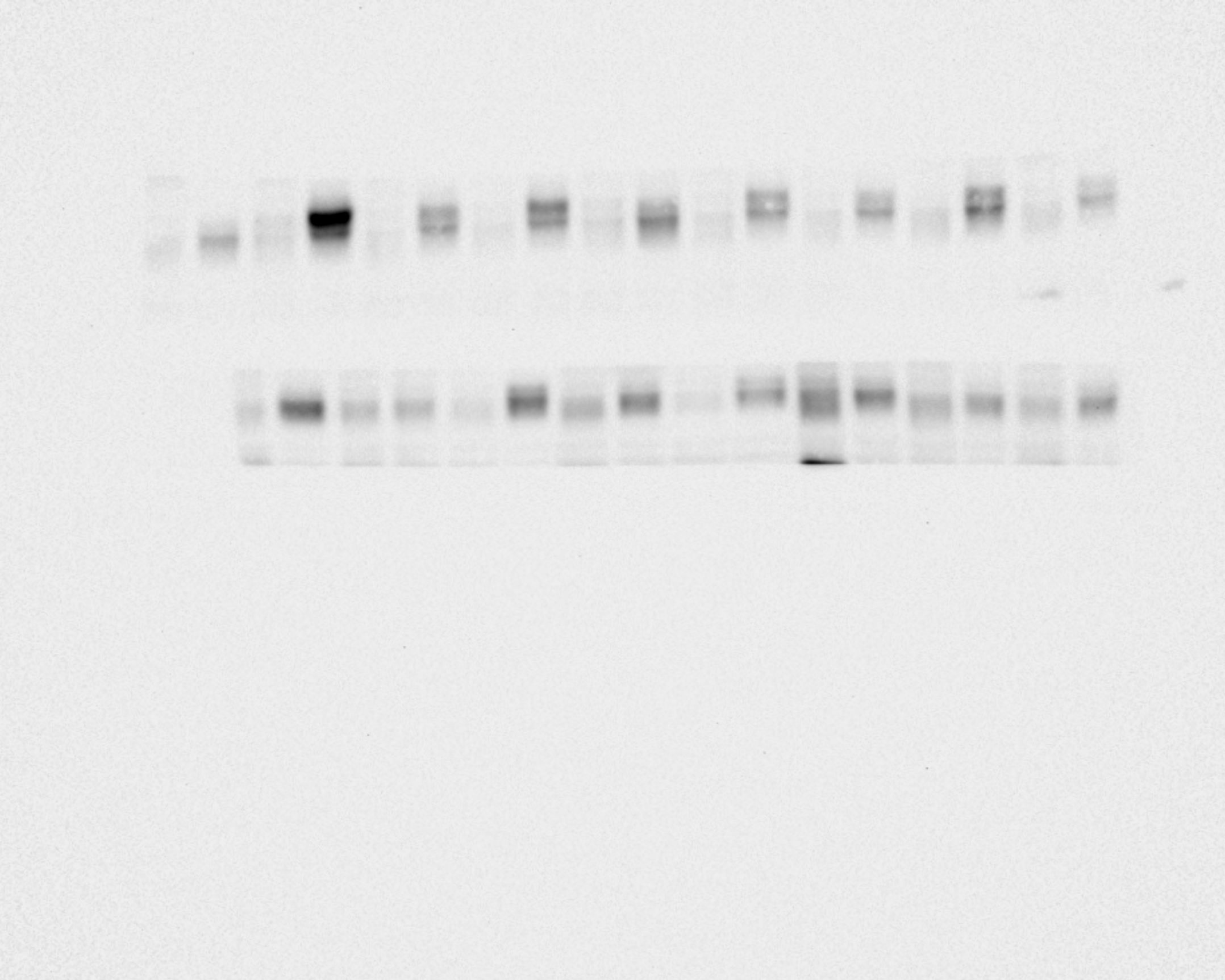

Supplement: Figure 2—source data 1. [file elife-83159-fig2-data1.zip › Figure 2-source data 1/TTP Figure 2-source data 1/Versteeg 2021-04-02 12h07m35s 300.000s(Chemiluminescence).jpg]

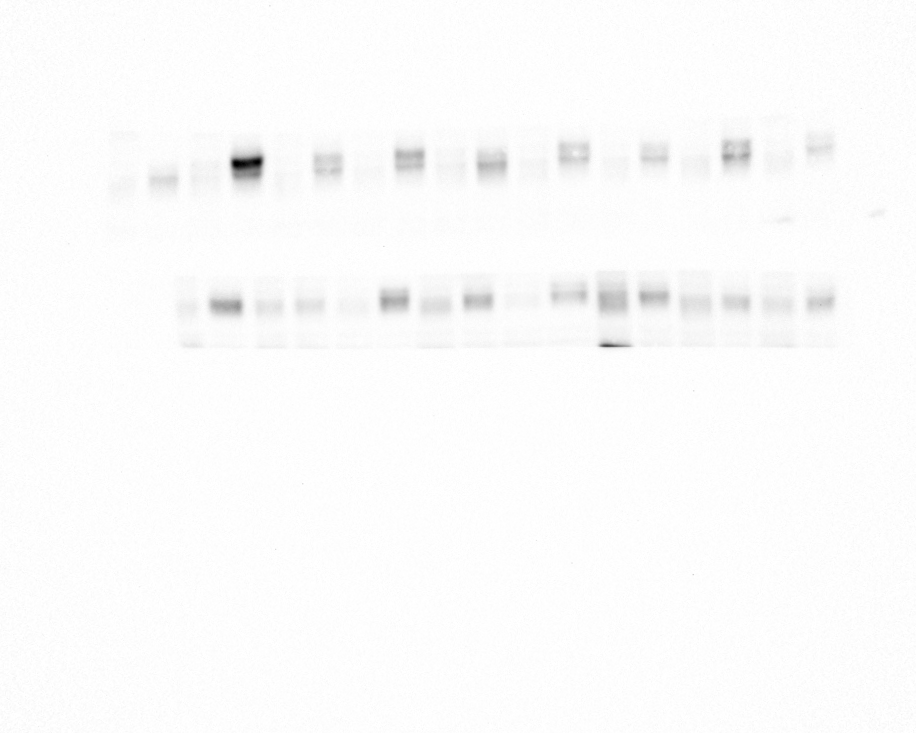

Supplement: Figure 2—source data 1. [file elife-83159-fig2-data1.zip › Figure 2-source data 1/TTP Figure 2-source data 1/Versteeg 2021-04-02 12h07m35s 300.000s(Chemiluminescence).raw16.tif]

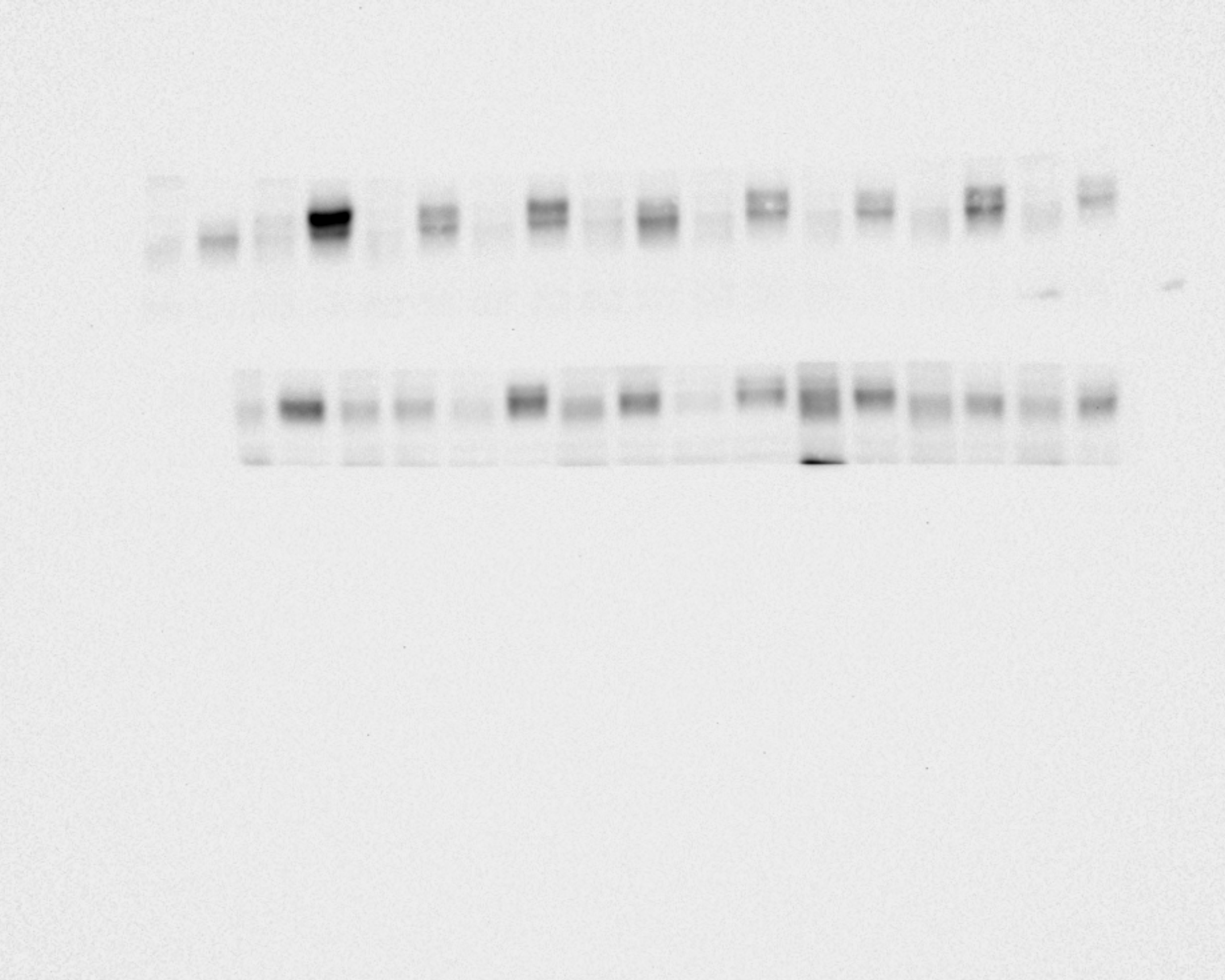

Supplement: Figure 2—source data 1. [file elife-83159-fig2-data1.zip › Figure 2-source data 1/TTP Figure 2-source data 1/Versteeg 2021-04-02 12h07m35s 300.000s(Chemiluminescence).tif]

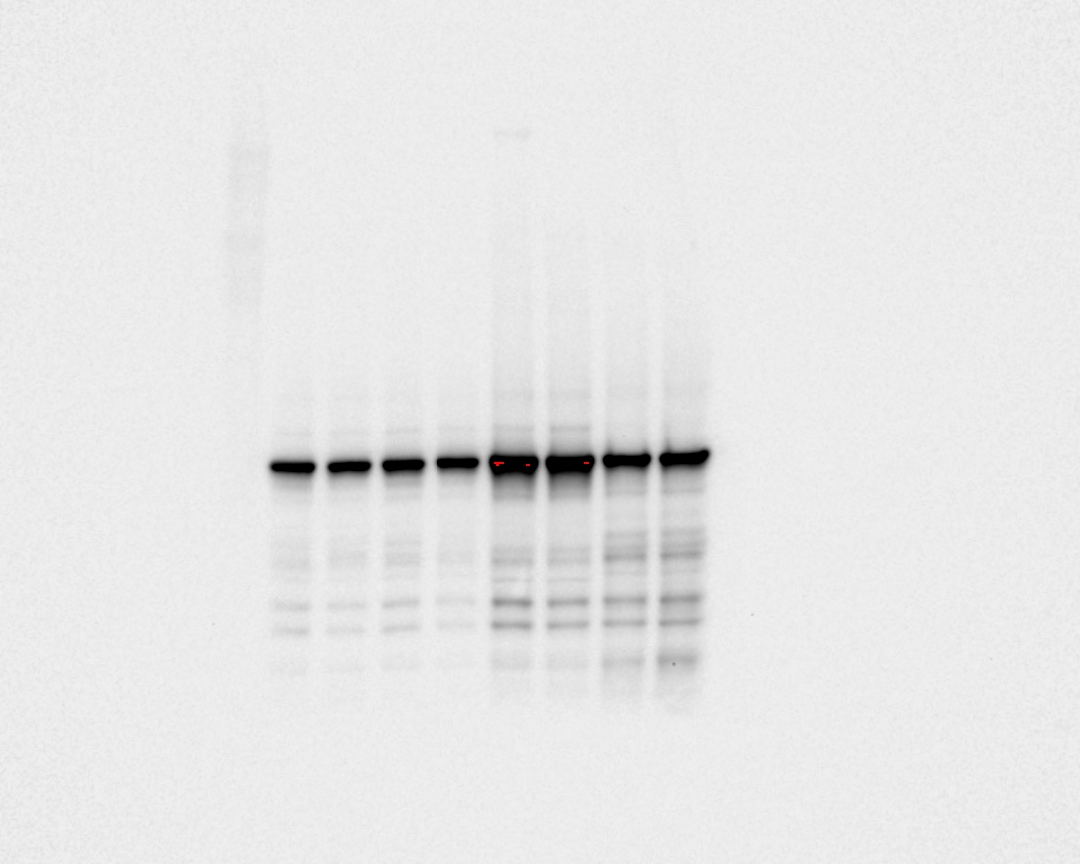

Supplement: Figure 2—source data 1. [file elife-83159-fig2-data1.zip › Figure 2-source data 1/VINCULIN Figure 2-source data 1/Versteeg 2021-04-16 12h51m17s 15.568s(Chemiluminescence).jpg]

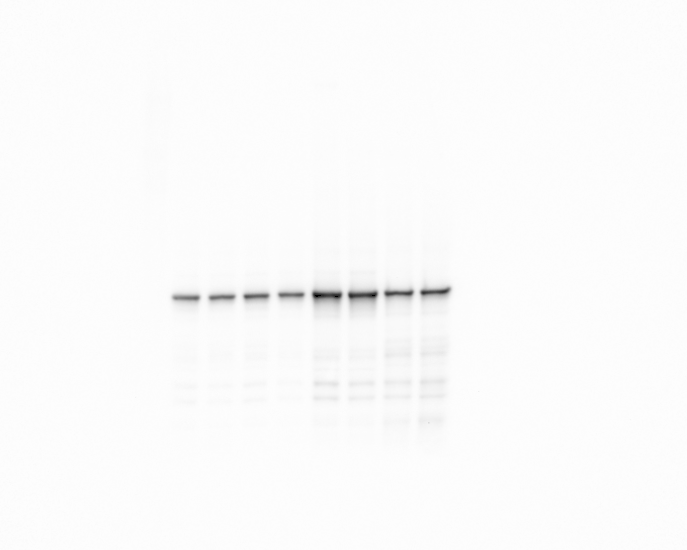

Supplement: Figure 2—source data 1. [file elife-83159-fig2-data1.zip › Figure 2-source data 1/VINCULIN Figure 2-source data 1/Versteeg 2021-04-16 12h51m17s 15.568s(Chemiluminescence).raw16.tif]

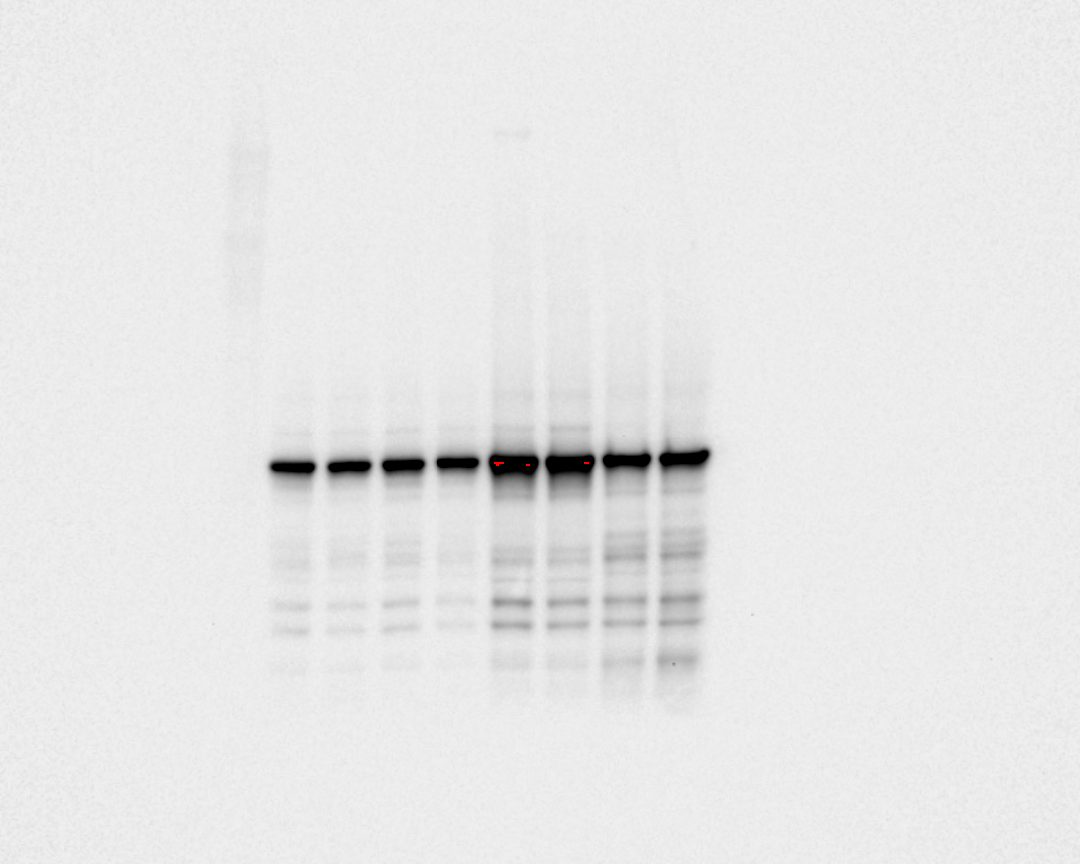

Supplement: Figure 2—source data 1. [file elife-83159-fig2-data1.zip › Figure 2-source data 1/VINCULIN Figure 2-source data 1/Versteeg 2021-04-16 12h51m17s 15.568s(Chemiluminescence).tif]

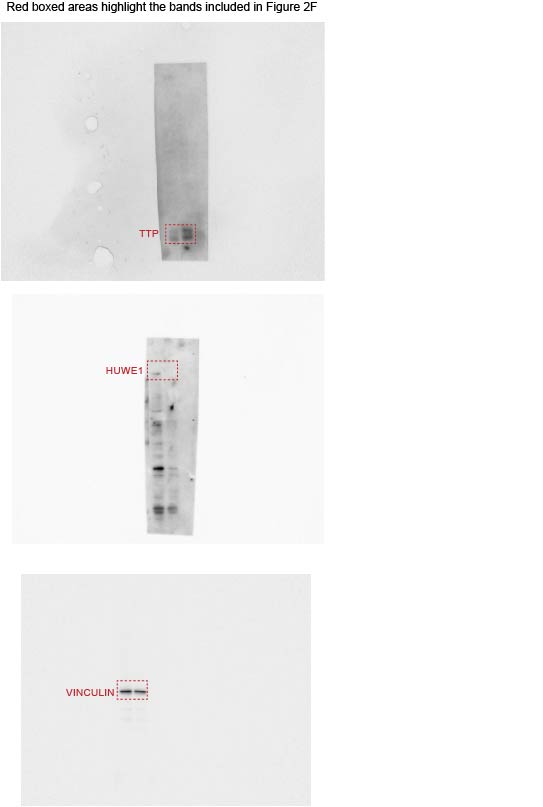

Supplement: Figure 2—source data 2. [file elife-83159-fig2-data2.zip › Figure 2-source data 2/Figure 2-source data 2.jpg]

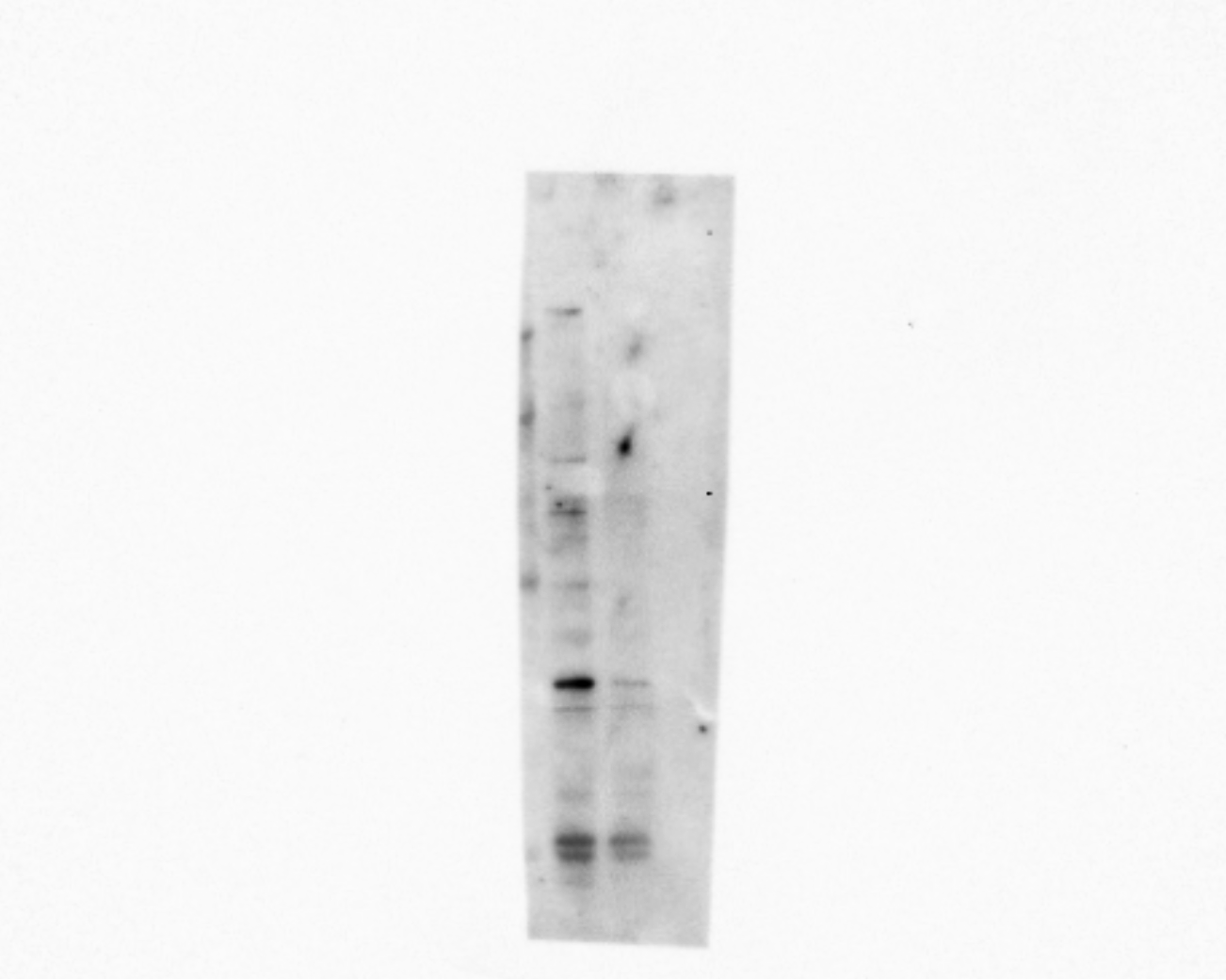

Supplement: Figure 2—source data 2. [file elife-83159-fig2-data2.zip › Figure 2-source data 2/HUWE1 Figure 2-source data 2/Versteeg 2021-10-19 13h48m22s 95.170s(Chemiluminescence) modified.tif]

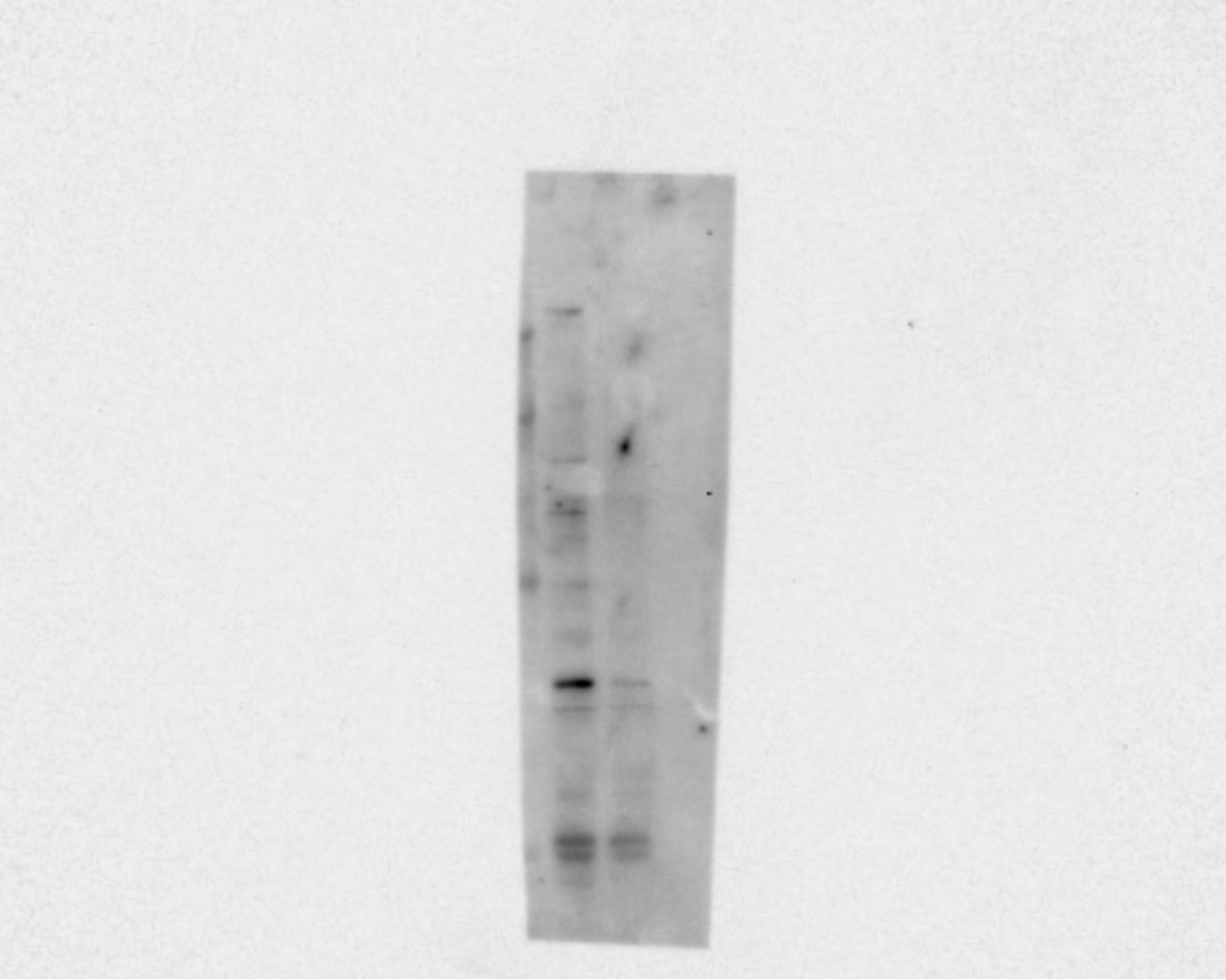

Supplement: Figure 2—source data 2. [file elife-83159-fig2-data2.zip › Figure 2-source data 2/HUWE1 Figure 2-source data 2/Versteeg 2021-10-19 13h48m22s 95.170s(Chemiluminescence).jpg]

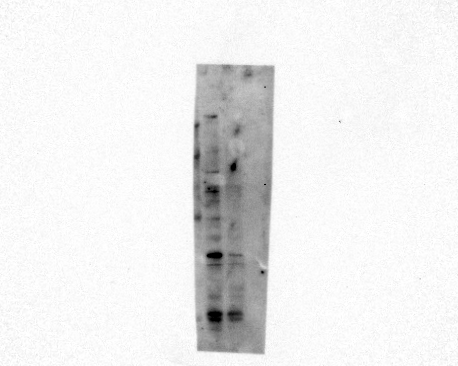

Supplement: Figure 2—source data 2. [file elife-83159-fig2-data2.zip › Figure 2-source data 2/HUWE1 Figure 2-source data 2/Versteeg 2021-10-19 13h48m22s 95.170s(Chemiluminescence).raw16.tif]

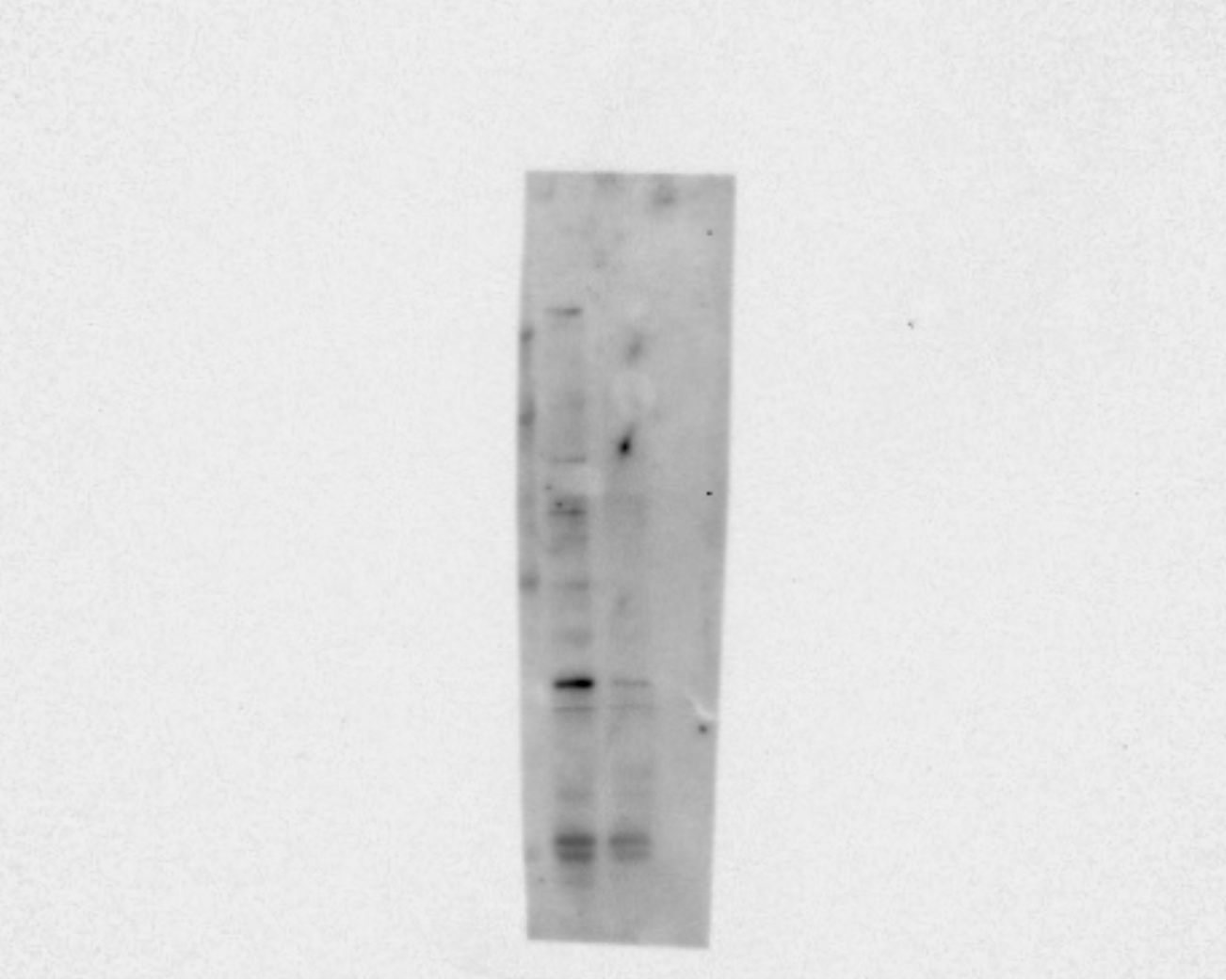

Supplement: Figure 2—source data 2. [file elife-83159-fig2-data2.zip › Figure 2-source data 2/HUWE1 Figure 2-source data 2/Versteeg 2021-10-19 13h48m22s 95.170s(Chemiluminescence).tif]

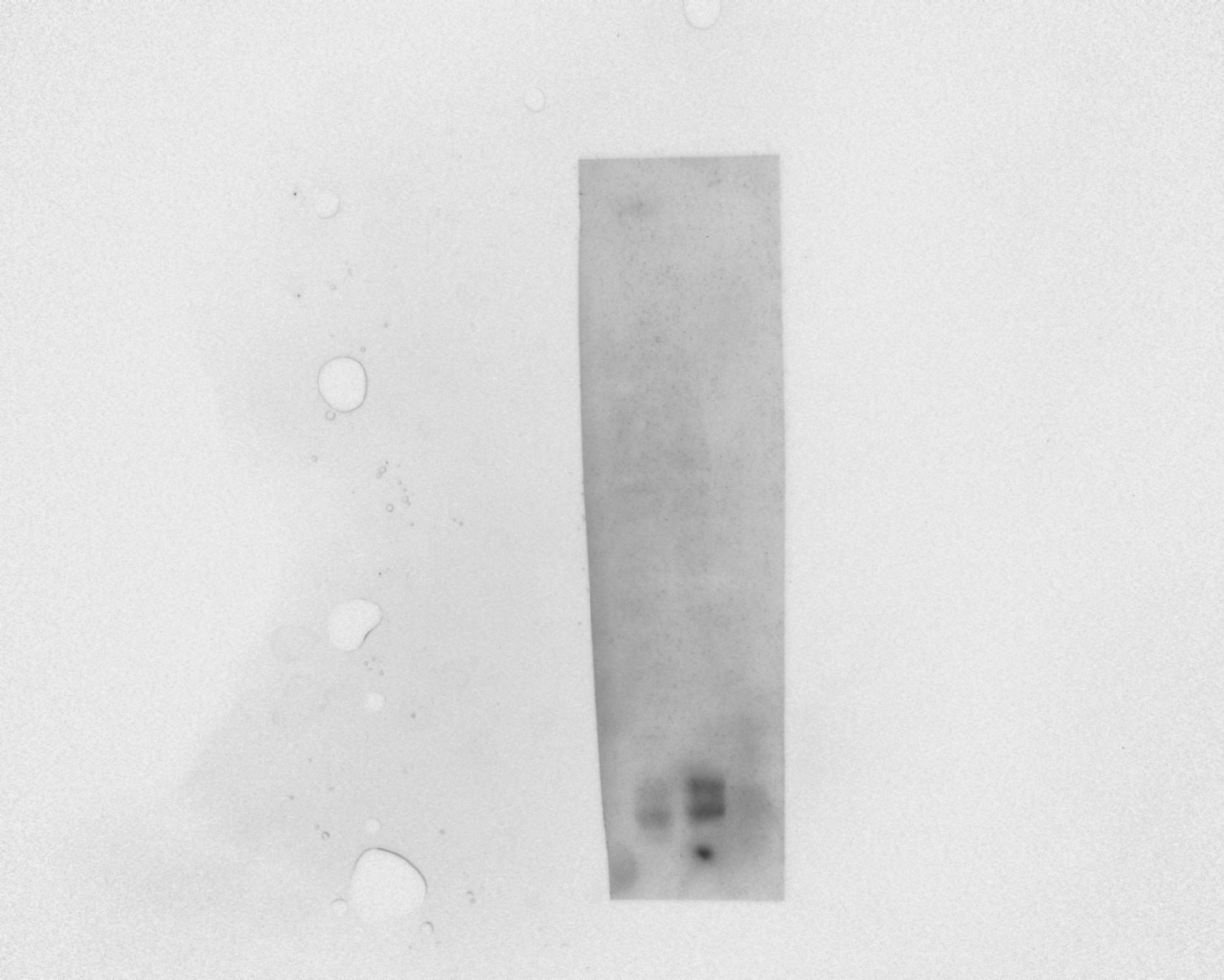

Supplement: Figure 2—source data 2. [file elife-83159-fig2-data2.zip › Figure 2-source data 2/TTP Figure 2-source data 2/Versteeg 2021-10-15 11h20m05s 228.940s(Chemiluminescence).jpg]

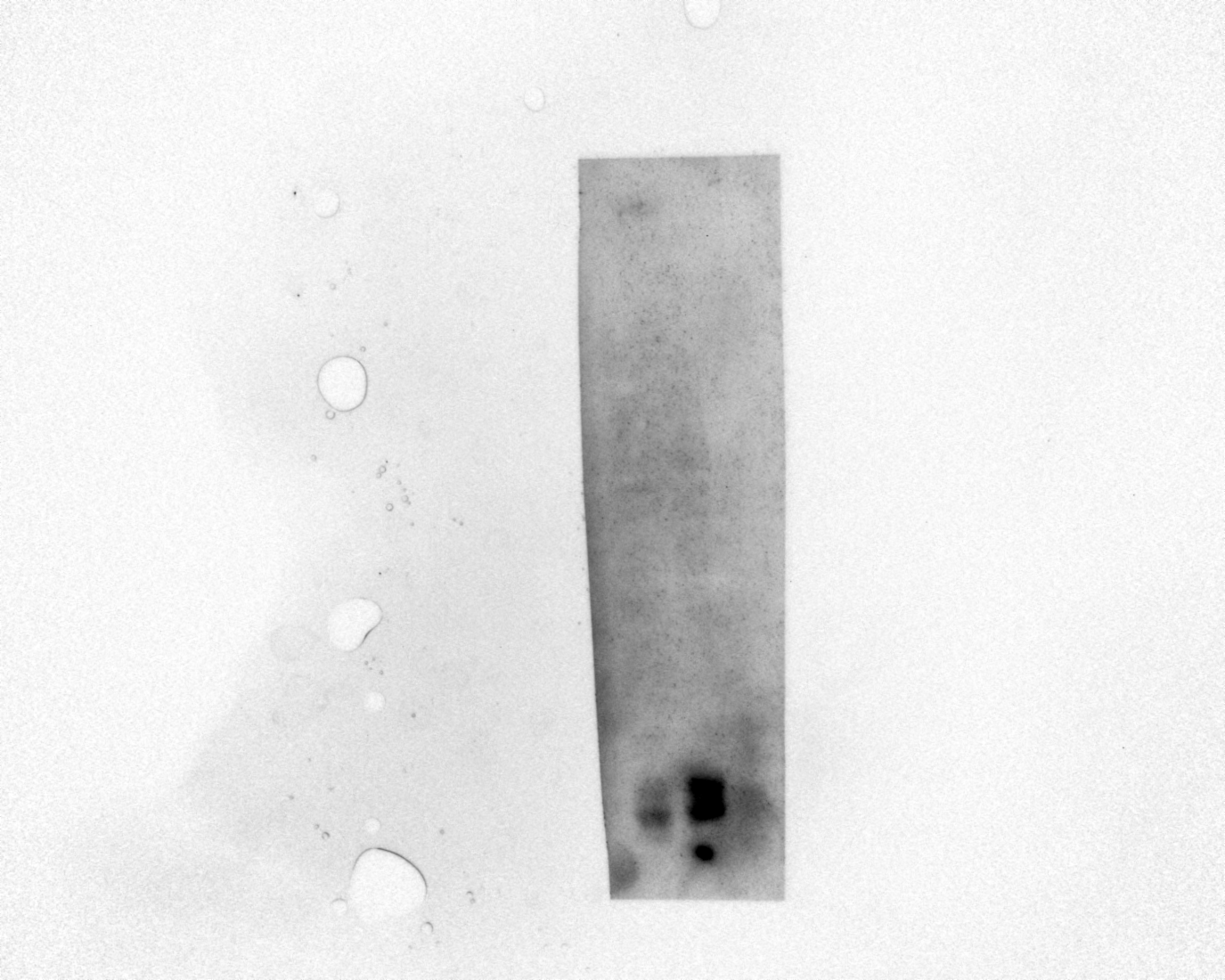

Supplement: Figure 2—source data 2. [file elife-83159-fig2-data2.zip › Figure 2-source data 2/TTP Figure 2-source data 2/Versteeg 2021-10-15 11h20m05s 228.940s(Chemiluminescence).raw16.tif]

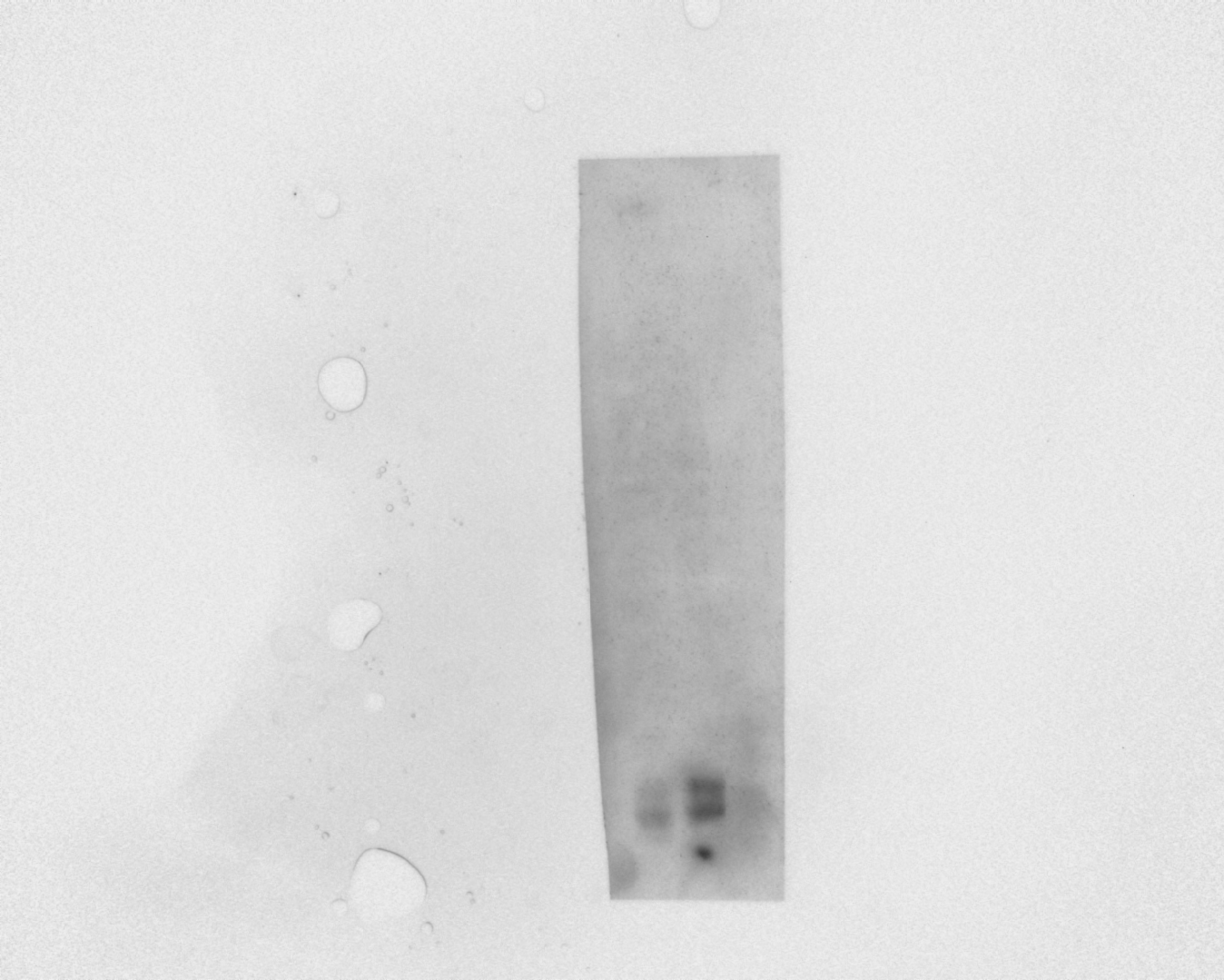

Supplement: Figure 2—source data 2. [file elife-83159-fig2-data2.zip › Figure 2-source data 2/TTP Figure 2-source data 2/Versteeg 2021-10-15 11h20m05s 228.940s(Chemiluminescence).tif]

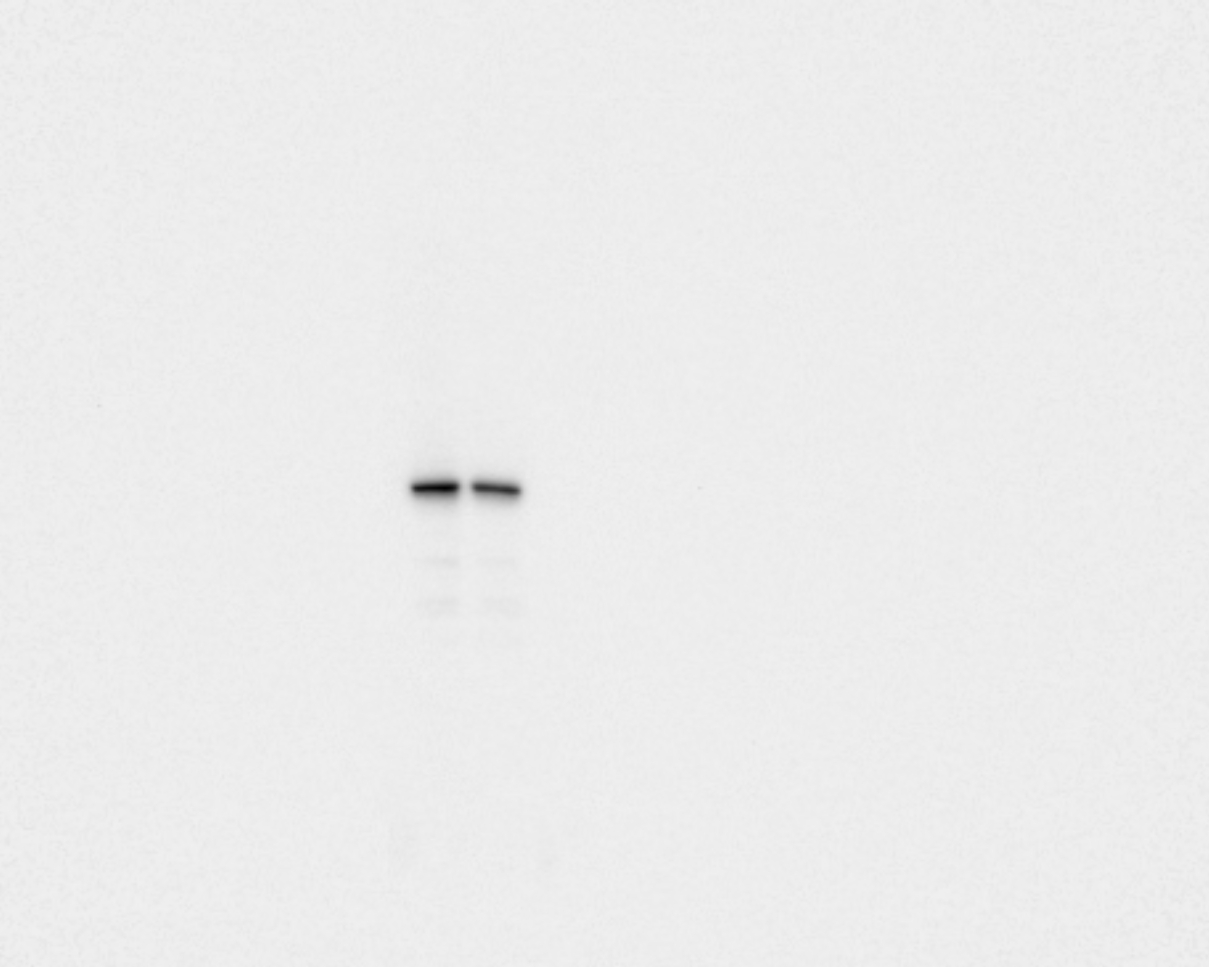

Supplement: Figure 2—source data 2. [file elife-83159-fig2-data2.zip › Figure 2-source data 2/VINCULIN Figure 2-source data 2/Versteeg 2021-10-19 16h49m06s 7.750s(Chemiluminescence).jpg]

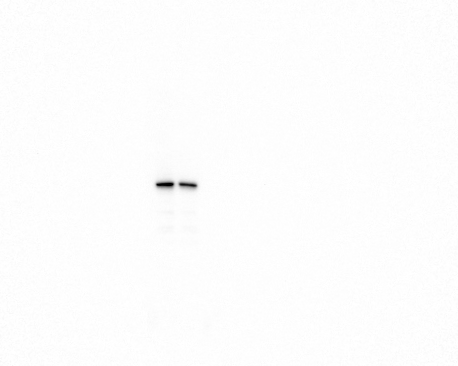

Supplement: Figure 2—source data 2. [file elife-83159-fig2-data2.zip › Figure 2-source data 2/VINCULIN Figure 2-source data 2/Versteeg 2021-10-19 16h49m06s 7.750s(Chemiluminescence).raw16.tif]

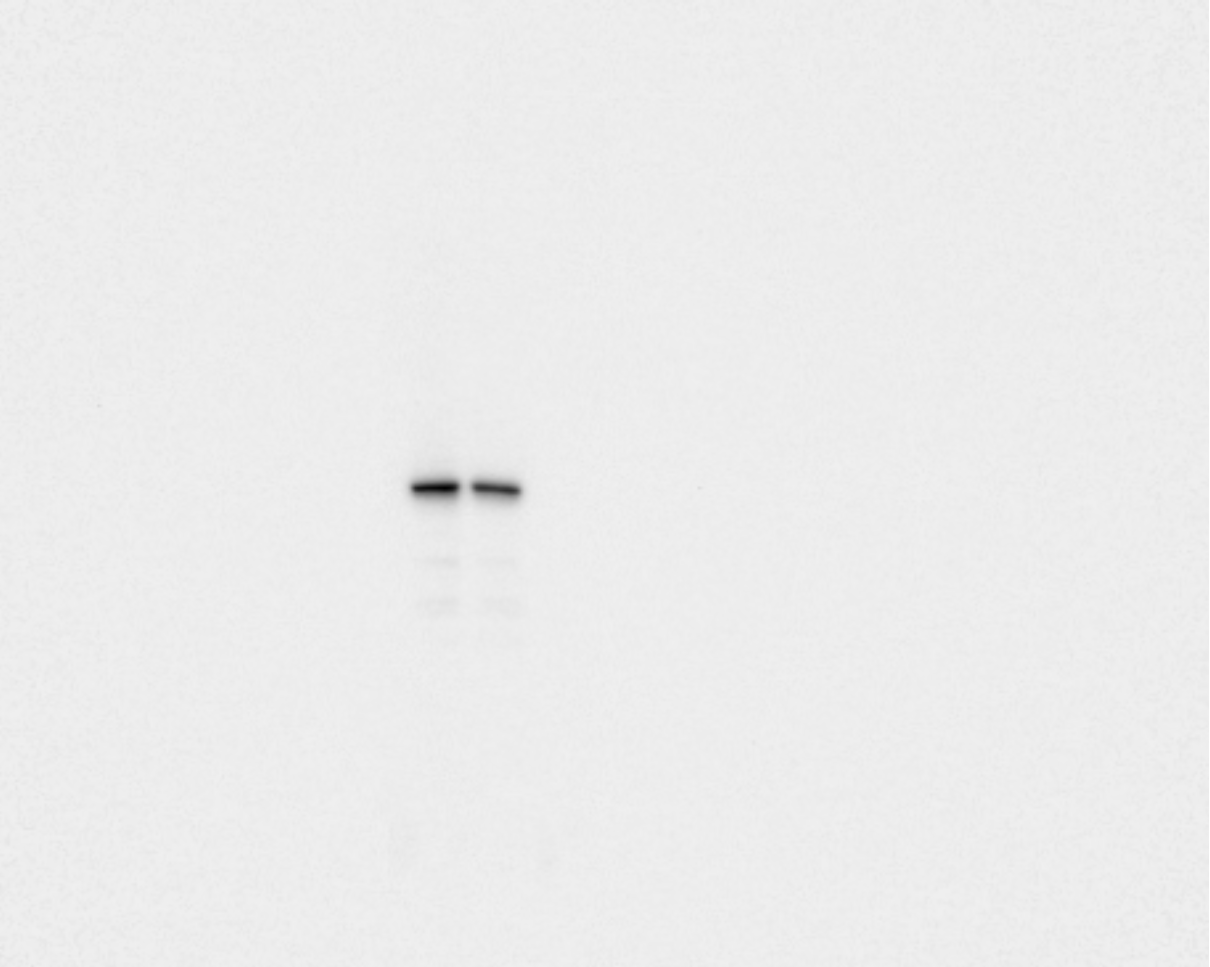

Supplement: Figure 2—source data 2. [file elife-83159-fig2-data2.zip › Figure 2-source data 2/VINCULIN Figure 2-source data 2/Versteeg 2021-10-19 16h49m06s 7.750s(Chemiluminescence).tif]

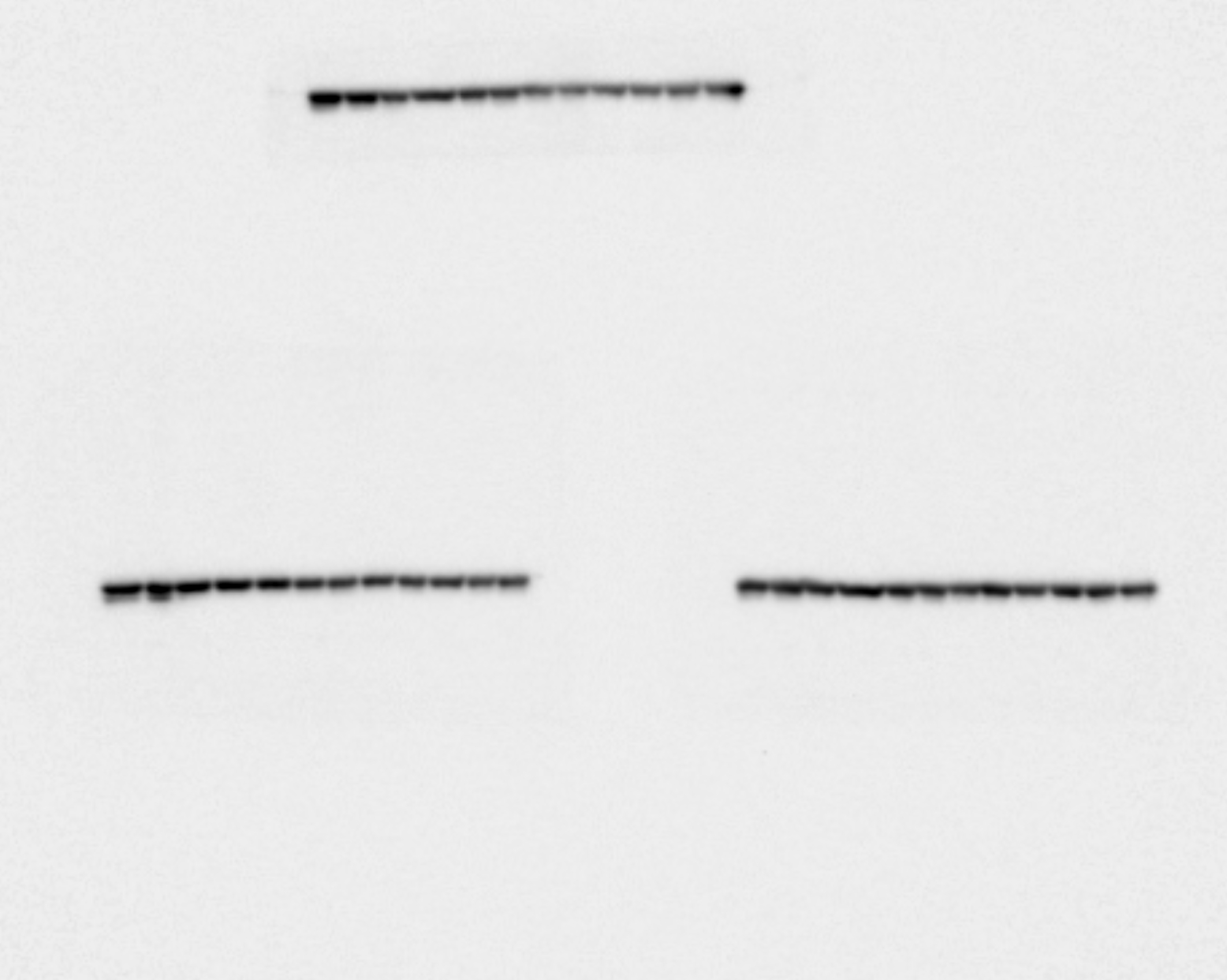

Supplement: Figure 2—source data 3. [file elife-83159-fig2-data3.zip › ACTIN Figure 2-source data 3/Versteeg 2023-01-27 17h00m01s 5.524s(Chemiluminescence).jpg]

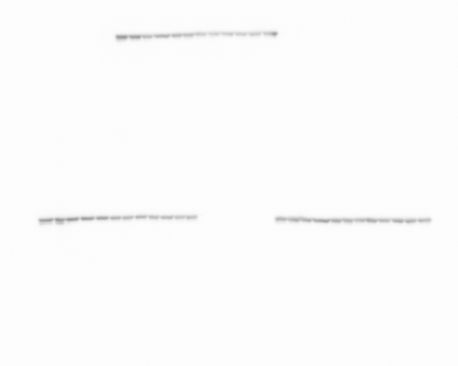

Supplement: Figure 2—source data 3. [file elife-83159-fig2-data3.zip › ACTIN Figure 2-source data 3/Versteeg 2023-01-27 17h00m01s 5.524s(Chemiluminescence).raw16.tif]

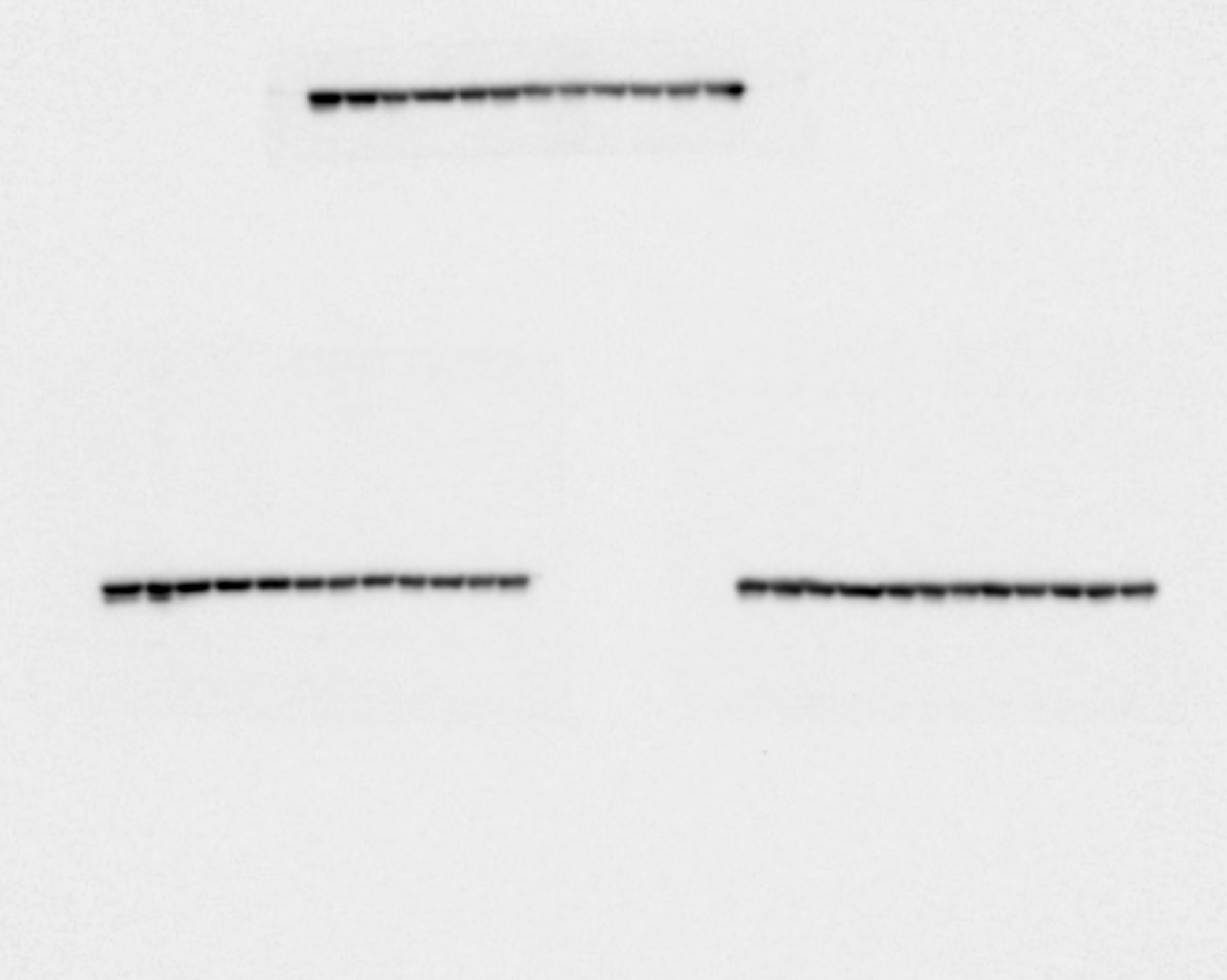

Supplement: Figure 2—source data 3. [file elife-83159-fig2-data3.zip › ACTIN Figure 2-source data 3/Versteeg 2023-01-27 17h00m01s 5.524s(Chemiluminescence).tif]

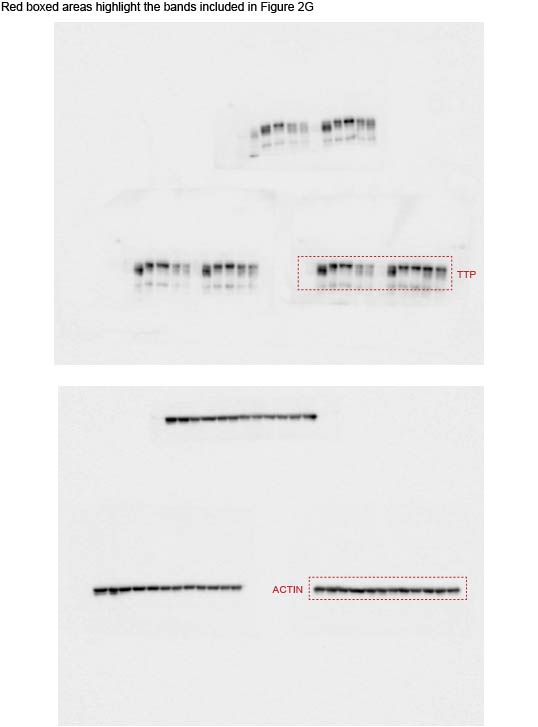

Supplement: Figure 2—source data 3. [file elife-83159-fig2-data3.zip › Figure 2-source data 3.jpg]

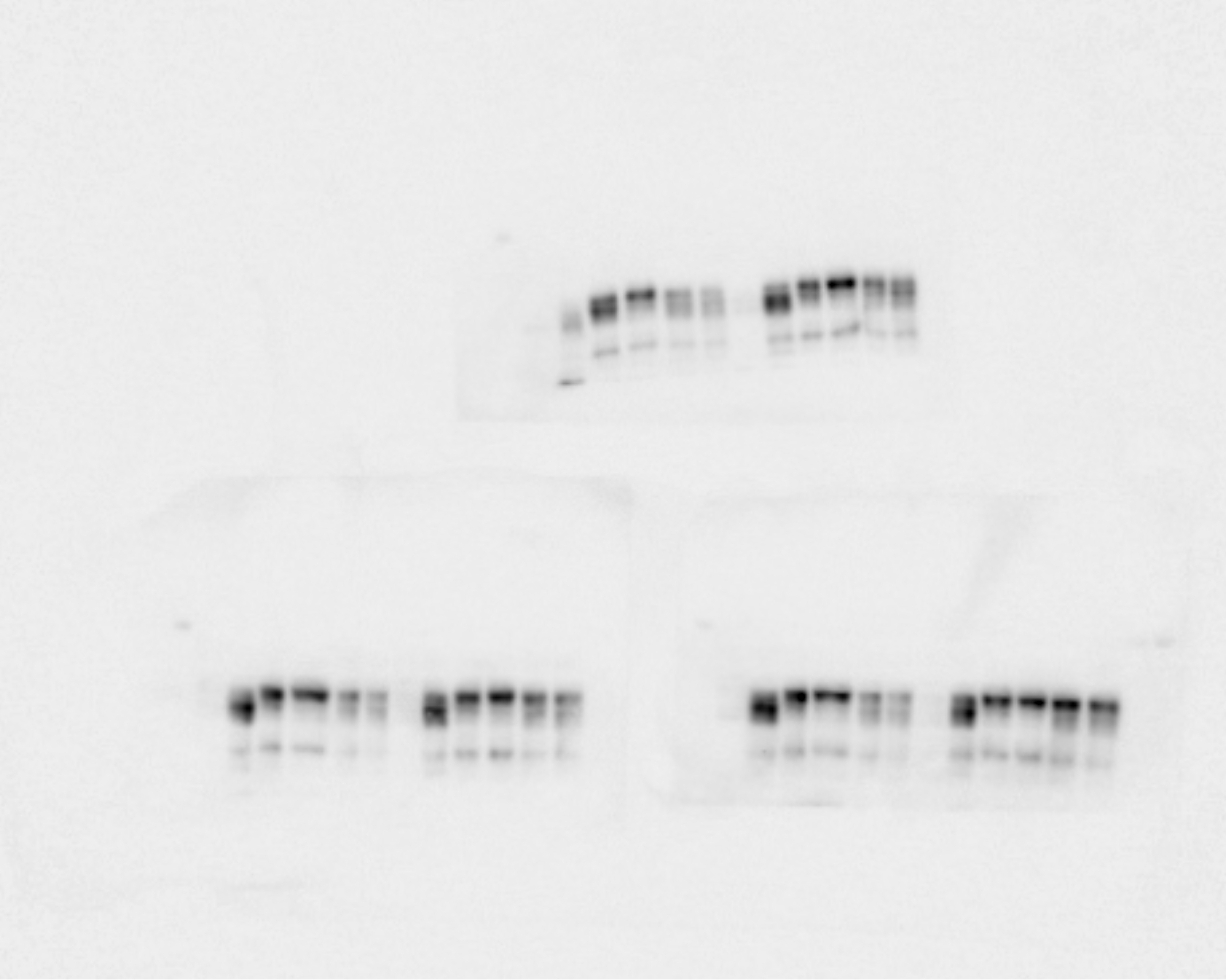

Supplement: Figure 2—source data 3. [file elife-83159-fig2-data3.zip › TTP Figure 2-source data 3/Versteeg 2023-01-26 11h53m39s 4.016s(Chemiluminescence).jpg]

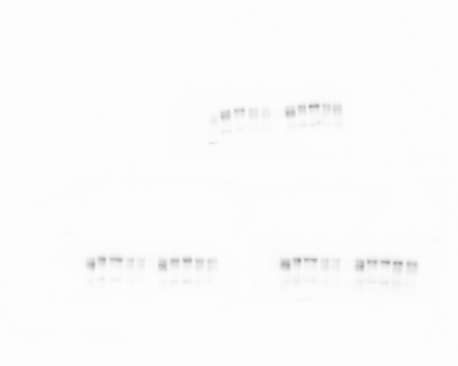

Supplement: Figure 2—source data 3. [file elife-83159-fig2-data3.zip › TTP Figure 2-source data 3/Versteeg 2023-01-26 11h53m39s 4.016s(Chemiluminescence).raw16.tif]

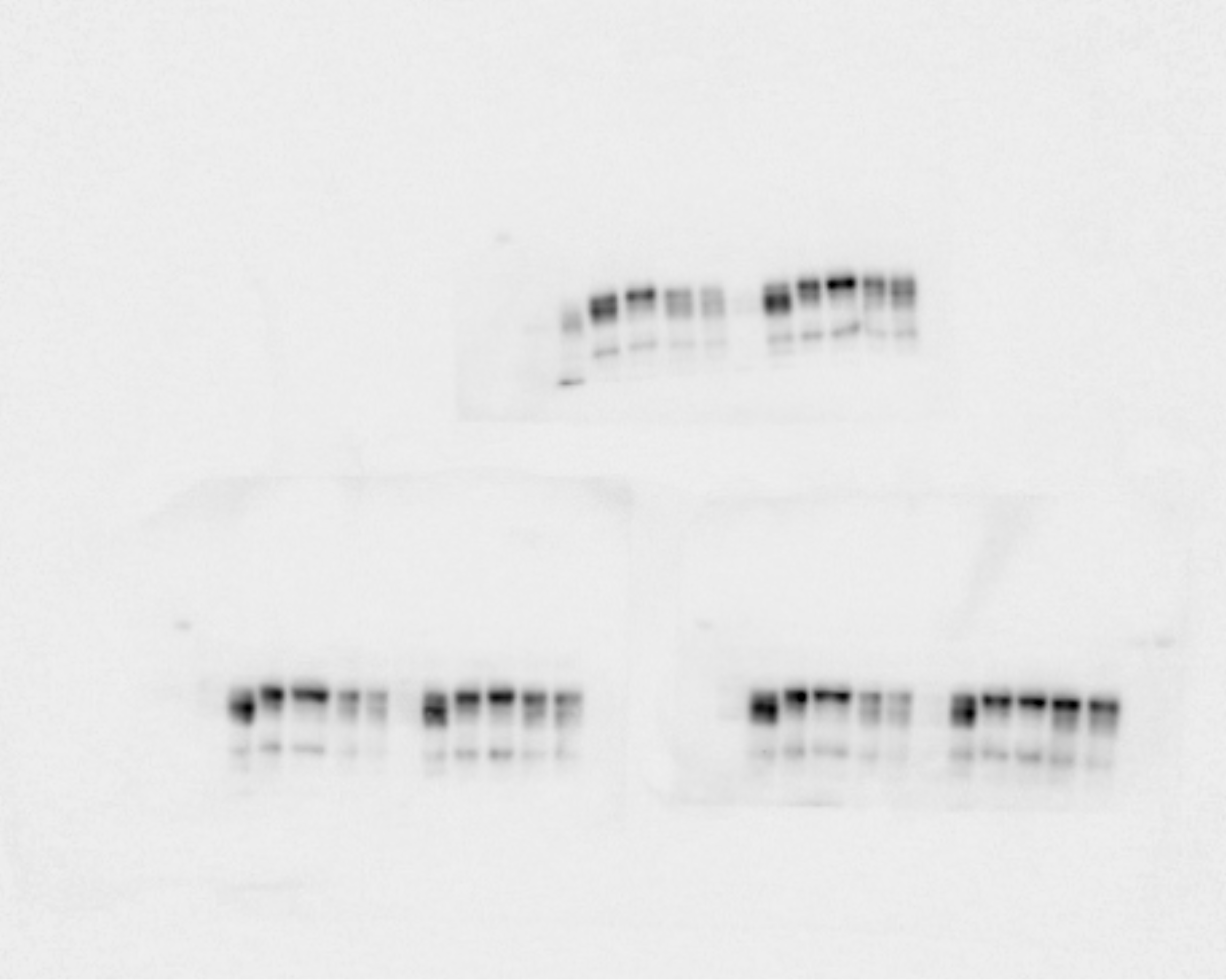

Supplement: Figure 2—source data 3. [file elife-83159-fig2-data3.zip › TTP Figure 2-source data 3/Versteeg 2023-01-26 11h53m39s 4.016s(Chemiluminescence).tif]

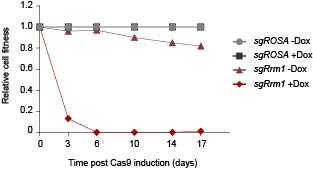

Supplement: Figure 2—source data 4. — RAW264.7-Dox-Cas9 cells expressing an sgRNA targeting the cell-essential gene Rrm1 or the ROSA safe-harbor locus. sgRNA-positive cells were monitored by flow cytometry in the presence or absence of Dox over the indicated time period. Relative cell viability of sgRrm1-transduced cells was compared to untransduced cells, normalized to sgROSA relative cell viability and plotted. [file elife-83159-fig2-data4.zip › Figure 2-source data 4/Figure 2-source data 4.jpg]

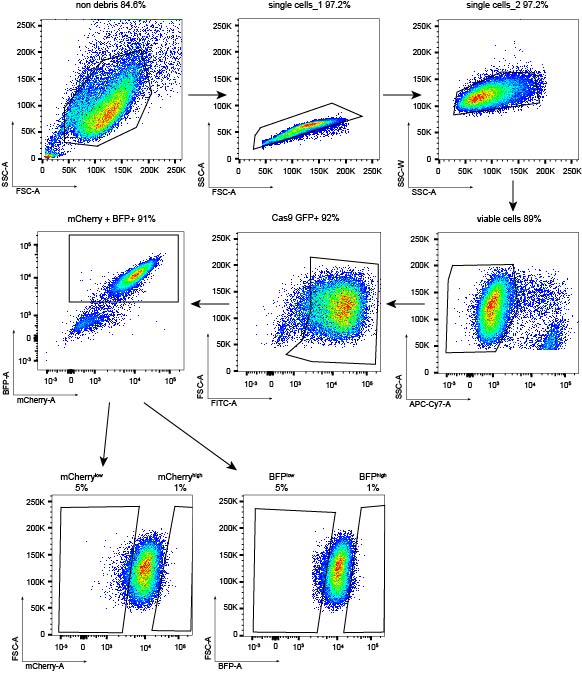

Supplement: Figure 2—source data 5. — Representative scatter plots are shown in hierarchical order. First debris, doublets, dead (APC-Cy7 positive), Cas9-negative (GFP), mCherry- and BFP-negative cells were excluded. 5% of cells with the lowest and 1% of cells with the highest mCherry and BFP signal were sorted. [file elife-83159-fig2-data5.zip › Figure 2-source data 5/Figure 2-source data 5.jpg]

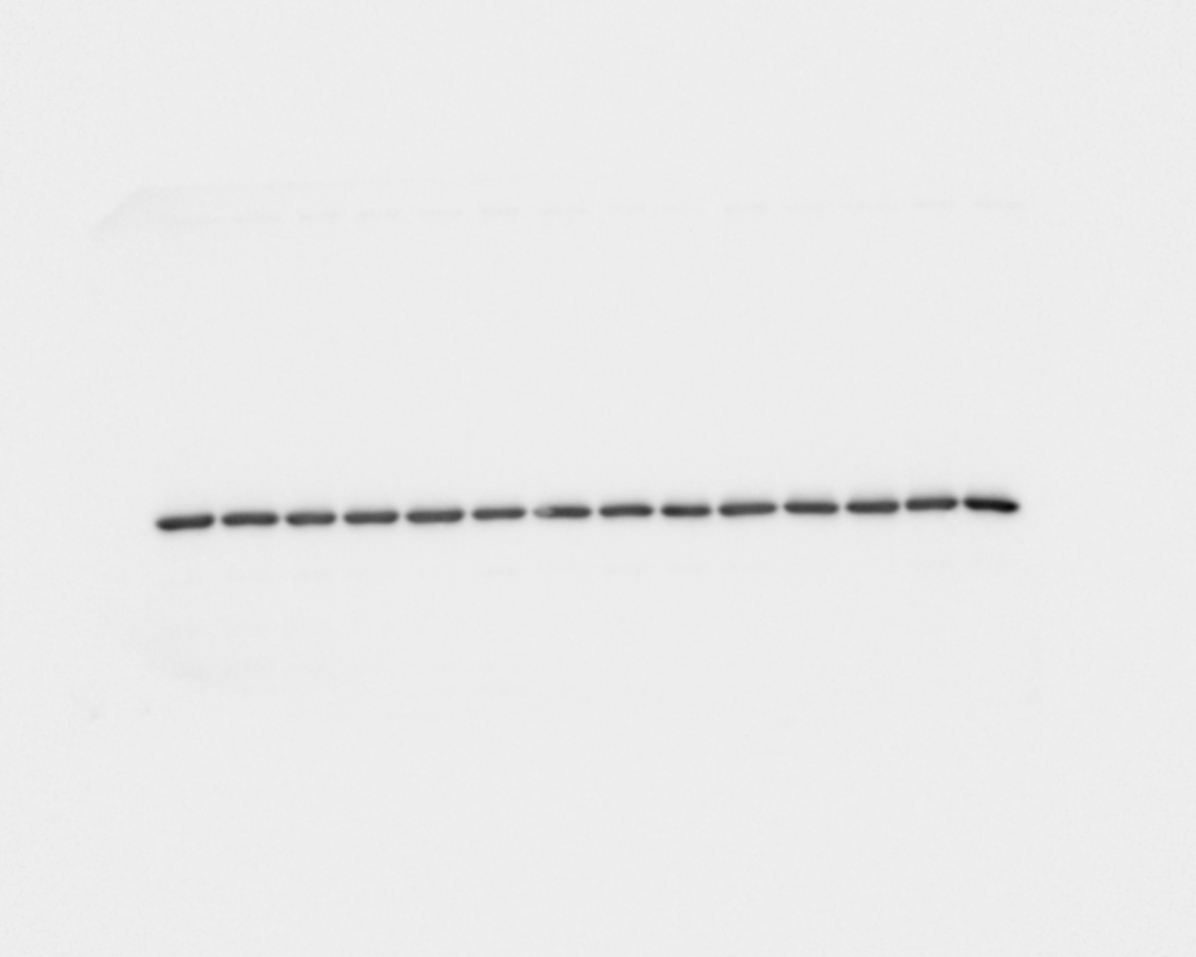

Supplement: Figure 2—figure supplement 1—source data 1. [file elife-83159-fig2-figsupp1-data1.zip › ACTIN Figure 2-figure supplement 1-source data 1/Versteeg 2021-05-03 14h07m04s 7.555s(Chemiluminescence).jpg]

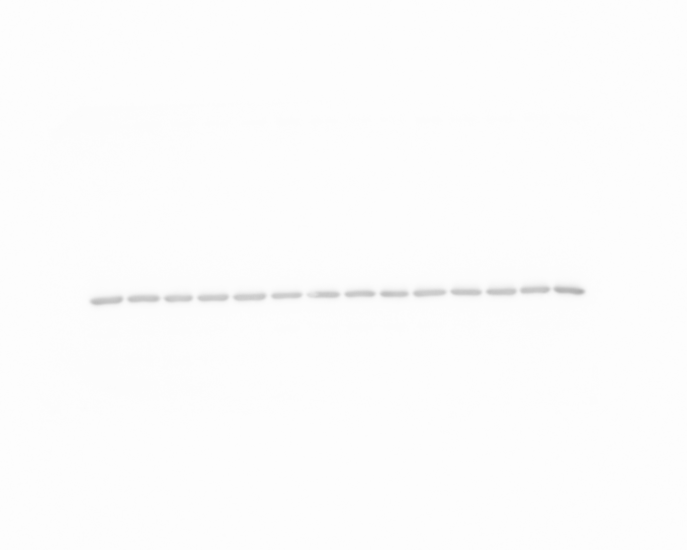

Supplement: Figure 2—figure supplement 1—source data 1. [file elife-83159-fig2-figsupp1-data1.zip › ACTIN Figure 2-figure supplement 1-source data 1/Versteeg 2021-05-03 14h07m04s 7.555s(Chemiluminescence).raw16.tif]

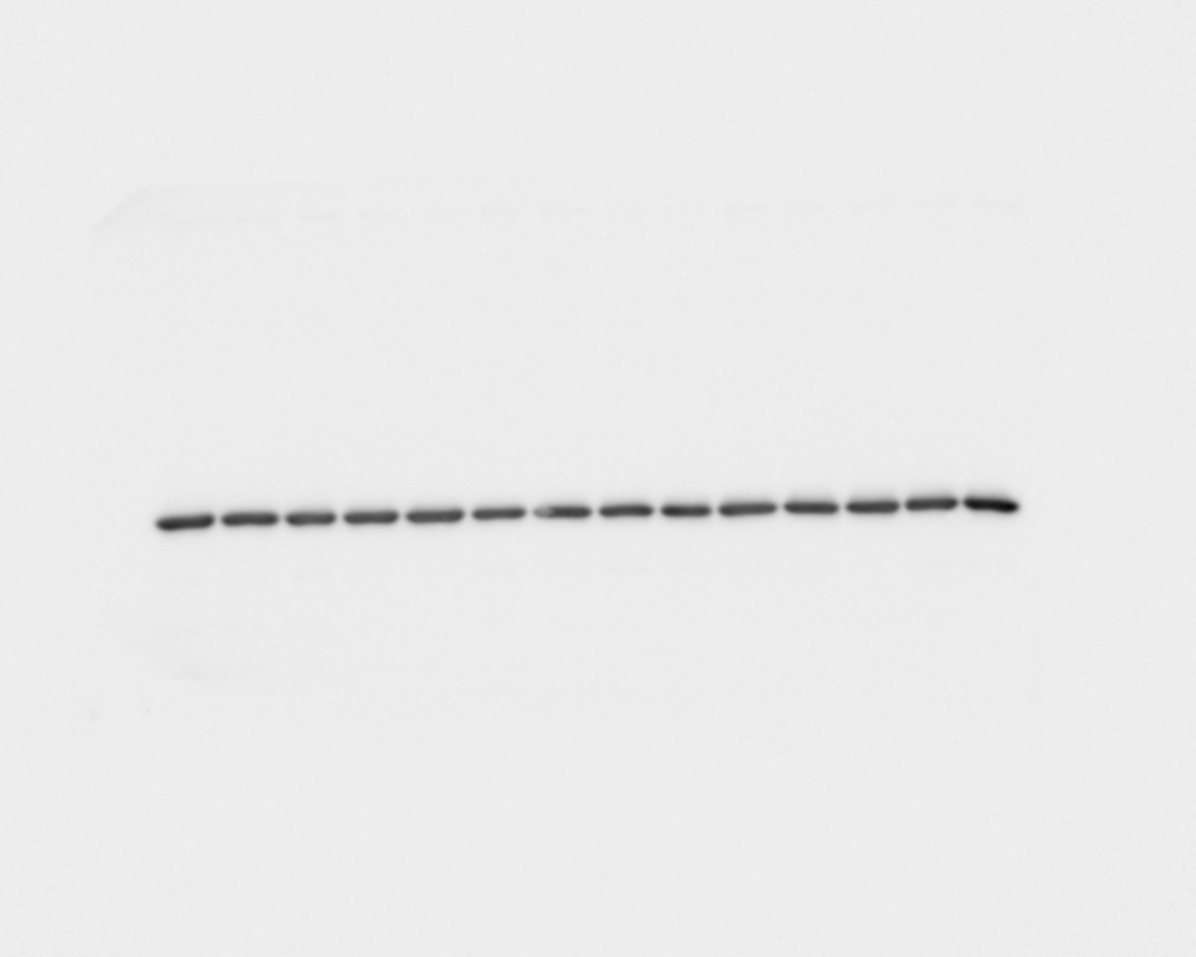

Supplement: Figure 2—figure supplement 1—source data 1. [file elife-83159-fig2-figsupp1-data1.zip › ACTIN Figure 2-figure supplement 1-source data 1/Versteeg 2021-05-03 14h07m04s 7.555s(Chemiluminescence).tif]

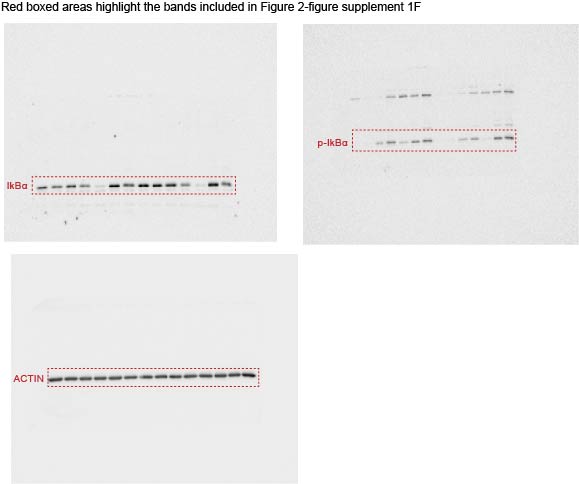

Supplement: Figure 2—figure supplement 1—source data 1. [file elife-83159-fig2-figsupp1-data1.zip › Figure 2-figure supplement 1-source data 1.jpg]

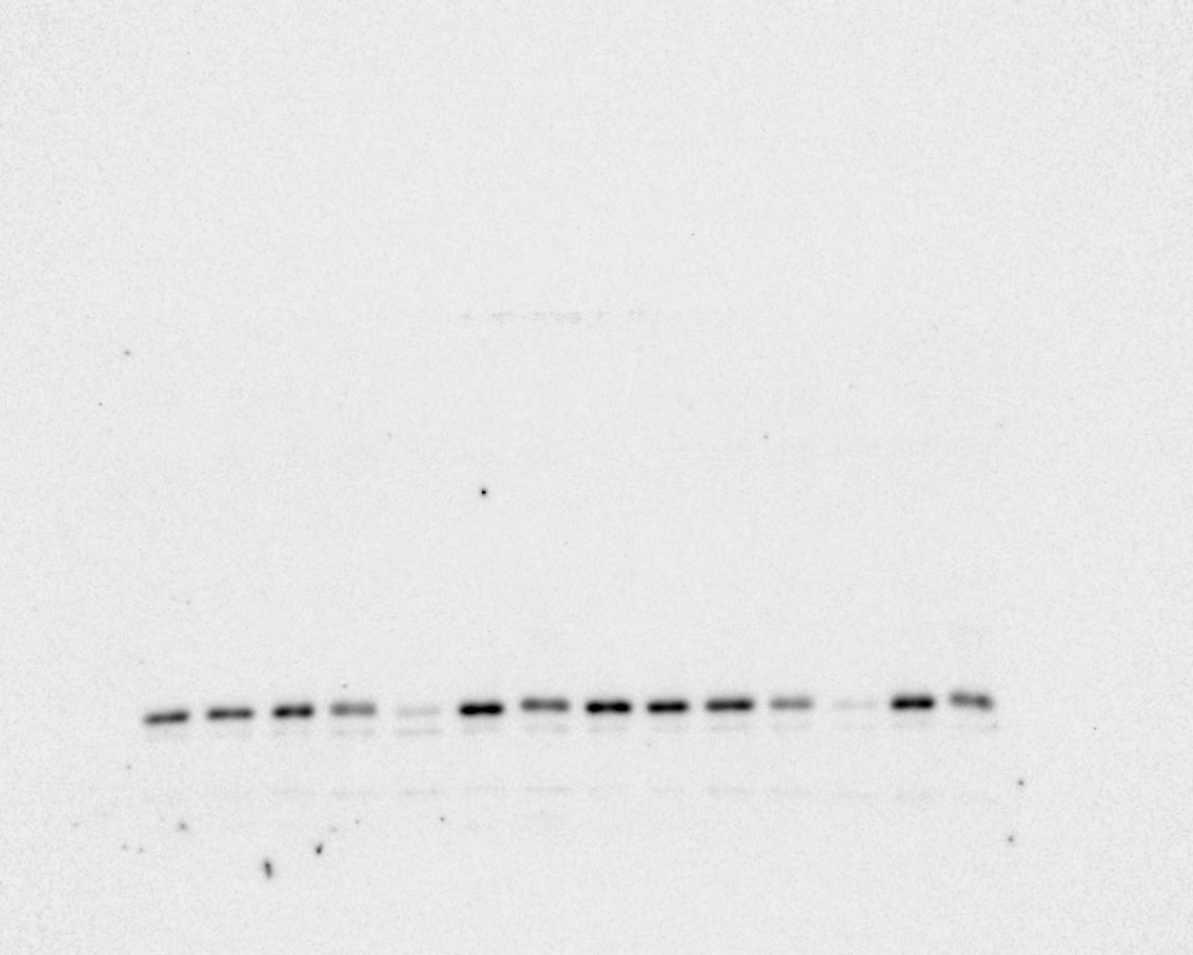

Supplement: Figure 2—figure supplement 1—source data 1. [file elife-83159-fig2-figsupp1-data1.zip › IkBa Figure 2-figure supplement 1-source data 1/Versteeg 2021-05-03 11h45m16s 123.100s(Chemiluminescence).jpg]

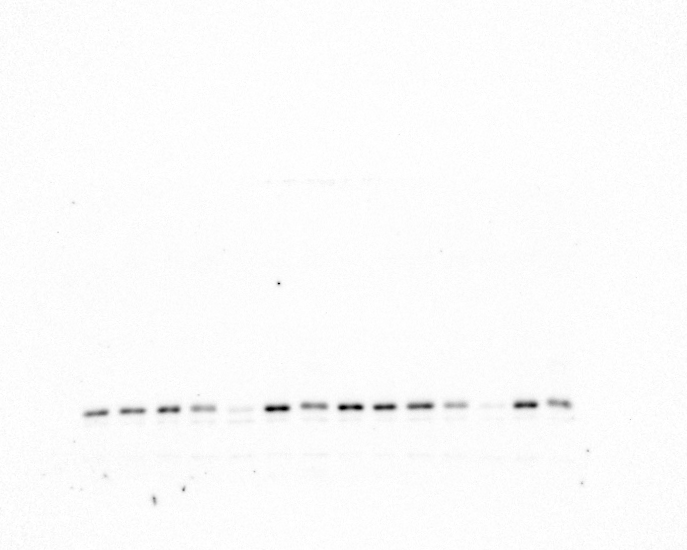

Supplement: Figure 2—figure supplement 1—source data 1. [file elife-83159-fig2-figsupp1-data1.zip › IkBa Figure 2-figure supplement 1-source data 1/Versteeg 2021-05-03 11h45m16s 123.100s(Chemiluminescence).raw16.tif]

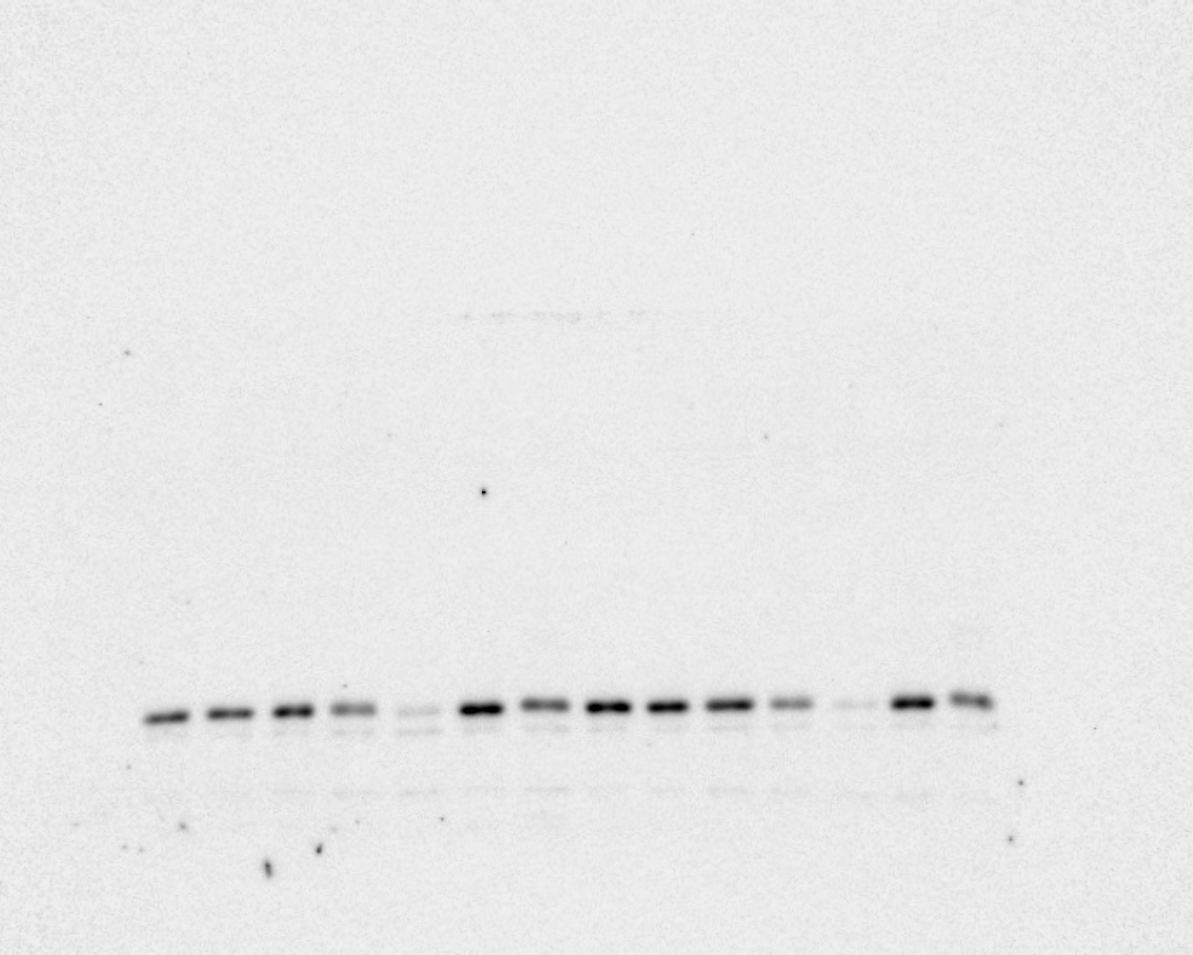

Supplement: Figure 2—figure supplement 1—source data 1. [file elife-83159-fig2-figsupp1-data1.zip › IkBa Figure 2-figure supplement 1-source data 1/Versteeg 2021-05-03 11h45m16s 123.100s(Chemiluminescence).tif]

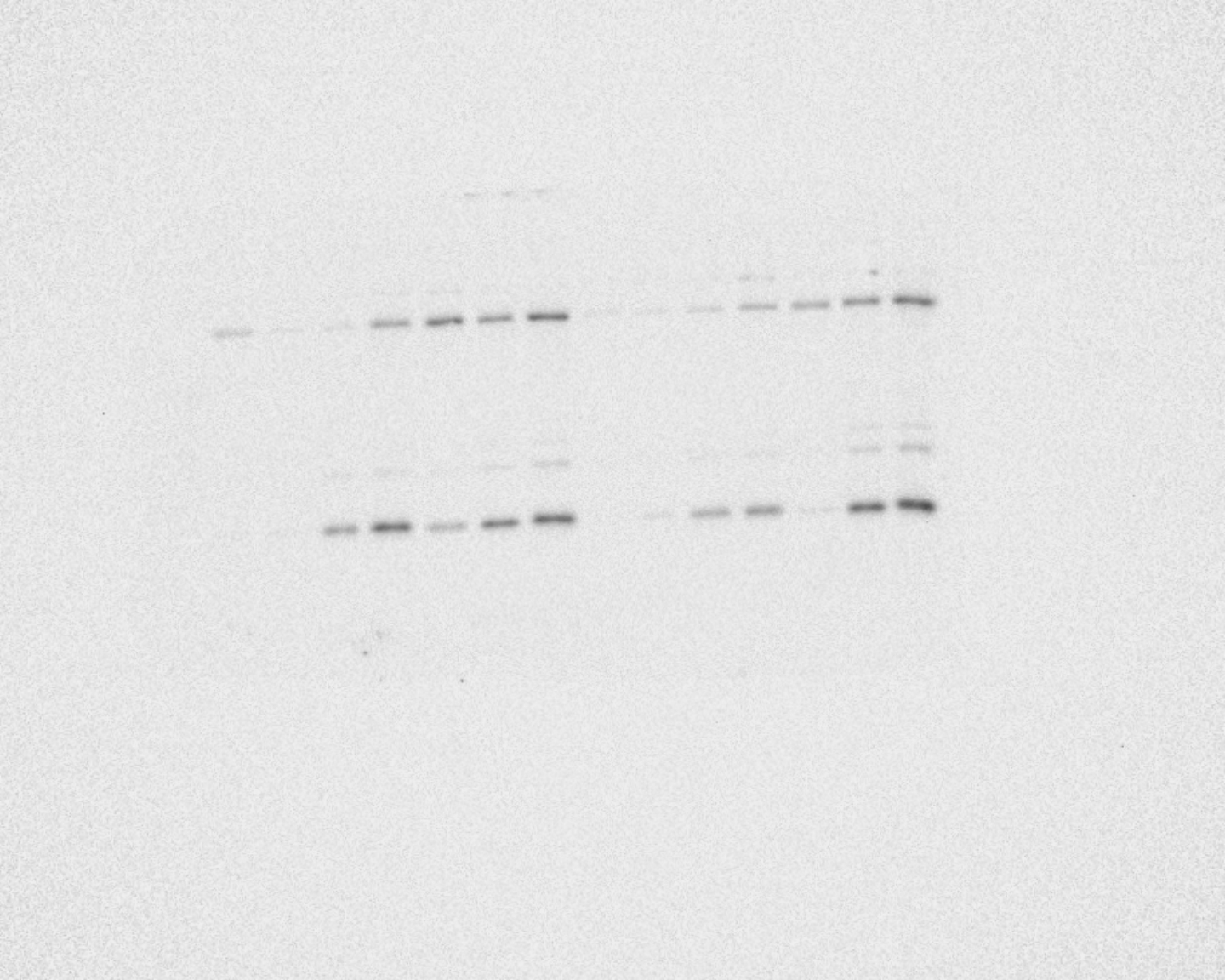

Supplement: Figure 2—figure supplement 1—source data 1. [file elife-83159-fig2-figsupp1-data1.zip › p-IkBa Figure 2-figure supplement 1-source data 1/Versteeg 2021-05-02 15h12m07s 199.992s(Chemiluminescence).jpg]

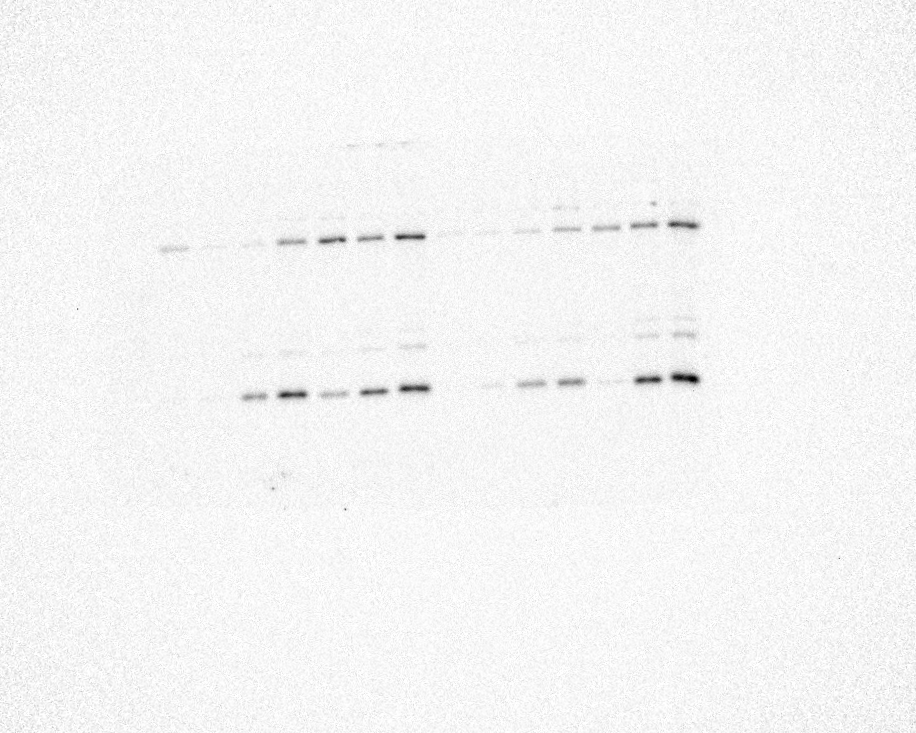

Supplement: Figure 2—figure supplement 1—source data 1. [file elife-83159-fig2-figsupp1-data1.zip › p-IkBa Figure 2-figure supplement 1-source data 1/Versteeg 2021-05-02 15h12m07s 199.992s(Chemiluminescence).raw16.tif]

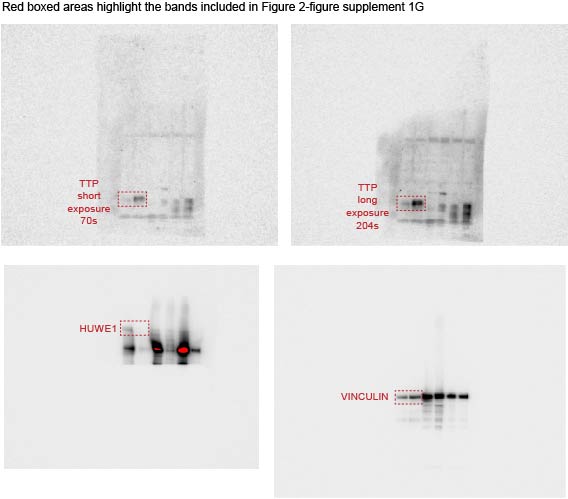

Supplement: Figure 2—figure supplement 1—source data 2. [file elife-83159-fig2-figsupp1-data2.zip › Figure 2-figure supplement 1-source data 2.jpg]

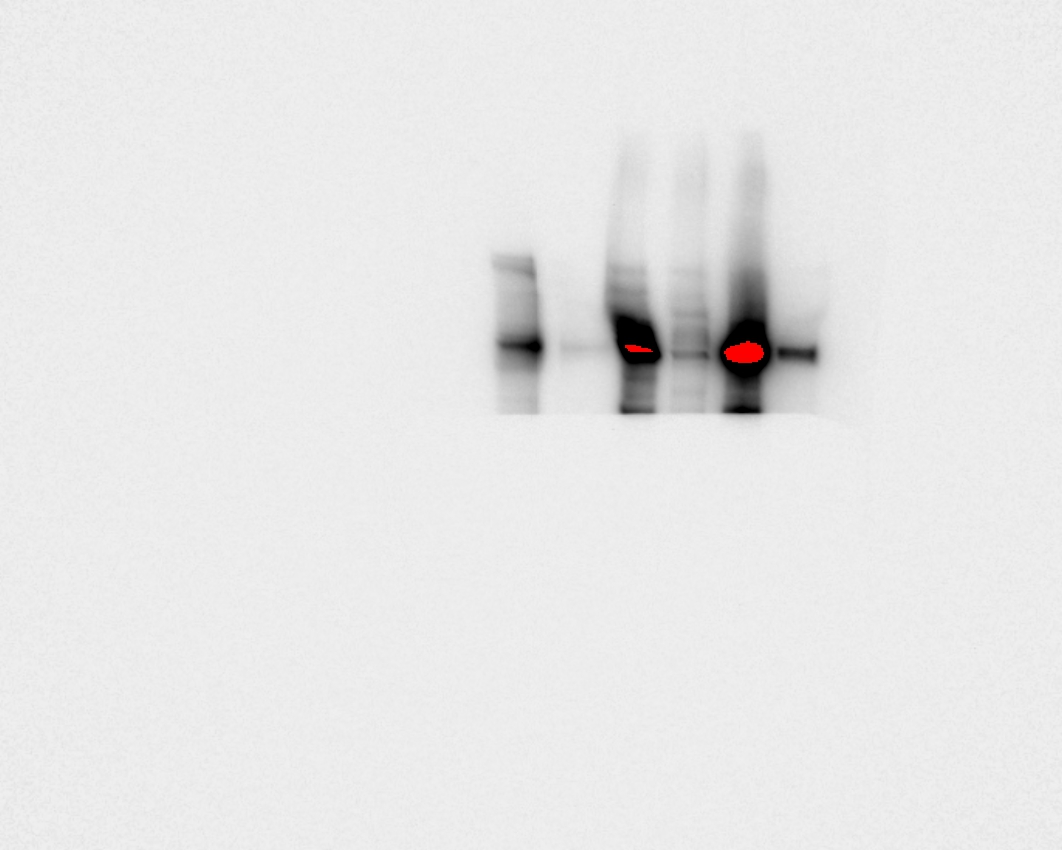

Supplement: Figure 2—figure supplement 1—source data 2. [file elife-83159-fig2-figsupp1-data2.zip › HUWE1 Figure 2-figure supplement 1-source data 2/Versteeg 2021-10-06 09h48m39s 21.150s(Chemiluminescence).jpg]

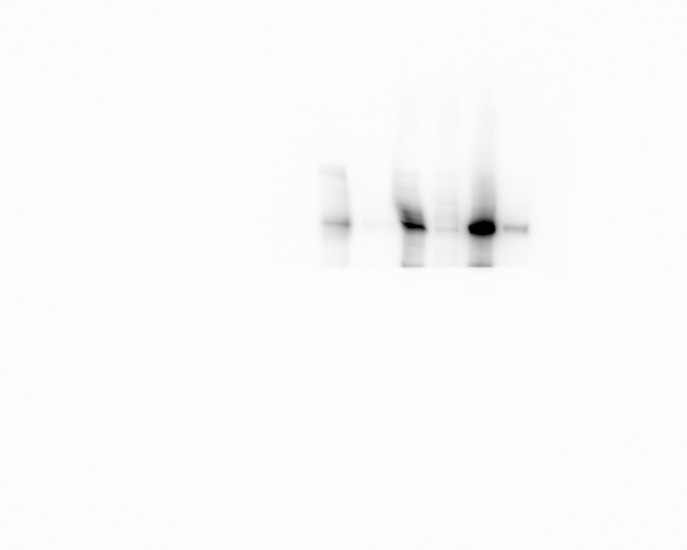

Supplement: Figure 2—figure supplement 1—source data 2. [file elife-83159-fig2-figsupp1-data2.zip › HUWE1 Figure 2-figure supplement 1-source data 2/Versteeg 2021-10-06 09h48m39s 21.150s(Chemiluminescence).raw16.tif]

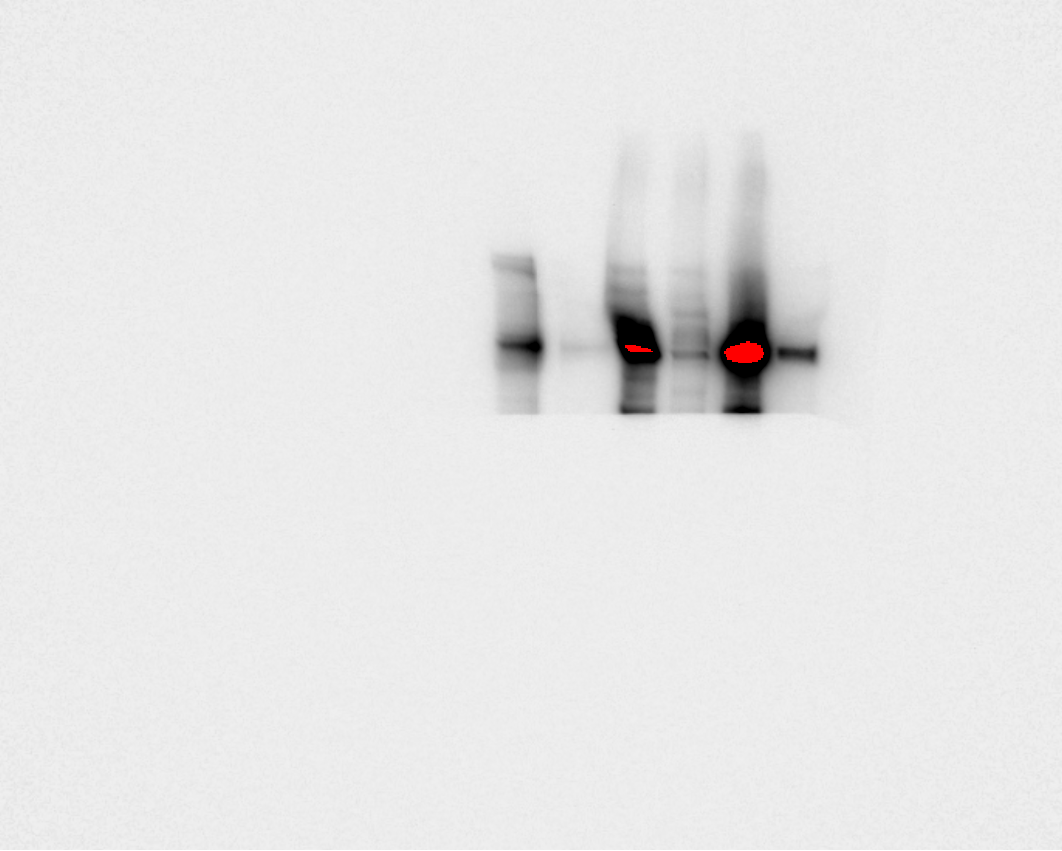

Supplement: Figure 2—figure supplement 1—source data 2. [file elife-83159-fig2-figsupp1-data2.zip › HUWE1 Figure 2-figure supplement 1-source data 2/Versteeg 2021-10-06 09h48m39s 21.150s(Chemiluminescence).tif]

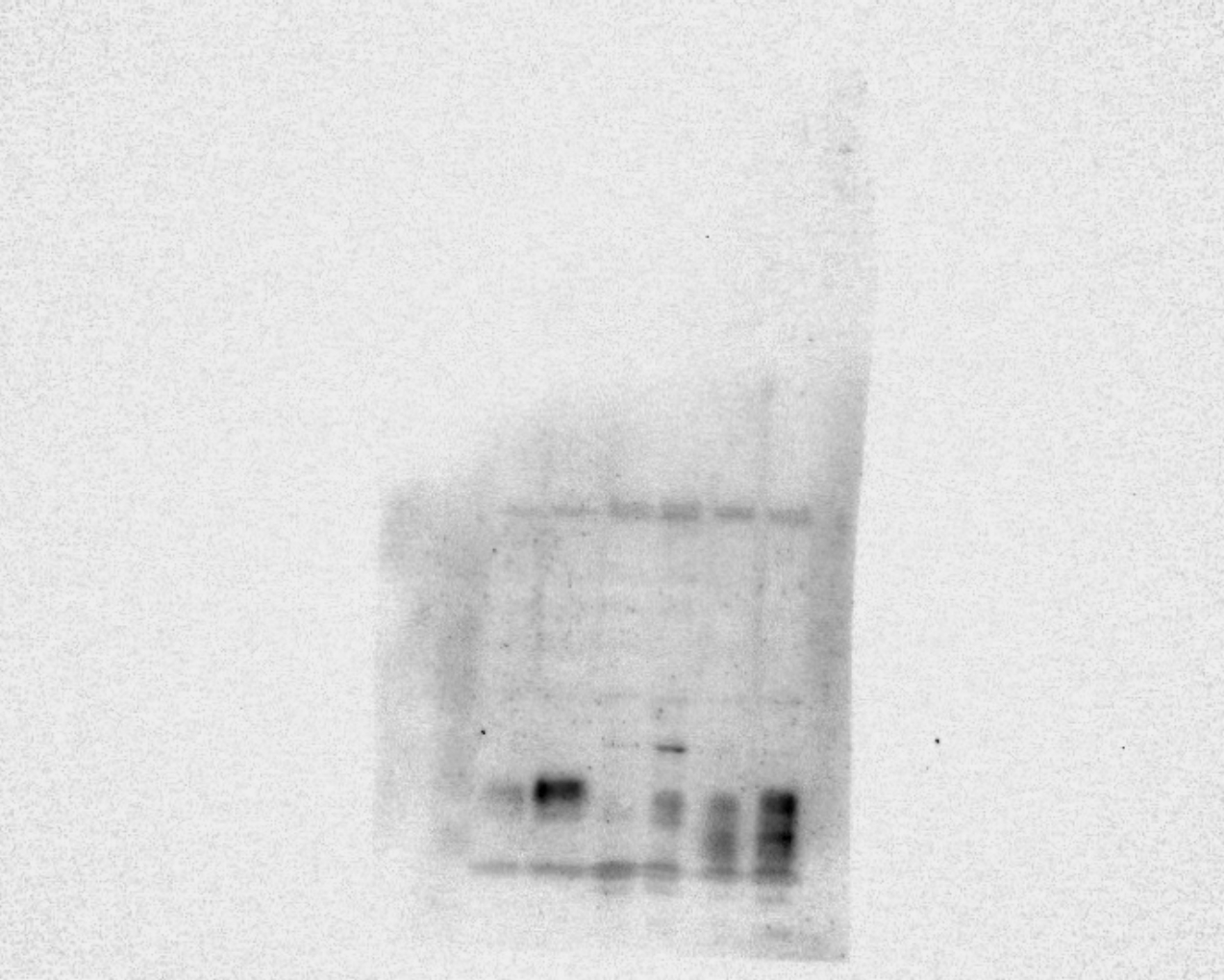

Supplement: Figure 2—figure supplement 1—source data 2. [file elife-83159-fig2-figsupp1-data2.zip › TTP long exp Figure 2-figure supplement 1-source data 2/Versteeg 2021-09-24 09h14m38s 204.550s(Chemiluminescence).jpg]

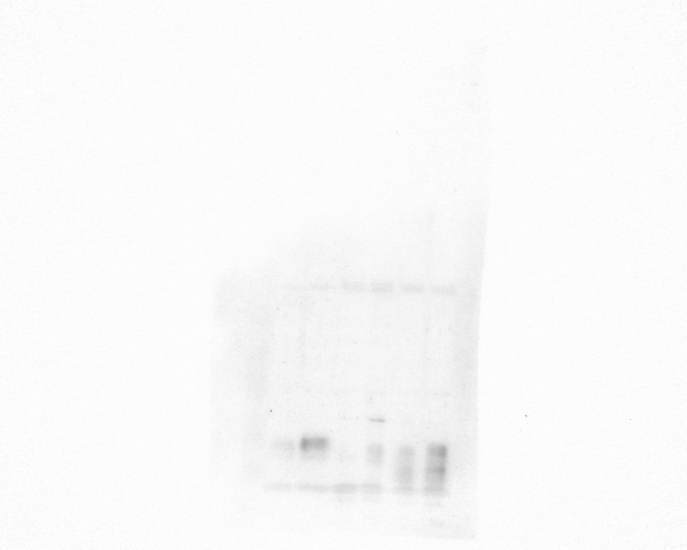

Supplement: Figure 2—figure supplement 1—source data 2. [file elife-83159-fig2-figsupp1-data2.zip › TTP long exp Figure 2-figure supplement 1-source data 2/Versteeg 2021-09-24 09h14m38s 204.550s(Chemiluminescence).raw16.tif]

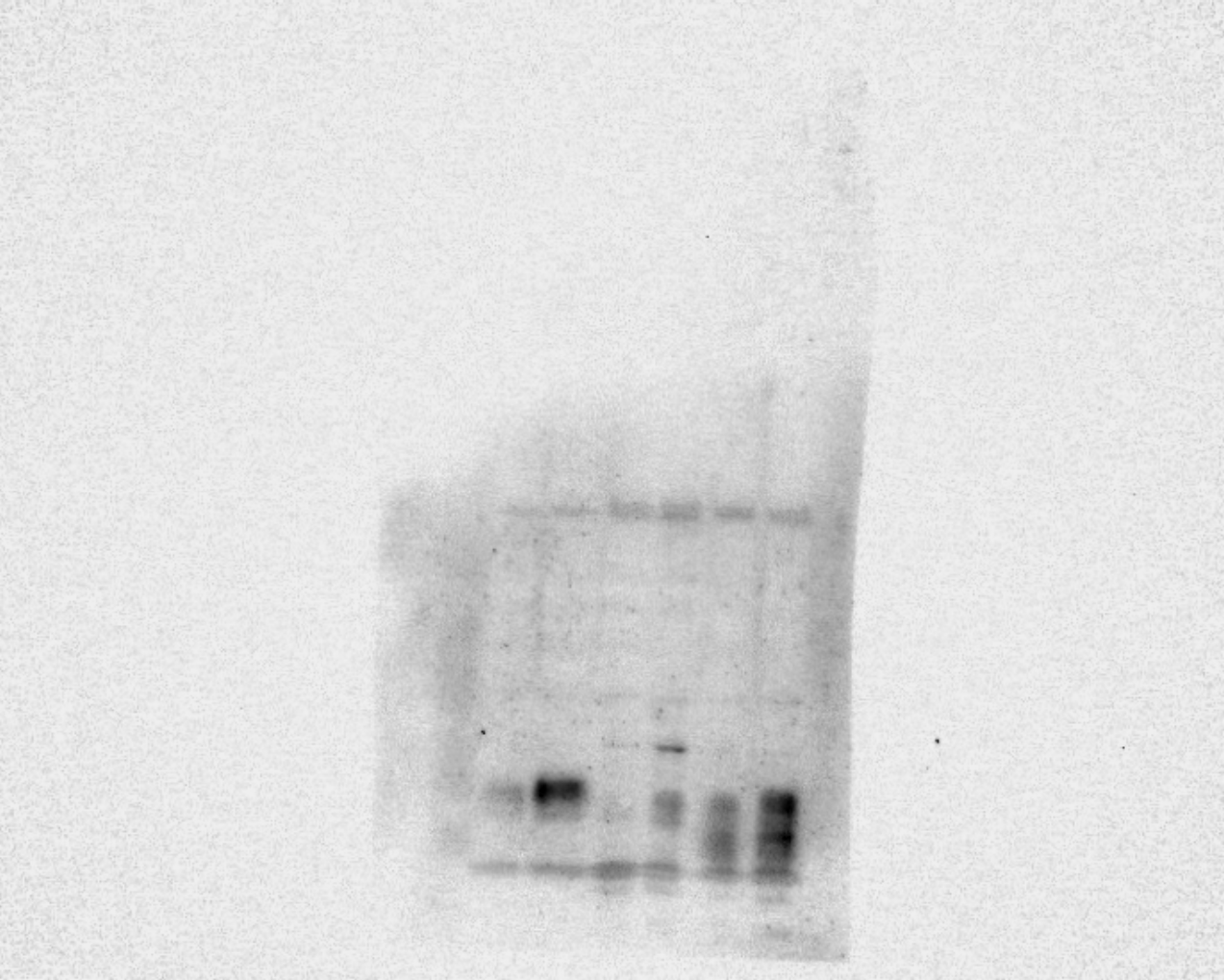

Supplement: Figure 2—figure supplement 1—source data 2. [file elife-83159-fig2-figsupp1-data2.zip › TTP long exp Figure 2-figure supplement 1-source data 2/Versteeg 2021-09-24 09h14m38s 204.550s(Chemiluminescence).tif]

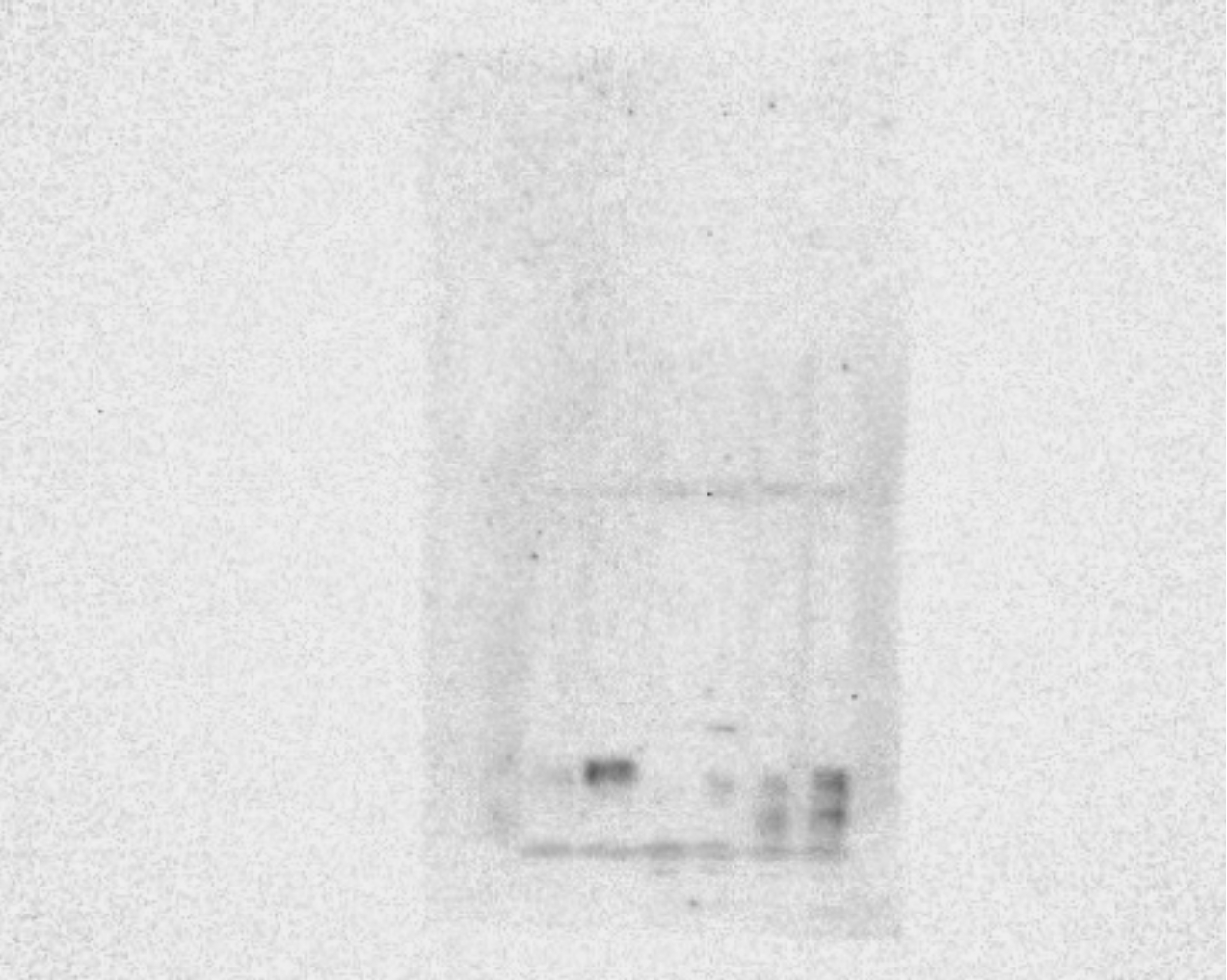

Supplement: Figure 2—figure supplement 1—source data 2. [file elife-83159-fig2-figsupp1-data2.zip › TTP short exp Figure 2-figure supplement 1-source data 2/Versteeg 2021-09-23 16h37m47s 70.460s(Chemiluminescence).jpg]

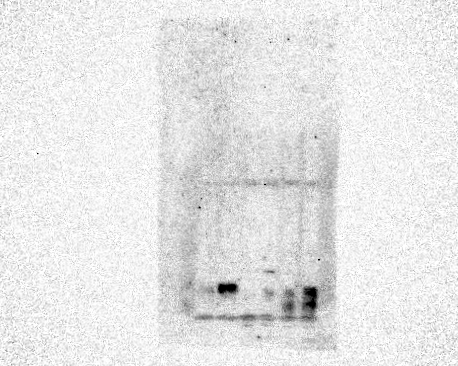

Supplement: Figure 2—figure supplement 1—source data 2. [file elife-83159-fig2-figsupp1-data2.zip › TTP short exp Figure 2-figure supplement 1-source data 2/Versteeg 2021-09-23 16h37m47s 70.460s(Chemiluminescence).raw16.tif]

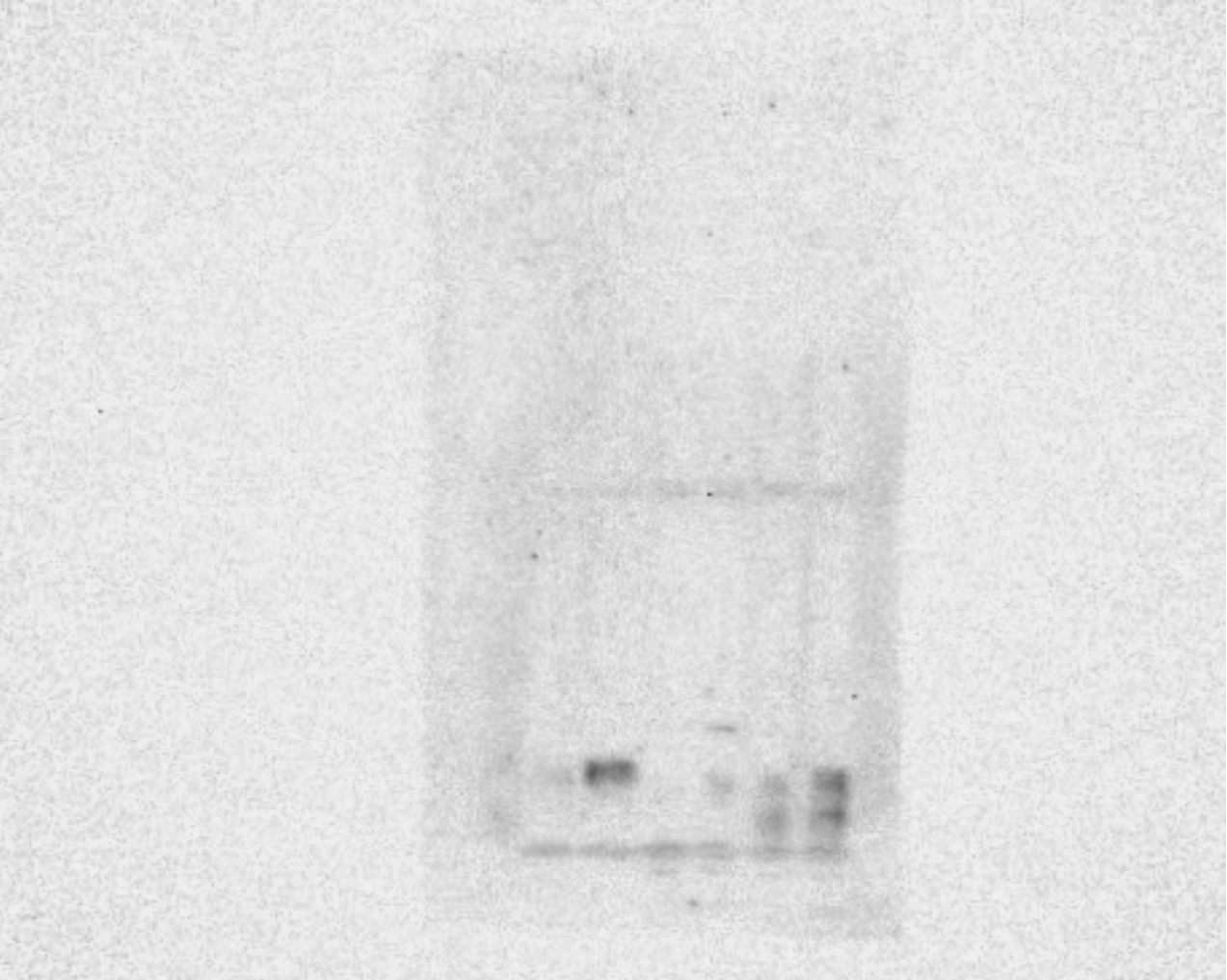

Supplement: Figure 2—figure supplement 1—source data 2. [file elife-83159-fig2-figsupp1-data2.zip › TTP short exp Figure 2-figure supplement 1-source data 2/Versteeg 2021-09-23 16h37m47s 70.460s(Chemiluminescence).tif]

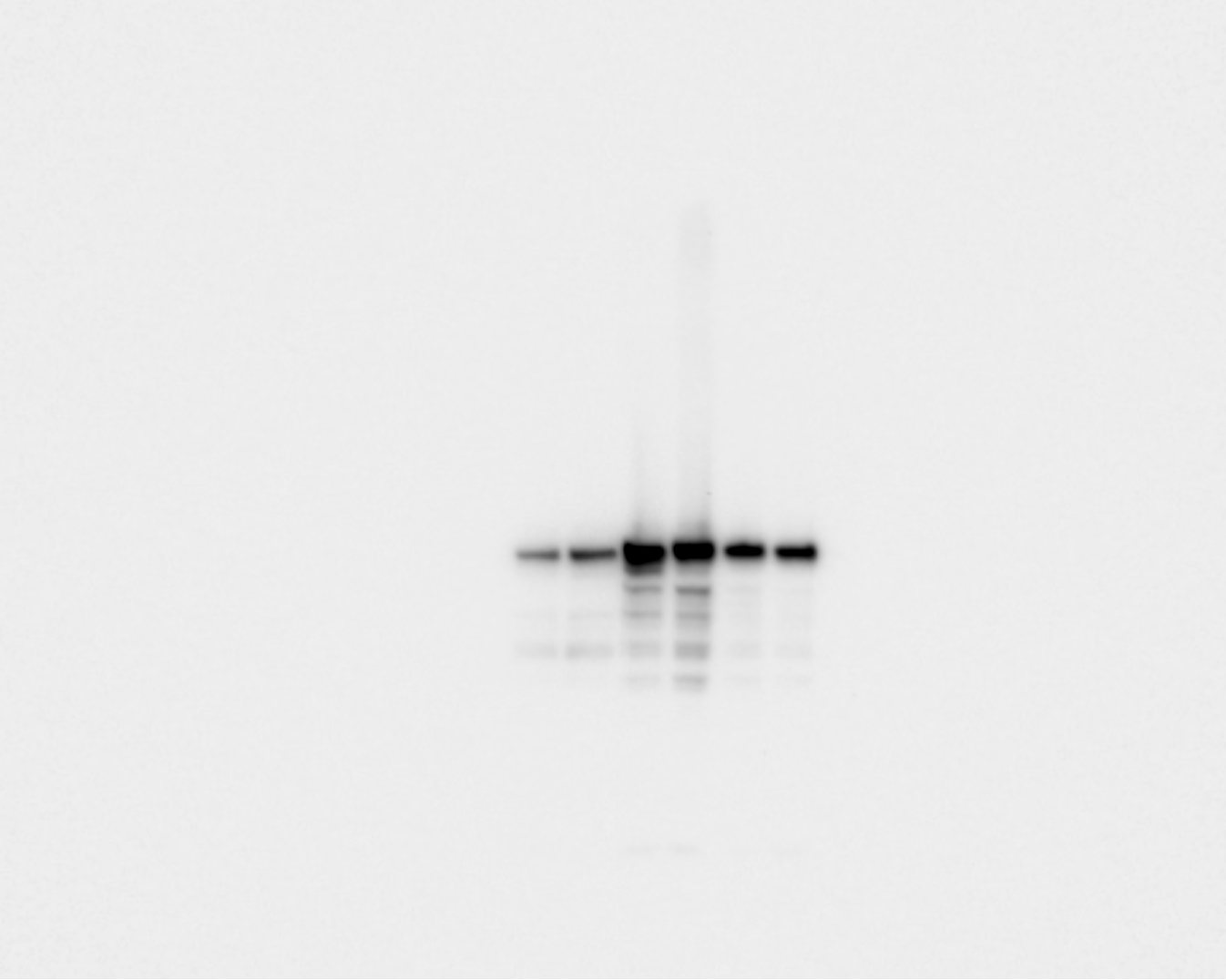

Supplement: Figure 2—figure supplement 1—source data 2. [file elife-83159-fig2-figsupp1-data2.zip › VINCULIN Figure 2-figure supplement 1-source data 2/Versteeg 2021-09-28 17h44m49s 7.102s(Chemiluminescence).jpg]

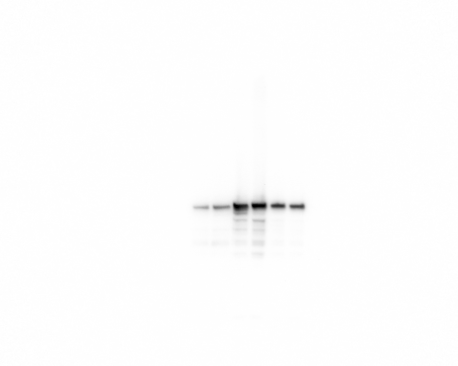

Supplement: Figure 2—figure supplement 1—source data 2. [file elife-83159-fig2-figsupp1-data2.zip › VINCULIN Figure 2-figure supplement 1-source data 2/Versteeg 2021-09-28 17h44m49s 7.102s(Chemiluminescence).raw16.tif]

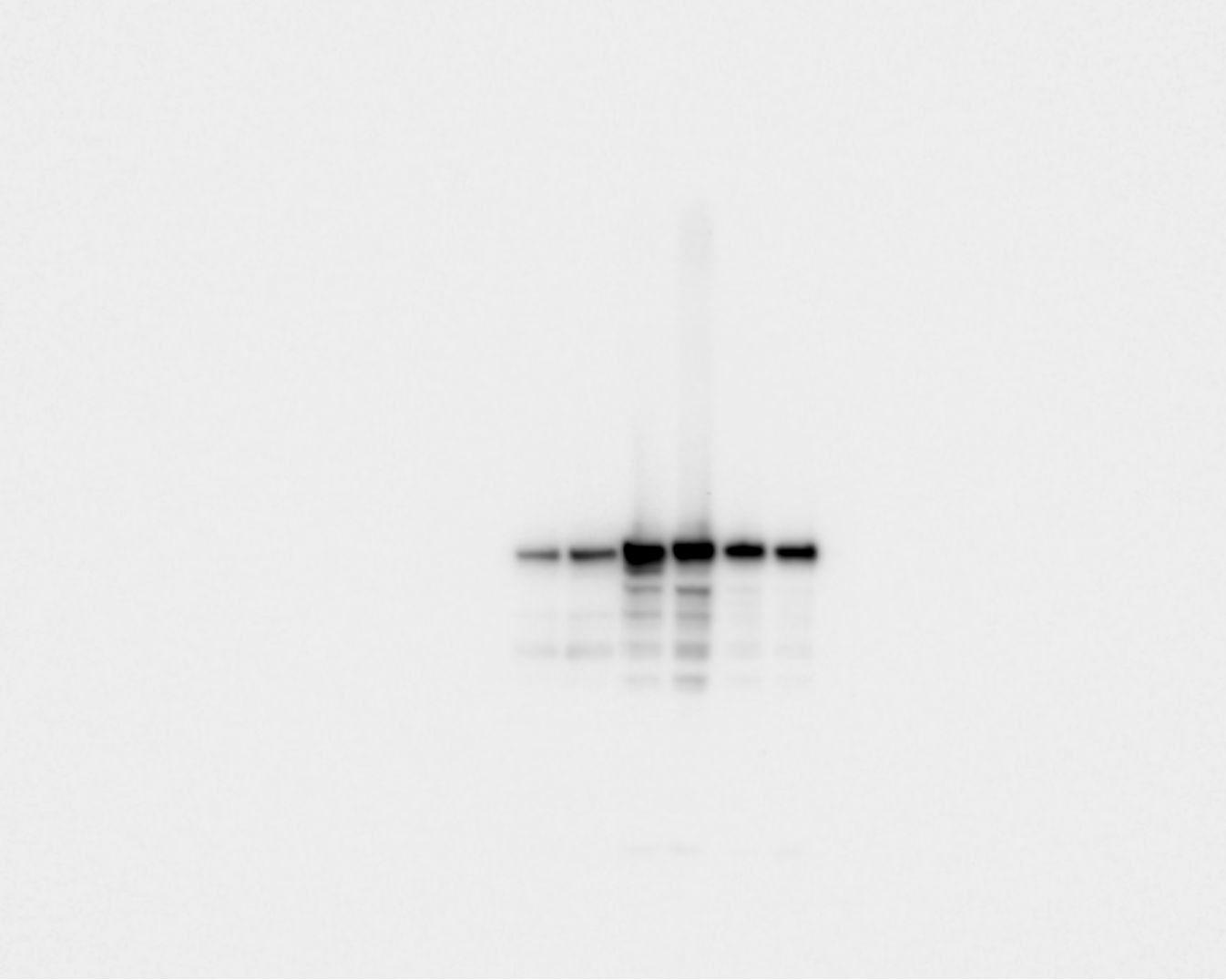

Supplement: Figure 2—figure supplement 1—source data 2. [file elife-83159-fig2-figsupp1-data2.zip › VINCULIN Figure 2-figure supplement 1-source data 2/Versteeg 2021-09-28 17h44m49s 7.102s(Chemiluminescence).tif]

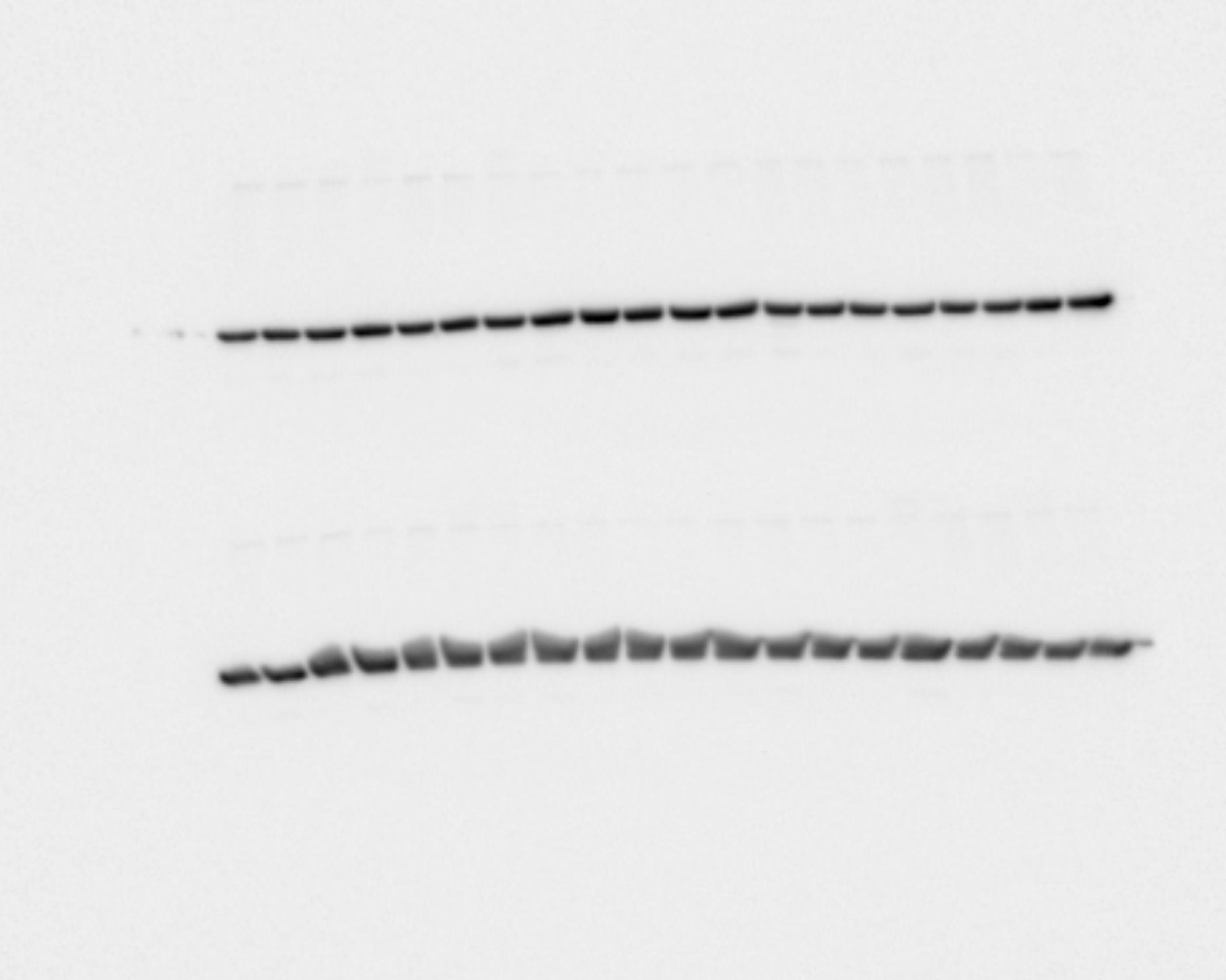

Supplement: Figure 2—figure supplement 1—source data 3. [file elife-83159-fig2-figsupp1-data3.zip › ACTIN Figure 2-figure supplement 1- source data 3/Versteeg 2021-08-13 18h02m26s 5.103s(Chemiluminescence).jpg]

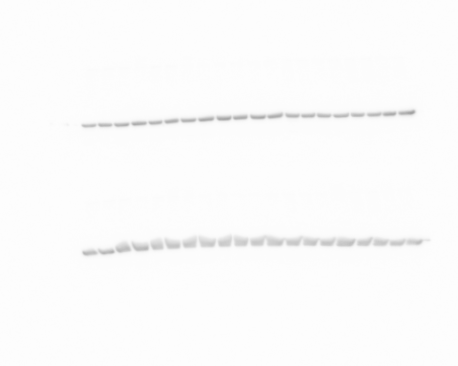

Supplement: Figure 2—figure supplement 1—source data 3. [file elife-83159-fig2-figsupp1-data3.zip › ACTIN Figure 2-figure supplement 1- source data 3/Versteeg 2021-08-13 18h02m26s 5.103s(Chemiluminescence).raw16.tif]

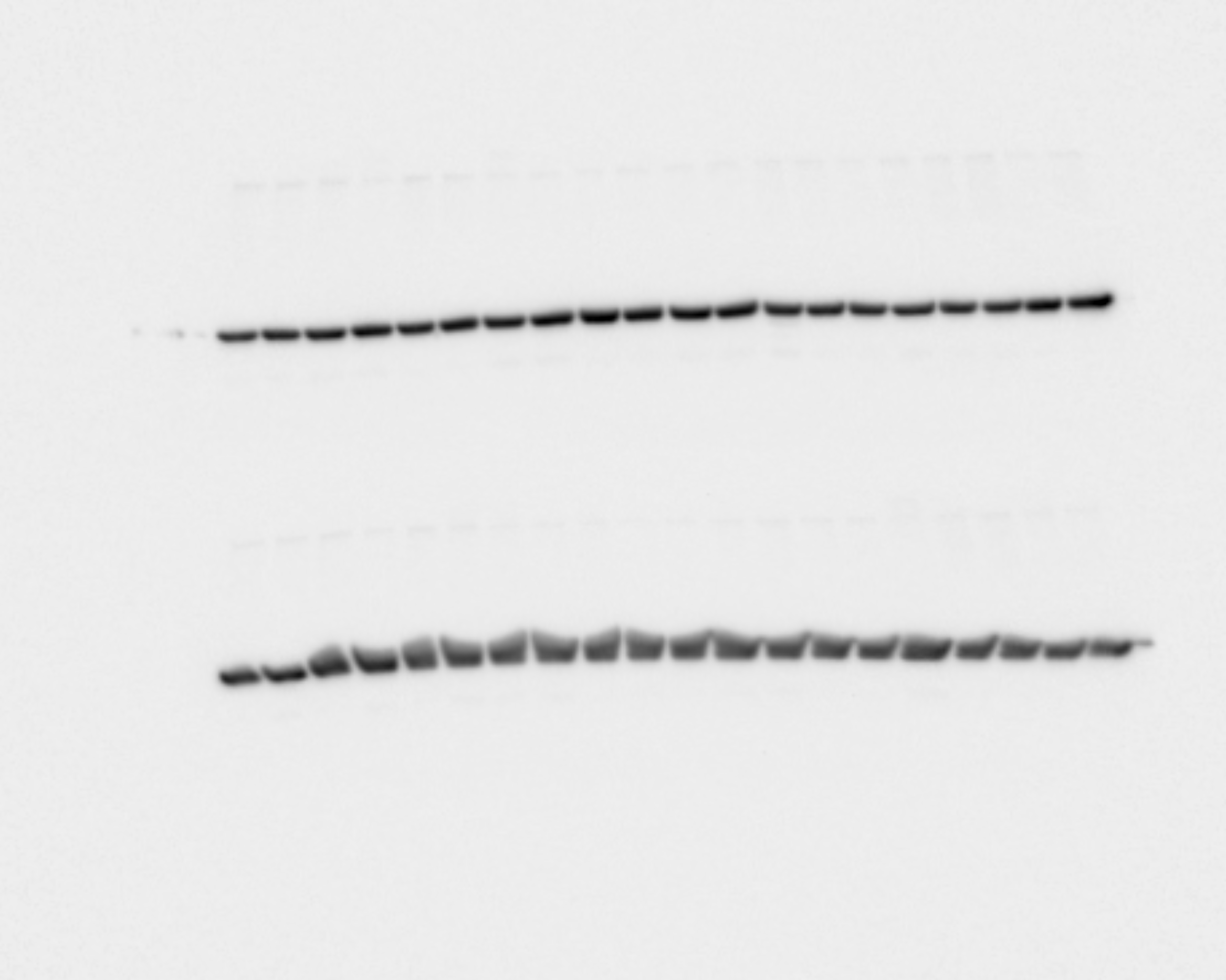

Supplement: Figure 2—figure supplement 1—source data 3. [file elife-83159-fig2-figsupp1-data3.zip › ACTIN Figure 2-figure supplement 1- source data 3/Versteeg 2021-08-13 18h02m26s 5.103s(Chemiluminescence).tif]

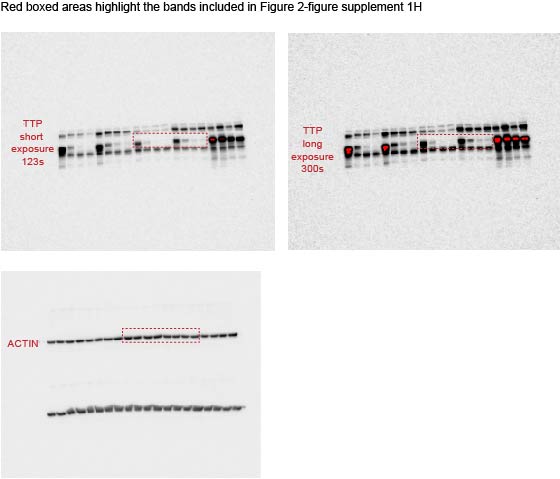

Supplement: Figure 2—figure supplement 1—source data 3. [file elife-83159-fig2-figsupp1-data3.zip › Figure 2-figure supplement 1- source data 3.jpg]

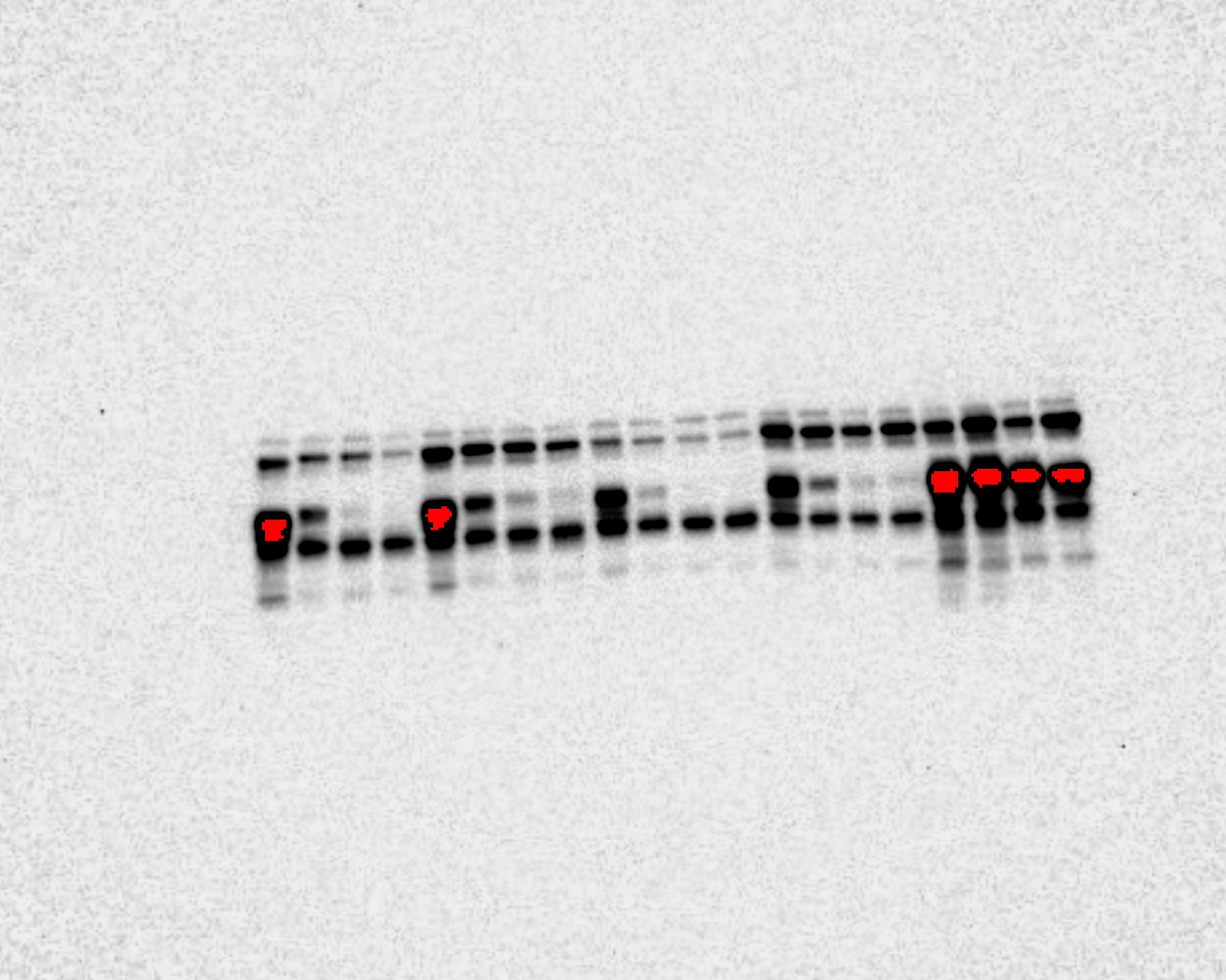

Supplement: Figure 2—figure supplement 1—source data 3. [file elife-83159-fig2-figsupp1-data3.zip › HA-TTP long exp Figure 2-figure supplement 1- source data 3/Versteeg 2021-08-13 11h24m21s 299.983s(Chemiluminescence).jpg]

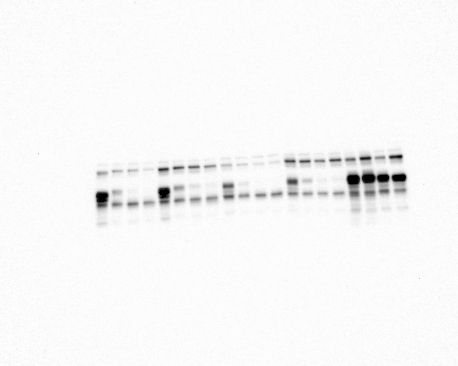

Supplement: Figure 2—figure supplement 1—source data 3. [file elife-83159-fig2-figsupp1-data3.zip › HA-TTP long exp Figure 2-figure supplement 1- source data 3/Versteeg 2021-08-13 11h24m21s 299.983s(Chemiluminescence).raw16.tif]

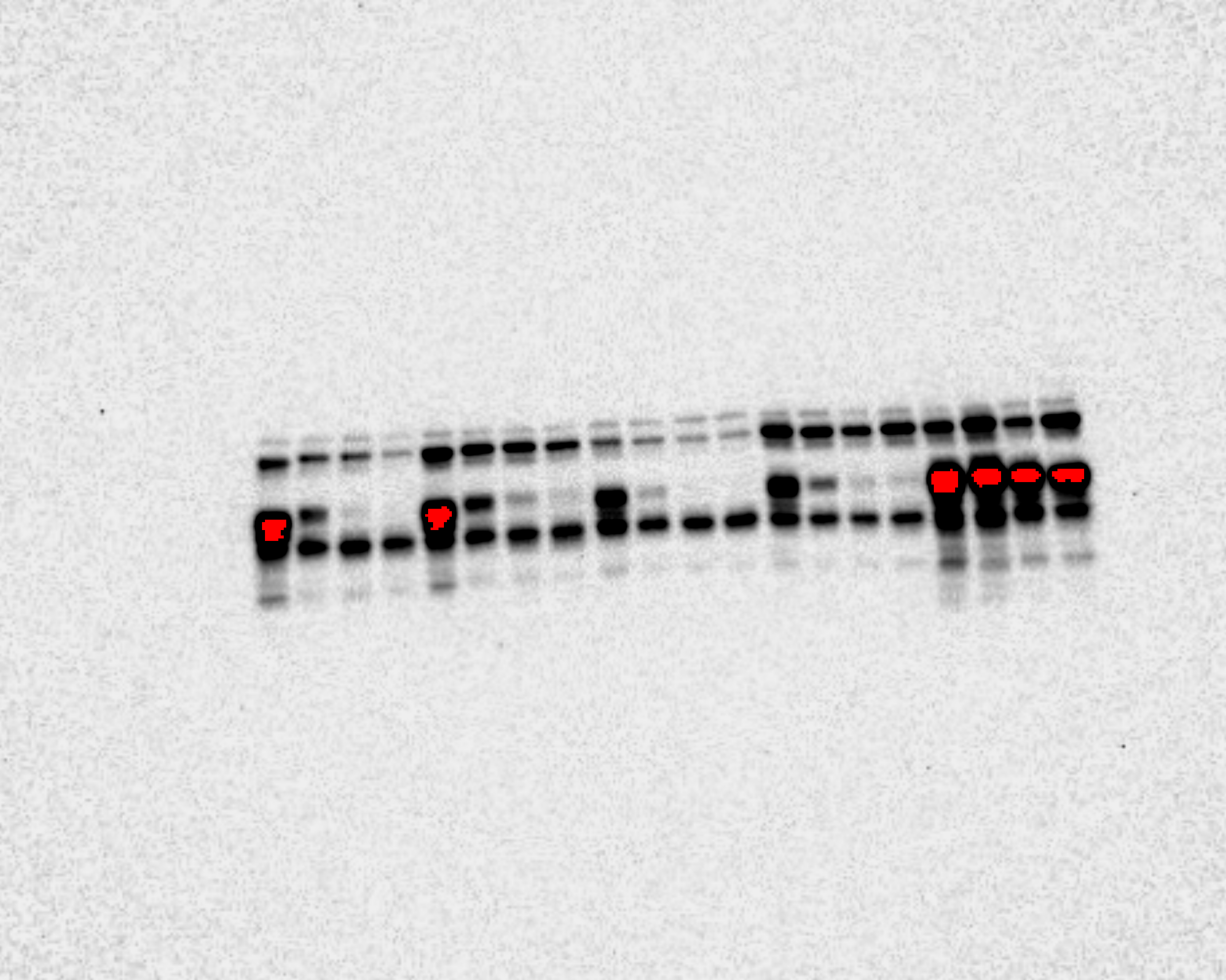

Supplement: Figure 2—figure supplement 1—source data 3. [file elife-83159-fig2-figsupp1-data3.zip › HA-TTP long exp Figure 2-figure supplement 1- source data 3/Versteeg 2021-08-13 11h24m21s 299.983s(Chemiluminescence).tif]
