# Supplementary figures and images for: HUWE1 controls tristetraprolin proteasomal degradation by regulating its phosphorylation (part 3 of 4)
Source: eLife. 2023 Mar 24;12:e83159. doi: 10.7554/eLife.83159 (PMC10038661; doi:10.7554/eLife.83159)

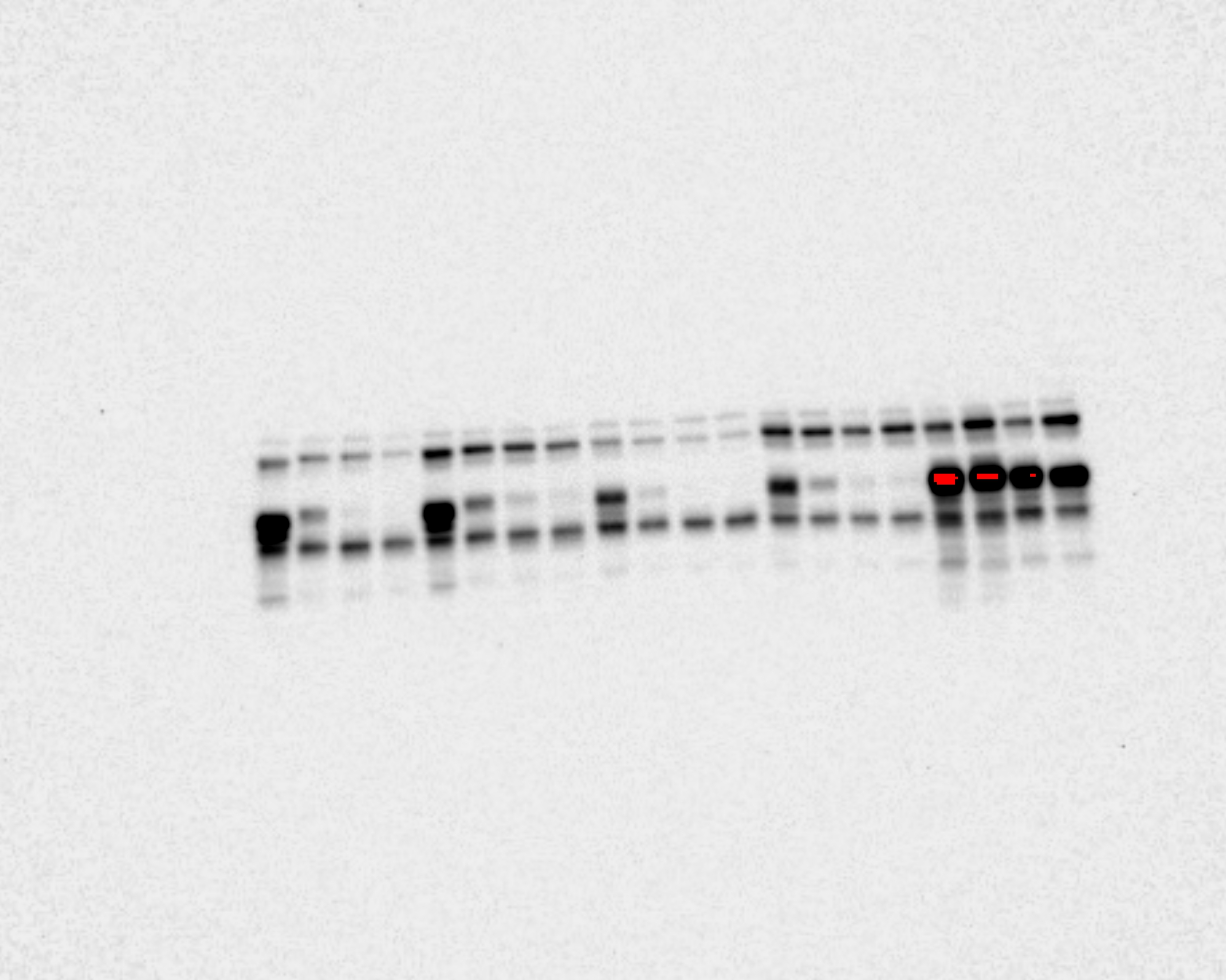

Supplement: Figure 2—figure supplement 1—source data 3. [file elife-83159-fig2-figsupp1-data3.zip › HA-TTP short exp Figure 2-figure supplement 1- source data 3/Versteeg 2021-08-13 11h21m15s 123.097s(Chemiluminescence).jpg]

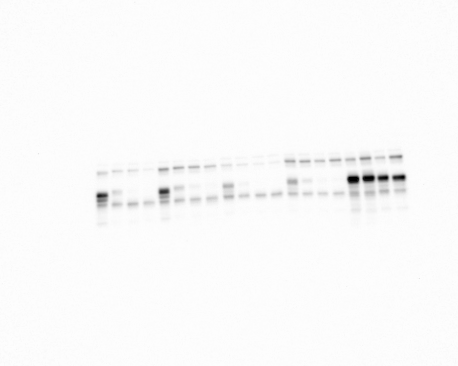

Supplement: Figure 2—figure supplement 1—source data 3. [file elife-83159-fig2-figsupp1-data3.zip › HA-TTP short exp Figure 2-figure supplement 1- source data 3/Versteeg 2021-08-13 11h21m15s 123.097s(Chemiluminescence).raw16.tif]

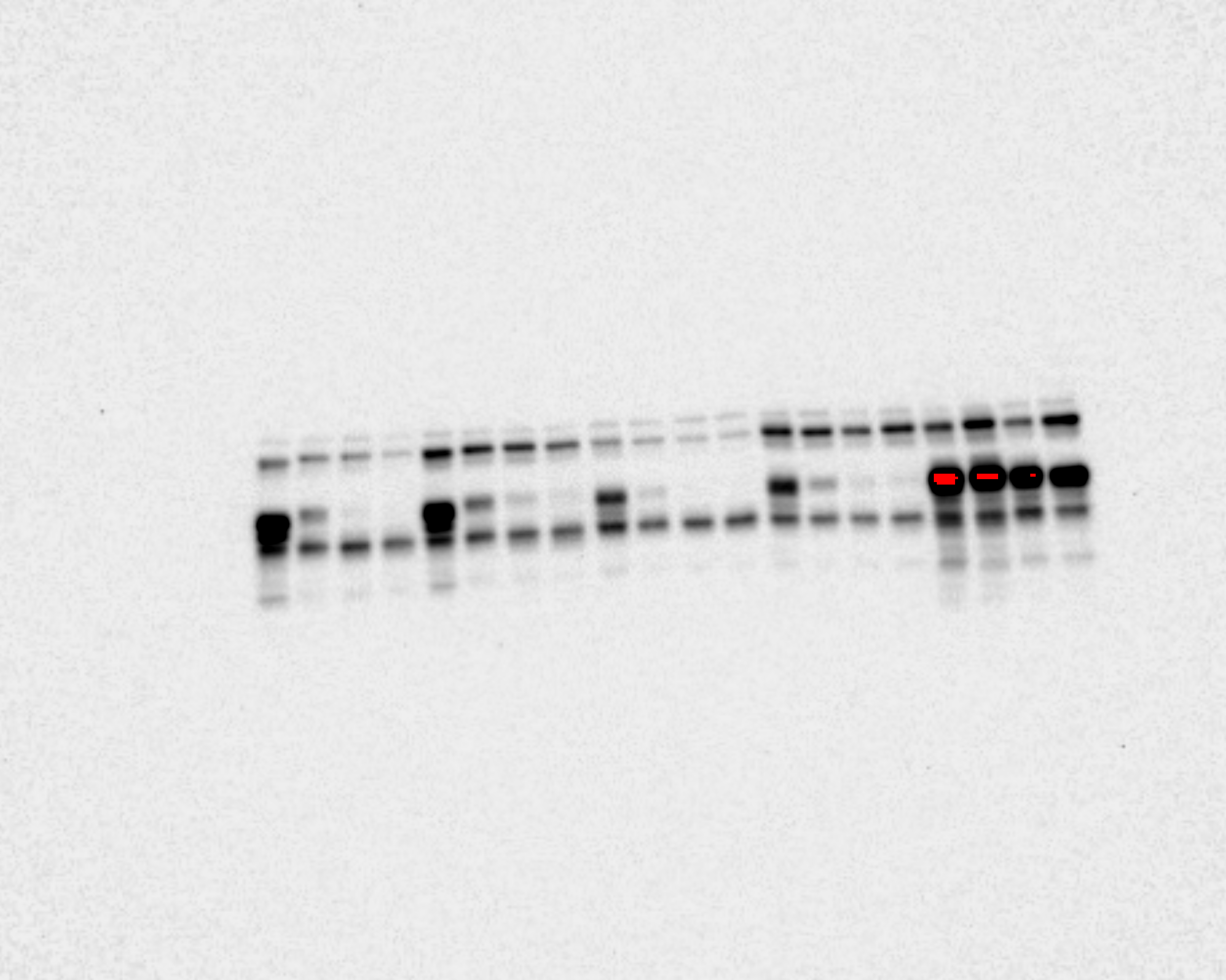

Supplement: Figure 2—figure supplement 1—source data 3. [file elife-83159-fig2-figsupp1-data3.zip › HA-TTP short exp Figure 2-figure supplement 1- source data 3/Versteeg 2021-08-13 11h21m15s 123.097s(Chemiluminescence).tif]

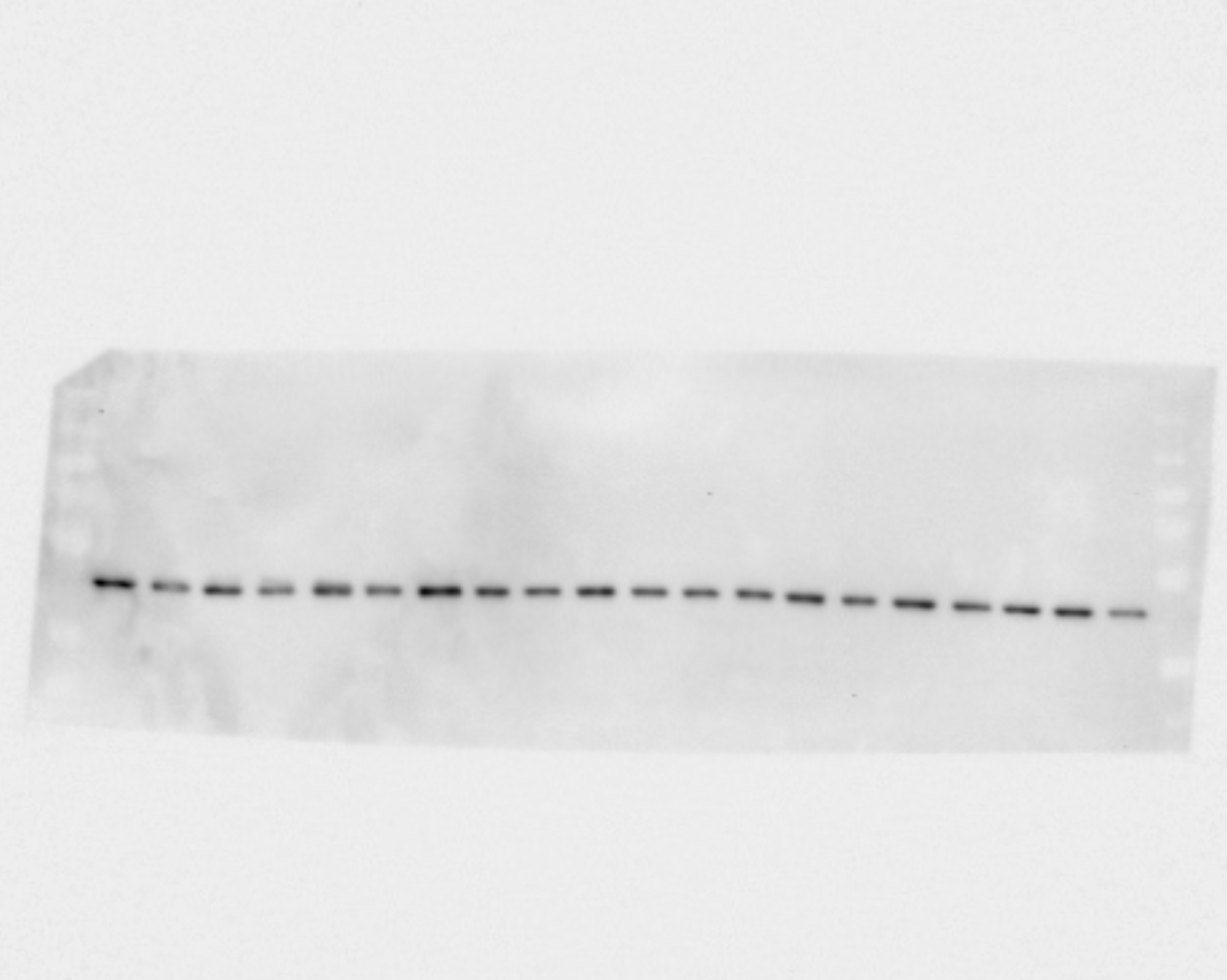

Supplement: Figure 4—source data 1. [file elife-83159-fig4-data1.zip › Figure 4-source data 1/ACTIN Figure 4-source data 1/Versteeg 2021-08-10 14h42m35s 34.654s(Chemiluminescence).jpg]

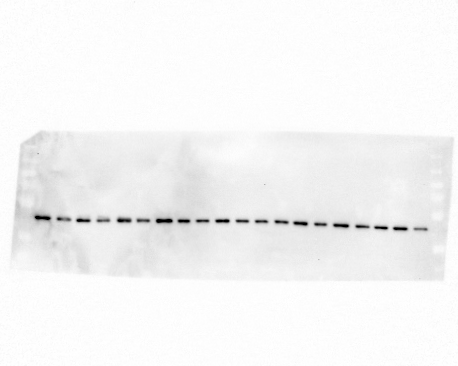

Supplement: Figure 4—source data 1. [file elife-83159-fig4-data1.zip › Figure 4-source data 1/ACTIN Figure 4-source data 1/Versteeg 2021-08-10 14h42m35s 34.654s(Chemiluminescence).raw16.tif]

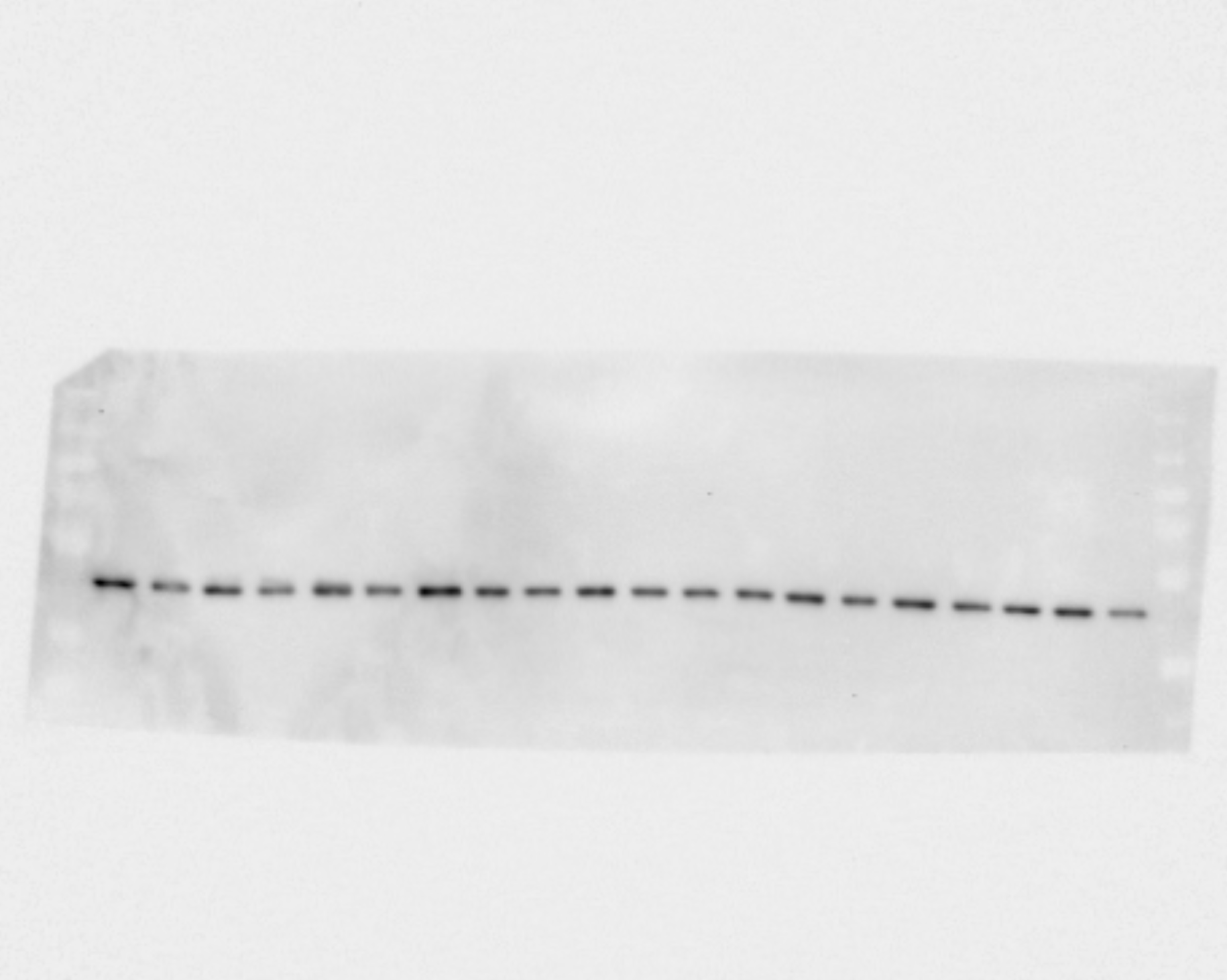

Supplement: Figure 4—source data 1. [file elife-83159-fig4-data1.zip › Figure 4-source data 1/ACTIN Figure 4-source data 1/Versteeg 2021-08-10 14h42m35s 34.654s(Chemiluminescence).tif]

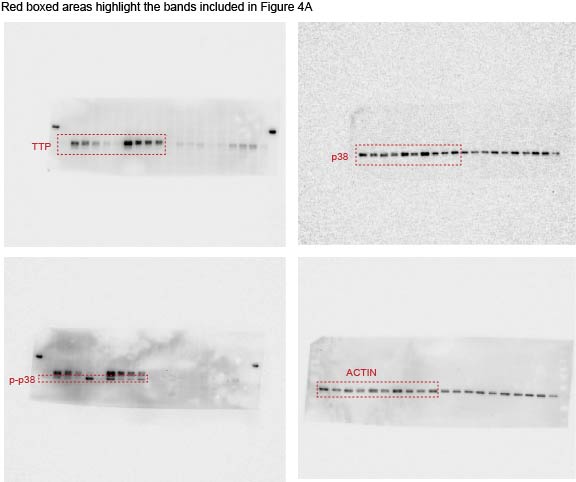

Supplement: Figure 4—source data 1. [file elife-83159-fig4-data1.zip › Figure 4-source data 1/Figure 4-source data 1.jpg]

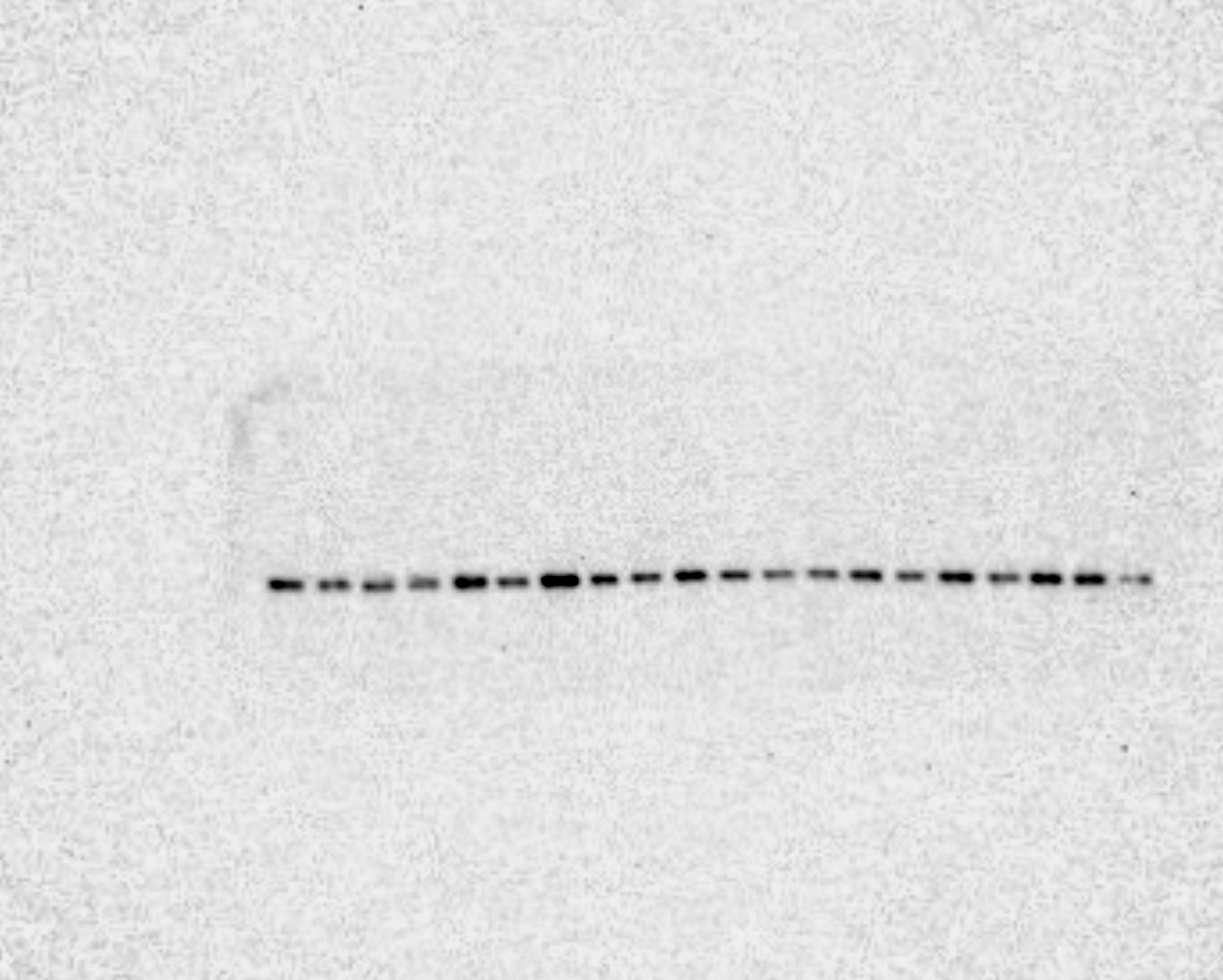

Supplement: Figure 4—source data 1. [file elife-83159-fig4-data1.zip › Figure 4-source data 1/p38 Figure 4-source data 1/Versteeg 2021-08-04 15h39m34s 299.990s(Chemiluminescence).jpg]

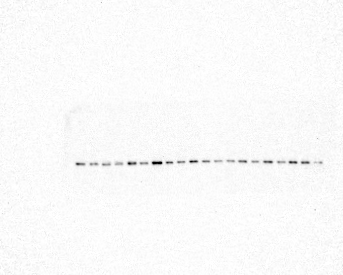

Supplement: Figure 4—source data 1. [file elife-83159-fig4-data1.zip › Figure 4-source data 1/p38 Figure 4-source data 1/Versteeg 2021-08-04 15h39m34s 299.990s(Chemiluminescence).raw16.tif]

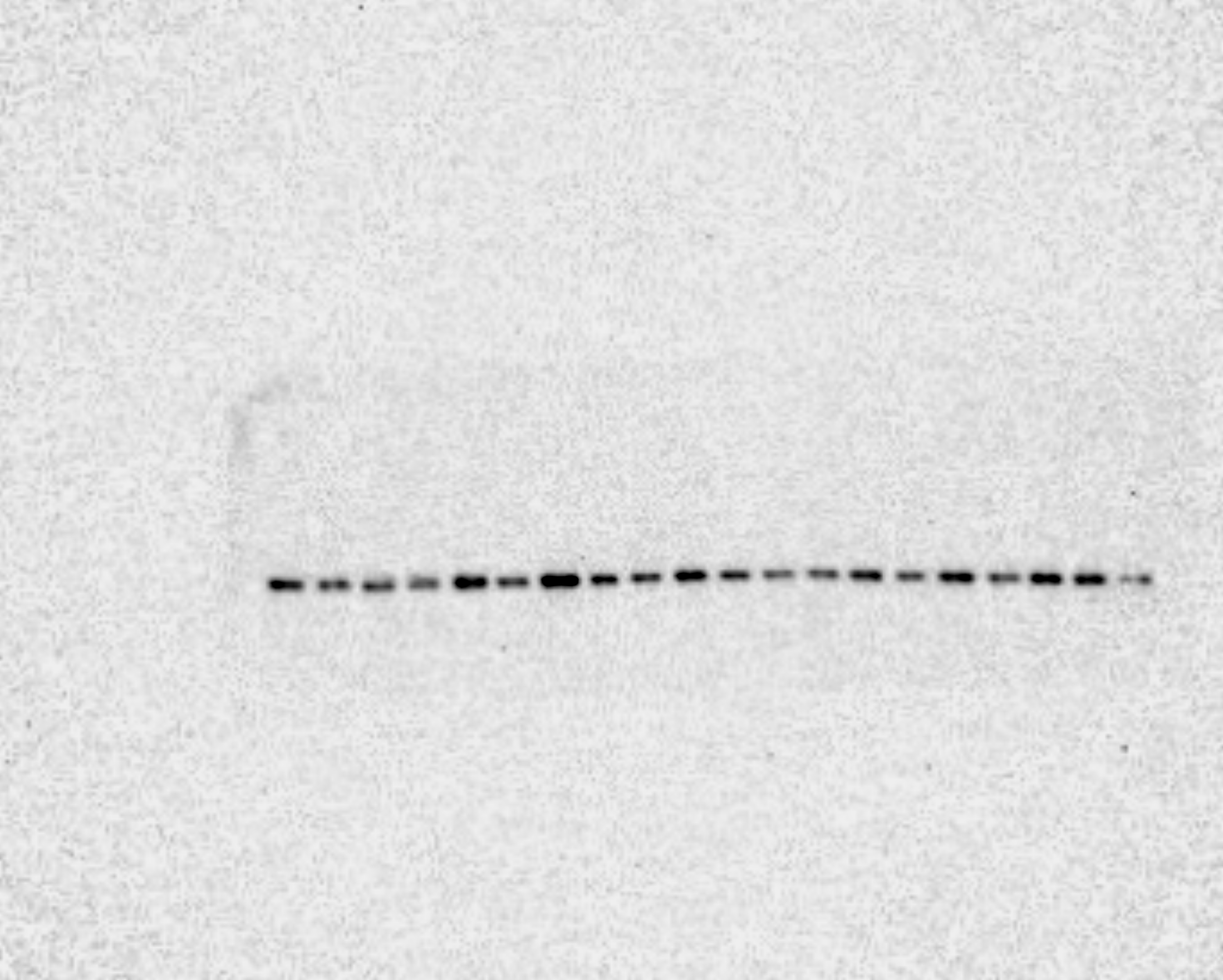

Supplement: Figure 4—source data 1. [file elife-83159-fig4-data1.zip › Figure 4-source data 1/p38 Figure 4-source data 1/Versteeg 2021-08-04 15h39m34s 299.990s(Chemiluminescence).tif]

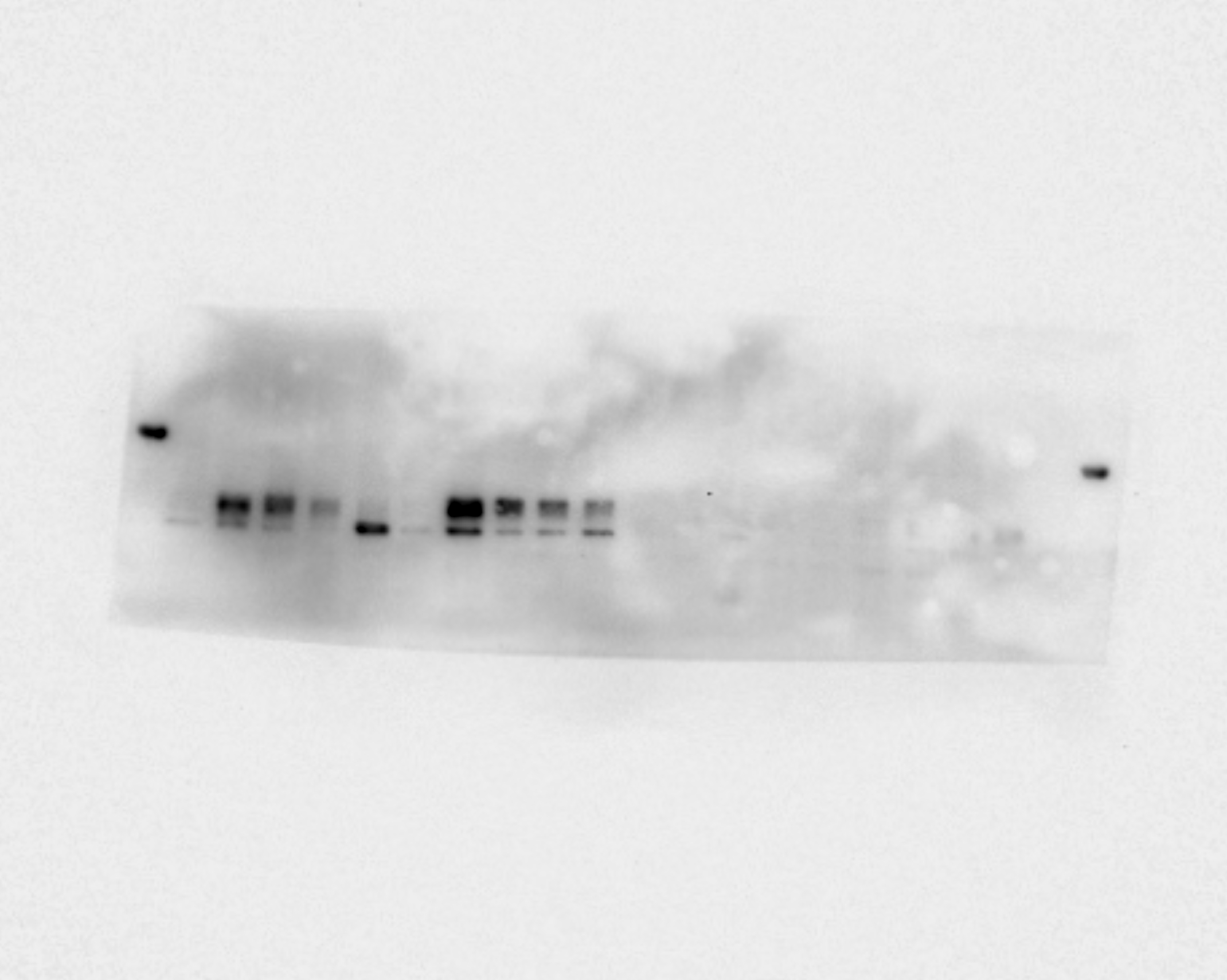

Supplement: Figure 4—source data 1. [file elife-83159-fig4-data1.zip › Figure 4-source data 1/p-p38 Figure 4-source data 1/Versteeg 2021-07-30 14h38m25s 67.240s(Chemiluminescence).jpg]

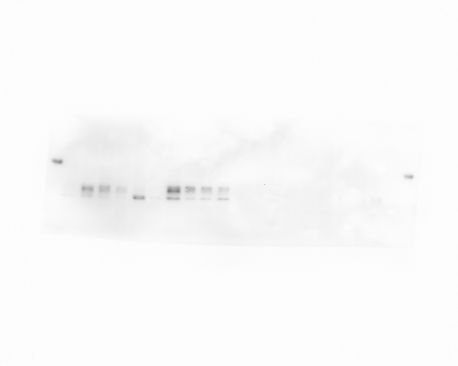

Supplement: Figure 4—source data 1. [file elife-83159-fig4-data1.zip › Figure 4-source data 1/p-p38 Figure 4-source data 1/Versteeg 2021-07-30 14h38m25s 67.240s(Chemiluminescence).raw16.tif]

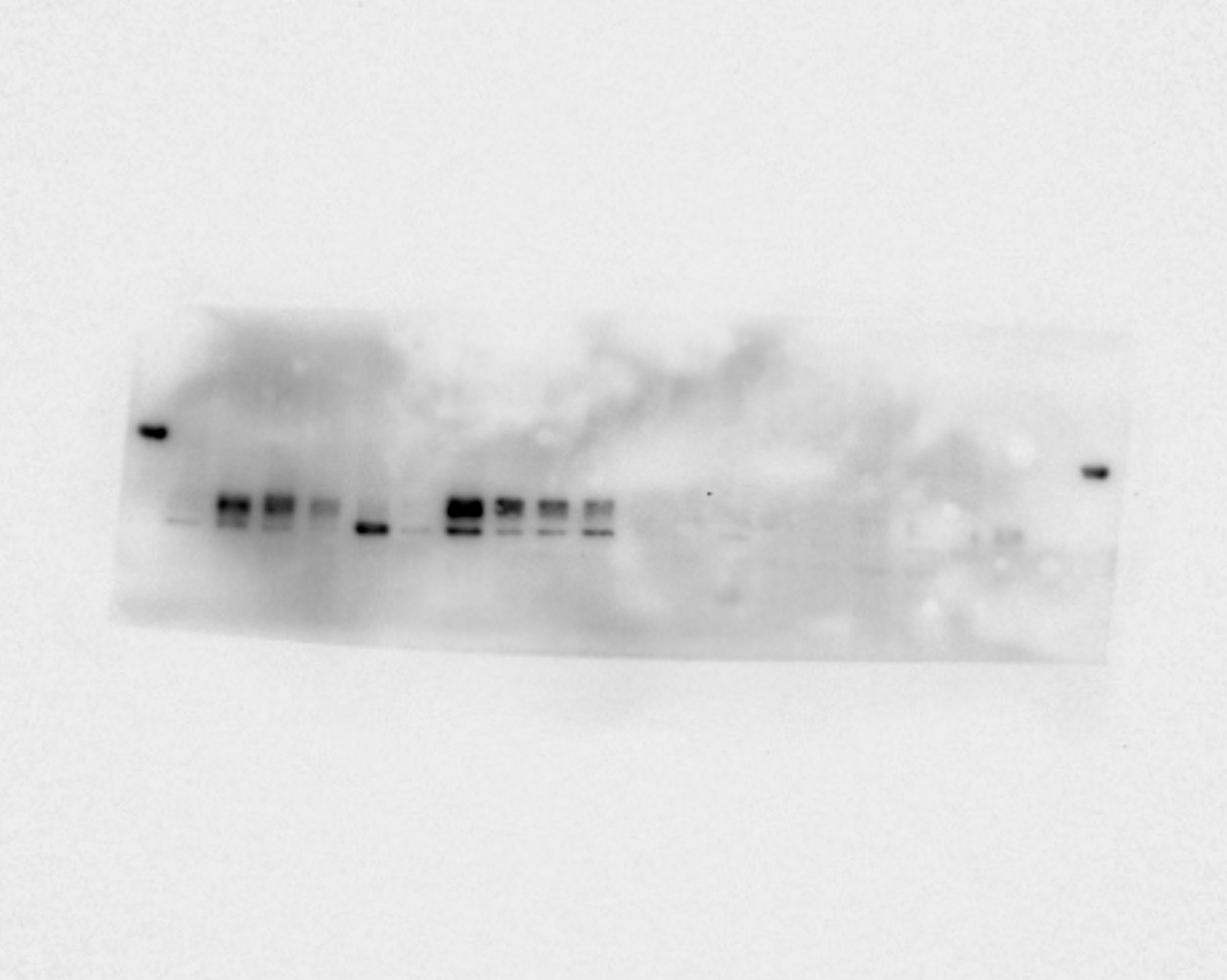

Supplement: Figure 4—source data 1. [file elife-83159-fig4-data1.zip › Figure 4-source data 1/p-p38 Figure 4-source data 1/Versteeg 2021-07-30 14h38m25s 67.240s(Chemiluminescence).tif]

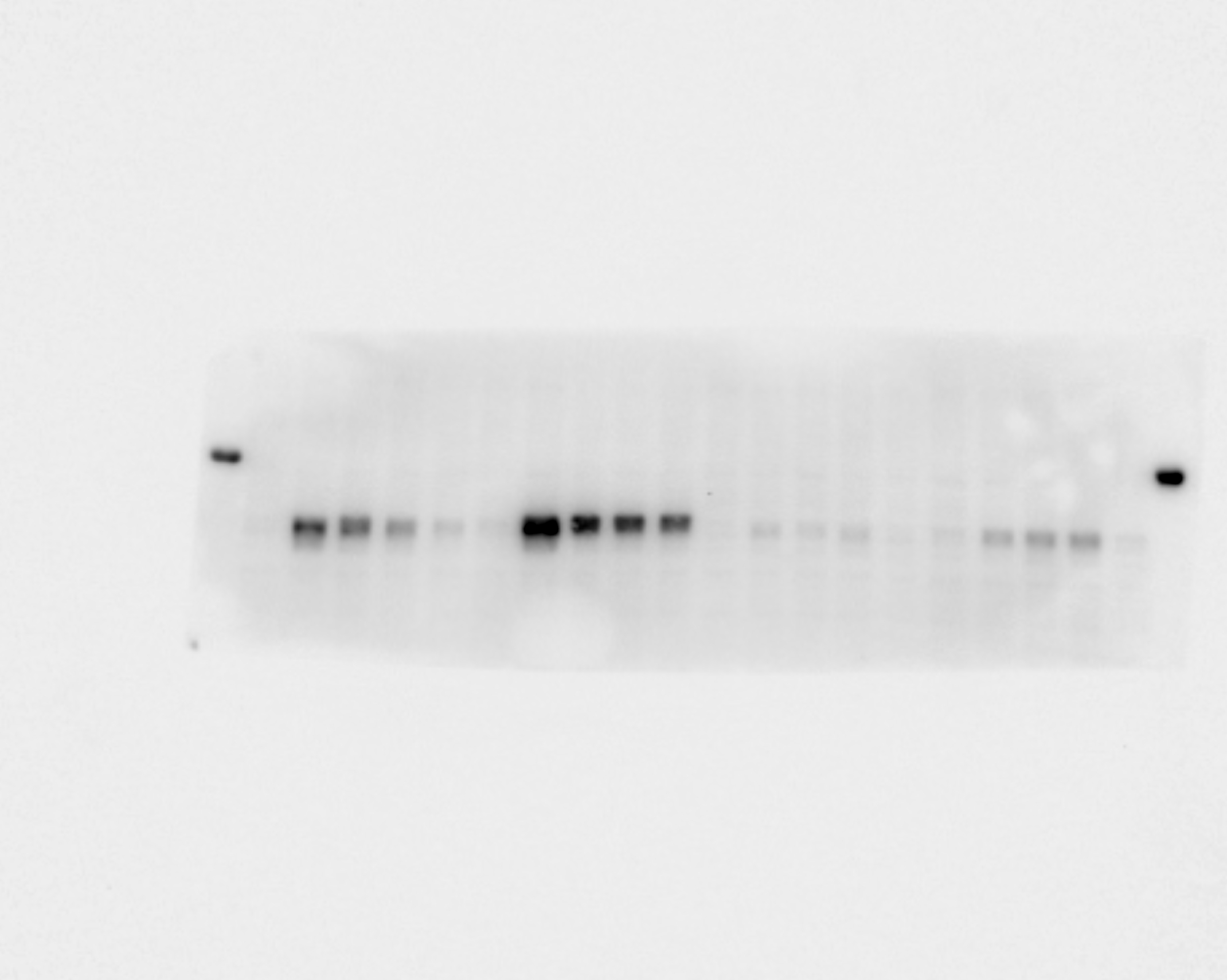

Supplement: Figure 4—source data 1. [file elife-83159-fig4-data1.zip › Figure 4-source data 1/TTP Figure 4-source data 1/Versteeg 2021-07-29 15h32m56s 20.000s(Chemiluminescence).jpg]

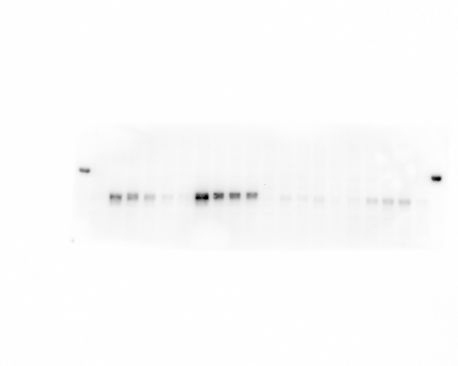

Supplement: Figure 4—source data 1. [file elife-83159-fig4-data1.zip › Figure 4-source data 1/TTP Figure 4-source data 1/Versteeg 2021-07-29 15h32m56s 20.000s(Chemiluminescence).raw16.tif]

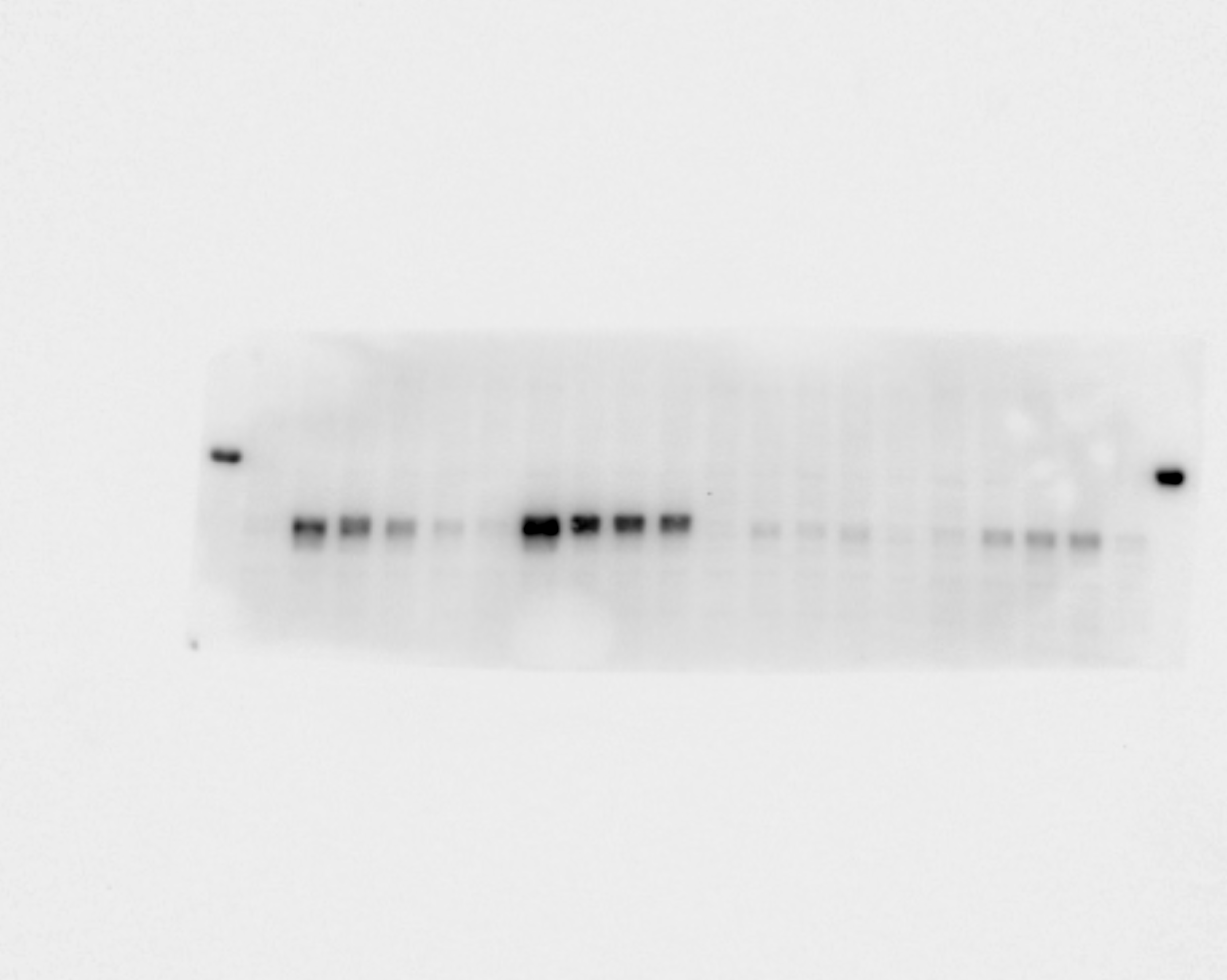

Supplement: Figure 4—source data 1. [file elife-83159-fig4-data1.zip › Figure 4-source data 1/TTP Figure 4-source data 1/Versteeg 2021-07-29 15h32m56s 20.000s(Chemiluminescence).tif]

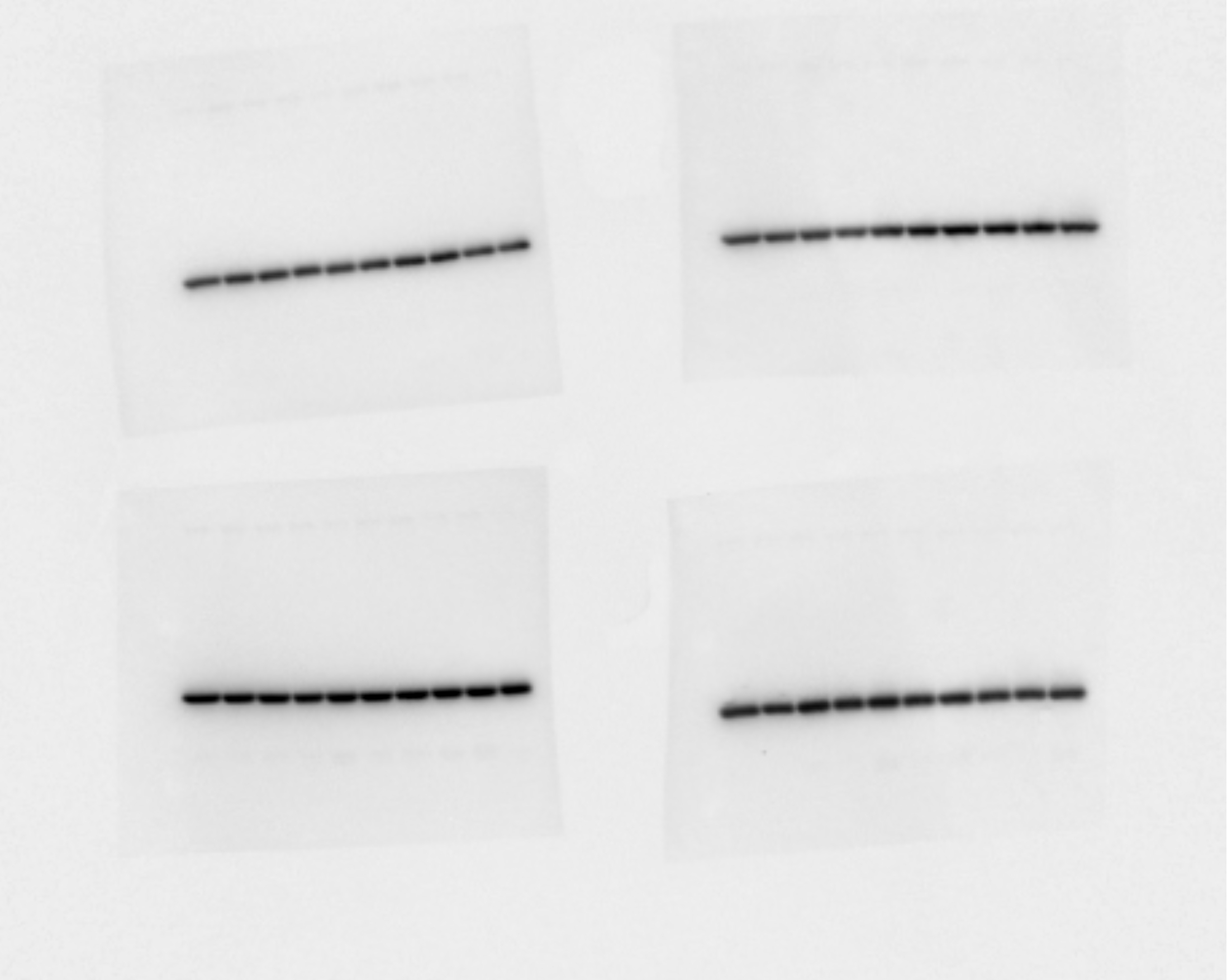

Supplement: Figure 4—source data 2. [file elife-83159-fig4-data2.zip › Figure 4-source data 2/ACTIN Figure 4-source data 2/Versteeg 2021-08-13 18h38m04s 5.103s(Chemiluminescence).jpg]

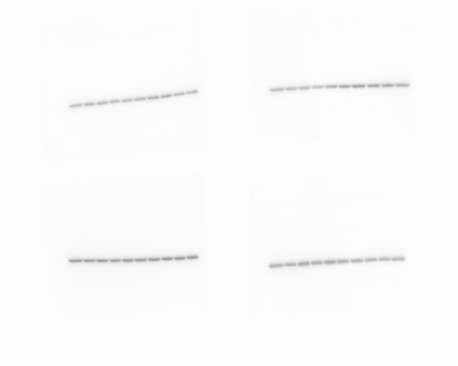

Supplement: Figure 4—source data 2. [file elife-83159-fig4-data2.zip › Figure 4-source data 2/ACTIN Figure 4-source data 2/Versteeg 2021-08-13 18h38m04s 5.103s(Chemiluminescence).raw16.tif]

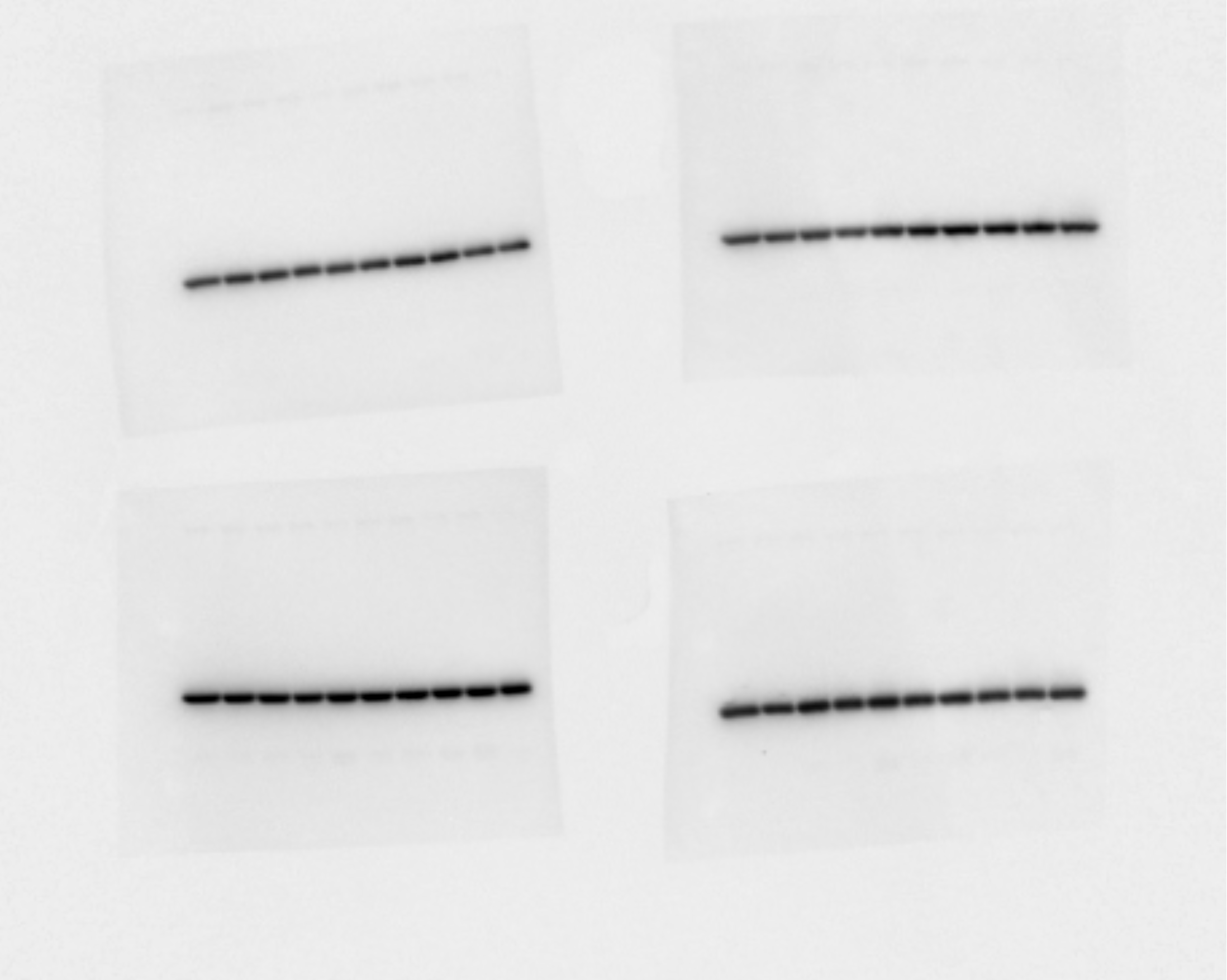

Supplement: Figure 4—source data 2. [file elife-83159-fig4-data2.zip › Figure 4-source data 2/ACTIN Figure 4-source data 2/Versteeg 2021-08-13 18h38m04s 5.103s(Chemiluminescence).tif]

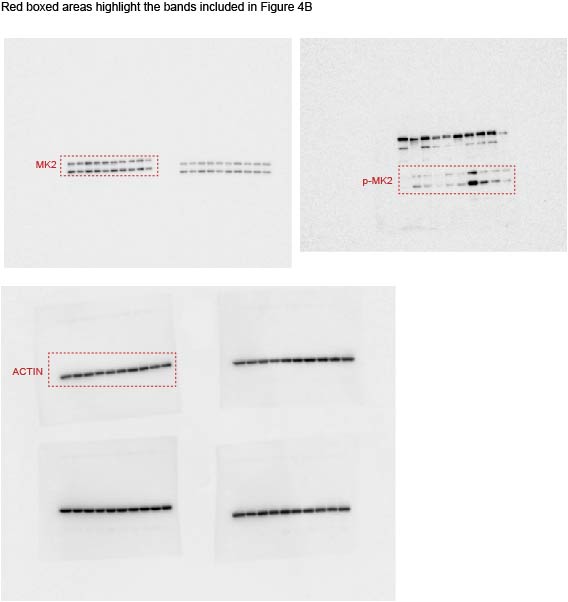

Supplement: Figure 4—source data 2. [file elife-83159-fig4-data2.zip › Figure 4-source data 2/Figure 4-source data 2.jpg]

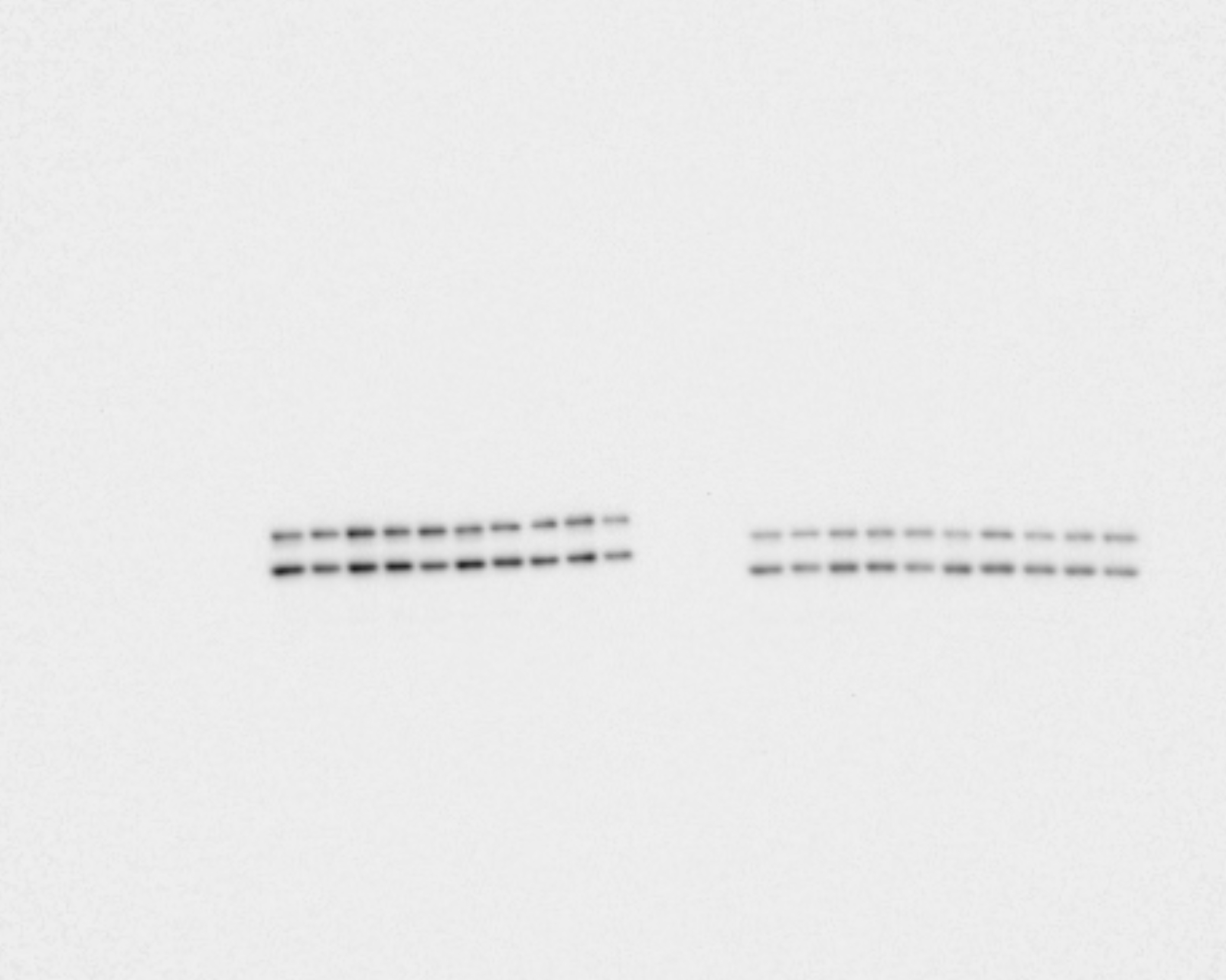

Supplement: Figure 4—source data 2. [file elife-83159-fig4-data2.zip › Figure 4-source data 2/MK2 Figure 4-source data 2/Versteeg 2021-08-13 13h44m08s 7.102s(Chemiluminescence).jpg]

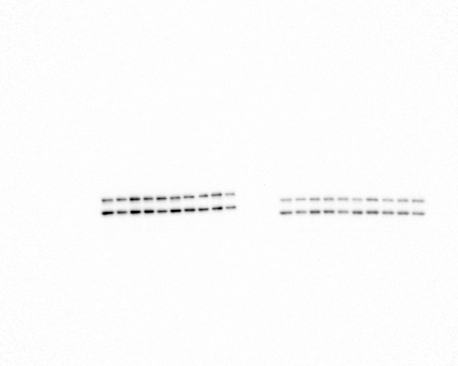

Supplement: Figure 4—source data 2. [file elife-83159-fig4-data2.zip › Figure 4-source data 2/MK2 Figure 4-source data 2/Versteeg 2021-08-13 13h44m08s 7.102s(Chemiluminescence).raw16.tif]

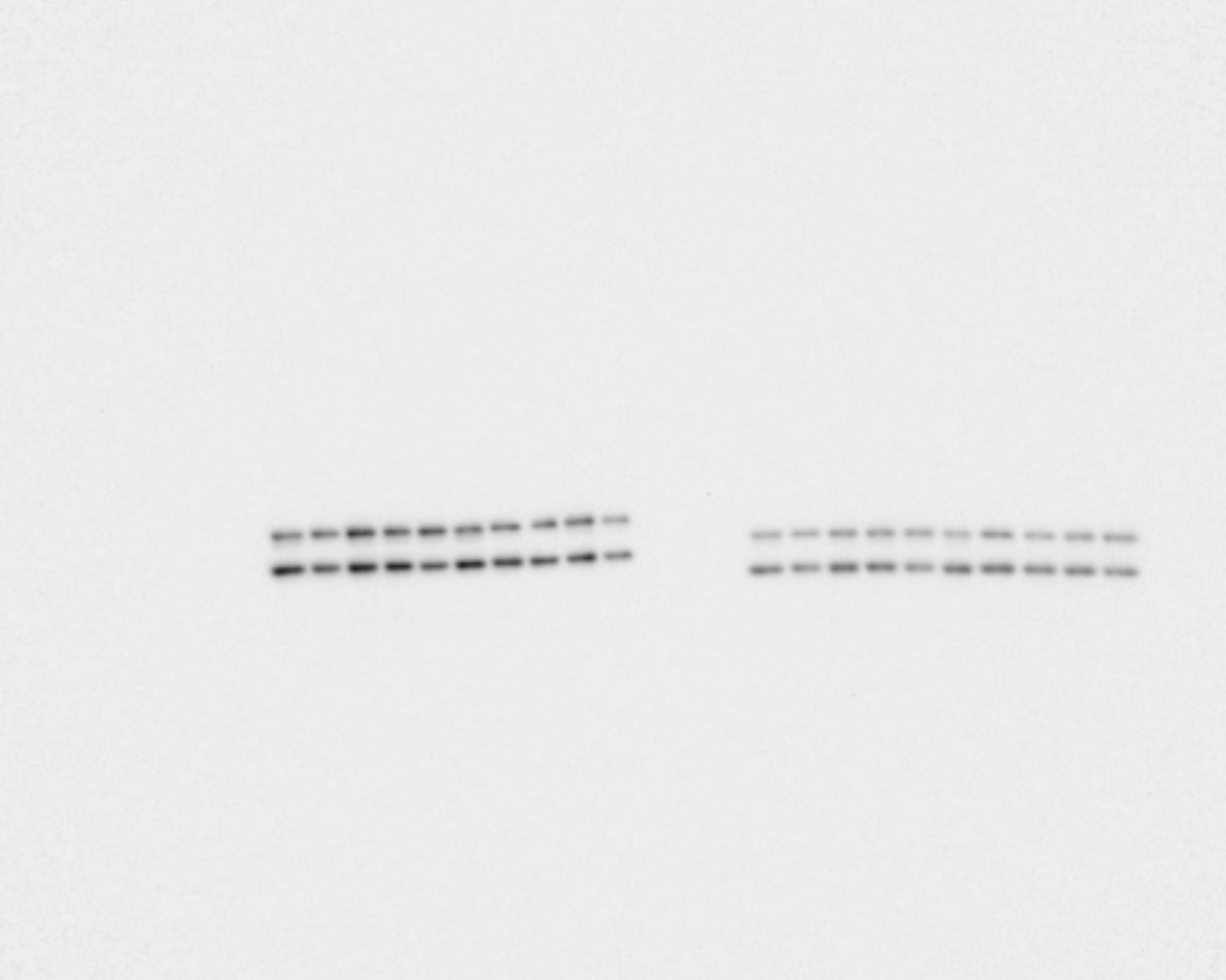

Supplement: Figure 4—source data 2. [file elife-83159-fig4-data2.zip › Figure 4-source data 2/MK2 Figure 4-source data 2/Versteeg 2021-08-13 13h44m08s 7.102s(Chemiluminescence).tif]

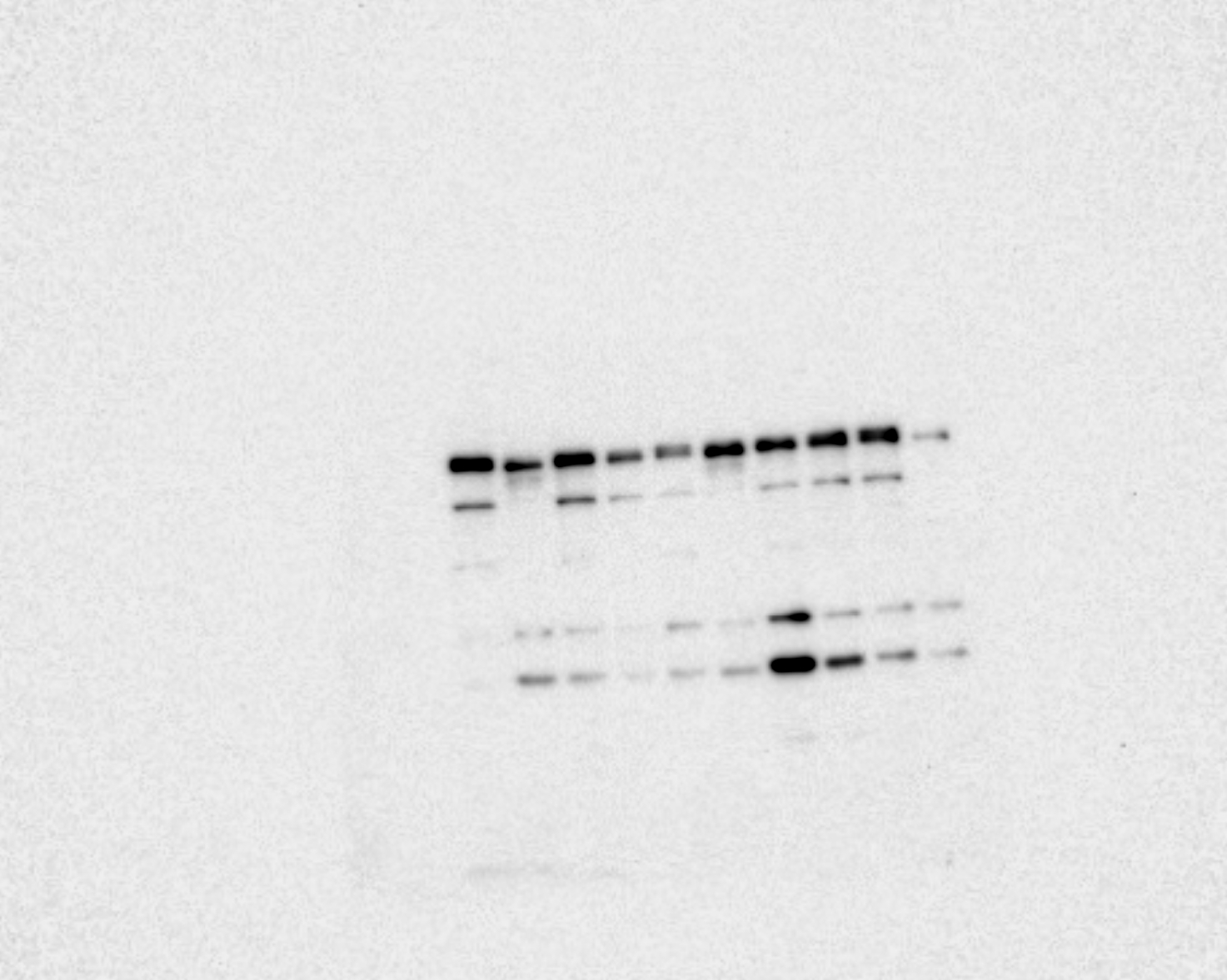

Supplement: Figure 4—source data 2. [file elife-83159-fig4-data2.zip › Figure 4-source data 2/p-MK2 Figure 4-source data 2/Versteeg 2021-08-12 17h02m58s 132.924s(Chemiluminescence).jpg]

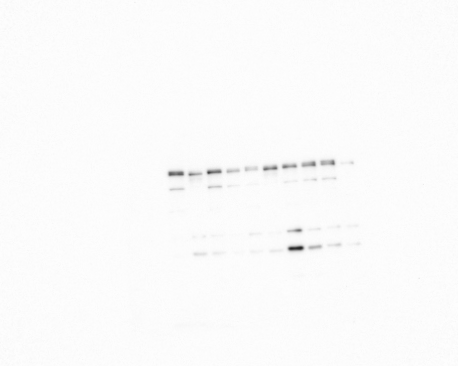

Supplement: Figure 4—source data 2. [file elife-83159-fig4-data2.zip › Figure 4-source data 2/p-MK2 Figure 4-source data 2/Versteeg 2021-08-12 17h02m58s 132.924s(Chemiluminescence).raw16.tif]

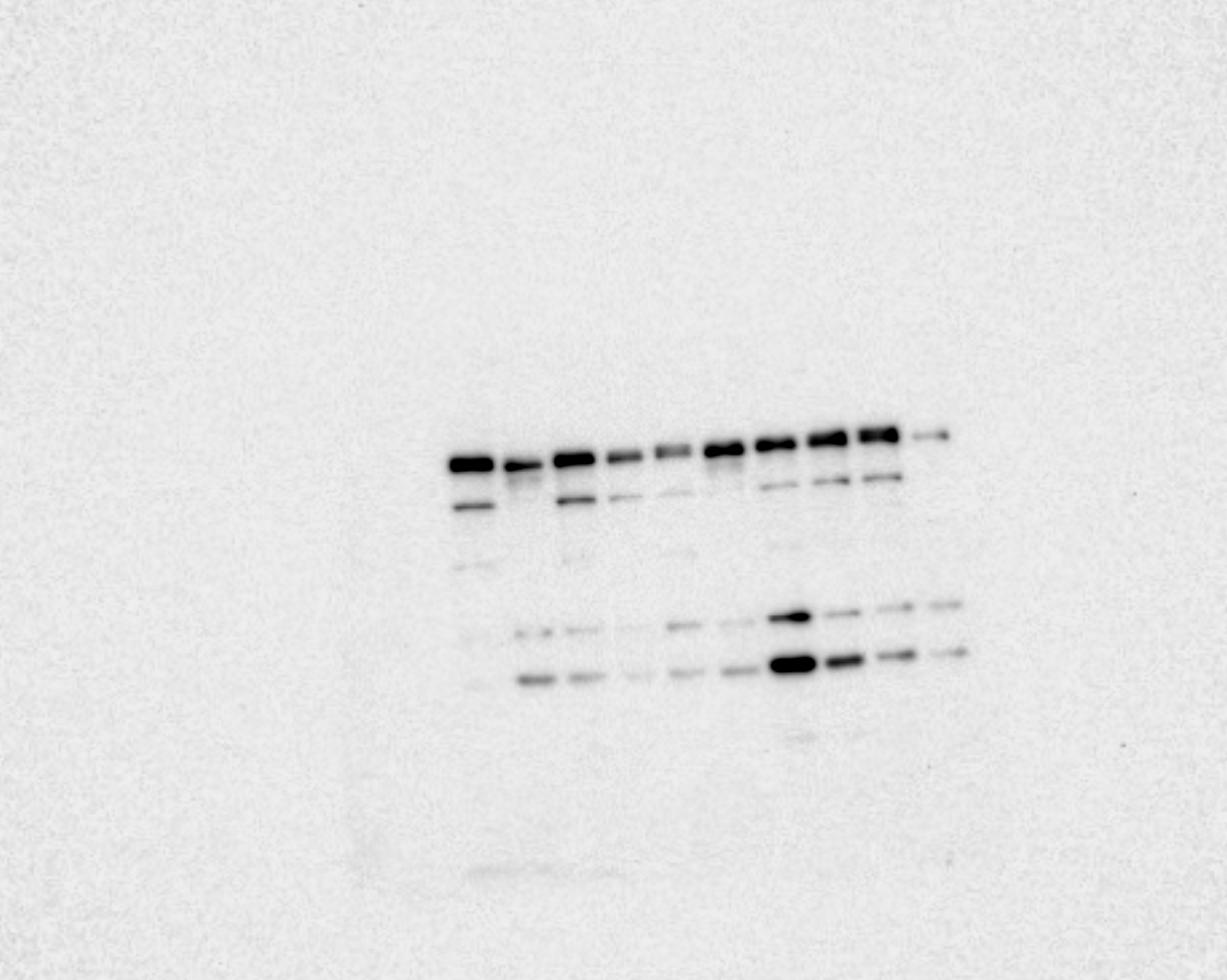

Supplement: Figure 4—source data 2. [file elife-83159-fig4-data2.zip › Figure 4-source data 2/p-MK2 Figure 4-source data 2/Versteeg 2021-08-12 17h02m58s 132.924s(Chemiluminescence).tif]

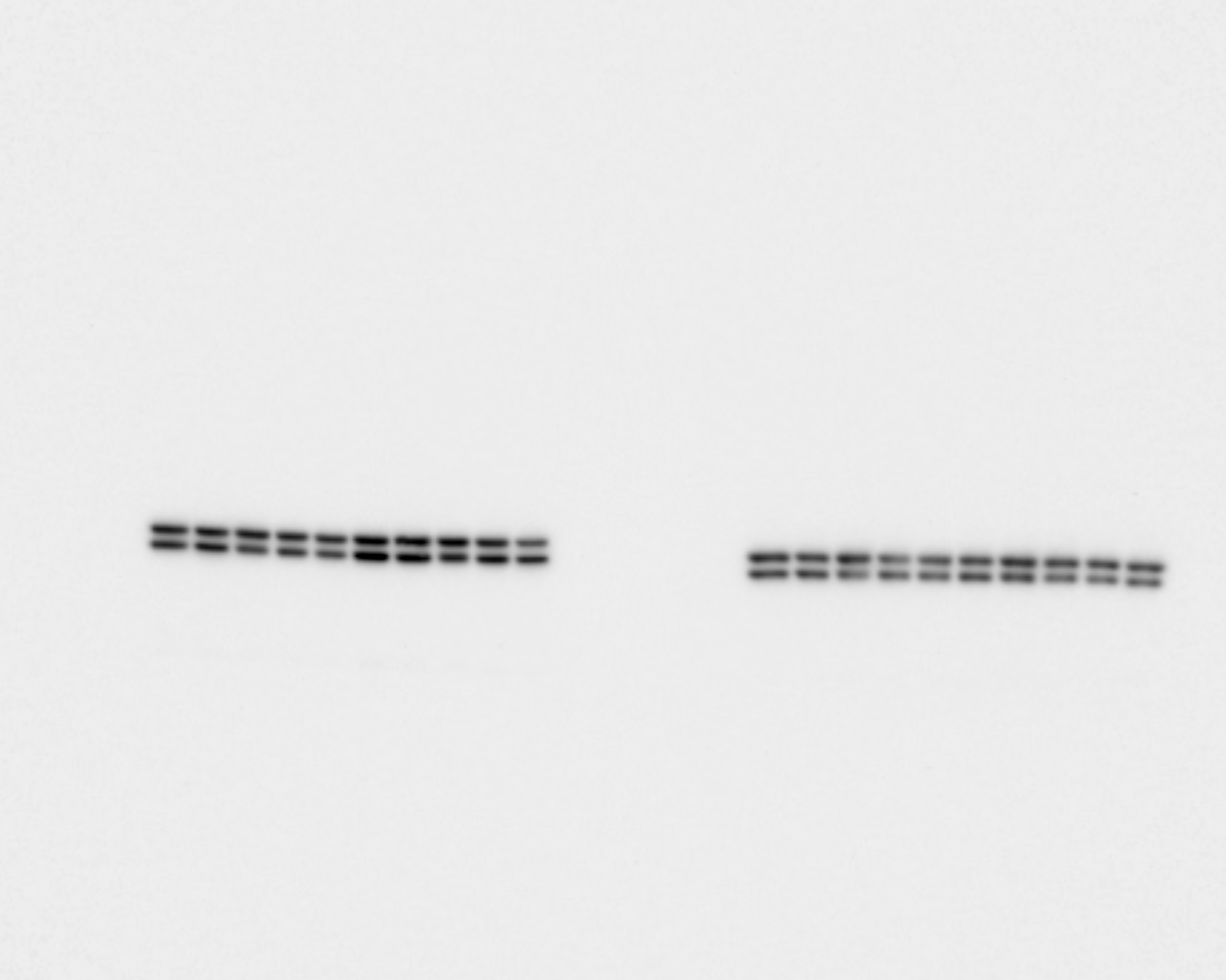

Supplement: Figure 4—source data 3. [file elife-83159-fig4-data3.zip › Figure 4-source data 3/ERK Figure 4-source data 3/Versteeg 2021-08-13 13h23m23s 39.310s(Chemiluminescence).jpg]

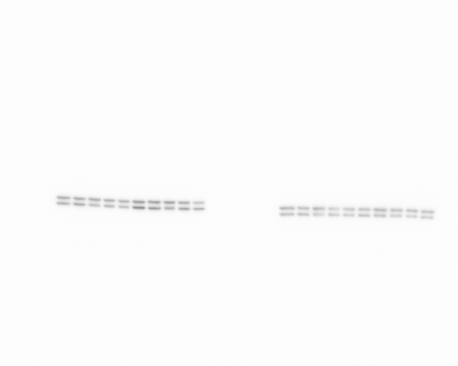

Supplement: Figure 4—source data 3. [file elife-83159-fig4-data3.zip › Figure 4-source data 3/ERK Figure 4-source data 3/Versteeg 2021-08-13 13h23m23s 39.310s(Chemiluminescence).raw16.tif]

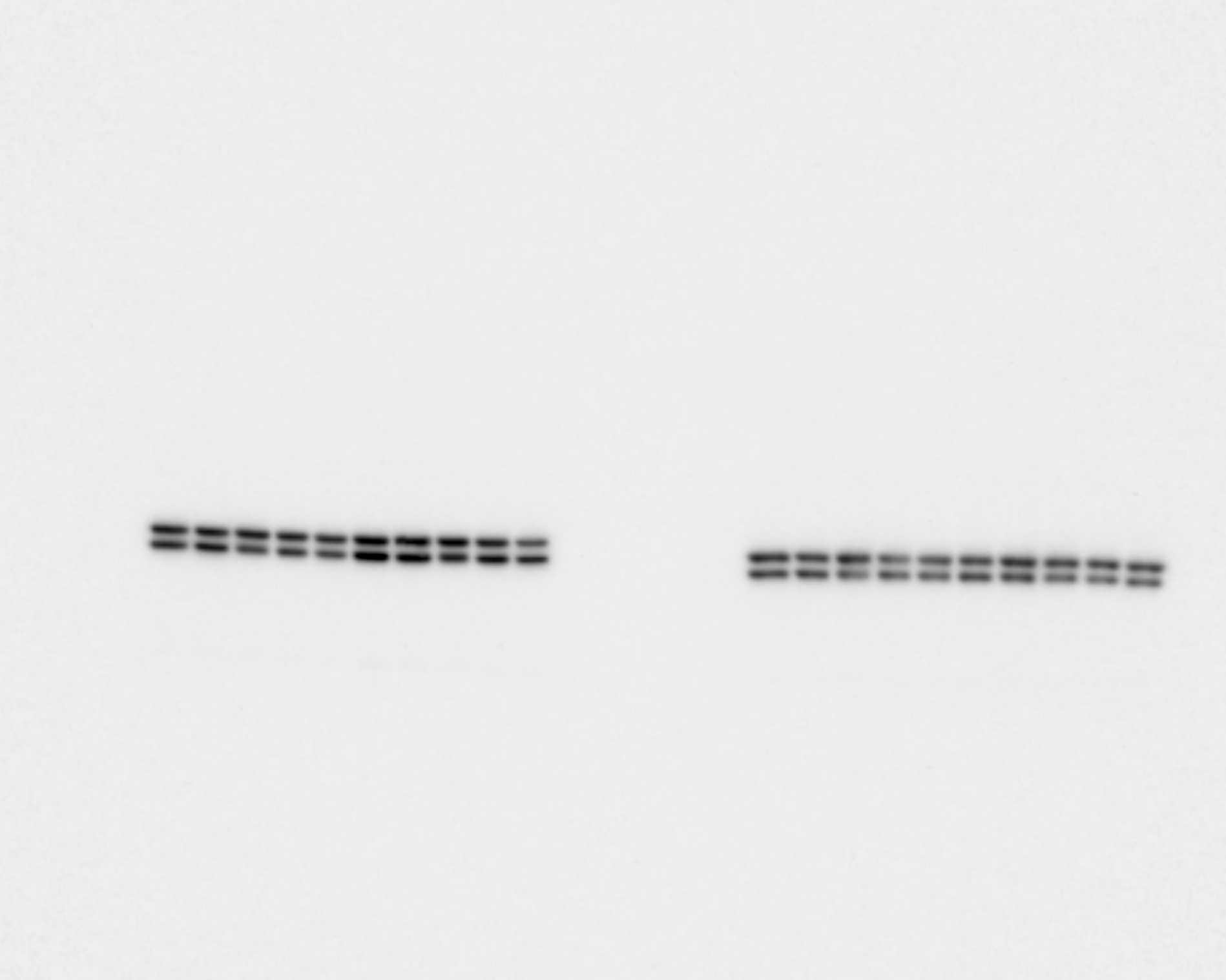

Supplement: Figure 4—source data 3. [file elife-83159-fig4-data3.zip › Figure 4-source data 3/ERK Figure 4-source data 3/Versteeg 2021-08-13 13h23m23s 39.310s(Chemiluminescence).tif]

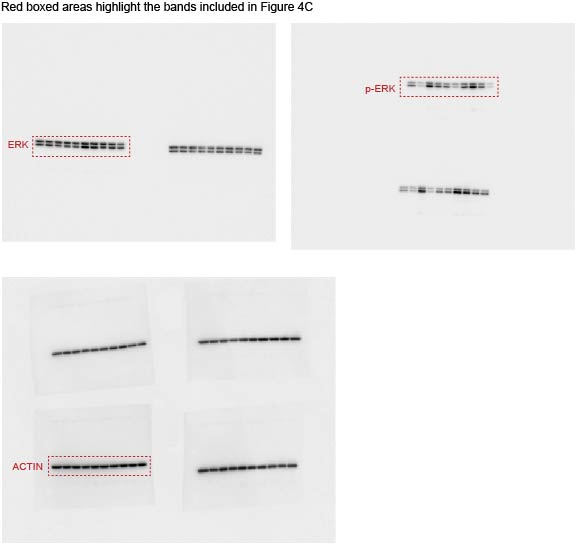

Supplement: Figure 4—source data 3. [file elife-83159-fig4-data3.zip › Figure 4-source data 3/Figure 4-source data 3.jpg]

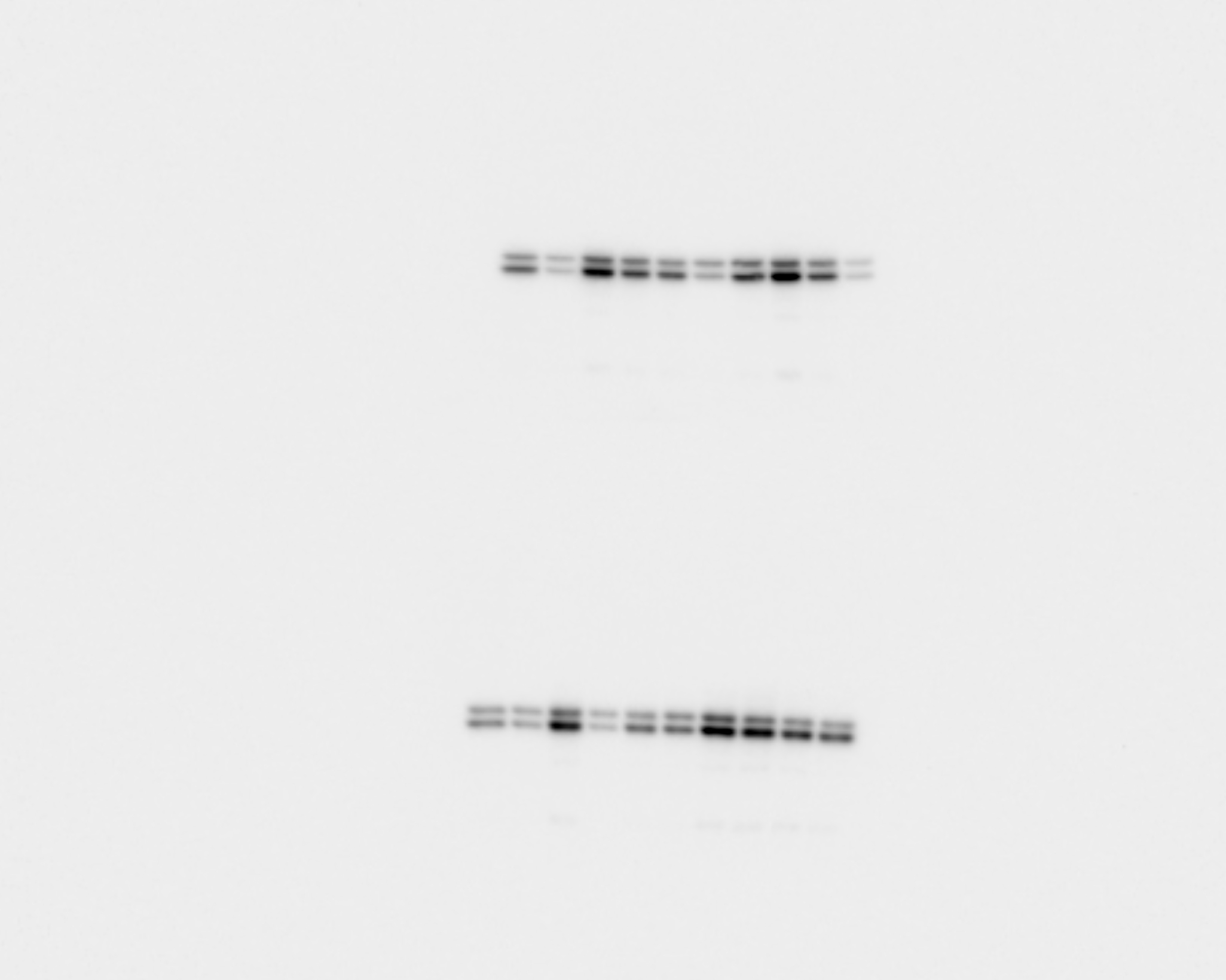

Supplement: Figure 4—source data 3. [file elife-83159-fig4-data3.zip › Figure 4-source data 3/p-ERK Figure 4-source data 3/Versteeg 2021-08-12 15h46m30s 15.000s(Chemiluminescence).jpg]

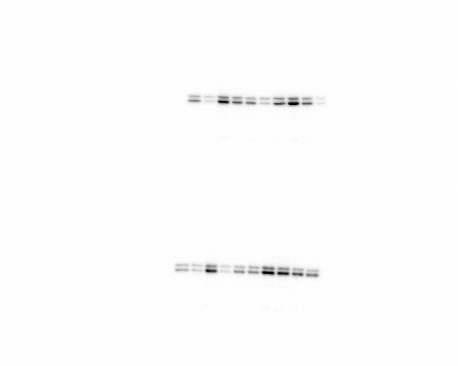

Supplement: Figure 4—source data 3. [file elife-83159-fig4-data3.zip › Figure 4-source data 3/p-ERK Figure 4-source data 3/Versteeg 2021-08-12 15h46m30s 15.000s(Chemiluminescence).raw16.tif]

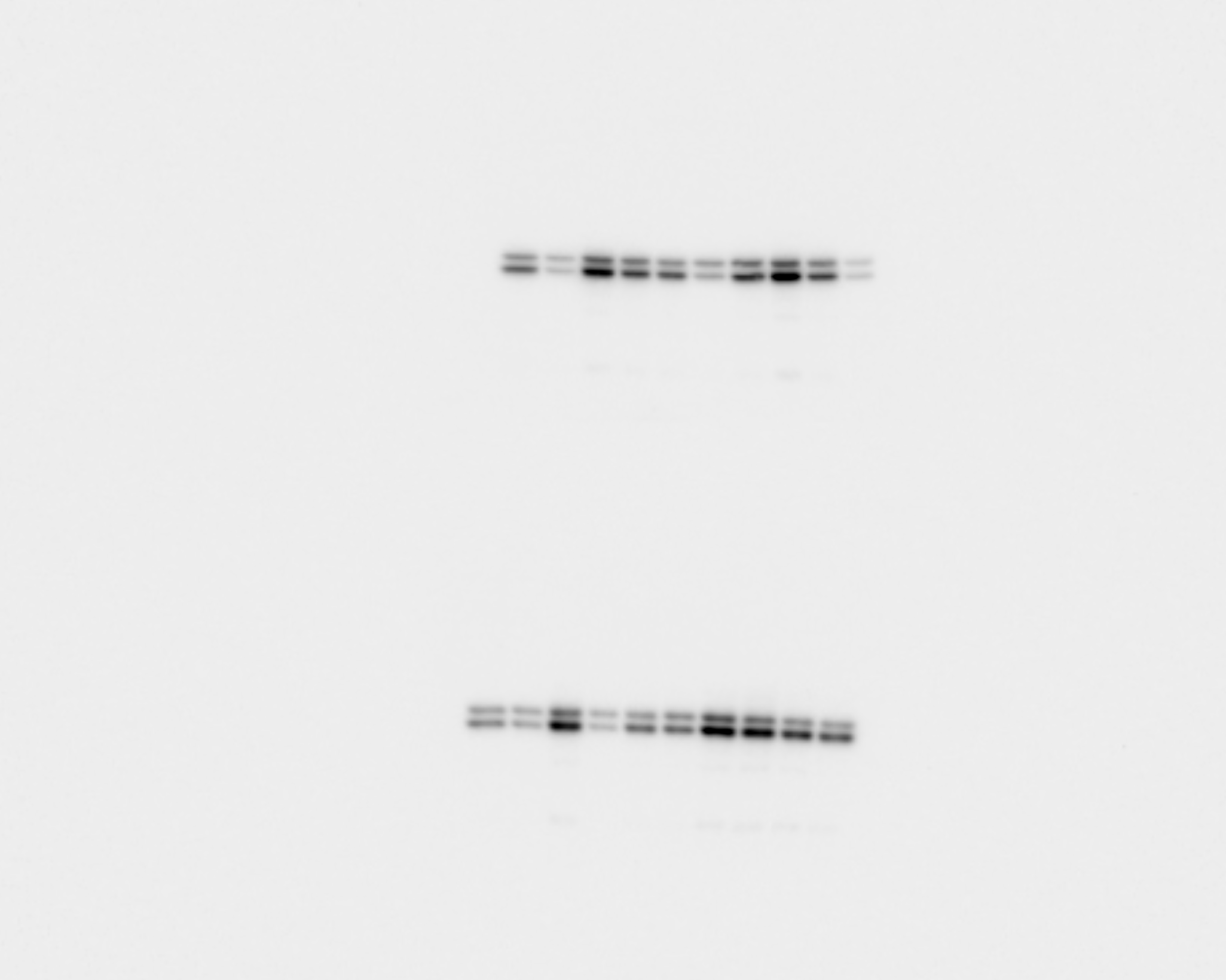

Supplement: Figure 4—source data 3. [file elife-83159-fig4-data3.zip › Figure 4-source data 3/p-ERK Figure 4-source data 3/Versteeg 2021-08-12 15h46m30s 15.000s(Chemiluminescence).tif]

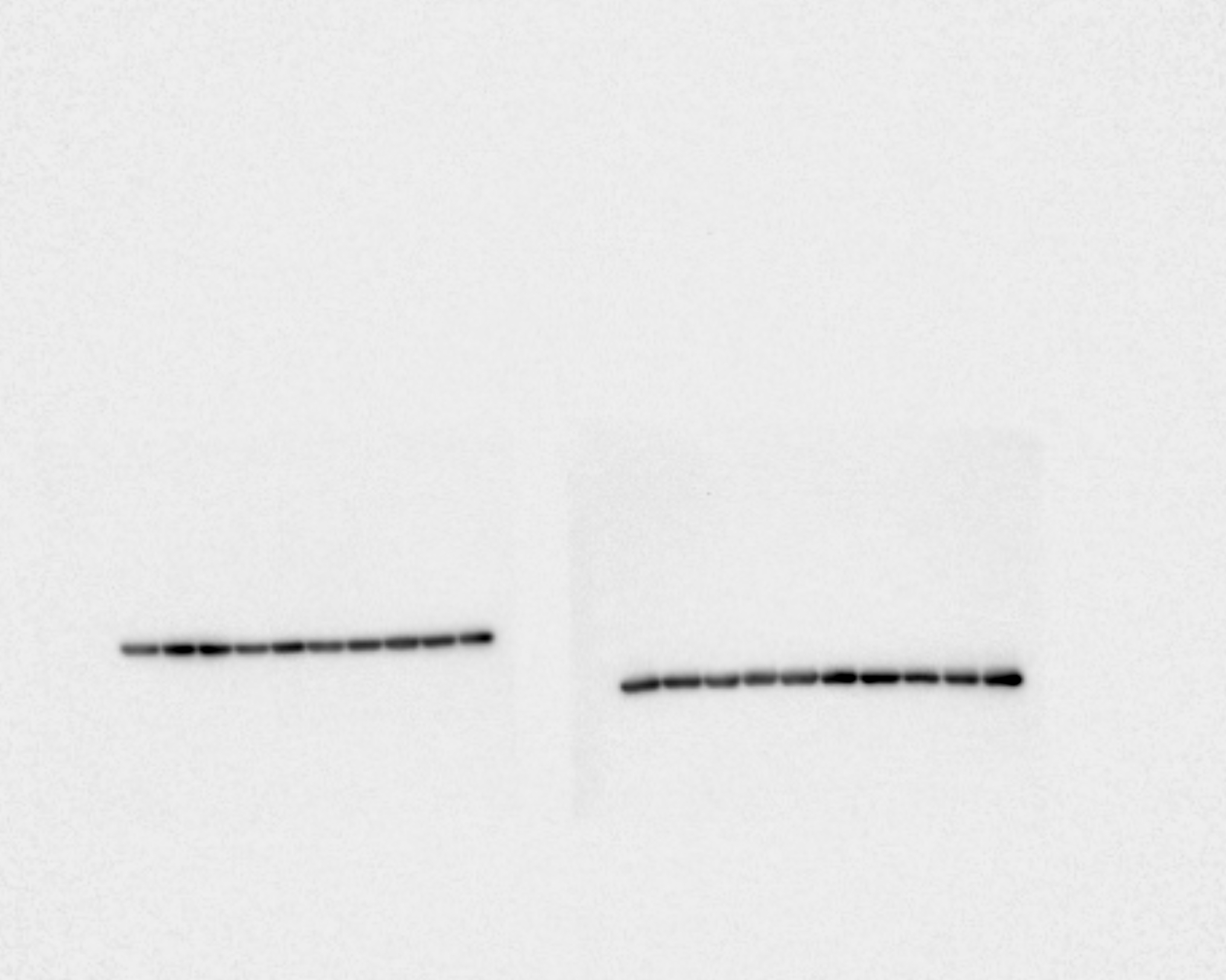

Supplement: Figure 4—source data 4. [file elife-83159-fig4-data4.zip › Figure 4-source data 4/ACTIN Figure 4-source data 4/Versteeg 2021-10-20 16h49m06s 11.170s(Chemiluminescence).jpg]

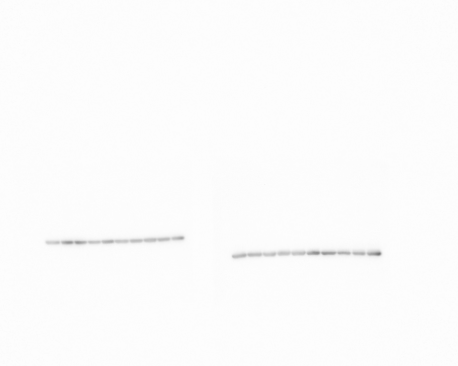

Supplement: Figure 4—source data 4. [file elife-83159-fig4-data4.zip › Figure 4-source data 4/ACTIN Figure 4-source data 4/Versteeg 2021-10-20 16h49m06s 11.170s(Chemiluminescence).raw16.tif]

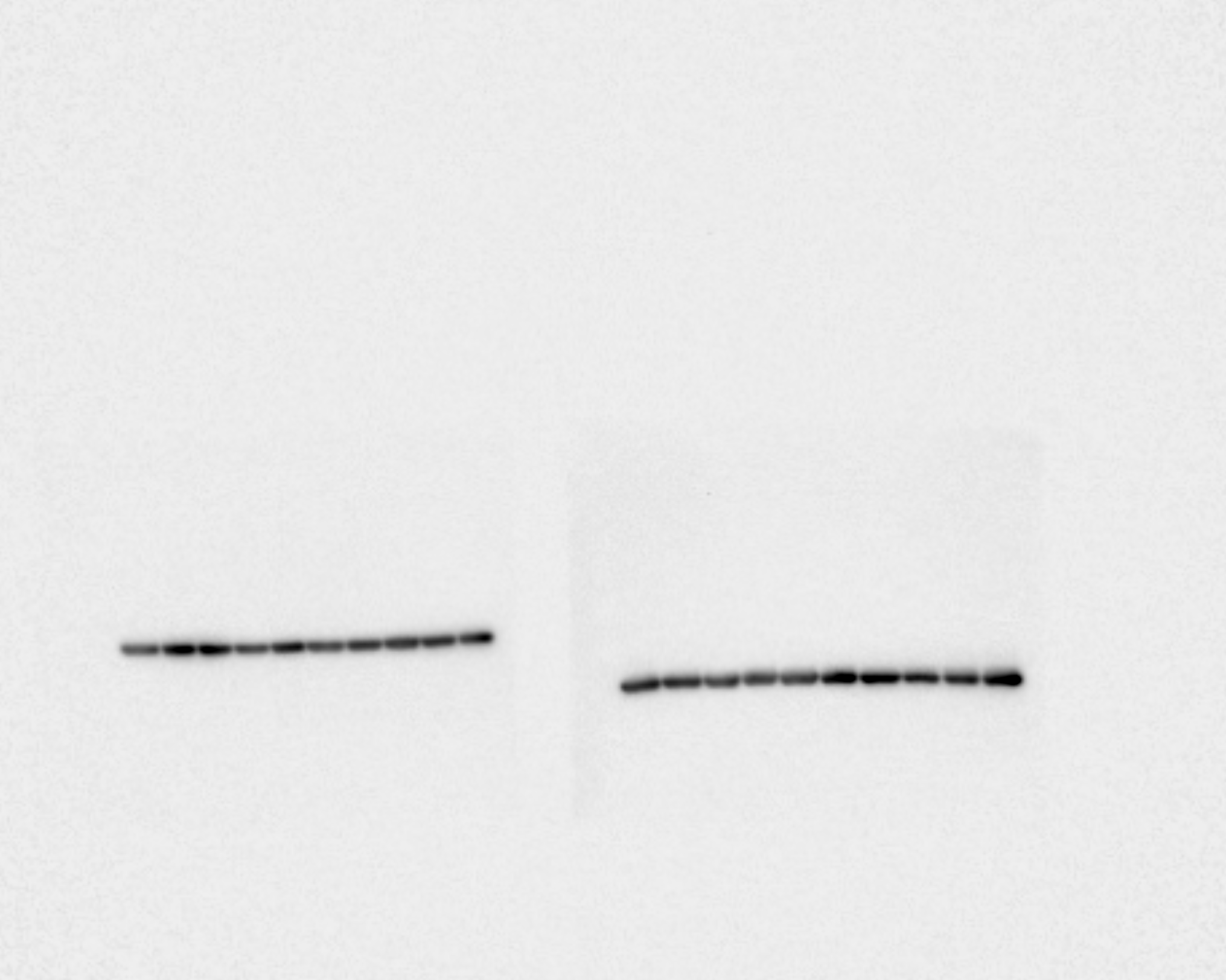

Supplement: Figure 4—source data 4. [file elife-83159-fig4-data4.zip › Figure 4-source data 4/ACTIN Figure 4-source data 4/Versteeg 2021-10-20 16h49m06s 11.170s(Chemiluminescence).tif]

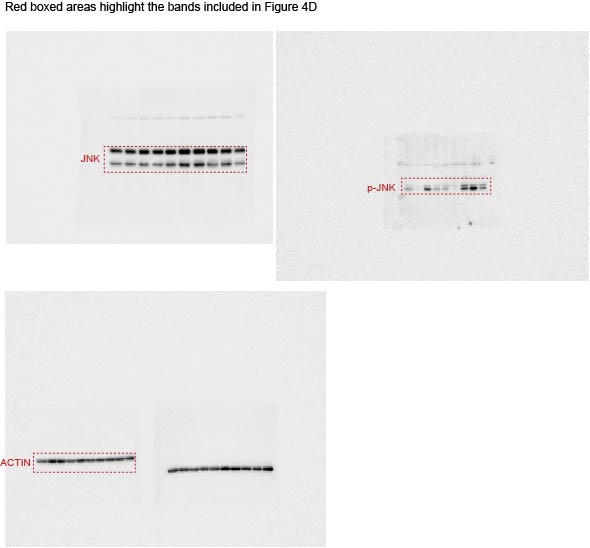

Supplement: Figure 4—source data 4. [file elife-83159-fig4-data4.zip › Figure 4-source data 4/Figure 4-source data 4.jpg]

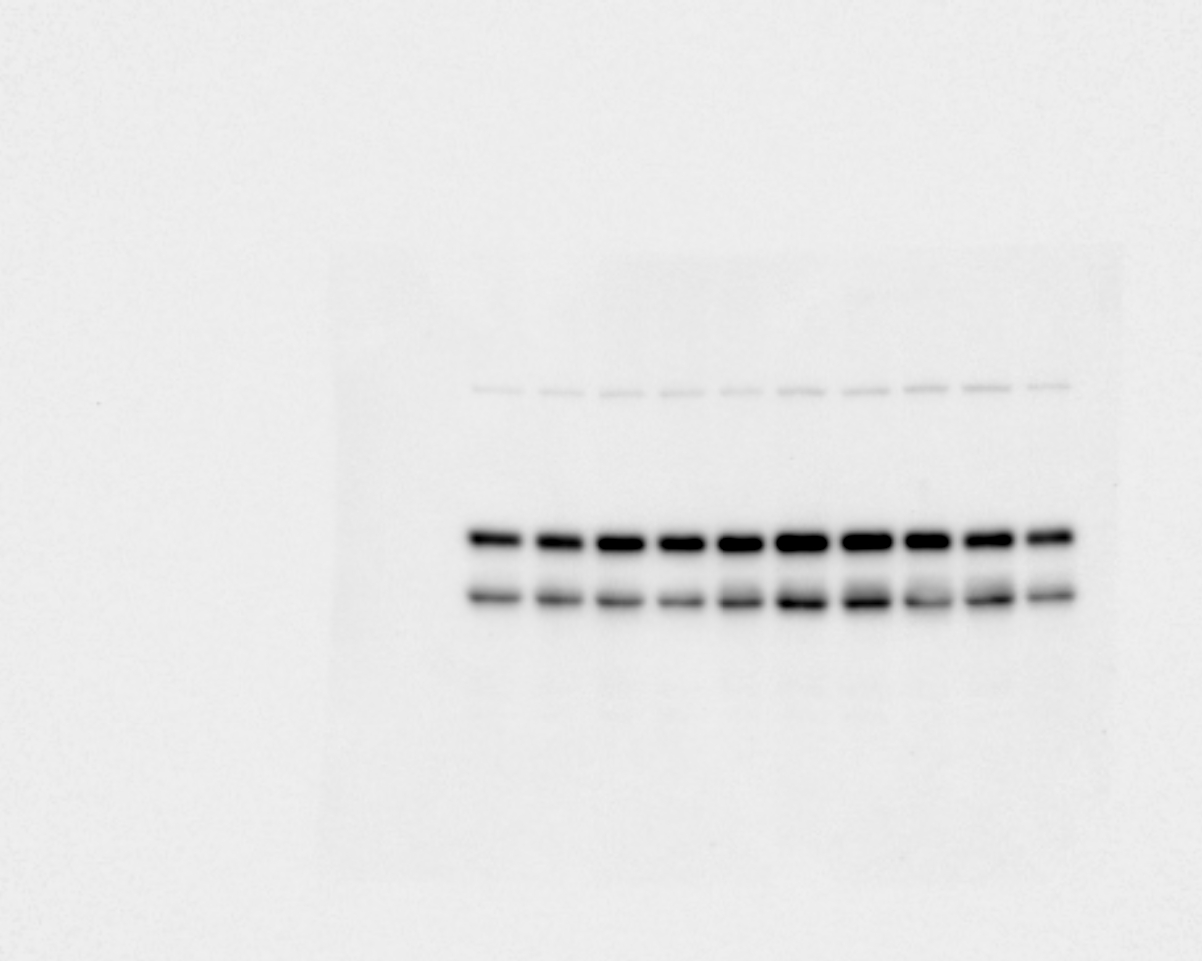

Supplement: Figure 4—source data 4. [file elife-83159-fig4-data4.zip › Figure 4-source data 4/JNK Figure 4-source data 4/Versteeg 2021-10-20 12h58m10s 30.000s(Chemiluminescence).jpg]

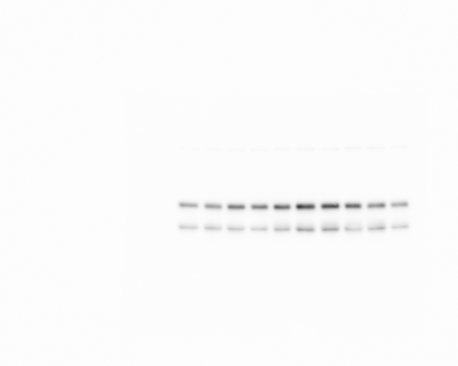

Supplement: Figure 4—source data 4. [file elife-83159-fig4-data4.zip › Figure 4-source data 4/JNK Figure 4-source data 4/Versteeg 2021-10-20 12h58m10s 30.000s(Chemiluminescence).raw16.tif]

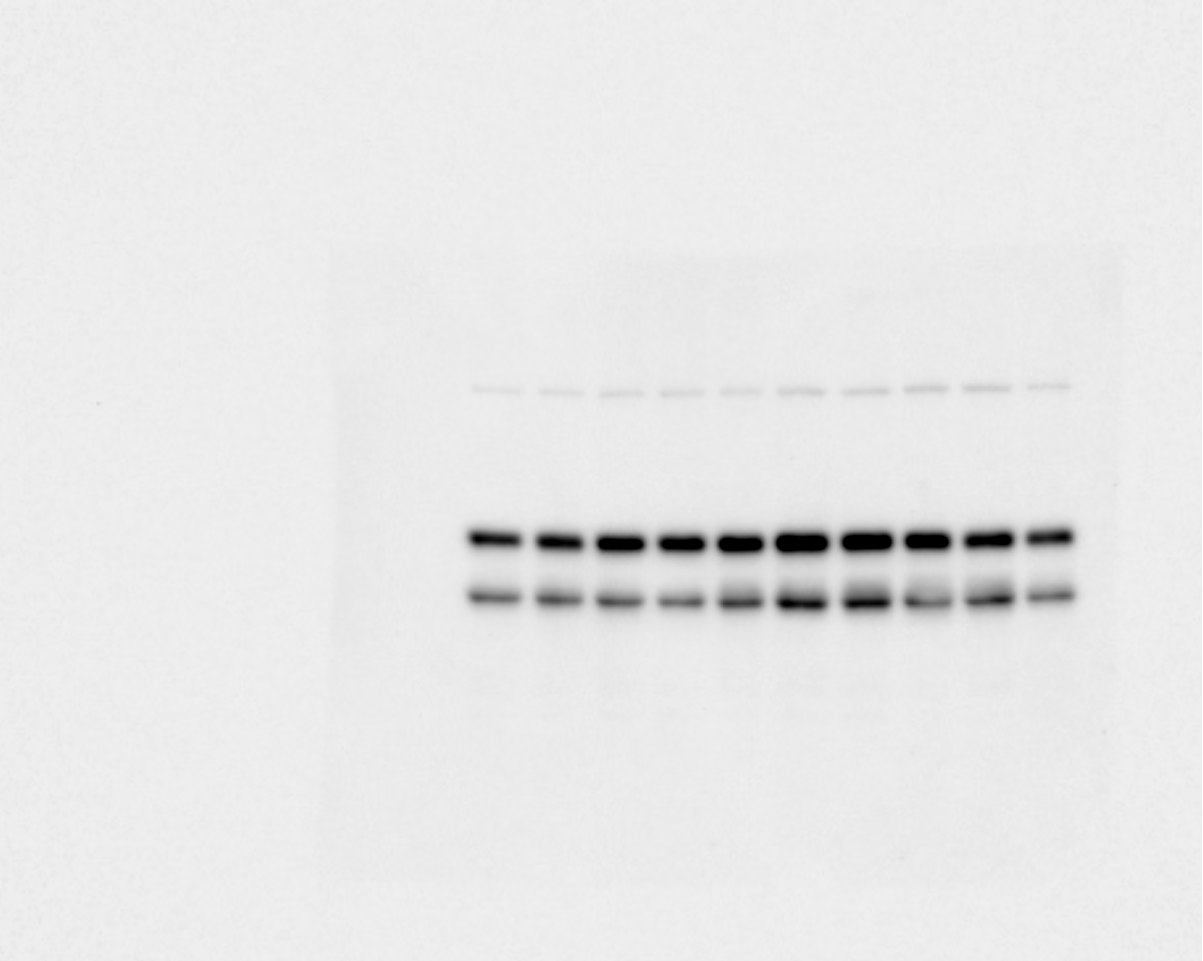

Supplement: Figure 4—source data 4. [file elife-83159-fig4-data4.zip › Figure 4-source data 4/JNK Figure 4-source data 4/Versteeg 2021-10-20 12h58m10s 30.000s(Chemiluminescence).tif]

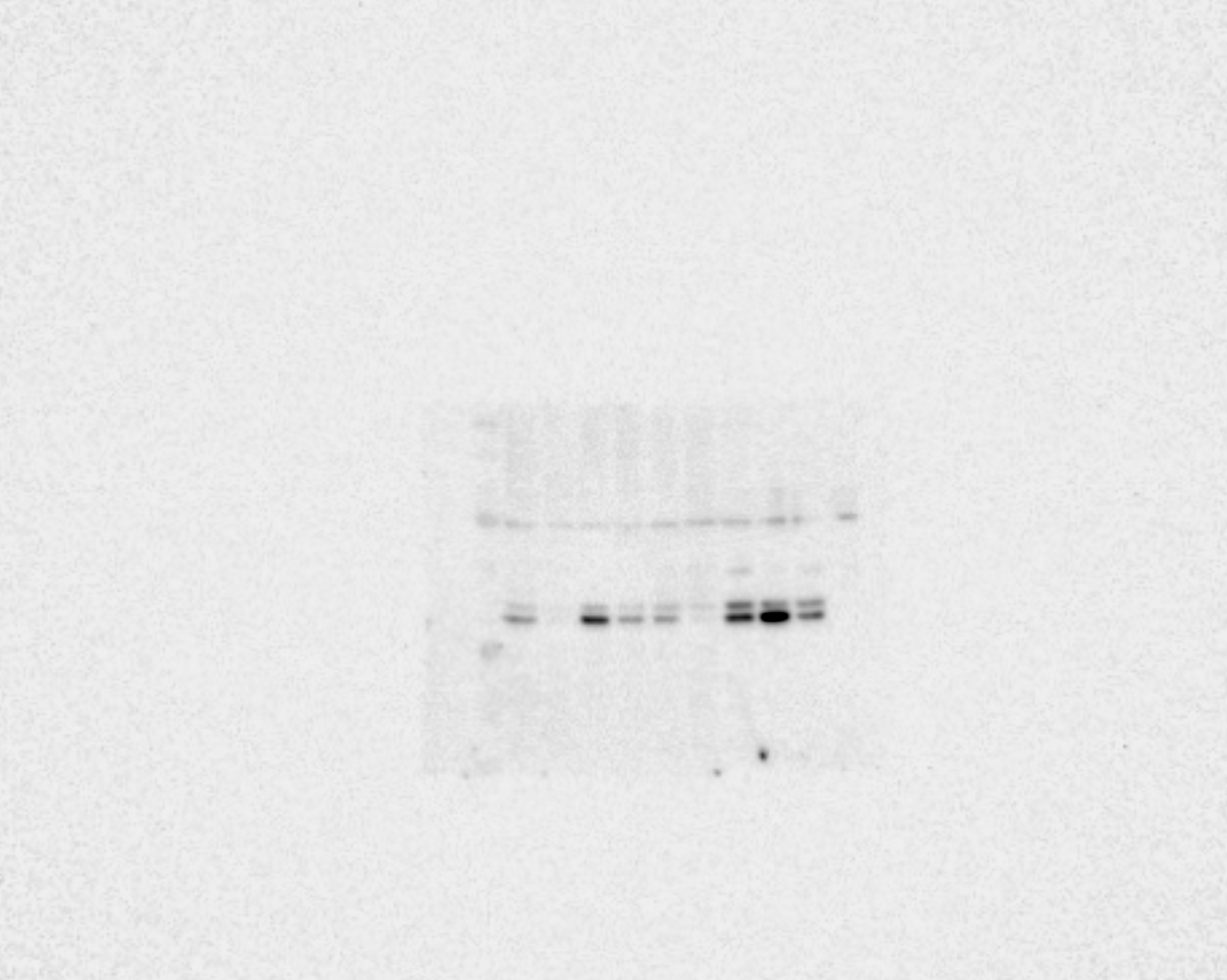

Supplement: Figure 4—source data 4. [file elife-83159-fig4-data4.zip › Figure 4-source data 4/p-JNK Figure 4-source data 4/Versteeg 2021-08-13 14h42m12s 159.300s(Chemiluminescence).jpg]

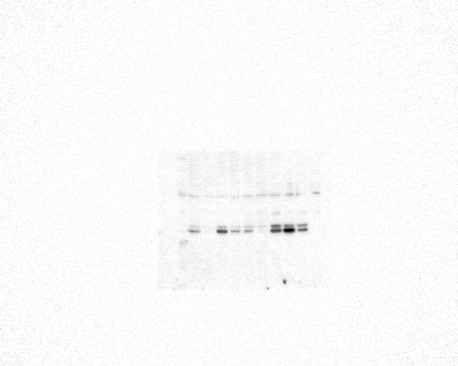

Supplement: Figure 4—source data 4. [file elife-83159-fig4-data4.zip › Figure 4-source data 4/p-JNK Figure 4-source data 4/Versteeg 2021-08-13 14h42m12s 159.300s(Chemiluminescence).raw16.tif]

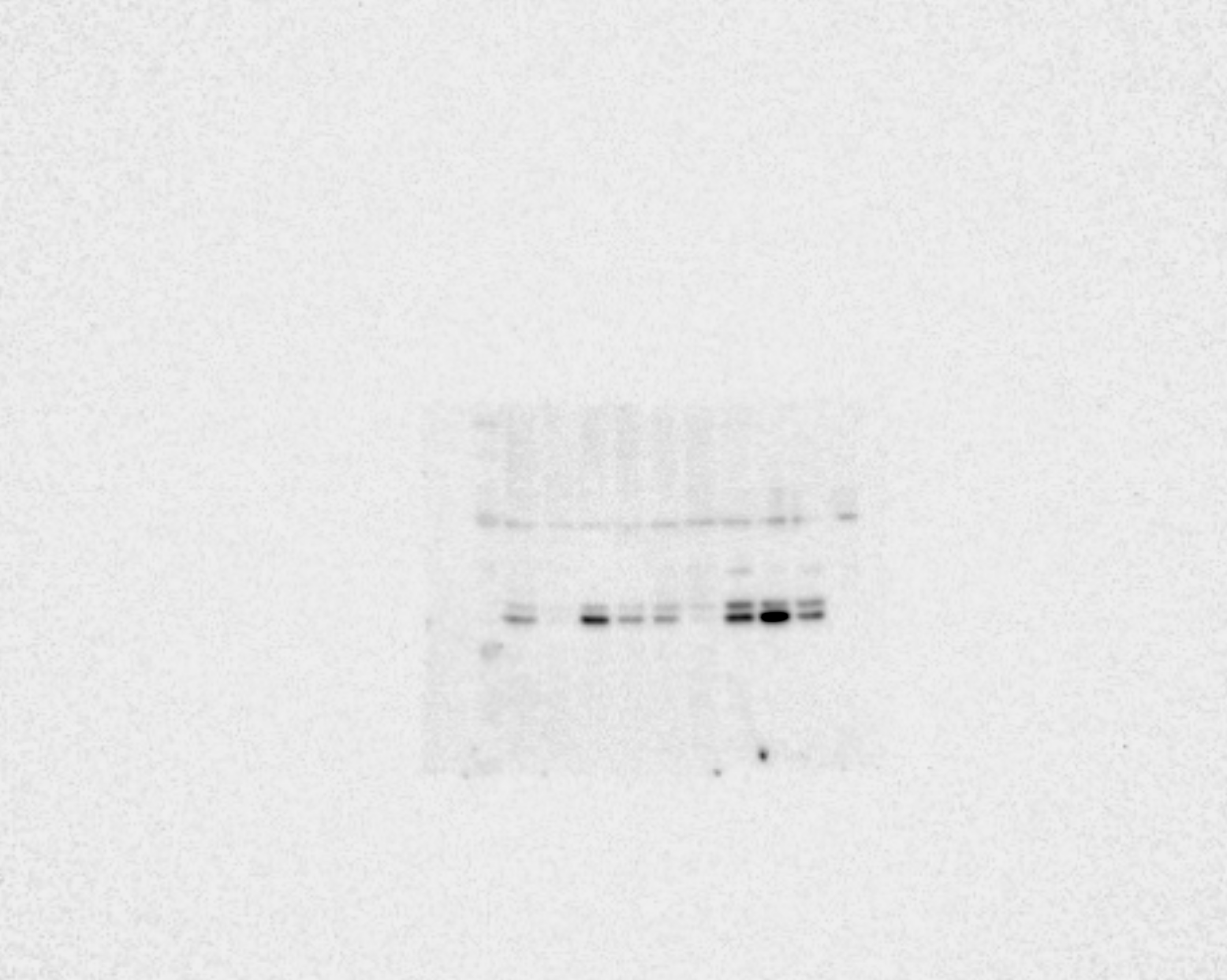

Supplement: Figure 4—source data 4. [file elife-83159-fig4-data4.zip › Figure 4-source data 4/p-JNK Figure 4-source data 4/Versteeg 2021-08-13 14h42m12s 159.300s(Chemiluminescence).tif]

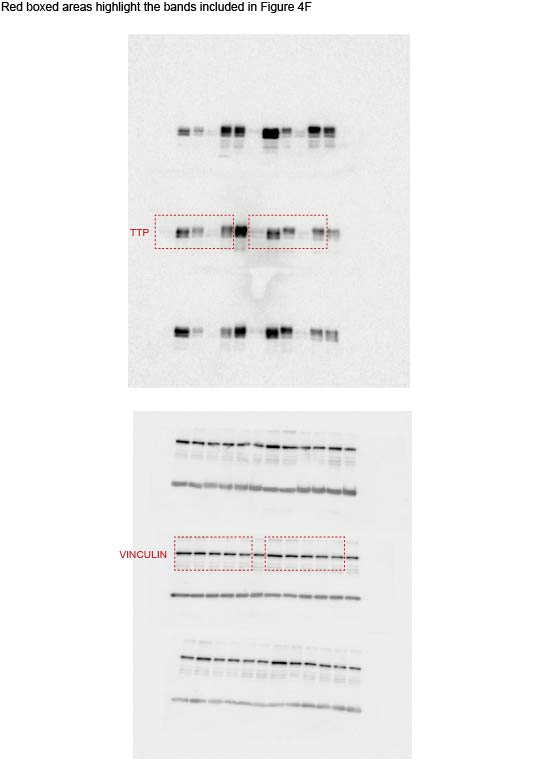

Supplement: Figure 4—source data 5. [file elife-83159-fig4-data5.zip › Figure 4-source data 5.jpg]

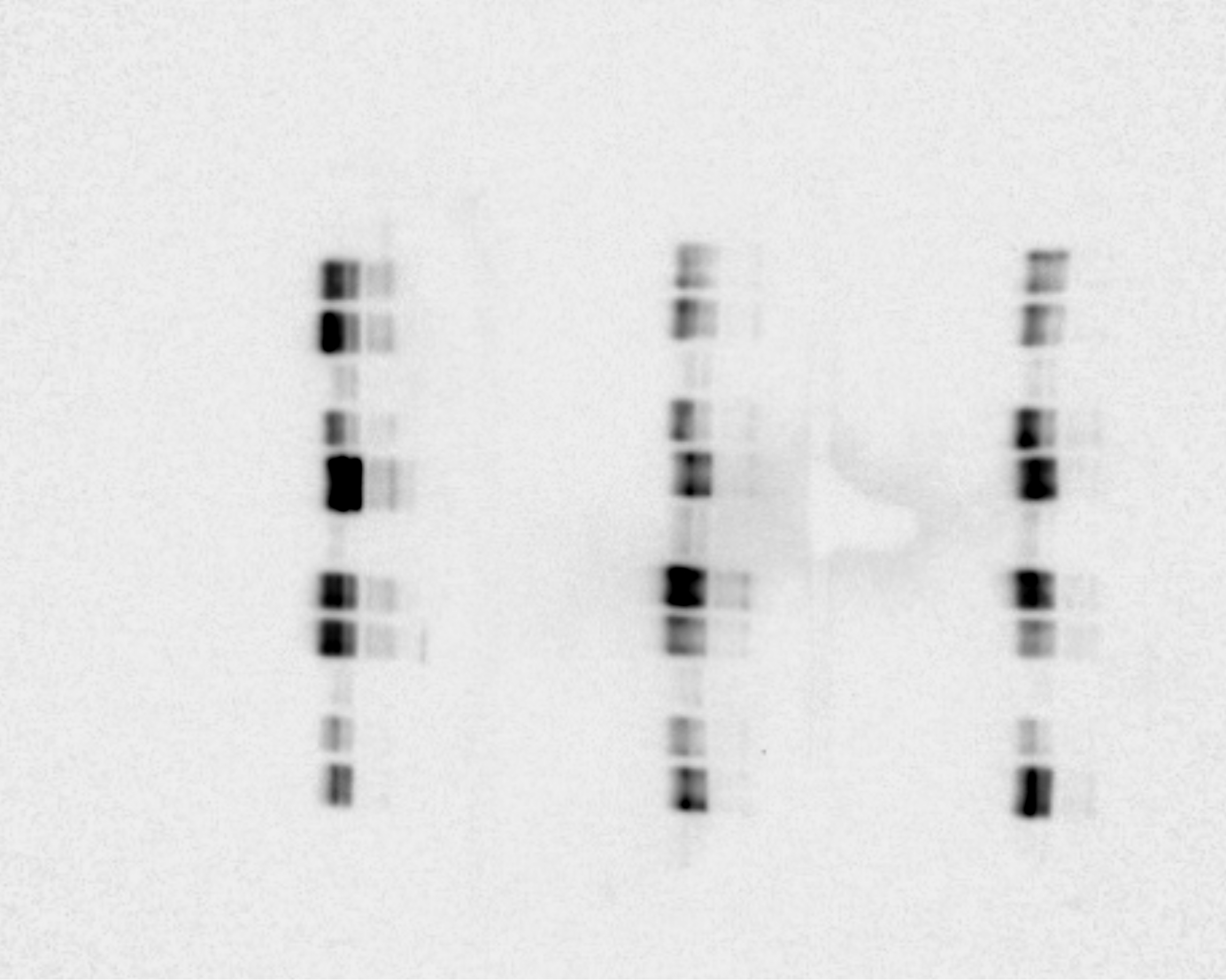

Supplement: Figure 4—source data 5. [file elife-83159-fig4-data5.zip › TTP Figure 4-source data 5/Versteeg 2023-01-26 11h28m58s 13.033s(Chemiluminescence).jpg]

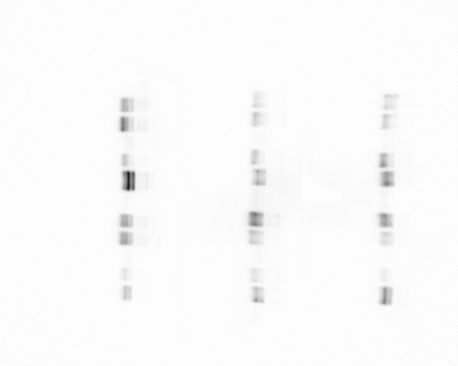

Supplement: Figure 4—source data 5. [file elife-83159-fig4-data5.zip › TTP Figure 4-source data 5/Versteeg 2023-01-26 11h28m58s 13.033s(Chemiluminescence).raw16.tif]

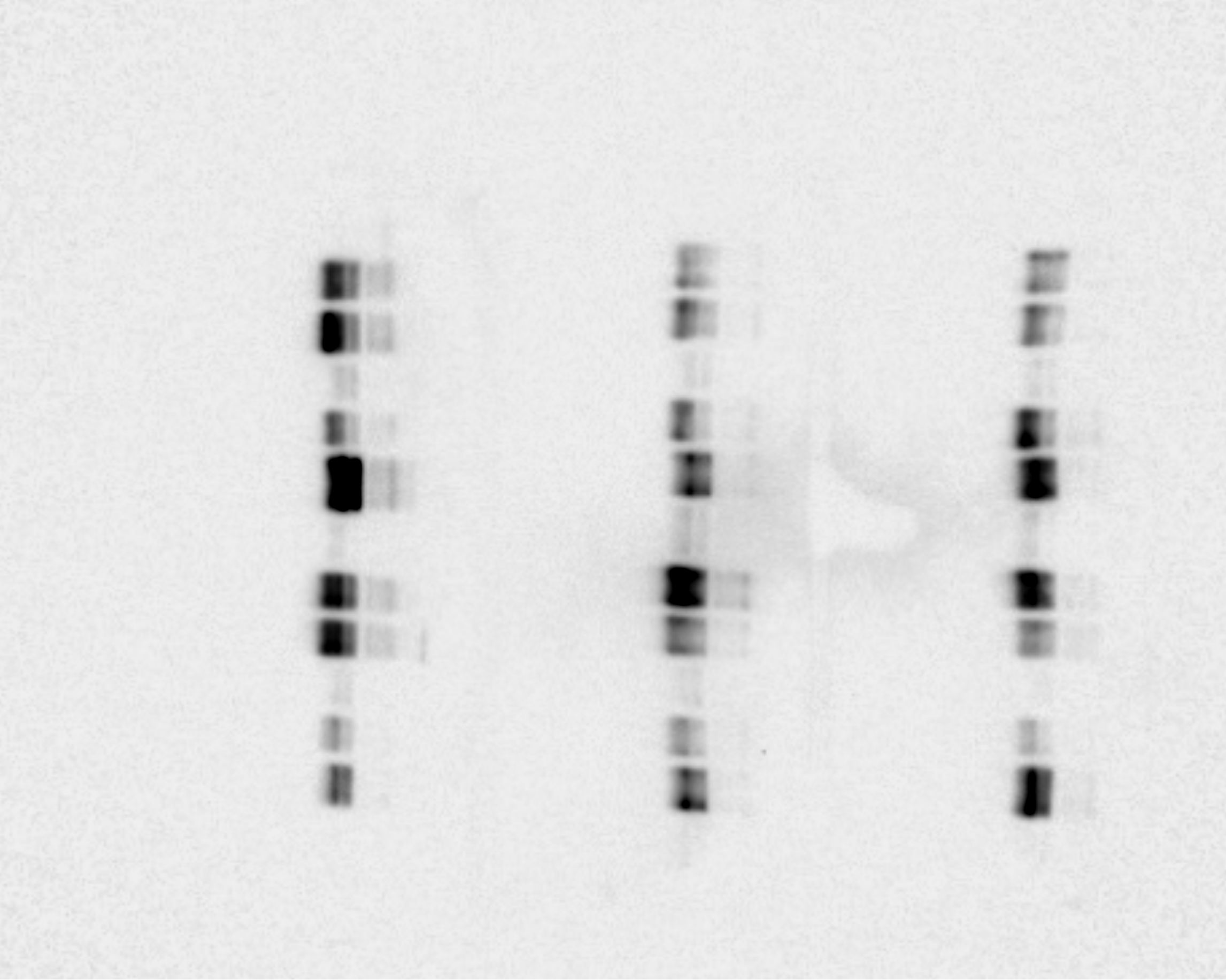

Supplement: Figure 4—source data 5. [file elife-83159-fig4-data5.zip › TTP Figure 4-source data 5/Versteeg 2023-01-26 11h28m58s 13.033s(Chemiluminescence).tif]

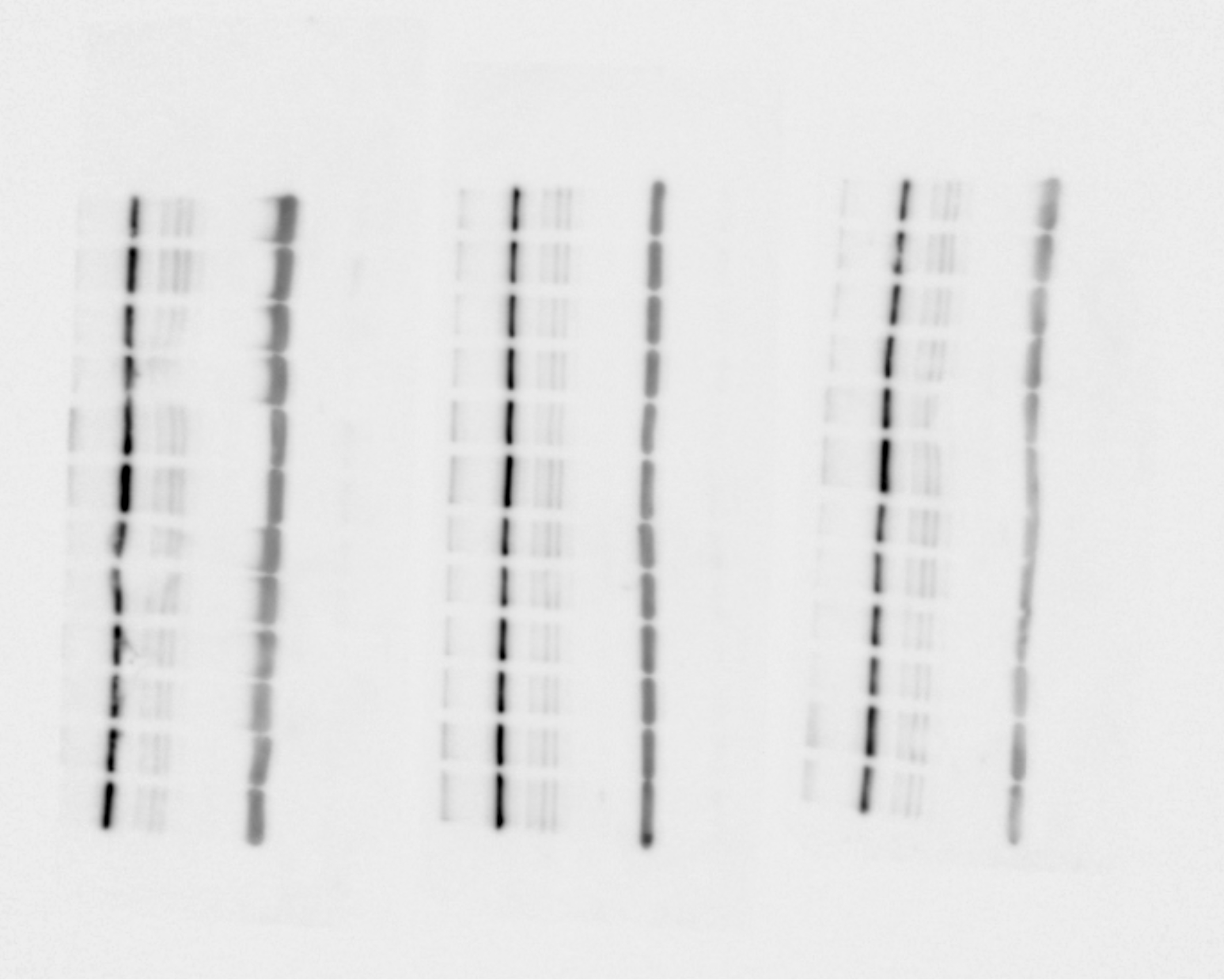

Supplement: Figure 4—source data 5. [file elife-83159-fig4-data5.zip › Vinculin Figure 4-source data 5/Versteeg 2023-01-27 12h50m24s 4.016s(Chemiluminescence).jpg]

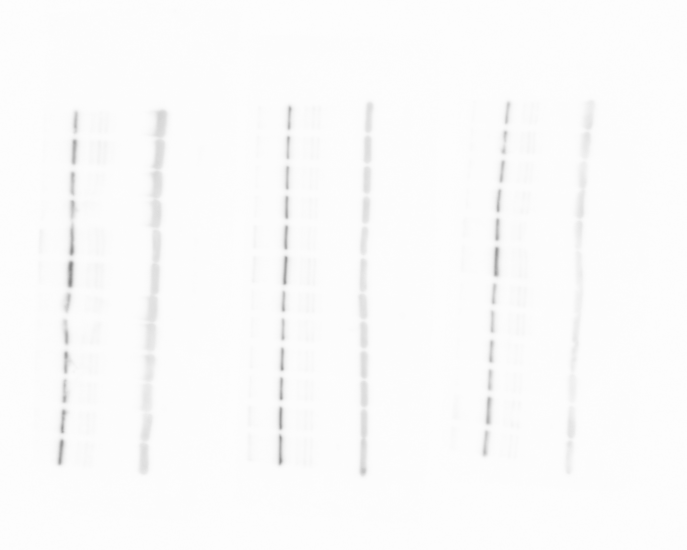

Supplement: Figure 4—source data 5. [file elife-83159-fig4-data5.zip › Vinculin Figure 4-source data 5/Versteeg 2023-01-27 12h50m24s 4.016s(Chemiluminescence).raw16.tif]

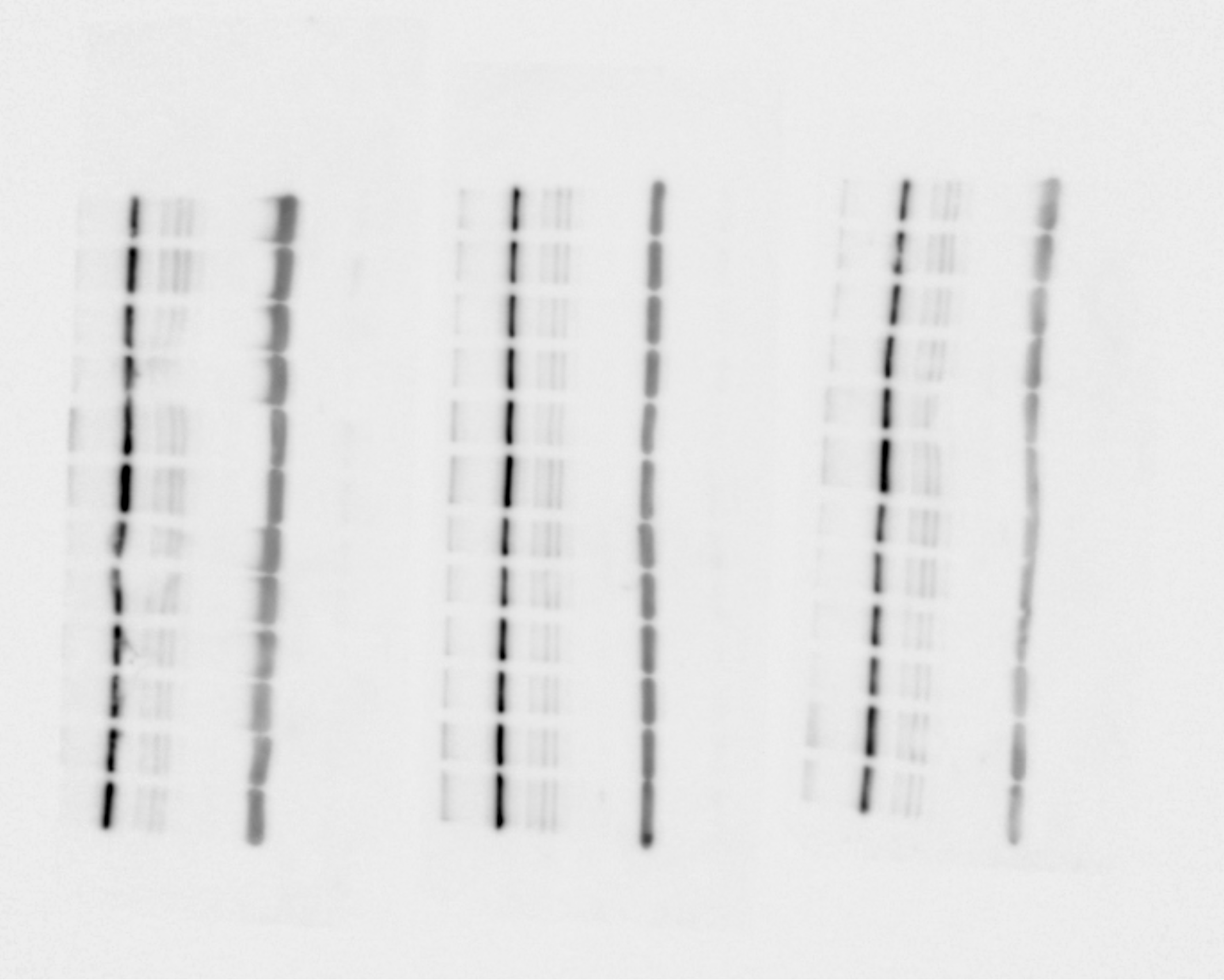

Supplement: Figure 4—source data 5. [file elife-83159-fig4-data5.zip › Vinculin Figure 4-source data 5/Versteeg 2023-01-27 12h50m24s 4.016s(Chemiluminescence).tif]

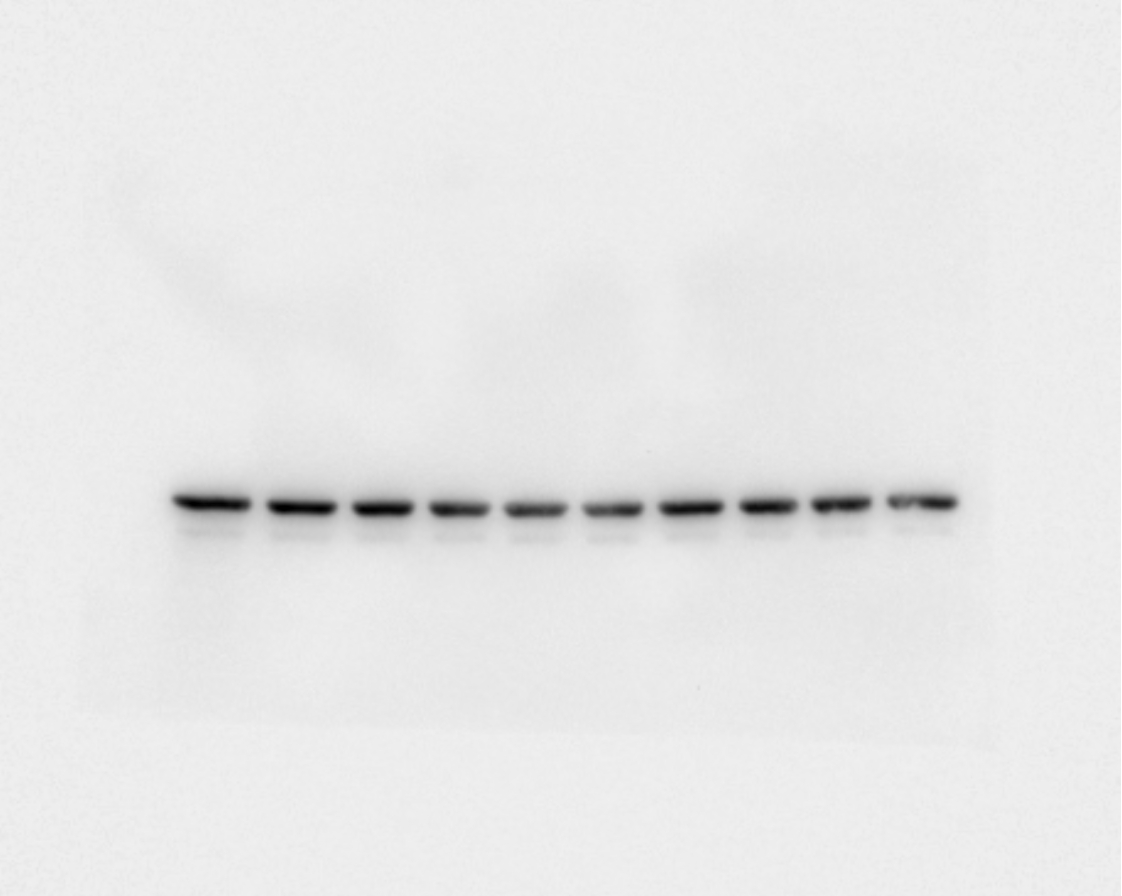

Supplement: Figure 4—figure supplement 1—source data 1. [file elife-83159-fig4-figsupp1-data1.zip › ACTIN Figure 4-figure supplement 1-source data 1/Versteeg 2022-03-28 19h45m37s 4.024s(Chemiluminescence).jpg]

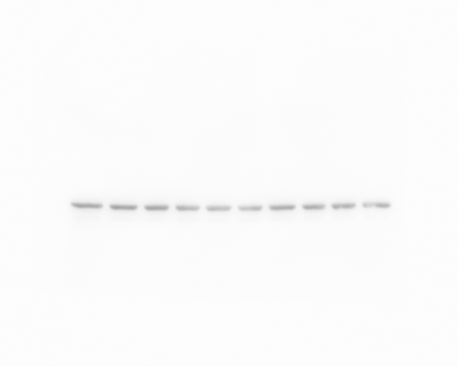

Supplement: Figure 4—figure supplement 1—source data 1. [file elife-83159-fig4-figsupp1-data1.zip › ACTIN Figure 4-figure supplement 1-source data 1/Versteeg 2022-03-28 19h45m37s 4.024s(Chemiluminescence).raw16.tif]

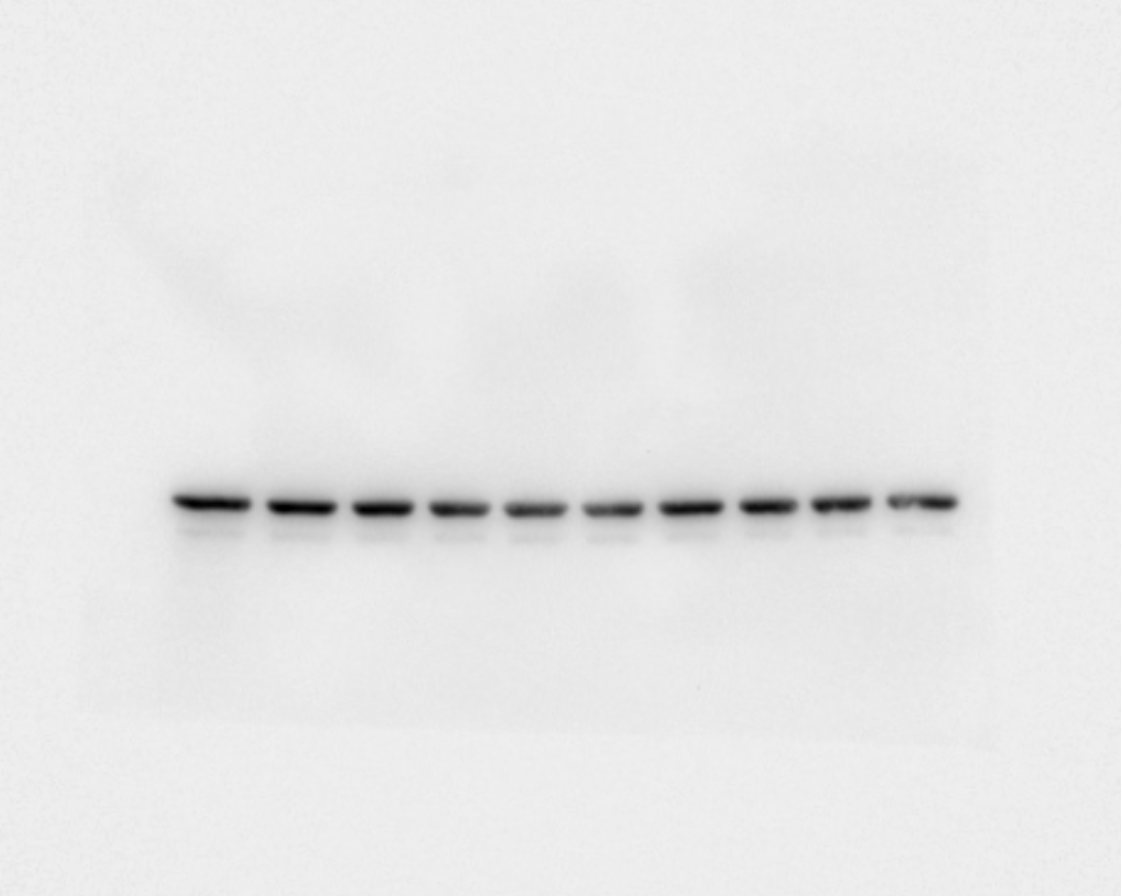

Supplement: Figure 4—figure supplement 1—source data 1. [file elife-83159-fig4-figsupp1-data1.zip › ACTIN Figure 4-figure supplement 1-source data 1/Versteeg 2022-03-28 19h45m37s 4.024s(Chemiluminescence).tif]

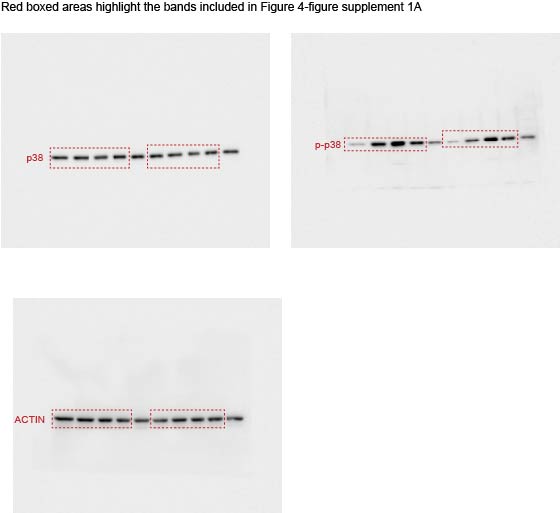

Supplement: Figure 4—figure supplement 1—source data 1. [file elife-83159-fig4-figsupp1-data1.zip › Figure 4-figure supplement 1-source data 1.jpg]

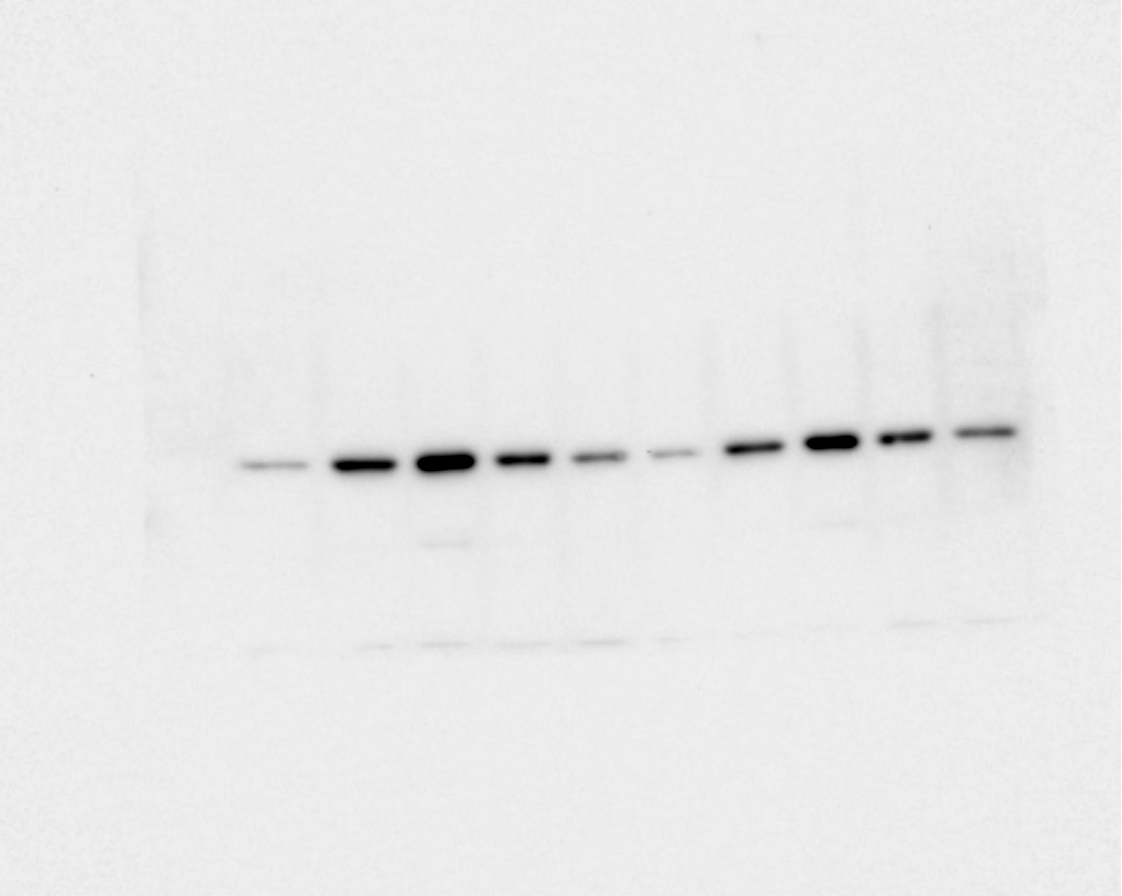

Supplement: Figure 4—figure supplement 1—source data 1. [file elife-83159-fig4-figsupp1-data1.zip › p-p38 Figure 4-figure supplement 1-source data 1/Versteeg 2022-03-16 14h06m44s 16.201s(Chemiluminescence).jpg]

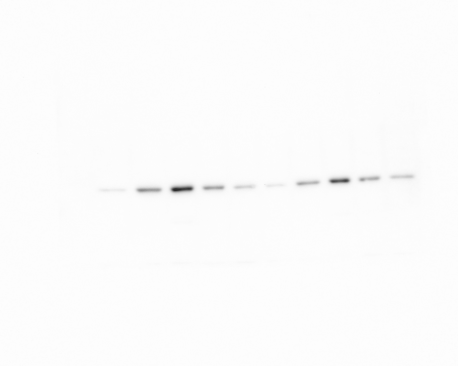

Supplement: Figure 4—figure supplement 1—source data 1. [file elife-83159-fig4-figsupp1-data1.zip › p-p38 Figure 4-figure supplement 1-source data 1/Versteeg 2022-03-16 14h06m44s 16.201s(Chemiluminescence).raw16.tif]

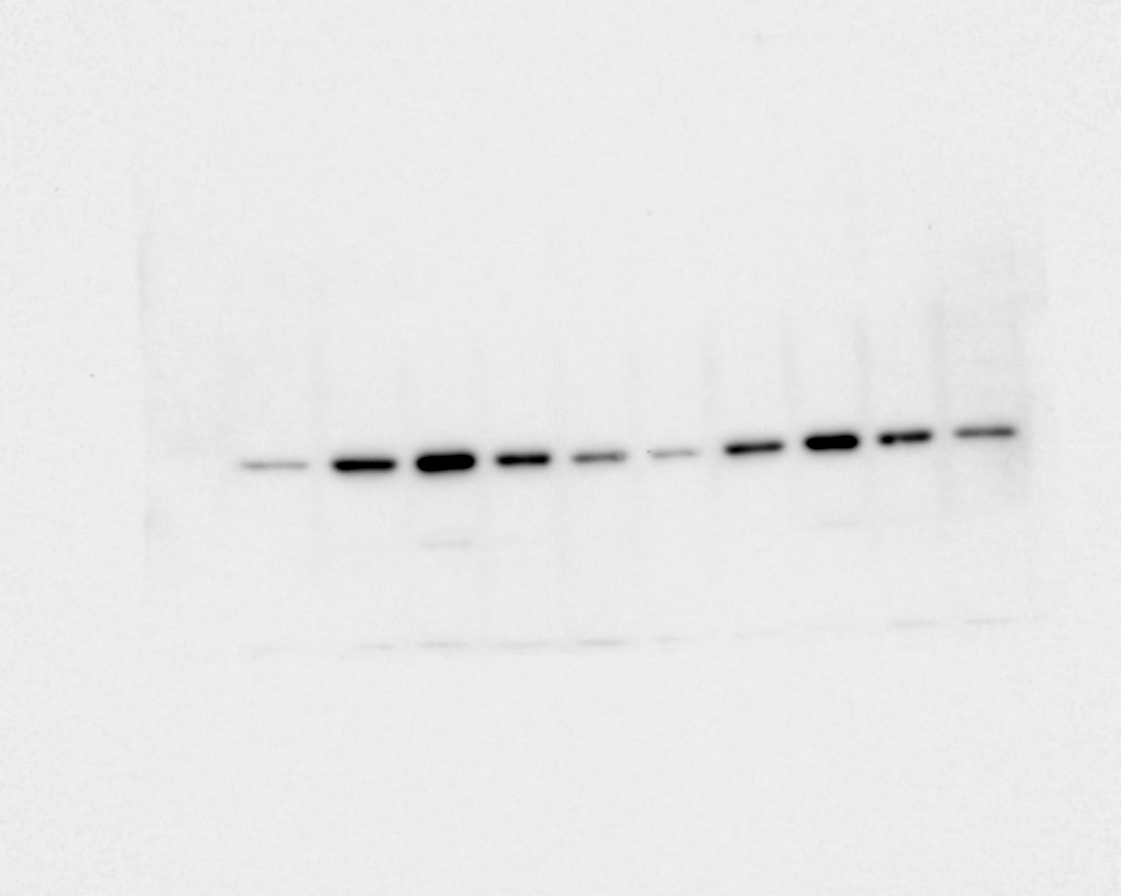

Supplement: Figure 4—figure supplement 1—source data 1. [file elife-83159-fig4-figsupp1-data1.zip › p-p38 Figure 4-figure supplement 1-source data 1/Versteeg 2022-03-16 14h06m44s 16.201s(Chemiluminescence).tif]

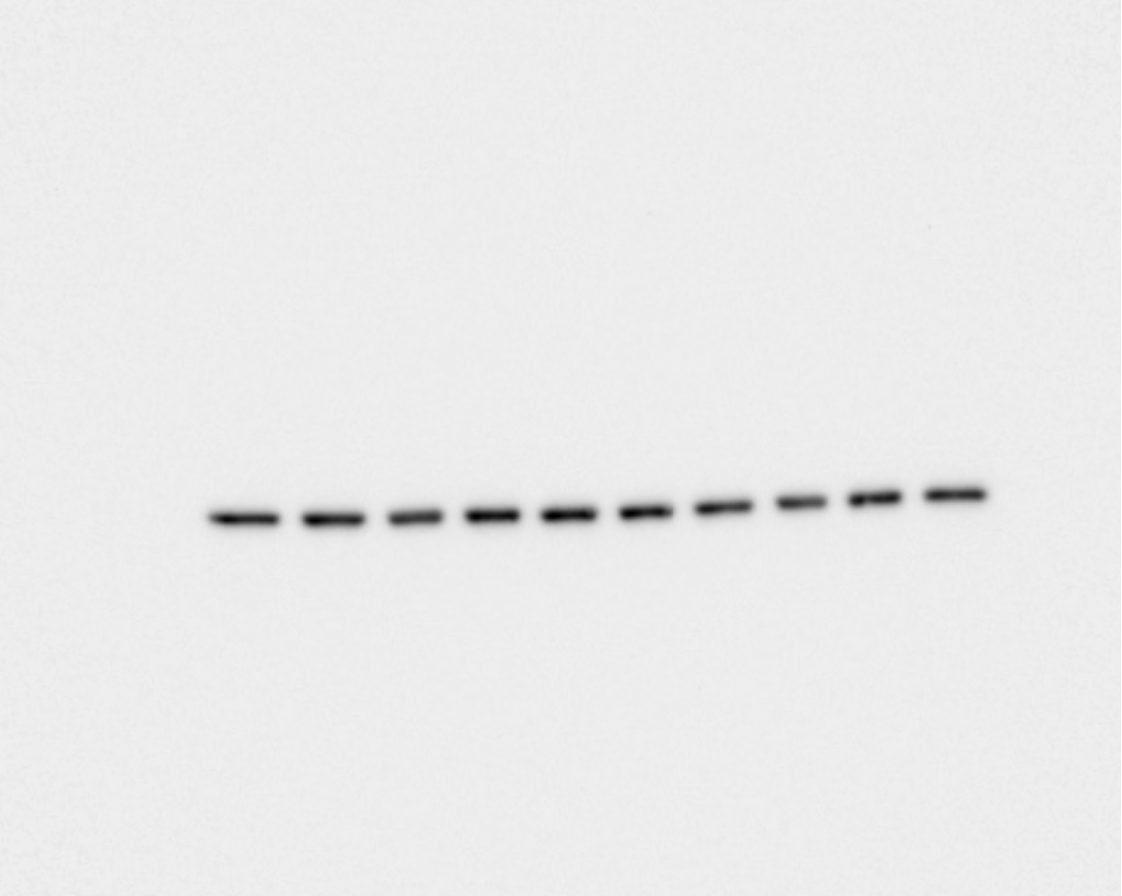

Supplement: Figure 4—figure supplement 1—source data 1. [file elife-83159-fig4-figsupp1-data1.zip › p38 Figure 4-figure supplement 1-source data 1/Versteeg 2022-03-23 15h27m58s 11.134s(Chemiluminescence).jpg]

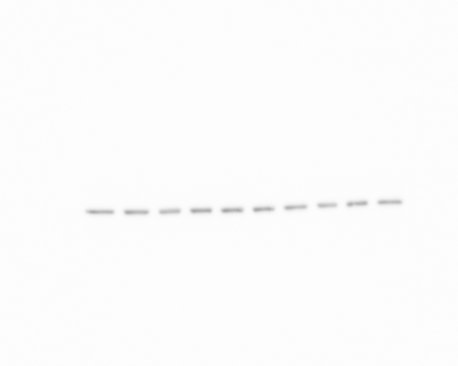

Supplement: Figure 4—figure supplement 1—source data 1. [file elife-83159-fig4-figsupp1-data1.zip › p38 Figure 4-figure supplement 1-source data 1/Versteeg 2022-03-23 15h27m58s 11.134s(Chemiluminescence).raw16.tif]

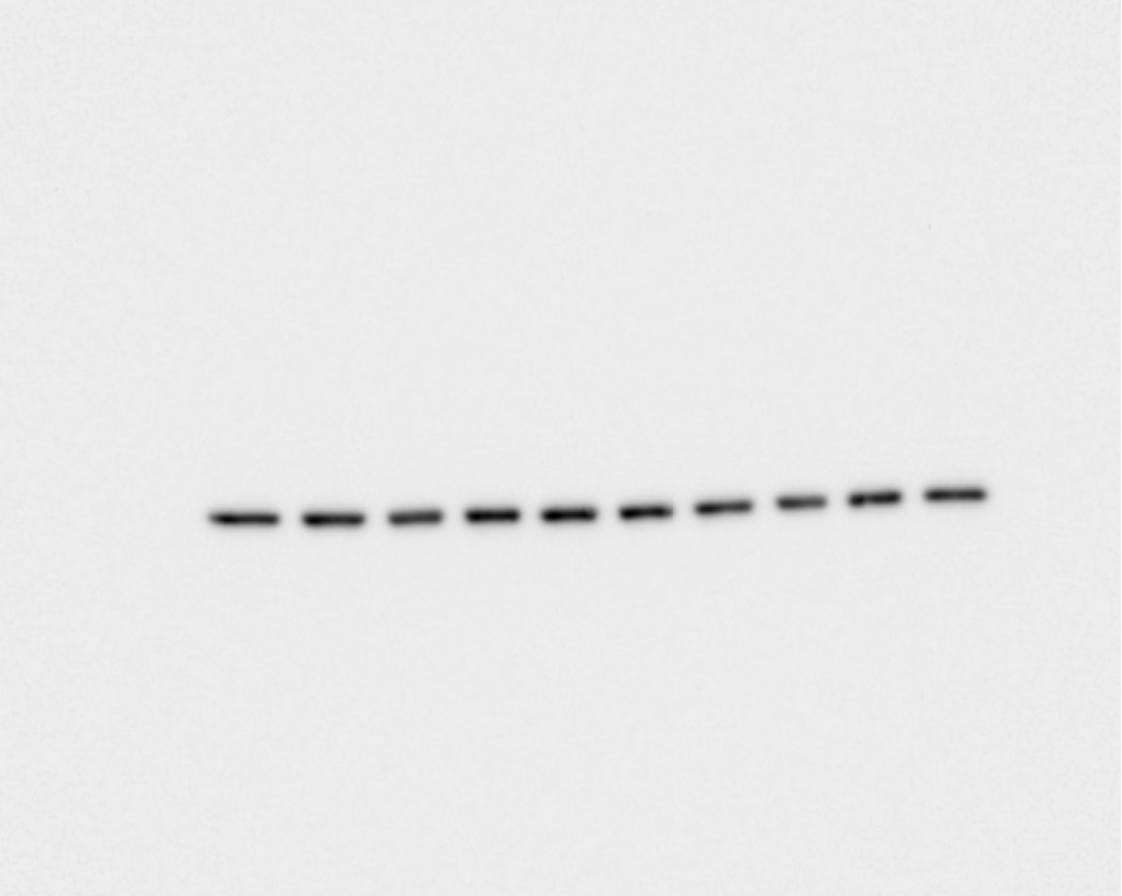

Supplement: Figure 4—figure supplement 1—source data 1. [file elife-83159-fig4-figsupp1-data1.zip › p38 Figure 4-figure supplement 1-source data 1/Versteeg 2022-03-23 15h27m58s 11.134s(Chemiluminescence).tif]

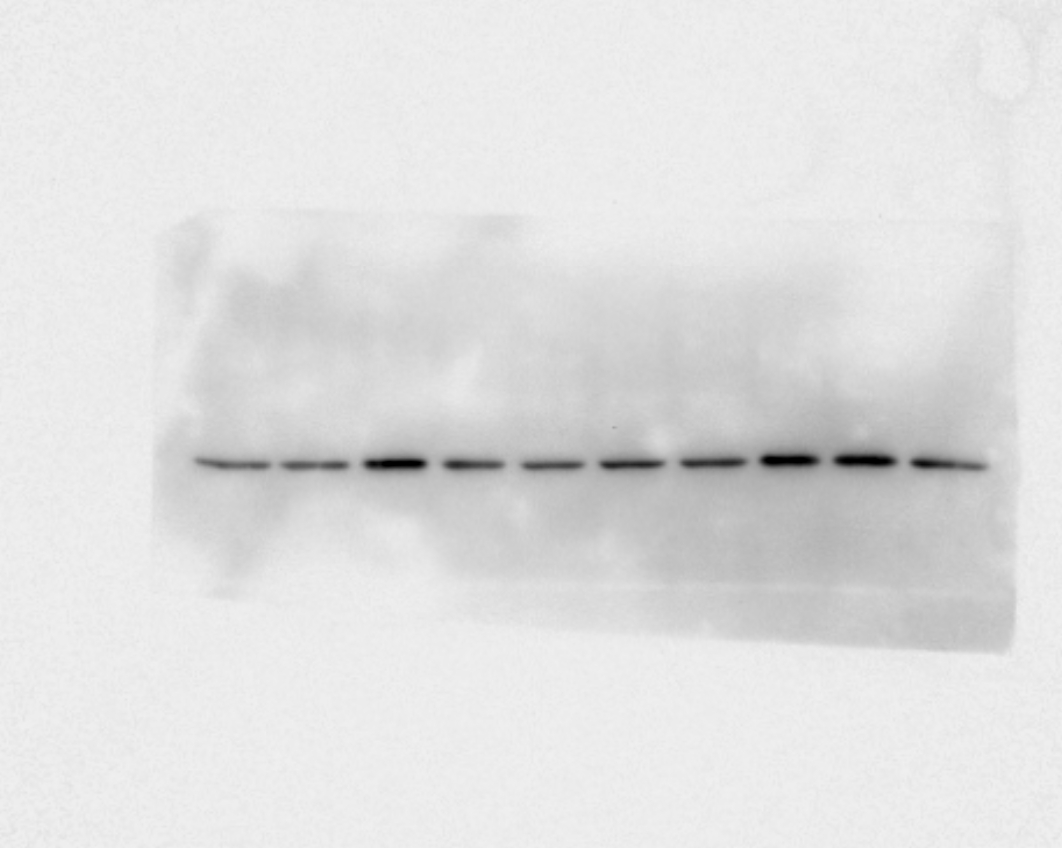

Supplement: Figure 4—figure supplement 1—source data 2. [file elife-83159-fig4-figsupp1-data2.zip › ACTIN Figure 4-figure supplement 1-source data 2/Versteeg 2022-03-28 19h10m23s 13.204s(Chemiluminescence).jpg]

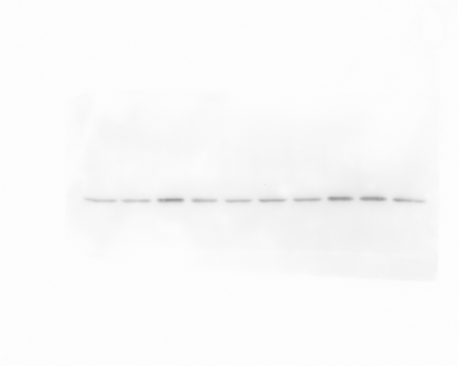

Supplement: Figure 4—figure supplement 1—source data 2. [file elife-83159-fig4-figsupp1-data2.zip › ACTIN Figure 4-figure supplement 1-source data 2/Versteeg 2022-03-28 19h10m23s 13.204s(Chemiluminescence).raw16.tif]

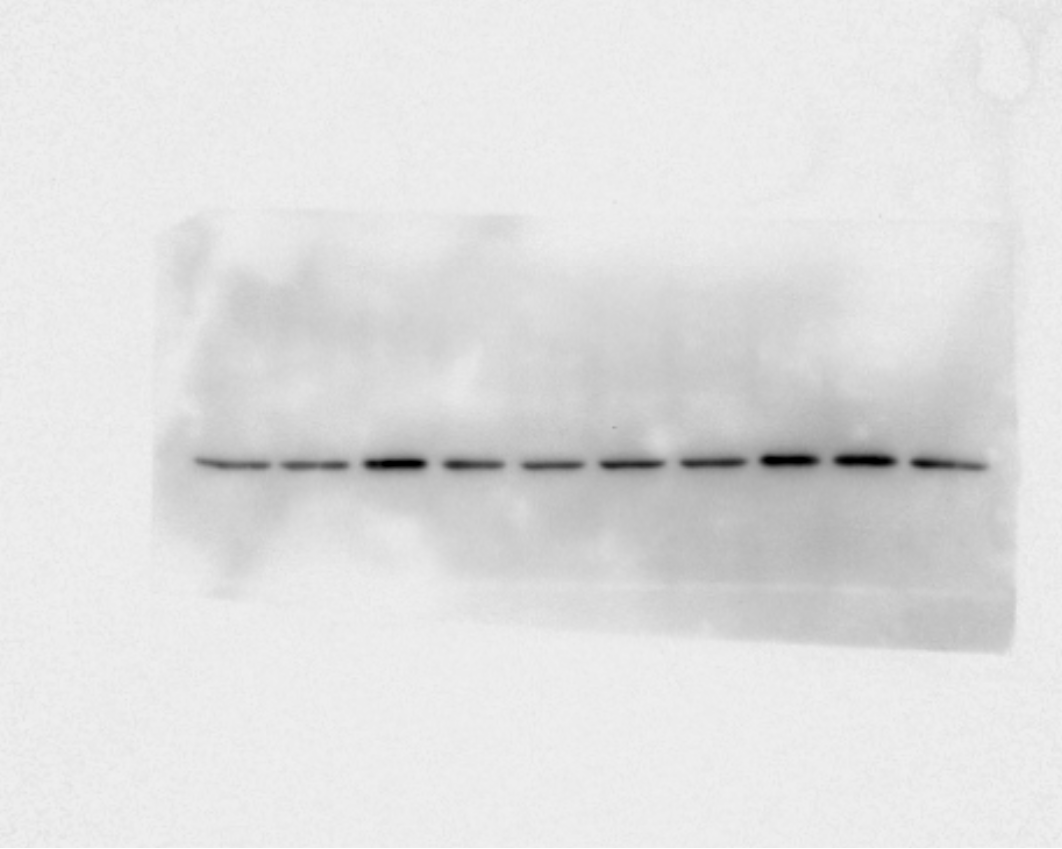

Supplement: Figure 4—figure supplement 1—source data 2. [file elife-83159-fig4-figsupp1-data2.zip › ACTIN Figure 4-figure supplement 1-source data 2/Versteeg 2022-03-28 19h10m23s 13.204s(Chemiluminescence).tif]

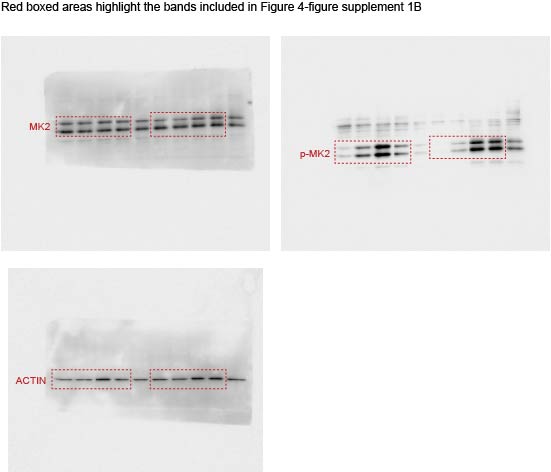

Supplement: Figure 4—figure supplement 1—source data 2. [file elife-83159-fig4-figsupp1-data2.zip › Figure 4-figure supplement 1-source data 2.jpg]

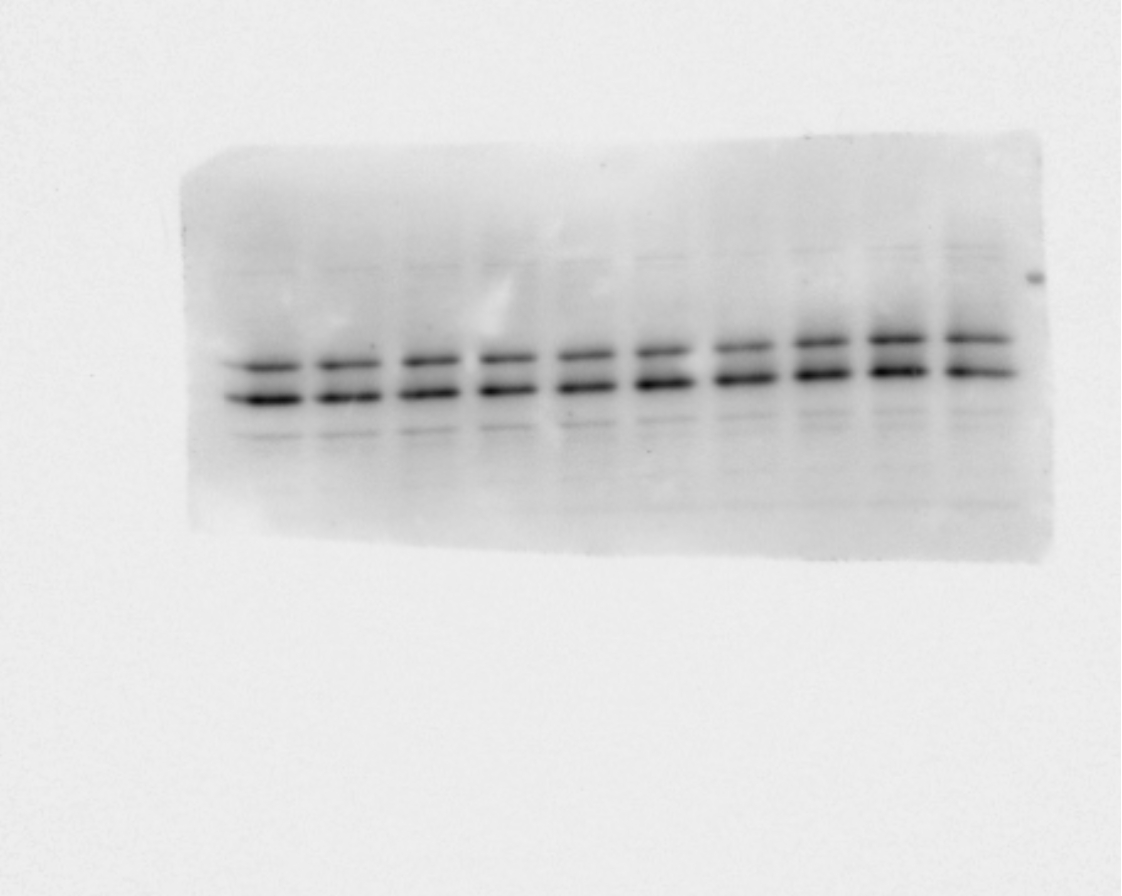

Supplement: Figure 4—figure supplement 1—source data 2. [file elife-83159-fig4-figsupp1-data2.zip › MK2 Figure 4-figure supplement 1-source data 2/Versteeg 2022-03-23 17h27m05s 11.134s(Chemiluminescence).jpg]

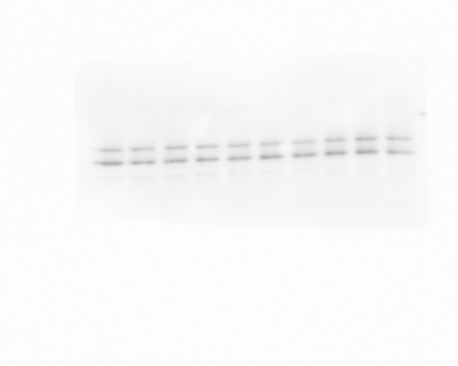

Supplement: Figure 4—figure supplement 1—source data 2. [file elife-83159-fig4-figsupp1-data2.zip › MK2 Figure 4-figure supplement 1-source data 2/Versteeg 2022-03-23 17h27m05s 11.134s(Chemiluminescence).raw16.tif]

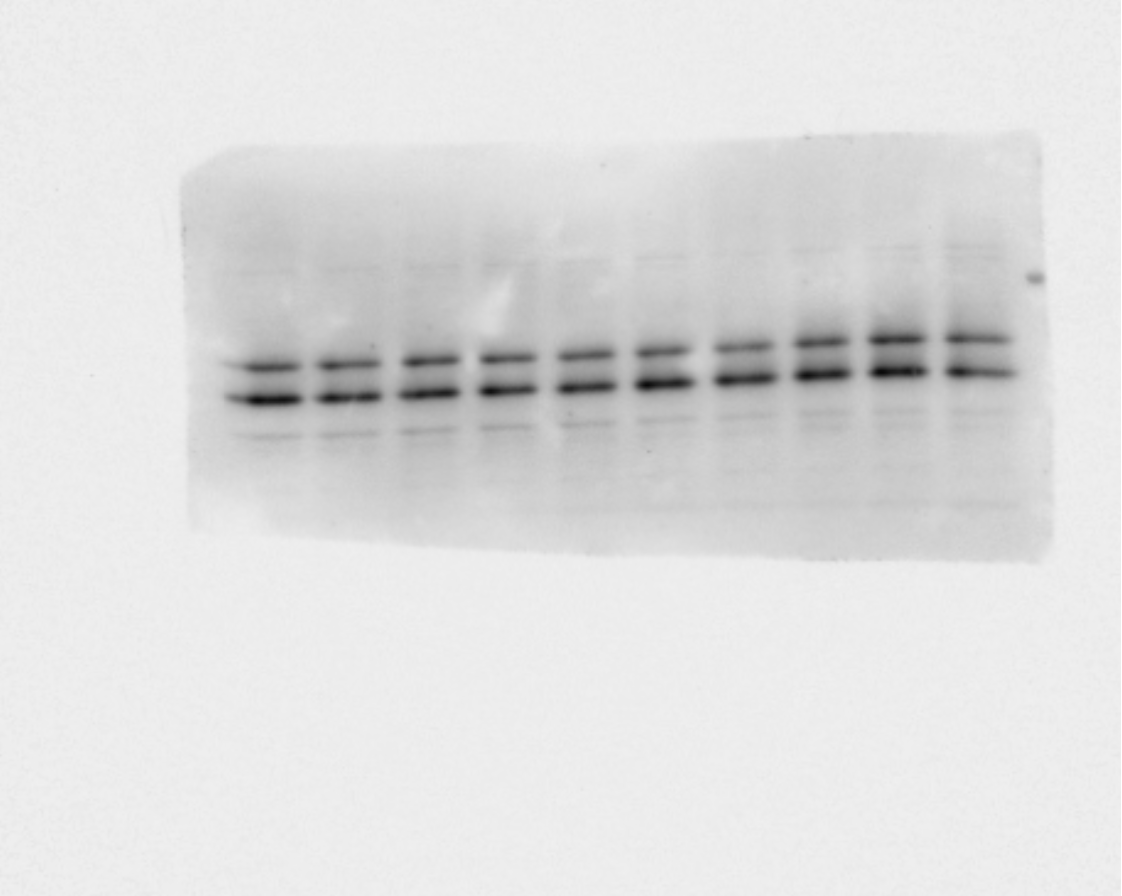

Supplement: Figure 4—figure supplement 1—source data 2. [file elife-83159-fig4-figsupp1-data2.zip › MK2 Figure 4-figure supplement 1-source data 2/Versteeg 2022-03-23 17h27m05s 11.134s(Chemiluminescence).tif]

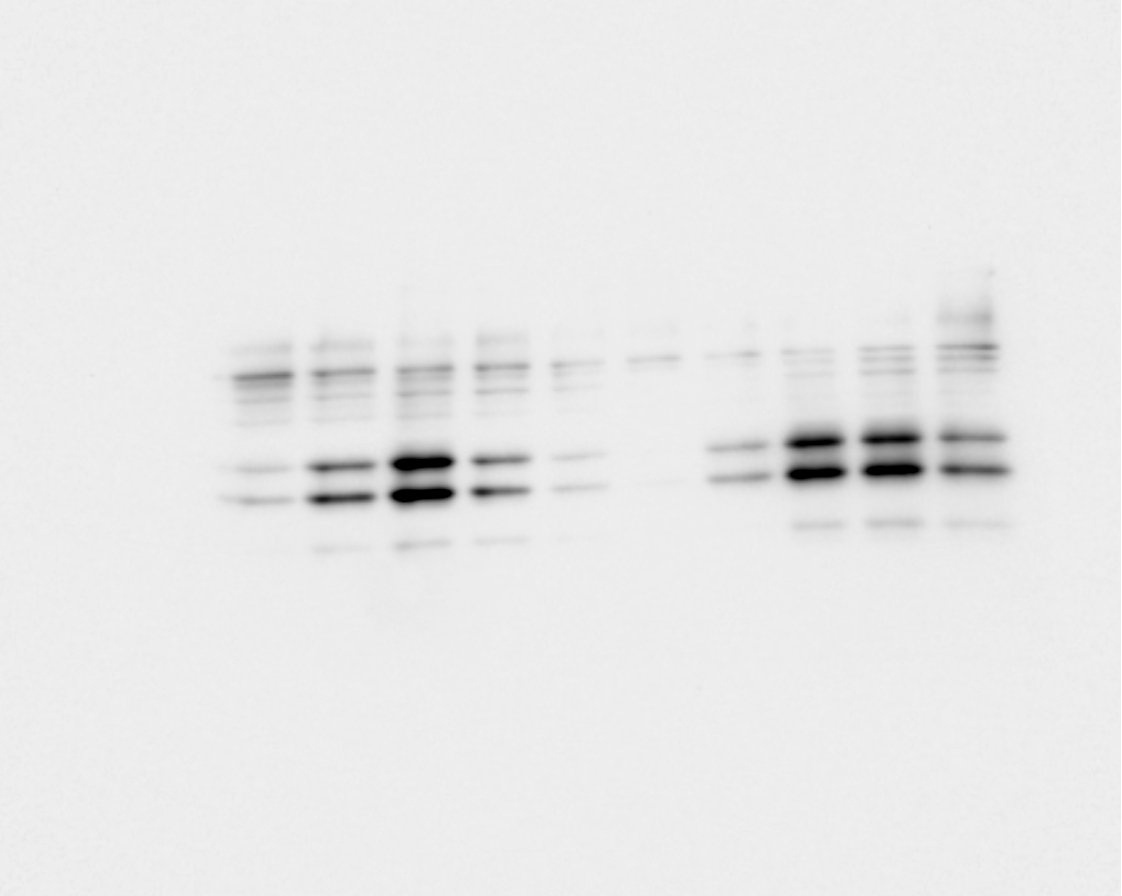

Supplement: Figure 4—figure supplement 1—source data 2. [file elife-83159-fig4-figsupp1-data2.zip › p-MK2 Figure 4-figure supplement 1-source data 2/Versteeg 2022-03-16 14h44m42s 6.067s(Chemiluminescence).jpg]

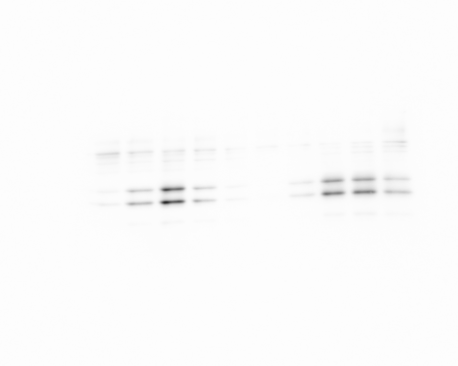

Supplement: Figure 4—figure supplement 1—source data 2. [file elife-83159-fig4-figsupp1-data2.zip › p-MK2 Figure 4-figure supplement 1-source data 2/Versteeg 2022-03-16 14h44m42s 6.067s(Chemiluminescence).raw16.tif]

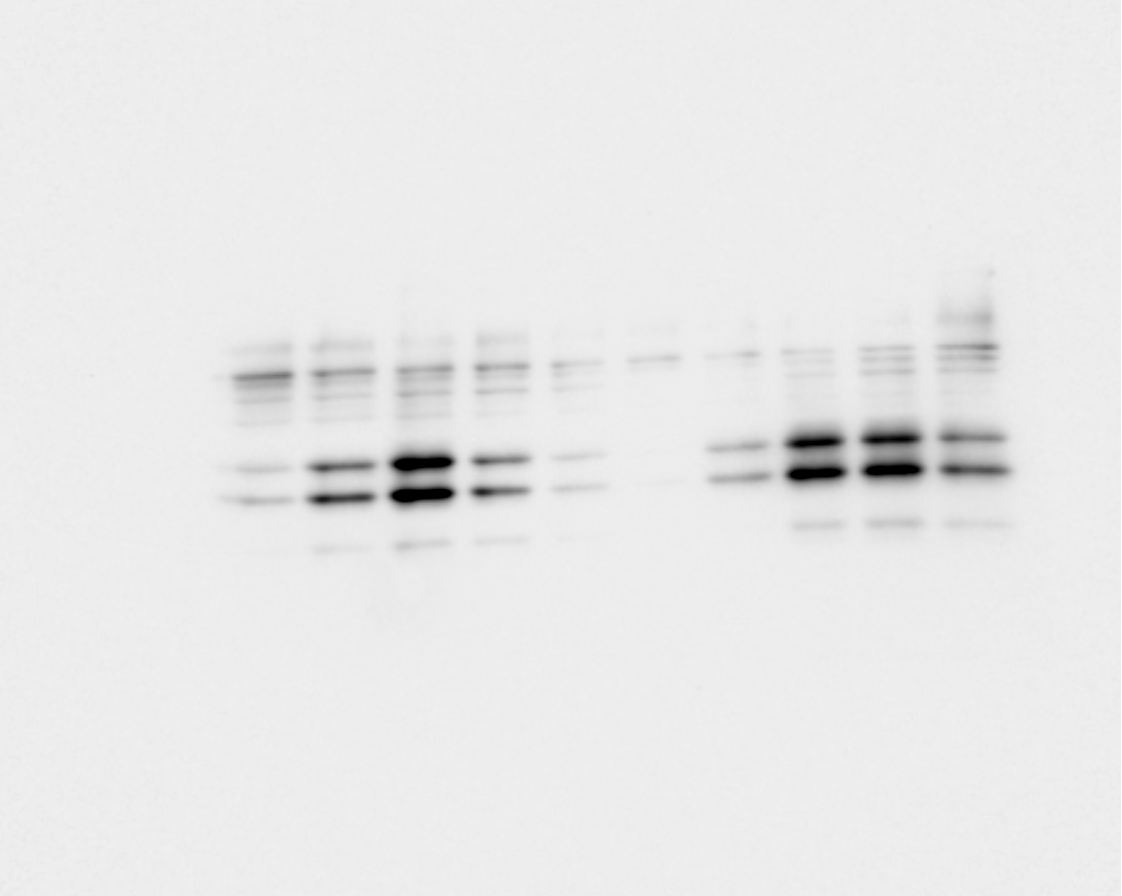

Supplement: Figure 4—figure supplement 1—source data 2. [file elife-83159-fig4-figsupp1-data2.zip › p-MK2 Figure 4-figure supplement 1-source data 2/Versteeg 2022-03-16 14h44m42s 6.067s(Chemiluminescence).tif]

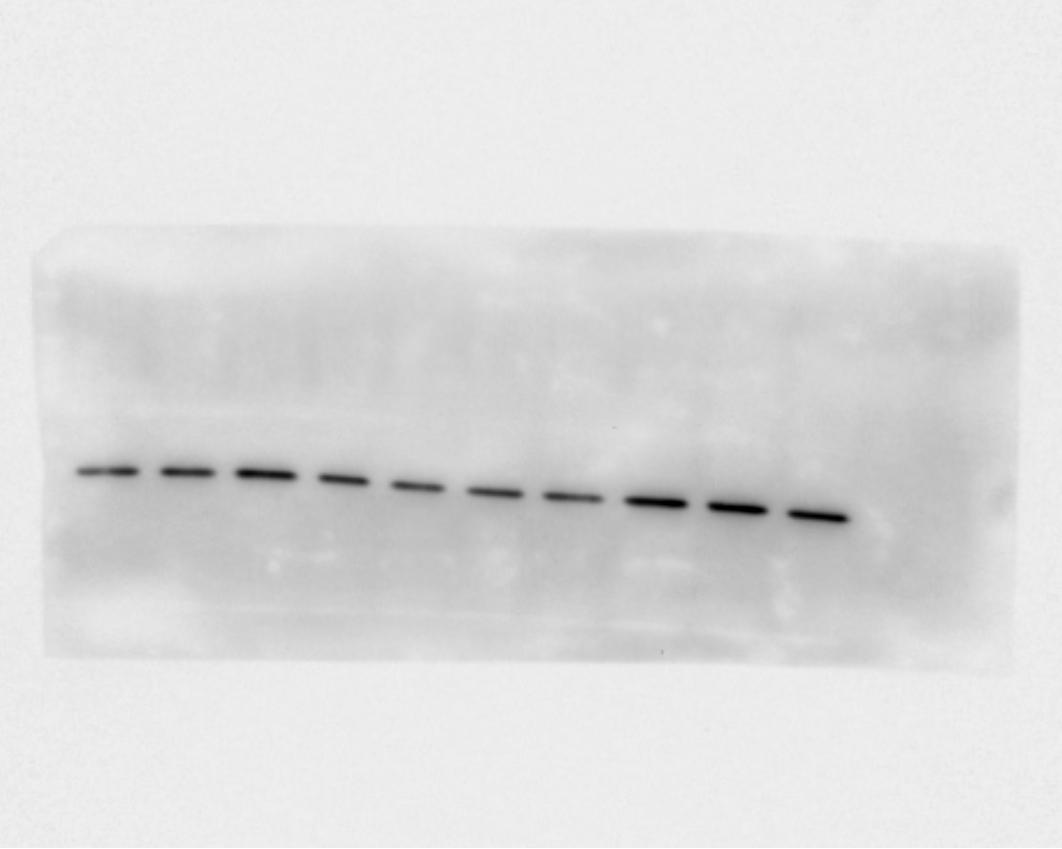

Supplement: Figure 4—figure supplement 1—source data 3. [file elife-83159-fig4-figsupp1-data3.zip › ACTIN Figure 4-figure supplement 1-source data 3/Versteeg 2022-03-28 19h05m49s 7.102s(Chemiluminescence).jpg]

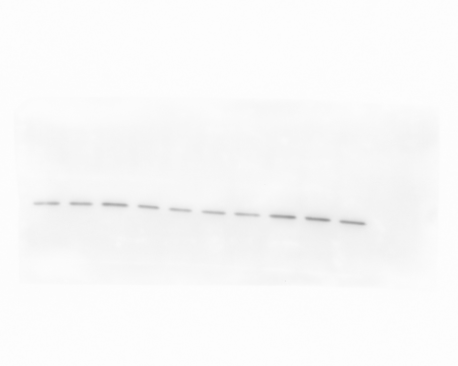

Supplement: Figure 4—figure supplement 1—source data 3. [file elife-83159-fig4-figsupp1-data3.zip › ACTIN Figure 4-figure supplement 1-source data 3/Versteeg 2022-03-28 19h05m49s 7.102s(Chemiluminescence).raw16.tif]

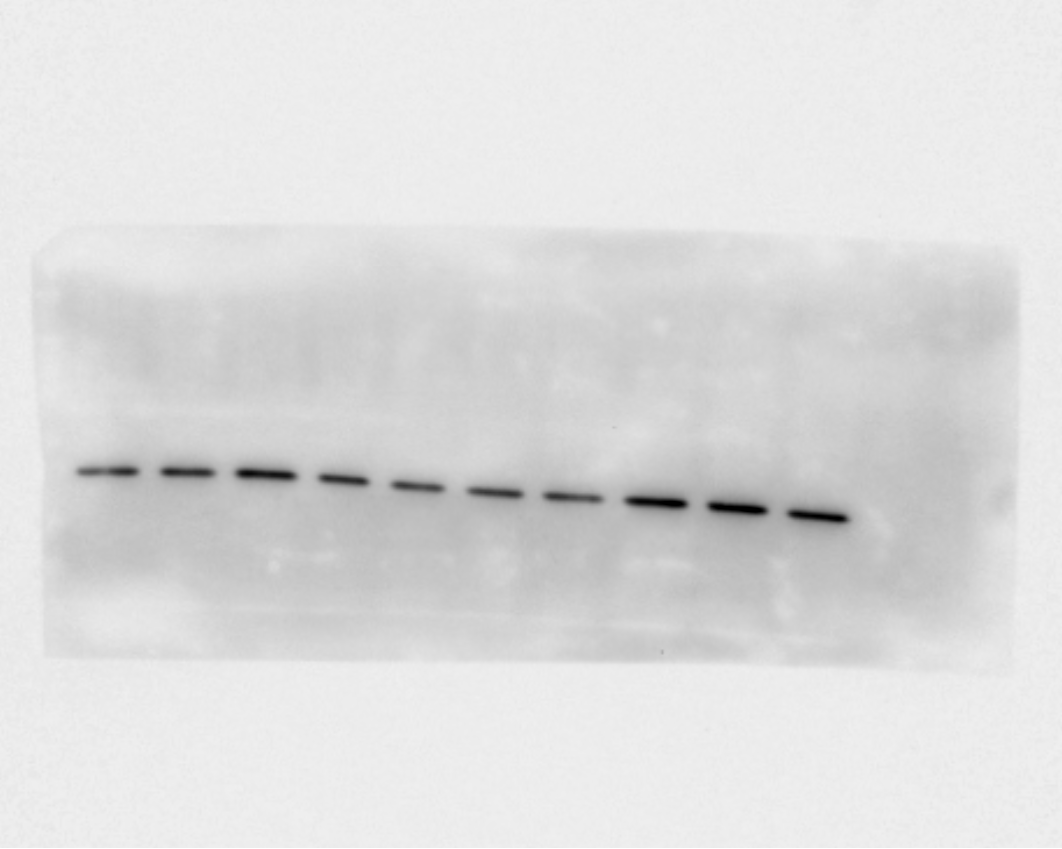

Supplement: Figure 4—figure supplement 1—source data 3. [file elife-83159-fig4-figsupp1-data3.zip › ACTIN Figure 4-figure supplement 1-source data 3/Versteeg 2022-03-28 19h05m49s 7.102s(Chemiluminescence).tif]

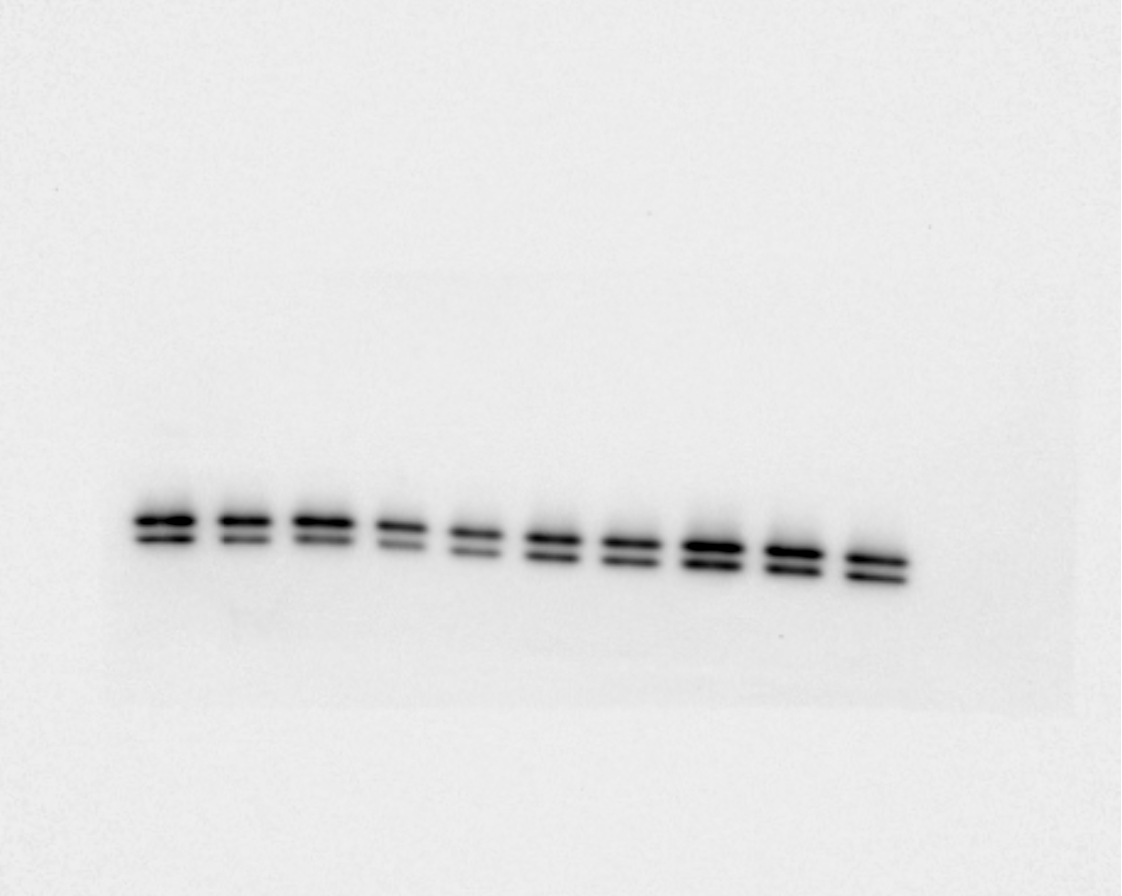

Supplement: Figure 4—figure supplement 1—source data 3. [file elife-83159-fig4-figsupp1-data3.zip › ERK Figure 4-figure supplement 1-source data 3/Versteeg 2022-03-23 14h41m54s 16.201s(Chemiluminescence).jpg]

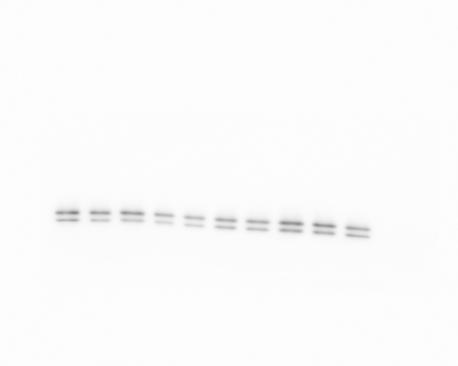

Supplement: Figure 4—figure supplement 1—source data 3. [file elife-83159-fig4-figsupp1-data3.zip › ERK Figure 4-figure supplement 1-source data 3/Versteeg 2022-03-23 14h41m54s 16.201s(Chemiluminescence).raw16.tif]

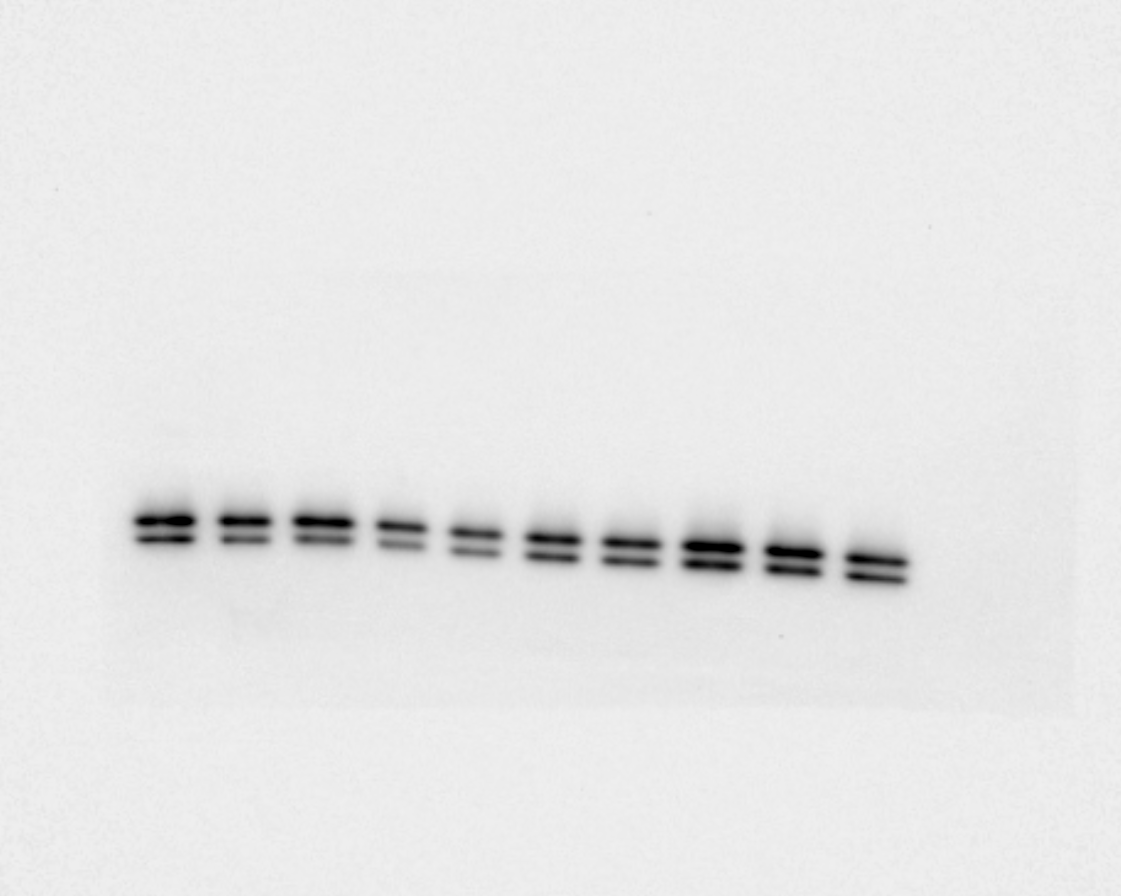

Supplement: Figure 4—figure supplement 1—source data 3. [file elife-83159-fig4-figsupp1-data3.zip › ERK Figure 4-figure supplement 1-source data 3/Versteeg 2022-03-23 14h41m54s 16.201s(Chemiluminescence).tif]

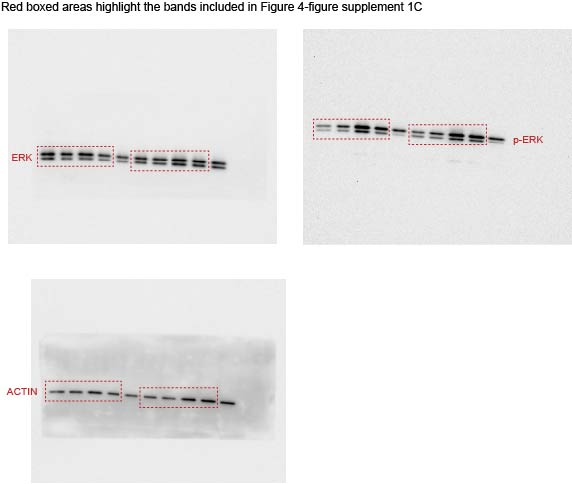

Supplement: Figure 4—figure supplement 1—source data 3. [file elife-83159-fig4-figsupp1-data3.zip › Figure 4-figure supplement 1-source data 3.jpg]

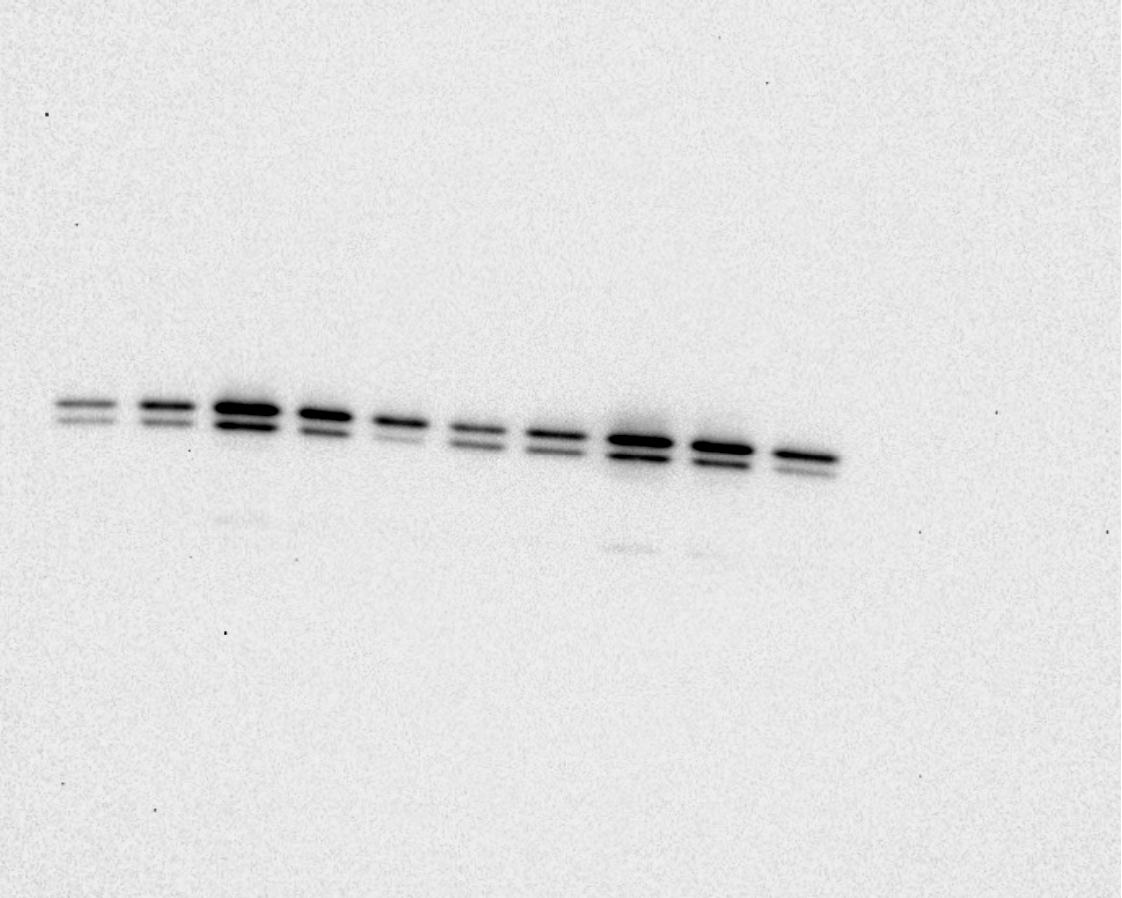

Supplement: Figure 4—figure supplement 1—source data 3. [file elife-83159-fig4-figsupp1-data3.zip › p-ERK Figure 4-figure supplement 1-source data 3/Run 2022-03-16 14h10m04s 119.987s(Chemiluminescence).jpg]

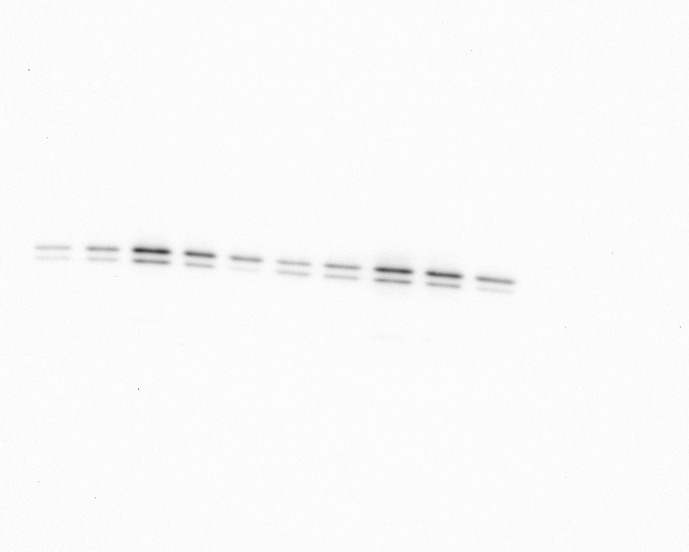

Supplement: Figure 4—figure supplement 1—source data 3. [file elife-83159-fig4-figsupp1-data3.zip › p-ERK Figure 4-figure supplement 1-source data 3/Run 2022-03-16 14h10m04s 119.987s(Chemiluminescence).raw16.tif]

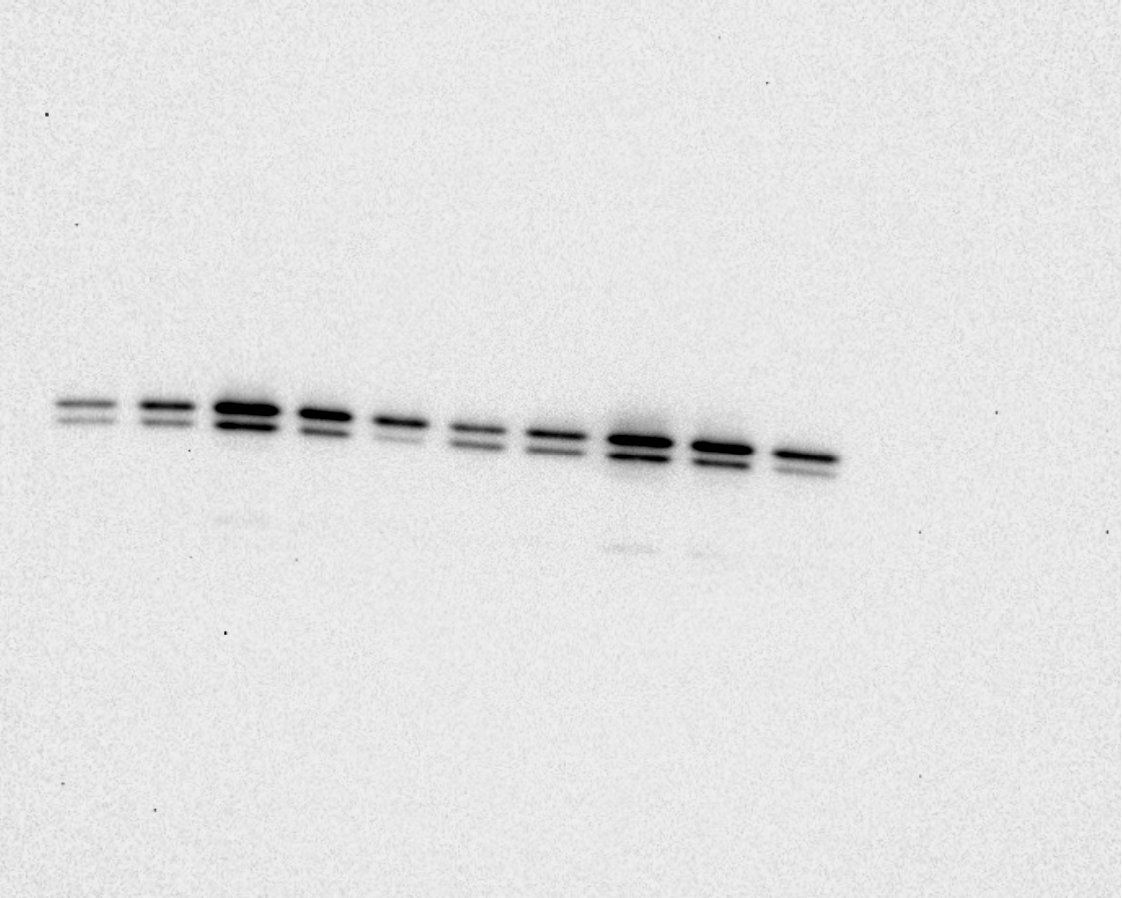

Supplement: Figure 4—figure supplement 1—source data 3. [file elife-83159-fig4-figsupp1-data3.zip › p-ERK Figure 4-figure supplement 1-source data 3/Run 2022-03-16 14h10m04s 119.987s(Chemiluminescence).tif]

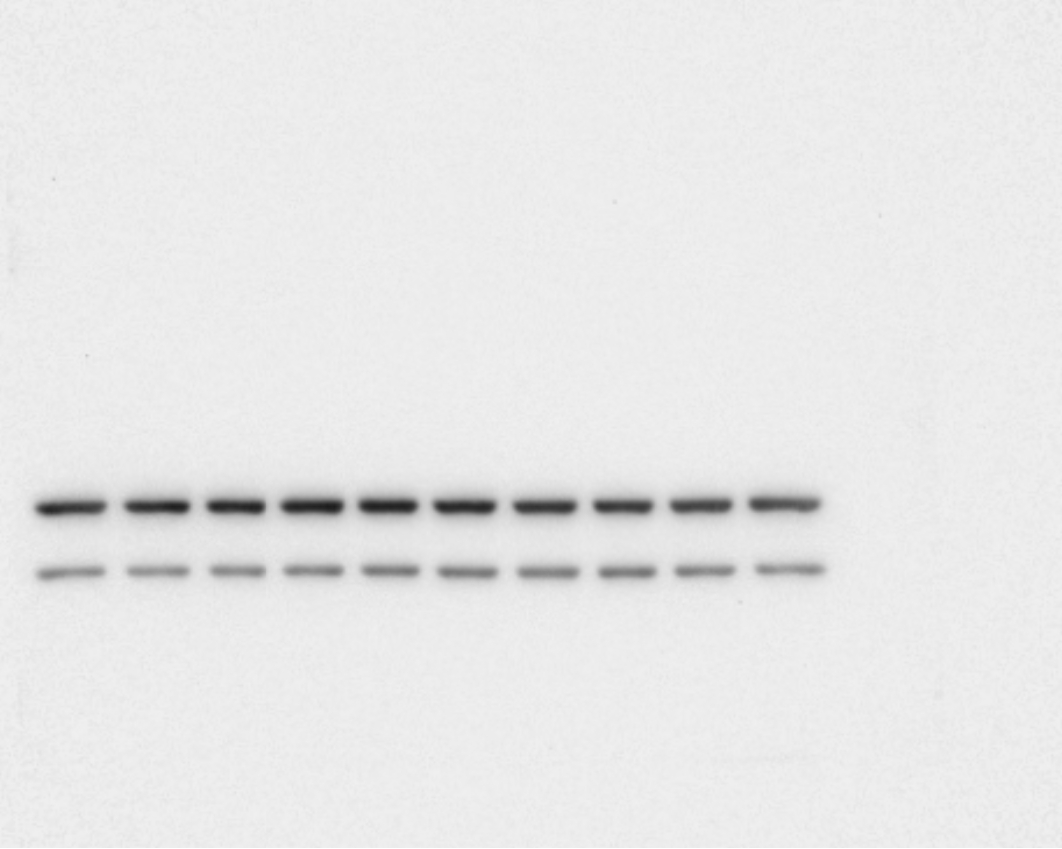

Supplement: Figure 4—figure supplement 1—source data 4. [file elife-83159-fig4-figsupp1-data4.zip › ACTIN Figure 4-figure supplement 1-source data 4/Versteeg 2022-03-28 15h49m37s 25.408s(Chemiluminescence).jpg]

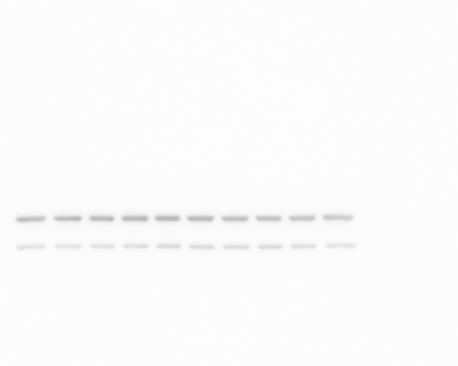

Supplement: Figure 4—figure supplement 1—source data 4. [file elife-83159-fig4-figsupp1-data4.zip › ACTIN Figure 4-figure supplement 1-source data 4/Versteeg 2022-03-28 15h49m37s 25.408s(Chemiluminescence).raw16.tif]

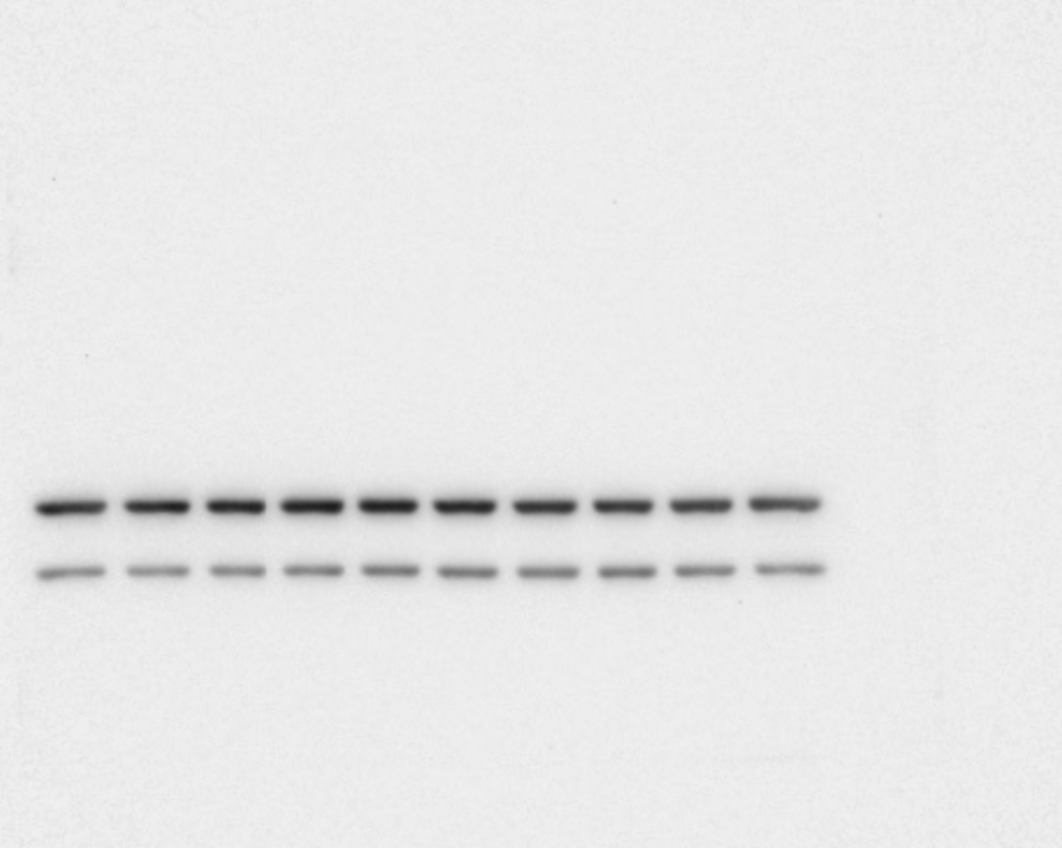

Supplement: Figure 4—figure supplement 1—source data 4. [file elife-83159-fig4-figsupp1-data4.zip › ACTIN Figure 4-figure supplement 1-source data 4/Versteeg 2022-03-28 15h49m37s 25.408s(Chemiluminescence).tif]

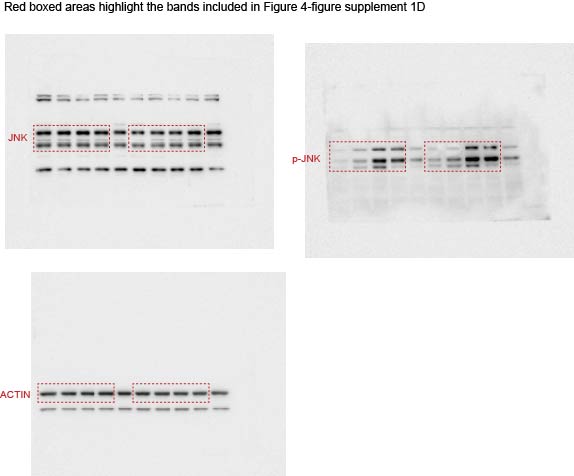

Supplement: Figure 4—figure supplement 1—source data 4. [file elife-83159-fig4-figsupp1-data4.zip › Figure 4-figure supplement 1-source data 4.jpg]

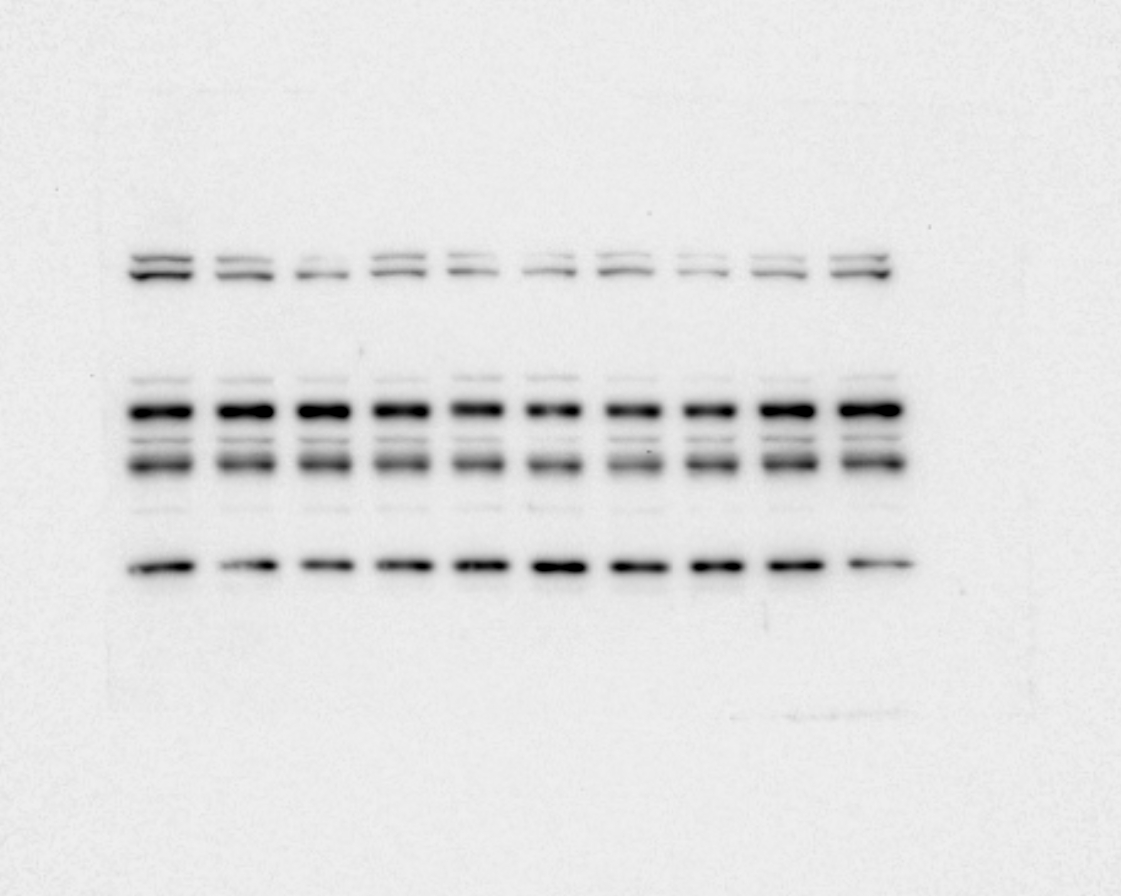

Supplement: Figure 4—figure supplement 1—source data 4. [file elife-83159-fig4-figsupp1-data4.zip › JNK Figure 4-figure supplement 1-source data 4/Versteeg 2022-03-23 15h46m59s 26.335s(Chemiluminescence).jpg]

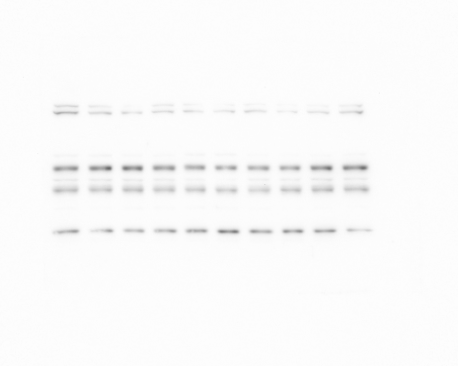

Supplement: Figure 4—figure supplement 1—source data 4. [file elife-83159-fig4-figsupp1-data4.zip › JNK Figure 4-figure supplement 1-source data 4/Versteeg 2022-03-23 15h46m59s 26.335s(Chemiluminescence).raw16.tif]

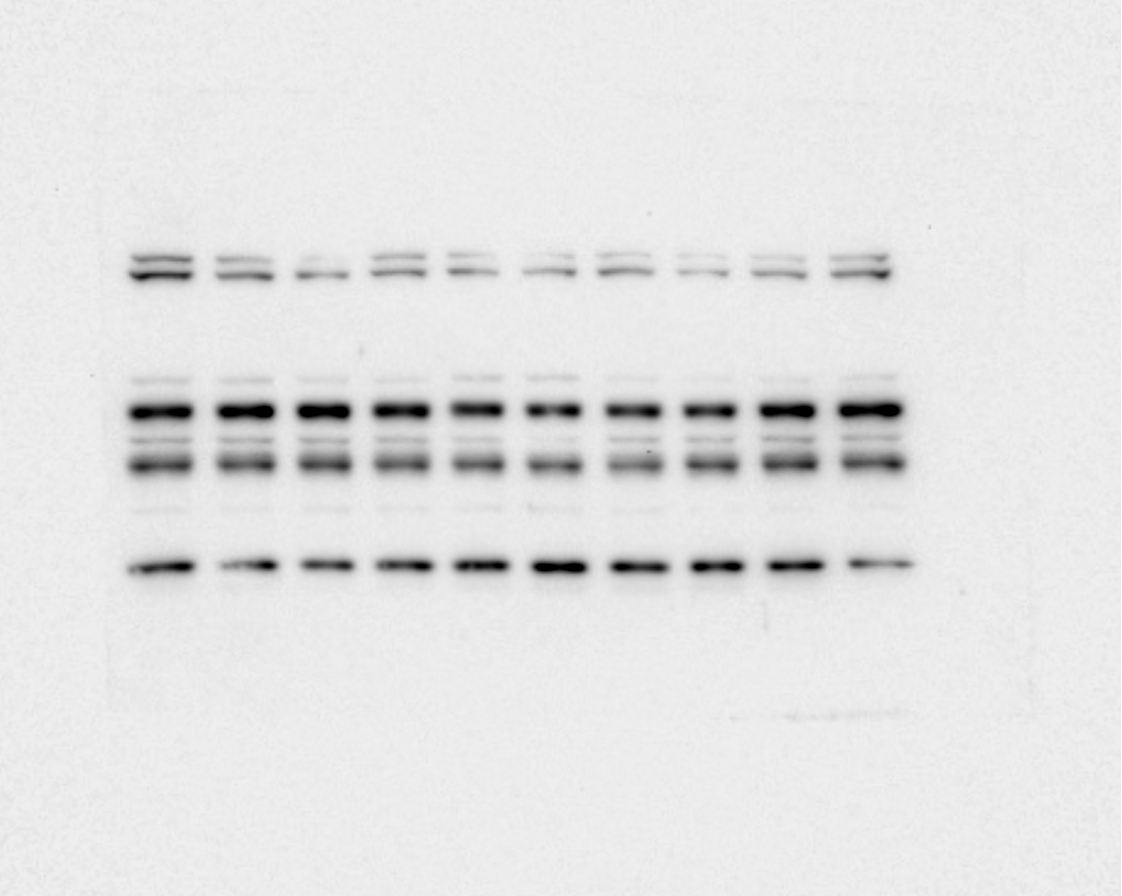

Supplement: Figure 4—figure supplement 1—source data 4. [file elife-83159-fig4-figsupp1-data4.zip › JNK Figure 4-figure supplement 1-source data 4/Versteeg 2022-03-23 15h46m59s 26.335s(Chemiluminescence).tif]

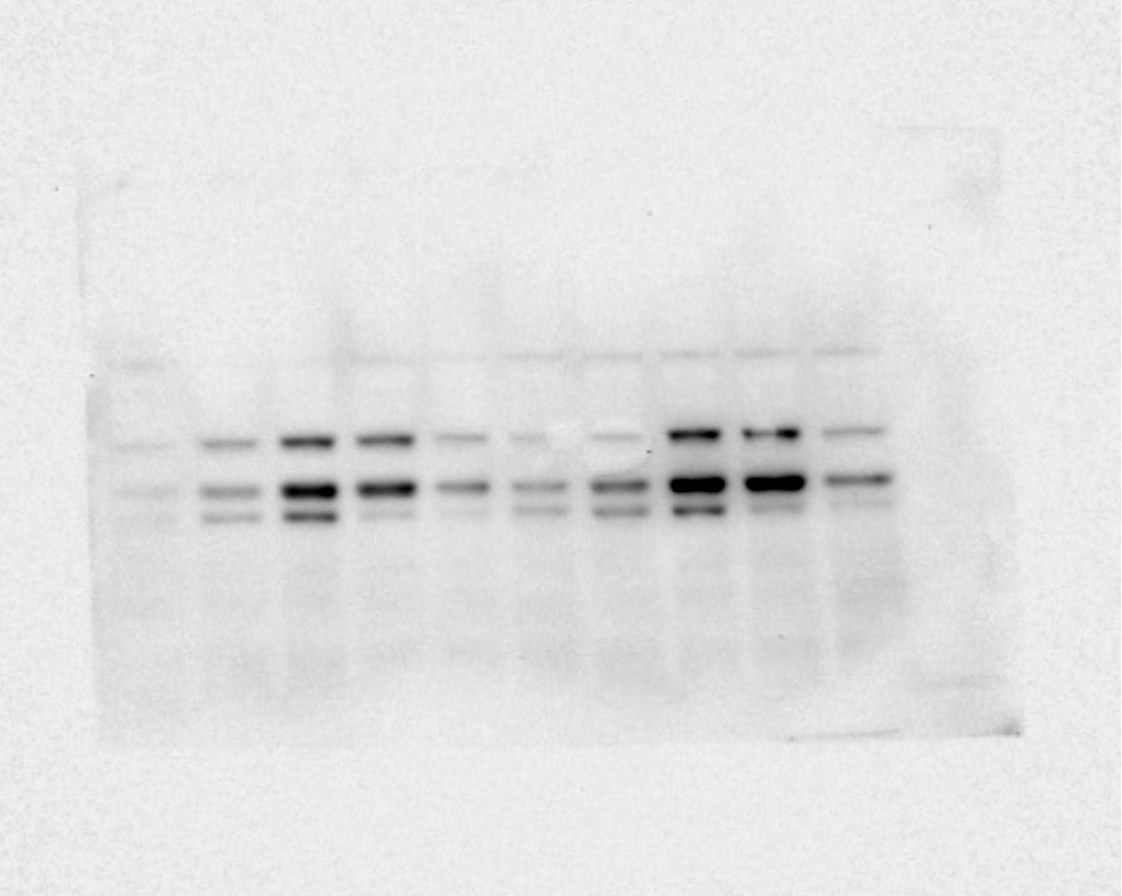

Supplement: Figure 4—figure supplement 1—source data 4. [file elife-83159-fig4-figsupp1-data4.zip › p-JNK Figure 4-figure supplement 1-source data 4/Versteeg 2022-03-17 20h19m40s 41.536s(Chemiluminescence).jpg]

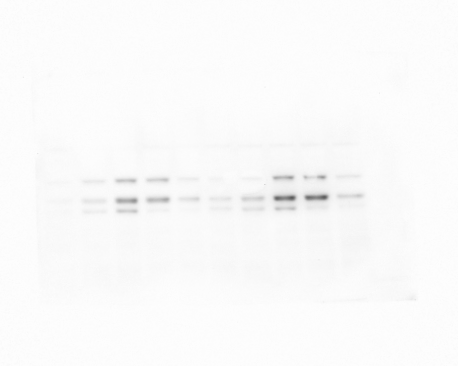

Supplement: Figure 4—figure supplement 1—source data 4. [file elife-83159-fig4-figsupp1-data4.zip › p-JNK Figure 4-figure supplement 1-source data 4/Versteeg 2022-03-17 20h19m40s 41.536s(Chemiluminescence).raw16.tif]

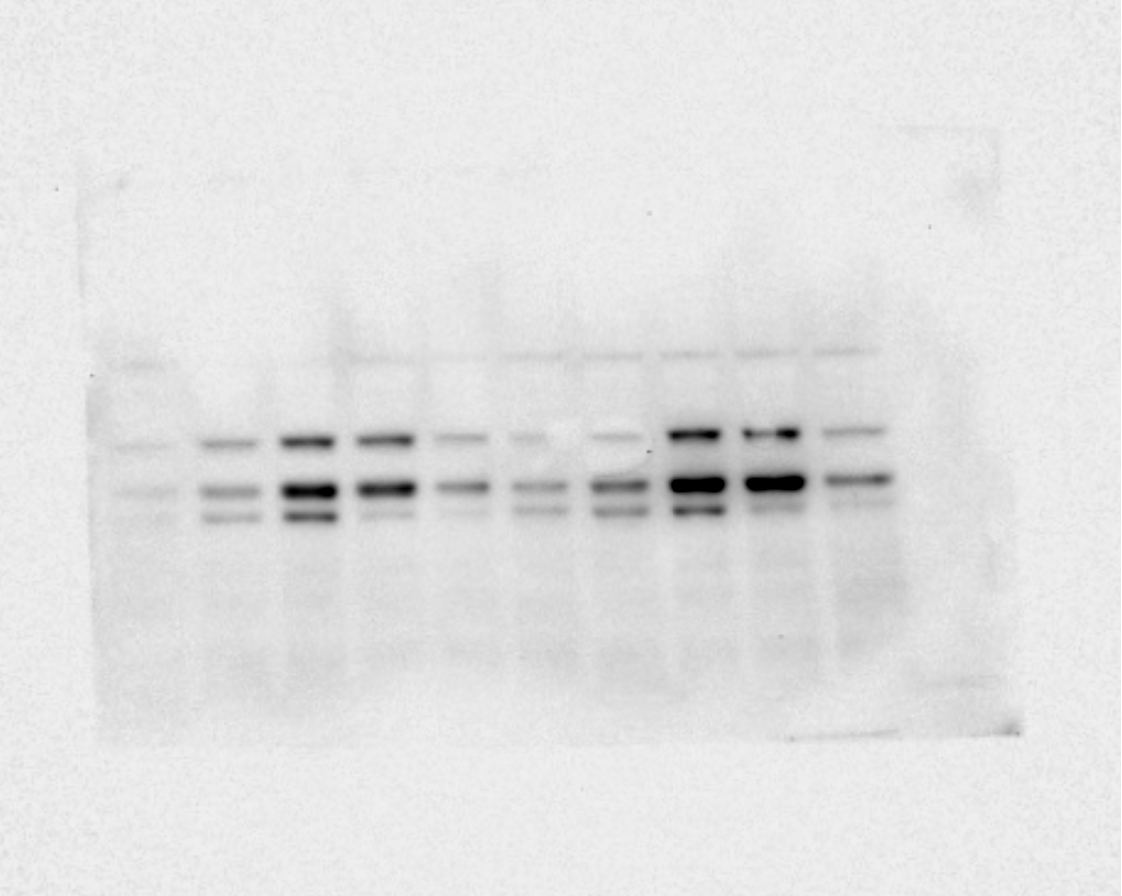

Supplement: Figure 4—figure supplement 1—source data 4. [file elife-83159-fig4-figsupp1-data4.zip › p-JNK Figure 4-figure supplement 1-source data 4/Versteeg 2022-03-17 20h19m40s 41.536s(Chemiluminescence).tif]

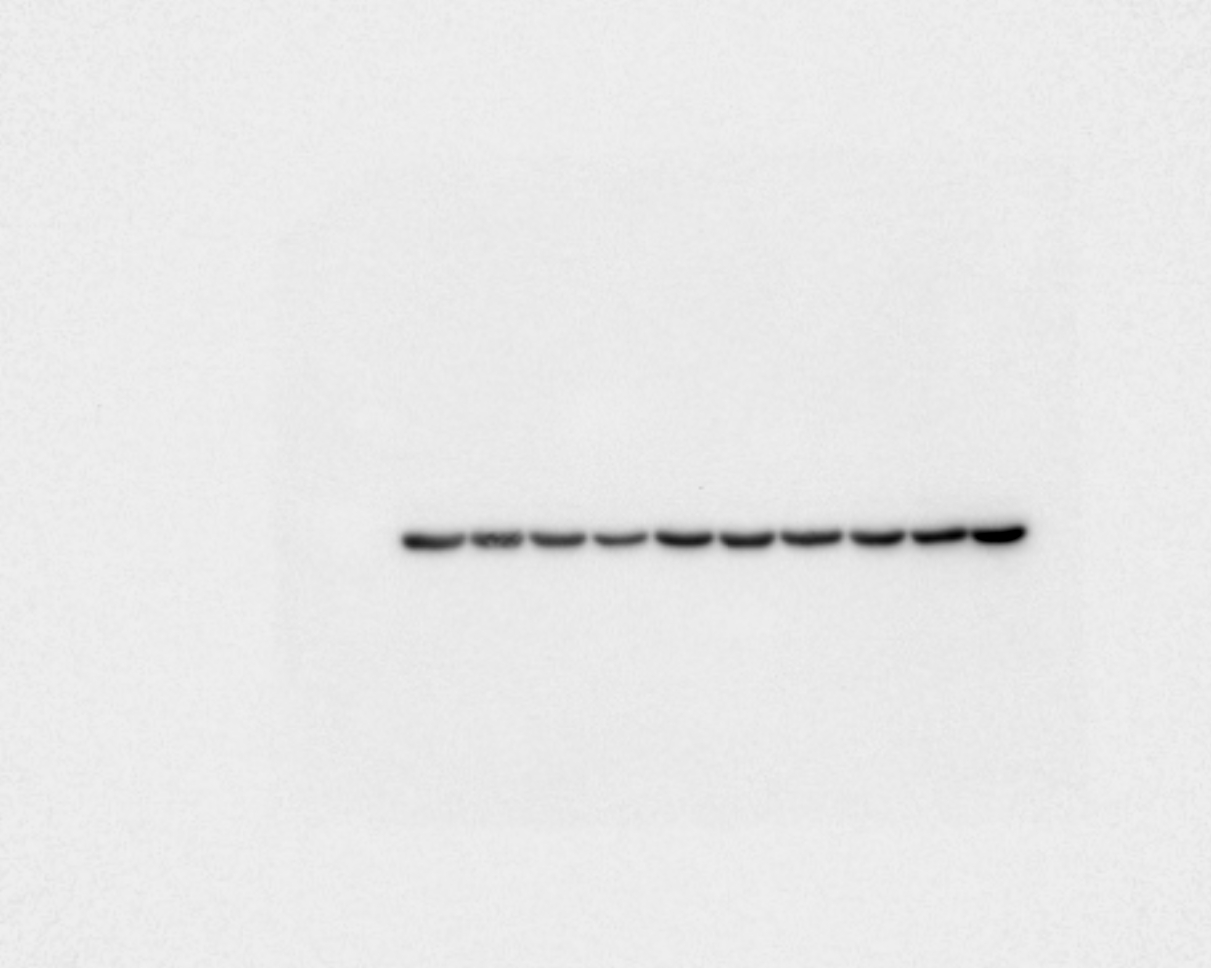

Supplement: Figure 4—figure supplement 1—source data 5. [file elife-83159-fig4-figsupp1-data5.zip › ACTIN_1 Figure 4-figure supplement 1-source data 5/Versteeg 2021-12-21 15h54m42s 9.136s(Chemiluminescence).jpg]

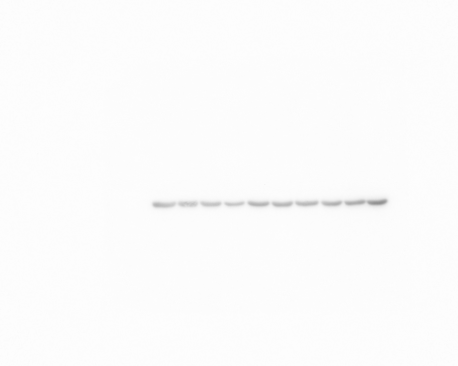

Supplement: Figure 4—figure supplement 1—source data 5. [file elife-83159-fig4-figsupp1-data5.zip › ACTIN_1 Figure 4-figure supplement 1-source data 5/Versteeg 2021-12-21 15h54m42s 9.136s(Chemiluminescence).raw16.tif]

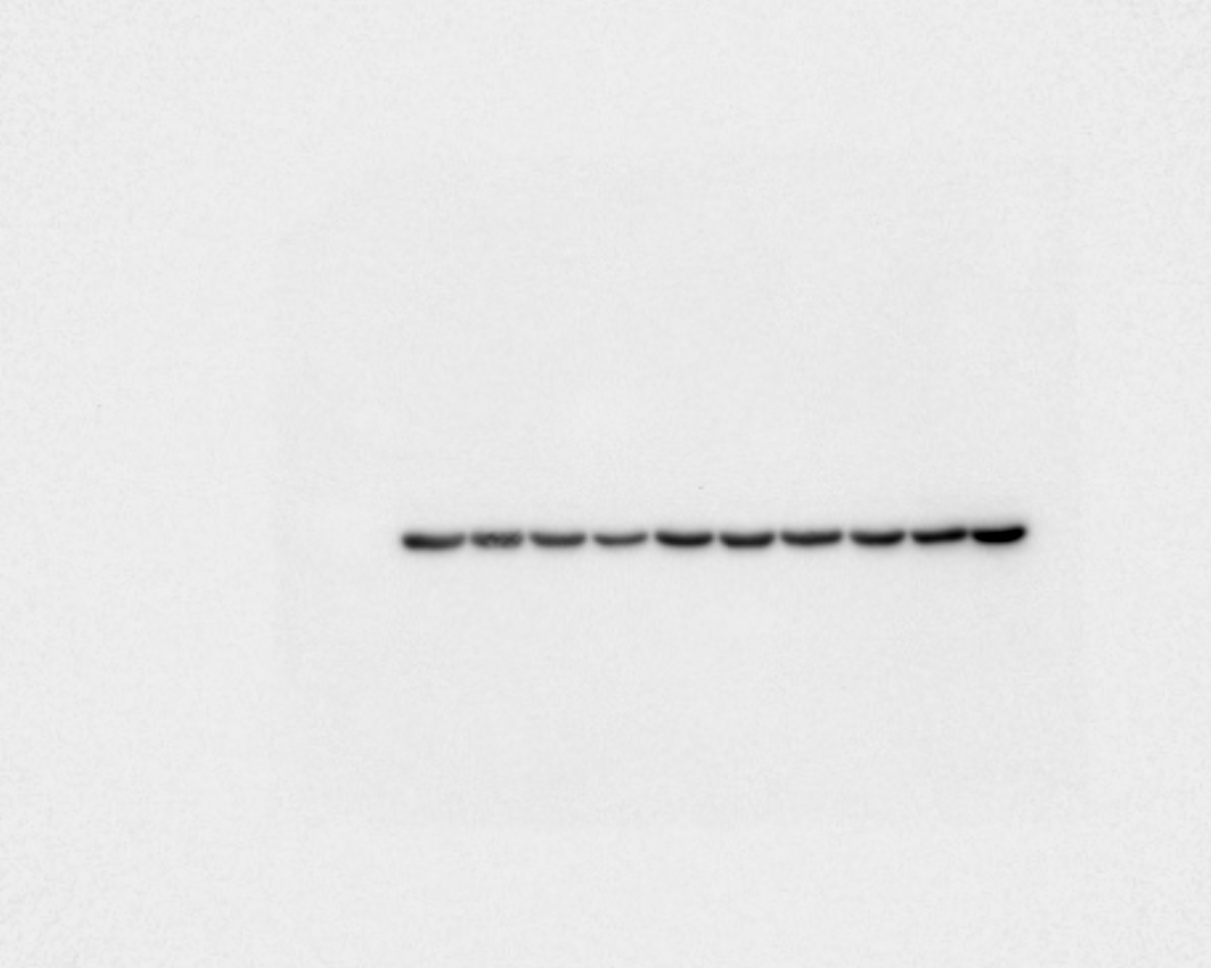

Supplement: Figure 4—figure supplement 1—source data 5. [file elife-83159-fig4-figsupp1-data5.zip › ACTIN_1 Figure 4-figure supplement 1-source data 5/Versteeg 2021-12-21 15h54m42s 9.136s(Chemiluminescence).tif]

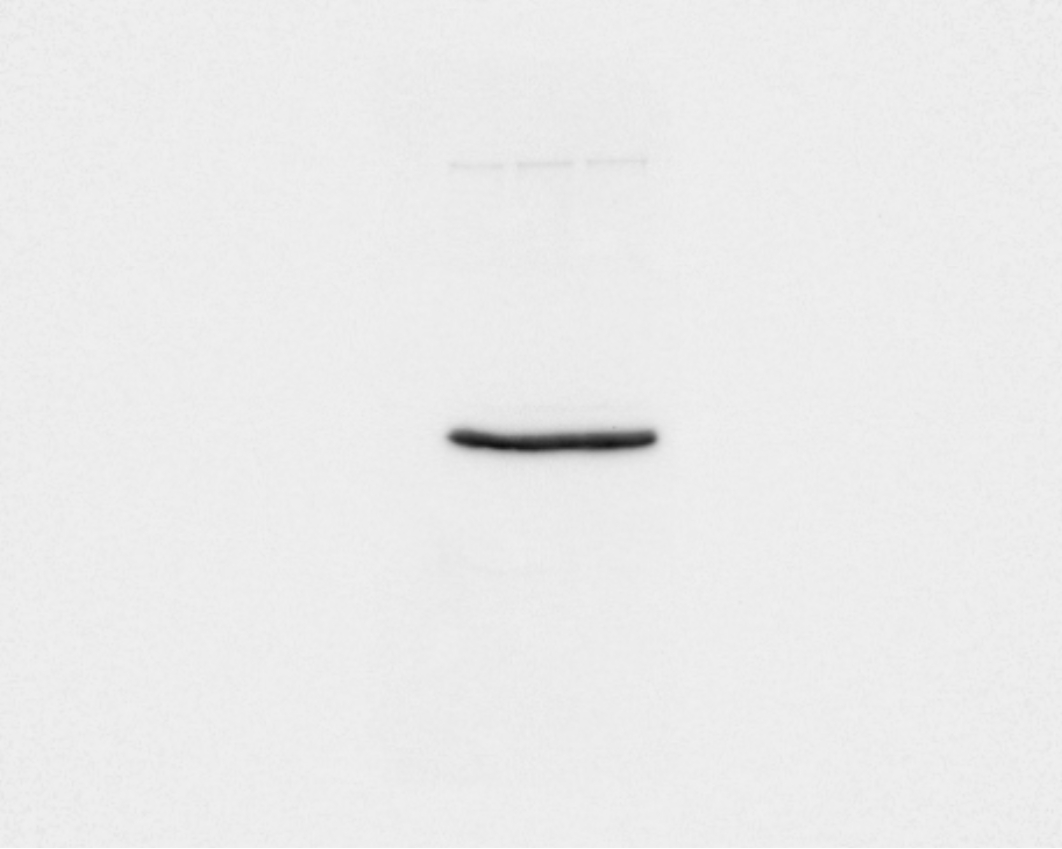

Supplement: Figure 4—figure supplement 1—source data 5. [file elife-83159-fig4-figsupp1-data5.zip › ACTIN_2 Figure 4-figure supplement 1-source data 5/Versteeg 2022-01-20 10h57m40s 7.750s(Chemiluminescence).jpg]

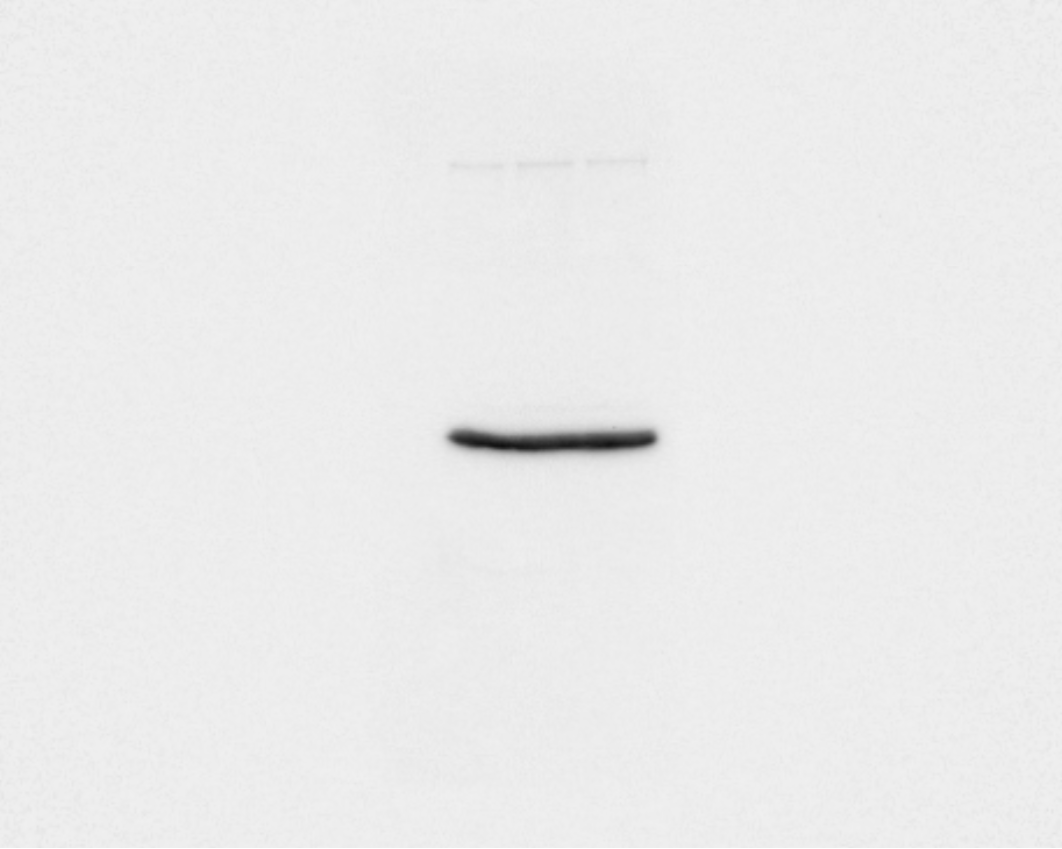

Supplement: Figure 4—figure supplement 1—source data 5. [file elife-83159-fig4-figsupp1-data5.zip › ACTIN_2 Figure 4-figure supplement 1-source data 5/Versteeg 2022-01-20 10h57m40s 7.750s(Chemiluminescence).tif]

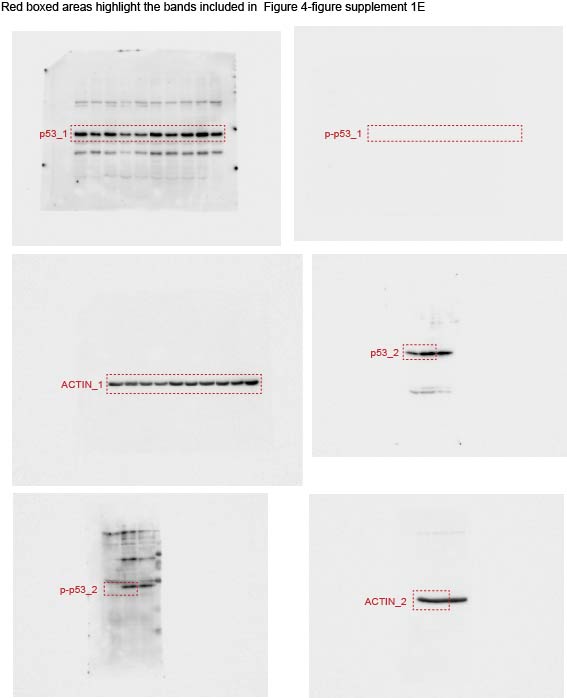

Supplement: Figure 4—figure supplement 1—source data 5. [file elife-83159-fig4-figsupp1-data5.zip › Figure 4-figure supplement 1-source data 5.jpg]

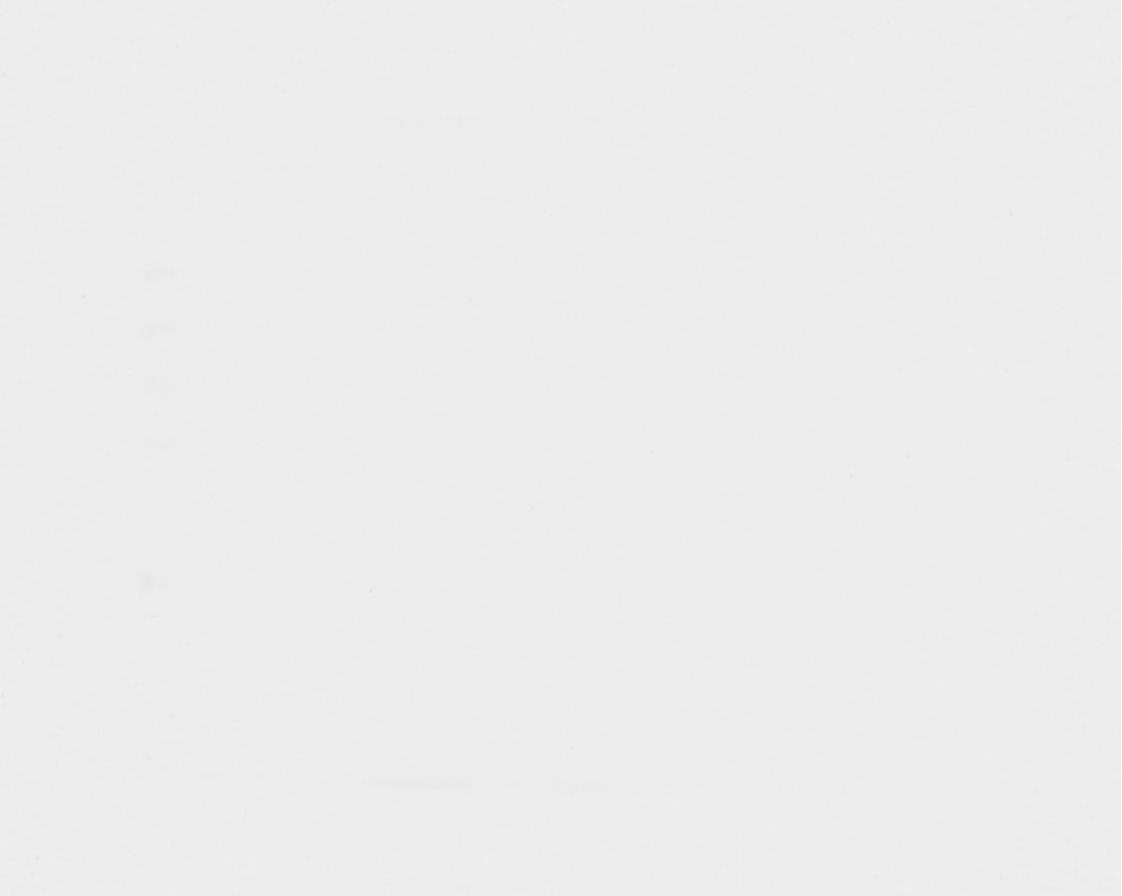

Supplement: Figure 4—figure supplement 1—source data 5. [file elife-83159-fig4-figsupp1-data5.zip › p-p53_1 Figure 4-figure supplement 1-source data 5/Versteeg 2021-12-22 14h17m39s 60.000s(Chemiluminescence).jpg]

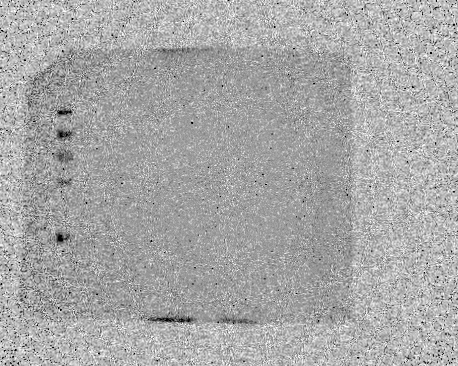

Supplement: Figure 4—figure supplement 1—source data 5. [file elife-83159-fig4-figsupp1-data5.zip › p-p53_1 Figure 4-figure supplement 1-source data 5/Versteeg 2021-12-22 14h17m39s 60.000s(Chemiluminescence).raw16.tif]

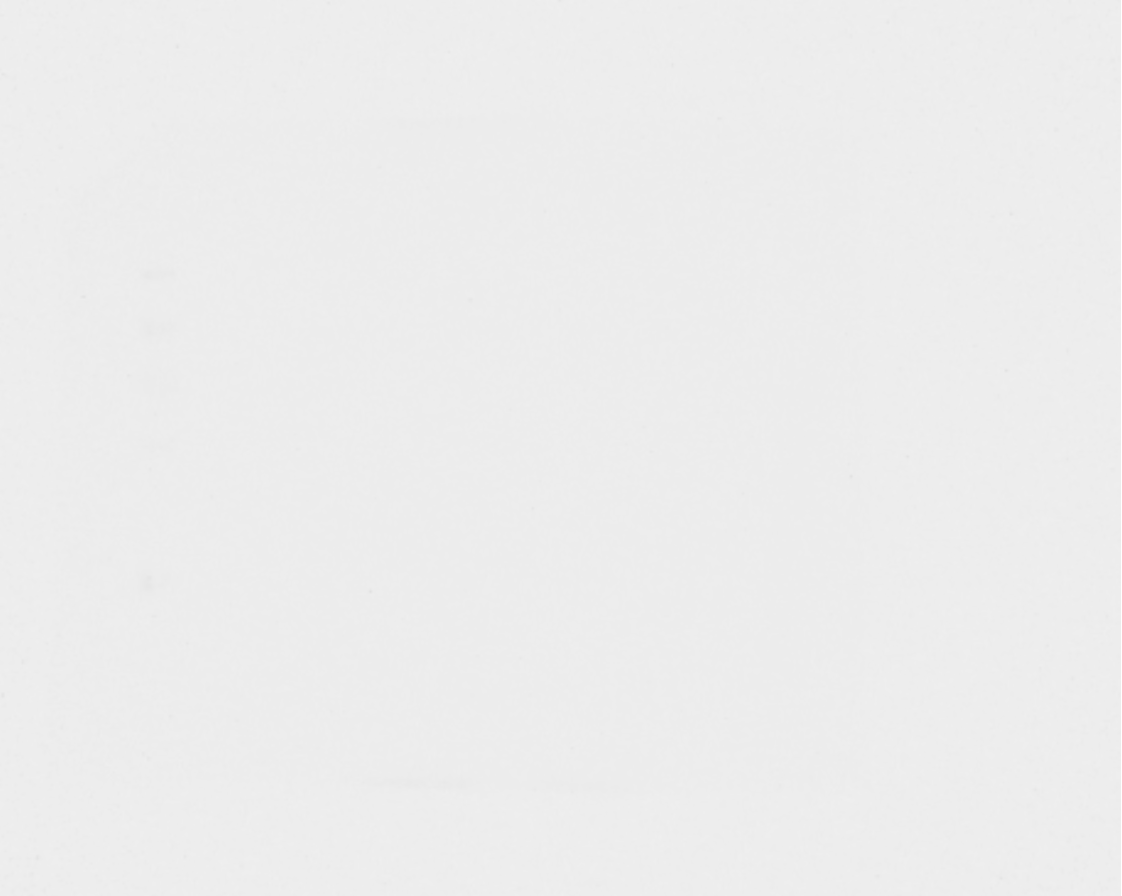

Supplement: Figure 4—figure supplement 1—source data 5. [file elife-83159-fig4-figsupp1-data5.zip › p-p53_1 Figure 4-figure supplement 1-source data 5/Versteeg 2021-12-22 14h17m39s 60.000s(Chemiluminescence).tif]

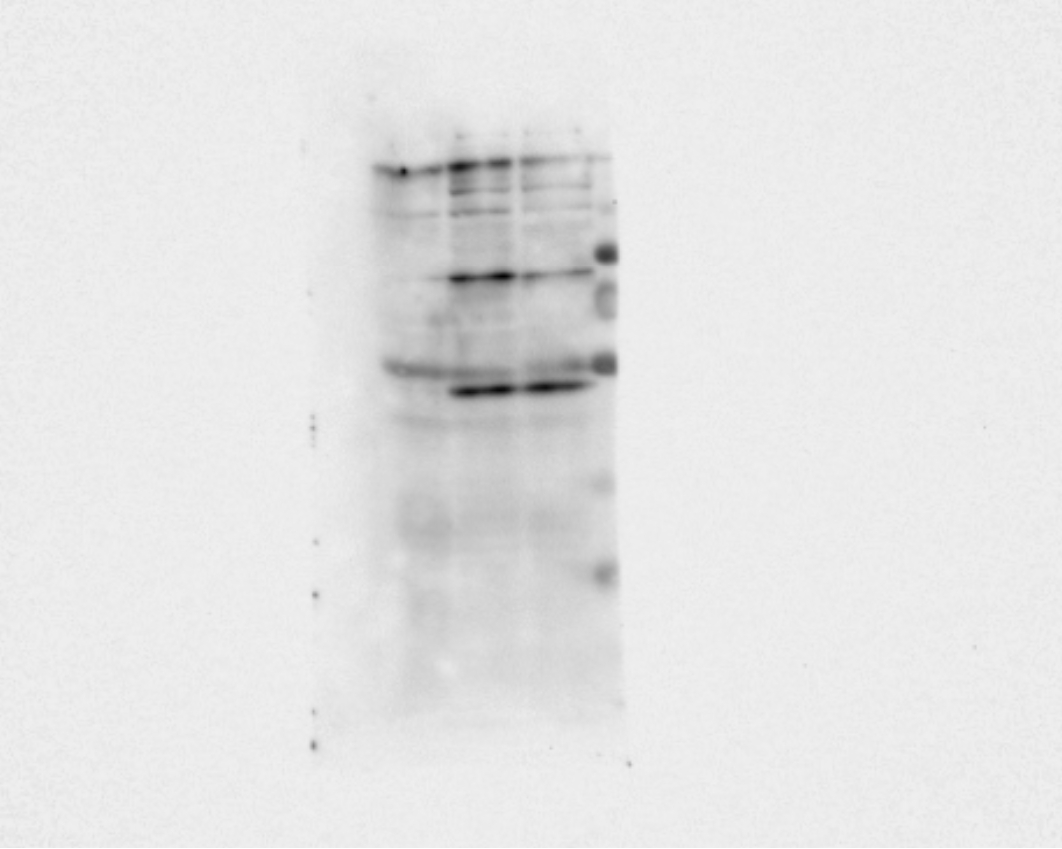

Supplement: Figure 4—figure supplement 1—source data 5. [file elife-83159-fig4-figsupp1-data5.zip › p-p53_2 Figure 4-figure supplement 1-source data 5/Versteeg 2022-01-14 12h40m26s 57.856s(Chemiluminescence).jpg]
