# Supplementary figures and images for: HUWE1 controls tristetraprolin proteasomal degradation by regulating its phosphorylation (part 4 of 4)
Source: eLife. 2023 Mar 24;12:e83159. doi: 10.7554/eLife.83159 (PMC10038661; doi:10.7554/eLife.83159)

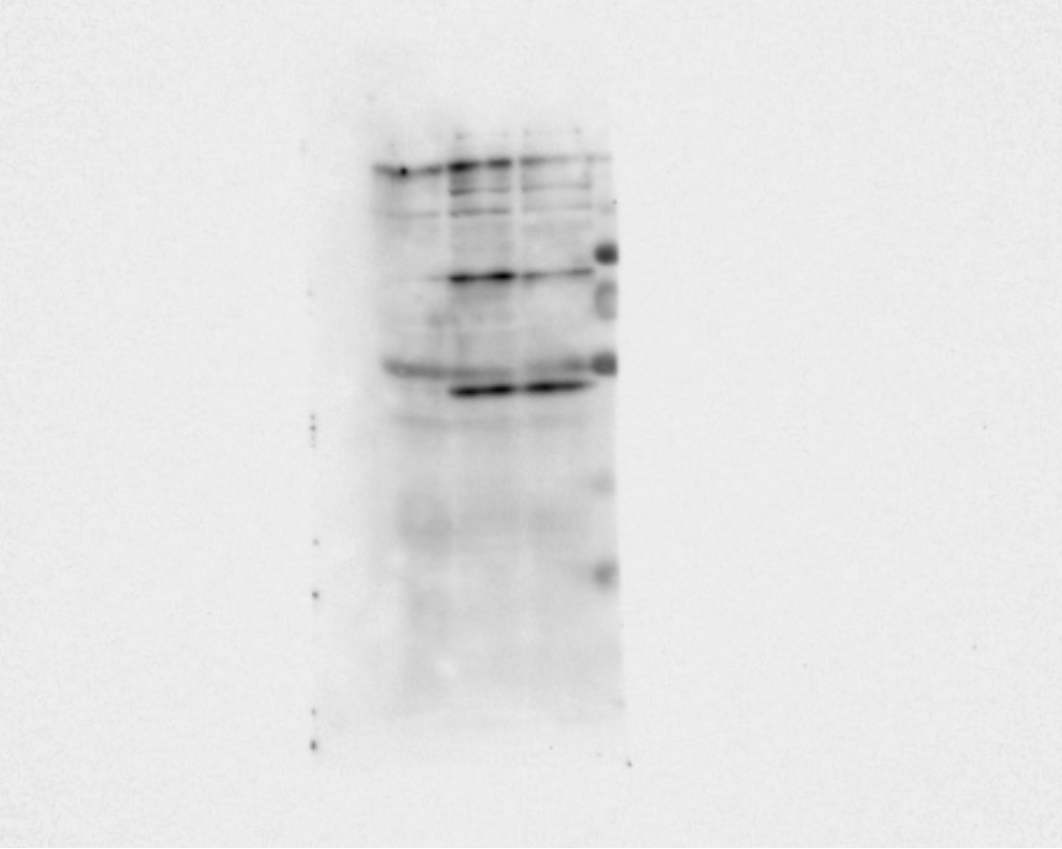

Supplement: Figure 4—figure supplement 1—source data 5. [file elife-83159-fig4-figsupp1-data5.zip › p-p53_2 Figure 4-figure supplement 1-source data 5/Versteeg 2022-01-14 12h40m26s 57.856s(Chemiluminescence).tif]

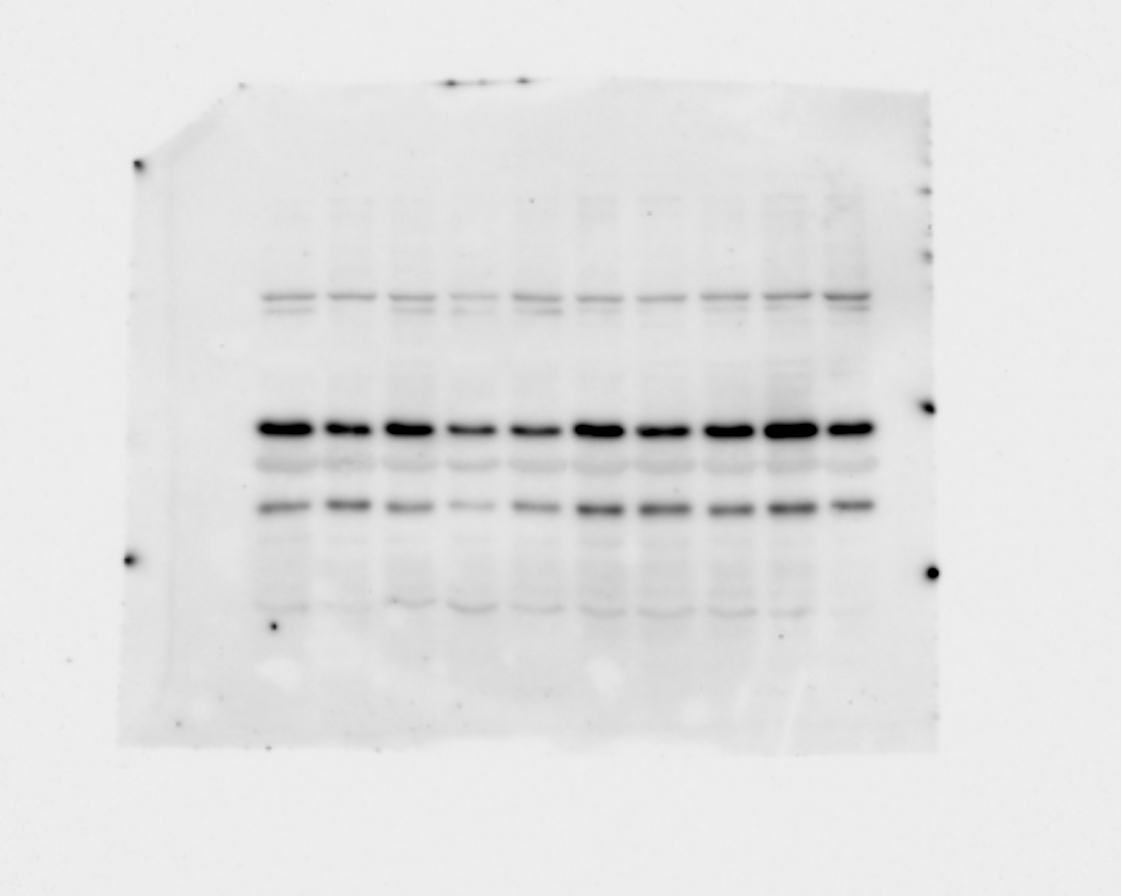

Supplement: Figure 4—figure supplement 1—source data 5. [file elife-83159-fig4-figsupp1-data5.zip › p53_1 Figure 4-figure supplement 1-source data 5/Versteeg 2021-12-21 12h10m56s 60.000s(Chemiluminescence).jpg]

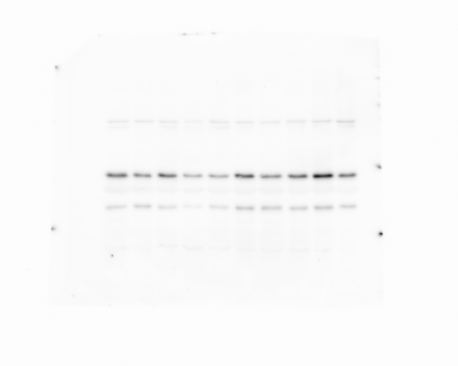

Supplement: Figure 4—figure supplement 1—source data 5. [file elife-83159-fig4-figsupp1-data5.zip › p53_1 Figure 4-figure supplement 1-source data 5/Versteeg 2021-12-21 12h10m56s 60.000s(Chemiluminescence).raw16.tif]

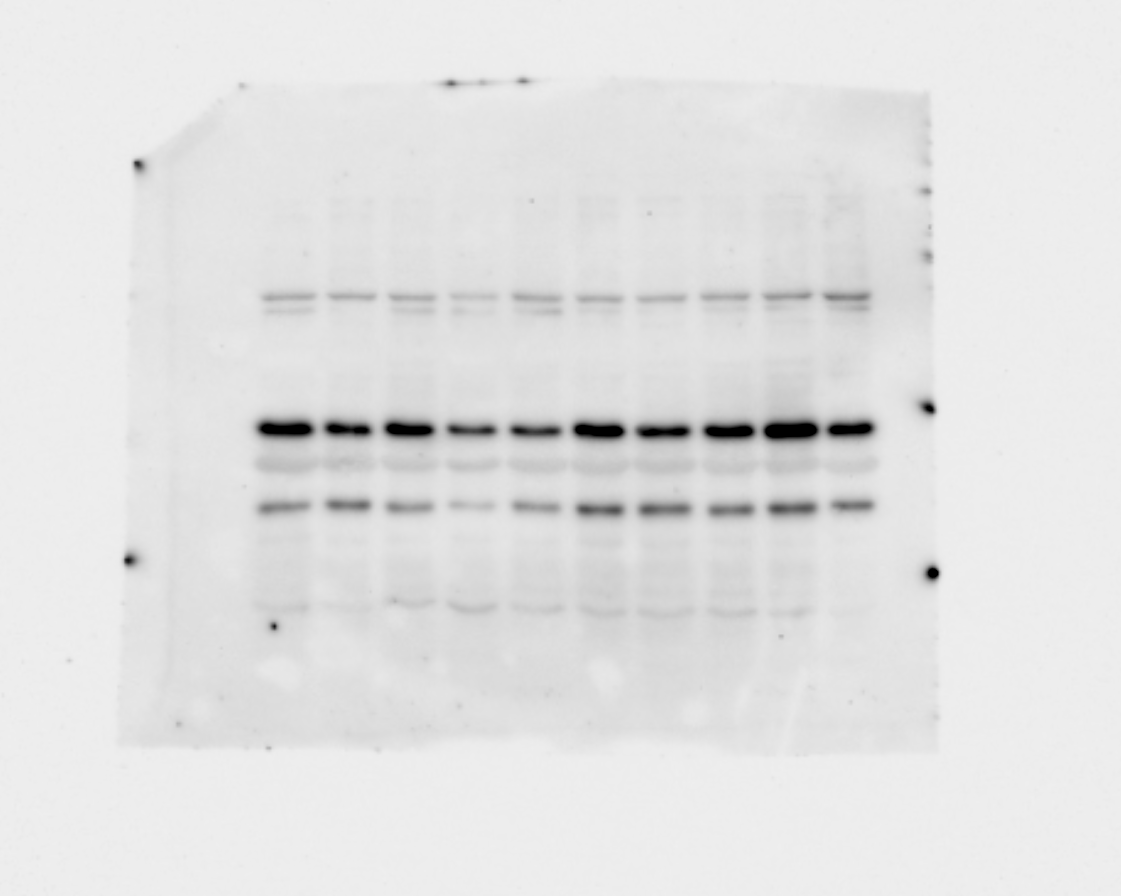

Supplement: Figure 4—figure supplement 1—source data 5. [file elife-83159-fig4-figsupp1-data5.zip › p53_1 Figure 4-figure supplement 1-source data 5/Versteeg 2021-12-21 12h10m56s 60.000s(Chemiluminescence).tif]

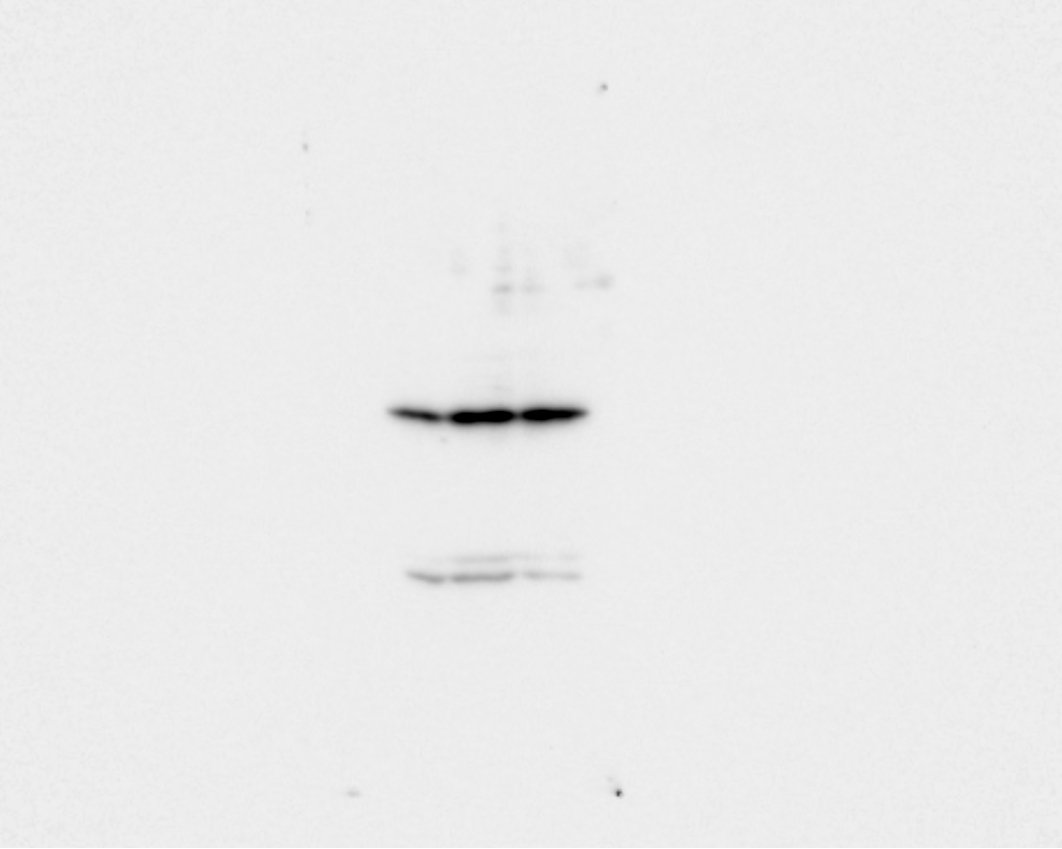

Supplement: Figure 4—figure supplement 1—source data 5. [file elife-83159-fig4-figsupp1-data5.zip › p53_2 Figure 4-figure supplement 1-source data 5/Versteeg 2022-01-19 12h40m25s 18.000s(Chemiluminescence).jpg]

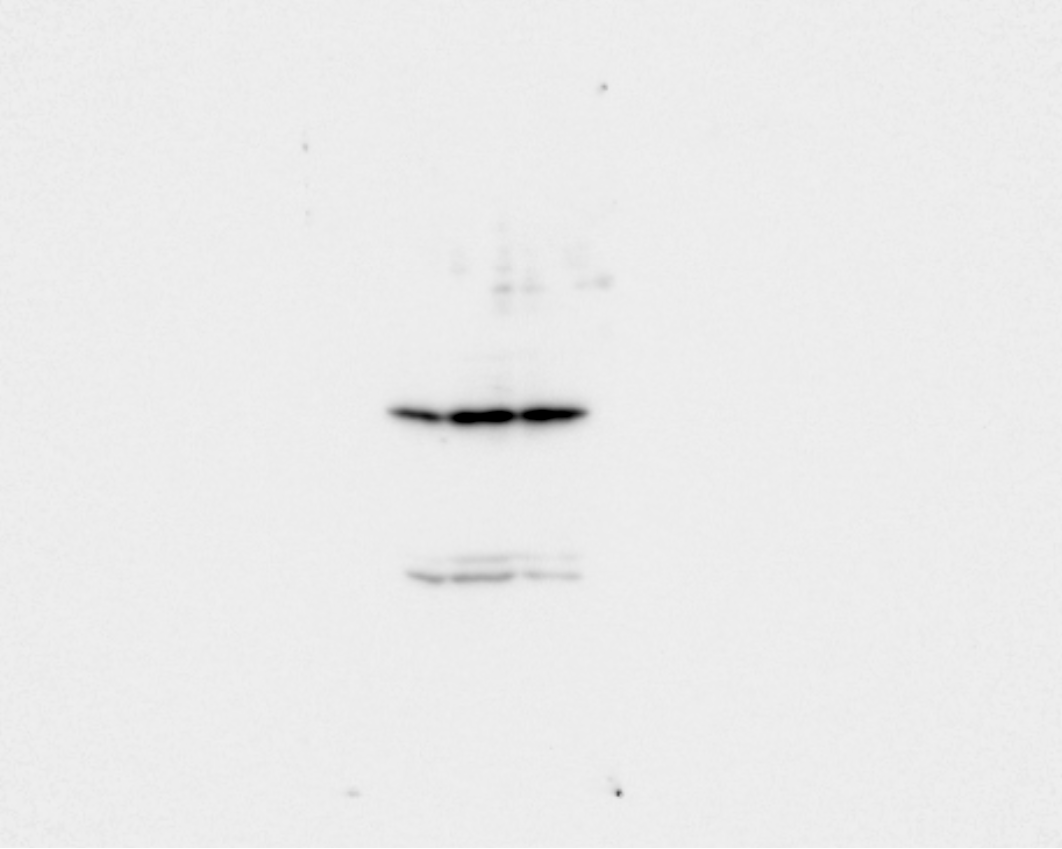

Supplement: Figure 4—figure supplement 1—source data 5. [file elife-83159-fig4-figsupp1-data5.zip › p53_2 Figure 4-figure supplement 1-source data 5/Versteeg 2022-01-19 12h40m25s 18.000s(Chemiluminescence).tif]

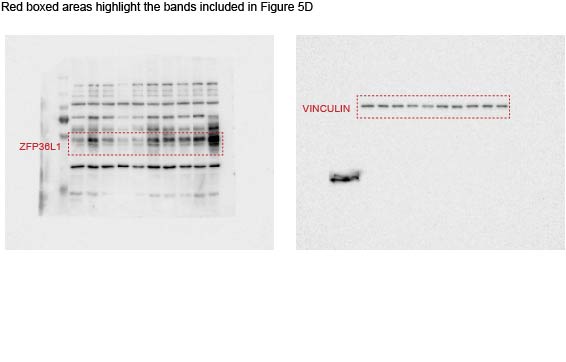

Supplement: Figure 5—source data 1. [file elife-83159-fig5-data1.zip › Figure 5-source data 1/Figure 5-source data 1.jpg]

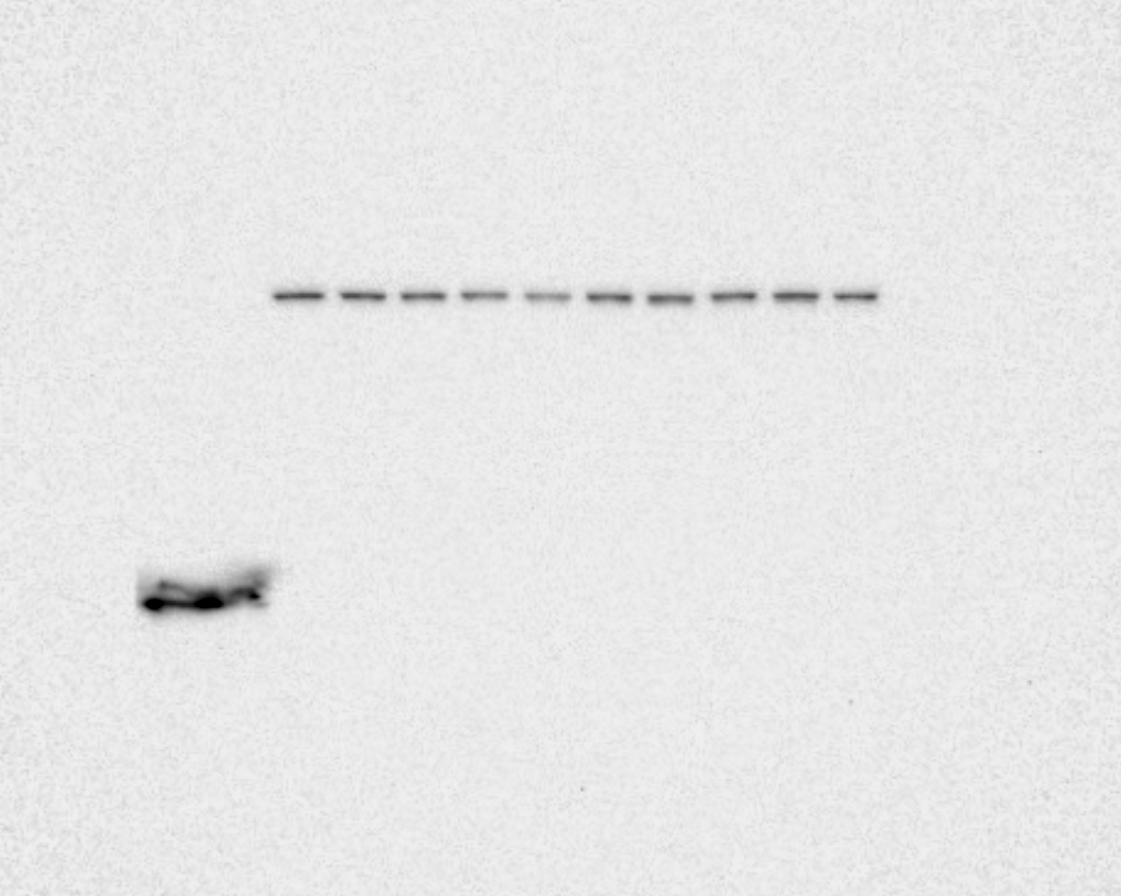

Supplement: Figure 5—source data 1. [file elife-83159-fig5-data1.zip › Figure 5-source data 1/VINCULIN Figure 5-source data 1/Versteeg 2022-02-09 17h09m29s 184.125s(Chemiluminescence).jpg]

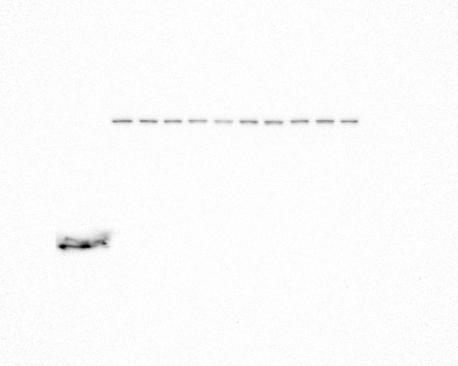

Supplement: Figure 5—source data 1. [file elife-83159-fig5-data1.zip › Figure 5-source data 1/VINCULIN Figure 5-source data 1/Versteeg 2022-02-09 17h09m29s 184.125s(Chemiluminescence).raw16.tif]

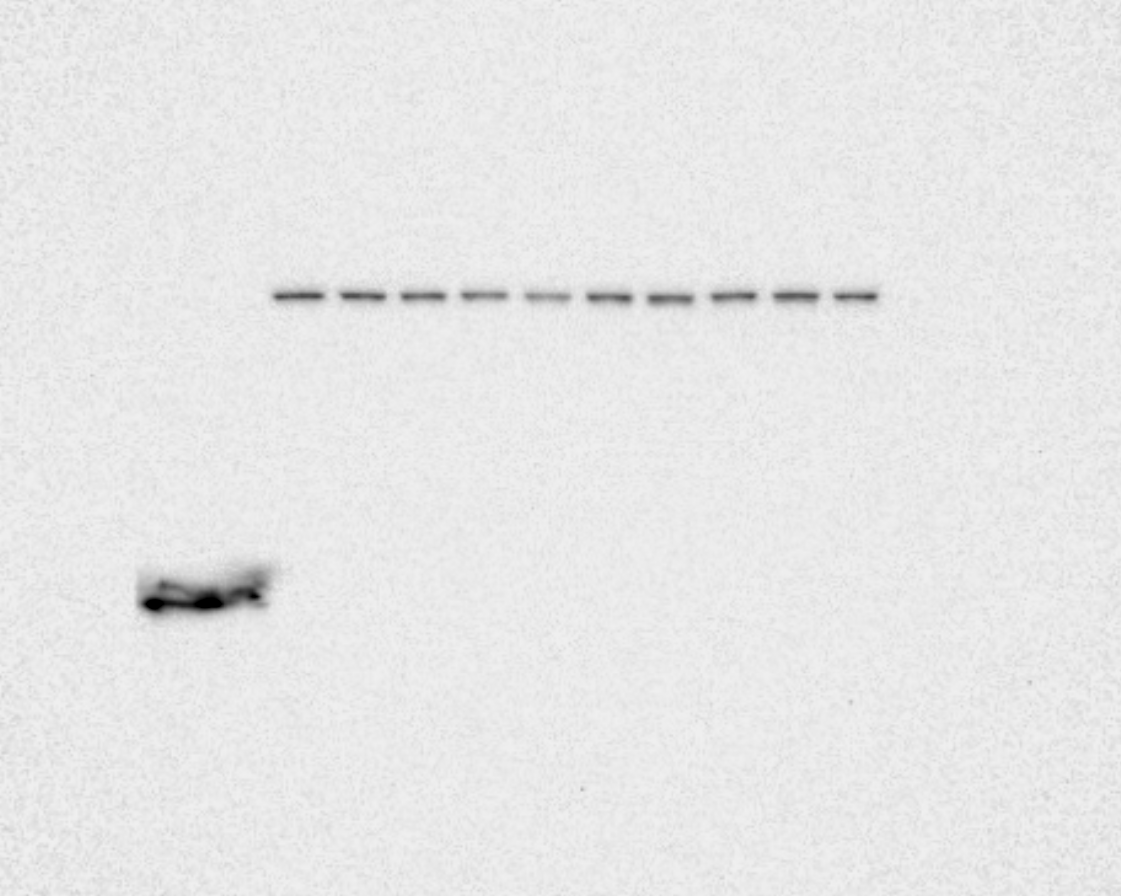

Supplement: Figure 5—source data 1. [file elife-83159-fig5-data1.zip › Figure 5-source data 1/VINCULIN Figure 5-source data 1/Versteeg 2022-02-09 17h09m29s 184.125s(Chemiluminescence).tif]

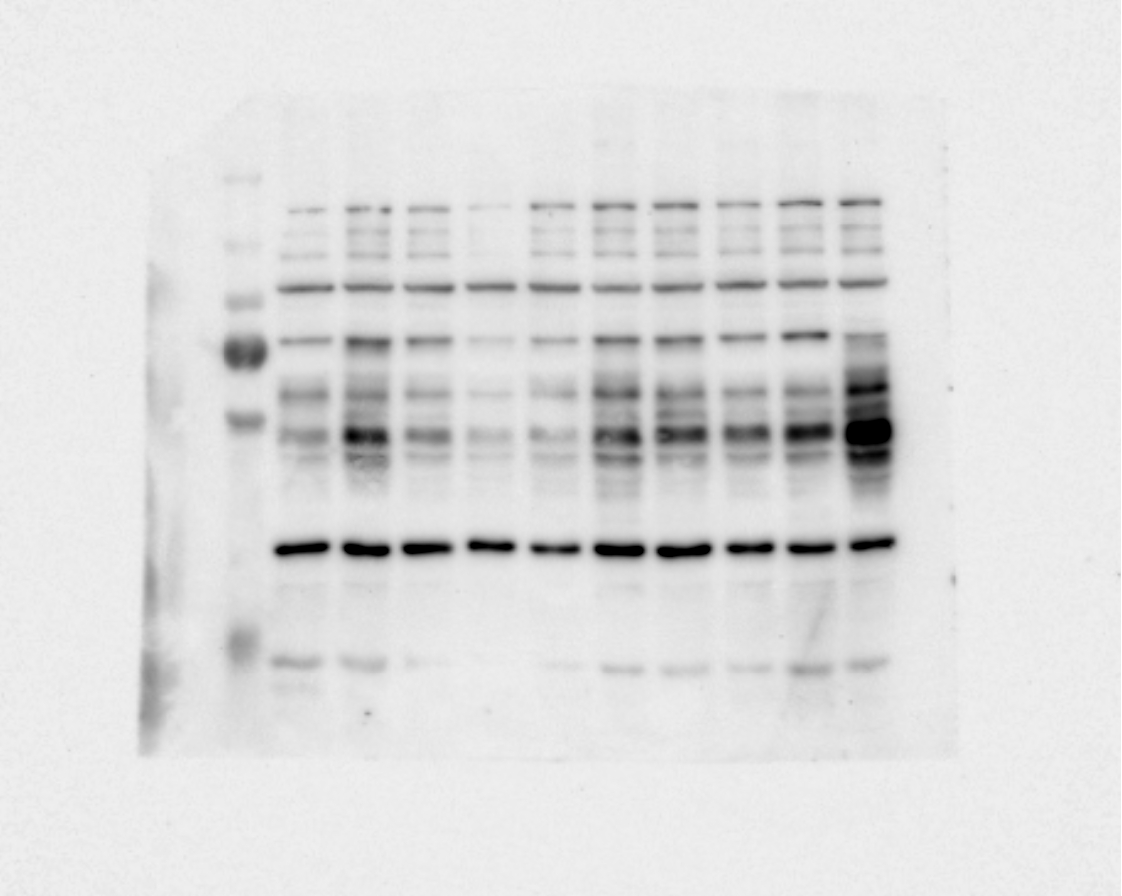

Supplement: Figure 5—source data 1. [file elife-83159-fig5-data1.zip › Figure 5-source data 1/ZFP36L1 Figure 5-source data 1/Versteeg 2021-12-20 14h29m06s 84.825s(Chemiluminescence).jpg]

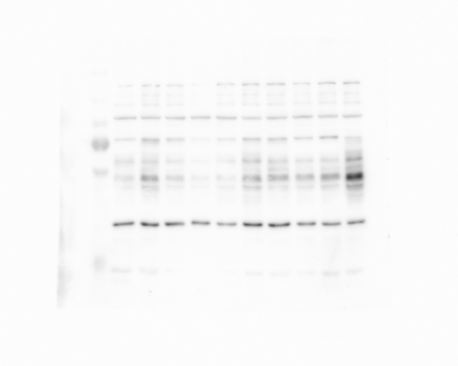

Supplement: Figure 5—source data 1. [file elife-83159-fig5-data1.zip › Figure 5-source data 1/ZFP36L1 Figure 5-source data 1/Versteeg 2021-12-20 14h29m06s 84.825s(Chemiluminescence).raw16.tif]

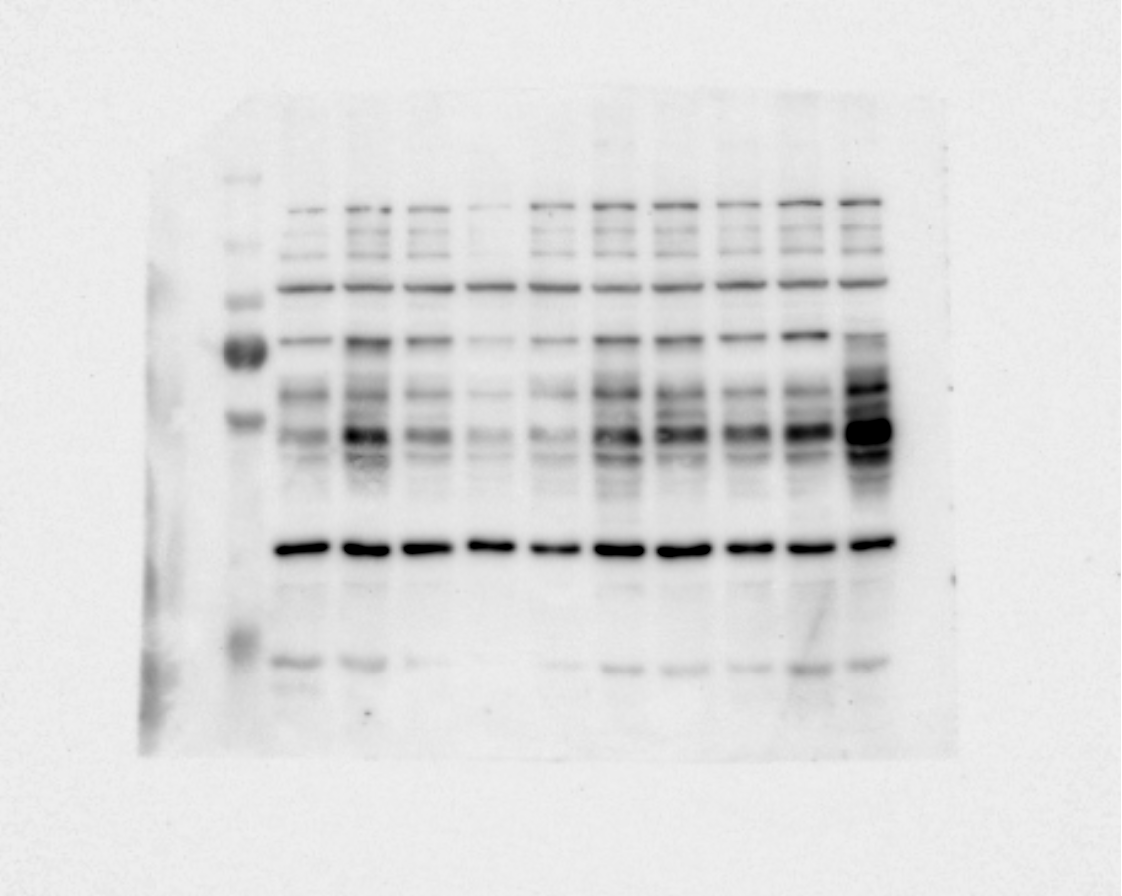

Supplement: Figure 5—source data 1. [file elife-83159-fig5-data1.zip › Figure 5-source data 1/ZFP36L1 Figure 5-source data 1/Versteeg 2021-12-20 14h29m06s 84.825s(Chemiluminescence).tif]

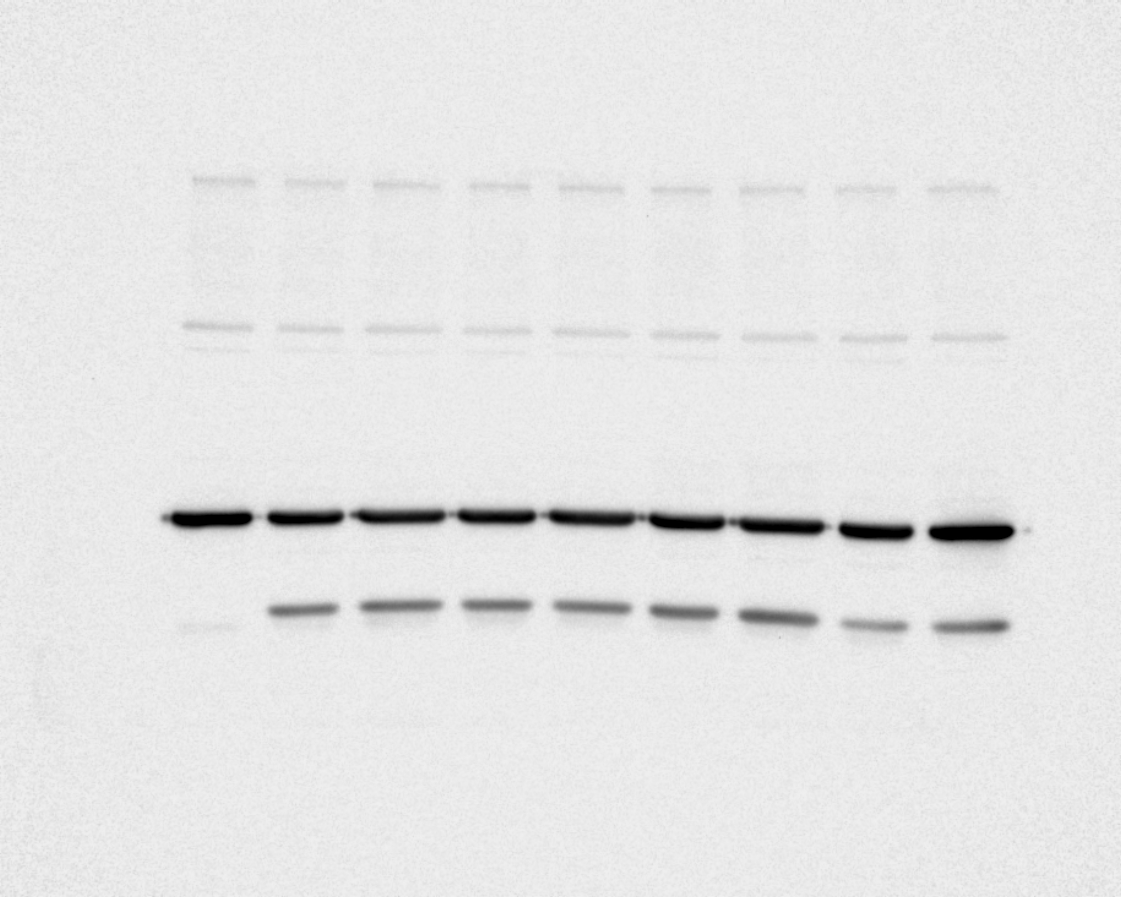

Supplement: Figure 6—source data 1. [file elife-83159-fig6-data1.zip › Figure 6-source data 1/ACTIN Figure 6-source data 1/Versteeg 2022-08-16 16h59m43s 46.495s(Chemiluminescence).jpg]

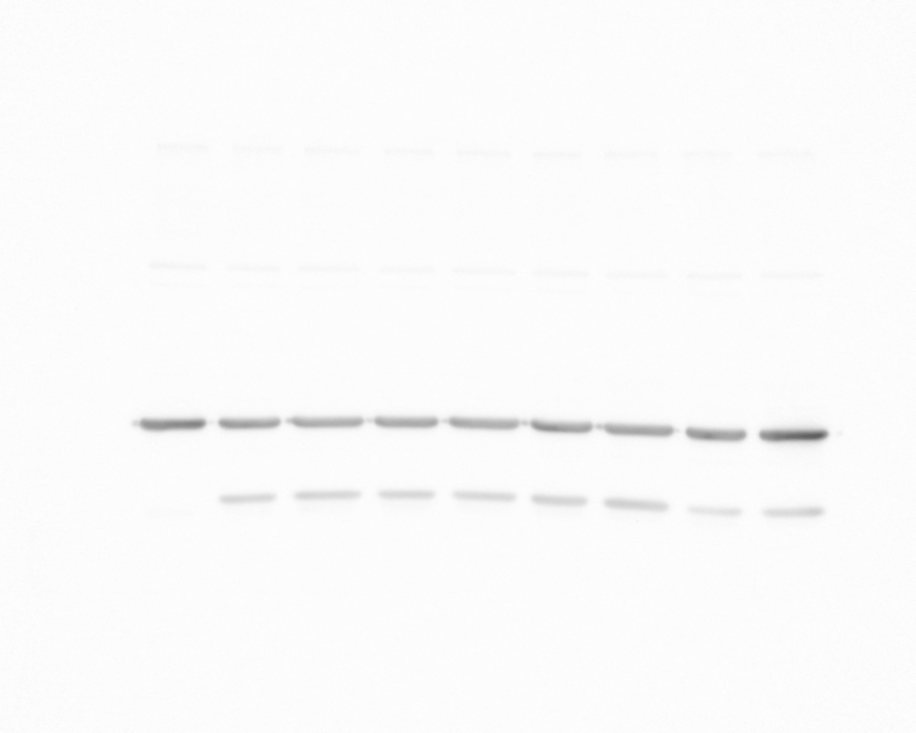

Supplement: Figure 6—source data 1. [file elife-83159-fig6-data1.zip › Figure 6-source data 1/ACTIN Figure 6-source data 1/Versteeg 2022-08-16 16h59m43s 46.495s(Chemiluminescence).raw16.tif]

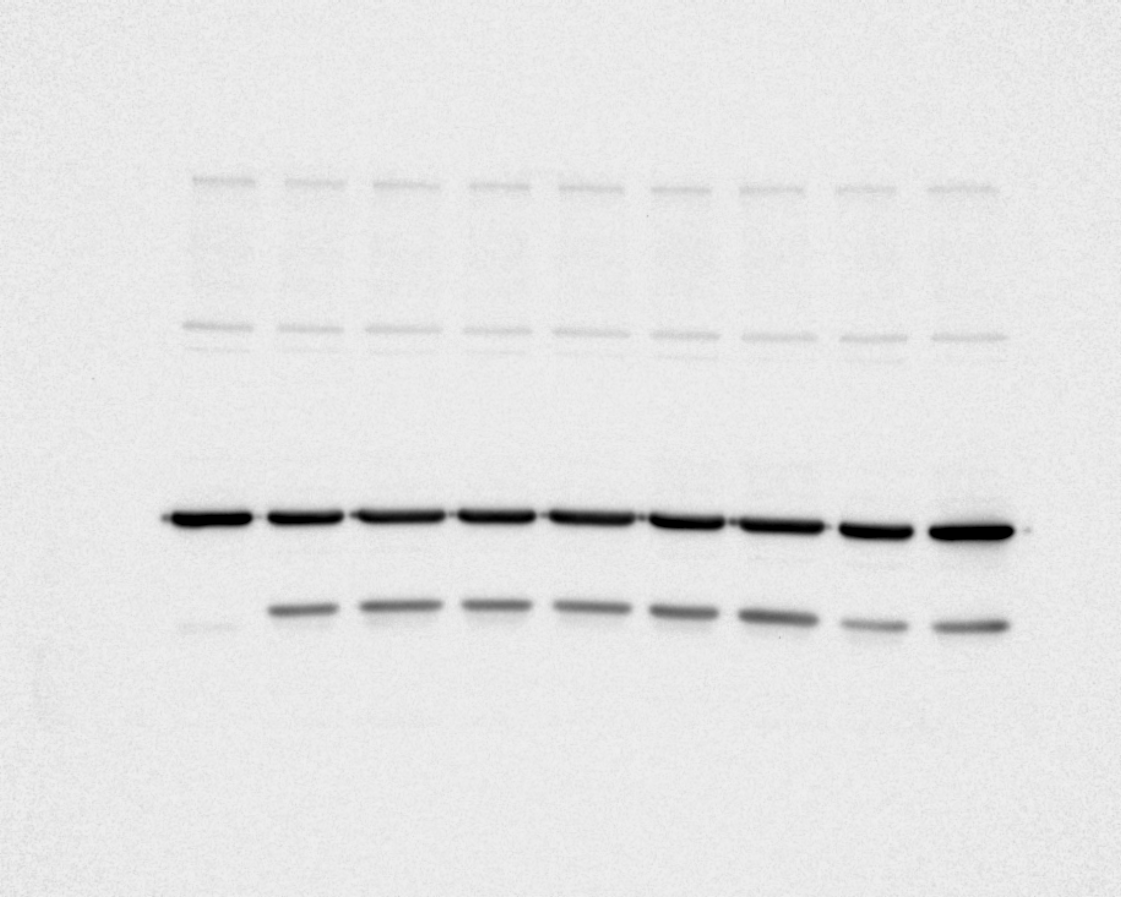

Supplement: Figure 6—source data 1. [file elife-83159-fig6-data1.zip › Figure 6-source data 1/ACTIN Figure 6-source data 1/Versteeg 2022-08-16 16h59m43s 46.495s(Chemiluminescence).tif]

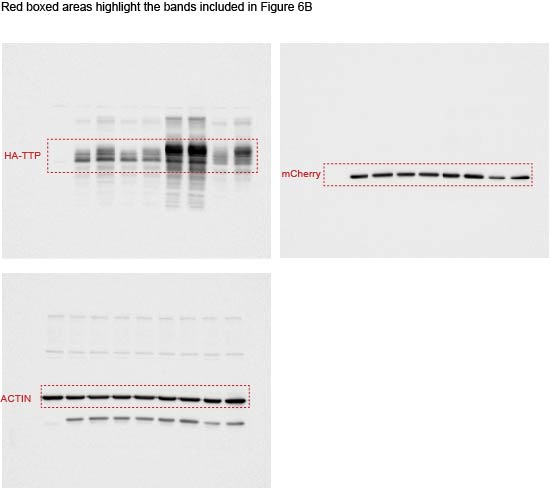

Supplement: Figure 6—source data 1. [file elife-83159-fig6-data1.zip › Figure 6-source data 1/Figure 6-source data 1.jpg]

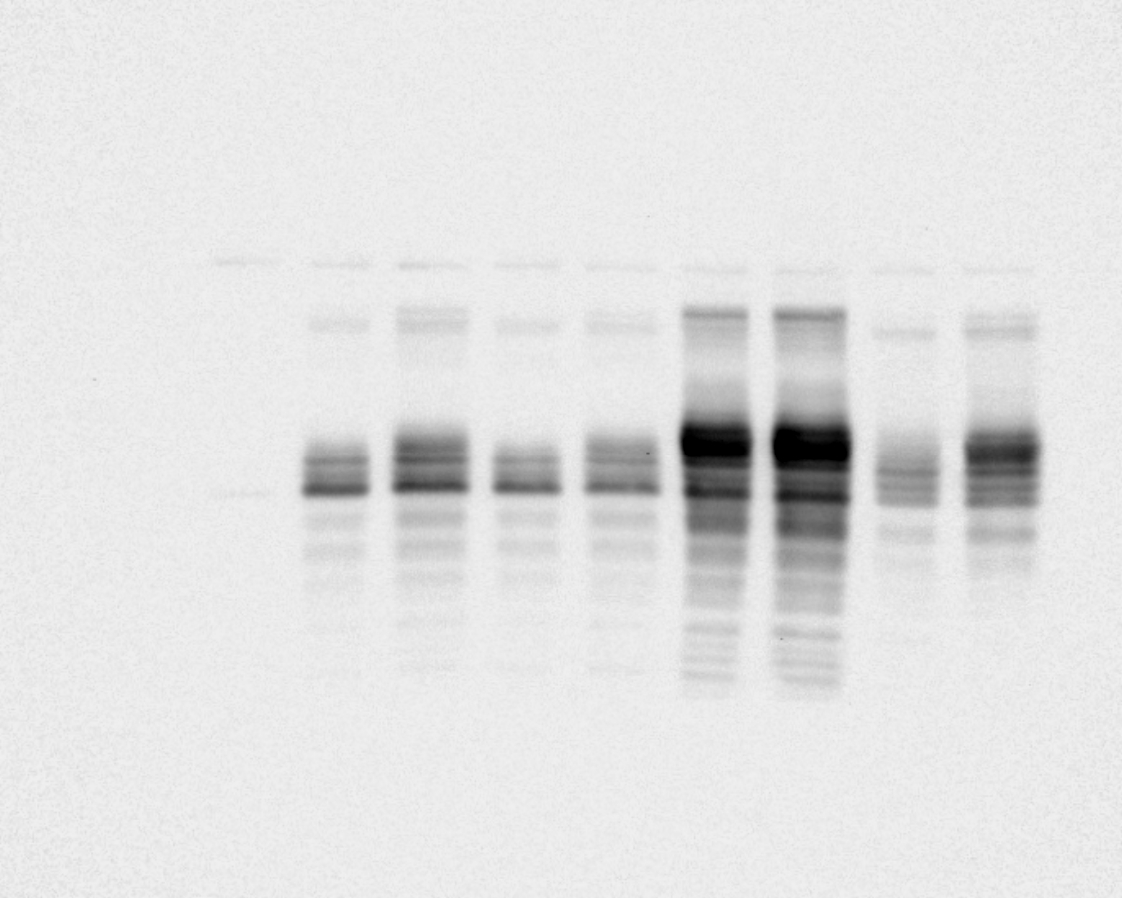

Supplement: Figure 6—source data 1. [file elife-83159-fig6-data1.zip › Figure 6-source data 1/HA-TTP Figure 6-source data 1/Versteeg 2022-08-15 18h37m13s 34.720s(Chemiluminescence).jpg]

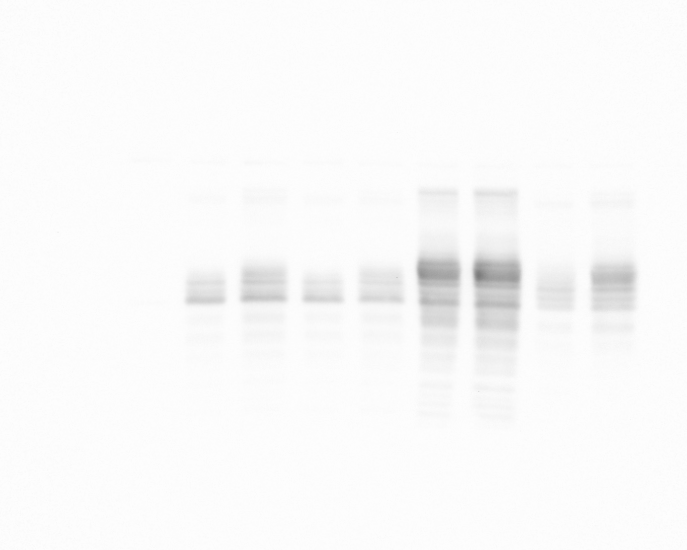

Supplement: Figure 6—source data 1. [file elife-83159-fig6-data1.zip › Figure 6-source data 1/HA-TTP Figure 6-source data 1/Versteeg 2022-08-15 18h37m13s 34.720s(Chemiluminescence).raw16.tif]

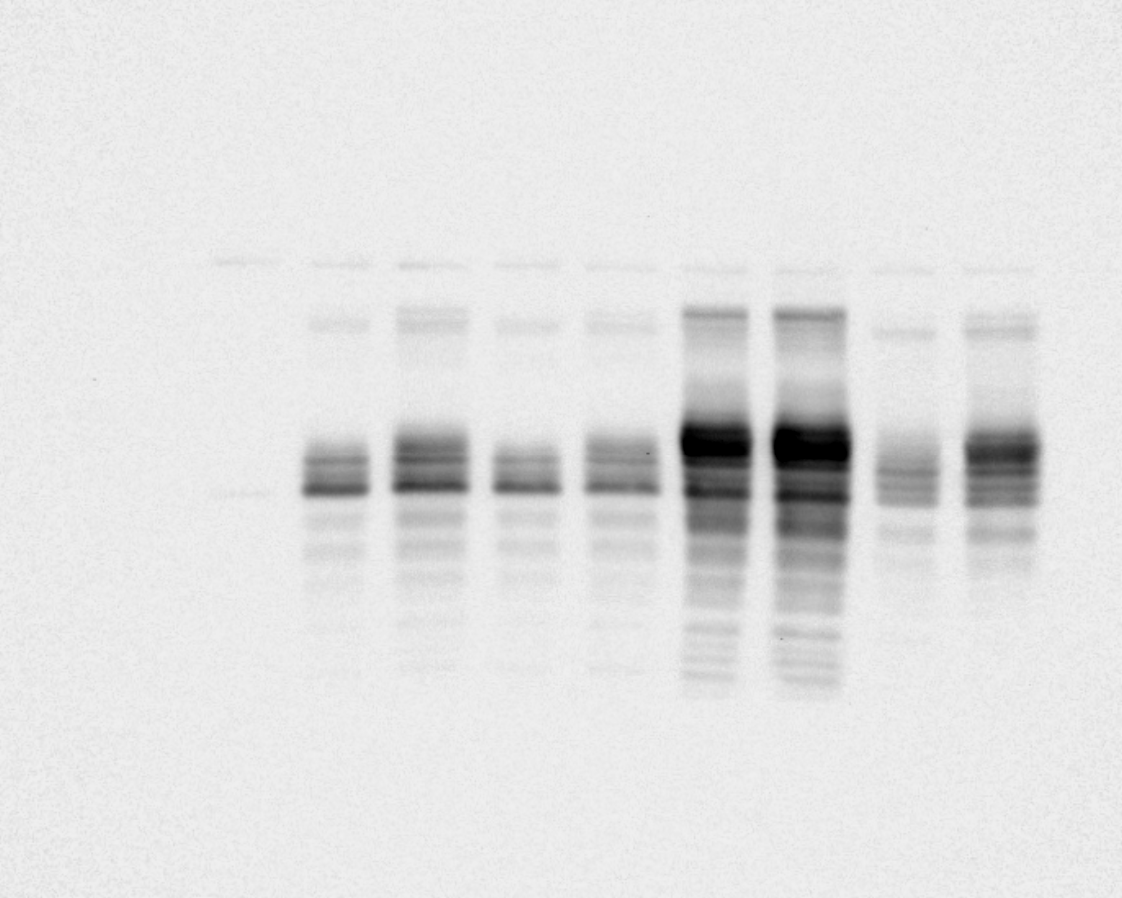

Supplement: Figure 6—source data 1. [file elife-83159-fig6-data1.zip › Figure 6-source data 1/HA-TTP Figure 6-source data 1/Versteeg 2022-08-15 18h37m13s 34.720s(Chemiluminescence).tif]

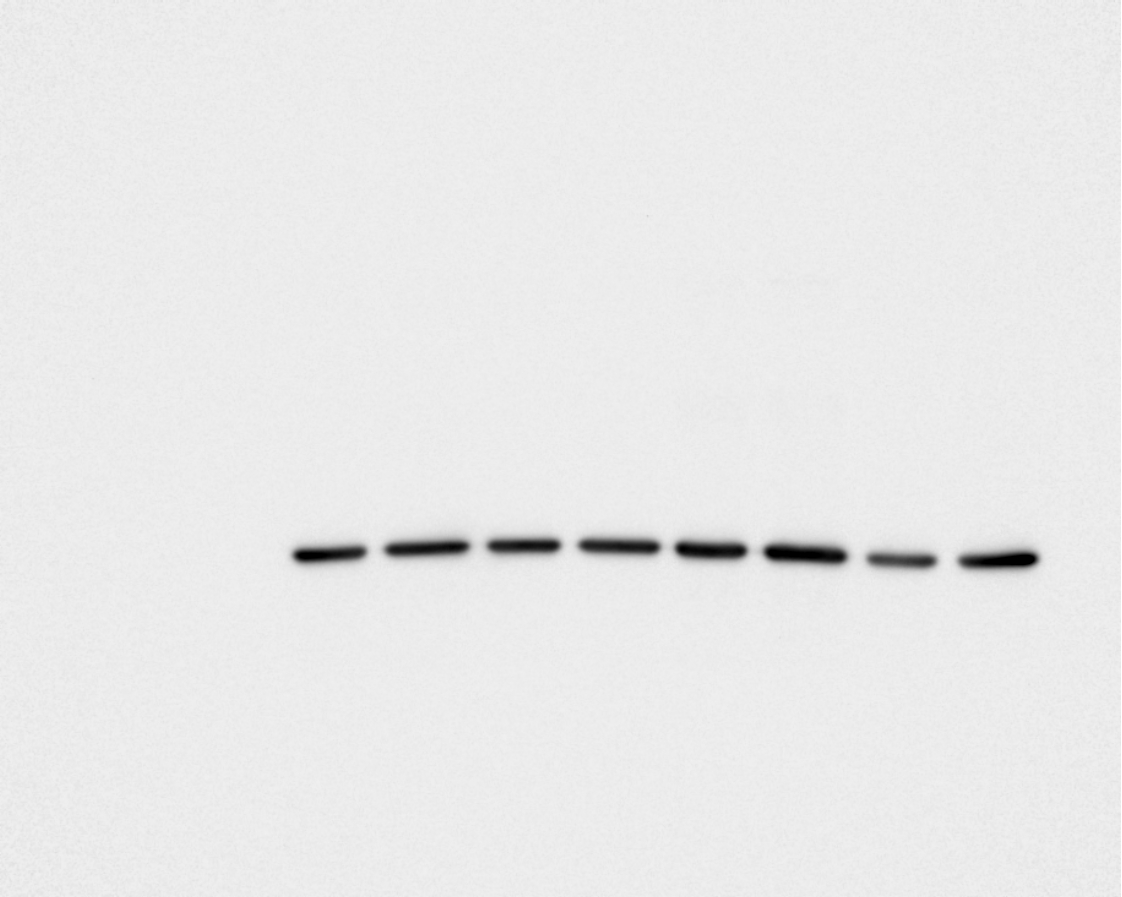

Supplement: Figure 6—source data 1. [file elife-83159-fig6-data1.zip › Figure 6-source data 1/mCherry Figure 6-source data 1/Versteeg 2022-08-16 12h55m04s 24.604s(Chemiluminescence).jpg]

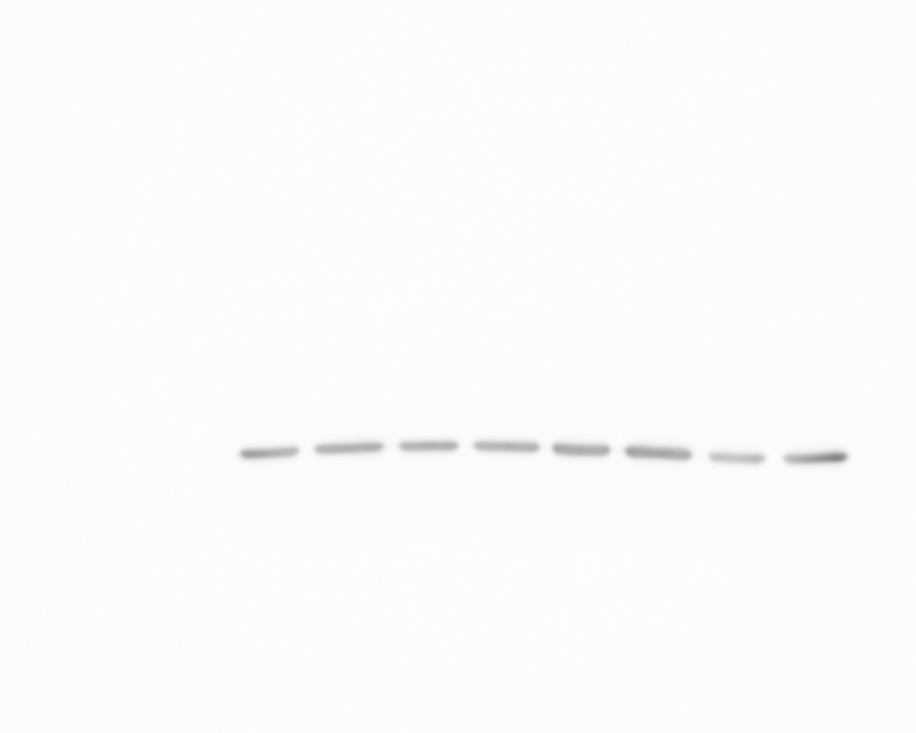

Supplement: Figure 6—source data 1. [file elife-83159-fig6-data1.zip › Figure 6-source data 1/mCherry Figure 6-source data 1/Versteeg 2022-08-16 12h55m04s 24.604s(Chemiluminescence).raw16.tif]

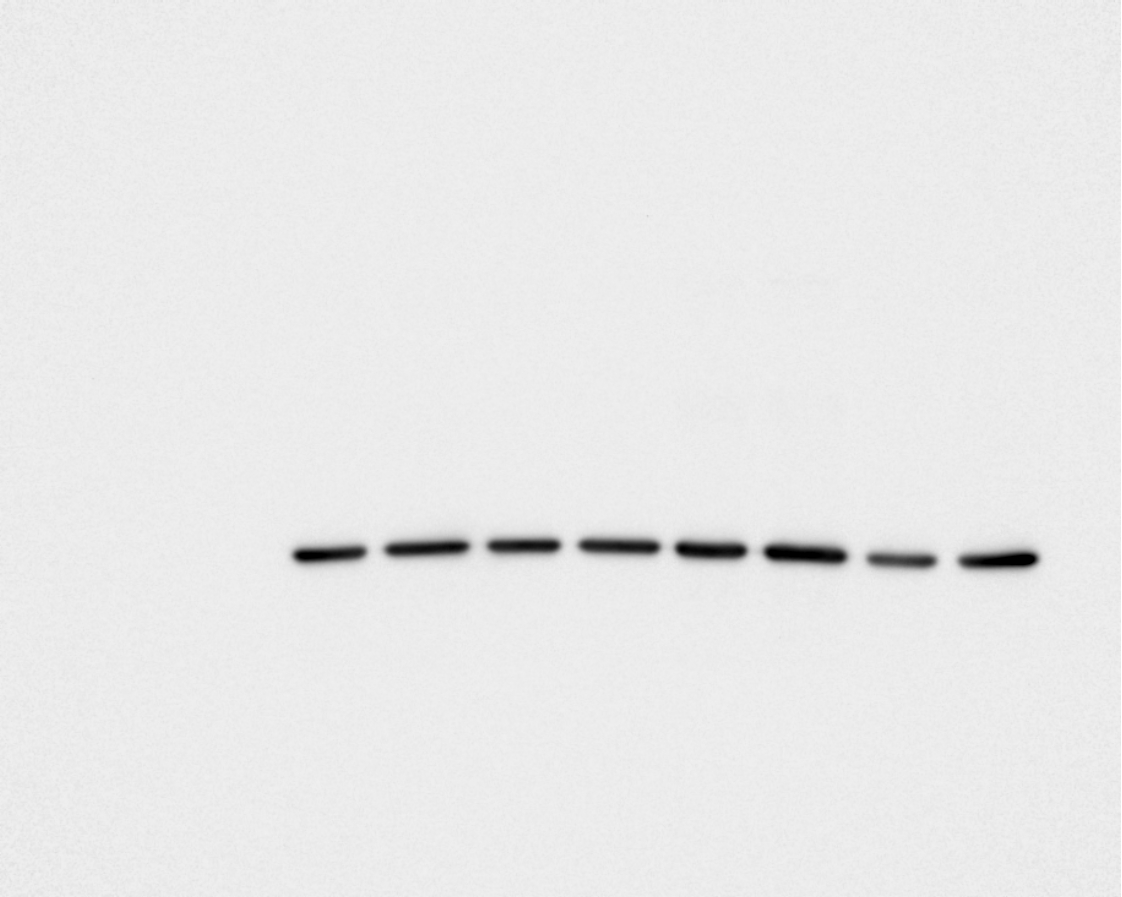

Supplement: Figure 6—source data 1. [file elife-83159-fig6-data1.zip › Figure 6-source data 1/mCherry Figure 6-source data 1/Versteeg 2022-08-16 12h55m04s 24.604s(Chemiluminescence).tif]

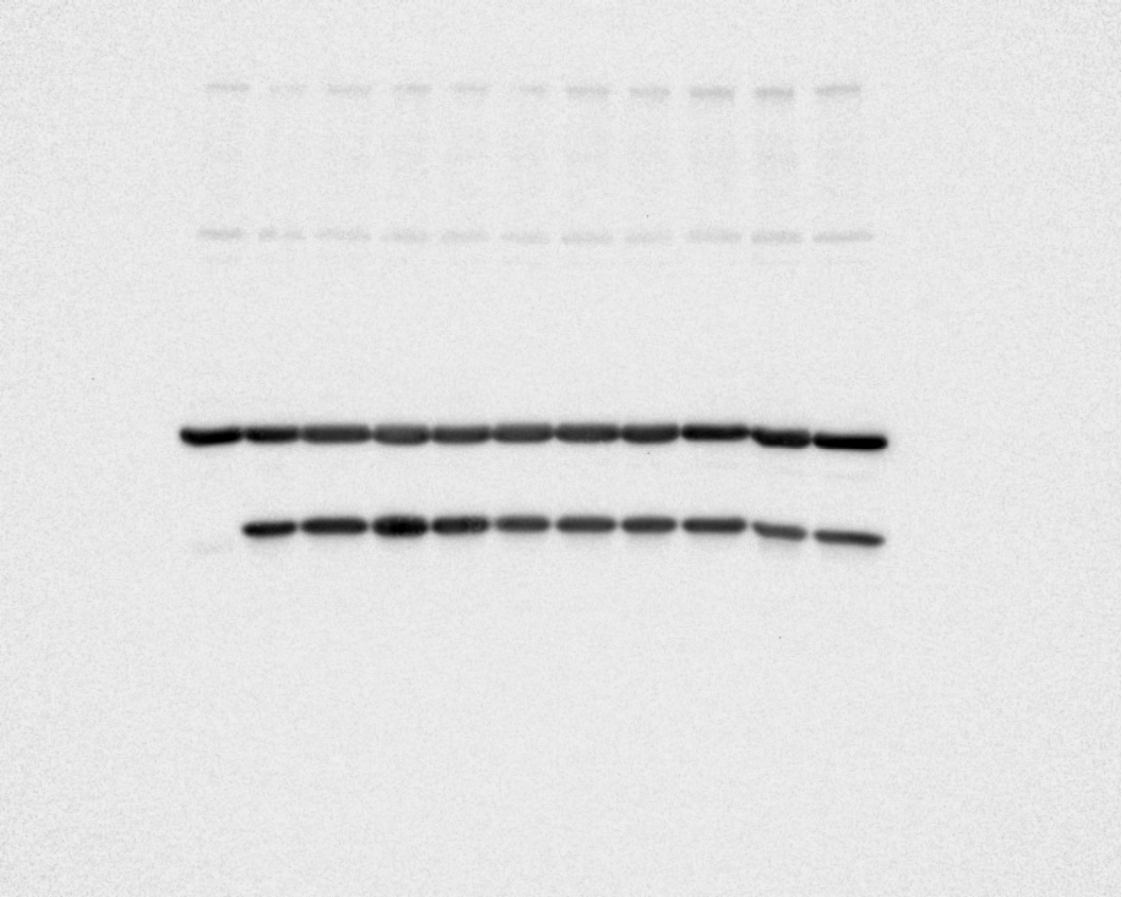

Supplement: Figure 6—source data 2. [file elife-83159-fig6-data2.zip › Figure 6-source data 2/ACTIN Figure 6-source data 2/Versteeg 2022-08-16 17h31m32s 55.594s(Chemiluminescence).jpg]

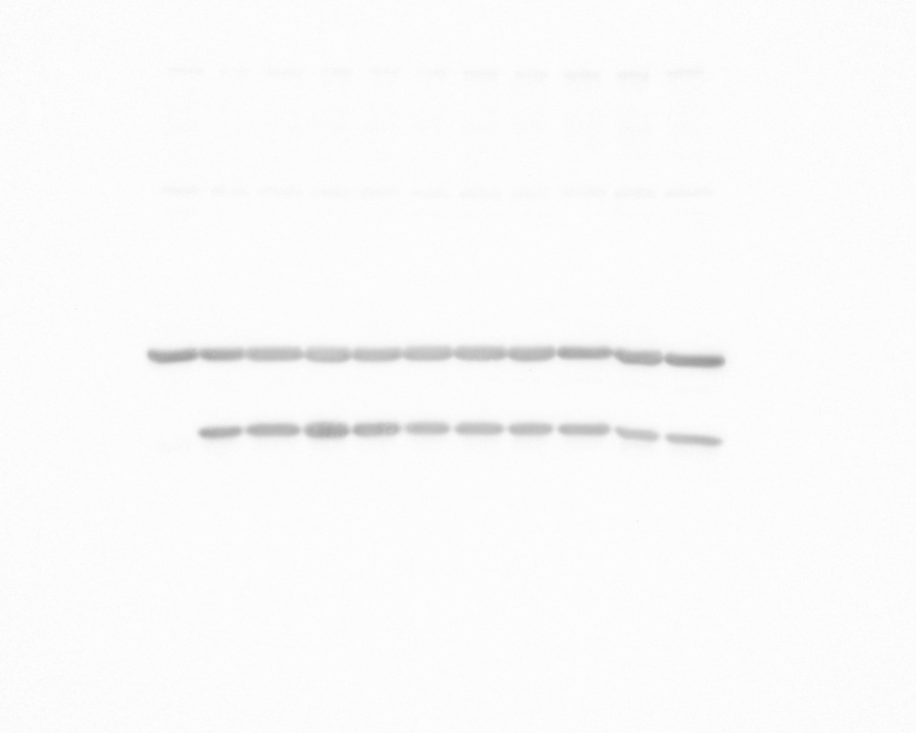

Supplement: Figure 6—source data 2. [file elife-83159-fig6-data2.zip › Figure 6-source data 2/ACTIN Figure 6-source data 2/Versteeg 2022-08-16 17h31m32s 55.594s(Chemiluminescence).raw16.tif]

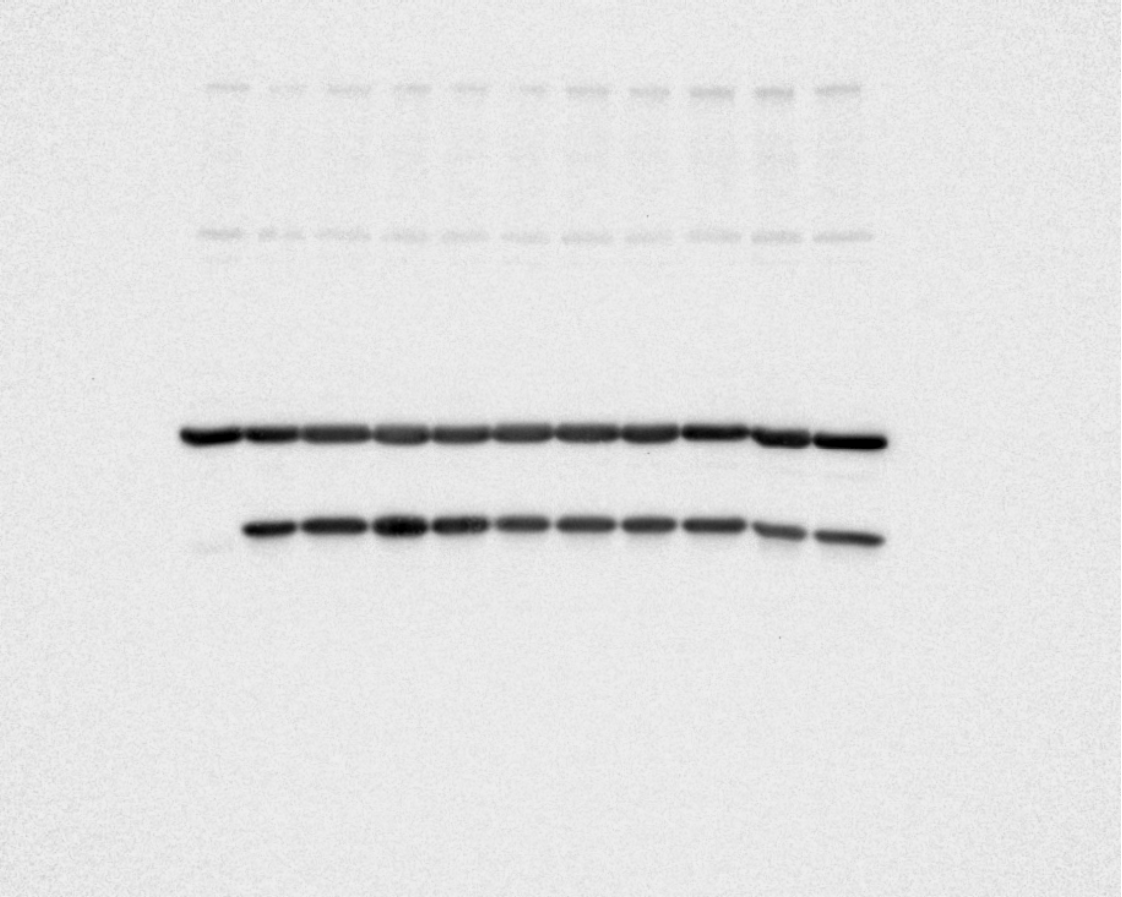

Supplement: Figure 6—source data 2. [file elife-83159-fig6-data2.zip › Figure 6-source data 2/ACTIN Figure 6-source data 2/Versteeg 2022-08-16 17h31m32s 55.594s(Chemiluminescence).tif]

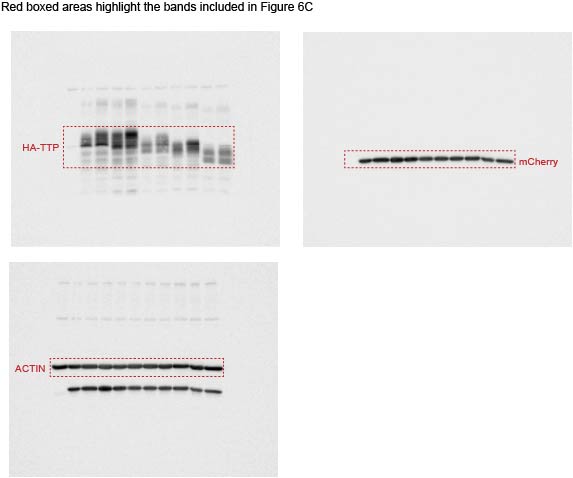

Supplement: Figure 6—source data 2. [file elife-83159-fig6-data2.zip › Figure 6-source data 2/Figure 6-source data 2.jpg]

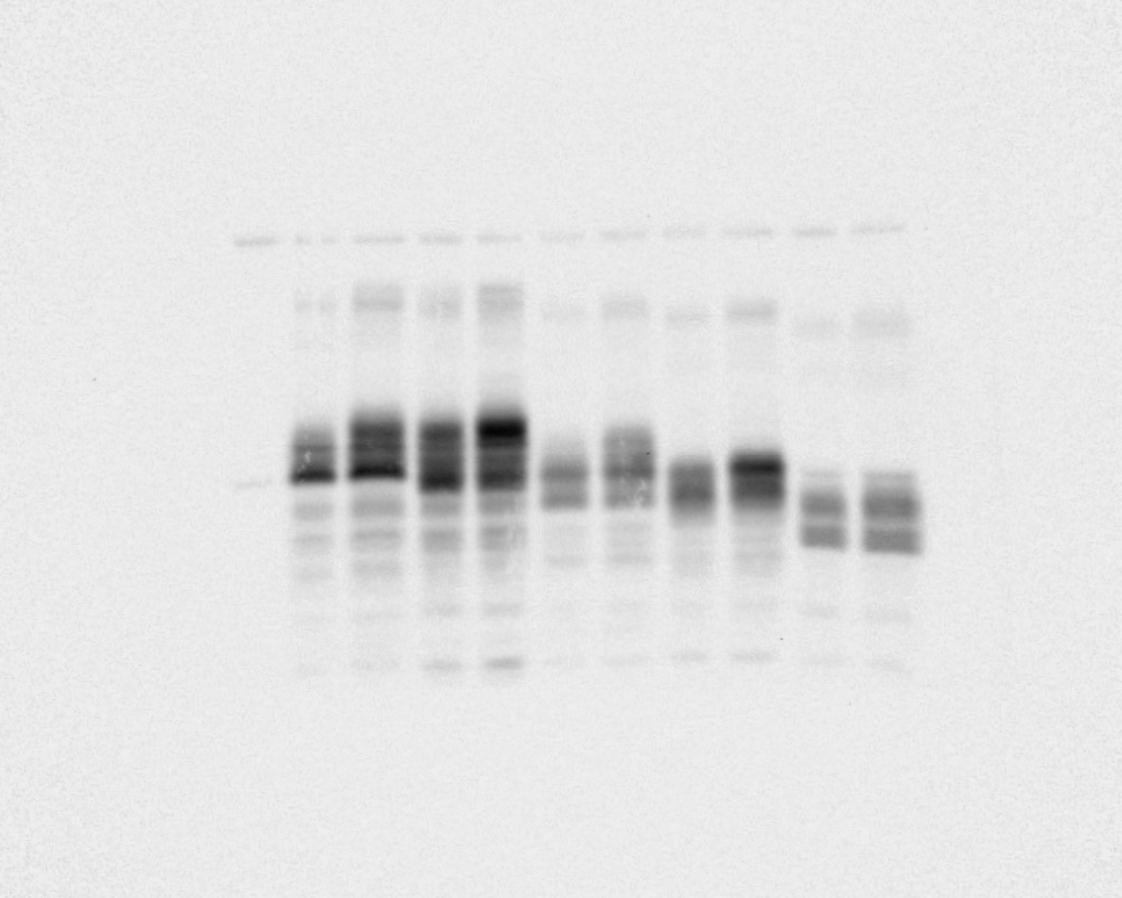

Supplement: Figure 6—source data 2. [file elife-83159-fig6-data2.zip › Figure 6-source data 2/HA-TTP Figure 6-source data 2/Versteeg 2022-08-15 18h15m04s 31.348s(Chemiluminescence).jpg]

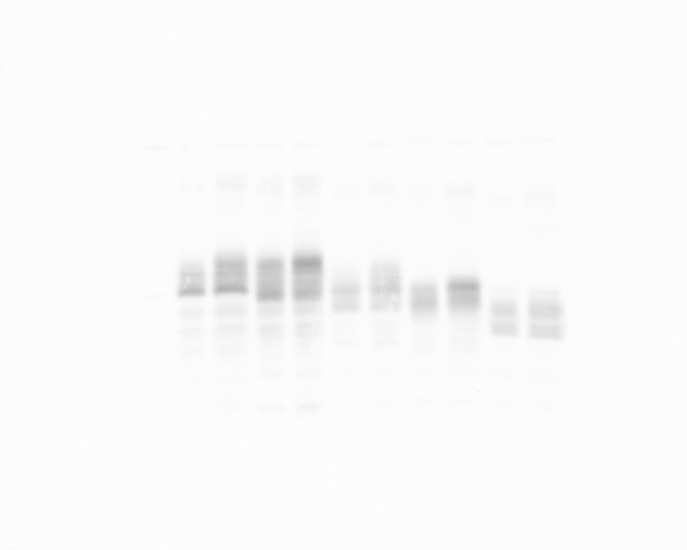

Supplement: Figure 6—source data 2. [file elife-83159-fig6-data2.zip › Figure 6-source data 2/HA-TTP Figure 6-source data 2/Versteeg 2022-08-15 18h15m04s 31.348s(Chemiluminescence).raw16.tif]

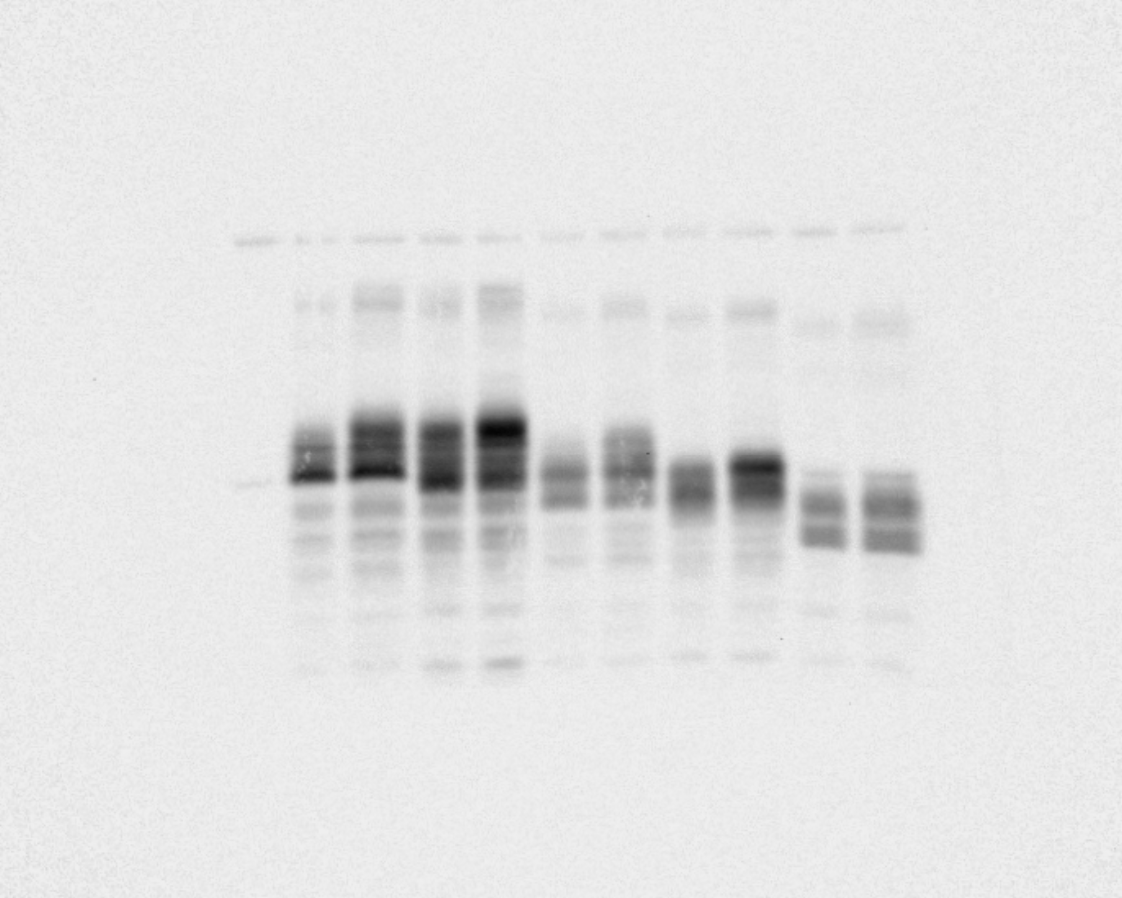

Supplement: Figure 6—source data 2. [file elife-83159-fig6-data2.zip › Figure 6-source data 2/HA-TTP Figure 6-source data 2/Versteeg 2022-08-15 18h15m04s 31.348s(Chemiluminescence).tif]

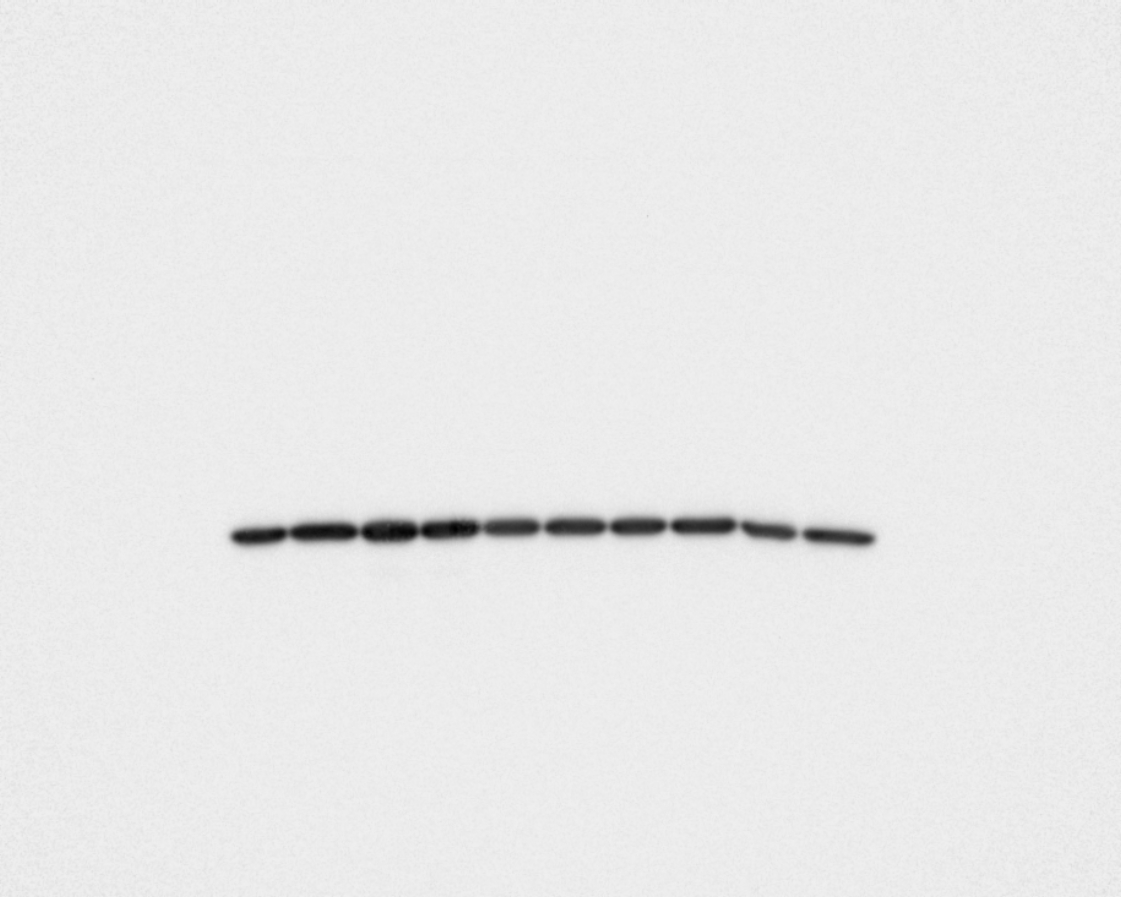

Supplement: Figure 6—source data 2. [file elife-83159-fig6-data2.zip › Figure 6-source data 2/mCherry Figure 6-source data 2/Versteeg 2022-08-16 13h21m37s 27.976s(Chemiluminescence).jpg]

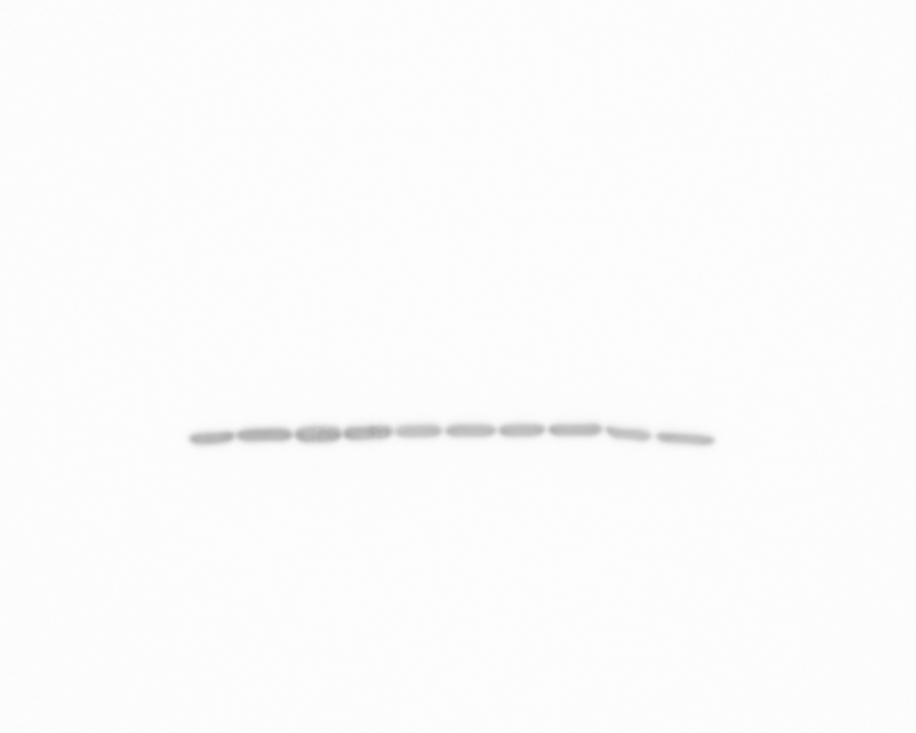

Supplement: Figure 6—source data 2. [file elife-83159-fig6-data2.zip › Figure 6-source data 2/mCherry Figure 6-source data 2/Versteeg 2022-08-16 13h21m37s 27.976s(Chemiluminescence).raw16.tif]

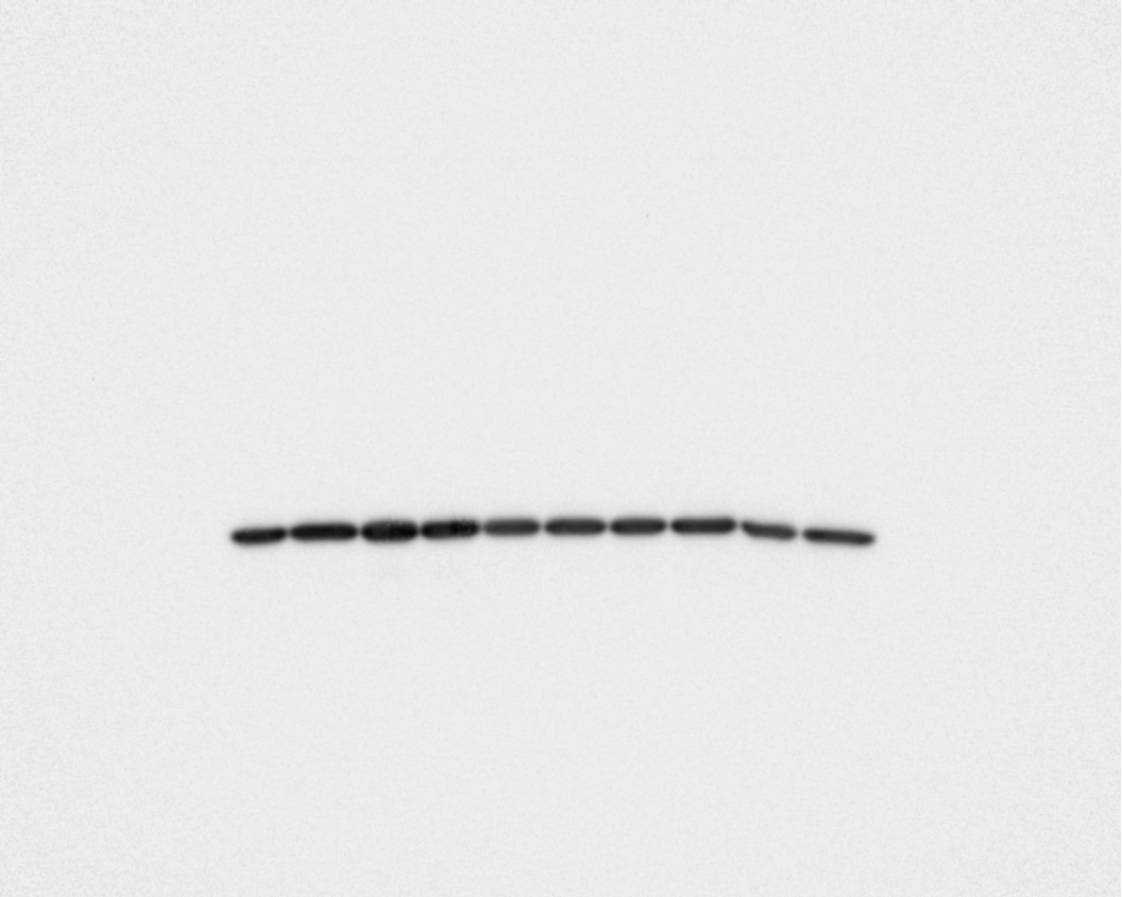

Supplement: Figure 6—source data 2. [file elife-83159-fig6-data2.zip › Figure 6-source data 2/mCherry Figure 6-source data 2/Versteeg 2022-08-16 13h21m37s 27.976s(Chemiluminescence).tif]

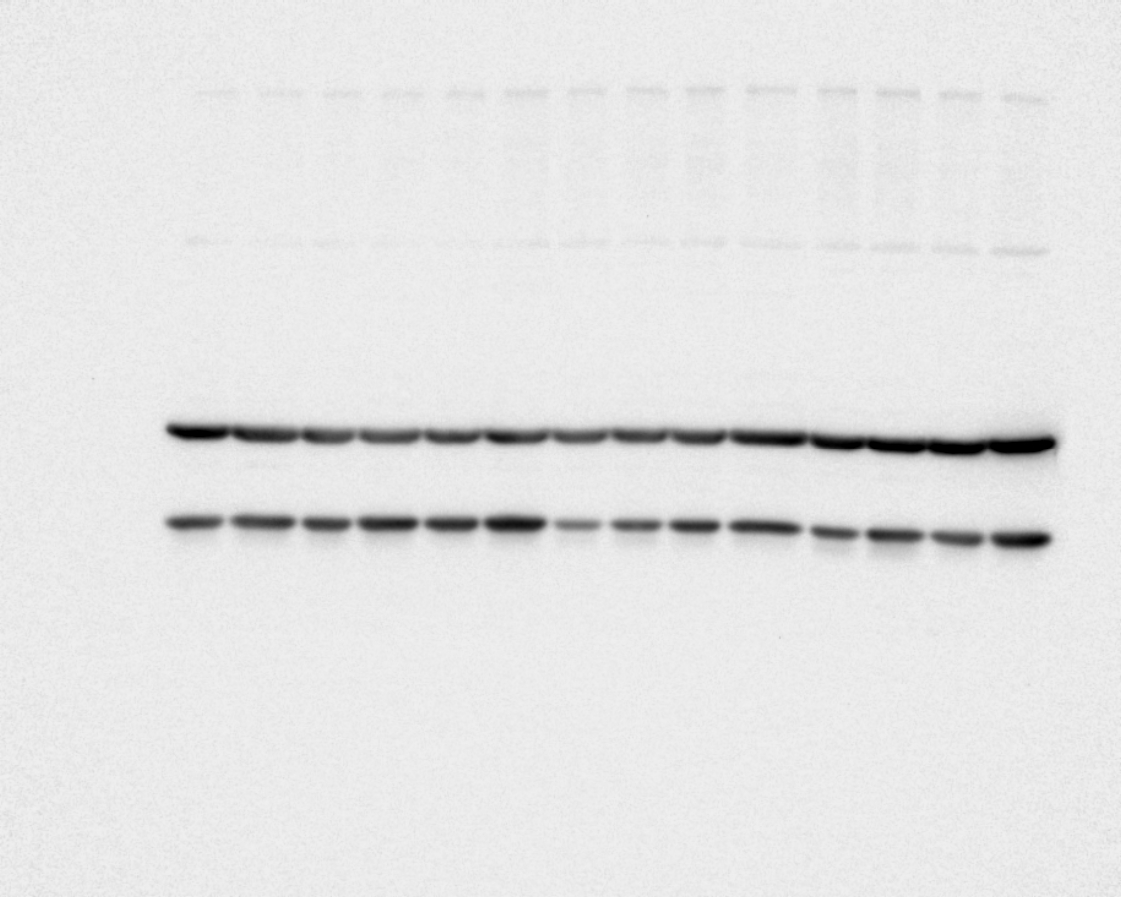

Supplement: Figure 6—source data 3. [file elife-83159-fig6-data3.zip › Figure 6-source data 3/ACTIN Figure 6-source data 3/Versteeg 2022-08-16 17h10m06s 37.396s(Chemiluminescence).jpg]

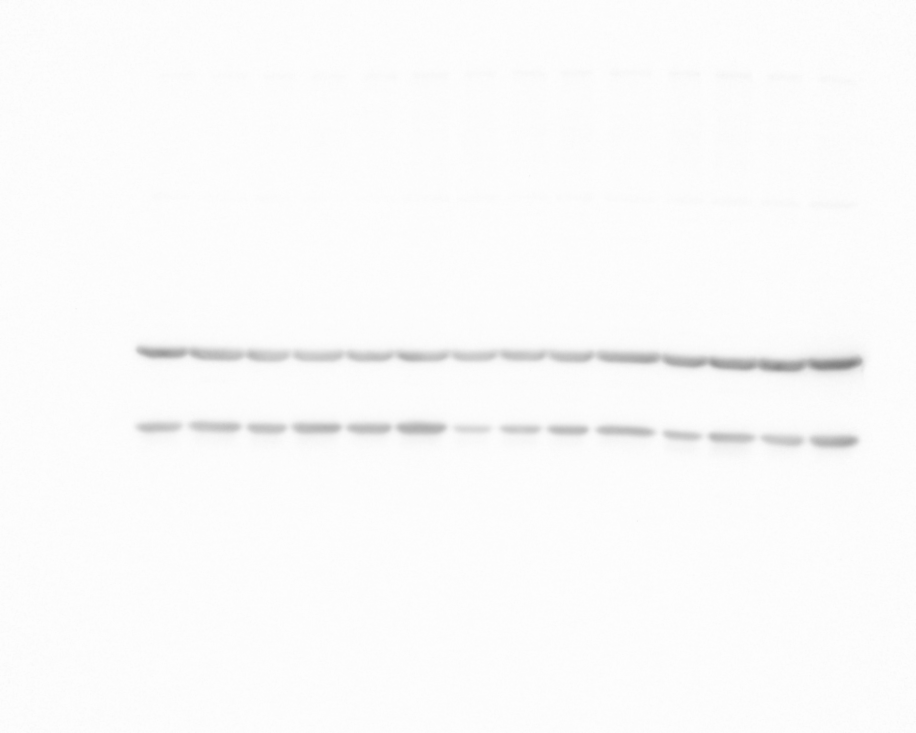

Supplement: Figure 6—source data 3. [file elife-83159-fig6-data3.zip › Figure 6-source data 3/ACTIN Figure 6-source data 3/Versteeg 2022-08-16 17h10m06s 37.396s(Chemiluminescence).raw16.tif]

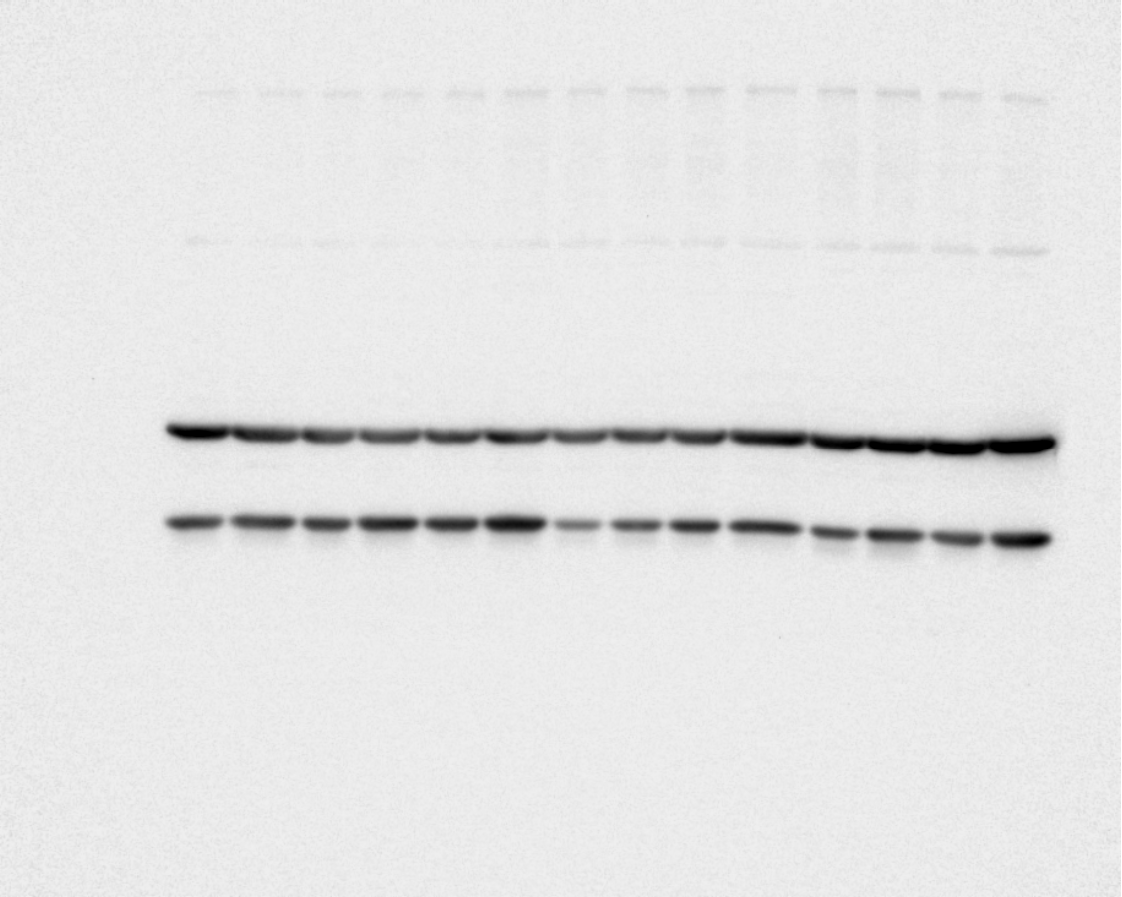

Supplement: Figure 6—source data 3. [file elife-83159-fig6-data3.zip › Figure 6-source data 3/ACTIN Figure 6-source data 3/Versteeg 2022-08-16 17h10m06s 37.396s(Chemiluminescence).tif]

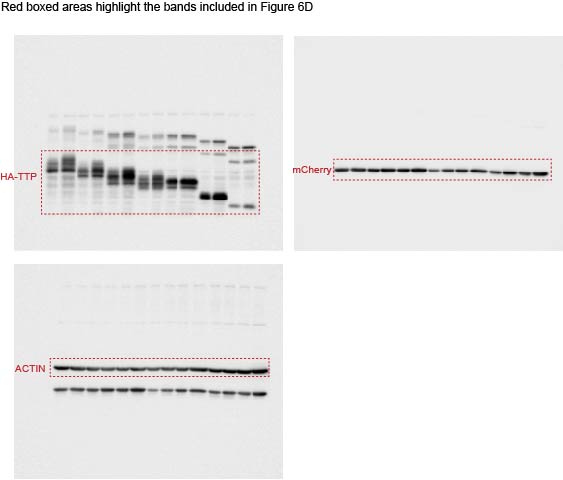

Supplement: Figure 6—source data 3. [file elife-83159-fig6-data3.zip › Figure 6-source data 3/Figure 6-source data 3.jpg]

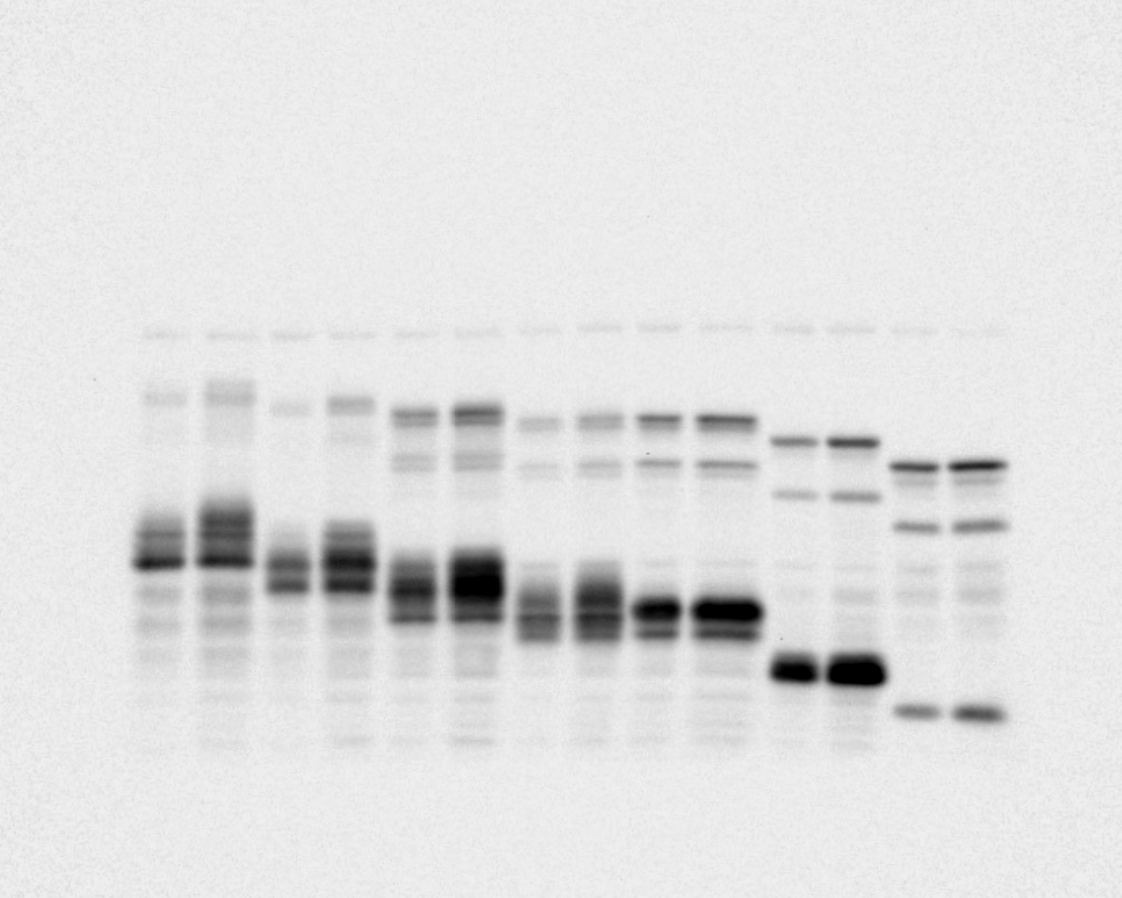

Supplement: Figure 6—source data 3. [file elife-83159-fig6-data3.zip › Figure 6-source data 3/HA-TTP Figure 6-source data 3/Versteeg 2022-08-15 18h44m42s 31.348s(Chemiluminescence).jpg]

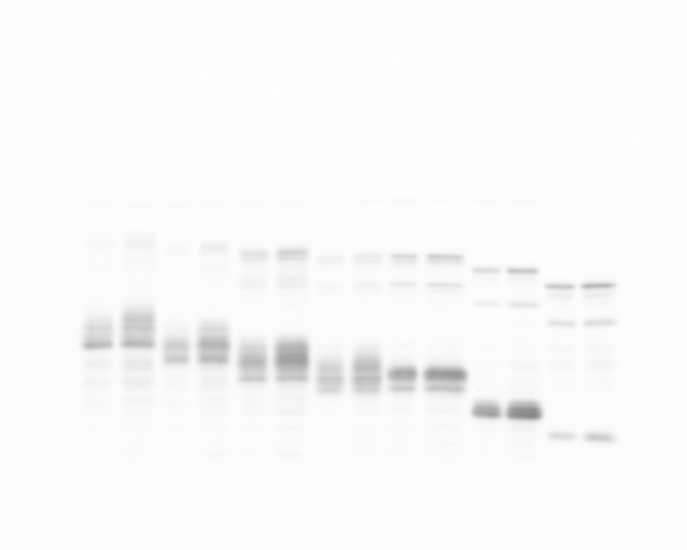

Supplement: Figure 6—source data 3. [file elife-83159-fig6-data3.zip › Figure 6-source data 3/HA-TTP Figure 6-source data 3/Versteeg 2022-08-15 18h44m42s 31.348s(Chemiluminescence).raw16.tif]

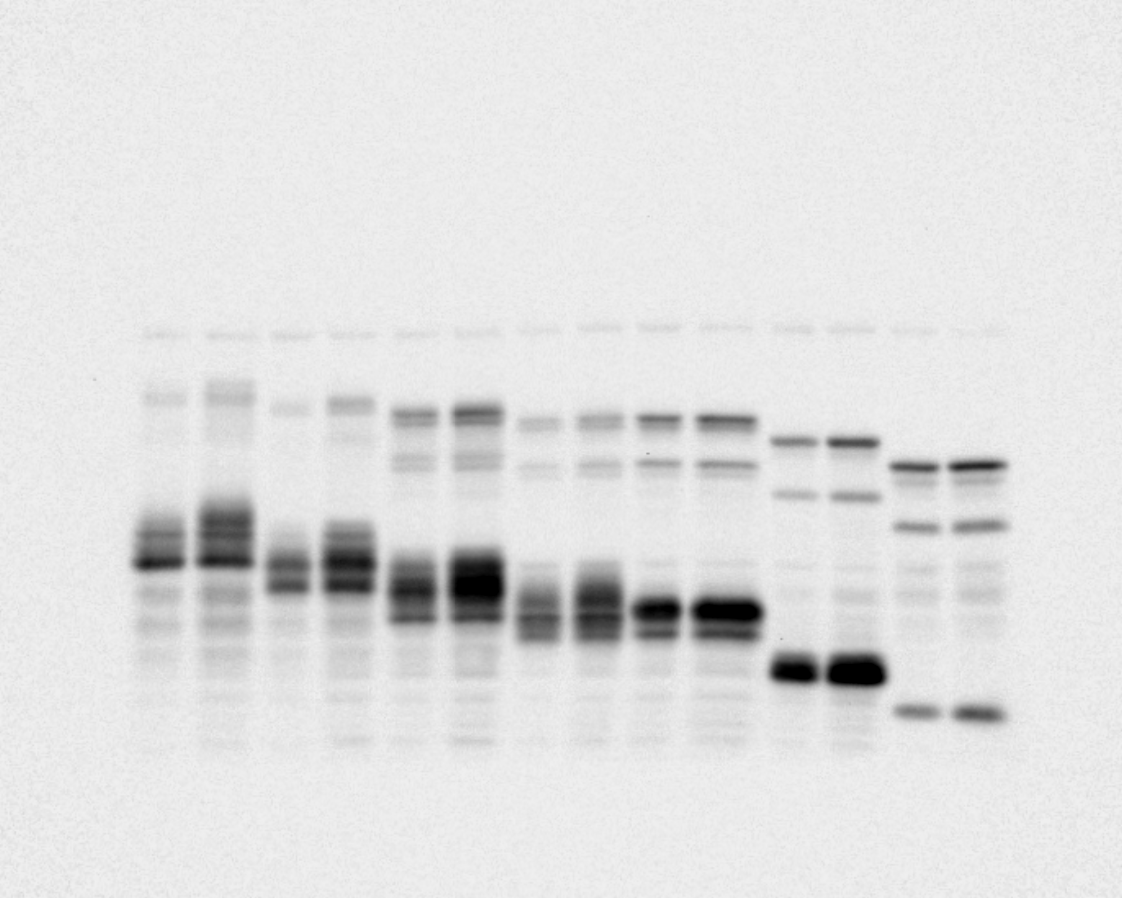

Supplement: Figure 6—source data 3. [file elife-83159-fig6-data3.zip › Figure 6-source data 3/HA-TTP Figure 6-source data 3/Versteeg 2022-08-15 18h44m42s 31.348s(Chemiluminescence).tif]

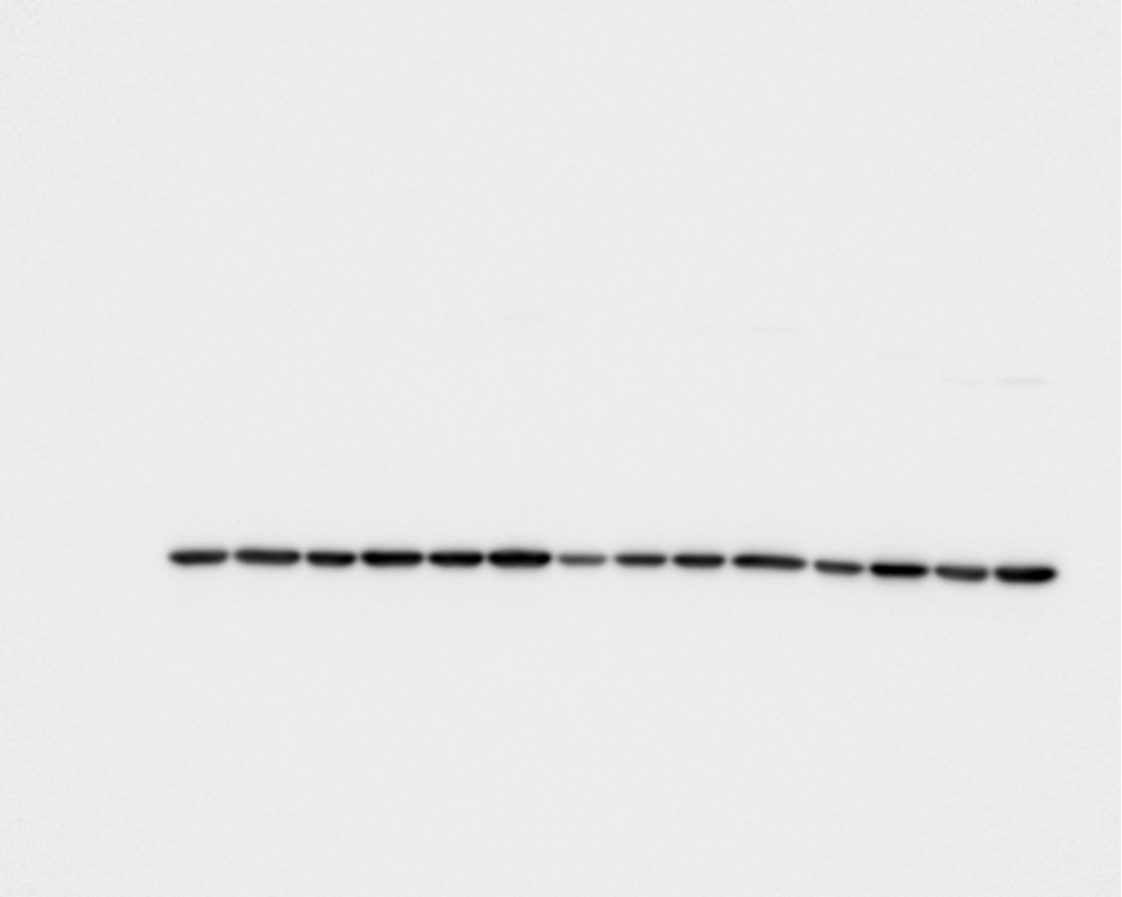

Supplement: Figure 6—source data 3. [file elife-83159-fig6-data3.zip › Figure 6-source data 3/mCherry Figure 6-source data 3/Versteeg 2022-08-16 12h48m05s 16.201s(Chemiluminescence).jpg]

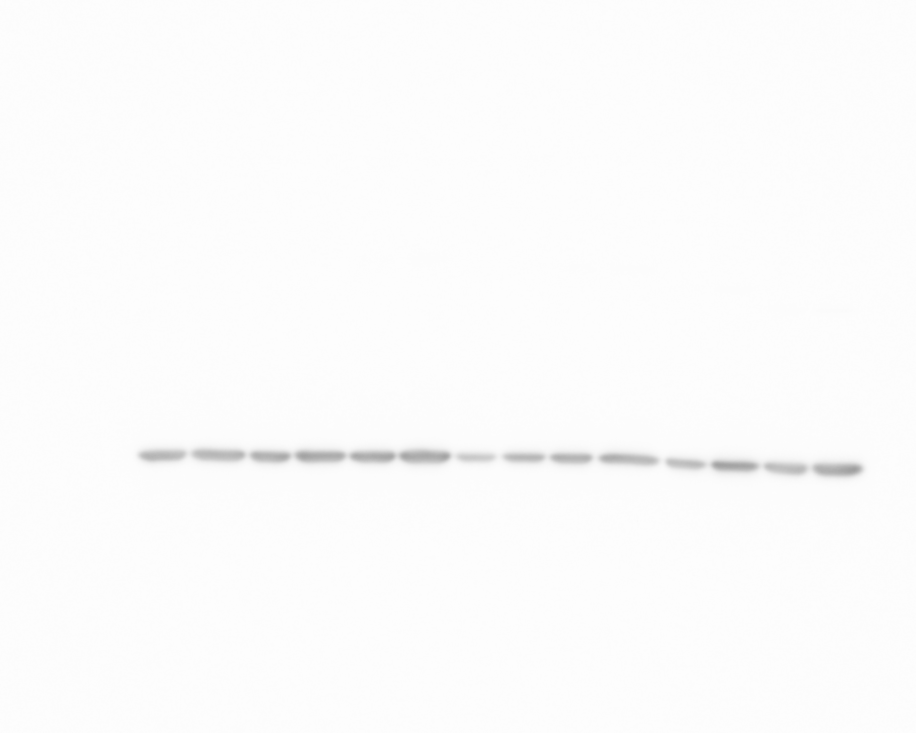

Supplement: Figure 6—source data 3. [file elife-83159-fig6-data3.zip › Figure 6-source data 3/mCherry Figure 6-source data 3/Versteeg 2022-08-16 12h48m05s 16.201s(Chemiluminescence).raw16.tif]

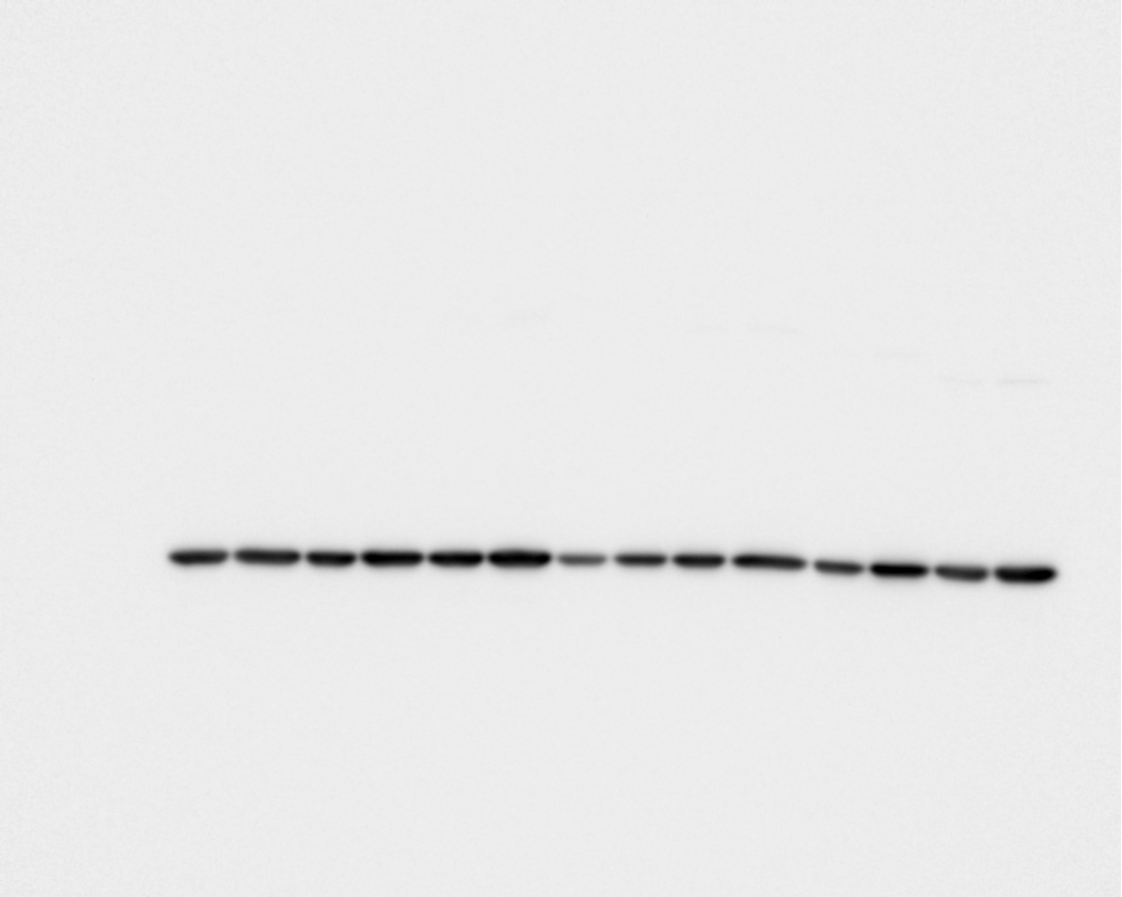

Supplement: Figure 6—source data 3. [file elife-83159-fig6-data3.zip › Figure 6-source data 3/mCherry Figure 6-source data 3/Versteeg 2022-08-16 12h48m05s 16.201s(Chemiluminescence).tif]

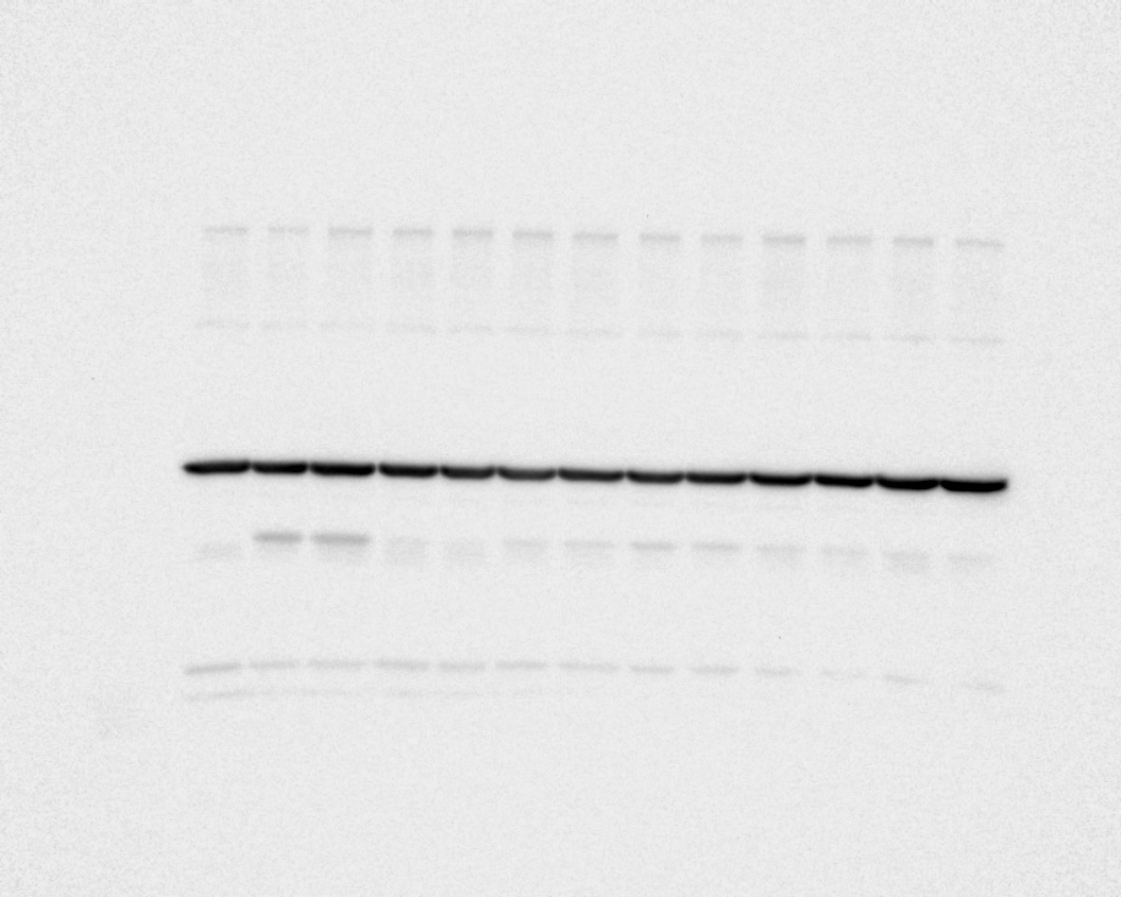

Supplement: Figure 6—source data 4. [file elife-83159-fig6-data4.zip › Figure 6-source data 4/ACTIN Figure 6-source data 4/Versteeg 2022-08-16 17h16m02s 40.429s(Chemiluminescence).jpg]

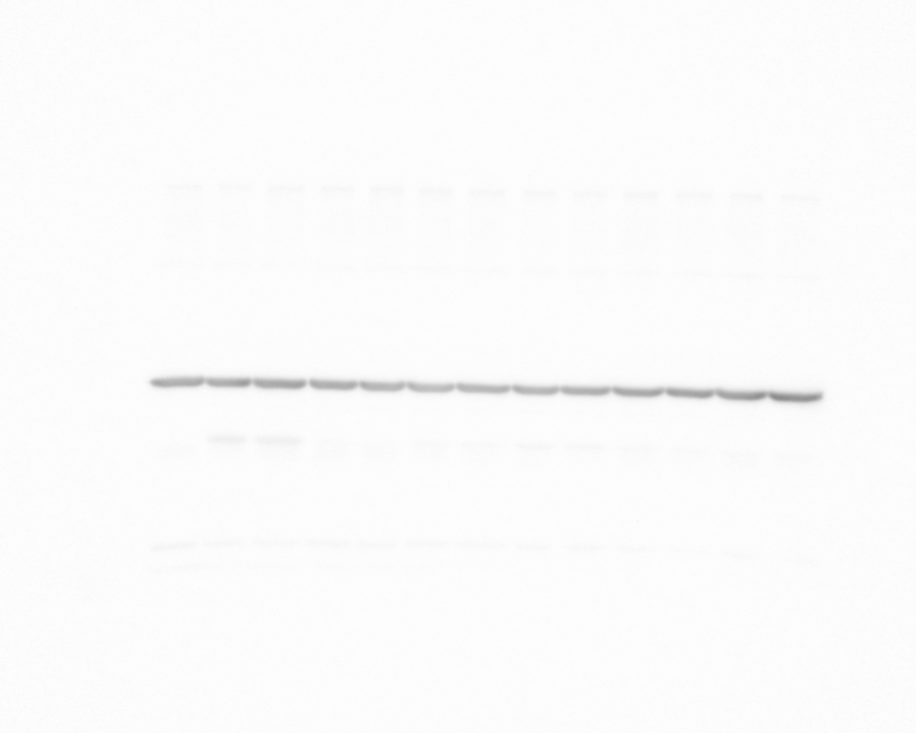

Supplement: Figure 6—source data 4. [file elife-83159-fig6-data4.zip › Figure 6-source data 4/ACTIN Figure 6-source data 4/Versteeg 2022-08-16 17h16m02s 40.429s(Chemiluminescence).raw16.tif]

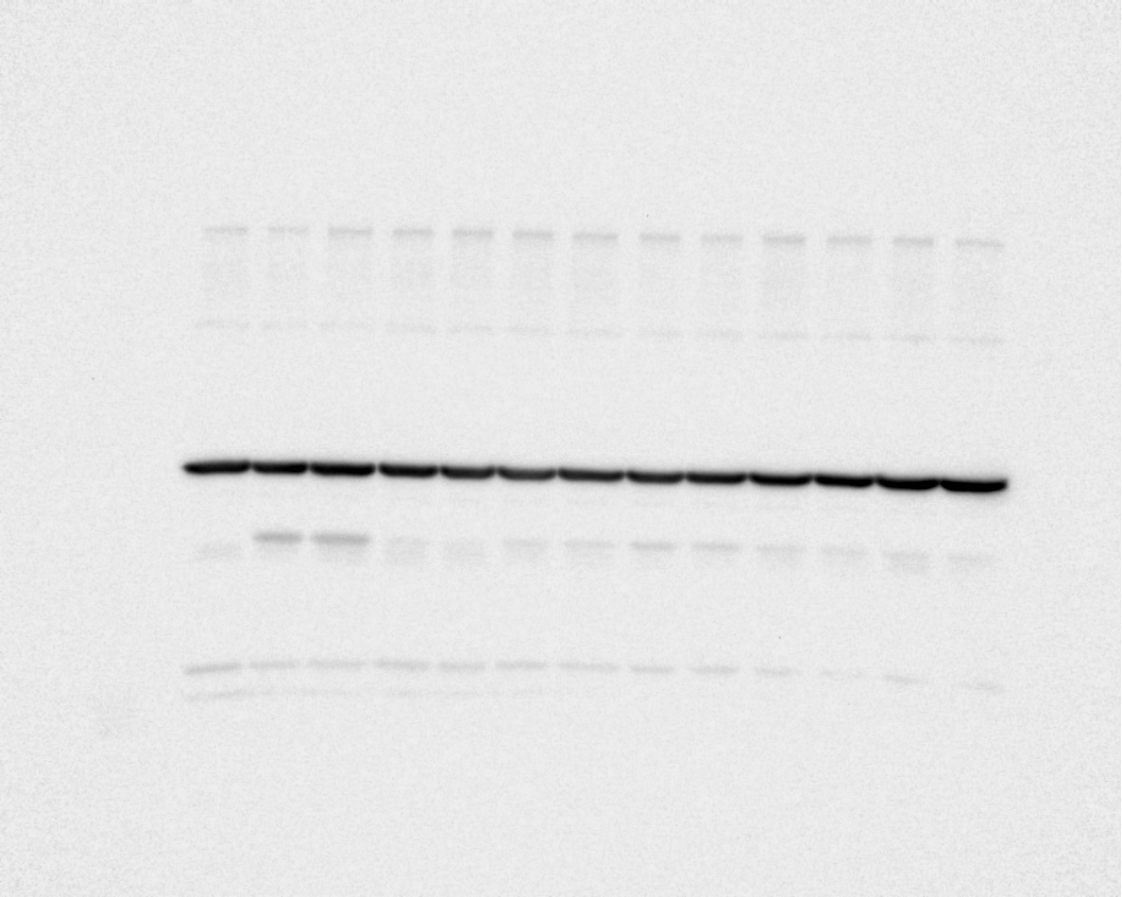

Supplement: Figure 6—source data 4. [file elife-83159-fig6-data4.zip › Figure 6-source data 4/ACTIN Figure 6-source data 4/Versteeg 2022-08-16 17h16m02s 40.429s(Chemiluminescence).tif]

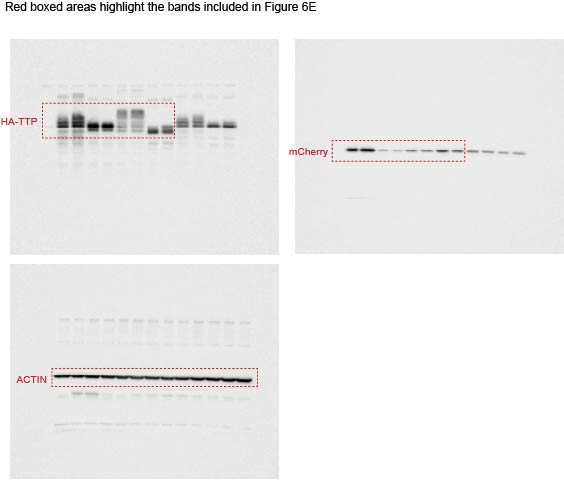

Supplement: Figure 6—source data 4. [file elife-83159-fig6-data4.zip › Figure 6-source data 4/Figure 6-source data 4.jpg]

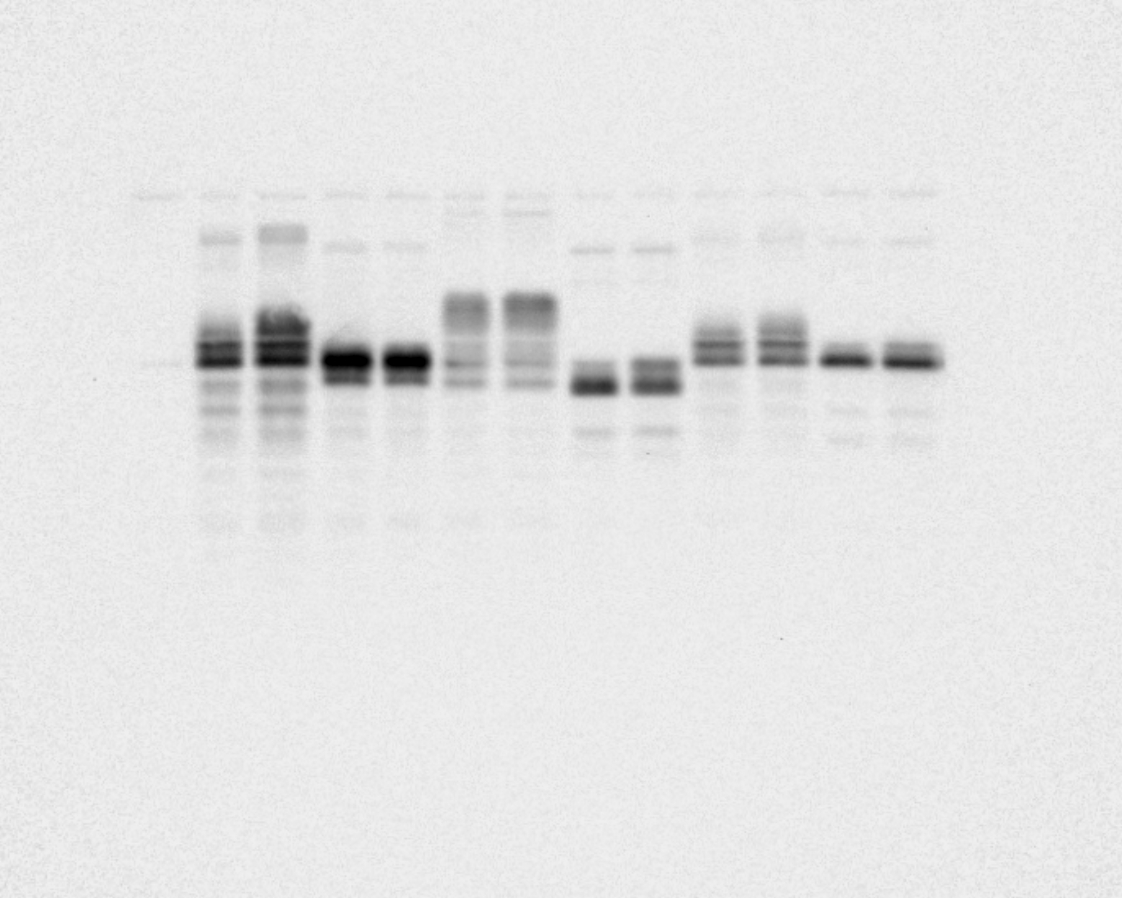

Supplement: Figure 6—source data 4. [file elife-83159-fig6-data4.zip › Figure 6-source data 4/HA-TTP Figure 6-source data 4/Versteeg 2022-08-15 18h30m56s 38.092s(Chemiluminescence).jpg]

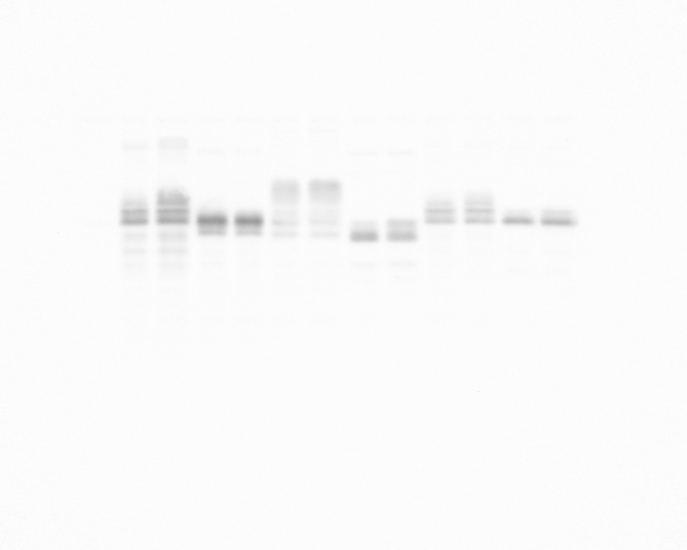

Supplement: Figure 6—source data 4. [file elife-83159-fig6-data4.zip › Figure 6-source data 4/HA-TTP Figure 6-source data 4/Versteeg 2022-08-15 18h30m56s 38.092s(Chemiluminescence).raw16.tif]

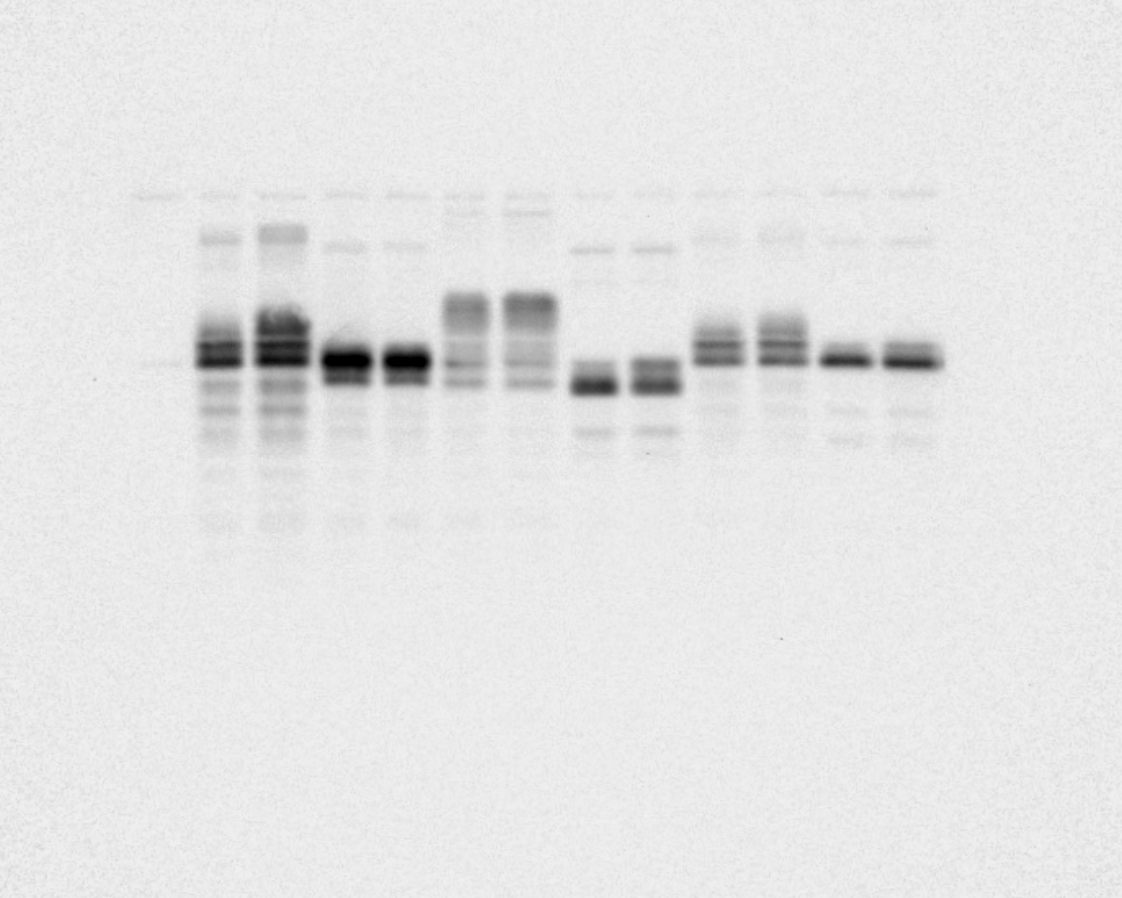

Supplement: Figure 6—source data 4. [file elife-83159-fig6-data4.zip › Figure 6-source data 4/HA-TTP Figure 6-source data 4/Versteeg 2022-08-15 18h30m56s 38.092s(Chemiluminescence).tif]

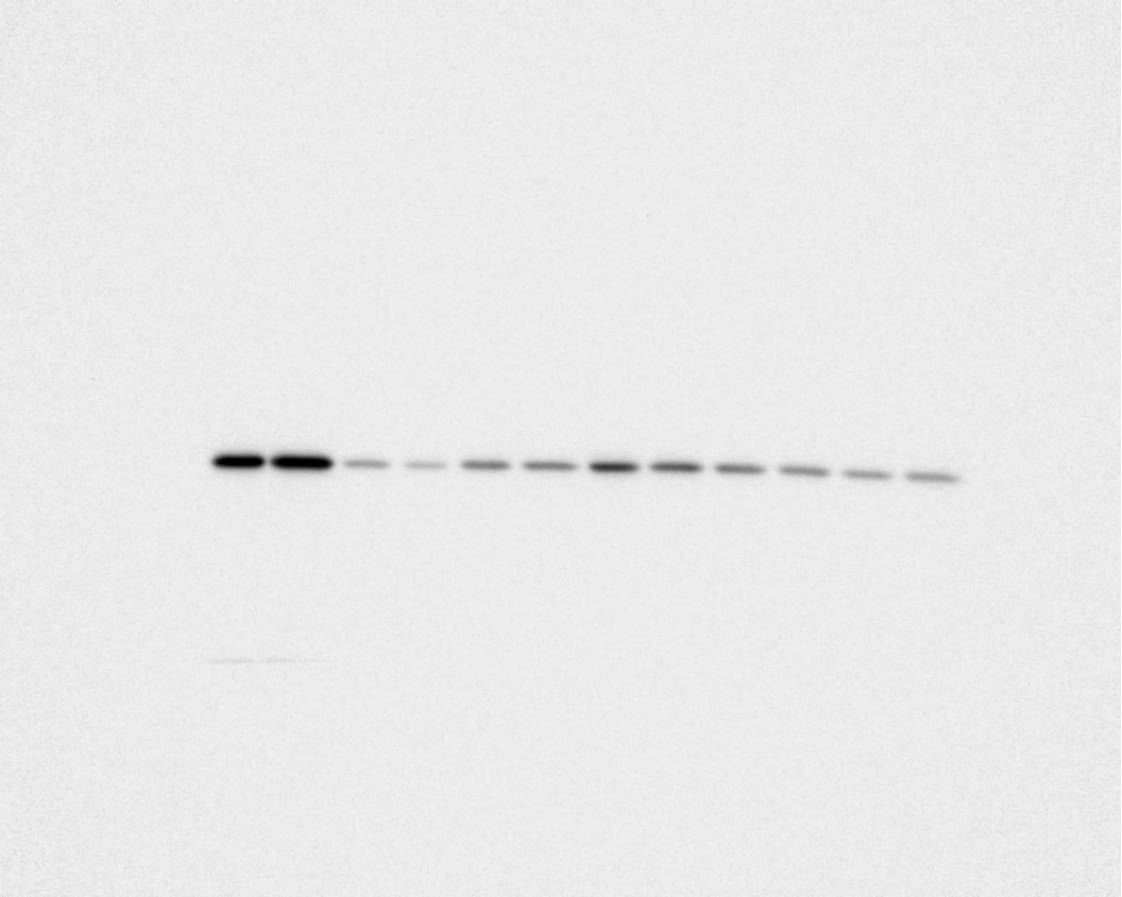

Supplement: Figure 6—source data 4. [file elife-83159-fig6-data4.zip › Figure 6-source data 4/mCherry Figure 6-source data 4/Versteeg 2022-08-16 13h47m20s 38.092s(Chemiluminescence).jpg]

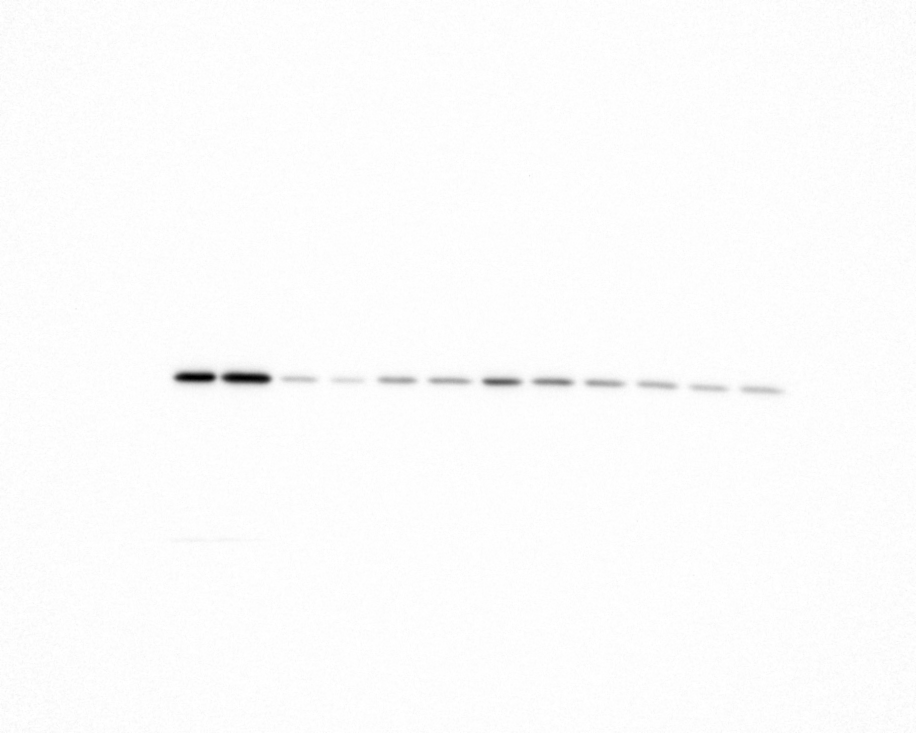

Supplement: Figure 6—source data 4. [file elife-83159-fig6-data4.zip › Figure 6-source data 4/mCherry Figure 6-source data 4/Versteeg 2022-08-16 13h47m20s 38.092s(Chemiluminescence).raw16.tif]

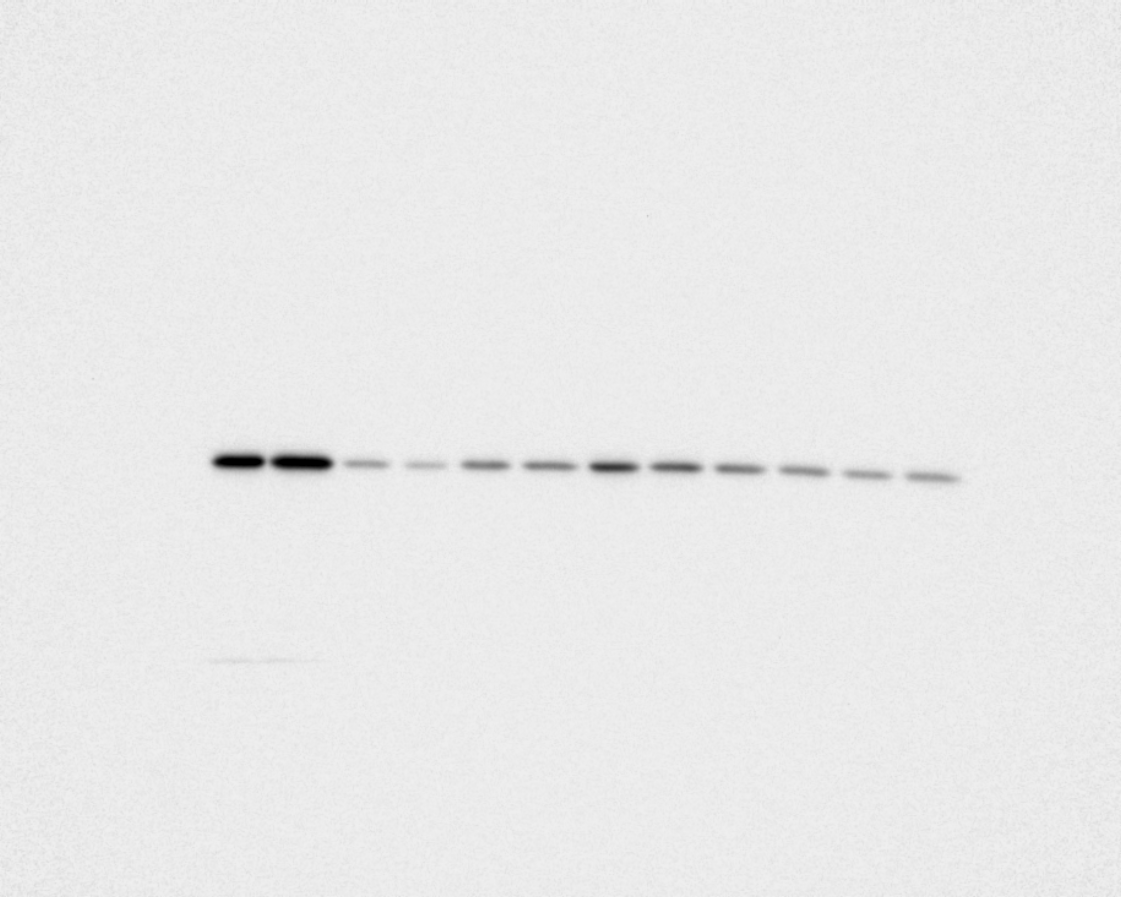

Supplement: Figure 6—source data 4. [file elife-83159-fig6-data4.zip › Figure 6-source data 4/mCherry Figure 6-source data 4/Versteeg 2022-08-16 13h47m20s 38.092s(Chemiluminescence).tif]

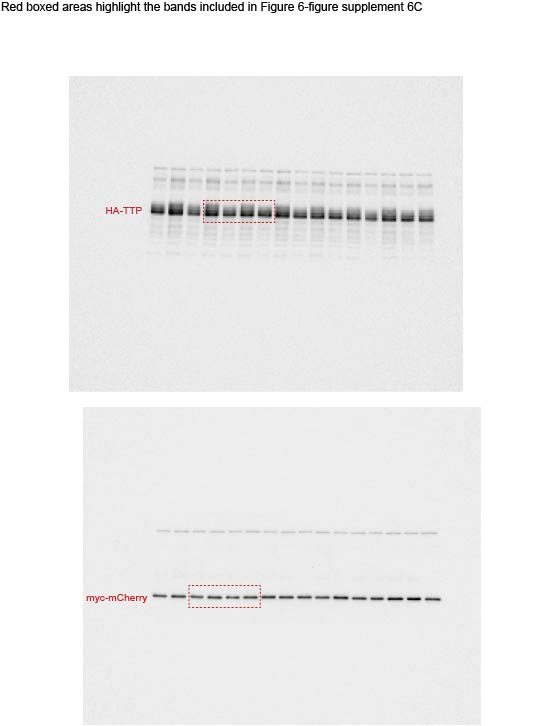

Supplement: Figure 6—figure supplement 1—source data 1. [file elife-83159-fig6-figsupp1-data1.zip › Figure 6-figure supplement 1-source data 1.jpg]

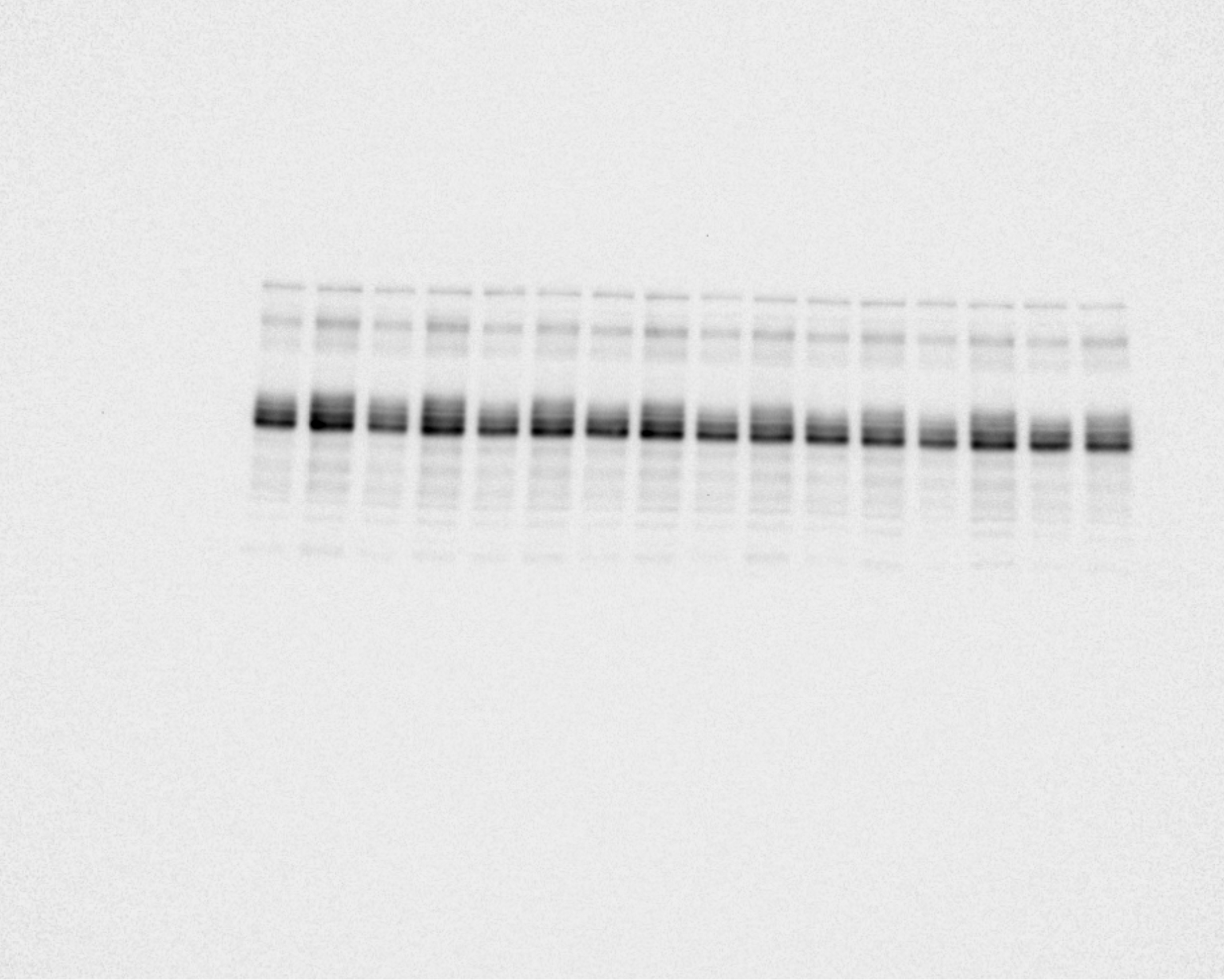

Supplement: Figure 6—figure supplement 1—source data 1. [file elife-83159-fig6-figsupp1-data1.zip › HA-TTP Figure 6-figure supplement 1-source data 1/Versteeg 2022-12-22 11h40m52s 56.737s(Chemiluminescence).jpg]

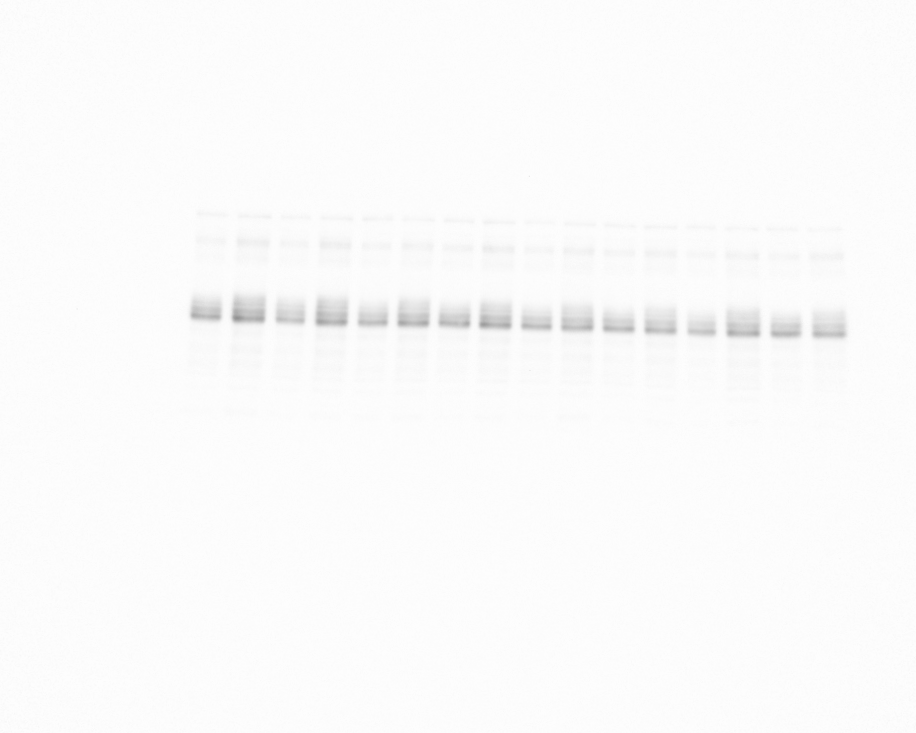

Supplement: Figure 6—figure supplement 1—source data 1. [file elife-83159-fig6-figsupp1-data1.zip › HA-TTP Figure 6-figure supplement 1-source data 1/Versteeg 2022-12-22 11h40m52s 56.737s(Chemiluminescence).raw16.tif]

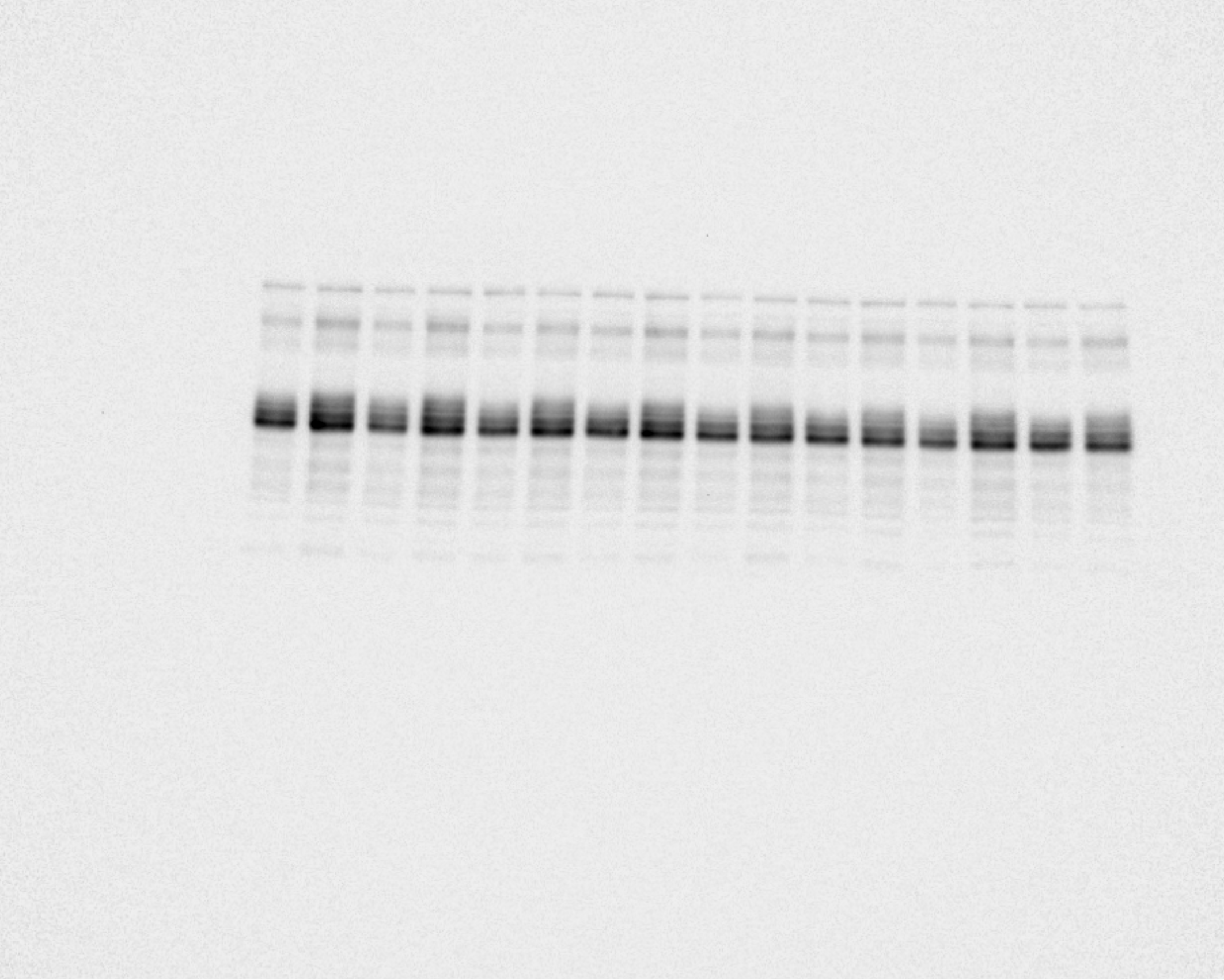

Supplement: Figure 6—figure supplement 1—source data 1. [file elife-83159-fig6-figsupp1-data1.zip › HA-TTP Figure 6-figure supplement 1-source data 1/Versteeg 2022-12-22 11h40m52s 56.737s(Chemiluminescence).tif]

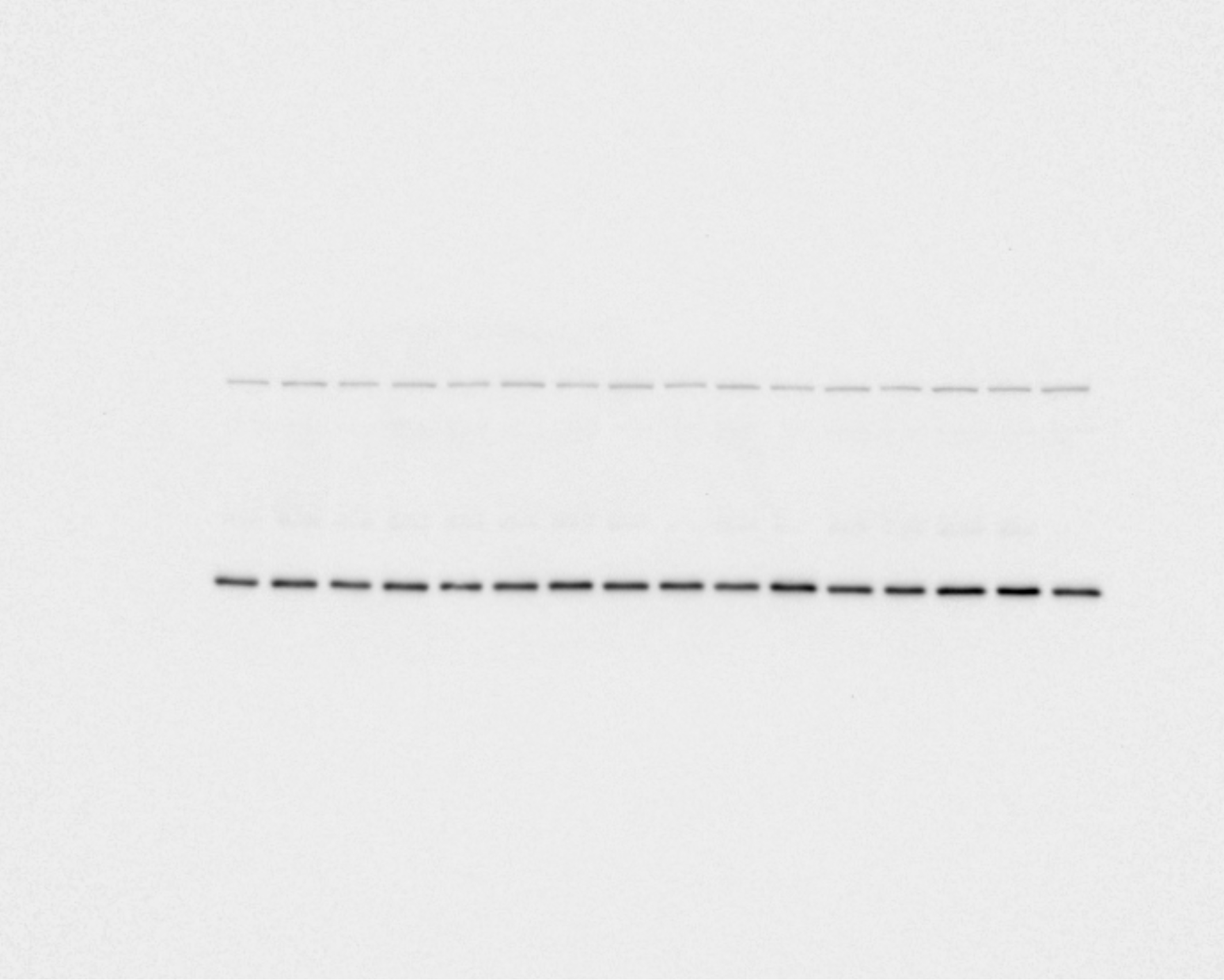

Supplement: Figure 6—figure supplement 1—source data 1. [file elife-83159-fig6-figsupp1-data1.zip › mCherry Figure 6-figure supplement 1-source data 1/Versteeg 2022-12-22 14h58m40s 29.660s(Chemiluminescence).jpg]

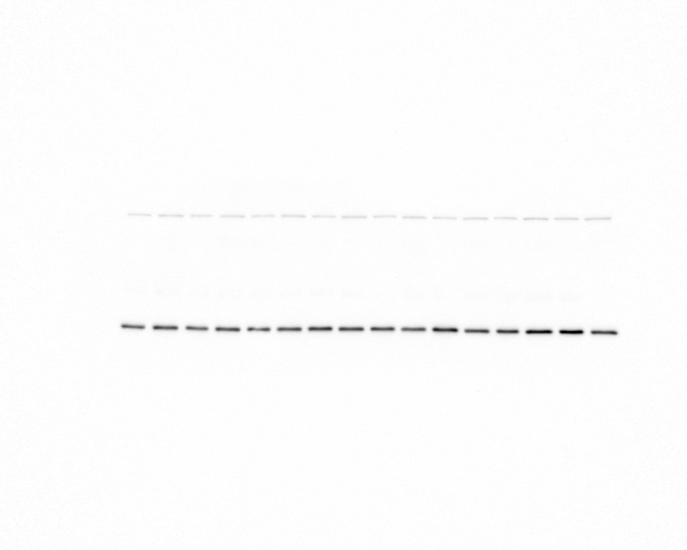

Supplement: Figure 6—figure supplement 1—source data 1. [file elife-83159-fig6-figsupp1-data1.zip › mCherry Figure 6-figure supplement 1-source data 1/Versteeg 2022-12-22 14h58m40s 29.660s(Chemiluminescence).raw16.tif]

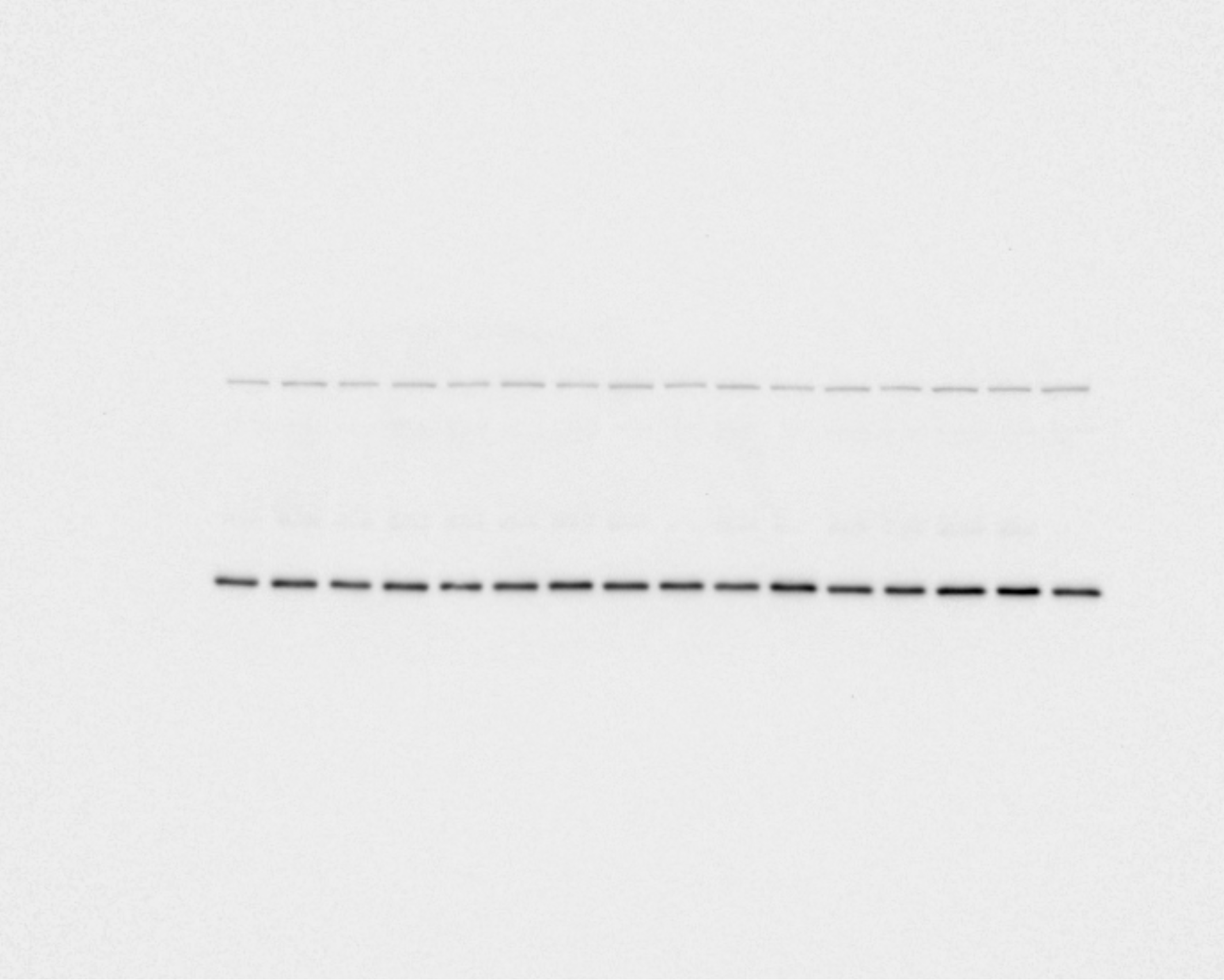

Supplement: Figure 6—figure supplement 1—source data 1. [file elife-83159-fig6-figsupp1-data1.zip › mCherry Figure 6-figure supplement 1-source data 1/Versteeg 2022-12-22 14h58m40s 29.660s(Chemiluminescence).tif]

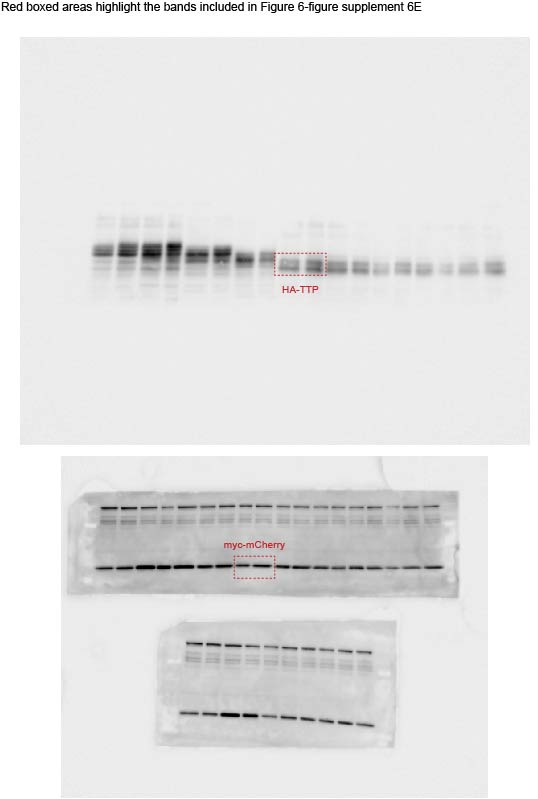

Supplement: Figure 6—figure supplement 1—source data 2. [file elife-83159-fig6-figsupp1-data2.zip › Figure 6-figure supplement 1-source data 2.jpg]

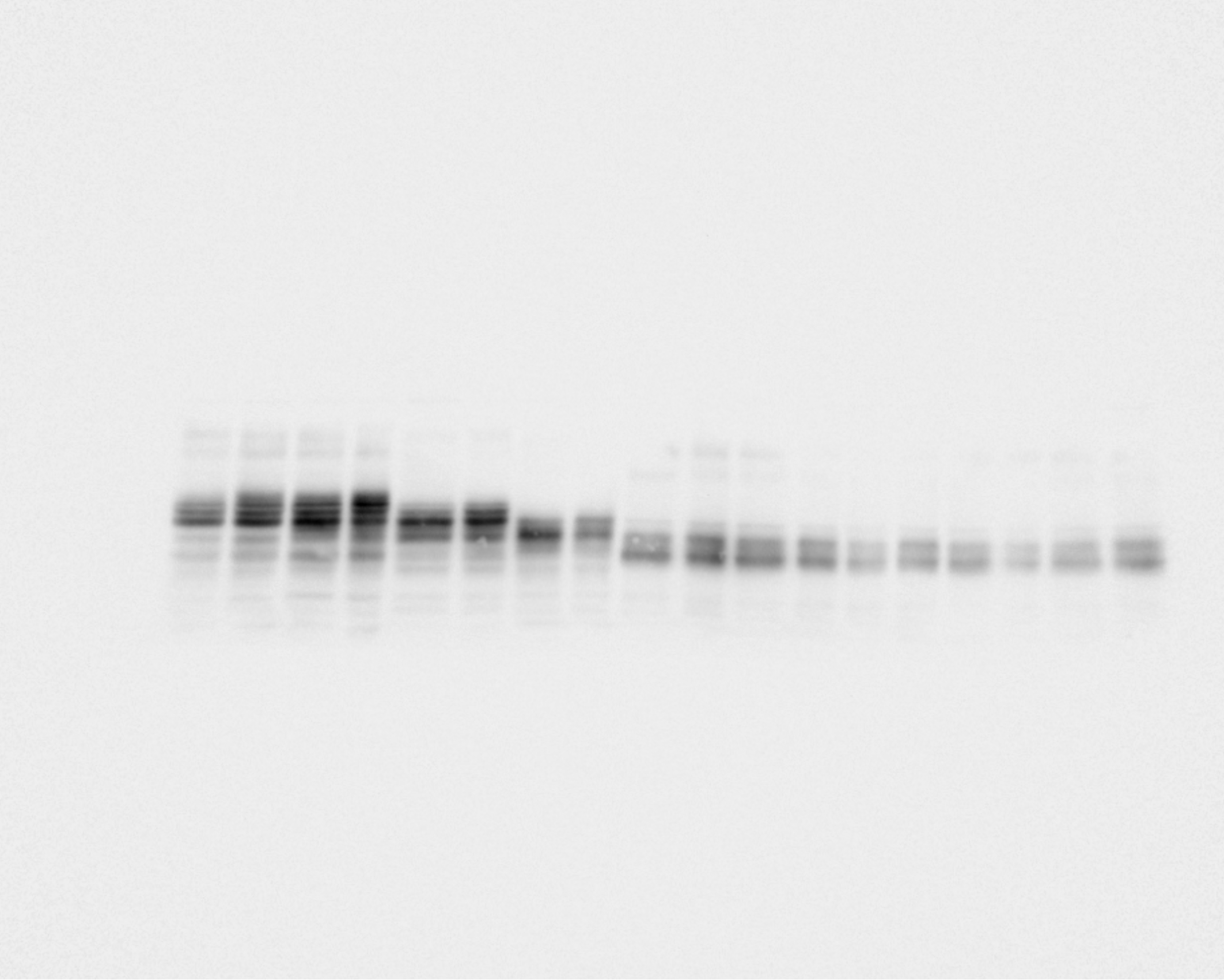

Supplement: Figure 6—figure supplement 1—source data 2. [file elife-83159-fig6-figsupp1-data2.zip › HA-TTP Figure 6-figure supplement 1-source data 2/Versteeg 2023-01-27 16h05m46s 13.132s(Chemiluminescence).jpg]

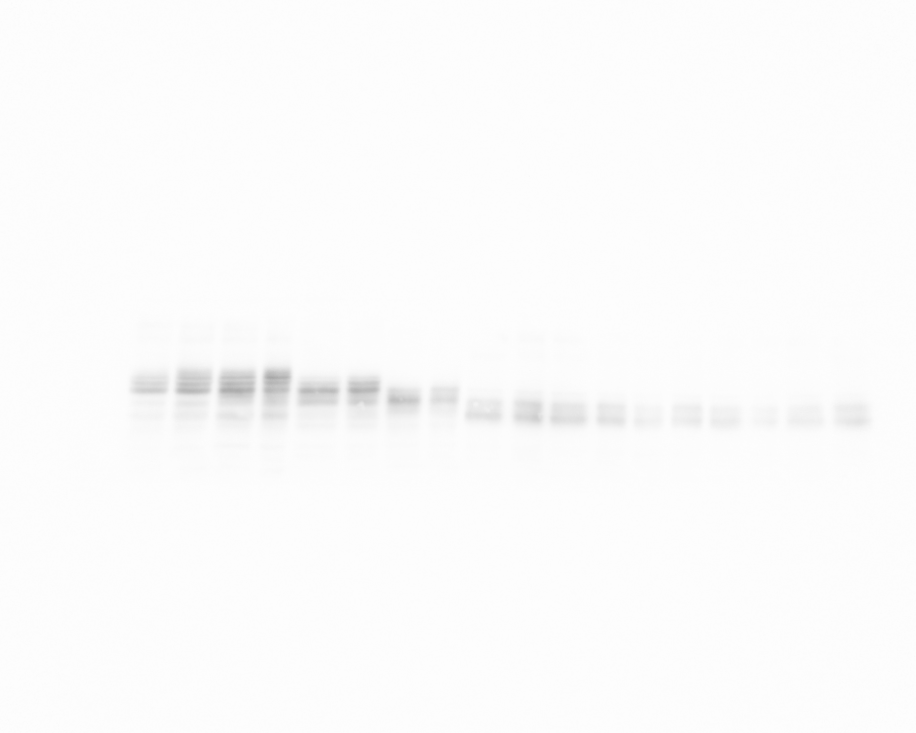

Supplement: Figure 6—figure supplement 1—source data 2. [file elife-83159-fig6-figsupp1-data2.zip › HA-TTP Figure 6-figure supplement 1-source data 2/Versteeg 2023-01-27 16h05m46s 13.132s(Chemiluminescence).raw16.tif]

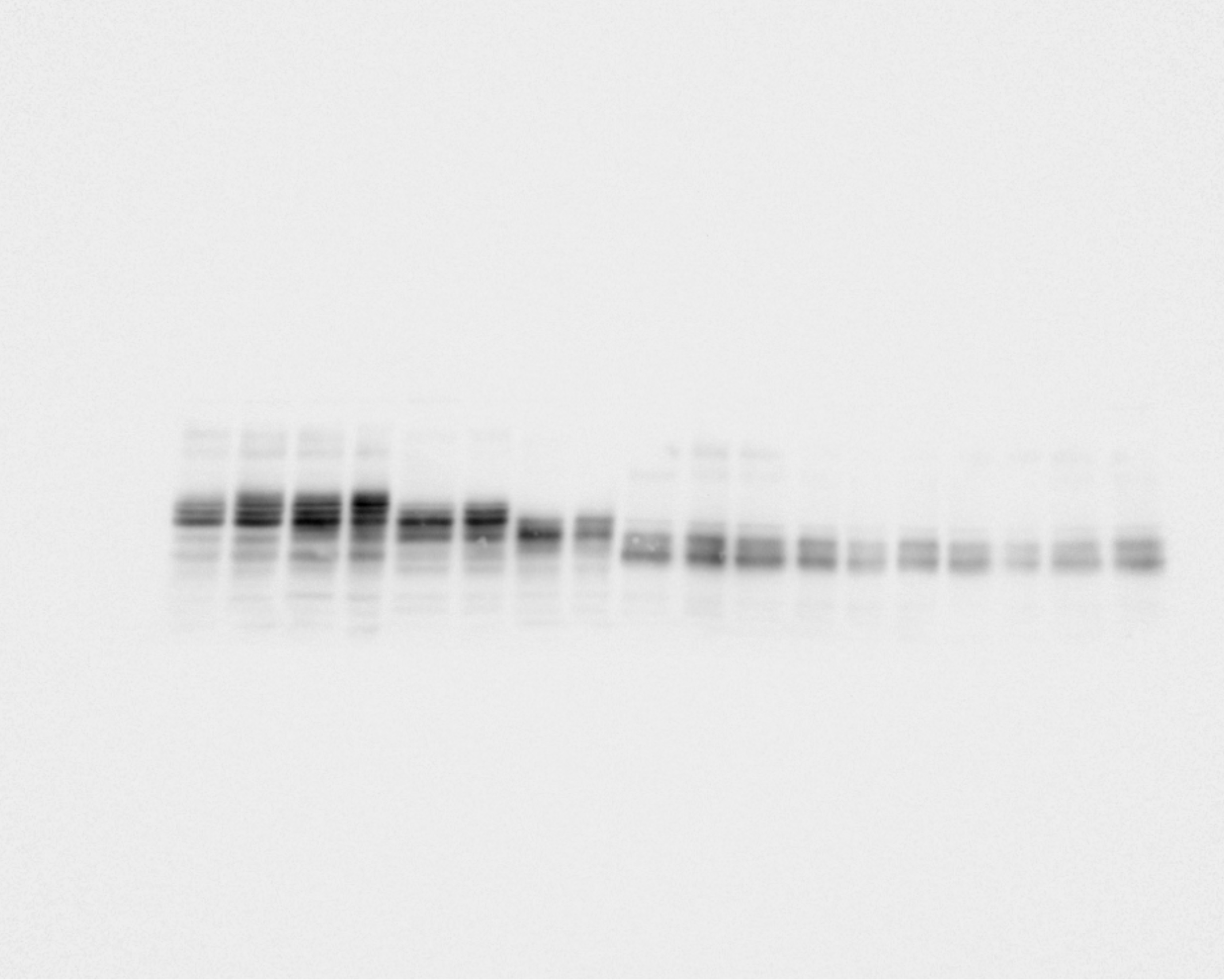

Supplement: Figure 6—figure supplement 1—source data 2. [file elife-83159-fig6-figsupp1-data2.zip › HA-TTP Figure 6-figure supplement 1-source data 2/Versteeg 2023-01-27 16h05m46s 13.132s(Chemiluminescence).tif]

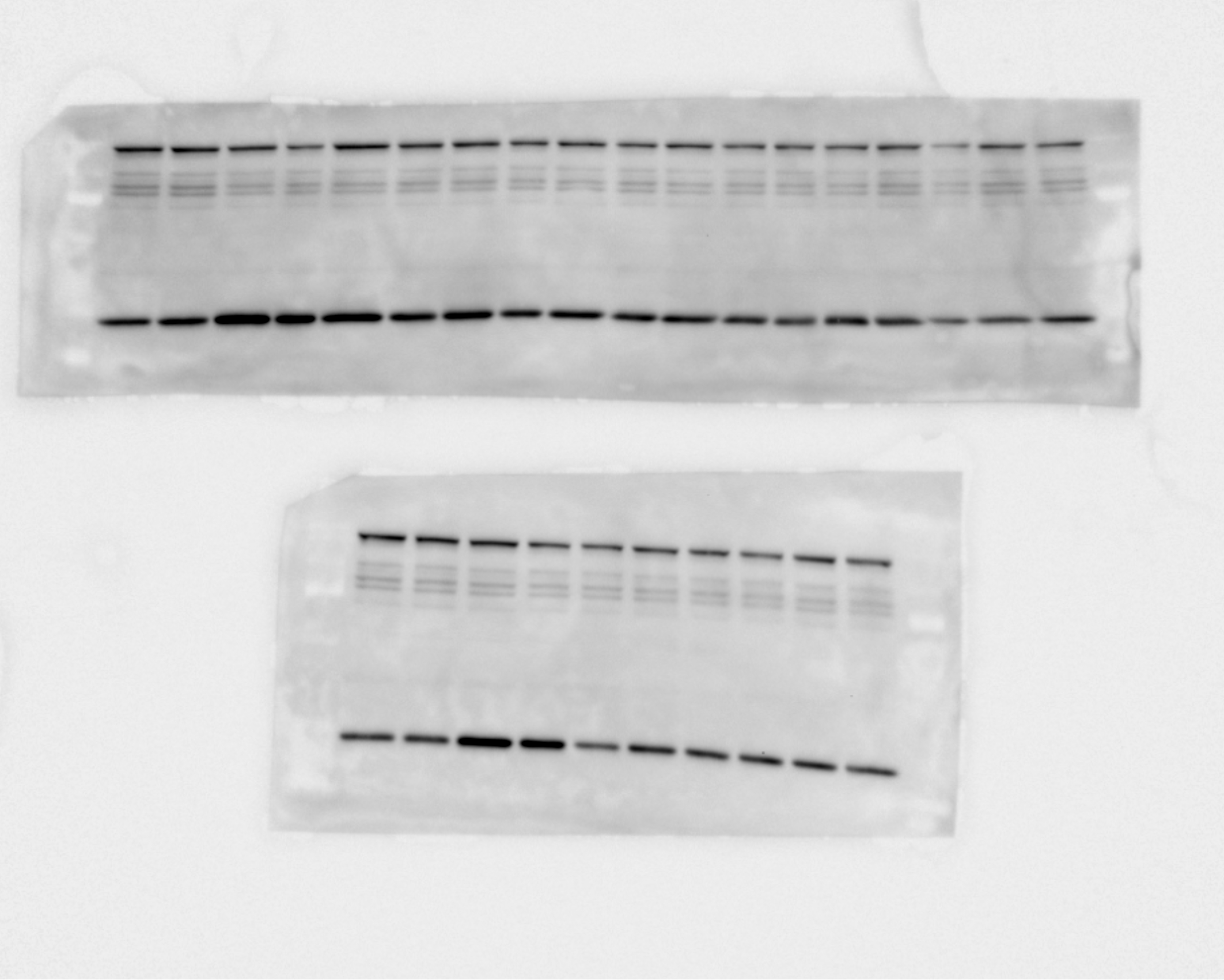

Supplement: Figure 6—figure supplement 1—source data 2. [file elife-83159-fig6-figsupp1-data2.zip › mCherry Figure 6-figure supplement 1-source data 2/Versteeg 2023-01-27 18h07m17s 23.102s(Chemiluminescence).jpg]

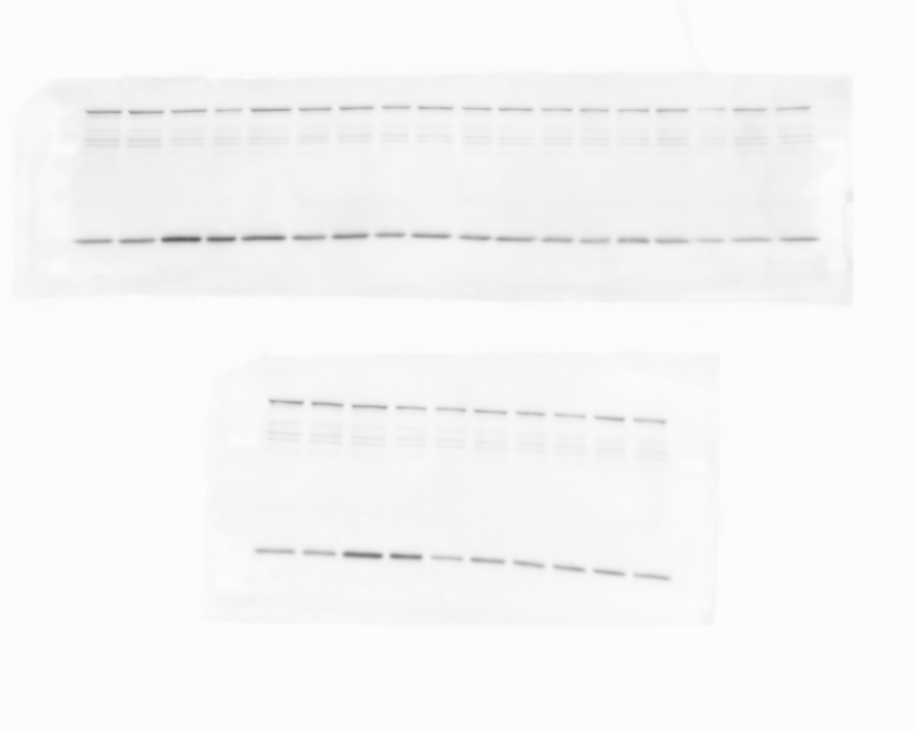

Supplement: Figure 6—figure supplement 1—source data 2. [file elife-83159-fig6-figsupp1-data2.zip › mCherry Figure 6-figure supplement 1-source data 2/Versteeg 2023-01-27 18h07m17s 23.102s(Chemiluminescence).raw16.tif]

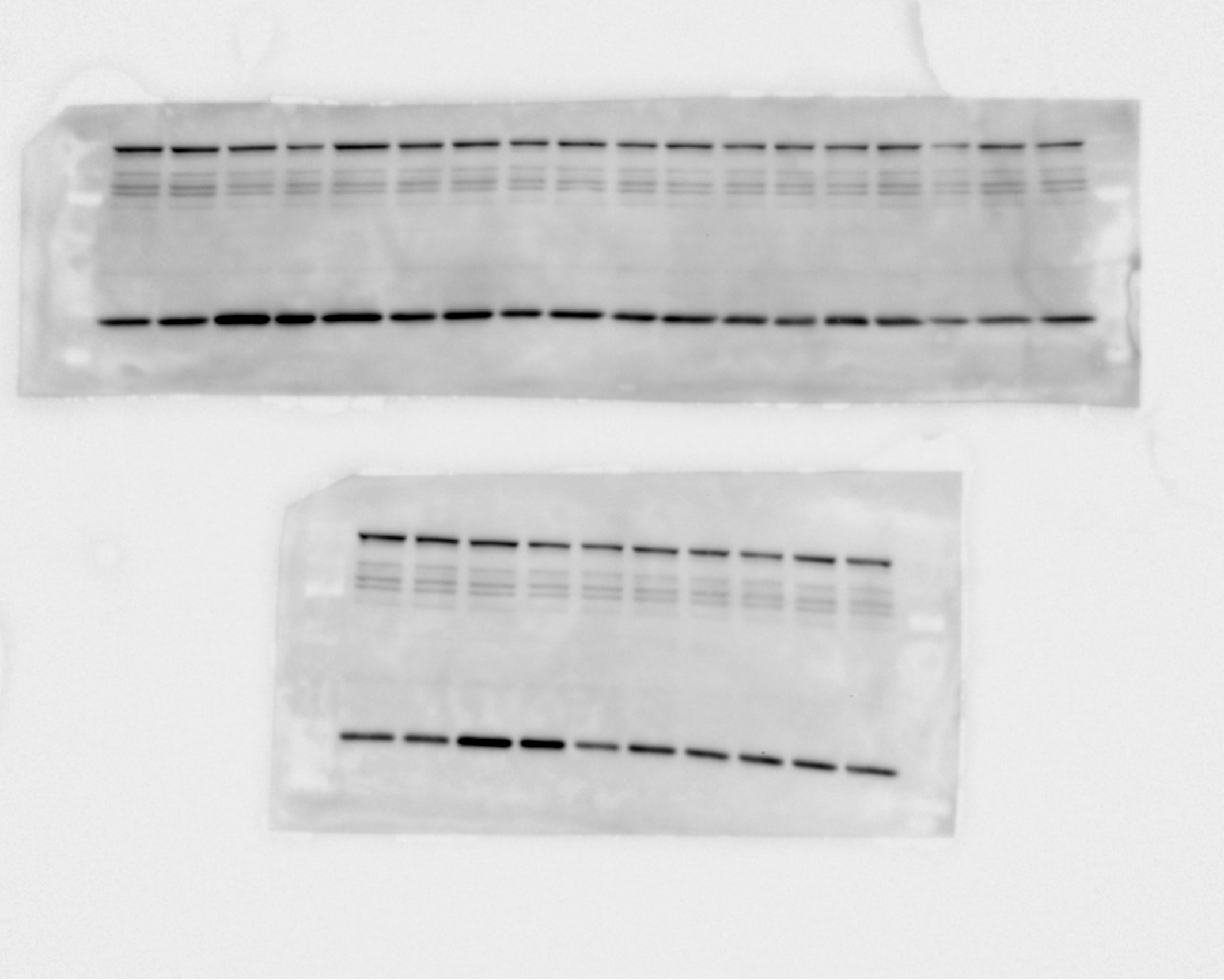

Supplement: Figure 6—figure supplement 1—source data 2. [file elife-83159-fig6-figsupp1-data2.zip › mCherry Figure 6-figure supplement 1-source data 2/Versteeg 2023-01-27 18h07m17s 23.102s(Chemiluminescence).tif]
